# Identification of the meiotic toolkit in diatoms and exploration of meiosis-specific *SPO11* and *RAD51* homologs in the sexual species *Pseudo-nitzschia multistriata* and *Seminavis robusta*

**DOI:** 10.1186/s12864-015-1983-5

**Published:** 2015-11-14

**Authors:** Shrikant Patil, Sara Moeys, Peter von Dassow, Marie J. J. Huysman, Daniel Mapleson, Lieven De Veylder, Remo Sanges, Wim Vyverman, Marina Montresor, Maria Immacolata Ferrante

**Affiliations:** Stazione Zoologica Anton Dohrn, Villa Comunale 1, 80121 Naples, Italy; Department of Biology, Protistology and Aquatic Ecology, Ghent University, 9000 Ghent, Belgium; Department of Plant Systems Biology, Flanders Institute for Biotechnology (VIB), 9052 Ghent, Belgium; Department of Plant Biotechnology and Bioinformatics, Ghent University, 9052 Ghent, Belgium; Facultad de Ciencias Biológicas, Instituto Milenio de Oceanografía, Pontificia Universidad Católica de Chile, Santiago, Chile; UMI 3614, Evolutionary Biology and Ecology of Algae, CNRS-UPMC Sorbonne Universités, PUCCh, UACH, Station Biologique de Roscoff, Roscoff, France; The Genome Analysis Centre (TGAC), Norwich Research Park, Norwich, NR4 7UH UK

**Keywords:** Meiosis, Diatoms, Sexual reproduction, *SPO11*, *RAD51*

## Abstract

**Background:**

Sexual reproduction is an obligate phase in the life cycle of most eukaryotes. Meiosis varies among organisms, which is reflected by the variability of the gene set associated to the process. Diatoms are unicellular organisms that belong to the stramenopile clade and have unique life cycles that can include a sexual phase.

**Results:**

The exploration of five diatom genomes and one diatom transcriptome led to the identification of 42 genes potentially involved in meiosis. While these include the majority of known meiosis-related genes, several meiosis-specific genes, including *DMC1,* could not be identified. Furthermore, phylogenetic analyses supported gene identification and revealed ancestral loss and recent expansion in the *RAD51* family in diatoms. The two sexual species *Pseudo-nitzschia multistriata* and *Seminavis robusta* were used to explore the expression of meiosis-related genes: *RAD21, SPO11-2, RAD51-A, RAD51-B* and *RAD51-C* were upregulated during meiosis, whereas other paralogs in these families showed no differential expression patterns, suggesting that they may play a role during vegetative divisions. An almost identical toolkit is shared among *Pseudo-nitzschia multiseries* and *Fragilariopsis cylindrus*, as well as two species for which sex has not been observed, *Phaeodactylum tricornutum* and *Thalassiosira pseudonana*, suggesting that these two may retain a facultative sexual phase.

**Conclusions:**

Our results reveal the conserved meiotic toolkit in six diatom species and indicate that Stramenopiles share major modifications of canonical meiosis processes ancestral to eukaryotes, with important divergences in each Kingdom.

**Electronic supplementary material:**

The online version of this article (doi:10.1186/s12864-015-1983-5) contains supplementary material, which is available to authorized users.

## Background

The process of sexual reproduction is a hallmark for all the major eukaryotic groups [1–3]. It is believed that all asexual eukaryotes have evolved from sexual ancestors [1, 4], and it has been proposed that the last eukaryotic common ancestor (LECA) possessed the full set of genes known to be involved in meiosis [5–8]. Meiosis is not performed exactly in the same way in different groups: differences can be found for instance in the mechanisms of crossover formation and in the structure of the synaptonemal complex, and these differences are reflected in a variable set of meiosis-related genes [9].

Our understanding of the meiotic process, and consequently our knowledge of the gene repertoire required, is relatively strong for Opisthokonta and plants. However, despite the fact that a number of publications have recently appeared on a variety of unicellular organisms [5, 6, 9–11], information on most groups that contribute to the greater part of eukaryotic diversity are still scarce.

Among aquatic protists, diatoms are an important group of microalgae, as they are one of the major primary producers in freshwater and marine ecosystems [12, 13] and often dominate planktonic and benthic microalgal assemblages. They are a relatively recent lineage of unicellular organisms belonging to the SAR (Stramenopiles, Alveolata, Rhizaria) supergroup [14]. The Stramenopiles include both unicellular and multicellular members, as well as phototrophic, heterotrophic and parasitic members. Diatoms are the only free-living unicellular Stramenopiles for which the sexual cycle can be fully controlled in the laboratory for some species [15].

Diatoms are divided in two major groups, centrics, with radial symmetry, and pennates, with bilateral symmetry. They are unique among unicellular eukaryotes as they are encased in intricately patterned siliceous cell walls, consisting of two halves (thecae) of which one (the epitheca) is slightly larger than the other (the hypotheca). Diatoms spend the majority of their life cycle as diploid cells and multiply by mitotic divisions. Repeated cell divisions are, however, constrained by the inflexible arrangement of the silica wall. As a consequence of this rigid structure, diatom cells progressively decrease in size after cell division, which may lead to cell death and extinction of the clonal lineage unless large cell size is restored [16]. Although some species have been shown to employ alternative routes to escape the progressive cell miniaturization, the majority of diatom species restore cell size via sexual reproduction [15]. Thus, besides the fundamental goal of sexual reproduction to generate genetic diversity within a population, in diatoms the sexual phase also plays a key role in cell size restitution [15, 16].

The best studied model diatoms are *Thalassiosira pseudonana* and *Phaeodactylum tricornutum*, for which genome sequences are available [17, 18], and gene function can be studied with conventional tools for manipulation [19–21]. In contrast to most diatoms [22], sexual stages have never been observed for these two species, and laboratory strains do not reduce in size and only reproduce vegetatively. This has imposed a limitation for the study of processes related to sexual reproduction, well-documented in other diatoms [15]. The few examples of molecular studies include the identification, in *Thalassiosira weissflogii*, of sex-induced genes (*SIG*) reported to contain epidermal growth factor (EGF)-like domains, putatively encoding for components of stramenopile mastigonemes [23], and investigations of the genetic basis for sex determination, in *Seminavis robusta*, by linkage mapping [24].

The rapid increase in molecular data for unicellular eukaryotes has made it possible to perform comparative genomic studies to search for genes involved specifically in meiosis, allowing to assess the evolutionary history of the molecular mechanisms underlying the sexual phase. The “meiosis detection toolkit” approach provided evidence for the conservation of several of these genes in eukaryotes [5, 6, 10, 25]. The inventory of meiotic genes includes both genes that are known to play meiosis-specific roles and genes that are required for meiosis, but whose expression and functions are shared with non-meiotic processes (Table 1). Mutations in the first category of genes result in severe disruptions specific to meiosis, without documented effects on non-meiotic functions [26, 27]. Examples of genes included in the second category are genes related to DNA replication, maintenance of chromosome structure (e.g. *MCM* [28], *SMC* [29] and *RAD21* [30]), as well as genes related to DNA repair (homologs of *RAD51* [31], homologs of the bacterial *MutS* and *MutL* genes [32]). However, it has been shown that some genes thought to be meiosis-specific may also be conserved in parthenogenetic organisms. The expression of *SPO11*, the gene responsible for the creation of double strand breaks (DSBs) in homologous chromosomes, and other meiotic genes, was detected in both cyclical and obligate parthenogenetic monogonont rotifers [33] and during parthenogenesis in the microcrustacean *Daphnia pulex* [25]. *SPO11* in *Candida albicans*, and *SPO11*, *HOP1* and *DMC1* in *Giardia intestinalis*, have been shown to function during parasexual genetic recombination [34, 35]. Likewise, in the haptophyte *Emiliania huxleyi*, genotypes that appear to have lost the ability to form haploid stages still retain *SPO11*, *DMC1*, and *RAD51* [36]. Thus, determining how the meiotic toolkit has been conserved in different eukaryotic lineages requires comparison of representatives for which sex and meiosis can be directly observed.

**Table 1:** Functional roles of meiotic genes searched in the diatom genomes

Protein	Role in meiosis
DNA replication and chromosome maintenance	
Mcm2-7	Mcm2-7 form hexamer and are involved in DNA replication [28]
Mcm8	Mcm8 and Mcm9 are involved in meiotic recombination [81, 83, 84]
Mcm9	Mcm8 and Mcm9 are involved in meiotic recombination [83, 84]
Smc1-Smc3	Part of sister chromatids cohesin subunit, act as a heterodimer
Smc2-Smc4	Heterodimer, essential for chromosome assembly and segregation, part of core condensing subunits
Smc5-Smc6	Heterodimer, involved in DNA repair and checkpoint response, binds to single stranded DNA (ssDNA)
Pds5	Involved in maintenance of sister chromatid cohesion in late prophase
Scc3	Interacts with cohesin complex Smc1-Smc3 and Rad21/Rec8 and helps in holding cohesin ring together
Rec8/Syn1^a^	Meiotic homolog of Rad21, involved in holding sister chromatids together during meiotic recombination
Rad21	Holds Smc1 and Smc3 together thus holding sister chromatids together during meiosis and mitosis
DNA double strand break formation	
Spo11-1^a^	Creates double strand breaks (DSBs) in homologous chromosomes in meiotic recombination
Spo11-2^a^	Creates DSBs in homologous chromosomes in meiotic recombination in plants
Spo11-3/Top VIA	Required for endoreduplication of DNA
DNA damage sensing and response	
Rad50	DNA binding ATPase, holds broken DNA strands while Mre11 trims DSBs
Mre11	3′–5′ dsDNA exonuclease and ssDNA endonuclease; trims broken DNA ends after DSBs and hairpins
Xrs2/Nbs1	Component of Mre11-Rad50-Xrs2, involved in homologous recombination and non-homologous end joining [125]
Crossover regulation	
Mer3^a^	DNA helicase that unwinds double stranded DNA during meiotic cross over formation [126]
Hop1^a^	Binds to DSBs, component of lateral and axial synaptonemal complex
Red1/Asy3^a^	Component of synaptonemal complex, interacts with Hop1 and facilitates meiosis I chromosome disjunction [48, 49]
Zip1/Zyp1^a^	Transverse filament protein involved in synaptonemal complex formation during meiosis [45, 47]
Zip2^a^,Zip3/Hei10^a^, Zip4^a^	Components of synaptonemal complex required for morphogenesis of the synaptonemal complex [45, 64]
Dmc1^a^	Meiotic member of Rad51-RadA-RecA superfamily of proteins, binds to ssDNA end of DSBs and is involved in inter-homologous recombination
Hop2^a^	Homology search together with Mnd1, works in Dmc1 dependent homology search pathway downstream of Rad51
Mnd1^a^	Together with Hop2 works in homology searching and is also required in stable DNA heteroduplex
Msh4^a^-Msh5^a^	Heterodimer, together with Mlh1/Mhl3 heterodimer directs Holliday junction resolution with crossover interference
Double-strand break repair (recombinational repair)	
Rad51, Xrcc2, Xrcc3	Mediate homologous pairing and strand invasion, involved in DNA repair mechanisms in mitosis and meiosis. Part of Rad51-RadA-RecA superfamily, exhibit multiple paralogs
Rad52	Binds to ssDNA and initiates homologous recombination, stimulates Rad51 mediated strand invasion
Rad1	5′-3′ endonuclease, required in meiotic crossing over, functions during nucleotide excision repair
Msh2	Forms heterodimer with Msh3 or Msh6, works in DNA mismatch repair
Msh6	Forms heterodimer with Msh2, works in DNA mismatch repair
Mlh1	DNA mismatch repair protein, forms heterodimers with Mlh2, Mlh3 and Pms1, interacts with Msh2/Msh6 and Msh4/Msh5
Mlh2	DNA mismatch repair protein, forms heterodimer with Mlh1
Mlh3	Forms heterodimer with Mlh1, interacts with Msh4/Msh5 to promote meiotic crossovers
Pms1	Forms heterodimer with Mlh1, involved in DNA mismatch repair
Mms4/Eme1	Interacts with Mus81 and is involved in interference insensitive, class II crossovers during meiotic recombination [104]
Mus81	Interacts with Mms4 and is involved in interference insensitive, class II crossovers during meiotic recombination [104]
Fancm	DNA helicase required for genome stability, involved in limiting meiotic crossovers [127]
Other accessory proteins required during meiosis	
Fen1	Fen1 functions during homologous recombination mediated DNA repair by removing divergent sequences at DNA break ends [128]
Exo1	A double-stranded DNA-specific 5′–3′ exonuclease [129]
Dna2	A conserved DNA nuclease involved in DNA stability [130]
Brca1	Regulates meiotic spindle assembly [131]
Brca2	Involved in DNA damage-induced Rad51 foci formation during meiosis [132]

Gene functions are taken from Malik et al. [6] and from Hanson et al. [33] unless otherwise mentioned. Genes marked with ^a^ are considered meiosis-specific genes and do not have known functions outside of meiosis

The *T. pseudonana* genome was included in a study assessing phylogenetic distribution of core meiotic proteins [6], however, as mentioned above, this species is currently considered asexual, and further datasets have become available for other species for which sexual reproduction can be controlled in the laboratory, such as members of the *Pseudo-nitzschia* genus [37, 38].

With the aim to improve the definition of the meiosis toolkit for diatoms, we assembled an expanded list of meiotic genes for eukaryotes [6, 9, 33] and looked for the presence of homologs in five diatom genomes and in transcriptome sequence data. We produced an inventory of putative meiotic genes and combined this information with gene expression data for two sexually reproducing species, demonstrating that their expression is indeed increased during sexual reproduction. Phylogenetic analyses for these genes revealed the presence of multiple paralogs for the *RAD51* family, the presence of two diatom homologs of *SPO11* and the presence of a single *RAD21* gene.

## Results

### Identification of meiotic genes in diatoms

Homology searches for 60 meiotic proteins [6, 9] were performed in five diatom genomes, those of *Thalassiosira pseudonana,* a centric species*, Phaeodactylum tricornutum, Fragilariopsis cylindrus, Pseudo-nitzschia multiseries* and *Pseudo-nitzschia multistriata*, and in the *de novo* transcriptome of *Seminavis robusta* (Table 2), all pennate species. The reference transcriptome for the latter species was produced using data from vegetatively as well as sexually reproducing samples.

**Table 2:** Protein, gene model or transcript IDs for the genes involved in meiosis analyzed in this study

Protein name	Accession numbers of proteins used as query	*Thalassiosira pseudonana* protein ID	*Phaeodactylum tricornutum* protein ID	*Fragilariopsis cylindrus* protein ID	*Pseudo-nitzschia multiseries* protein ID	*Pseudo-nitzschia multistriata* gene model ID	*Seminavis robusta* Transcript ID
DNA replication and chromosome maintenance							
Mcm2	NP_001185154.1	29936	18622	204899	209470	0061270.1	Semro_comp78811_c0_seq1
Mcm3	Q9FL33.1	34975	51597	264318	318351	0004850.1	Semro_comp50104_c0_seq1
Mcm4	NP_179236.3	269123	51412	146869	203268	0078600.1^b^	Semro_comp82592_c0_seq1
Mcm5	NP_001189521.1	31609	11490	224321	255529	0118810.1	Semro_comp83065_c1_seq1
Mcm6	AED95141.1	26545	468	193082	321321	0022580.1	Semro_comp61600_c0_seq1
Mcm7	P43299.2	262526	13243	184349	243980	0109820.1	Semro_comp70058_c0_seq2
Mcm8	NP_187577.1	261512	52561	189062	213178	0068370.1	Semro_comp79168_c0_seq3
Mcm9	NP_179021.3	37362	981	156569	183315	0056900.1	Semro_comp59174_c0_seq1
Smc1	AEE79265.1	35499	25506	212269	162817	0116990.1	Semro_comp83927_c0_seq1
Smc2	NP_201047.1	1393	30352	210755	191984	0096310.1	Semro_comp61213_c0_seq1
Smc3	NP_180285.4	259020	52607	208027	251818	0079810.1	Semro_comp78328_c0_seq1
Smc4	AED95695.1	42365	44165	212991	144962	0030660.1	Semro_comp76089_c0_seq1
Smc5	AED92224.1	9851	54192	193562	286374	0102810.1	Semro_comp64598_c0_seq1
Smc6	NP_196383.1	1743	36853	177172	165557	0090050.1	Semro_comp65344_c0_seq1
Pds5	NP_177883.5	5929	1590	136077	203285	0089060.1	Semro_comp82484_c0_seq6
Scc3	AEC10920.1	8747	51870	234878	38865	0079350.1	Semro_comp20575_c0_seq1
Rec8/Syn1^a^	NP_196168.1	NF	NF	NF	NF	NF	NF
Rad21	NP_851110.1	8557	44595	245879	324402	0072170.1	Semro_comp80503_c0_seq6
Double-strand break formation							
Spo11-1^a^	AEE75304.1	NF	NF	NF	NF	NF	NF
Spo11-2^a^	AEE34178.1	263510	36531	242364	156625	0108120.1^b^	Semro_comp74200_c0_seq1
Spo11-3/Top VIA	NP_195902.1	42646	24838	239125	251788	0081370.1	Semro_comp59497_c0_seq2
DNA damage sensing and response							
Rad50	AEC08614.1	9195	51876	243939	320939	0001820.1	Semro_comp61512_c0_seq1
Mre11	NP_200237.1	34332	54699	275781	233741	0086370.1	Semro_comp82091_c0_seq3
Xrs2/Nbs1	ABA54896.1	NF	NF	NF	NF	NF	NF
Crossover regulation							
Mer3^a^	AAX14498.1	11979	39994	239915	285411	0087420.1	Semro_comp60890_c0_seq1
Hop1/Asy1^a^	AEE34638.1	NF	NF	NF	NF	NF	NF
Red1/Asy3^a^	AEC10782.1	NF	NF	NF	NF	NF	NF
Zip1/Zyp1^a^	AEE30217.1	NF	NF	NF	NF	NF	NF
Zip2^a^	**NP_011265.1**	NF	NF	NF	NF	NF	NF
Zip3/Hei10^a^	NP_175754.2	NF	NF	NF	NF	NF	NF
Zip4^a^	ABO71664.1	NF	NF	NF	NF	NF	NF
Dmc1^a^	AAC49617.1	NF	NF	NF	NF	NF	NF
Hop2^a^	CAF28783.1	NF	NF	NF	NF	NF	NF
Mnd1^a^	NP_194646.2	25513	54296	273989	295346	0080640.1	Semro_comp20014_c0_seq1
Msh4^a^	AAT70180.1	261368	51916	144820	259109	0116300.1^b^	Semro_comp57561_c0_seq2
Msh5^a^	NP_188683.3	16039	52173	149505	183820	0023810.1	Semro_comp80580_c0_seq6
Double-strand break repair (recombinational repair)							
Rad51-A	BAE99388.1	261303	51999	165795 (A1) 197408 (A2)	212272 (A1) 1352 (A2)	0056780.1 (A1) 0086180.1 (A2)	Semro_comp76648_c0_seq1
Rad51-B	NP_180423.3	261577	40092	241710	325273	0105810.1	Semro_comp71219_c0_seq1
Rad51-C	CAC14091.1	257784^b^	54137	201530	29459	0104040.1	Semro_comp71710_c0_seq3
Rad51-D	NP_001077479.1	NF	NF	NF	NF	NF	NF
Xrcc2	NP_201257.2	NF	NF	NF	NF	NF	NF
Xrcc3	NP_200554.1	2081^c^	31781	242664^c^	292867^c^	comp26486_c0_seq1^c^	Semro_comp70556_c0_seq2^c^
Rec-A	BAE99388.1	267595	51425	186275	166360	0063260.1	Semro_comp77000_c0_seq1
Rad52	**CAA86623.1**	25447	49083	238228	50181	0088620.1	Semro_comp79910_c0_seq1
Rad1	Q9LKI5.2	22869	30908	208467	230429	0074360.1^b^	Semro_comp82187_c0_seq2
Msh2	AEE76112.1	32661	19604	159571	153636	0083140.1	Semro_comp80478_c0_seq1
Msh6	NP_001190656.1	261781	53969	212924	190397	0084190.1	Semro_comp80580_c0_seq6
Mlh1	NP_567345.2	263509	54331	136590	257081	0125040.1	Semro_comp75421_c0_seq1
Mlh2	**NP 013135.1**	NF	NF	NF	NF	NF	NF
Mlh3	NP_195277.5	NF	NF	NF	NF	NF	NF
Pms1	AAM00563.1	264783	14607	248102	242883	0117080.1	Semro_comp73186_c0_seq1
Mms4/Eme1	**AAF06816.1**	NF	NF	NF	NF	NF	NF
Mus81	NP_194816.2	NF	36625	241086	63674	0100930.1^b^	Semro_comp84506_c0_seq2
Fancm	NP_001185141.1	11922	47619	248113	68428	0010100.1	Semro_comp74927_c0_seq1^c^
Accessory proteins required during meiosis							
Fen1	AED93576.1	269347	48638	206746	260195	0006800.1	Semro_comp51200_c0_seq1
Exo1	Q8L6Z7.2	4742	48206	261553	110816	0067080.1	Semro_comp61722_c0_seq1
Dna2	NP_001184943.1	10652	35426	241656	326992	0027070.1	Semro_comp83726_c0_seq1
Brca1	AAO39850.1	NF	NF	NF	NF	NF	NF
Brca2	AEE81814.1	6763	36784	253990	284242	0067160.1	Semro_comp82255_c0_seq4

Genes marked with ^a^ are genes that do not have known functions outside meiosis. *Arabidopsis thaliana* meiotic proteins were used as query sequence; whenever the specific query gene was not found/present in *A. thaliana*, *Saccharomyces cerevisiae* proteins were used (accession numbers in bold). Protein IDs are given for the diatom genomes available at the JGI portal, gene models IDs are given for the *Pseudo-nitzschia multistriata* genome and transcripts IDs for *Seminavis robusta*. Actual gene model IDs for *P. multistriata* include the prefix PSNMU-V1.4_AUG-EV-PASAV3

^b^corrected gene model, *NF* not found, ^c^gene model might need validation

Of the 60 meiosis-related genes known to play roles in DNA duplication, chromosome maintenance and stability, and DNA repair, 42 were found to be present in all diatom genomes, with the exception of *MUS81* endonuclease, which could not be found in the *T. pseudonana* genome. Of the 15 genes known to be exclusive to meiosis (marked with an "a" in Tables 1 and 2), five were detected in all the diatom genomes and the transcriptome surveyed (Table 2)*.* These genes include *SPO11-2*, a meiosis-specific gene required for the formation of double-strand breaks (DSBs) in paired chromosome homologs and highly conserved throughout eukaryotic lineages [39, 40]. The other four genes include *MND1,* whose protein product forms a heterodimer with Hop2 and facilitates Dmc1 dependent crossover formation [41, 42], *MSH4* and *MSH5,* whose products form a complex and are thought to stabilize crossover intermediates [43, 44], and *MER3*, whose product is thought to function in the synaptonemal complex [45, 46]. The other meiosis-specific *SPO11* gene, *SPO11-1*, and nine more meiosis-specific genes, *ZIP1, ZIP2, ZIP3, ZIP4, RED1, HOP1, HOP2, DMC1* and *REC8,* could not be identified in any of the diatom genomes. Zip1-4, Red1 and Hop1 are known to be involved in formation of the synaptonemal complex [45, 47–49]. Seven other genes (*XRS2, RAD51-D, XRCC2, MLH2, MLH3, MMS4* and *BRCA1*), known to function during DNA damage repair, were not detected in our search (Table 2). In a few cases the gene models retrieved were incomplete, when possible these incorrect gene models were manually corrected (marked with "b" in Table 2).

### Phylogenetic analyses

To support the identification of diatom homologs of the meiotic genes analyzed, we created maximum likelihood phylogenetic trees for the 42 meiotic proteins. All the meiotic toolkit proteins of diatoms clustered together with significant bootstrap support and the branching confirmed relatedness with the respective homolog in other eukaryotes (Additional file 1).

**Fig. 1:**
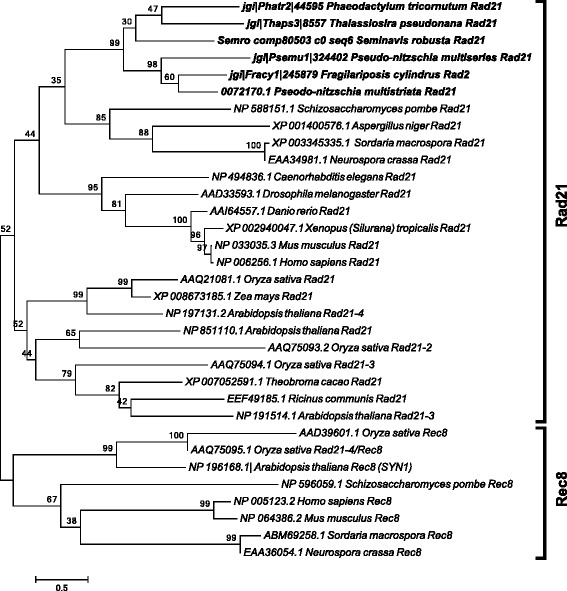
Phylogenetic tree of Rad21 proteins inferred from maximum likelihood analysis. Numbers on the branches indicate bootstrap support from 1000 replicates. Among-site substitution rate heterogeneity was corrected using two gamma-distributed substitution rate categories and WAG with frequencies (WAG + F) substitution model for amino acid substitutions. Diatom sequences are indicated in bold

The *REC-8/RAD21*, *SPO11* and *RAD51* gene families were analyzed in more detail to verify hypotheses on the putative roles of the different paralogs. *RAD21-REC8* have interchangeable roles in different organisms, *REC8* being generally required for meiosis [50, 51]. A single *RAD21-REC8* homolog was identified in each diatom genome (Table 2) and the sequences clustered with the mitotic *RAD21* from other eukaryotes (Fig. 1 and Additional file 2).

**Fig. 2:**
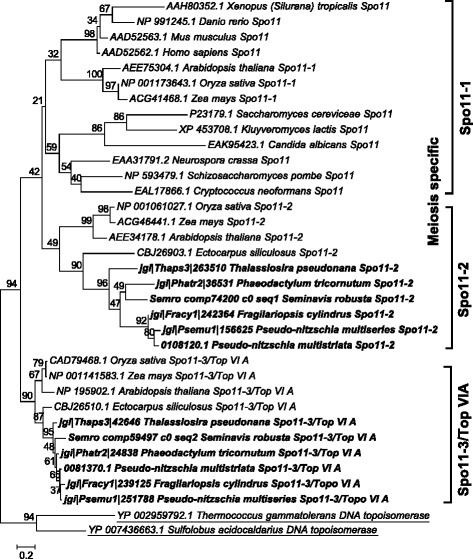
Phylogenetic tree of Spo11 proteins inferred from maximum likelihood analysis. Archaeal topoisomerase VIA protein sequences (underlined) were used as an out-group. Numbers on the branches indicate bootstrap support from 1000 replicates. Among-site substitution rate heterogeneity was corrected using two gamma-distributed substitution rate categories and LG substitution model for amino acid substitutions. Diatom sequences are indicated in bold

The *SPO11* gene family has a conserved and central role in meiotic recombination [40]. *SPO11-1* is required for meiosis in animals. In plants, *SPO11-1* and *SPO11-2* are the meiosis-specific homologs, whereas *SPO11-3/TOP VIA * is involved in vegetative growth [27, 52]. Phylogenetic analysis of *SPO11* paralogs in diatoms revealed that the two paralogs *SPO11-2* and *SPO11-3/TOP VIA * clustered closely to the respective *SPO11* homologs from plants (Fig. 2 and Additional file 3)*.*

**Fig. 3:**
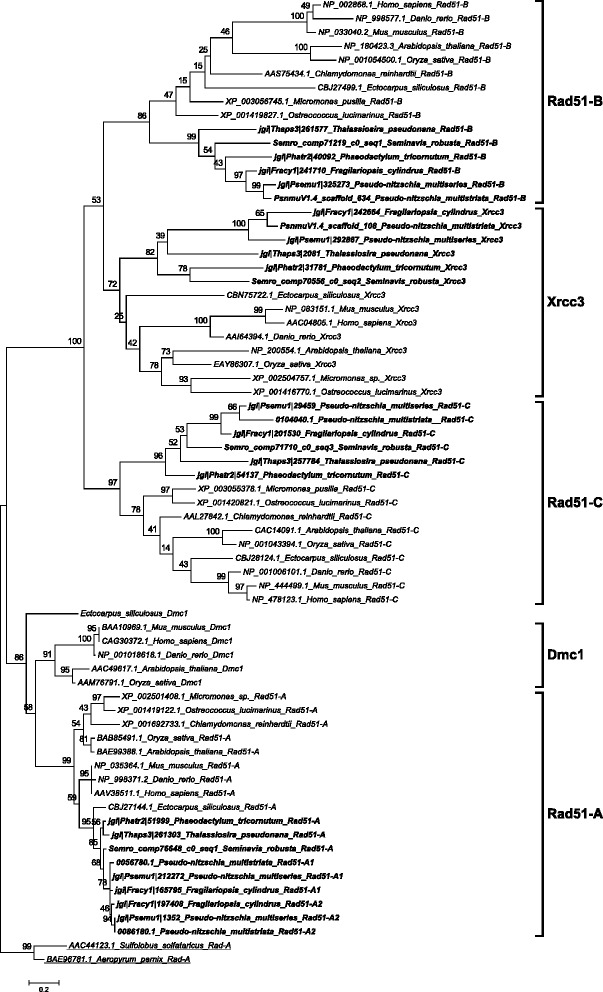
Phylogenetic tree of Rad51 proteins inferred from maximum likelihood analysis. Archaeal Rad-A protein sequences (underlined) were used as an out-group. Numbers on the branches indicate bootstrap support from 1000 replicates. Among-site substitution rate heterogeneity was corrected using two gamma-distributed substitution rate categories and LG substitution model for amino acid substitutions. Diatom sequences are indicated in bold

*RAD51* is an important gene family whose members are employed in homologous recombination during both mitotic and meiotic DNA repair whereas *DMC1* functions exclusively during meiosis [53, 54]. None of the diatom Rad51 homologs clustered with Dmc1 representatives from other organisms (Fig. 3). However, in some sexually reproducing organisms, such as *Drosophila melanogaster* and *Caenorhabditis elegans, DMC1* is missing and other *RAD51* homologs exert its role [55, 56]. This could thus also be the case in diatoms.

When searching for homologs of the other members of the *RAD51* family in diatom genomes, orthologs could be found for all the sequences except for *RAD51-D* and *XRCC2* (Table 2). The Rec-A/Rad51 domain consists of 230 amino acids and contains two conserved consensus motifs, Walker A and Walker B [57], that are found in ATPases and endow hydrolysis and ATPase activity [58]. This domain was present and complete in the diatom Rad51 proteins. For all the diatom Xrcc3 homologs, excluding the *Phaeodactylum tricornutum* Xrcc3 protein 31781, the Rad51 domain was predicted with low e-values. A phylogenetic analysis was performed including all diatom Rad51 homologs (Fig. 3 and Additional file 4). From this analysis, we found that the Xrcc3 proteins clustered correctly with the Xrcc3 sequences from other organisms. In addition, we found two copies of *RAD51-A* in the two *Pseudo-nitzschia* species and in *F. cylindrus*, which were named *RAD51-A1* and *RAD51-A2*.

Of the members of the Rec-A/Rad51 family, Rec-A has been reported to be functioning in the chloroplast [59, 60]. An analysis using SignalP 3.0 [61] and ASAFind [62] for the *P. multistriata*, *T. pseudonana*, and *P. tricornutum* protein sequences revealed the presence of a signal peptide for chloroplastic transport (data not shown), supporting the hypothesis of a role for this homolog in the chloroplast rather than in the nucleus.

### Gene expression analyses in *S. robusta*

In the diatom *S. robusta*, which has a described and controllable sexual phase, the two mating types (MT+ and MT-) can form mating pairs and reproduce sexually once they are below the sexual size threshold, which lies around 50 μm [63]. RNA extracts from synchronized co-cultures of two *S. robusta* strains of opposite mating type were collected at multiple time points during meiosis (when pairing cells can be observed) and after meiosis (auxosporulation, when the production of an elongated specialized zygote, the auxospore, occurs). The same two strains were also grown as monoclonal cultures and were collected at the same time points (vegetatively growing controls). Gene expression changes between the sexually reproducing and the vegetatively growing cultures were assessed using RNA-seq.

**Fig. 4:**
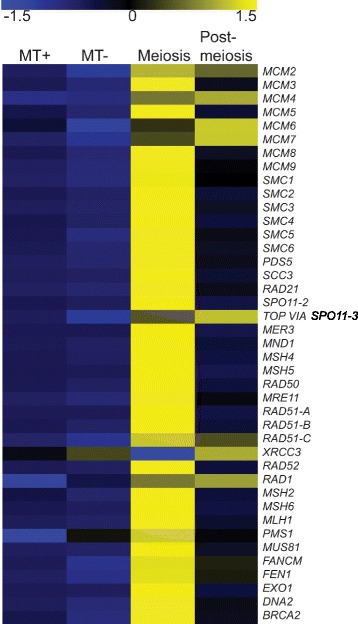
Expression profiles of the meiotic toolkit transcripts found in the transcriptome of *Seminavis robusta.* Expression values (normalized cpm) were determined for dark-synchronized monoclonal cultures (MT+ and MT-) (7 to 10 h after illumination) and for dark-synchronized mixed MT+ and MT- cultures sampled at the time for meiosis (9-10 h after illumination) and post-meiosis (19–22 h after illumination). Blue colour indicates down-regulation and yellow colour upregulation of expression

Cpm values were extracted for the transcripts belonging to the meiotic toolkit (excluding *REC-A* which is supposed to be chloroplastic) and normalized, after which a heatmap was constructed (Fig. 4). For 37 of the transcripts considered in the present analysis, including *RAD21*, *SPO11-2*, *RAD51-A*, *RAD51-B* and *RAD51-C*, expression levels were higher during meiosis when compared to the expression levels at the same time after illumination in vegetatively growing monoclonal cultures. In samples obtained from post-meiotic sexual stages, the expression of meiotic genes decreases, as expected (post-meiosis phase in Fig. 4). Interestingly, the putative mitosis-specific homolog *SPO11-3/Top VIA * appeared to be more abundant during auxosporulation than in other conditions. The *XRCC3* homolog was also more expressed during auxosporulation than during meiosis, similarly to *MCM6* and *MCM7*. For *MCM2*, *MCM4* and *RAD1*, expression was increased during mating compared to the vegetatively growing samples and remained high during auxosporulation.

### Gene expression analyses in *P. multistriata*

**Fig. 5:**
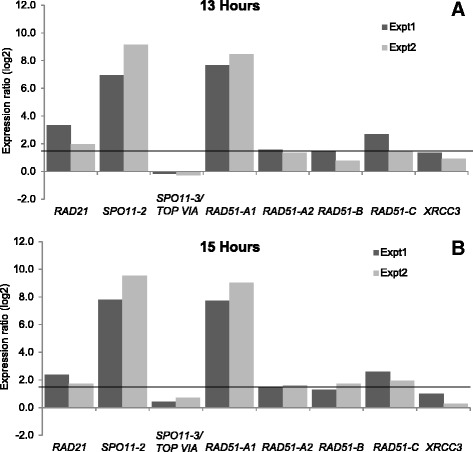
Differential expression analysis of *RAD21*, *SPO11* and *RAD51* homologs during meiosis in *Pseudo-nitzschia multistriata*. Two time points, 13 h (**a**) and 15 h (**b**) after strains of opposite mating type were mixed together, were selected for expression analyses. Dark gray bars represent experiment 1 (Expt1, B937 (MT+) with B936 (MT-)) and light gray bars represent experiment 2 (Expt2, B938 (MT+) with B939 (MT-)). Relative fold changes, with respect to vegetatively growing cultures, in log2 scale, are reported on the Y-axis. A gene was considered differentially expressed if its relative expression change is 1.5 fold or greater (horizontal black line)

We investigated the gene expression profile of selected meiotic genes at two time points during sexual reproduction in *P. multistriata* using real-time quantitative PCR (qPCR)*.* Monoclonal cultures of opposite mating type were used as controls. Specifically, *RAD21*, *SPO11* and *RAD51* paralogs were selected for expression analyses to assess whether there was an indication of a specific requirement for the only *RAD21* homolog and for any of the *SPO11* and *RAD51* paralogs during meiosis in this species as well. *REC8* (the meiotic homolog of *RAD21*), *SPO11* and *RAD51* are known to be expressed early in meiotic prophase I [39, 53]. At both time points, the *RAD21* transcript was significantly upregulated in the co-cultures with respect to the monoclonal cultures, clearly indicating that this gene functions during meiosis (Fig. 5). Similarly, *SPO11-2* showed significant upregulation in its transcript expression in the cultures undergoing sexual reproduction as compared to monoclonal cultures, whereas *SPO11-3/TOP VIA * did not show any significant change between sexually reproducing cultures and monoclonal cultures (Fig. 5). Although all of the *RAD51* homologs examined (*RAD51-A1*, *RAD51-A2, RAD51-B*, *RAD51-C* and *XRCC3*) showed higher expression during sexual reproduction at both time points investigated (Fig. 5), significant upregulation was observed only for *RAD51-A1* and *RAD51-C*.

## Discussion

The availability of genomic data from six diatom species with different life cycles has been exploited to define the set of meiotic and meiosis-related genes for this important group of stramenopile microalgae (Additional file 5: Table S1) and guided experiments to define their expression pattern during sexual reproduction in two pennate species. Importantly, a functional differentiation between the two diatom *SPO11/TOP VIA * homologs can be hypothesized. *RAD21* and homologs of *RAD51* were also found to be more expressed in cells that were undergoing sexual reproduction in both diatoms, indicating their specific requirement during meiosis.

### Genes exclusive to meiosis

Compared to other studies, our list of meiosis-specific genes (Tables 1 and 2) contained three more *ZIP* (Zipping up meiotic chromosomes protein) genes, *ZIP2*, *ZIP3* and *ZIP4* [64], all reported to be required for the synaptonemal complex in budding yeast [47, 64, 65]. Moreover, we list as meiosis-specific both *SPO11-1* and *SPO11-2*, therefore the total number of meiosis-specific genes increases from 11 [9] to 15 (Table 1). Of these 15 genes, five were detected in all the diatom genomes surveyed (Table 2)*.* In the *Seminavis robusta* dataset, these five genes were all clearly upregulated during mating (Fig. 4), as was the one tested in *Pseudo-nitzschia multistriata* (*SPO11-2*, Fig. 5). The role of sex in the ecology and biogeochemical function of natural diatom populations has been challenging to investigate with classical methods as diatom sexual stages are difficult to recognize and preserve poorly [15]. The genes identified in the present study might prove useful as molecular markers to detect events of diatom sexual reproduction in nature.

Ten meiosis-specific genes could not be identified in any of the diatom genomes. The absence of some of these genes has also been reported for other species (Additional file 5: Table S1) and has been correlated with the presence of alternative structures and pathways required during meiotic recombination [66–69].

While duplications of some of the meiotic toolkit genes have been observed in protists and other organisms (Additional file 5: Table S1), the diatom genes were all present in single copies, except for the notable expansion observed in the *RAD51* family.

### Does Rad21 function as a component of the cohesin complex both during mitosis and meiosis in diatoms and other Stramenopiles?

Although the *SMC1, SMC2, SMC3, SMC4, SMC5* and *SMC6* genes, whose products are functional components of the cohesin and condensin complexes (required for chromatin organization during cell division), were identified in diatoms, *REC8*, an important component of cohesin complex and meiosis-specific homolog of *RAD21* [50, 70], seemed to be absent in the diatoms investigated (Additional file 5: Table S1). Gene expression analysis of the *RAD21* homolog during sexual reproduction in *P. multistriata* and *S. robusta* supports the hypothesis that *REC8* might be substituted by *RAD21,* which would function as a component of the cohesin complex both during mitosis and meiosis (Figs. 4 and 5). Indeed, during mammalian meiosis, the mitotic *RAD21* cohesin has been shown to perform the role of *REC8* [30, 71]. On the contrary, in the ciliate *Tetrahymena thermophila* that is lacking *RAD21*, it has been shown that *REC8*, the meiotic homolog of *RAD21,* replaces the function of *RAD21* during mitosis [72]. The loss of *REC8* appears to be a character shared by all Stramenopiles (Additional file 5: Table S1).

### *SPO11-2* is the meiosis-specific *SPO11* paralog

*SPO11*, encoding a conserved protein involved in DNA DSBs formation and thus in initiation of homologous recombination, was found to have two paralogs in diatoms, *SPO11-2* and *SPO11-3/TOP VIA* (Fig. 2). In many plants, including *Arabidopsis thaliana*, three paralogs of *SPO11* have been reported, of which *SPO11-1* and *SPO11-2* are meiosis-specific, while *SPO11-3/TOP VIA * has a topoisomerase function as it interacts with topoisomerase VIB (Top VIB) and is required during vegetative growth [27]. However, most animals, insects and yeasts possess the meiosis-specific *SPO11-1* homolog [73] (Additional file 5: Table S1) and lack the other counterpart of topoisomerase assembly, the *TOP VIB* homolog [73]. In diatoms, red algae and prasinophytes, the *SPO11-1* gene seems to be lost, although the *SPO11-2* and *SPO11-3/TOP VIA * homologs are conserved [74, 75]. Although functional differentiation between *SPO11-1* and *SPO11-2* is not fully resolved in plants, in *A. thaliana SPO11-2* mutants the male and female meiosis is severely disrupted, while the mutation does not affect vegetative growth [27]. This suggests that *SPO11-2* is involved in meiotic recombination. Similar to many plants, it had been hypothesized that the *SPO11-2* homolog in diatoms is involved in meiosis, while the product of *SPO11-3/TOP VIA * homolog may interact with the Top VIB subunit and might be involved in vegetative growth [27, 73, 74, 76]. The present study demonstrated that *SPO11-2* mRNA levels were significantly upregulated during sexual reproduction in *P. multistriata* and *S. robusta*, providing the first experimental evidence for the functional distinction between *SPO11* paralogs in diatoms. Gene expression studies in the centric diatom *Thalassiosira weissflogii* also revealed the meiosis-specific role of *SPO11-2* (Additional file 6). *SPO11-1* is absent in all members of the Stramenopiles analyzed to date, and might have been lost early in the divergence of the alveolate and stramenopile lineages.

### Homologous recombination and strand exchange in the absence of *DMC1*

Archaeal Rad-A homologs Rad51 and Dmc1 work collectively in homology search and strand exchange processes during meiotic recombination, although it is unclear how they cooperate [54, 77]. However, in mitotic cells only Rad51 is induced and carries out recombination [77]. Tsubouchi et al. [78] have proposed two different pathways of homology searching during meiosis. In the first pathway, Dmc1 and Rad51 act together with Hop2 and other accessory proteins to accomplish efficient homology searching. In budding yeast, mutation in the *HOP2* gene results in inappropriate homology searching, leading to extensive synaptonemal complex formation between non-homologous chromatids [41, 79]. In this Dmc1-dependent pathway, Hop2 interacts with Mnd1 downstream of Dmc1 and Rad51 homology searching, with Hop2 being a major DNA binding protein and Mnd1 the foremost protein interacting with Rad51 [42, 80]. In the second pathway, only Rad51 is involved in homology searching. The same study [78] also reported that overexpression of *RAD51* suppresses defects in *DMC1* mutants, indicating Rad51 can carry out effective homology searching independently. Crismani et al. [81] recently reported that in *Arabidopsis* Rad51 can work together with Mcm8 to repair meiotic double strand breaks when the Dmc1-dependent major repair pathway fails. The Mcm family of DNA helicases consists of nine homologs of which six (Mcm2-7) are conserved across the eukaryotic kingdom and function as heterohexamer helicase in DNA replication [28], whereas the other three (Mcm8-10) are less conserved, with Mcm8-Mcm9 being proved to work during meiotic recombination [82, 83]. Mcm8 and Mcm9 form a stable complex and promote recruitment of Rad51 to the DNA damage sites [83, 84]. Although diatoms lack *DMC1* and *HOP2* genes, they possess five to six homologs of *RAD51* (either one or two homologs of *RAD51-A,* and one homolog each of *RAD51-B, RAD51-C, XRCC3* and *REC-A*) and *MCM8* and *MCM9*. In *A. thaliana*, *RAD51-C* and *XRCC3* have been shown to be involved in meiotic recombination [85, 86]. In *P. multistriata*, we observed that *RAD51-A1* and *RAD51-C* were upregulated during sexual reproduction, and in *S. robusta RAD51-A, RAD51-B* and *RAD51-C* appeared to be upregulated during sexual reproduction*.* Based on the RNA-seq data produced for *S. robusta*, *MCM8* and *MCM9* also appeared upregulated during meiosis (Fig. 4). Intriguingly, a homolog of the *HOP2* gene, whose protein product forms a heterodimer with Mnd1, could not be found, while *MND1* was found in diatoms. Either Hop2 is highly diverged in diatoms and is currently beyond the detection by homology searches or another mechanism involving Mnd1 exists, as the *S. robusta* homolog is indeed up-regulated during meiosis. Therefore, diatoms may have evolved an alternative meiotic double strand break repair pathway that does not involve Dmc1. Absence of *DMC1*, *HOP2* and *MND1* homologs has been reported for certain sexually reproducing organisms such as *Caenorhabditis elegans, Drosophila melanogaster* and *Neurospora crassa* (Additional file 5: Table S1) and absence of *HOP2* has been reported in *Gallus gallus*, *Phytophthora* species, and some fungi [6]*.* As *DMC1* and *HOP2* orthologs were identified in the *Ectocarpus siliculosus* genome but not in sexual diatoms, the losses of a *DMC1* dependent DSB repair pathway and of *HOP2* might be specific to certain lineages of Stramenopiles and do not correlate with obligate asexuality.

### Does cross-over occur without canonical synaptonemal complex (SC) in diatoms?

The synaptonemal complex (SC), a proteinaceous structure, is developed during early prophase I of meiosis and is thought to juxtapose homologous chromatids to enhance crossing over during meiotic recombination [45]. Almost all animals, plants and fungi capable of meiosis possess the SC [87]. The ZMM (Zip, Msh, Mer) group of proteins includes seven functionally similar yet structurally diverse proteins that coordinate recombination events and SC formation during meiosis [45]. Functionally, ZMM proteins can be categorized into three subgroups. Subgroup I includes Mer3 and Msh4-Msh5, subgroup II includes Zip2, Zip3 and Zip4 while subgroup III includes the Zip1 protein. The Zip1 protein produces a stable connection between two homologous chromosomes [47, 88], Zip2, Zip3 and Zip4 facilitate protein-protein interactions [64, 65, 89] and Mer3, Msh4 and Msh5 promote DNA recombination [45, 90]. However, homologs of Zip1 among fungi (Zip1, [47]), animals (Sycp1, [91]) and plants (Zyp1, [88, 92]) are highly divergent. Similarly, the other Zip proteins have functional analogues in fungi (Zip2, Zip3 and Zip4 [64, 65]), animals (Zhp-3/Hei10 [93], Zip4H/Tex11 [94]) and plants (Zip3/Hei10 [95], Zip4 [96]) albeit with very low or no sequence similarity among different taxa. The diatom genomes contained neither identifiable homologs of *ZIP* genes (Table 2), nor of *HOP1* and *RED1*, the components of lateral elements in SCs (Table 2 and Additional file 5: Table S1) and thus, diatoms may lack canonical SCs. Such a possibility was proposed for ciliates. In ciliate genomes, none of the genes related to canonical SCs were detected [9] and the corresponding absence of a canonical SC in the ciliate *Tetrahymena thermophila* was also supported by microscopic observations [67, 97]. The presence of a rigid siliceous frustule and dense ring of chromatin around the central spindle at metaphase make it difficult to perform routine cytological studies in diatoms [16, 98], however, in some species SC-like structures have been reported [99, 100], so SC-like structures might involve unidentified proteins that have replaced the Zip and Hop1 protein functions. Alternatively, the homologous proteins are present in diatoms but have diverged so much that they are unrecognizable.

### DNA Mismatch repair genes (*MSH* and *MLH* gene family)

The *MSH* gene family comprises homologs of bacterial *MutS* genes that are important for DNA mismatch recognition and repair (MMR). *MSH* genes have been reported in all eukaryotes and are fundamentally involved in the initial recognition of nucleotide mismatch during repair [101, 102]. Although seven *MSH* homologs (*MSH1-7*) have been identified among eukaryotes, *MSH1* and *MSH7* are less conserved [102]. Msh proteins form heterodimers, Msh2-Msh6 is principally involved in MMR during mitosis whereas Msh4-Msh5 functions during meiosis [32, 44], stabilizing single strand invasion intermediates formed during early stages of meiotic recombination [90, 103]. Further, it directs Holliday Junction resolution towards crossover formation following an interference sensitive pathway [103, 104]. T-DNA insertional mutation of *MSH4* of *Arabidopsis* exhibits reduced fertility with no effects on normal vegetative growth [103]. *MSH2, MSH4, MSH5* and *MSH6* were identified in all diatom genomes investigated and they were upregulated during meiosis in *S. robusta* indicating the presence of a complete and active MMR machinery.

Prokaryotic MutL homologs (Mlh) of DNA MMR proteins are another important group of conserved meiotic genes that work in coordination with Msh homologs. Multiple copies of *MLH* homologs (*MLH1-3* and *PMS1-2*) are present in eukaryotes [105]. Mlh1 and Pms1/2 form heterodimers and interact with Msh2-Msh4 or Msh4-Msh5 heterodimers to remove DNA mismatches during replication [101, 105, 106]. Msh heterodimers initiate DNA MMR by recognizing and binding to unpaired and impaired bases. In addition, they activate the Mlh complex endonuclease that further incises DNA mismatches [107]. Mlh1-Pms1 is the major heterodimer and in some eukaryotes the Mlh family contains multiple homologs (all of which form heterodimers with Mlh1) [107]. Diatoms contain *MLH1* and *PMS1* whereas *MLH2* and *MLH3* were not detected, suggesting that the Mlh1-Pms1 complex plays a major role in MMR in diatoms. The latter hypothesis is supported by the upregulation of both genes during mating in *S. robusta*. Although *MLH2* was not detected in other SAR supergroup members examined, as is the case for diatoms, an *MLH3* homolog was detected in *E. siliculosus*, suggesting that MMR varies among Stramenopiles.

### The meiotic toolkit in *P. tricornutum* and *T. pseudonana*

Centric and pennate diatoms differ in many aspects of their life cycles, and meiosis also differs in many ways, with a different number of gametes produced in pennates (generally two isogamous gametes) with respect to centrics (one large sized female gamete and many small sized male gametes) [108, 109]. Nevertheless, our results suggest that the molecular machinery employed in meiotic recombination may be shared by all diatom species studied. This includes *P. tricornutum* and *T. pseudonana* for which a sexual phase has never been reported. Differences could only be found for the *RAD51* family, where *P. multistriata, P. multiseries* and *F. cylindrus* appeared to have a duplicated *RAD51-A* gene, a canonical version of *XRCC3* appeared to be present only in *P. tricornutum,* while *T. pseudonana* lacked *MUS81*.

This would suggest that the apparent lack of a sexual phase for *P. tricornutum* and *T. pseudonana* is unlikely due to major losses in the meiotic toolkit genes. In spite of the rapid evolution of the genomes of these two diatoms and the presence of a significant amount of transposable elements in their genomes, the meiotic genes are conserved. Since meiotic genes have been reported in the genome of asexual organisms [33, 110], it cannot be excluded that these diatom species are truly asexual, with meiosis-related genes having undergone neo-functionalization and becoming employed in non-meiotic processes such as DNA repair. Moreover, detailed analyses should include an assessment of the integrity of the meiosis-related genes identified, to rule out a recent accumulation of mutations rendering the genes non-functional (i.e., species-specific or even strain-specific loss of sex after isolation in culture). For example, the meiotic *SPO11-2* homolog in the *T. pseudonana* genome appeared to be missing the N-terminal portion of the gene found in other diatoms (Additional file 4). The presence of meiosis related genes in the genome of *T. pseudonana*, even if some genes may be subject to recent loss-of-function mutations (in the CCMP1335 genome), suggests that some members of the species may have retained this capacity, as seen recently in the coccolithophore *Emiliania huxleyi* [36]. However, at least for *P. tricornutum*, which is a pennate diatom (pennate diatoms are generally heterothallic)*,* few strains have been used in laboratories across the world and it could be that the right mating partner has never been used in crossing experiments. This species is both important for fundamental research and promising in biotechnology, and the ability to conduct laboratory breeding would greatly enhance this potential. The isolation of additional wild type *P. tricornutum* strains should be pursued in order to verify if sexual reproduction can be induced in the laboratory.

## Conclusions

Analysis of the meiotic toolkit in diatoms revealed that the majority of meiosis-related genes are present and, in two species tested, showed an expression consistent with their proposed role. However, it seems that not all eukaryotic meiosis-specific genes are required to complete meiosis in diatoms. Specifically, our results suggest the presence of a Dmc1-independent pathway for double strand break repair during meiosis in diatoms. The absence of the genes required for canonical SC formation in diatoms may explain why the SC has not been seen during meiotic divisions. The assignment of specific functional roles to the meiosis-related genes in diatoms, for comparison to roles of homologous proteins in yeasts, plants, and animals, will need further investigation using various approaches, including reverse genetics and protein interaction analyses. More broadly, the presented data refine our knowledge of patterns of evolutionary divergence of meiosis, a fundamental process ancestral to all extant eukaryotes. The SAR supergroup has undergone fundamental modifications to the meiosis process compared to other representatives of both the Diaphoretickes/bikont megaclade (Archaeplastida) and the Amorphea/unikont megaclade (Opisthokonts: animals and fungi). Features common among the SAR members are the absence of Mlh2 and Xrs2/Nbs1 in DNA damage sensing and the loss of components involved in canonical SC formation. Within the Stramenopiles there is also a general trend to lose canonical components in meiotic recombination, some of which have occurred in specific branches, and in some cases the meiosis-specific components may have been replaced by distant homologs with known mitotic functions.

## Methods

### Culture conditions and strains used

*Seminavis robusta* strains were grown at 18 °C in a 12 L:12D h (light:dark) regime with cool white fluorescent lamps at approximately 80 μmol photons m^−2^ s^−1^. *S. robusta* strains 85A and 85B used in RNA-seq experiments are publicly available in the diatom culture collection of the Belgian Coordinated Collection of Micro-organisms (BCCM/DCG, http://bccm.belspo.be, accession numbers DCG 0105 and DCG 0107). *Pseudo-nitzschia multistriata* strains B936 (MT-), B937 (MT+), B938 (MT+) and B939 (MT-) were grown at 18 °C, under 100 μmol photons m^−2^ s^−1^ irradiance with 12 L:12D h (light:dark) photoperiod. Cultures were grown in Guillard F/2 medium [111] made with autoclaved filtered natural sea water collected from the North Sea (for *S. robusta*) or the Gulf of Naples (for *P. multistriata*) and Guillard’s F/2 solution (Sigma-Aldrich).

### Database search for conserved meiotic genes in diatoms

A list of conserved meiotic genes was taken from [6] and expanded with additional genes reported to be involved in meiosis. Meiotic protein sequences of *Arabidopsis thaliana* or *Saccharomyces cerevisiae* (Table 2) were used as query sequences for the homology searches. Keyword based searches in the NCBI protein database were made to retrieve the protein sequences. *S. cerevisiae* proteins were selected when no *A. thaliana* protein could be found for a given meiotic gene (Table 2). Meiotic protein homologs for four diatom species with publicly available genomes, including *Thalassiosira pseudonana* v3.0*, Phaeodactylum tricornutum* v2.0*, Fragilariopsis cylindrus* v1.0 and *Pseudo-nitzschia multiseries* v1.0*,* were retrieved by BLASTp searches from the Joint Genome Institute (JGI) database (http://genome.jgi-psf.org/). In the case of *Thalassiosira pseudonana* and *Phaeodactylum tricornutum* genomes, “unmapped sequences” databases (http://genome.jgi.doe.gov/Thaps3_bd/Thaps3_bd.home.html and http://genome.jgi.doe.gov/Phatr2_bd/Phatr2_bd.home.html, respectively) were also searched since a significant portion of their genome sequence is maintained in these additional databases. The diatom homologs were first searched in filtered models and search was further extended to all models only if the respective homolog was not detected in filtered models. Meiotic gene homologs of *P. multistriata* were retrieved by tBLASTn searches in the v1.4 genome assembly (Ferrante, in preparation). Sequences for the retrieved *P. multistriata* gene models are given in Additional file 7 and corresponding proteins are given in Additional file 8. The search for the presence of meiotic genes was extended to the *de novo* transcriptome of *S. robusta* using tBLASTn searches. Sequences for the *S. robusta* transcripts are given in Additional file 9. The protein sequences of the resulting transcripts were predicted using Trapid [112] and then manually curated by mapping the transcripts to an in-house draft genome of *S. robusta* (Vandepoele, De Veylder & Vyverman, in preparation). The resulting protein sequences were blasted (BLASTp) against the Uniprot-Swissprot database to confirm their functional annotation. The resulting protein sequences are given in Additional file 10.

We took into consideration only those sequences showing a BLAST e-value smaller or equal to 1e10^−4^. The resulting dataset was further manually curated verifying the presence of at least one functional domain using the phmmer search against the UniProtKB sequences with an e-value cutoff of 1e10^−4^ on the webserver HMMER (http://hmmer.janelia.org/) [113]. For dubious cases, Interpro scan (http://www.ebi.ac.uk/interpro/) and CD-search (http://www.ncbi.nlm.nih.gov/Structure/cdd/wrpsb.cgi?) were performed.

A reciprocal blast was performed to confirm that each retrieved diatom sequence had the corresponding query sequence as top hit when searching against the *A. thaliana* (or *S. cerevisiae*) protein database.

An alternative approach to verify absence of genes involved searches using HMMER. Protein sequences (covering major taxa from different eukaryotic groups) from the NCBI protein database were downloaded and aligned using the MUSCLE program [114]. Further, HMM profiles were generated (Additional file 11) using default settings of the HMMBUILD command in the HMMER 3.1b software and these HMM profiles for respective gene families were then used to search against the diatom protein databases (already mentioned above). No additional proteins from any of the diatom genomes could be identified when using this approach. In certain cases Hmmsearch did yield entries but manual inspection of the sequences showed that the resulted protein belonged to other gene families (data not shown).

There is not a defined convention for diatom gene nomenclature, we chose to indicate diatom gene names by capital letters and italics, and proteins in lowercase with a capital first letter, following the convention used for *S. cerevisiae*.

### Phylogenetic analyses

For the phylogenetic analysis of each gene, the corresponding protein sequences from representative taxa of plants, animals, fungi and protists were retrieved from NCBI (http://www.ncbi.nlm.nih.gov/protein/) and JGI with keyword searches and aligned using the sequence alignment software MUSCLE [114]. Maximum likelihood analysis was performed using MEGA 6 (Molecular Evolutionary Genetics Analysis) [115] with appropriate substitution model suggested by the software, specified in the figure legends.

### RNA-seq for *S. robusta*

For the mitotic libraries, *S. robusta* strains 85A (MT+) and 85B (MT-) with an average cell size below the sexual size threshold (SST) were grown under abovementioned growth conditions and before sampling, the dark period was extended with 12 h to synchronize cells at the G1 phase [116]. After illumination, synchronization was assessed by light microscopy. Pictures were taken using a digital camera connected to a Zeiss Axiovert 40 light microscope and the percentage of dividing cells (distinguished from interphase cells by the newly built cell wall between the two valve-appressed chloroplasts) was counted using cell counter plug-in of the ImageJ software. Cultures were harvested hourly from seven until ten hours post-illumination and cell pellets were frozen in liquid nitrogen and stored at –80 °C until RNA extraction.

For the sexual stages, monoclonal cultures were grown as described above. Three hours before illumination, 85A cell suspensions were added to 85B cultures in dark conditions. Harvesting was done analogous to the vegetative samples at nine and ten hours post-illumination, during which cell-pairing (and thus meiosis) was observed, and at 19, 20 and 21 h, when auxosporulation occurs and thus the meiotic phase is passed.

Total RNA was extracted from each sample using the RNeasy Plant Mini Kit (Qiagen). Cell lysis was achieved by mechanical disruption in 1 mL of RNeasy Lysis buffer (Qiagen) by highest speed agitation with glass/zirconium beads (0.1 mm diameter; Biospec) on a bead mill (Retsch). All other steps for RNA extraction were done according to the manufacturer’s instructions. RNA samples were pooled in equal amounts before sequencing.

Poly-(A) RNA was isolated from 5 μg total RNA using Dynabeads mRNA isolation kit (Invitrogen). Purified RNA was then fragmented using RNA Fragmentation Reagents (Ambion) at 70 °C for 3 mins, targeting fragments range 200–300 bp. Fragmented RNA was purified using Ampure XP beads (Agencourt). Reverse transcription was performed using SuperScript II Reverse Transcription (Invitrogen). Double stranded cDNA fragments were purified and selected for targeted fragments (200–300 bp) using Ampure XP beads. The cDNA was blunt-ended, poly-adenylated, and ligated with library adaptors using Kapa Library Amplification Kit (Kapa Biosystems). Digestion of dUTP was performed using AmpErase UNG (Applied Biosystems) to remove second strand cDNA. Digested cDNA was cleaned up with Ampure XP beads. This was followed by amplification by 10 cycles PCR using Kapa Library Amplification Kit (Kapa Biosystems). The final library was cleaned up with Ampure XP beads. Sequencing was done on the Illumina platform generating paired end reads of 150 bp each.

### *De novo* transcriptome of *S. robusta* and differential expression analysis

The *de novo* transcriptome for *S. robusta* was assembled using RNA-seq data generated in collaboration with the JGI institute (http://www.jgi.doe.gov/) within the project “A deep transcriptomic and genomic investigation of diatom life cycle regulation”. Raw reads are available at http://genome.jgi-psf.org/pages/dynamicOrganismDownload.jsf?organism=SemrobtraphaseII. Libraries used in this study are CYAG (MT+), CYAC (MT-), CYAN (meiosis) and CYAH (post-meiosis).

Raw reads were filtered and trimmed based on quality and adapter inclusion using Trimmomatic [117] with the following parameters: -threads 20 -phred64 ILLUMINACLIP:illumina_adapters.fa:2:40:15 LEADING:5 TRAILING:5 SLIDINGWINDOW:5:20 MINLEN:100. Trimmed and filtered reads were normalized using the normalize_by_kmer_coverage.pl script from the Trinity [118] software (release r2013_08_14) with the following parameters: --seqType fq --JM 240G --max_cov 30 --SS_lib_type RF --JELLY_CPU 24. Assembly was performed using Trinity on the trimmed, filtered and normalized reads with the following parameters: --seqType fq --JM 220G --inchworm_cpu 22 --bflyHeapSpaceInit 22G --bflyHeapSpaceMax 220G --bflyCalculateCPU --CPU 22 --SS_lib_type RF --min_kmer_cov 2 --jaccard_clip. All reads were mapped to the assembled transcriptome using bowtie (version 1) [119] with the following parameters -p 20 -S --chunkmbs 10240 -t --maxins 500 --trim5 20 --trim3 20 --seedlen 20 --tryhard –a. Quantification of the mapping to obtain the number of raw reads mapping on each transcript in each condition was performed using the samtools view, sort, index and idxstats programs with default parameters [120]. Cpm values were calculated for all the genes using R and extracted for the meiosis transcripts, after which a heatmap was constructed using MeV [121].

### Experimental set-up for the gene expression studies in *P. multistriata*

Two experiments were carried out: one (Expt. 1) with *P. multistriata* strains B936 (MT-) and B937 (MT+) and the other (Expt. 2) with strains B938 (MT+) and B939 (MT-). Exponentially growing cultures were synchronized by incubating them in the dark for 36 h. Monoclonal cultures of MT+ and MT- strains were grown as controls and the same MT+ and MT- strains were mixed together to induce the sexual phase. The timing for collection of samples for RNA was chosen based on earlier observations on the timing of gamete formation: under the specified experimental set up, pairing cells could be observed starting from 10 h after the opposite mating type cells were mixed together and gametes could be observed 24 h after the opposite mating type cells were mixed together (Scalco et al. in press). The samples for RNA were therefore collected from controls and mixed cultures at 13 and 15 h after the start of co-culturing. Mixed cultures and vegetative control samples were collected onto 1.2 μm pore-size membrane filters (RAWP04700 Millipore), placed in Trizol™, flash frozen in liquid nitrogen immediately and stored at -80 °C until RNA extractions. A control plate with the mixed culture was maintained and observed after 24 h of co-culturing to verify that gamete formation had occurred.

RNA was extracted according to the manufacturer’s instructions (Trizol reagent, Invitrogen) and the genomic DNA contamination was removed by DNase I treatment (RNase-Free DNase Set, Qiagen) followed by RNA purification using RNeasy Plant Mini Kit (Qiagen). The quantity of RNA was determined using the Qubit assay (Qubit® 2.0 Fluorometer, Life Technologies) and RNA integrity was assessed by running samples on a 1.5 % agarose gel. One microgram of total RNA was further used for cDNA preparation using the QuantiTect® Reverse Transcription Kit (Qiagen).

*RAD21* and homologs of *SPO11* and *RAD51* genes were retrieved from the genome sequence of *P. multistriata* and real time qPCR primers were designed manually (Additional file 12). To ensure specificity of the primer to the specific homolog, the homologs were aligned using ClustalX [122] and primers were designed on divergent fragments of the sequence.

The expression profiles of *RAD21*, *SPO11-2*, *SPO11-3/TopVIA*, *RAD51-A1*, *RAD51-A2*, *RAD51-B*, *RAD51-C* and *XRCC3* genes were analyzed using *CDK-A* and *COPA* as normalization genes [123]. qPCR amplification was performed as previously described [123]. The results were analyzed and collected in an Excel sheet using the ViiA™ 7 Software. Gene expression analysis was performed on two biological replicates. Each biological sample was run in technical triplicates. Expression analysis was performed using the Relative Expression Software Tool-Multiple Condition Solver (REST-MCS), the calculation software for the relative expression in qPCR, using Pair Wise Fixed Reallocation Randomization Test [124].

## Availability of supporting data

The datasets supporting the results of this article are available at http://genome.jgi-psf.org/pages/dynamicOrganismDownload.jsf?organism=SemrobtraphaseII.

## Additional files

Additional file 1: Figures S1-S18. Phylogenetic inferences of meiosis genes searched in diatom genomes. (PDF 862 kb) Additional file 2:
**Multiple sequence alignment of Rec8/Rad21proteins used in building the phylogenetic tree of Rad21, shown in Fig.** 1 **.** (PDF 894 kb) Additional file 3:
**Multiple sequence alignment of Spo11 proteins used in building the phylogenetic tree of Spo11, shown in Fig.** 2 **.** (PDF 553 kb) Additional file 4:
**Multiple sequence alignment of Rad51 proteins used in building the phylogenetic tree of Rad51, shown in Fig.** 3 **.** (PDF 1107 kb) Additional file 5: Table S1. Overview of phylogenetic distribution of core meiotic proteins among eukaryotes [11, 72, 133–142]. (DOC 235 kb) Additional file 6: Figure S19. Expression profiles of *SPO11-2* and *SPO11-3/TOP VIA * homologs in *Thalassiosira weissflogii* during spermatogenesis compared to purely asexually-dividing cultures. (PDF 248 kb) Additional file 7:
**Gene models for meiotic genes from the** ***Pseudo-nitzschia multistriata*** **genome.** (DOCX 142 kb) Additional file 8:
**Protein sequences for meiotic genes from the** ***Pseudo-nitzschia multistriata*** **genome in fasta format.** (FASTA 54 kb) Additional file 9:
**Transcript sequences for meiotic genes in** ***Seminavis robusta*** **.** (FAS 108 kb) Additional file 10:
**Protein sequences for meiotic genes for** ***Seminavis robusta*** **in fasta format.** (FAS 37 kb) Additional file 11:
**HMM profiles used for Hmmsearch.** (DOC 90 kb) Additional file 12:
**List of the genes and corresponding primers tested in** ***Pseudo-nitzschia multistriata*** **with qPCR.** (XLSX 13 kb)

## Abbreviations

DMC1: disrupted meiotic C-DNA1
DSB: double strand break
MCM: mini-chromosome maintenance complex
MMR: DNA mis-match repair
MT: mating type
qPCR: quantitative PCR
SPO11: sporulation-specific protein 11
SC: synaptonemal complex
SMC: structural maintenance of chromosomes
SST: sexual size threshold
TOP VI: topoisomerase VI
ZIP: zipping up meiotic chromosomes protein
