# Correction to: Identification of the meiotic toolkit in diatoms and exploration of meiosis-specific *SPO11* and *RAD51* homologs in the sexual species *Pseudo-nitzschia multistriata* and *Seminavis robusta*

**DOI:** 10.1186/s12864-019-5942-4

**Published:** 2019-07-05

**Authors:** Shrikant Patil, Sara Moeys, Peter von Dassow, Marie J. J. Huysman, Daniel Mapleson, Lieven De Veylder, Remo Sanges, Wim Vyverman, Marina Montresor, Maria Immacolata Ferrante

**Affiliations:** 1 Stazione Zoologica Anton Dohrn, Villa Comunale 1, 80121 Naples, Italy; 2 Department of Biology, Protistology and Aquatic Ecology, Ghent University, 9000 Ghent, Belgium; 3 Department of Plant Systems Biology, Flanders Institute for Biotechnology (VIB), 9052 Ghent, Belgium; 4 Department of Plant Biotechnology and Bioinformatics, Ghent University, 9052 Ghent, Belgium; 5 Facultad de Ciencias Biológicas, Instituto Milenio de Oceanografía, Pontificia Universidad Católica de Chile, Santiago, Chile; 6 UMI 3614, Evolutionary Biology and Ecology of Algae, CNRS-UPMC Sorbonne Universités, PUCCh, UACH, Station Biologique de Roscoff, Roscoff, France; 7 The Genome Analysis Centre (TGAC), Norwich Research Park, Norwich, NR4 7UH UK

**Correction to: BMC Genomics**

**https://doi.org/10.1186/s12864-015-1983-5**

Following the publication of this article [1], the authors reported that the link to Additional file 11 linked to the wrong set of data. The correct supplementary data is provided in this Correction article (Additional file 11).

## Additional file

Additional file 11: HMM profiles used for Hmmsearch. (ZIP 1469 kb)
