# Supplementary material for: Identification of the meiotic toolkit in diatoms and exploration of meiosis-specific SPO11 and RAD51 homologs in the sexual species Pseudo-nitzschia multistriata and Seminavis robusta
Source: BMC Genomics. 2015 Nov 14;16:930. doi: 10.1186/s12864-015-1983-5 (PMC4647503; doi:10.1186/s12864-015-1983-5)
Supplement: Additional file 1: Figures S1-S18. — Phylogenetic inferences of meiosis genes searched in diatom genomes. (PDF 862 kb) [file 12864_2015_1983_MOESM1_ESM.pdf]

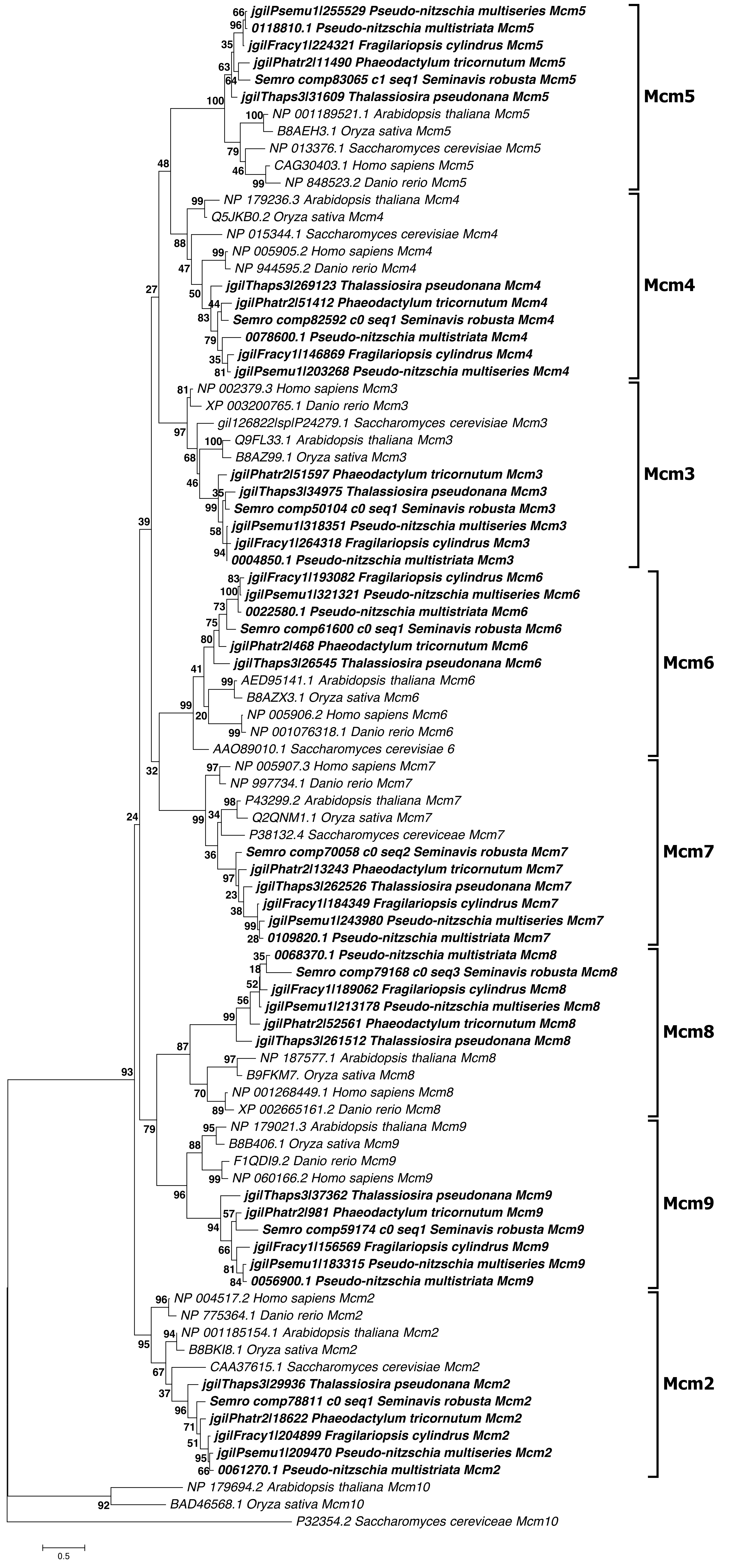

jgi|Psemu1|255529 Pseudo-nitzschia multiseries Mcm5
0118810.1 Pseudo-nitzschia multistriata Mcm5
jgi|Fracy1|224321 Fragilariopsis cylindrus Mcm5
jgi|Phatr2|11490 Phaeodactylum tricornutum Mcm5
Semro comp83065 c1 seq1 Seminavis robusta Mcm5
jgi|Thaps3|31609 Thalassiosira pseudonana Mcm5
NP 001189521.1 Arabidopsis thaliana Mcm5
B8AEH3.1 Oryza sativa Mcm5
NP 013376.1 Saccharomyces cerevisiae Mcm5
CAG30403.1 Homo sapiens Mcm5
NP 848523.2 Danio rerio Mcm5
Mcm5
NP 179236.3 Arabidopsis thaliana Mcm4
Q5JKB0.2 Oryza sativa Mcm4
NP 015344.1 Saccharomyces cerevisiae Mcm4
NP 005905.2 Homo sapiens Mcm4
NP 944595.2 Danio rerio Mcm4
jgi|Thaps3|269123 Thalassiosira pseudonana Mcm4
jgi|Phatr2|51412 Phaeodactylum tricornutum Mcm4
Semro comp82592 c0 seq1 Seminavis robusta Mcm4
0078600.1 Pseudo-nitzschia multistriata Mcm4
jgi|Fracy1|146869 Fragilariopsis cylindrus Mcm4
jgi|Psemu1|203268 Pseudo-nitzschia multiseries Mcm4
Mcm4
NP 002379.3 Homo sapiens Mcm3
XP 003200765.1 Danio rerio Mcm3
gi|126822|sp|P24279.1 Saccharomyces cerevisiae Mcm3
Q9FL33.1 Arabidopsis thaliana Mcm3
B8AZ99.1 Oryza sativa Mcm3
jgi|Phatr2|51597 Phaeodactylum tricornutum Mcm3
jgi|Thaps3|34975 Thalassiosira pseudonana Mcm3
Semro comp50104 c0 seq1 Seminavis robusta Mcm3
jgi|Psemu1|318351 Pseudo-nitzschia multiseries Mcm3
jgi|Fracy1|264318 Fragilariopsis cylindrus Mcm3
0004850.1 Pseudo-nitzschia multistriata Mcm3
Mcm3
jgi|Fracy1|193082 Fragilariopsis cylindrus Mcm6
jgi|Psemu1|321321 Pseudo-nitzschia multiseries Mcm6
0022580.1 Pseudo-nitzschia multistriata Mcm6
Semro comp61600 c0 seq1 Seminavis robusta Mcm6
jgi|Phatr2|468 Phaeodactylum tricornutum Mcm6
jgi|Thaps3|26545 Thalassiosira pseudonana Mcm6
AED95141.1 Arabidopsis thaliana Mcm6
B8AZX3.1 Oryza sativa Mcm6
NP 005906.2 Homo sapiens Mcm6
NP 001076318.1 Danio rerio Mcm6
AAO89010.1 Saccharomyces cerevisiae 6
Mcm6
NP 005907.3 Homo sapiens Mcm7
NP 997734.1 Danio rerio Mcm7
P43299.2 Arabidopsis thaliana Mcm7
Q2QNM1.1 Oryza sativa Mcm7
P38132.4 Saccharomyces cereviceae Mcm7
Semro comp70058 c0 seq2 Seminavis robusta Mcm7
jgi|Phatr2|13243 Phaeodactylum tricornutum Mcm7
jgi|Thaps3|262526 Thalassiosira pseudonana Mcm7
jgi|Fracy1|184349 Fragilariopsis cylindrus Mcm7
jgi|Psemu1|243980 Pseudo-nitzschia multiseries Mcm7
0109820.1 Pseudo-nitzschia multistriata Mcm7
Mcm7
0068370.1 Pseudo-nitzschia multistriata Mcm8
Semro comp79168 c0 seq3 Seminavis robusta Mcm8
jgi|Fracy1|189062 Fragilariopsis cylindrus Mcm8
jgi|Psemu1|213178 Pseudo-nitzschia multiseries Mcm8
jgi|Phatr2|52561 Phaeodactylum tricornutum Mcm8
jgi|Thaps3|261512 Thalassiosira pseudonana Mcm8
NP 187577.1 Arabidopsis thaliana Mcm8
B9FKM7. Oryza sativa Mcm8
NP 001268449.1 Homo sapiens Mcm8
XP 002665161.2 Danio rerio Mcm8
Mcm8
NP 179021.3 Arabidopsis thaliana Mcm9
B8B406.1 Oryza sativa Mcm9
F1QDI9.2 Danio rerio Mcm9
NP 060166.2 Homo sapiens Mcm9
jgi|Thaps3|37362 Thalassiosira pseudonana Mcm9
jgi|Phatr2|981 Phaeodactylum tricornutum Mcm9
Semro comp59174 c0 seq1 Seminavis robusta Mcm9
jgi|Fracy1|156569 Fragilariopsis cylindrus Mcm9
jgi|Psemu1|183315 Pseudo-nitzschia multiseries Mcm9
0056900.1 Pseudo-nitzschia multistriata Mcm9
Mcm9
NP 004517.2 Homo sapiens Mcm2
NP 775364.1 Danio rerio Mcm2
NP 001185154.1 Arabidopsis thaliana Mcm2
B8BKI8.1 Oryza sativa Mcm2
CAA37615.1 Saccharomyces cerevisiae Mcm2
jgi|Thaps3|29936 Thalassiosira pseudonana Mcm2
Semro comp78811 c0 seq1 Seminavis robusta Mcm2
jgi|Phatr2|18622 Phaeodactylum tricornutum Mcm2
jgi|Fracy1|204899 Fragilariopsis cylindrus Mcm2
jgi|Psemu1|209470 Pseudo-nitzschia multiseries Mcm2
0061270.1 Pseudo-nitzschia multistriata Mcm2
Mcm2
NP 179694.2 Arabidopsis thaliana Mcm10
BAD46568.1 Oryza sativa Mcm10
P32354.2 Saccharomyces cereviceae Mcm10
0.5

Figure S1. Phylogenetic tree of Mcm proteins inferred from maximum likelihood analysis. Numbers on the branches indicate bootstrap support from 1000 replicates. Among-site substitution rate heterogeneity was corrected using two gamma distributed substitution rate categories and LG substitution model for amino acid substitutions (LG+G). Diatom sequences are indicated in bold. The codes before the taxon name are the corresponding sequence IDs (For respective databases refer to materials and methods section).

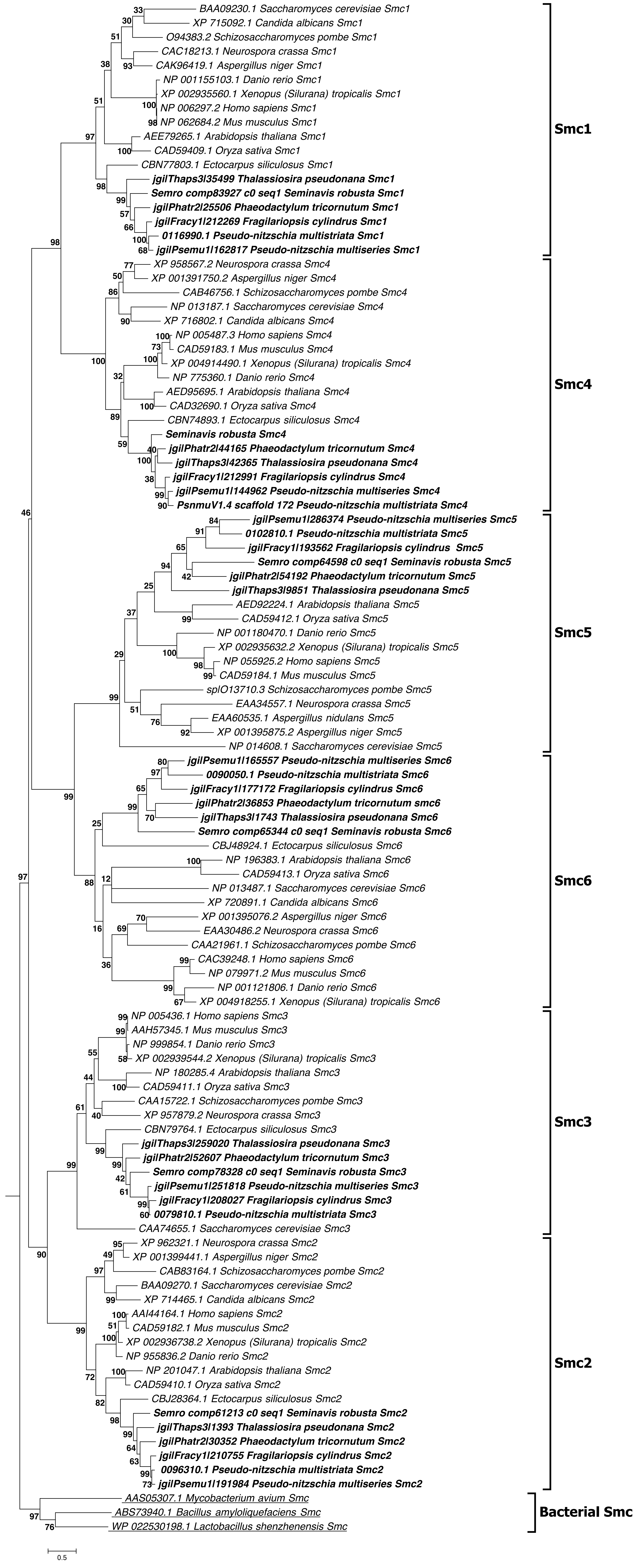
BAA09230.1 Saccharomyces cerevisiae Smc1
XP 715092.1 Candida albicans Smc1
O94383.2 Schizosaccharomyces pombe Smc1
CAC18213.1 Neurospora crassa Smc1
CAK96419.1 Aspergillus niger Smc1
NP 001155103.1 Danio rerio Smc1
XP 002935560.1 Xenopus (Silurana) tropicalis Smc1
NP 006297.2 Homo sapiens Smc1
NP 062684.2 Mus musculus Smc1
AEE79265.1 Arabidopsis thaliana Smc1
CAD59409.1 Oryza sativa Smc1
CBN77803.1 Ectocarpus siliculosus Smc1
jgi|Thaps3|35499 Thalassiosira pseudonana Smc1
Semro comp83927 c0 seq1 Seminavis robusta Smc1
jgi|Phatr2|25506 Phaeodactylum tricornutum Smc1
jgi|Fracy1|212269 Fragilariopsis cylindrus Smc1
0116990.1 Pseudo-nitzschia multistriata Smc1
jgi|Psemu1|162817 Pseudo-nitzschia multiseries Smc1
Smc1
XP 958567.2 Neurospora crassa Smc4
XP 001391750.2 Aspergillus niger Smc4
CAB46756.1 Schizosaccharomyces pombe Smc4
NP 013187.1 Saccharomyces cerevisiae Smc4
XP 716802.1 Candida albicans Smc4
NP 005487.3 Homo sapiens Smc4
CAD59183.1 Mus musculus Smc4
XP 004914490.1 Xenopus (Silurana) tropicalis Smc4
NP 775360.1 Danio rerio Smc4
AED95695.1 Arabidopsis thaliana Smc4
CAD32690.1 Oryza sativa Smc4
CBN74893.1 Ectocarpus siliculosus Smc4
Seminavis robusta Smc4
jgi|Phatr2|44165 Phaeodactylum tricornutum Smc4
jgi|Thaps3|42365 Thalassiosira pseudonana Smc4
jgi|Fracy1|212991 Fragilariopsis cylindrus Smc4
jgi|Psemu1|144962 Pseudo-nitzschia multiseries Smc4
PsnmuV1.4 scaffold 172 Pseudo-nitzschia multistriata Smc4
Smc4
jgi|Psemu1|286374 Pseudo-nitzschia multiseries Smc5
0102810.1 Pseudo-nitzschia multistriata Smc5
jgi|Fracy1|193562 Fragilariopsis cylindrus Smc5
Semro comp64598 c0 seq1 Seminavis robusta Smc5
jgi|Phatr2|54192 Phaeodactylum tricornutum Smc5
jgi|Thaps3|9851 Thalassiosira pseudonana Smc5
AED92224.1 Arabidopsis thaliana Smc5
CAD59412.1 Oryza sativa Smc5
NP 001180470.1 Danio rerio Smc5
XP 002935632.2 Xenopus (Silurana) tropicalis Smc5
NP 055925.2 Homo sapiens Smc5
CAD59184.1 Mus musculus Smc5
sp|O13710.3 Schizosaccharomyces pombe Smc5
EAA34557.1 Neurospora crassa Smc5
EAA60535.1 Aspergillus nidulans Smc5
XP 001395875.2 Aspergillus niger Smc5
NP 014608.1 Saccharomyces cerevisiae Smc5
Smc5
jgi|Psemu1|165557 Pseudo-nitzschia multiseries Smc6
0090050.1 Pseudo-nitzschia multistriata Smc6
jgi|Fracy1|177172 Fragilariopsis cylindrus Smc6
jgi|Phatr2|36853 Phaeodactylum tricornutum smc6
jgi|Thaps3|1743 Thalassiosira pseudonana Smc6
Semro comp65344 c0 seq1 Seminavis robusta Smc6
CBJ48924.1 Ectocarpus siliculosus Smc6
NP 196383.1 Arabidopsis thaliana Smc6
CAD59413.1 Oryza sativa Smc6
NP 013487.1 Saccharomyces cerevisiae Smc6
XP 720891.1 Candida albicans Smc6
XP 001395076.2 Aspergillus niger Smc6
EAA30486.2 Neurospora crassa Smc6
CAA21961.1 Schizosaccharomyces pombe Smc6
CAC39248.1 Homo sapiens Smc6
NP 079971.2 Mus musculus Smc6
NP 001121806.1 Danio rerio Smc6
XP 004918255.1 Xenopus (Silurana) tropicalis Smc6
Smc6
NP 005436.1 Homo sapiens Smc3
AAH57345.1 Mus musculus Smc3
NP 999854.1 Danio rerio Smc3
XP 002939544.2 Xenopus (Silurana) tropicalis Smc3
NP 180285.4 Arabidopsis thaliana Smc3
CAD59411.1 Oryza sativa Smc3
CAA15722.1 Schizosaccharomyces pombe Smc3
XP 957879.2 Neurospora crassa Smc3
CBN79764.1 Ectocarpus siliculosus Smc3
jgi|Thaps3|259020 Thalassiosira pseudonana Smc3
jgi|Phatr2|52607 Phaeodactylum tricornutum Smc3
Semro comp78328 c0 seq1 Seminavis robusta Smc3
jgi|Psemu1|251818 Pseudo-nitzschia multiseries Smc3
jgi|Fracy1|208027 Fragilariopsis cylindrus Smc3
0079810.1 Pseudo-nitzschia multistriata Smc3
CAA74655.1 Saccharomyces cerevisiae Smc3
Smc3
XP 962321.1 Neurospora crassa Smc2
XP 001399441.1 Aspergillus niger Smc2
CAB83164.1 Schizosaccharomyces pombe Smc2
BAA09270.1 Saccharomyces cerevisiae Smc2
XP 714465.1 Candida albicans Smc2
AAI44164.1 Homo sapiens Smc2
CAD59182.1 Mus musculus Smc2
XP 002936738.2 Xenopus (Silurana) tropicalis Smc2
NP 955836.2 Danio rerio Smc2
NP 201047.1 Arabidopsis thaliana Smc2
CAD59410.1 Oryza sativa Smc2
CBJ28364.1 Ectocarpus siliculosus Smc2
Semro comp61213 c0 seq1 Seminavis robusta Smc2
jgi|Thaps3|1393 Thalassiosira pseudonana Smc2
jgi|Phatr2|30352 Phaeodactylum tricornutum Smc2
jgi|Fracy1|210755 Fragilariopsis cylindrus Smc2
0096310.1 Pseudo-nitzschia multistriata Smc2
jgi|Psemu1|191984 Pseudo-nitzschia multiseries Smc2
Smc2
AAS05307.1 Mycobacterium avium Smc
ABS73940.1 Bacillus amyloliquefaciens Smc
WP 022530198.1 Lactobacillus shenzhenensis Smc
Bacterial Smc
0.5

Figure S2. Phylogenetic tree of Smc proteins inferred from maximum likelihood analysis, rooted with bacterial Smc proteins as an outgroup (underlined). Numbers on the branches indicate bootstrap support from 1000 replicates. Among-site substitution rate heterogeneity was corrected using two gamma distributed substitution rate categories and LG with frequencies substitution model for amino acid substitutions (LG+G+F). Diatom sequences are indicated in bold. The codes before the taxon name are the corresponding sequence IDs (For respective databases refer to materials and methods section).

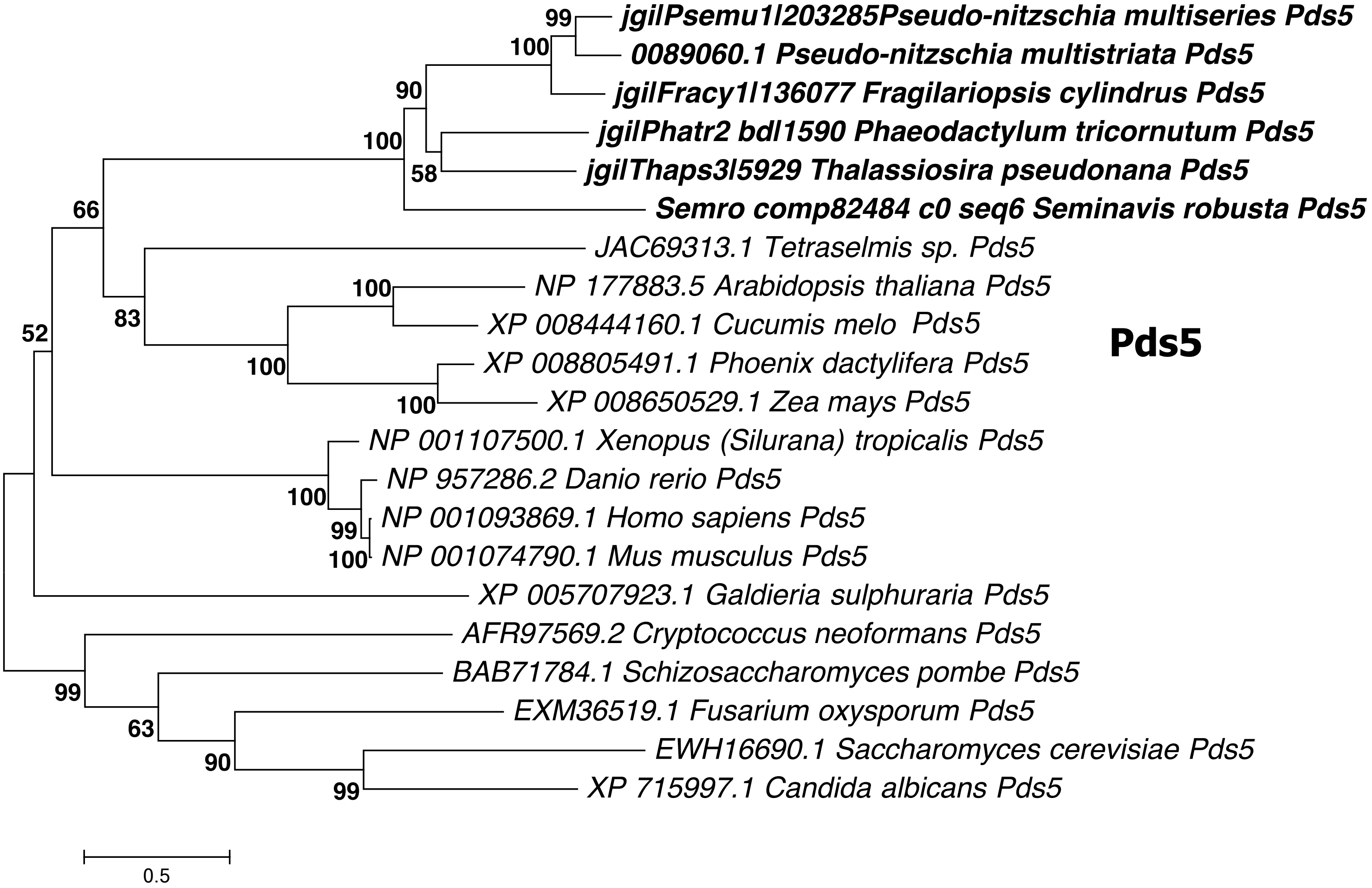

jgi|Psemu1|203285Pseudo-nitzschia multiseries Pds5
0089060.1 Pseudo-nitzschia multistriata Pds5
jgi|Fracy1|136077 Fragilariopsis cylindrus Pds5
jgi|Phatr2 bd|1590 Phaeodactylum tricornutum Pds5
jgi|Thaps3|5929 Thalassiosira pseudonana Pds5
Semro comp82484 c0 seq6 Seminavis robusta Pds5
JAC69313.1 Tetraselmis sp. Pds5
NP 177883.5 Arabidopsis thaliana Pds5
XP 008444160.1 Cucumis melo Pds5
XP 008805491.1 Phoenix dactylifera Pds5
XP 008650529.1 Zea mays Pds5
NP 001107500.1 Xenopus (Silurana) tropicalis Pds5
NP 957286.2 Danio rerio Pds5
NP 001093869.1 Homo sapiens Pds5
NP 001074790.1 Mus musculus Pds5
XP 005707923.1 Galdieria sulphuraria Pds5
AFR97569.2 Cryptococcus neoformans Pds5
BAB71784.1 Schizosaccharomyces pombe Pds5
EXM36519.1 Fusarium oxysporum Pds5
EWH16690.1 Saccharomyces cerevisiae Pds5
XP 715997.1 Candida albicans Pds5
Pds5
99
100
90
100
58
66
83
100
100
100
52
100
99
100
99
63
90
99
0.5

Figure S3. Phylogenetic tree of Pds5 proteins inferred from maximum likelihood analysis. Numbers on the branches indicate bootstrap support from 1000 replicates. Among-site substitution rate heterogeneity was corrected using three gamma distributed substitution rate categories and LG substitution model for amino acid substitutions (LG+G). Diatom sequences are indicated in bold. The codes before the taxon name are the corresponding sequence IDs (For respective databases refer to materials and methods section).

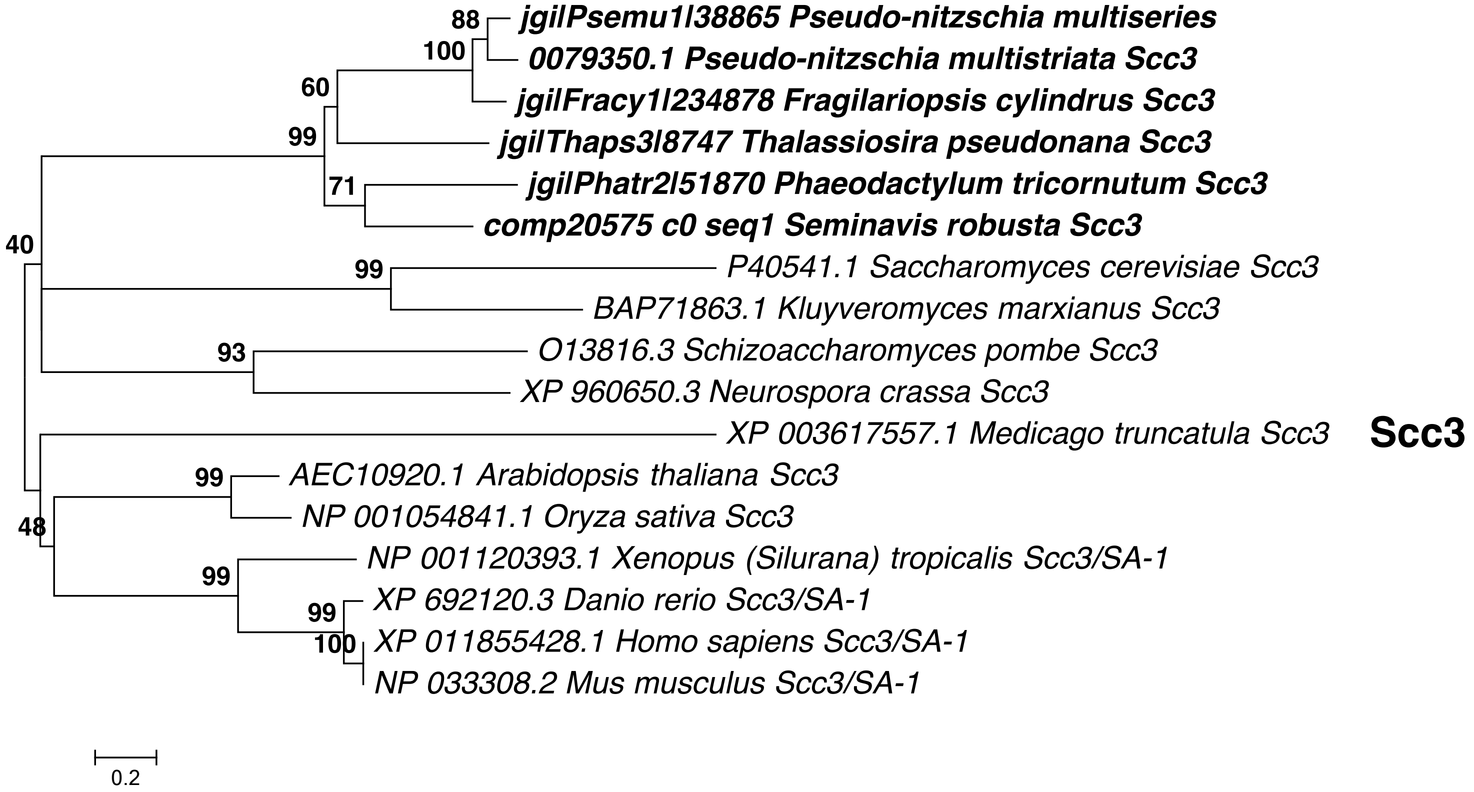

88
100
60
99
71
40
99
93
99
48
99
99
100
jgi|Psemu1|38865 Pseudo-nitzschia multiseries
0079350.1 Pseudo-nitzschia multistriata Scc3
jgi|Fracy1|234878 Fragilariopsis cylindrus Scc3
jgi|Thaps3|8747 Thalassiosira pseudonana Scc3
jgi|Phatr2|51870 Phaeodactylum tricornutum Scc3
comp20575 c0 seq1 Seminavis robusta Scc3
P40541.1 Saccharomyces cerevisiae Scc3
BAP71863.1 Kluyveromyces marxianus Scc3
O13816.3 Schizoaccharomyces pombe Scc3
XP 960650.3 Neurospora crassa Scc3
XP 003617557.1 Medicago truncatula Scc3
AEC10920.1 Arabidopsis thaliana Scc3
NP 001054841.1 Oryza sativa Scc3
NP 001120393.1 Xenopus (Silurana) tropicalis Scc3/SA-1
XP 692120.3 Danio rerio Scc3/SA-1
XP 011855428.1 Homo sapiens Scc3/SA-1
NP 033308.2 Mus musculus Scc3/SA-1
Scc3
0.2

Figure S4. Phylogenetic tree of Scc3 proteins inferred from maximum likelihood analysis. Numbers on the branches indicate bootstrap support from 1000 replicates. Among-site substitution rate heterogeneity was corrected using an invariable and three gamma distributed substitution rate categories and LG substitution model for amino acid substitutions (LG+G+I). Diatom sequences are indicated in bold. The codes before the taxon name are the corresponding sequence IDs (For respective databases refer to materials and methods section).

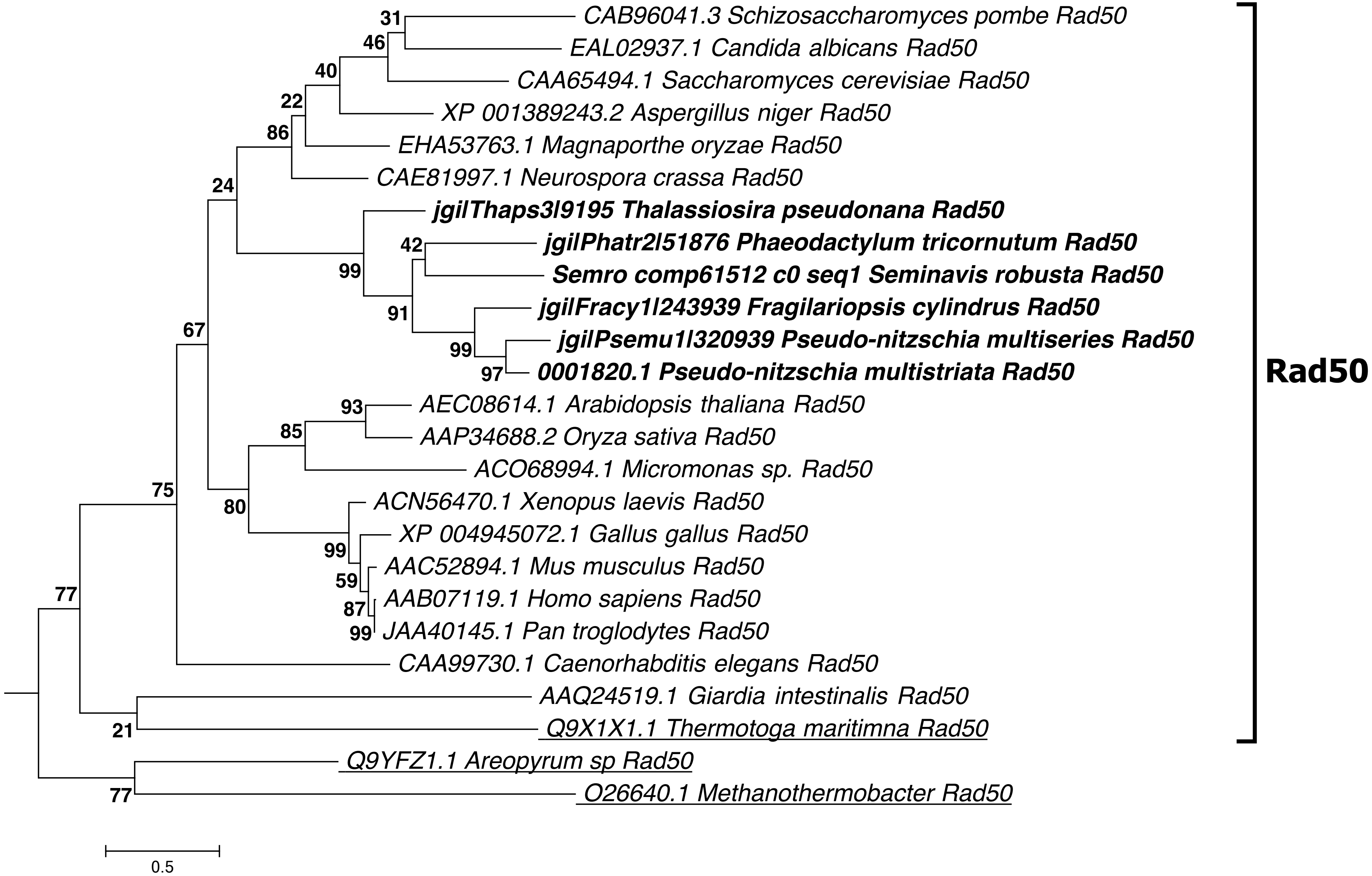
CAB96041.3 Schizosaccharomyces pombe Rad50
EAL02937.1 Candida albicans Rad50
CAA65494.1 Saccharomyces cerevisiae Rad50
XP 001389243.2 Aspergillus niger Rad50
EHA53763.1 Magnaporthe oryzae Rad50
CAE81997.1 Neurospora crassa Rad50
jgi|Thaps3|9195 Thalassiosira pseudonana Rad50
jgi|Phatr2|51876 Phaeodactylum tricornutum Rad50
Semro comp61512 c0 seq1 Seminavis robusta Rad50
jgi|Fracy1|243939 Fragilariopsis cylindrus Rad50
jgi|Psemu1|320939 Pseudo-nitzschia multiseries Rad50
0001820.1 Pseudo-nitzschia multistriata Rad50
AEC08614.1 Arabidopsis thaliana Rad50
AAP34688.2 Oryza sativa Rad50
ACO68994.1 Micromonas sp. Rad50
ACN56470.1 Xenopus laevis Rad50
XP 004945072.1 Gallus gallus Rad50
AAC52894.1 Mus musculus Rad50
AAB07119.1 Homo sapiens Rad50
JAA40145.1 Pan troglodytes Rad50
CAA99730.1 Caenorhabditis elegans Rad50
AAQ24519.1 Giardia intestinalis Rad50
Q9X1X1.1 Thermotoga maritimna Rad50
Q9YFZ1.1 Areopyrum sp Rad50
O26640.1 Methanothermobacter Rad50
Rad50
31
46
40
22
86
24
42
99
91
99
97
67
93
85
75
80
99
59
87
99
77
21
77
0.5

Figure S5. Phylogenetic tree of Rad50 proteins inferred from maximum likelihood analysis, rooted with bacterial Rad50 proteins as an outgroup (underlined). Numbers on the branches indicate bootstrap support from 1000 replicates. Among-site substitution rate heterogeneity was corrected using two gamma distributed substitution rate categories and LG with frequencies substitution model for amino acid substitutions (LG+G+F). Diatom sequences are indicated in bold. The codes before the taxon name are the corresponding sequence IDs (For respective databases refer to materials and methods section).

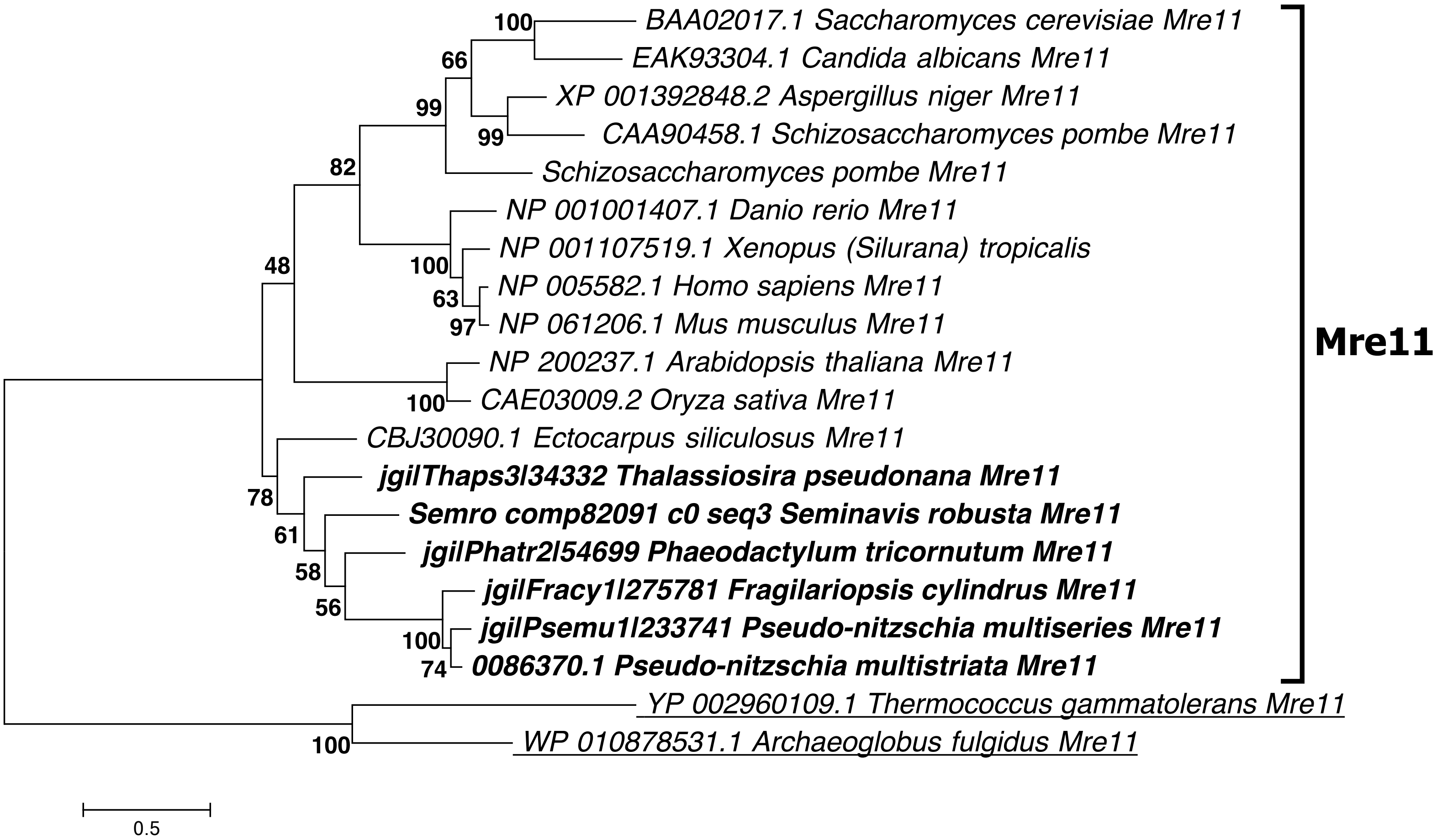

BAA02017.1 Saccharomyces cerevisiae Mre11
EAK93304.1 Candida albicans Mre11
XP 001392848.2 Aspergillus niger Mre11
CAA90458.1 Schizosaccharomyces pombe Mre11
Schizosaccharomyces pombe Mre11
NP 001001407.1 Danio rerio Mre11
NP 001107519.1 Xenopus (Silurana) tropicalis
NP 005582.1 Homo sapiens Mre11
NP 061206.1 Mus musculus Mre11
NP 200237.1 Arabidopsis thaliana Mre11
CAE03009.2 Oryza sativa Mre11
CBJ30090.1 Ectocarpus siliculosus Mre11
jgi|Thaps3|34332 Thalassiosira pseudonana Mre11
Semro comp82091 c0 seq3 Seminavis robusta Mre11
jgi|Phatr2|54699 Phaeodactylum tricornutum Mre11
jgi|Fracy1|275781 Fragilariopsis cylindrus Mre11
jgi|Psemu1|233741 Pseudo-nitzschia multiseries Mre11
0086370.1 Pseudo-nitzschia multistriata Mre11
YP 002960109.1 Thermococcus gammatolerans Mre11
WP 010878531.1 Archaeoglobus fulgidus Mre11
Mre11
100
66
99
99
82
100
63
97
48
100
78
61
58
56
100
74
100
0.5

Figure S6. Phylogenetic tree of Mre11 proteins inferred from maximum likelihood analysis, rooted with archaeal Mre11 proteins as an outgroup (underlined). Numbers on the branches indicate bootstrap support from 1000 replicates. Among-site substitution rate heterogeneity was corrected using two gamma distributed substitution rate categories and LG substitution model for amino acid substitutions (LG+G). Diatom sequences are indicated in bold. The codes before the taxon name are the corresponding sequence IDs (For respective databases refer to materials and methods section).

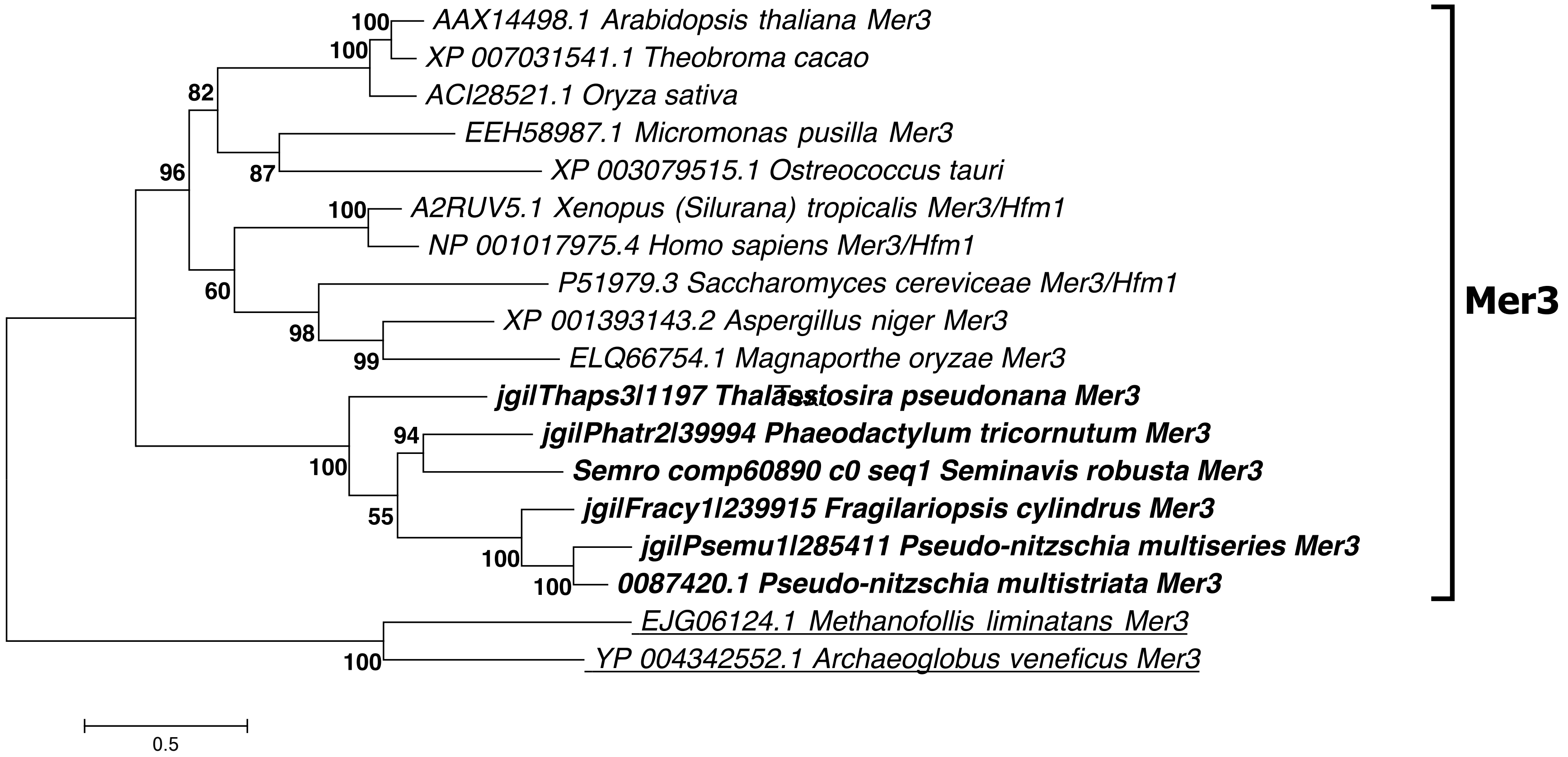

AAX14498.1 Arabidopsis thaliana Mer3
XP 007031541.1 Theobroma cacao
ACI28521.1 Oryza sativa
EEH58987.1 Micromonas pusilla Mer3
XP 003079515.1 Ostreococcus tauri
A2RUV5.1 Xenopus (Silurana) tropicalis Mer3/Hfm1
NP 001017975.4 Homo sapiens Mer3/Hfm1
P51979.3 Saccharomyces cereviceae Mer3/Hfm1
XP 001393143.2 Aspergillus niger Mer3
ELQ66754.1 Magnaporthe oryzae Mer3
jgilThaps3l1197 Thalassiosira pseudonana Mer3
jgilPhatr2l39994 Phaeodactylum tricornutum Mer3
Semro comp60890 c0 seq1 Seminavis robusta Mer3
jgilFracy1l239915 Fragilariopsis cylindrus Mer3
jgilPsemu1l285411 Pseudo-nitzschia multiseries Mer3
0087420.1 Pseudo-nitzschia multistriata Mer3
EJG06124.1 Methanofollis liminatans Mer3
YP 004342552.1 Archaeoglobus veneficus Mer3
100
100
82
96
87
100
60
98
99
94
100
55
100
100
100
Mer3
0.5

Figure S7. Phylogenetic tree of Mer3 proteins inferred from maximum likelihood analysis, rooted with archaeal Mer3 proteins as an outgroup (underlined). Numbers on the branches indicate bootstrap support from 1000 replicates. Among-site substitution rate heterogeneity was corrected using an invariable and two gamma distributed substitution rate categories and LG substitution model for amino acid substitutions (LG+G+I). Diatom sequences are indicated in bold. The codes before the taxon name are the corresponding sequence IDs (For respective databases refer to materials and methods section).

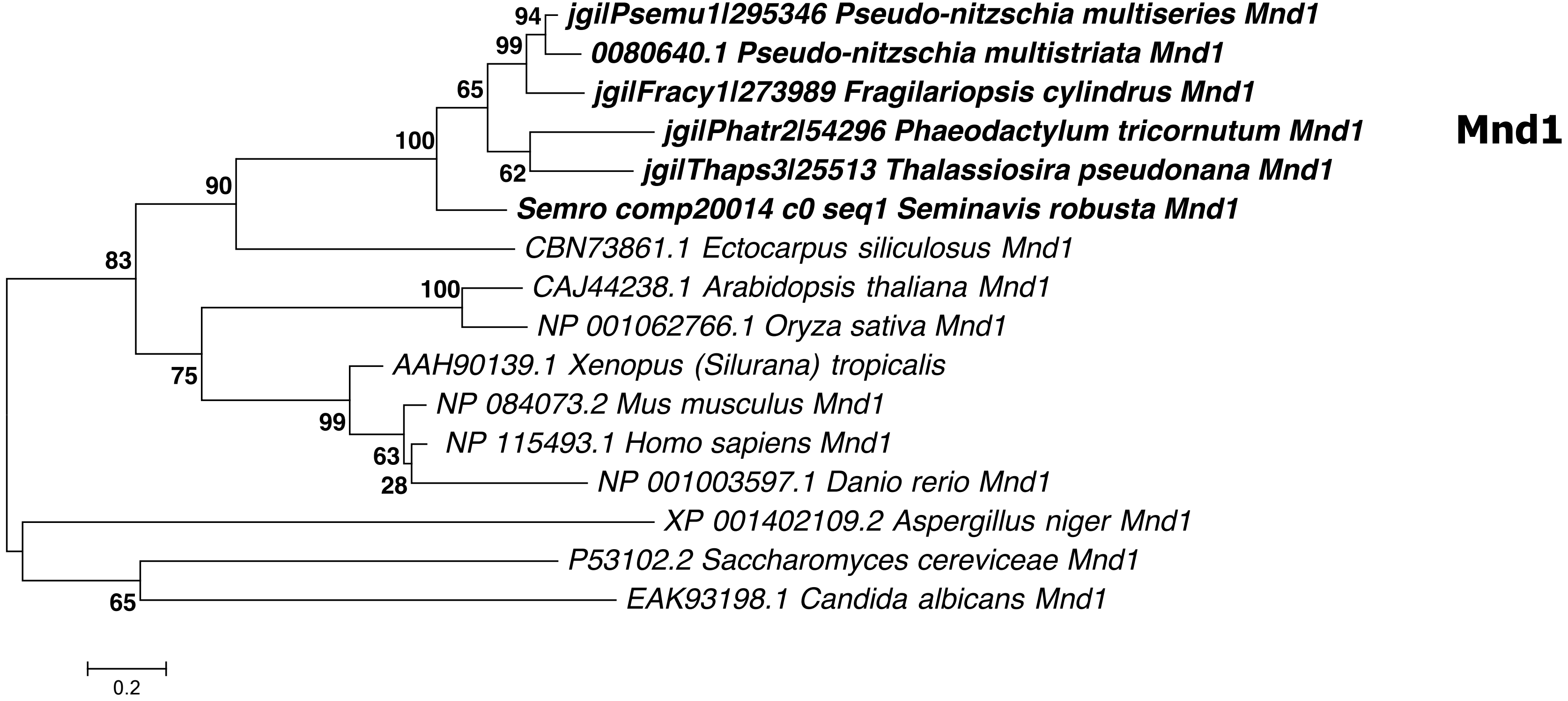
94
jgi|Psemu1|295346 Pseudo-nitzschia multiseries Mnd1
99
0080640.1 Pseudo-nitzschia multistriata Mnd1
65
jgi|Fracy1|273989 Fragilariopsis cylindrus Mnd1
100
jgi|Phatr2|54296 Phaeodactylum tricornutum Mnd1
Mnd1
62
jgi|Thaps3|25513 Thalassiosira pseudonana Mnd1
90
Semro comp20014 c0 seq1 Seminavis robusta Mnd1
83
CBN73861.1 Ectocarpus siliculosus Mnd1
100
CAJ44238.1 Arabidopsis thaliana Mnd1
NP 001062766.1 Oryza sativa Mnd1
75
AAH90139.1 Xenopus (Silurana) tropicalis
NP 084073.2 Mus musculus Mnd1
99
NP 115493.1 Homo sapiens Mnd1
63
28
NP 001003597.1 Danio rerio Mnd1
XP 001402109.2 Aspergillus niger Mnd1
P53102.2 Saccharomyces cereviceae Mnd1
65
EAK93198.1 Candida albicans Mnd1
0.2

Figure S8. Phylogenetic tree of Mnd1 proteins inferred from maximum likelihood analysis. Numbers on the branches indicate bootstrap support from 1000 replicates. Among-site substitution rate heterogeneity was corrected using two gamma distributed substitution rate categories and LG substitution model for amino acid substitutions (LG+G). Diatom sequences are indicated in bold. The codes before the taxon name are the corresponding sequence IDs (For respective databases refer to materials and methods section).

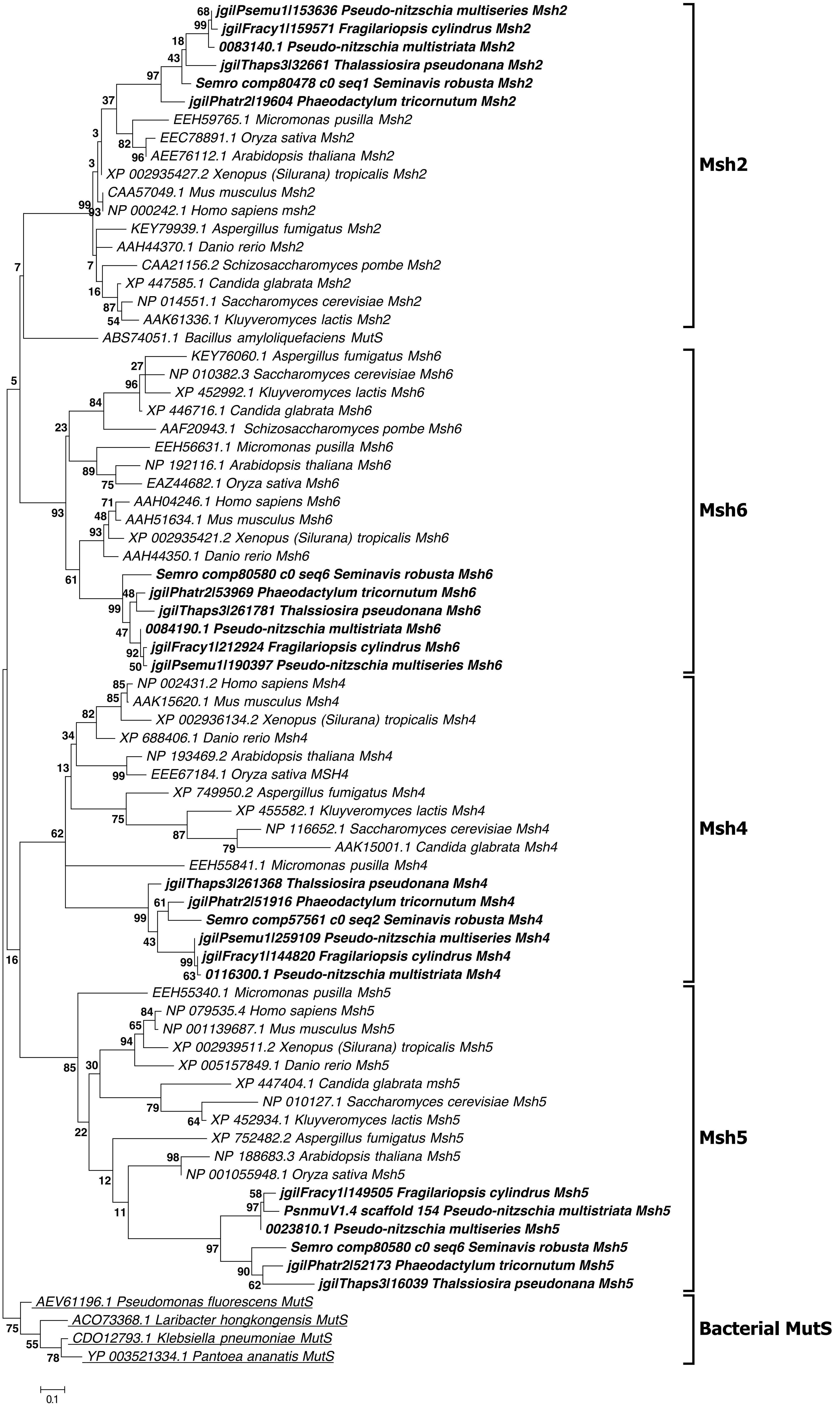
jgi|Psemu1|153636 Pseudo-nitzschia multiseries Msh2
jgi|Fracy1|159571 Fragilariopsis cylindrus Msh2
0083140.1 Pseudo-nitzschia multistriata Msh2
jgi|Thaps3|32661 Thalassiosira pseudonana Msh2
Semro comp80478 c0 seq1 Seminavis robusta Msh2
jgi|Phatr2|19604 Phaeodactylum tricornutum Msh2
EEH59765.1 Micromonas pusilla Msh2
EEC78891.1 Oryza sativa Msh2
AEE76112.1 Arabidopsis thaliana Msh2
XP 002935427.2 Xenopus (Silurana) tropicalis Msh2
CAA57049.1 Mus musculus Msh2
NP 000242.1 Homo sapiens msh2
KEY79939.1 Aspergillus fumigatus Msh2
AAH44370.1 Danio rerio Msh2
CAA21156.2 Schizosaccharomyces pombe Msh2
XP 447585.1 Candida glabrata Msh2
NP 014551.1 Saccharomyces cerevisiae Msh2
AAK61336.1 Kluyveromyces lactis Msh2
ABS74051.1 Bacillus amyloliquefaciens MutS
KEY76060.1 Aspergillus fumigatus Msh6
NP 010382.3 Saccharomyces cerevisiae Msh6
XP 452992.1 Kluyveromyces lactis Msh6
XP 446716.1 Candida glabrata Msh6
AAF20943.1 Schizosaccharomyces pombe Msh6
EEH56631.1 Micromonas pusilla Msh6
NP 192116.1 Arabidopsis thaliana Msh6
EAZ44682.1 Oryza sativa Msh6
AAH04246.1 Homo sapiens Msh6
AAH51634.1 Mus musculus Msh6
XP 002935421.2 Xenopus (Silurana) tropicalis Msh6
AAH44350.1 Danio rerio Msh6
Semro comp80580 c0 seq6 Seminavis robusta Msh6
jgi|Phatr2|53969 Phaeodactylum tricornutum Msh6
jgi|Thaps3|261781 Thalssiosira pseudonana Msh6
0084190.1 Pseudo-nitzschia multistriata Msh6
jgi|Fracy1|212924 Fragilariopsis cylindrus Msh6
jgi|Psemu1|190397 Pseudo-nitzschia multiseries Msh6
NP 002431.2 Homo sapiens Msh4
AAK15620.1 Mus musculus Msh4
XP 002936134.2 Xenopus (Silurana) tropicalis Msh4
XP 688406.1 Danio rerio Msh4
NP 193469.2 Arabidopsis thaliana Msh4
EEE67184.1 Oryza sativa MSH4
XP 749950.2 Aspergillus fumigatus Msh4
XP 455582.1 Kluyveromyces lactis Msh4
NP 116652.1 Saccharomyces cerevisiae Msh4
AAK15001.1 Candida glabrata Msh4
EEH55841.1 Micromonas pusilla Msh4
jgi|Thaps3|261368 Thalssiosira pseudonana Msh4
jgi|Phatr2|51916 Phaeodactylum tricornutum Msh4
Semro comp57561 c0 seq2 Seminavis robusta Msh4
jgi|Psemu1|259109 Pseudo-nitzschia multiseries Msh4
jgi|Fracy1|144820 Fragilariopsis cylindrus Msh4
0116300.1 Pseudo-nitzschia multistriata Msh4
EEH55340.1 Micromonas pusilla Msh5
NP 079535.4 Homo sapiens Msh5
NP 001139687.1 Mus musculus Msh5
XP 002939511.2 Xenopus (Silurana) tropicalis Msh5
XP 005157849.1 Danio rerio Msh5
XP 447404.1 Candida glabrata msh5
NP 010127.1 Saccharomyces cerevisiae Msh5
XP 452934.1 Kluyveromyces lactis Msh5
XP 752482.2 Aspergillus fumigatus Msh5
NP 188683.3 Arabidopsis thaliana Msh5
NP 001055948.1 Oryza sativa Msh5
jgi|Fracy1|149505 Fragilariopsis cylindrus Msh5
PsnmuV1.4 scaffold 154 Pseudo-nitzschia multistriata Msh5
0023810.1 Pseudo-nitzschia multiseries Msh5
Semro comp80580 c0 seq6 Seminavis robusta Msh5
jgi|Phatr2|52173 Phaeodactylum tricornutum Msh5
jgi|Thaps3|16039 Thalssiosira pseudonana Msh5
AEV61196.1 Pseudomonas fluorescens MutS
ACO73368.1 Laribacter hongkongensis MutS
CDO12793.1 Klebsiella pneumoniae MutS
YP 003521334.1 Pantoea ananatis MutS
Msh2
Msh6
Msh4
Msh5
Bacterial MutS
0.1

Figure S9. Phylogenetic tree of Msh proteins inferred from maximum likelihood analysis, rooted with bacterial MutS proteins as an outgroup (underlined). Numbers on the branches indicate bootstrap support from 1000 replicates. Among-site substitution rate heterogeneity was corrected using two gamma distributed substitution rate categories and LG substitution model for amino acid substitutions (LG+G). Diatom sequences are indicated in bold. The codes before the taxon name are the corresponding sequence IDs (For respective databases refer to materials and methods section).

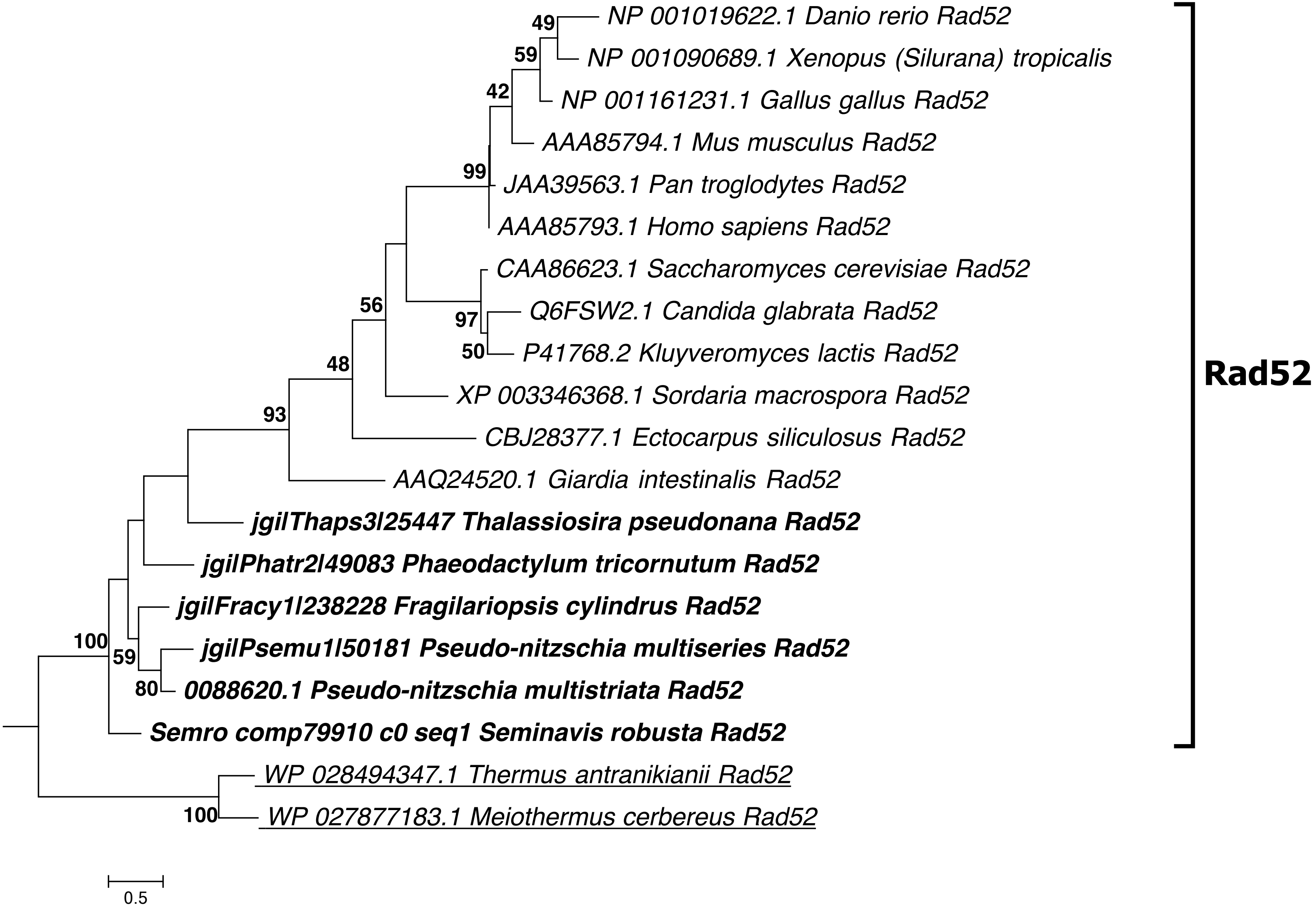

NP 001019622.1 Danio rerio Rad52
NP 001090689.1 Xenopus (Silurana) tropicalis
NP 001161231.1 Gallus gallus Rad52
AAA85794.1 Mus musculus Rad52
JAA39563.1 Pan troglodytes Rad52
AAA85793.1 Homo sapiens Rad52
CAA86623.1 Saccharomyces cerevisiae Rad52
Q6FSW2.1 Candida glabrata Rad52
P41768.2 Kluyveromyces lactis Rad52
XP 003346368.1 Sordaria macrospora Rad52
CBJ28377.1 Ectocarpus siliculosus Rad52
AAQ24520.1 Giardia intestinalis Rad52
jgi|Thaps3|25447 Thalassiosira pseudonana Rad52
jgi|Phatr2|49083 Phaeodactylum tricornutum Rad52
jgi|Fracy1|238228 Fragilariopsis cylindrus Rad52
jgi|Psemu1|50181 Pseudo-nitzschia multiseries Rad52
0088620.1 Pseudo-nitzschia multistriata Rad52
Semro comp79910 c0 seq1 Seminavis robusta Rad52
WP 028494347.1 Thermus antranikianii Rad52
WP 027877183.1 Meiothermus cerbereus Rad52
Rad52
49
59
42
99
97
50
56
48
93
100
59
80
100
0.5

Figure S10. Phylogenetic tree of Rad52 proteins inferred from maximum likelihood analysis, rooted with archaeal Rad52 proteins as an outgroup (underlined). Numbers on the branches indicate bootstrap support from 1000 replicates. Among-site substitution rate heterogeneity was corrected using two gamma distributed substitution rate categories and JTT substitution model for amino acid substitutions (JTT+G). Diatom sequences are indicated in bold. The codes before the taxon name are the corresponding sequence IDs (For respective databases refer to materials and methods section).

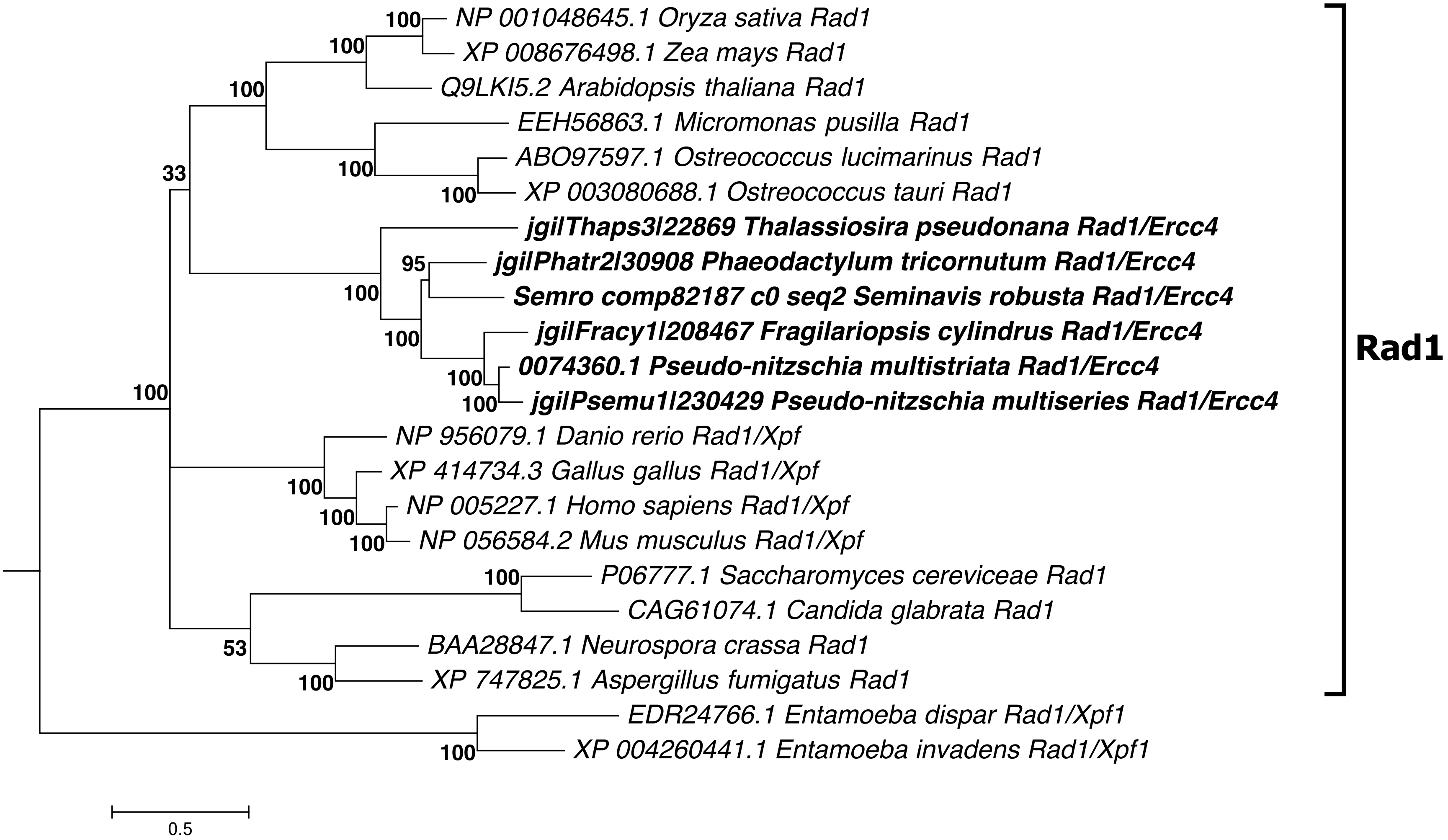
100
NP 001048645.1 Oryza sativa Rad1
100
XP 008676498.1 Zea mays Rad1
100
Q9LKI5.2 Arabidopsis thaliana Rad1
EEH56863.1 Micromonas pusilla Rad1
100
ABO97597.1 Ostreococcus lucimarinus Rad1
33
100
XP 003080688.1 Ostreococcus tauri Rad1
jgi|Thaps3|22869 Thalassiosira pseudonana Rad1/Ercc4
95
jgi|Phatr2|30908 Phaeodactylum tricornutum Rad1/Ercc4
100
Semro comp82187 c0 seq2 Seminavis robusta Rad1/Ercc4
100
jgi|Fracy1|208467 Fragilariopsis cylindrus Rad1/Ercc4
100
0074360.1 Pseudo-nitzschia multistriata Rad1/Ercc4
100
100
jgi|Psemu1|230429 Pseudo-nitzschia multiseries Rad1/Ercc4
NP 956079.1 Danio rerio Rad1/Xpf
XP 414734.3 Gallus gallus Rad1/Xpf
100
NP 005227.1 Homo sapiens Rad1/Xpf
100
100
NP 056584.2 Mus musculus Rad1/Xpf
100
P06777.1 Saccharomyces cereviceae Rad1
CAG61074.1 Candida glabrata Rad1
BAA28847.1 Neurospora crassa Rad1
53
100
XP 747825.1 Aspergillus fumigatus Rad1
EDR24766.1 Entamoeba dispar Rad1/Xpf1
100
XP 004260441.1 Entamoeba invadens Rad1/Xpf1
Rad1
0.5

Figure S11. Phylogenetic tree of Rad1 proteins inferred from maximum likelihood analysis. Numbers on the branches indicate bootstrap support from 1000 replicates. Among-site substitution rate heterogeneity was corrected using an invariable two gamma distributed substitution rate categories and LG substitution model for amino acid substitutions (LG+G+I). Diatom sequences are indicated in bold. The codes before the taxon name are the corresponding sequence IDs (For respective databases refer to materials and methods section).

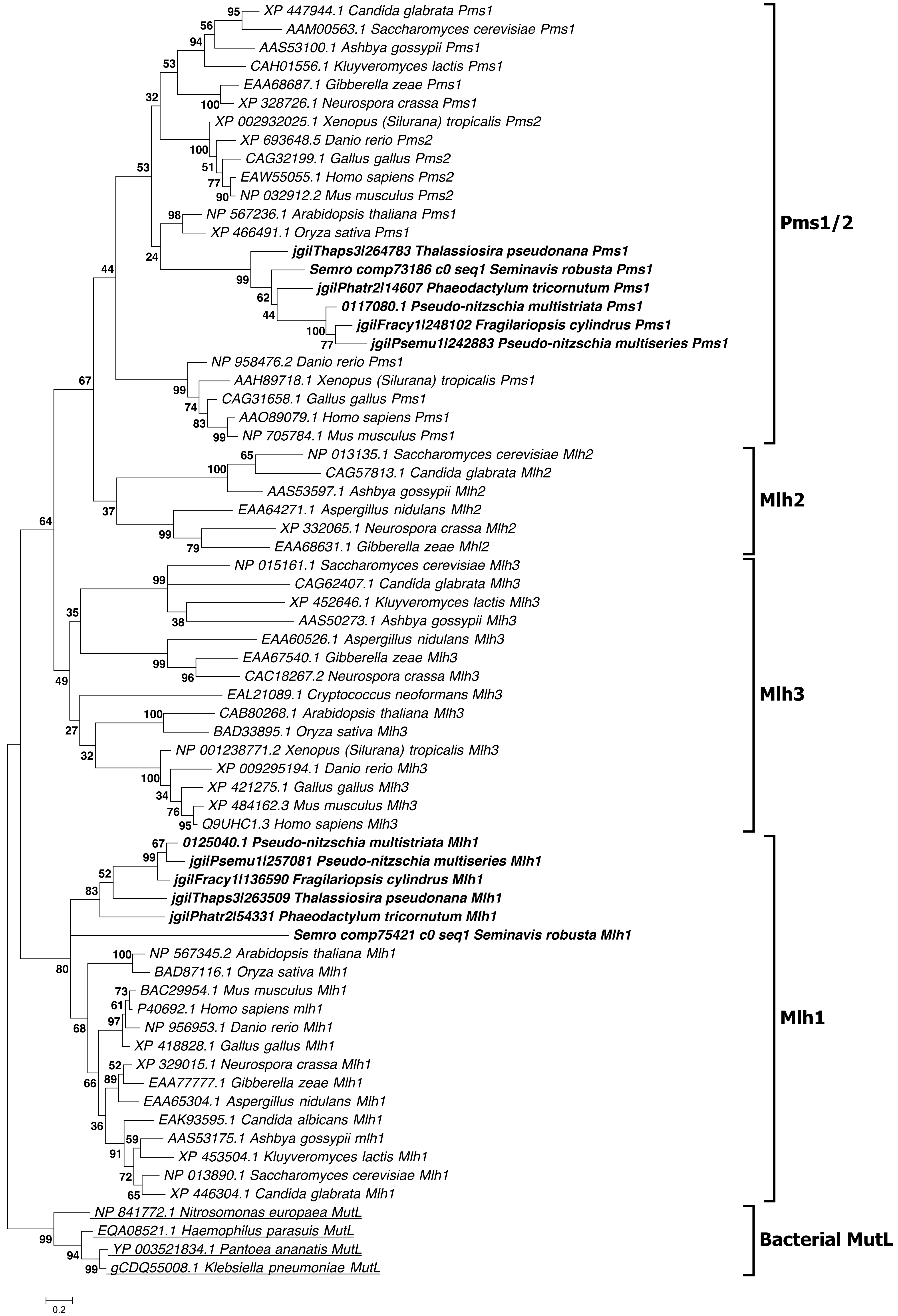

XP 447944.1 Candida glabrata Pms1
AAM00563.1 Saccharomyces cerevisiae Pms1
AAS53100.1 Ashbya gossypii Pms1
CAH01556.1 Kluyveromyces lactis Pms1
EAA68687.1 Gibberella zeae Pms1
XP 328726.1 Neurospora crassa Pms1
XP 002932025.1 Xenopus (Silurana) tropicalis Pms2
XP 693648.5 Danio rerio Pms2
CAG32199.1 Gallus gallus Pms2
EAW55055.1 Homo sapiens Pms2
NP 032912.2 Mus musculus Pms2
NP 567236.1 Arabidopsis thaliana Pms1
XP 466491.1 Oryza sativa Pms1
jgilThaps3l264783 Thalassiosira pseudonana Pms1
Semro comp73186 c0 seq1 Seminavis robusta Pms1
jgilPhatr2l14607 Phaeodactylum tricornutum Pms1
0117080.1 Pseudo-nitzschia multistriata Pms1
jgilFracy1l248102 Fragilariopsis cylindrus Pms1
jgilPsemu1l242883 Pseudo-nitzschia multiseries Pms1
NP 958476.2 Danio rerio Pms1
AAH89718.1 Xenopus (Silurana) tropicalis Pms1
CAG31658.1 Gallus gallus Pms1
AAO89079.1 Homo sapiens Pms1
NP 705784.1 Mus musculus Pms1
Pms1/2
NP 013135.1 Saccharomyces cerevisiae Mlh2
CAG57813.1 Candida glabrata Mlh2
AAS53597.1 Ashbya gossypii Mlh2
EAA64271.1 Aspergillus nidulans Mlh2
XP 332065.1 Neurospora crassa Mlh2
EAA68631.1 Gibberella zeae Mhl2
Mlh2
NP 015161.1 Saccharomyces cerevisiae Mlh3
CAG62407.1 Candida glabrata Mlh3
XP 452646.1 Kluyveromyces lactis Mlh3
AAS50273.1 Ashbya gossypii Mlh3
EAA60526.1 Aspergillus nidulans Mlh3
EAA67540.1 Gibberella zeae Mlh3
CAC18267.2 Neurospora crassa Mlh3
EAL21089.1 Cryptococcus neoformans Mlh3
CAB80268.1 Arabidopsis thaliana Mlh3
BAD33895.1 Oryza sativa Mlh3
NP 001238771.2 Xenopus (Silurana) tropicalis Mlh3
XP 009295194.1 Danio rerio Mlh3
XP 421275.1 Gallus gallus Mlh3
XP 484162.3 Mus musculus Mlh3
Q9UHC1.3 Homo sapiens Mlh3
Mlh3
0125040.1 Pseudo-nitzschia multistriata Mlh1
jgilPsemu1l257081 Pseudo-nitzschia multiseries Mlh1
jgilFracy1l136590 Fragilariopsis cylindrus Mlh1
jgilThaps3l263509 Thalassiosira pseudonana Mlh1
jgilPhatr2l54331 Phaeodactylum tricornutum Mlh1
Semro comp75421 c0 seq1 Seminavis robusta Mlh1
NP 567345.2 Arabidopsis thaliana Mlh1
BAD87116.1 Oryza sativa Mlh1
BAC29954.1 Mus musculus Mlh1
P40692.1 Homo sapiens mlh1
NP 956953.1 Danio rerio Mlh1
XP 418828.1 Gallus gallus Mlh1
XP 329015.1 Neurospora crassa Mlh1
EAA77777.1 Gibberella zeae Mlh1
EAA65304.1 Aspergillus nidulans Mlh1
EAK93595.1 Candida albicans Mlh1
AAS53175.1 Ashbya gossypii mlh1
XP 453504.1 Kluyveromyces lactis Mlh1
NP 013890.1 Saccharomyces cerevisiae Mlh1
XP 446304.1 Candida glabrata Mlh1
Mlh1
NP 841772.1 Nitrosomonas europaea MutL
EQA08521.1 Haemophilus parasuis MutL
YP 003521834.1 Pantoea ananatis MutL
gCDQ55008.1 Klebsiella pneumoniae MutL
Bacterial MutL
0.2

Figure S12. Phylogenetic tree of Mlh proteins inferred from maximum likelihood analysis, rooted with bacterial MutL proteins (underlined). Numbers on the branches indicate bootstrap support from 1000 replicates. Among-site substitution rate heterogeneity was corrected using three gamma distributed substitution rate categories and LG substitution model for amino acid substitutions (LG+G). Diatom sequences are indicated in bold. The codes before the taxon name are the corresponding sequence IDs (For respective databases refer to materials and methods section).

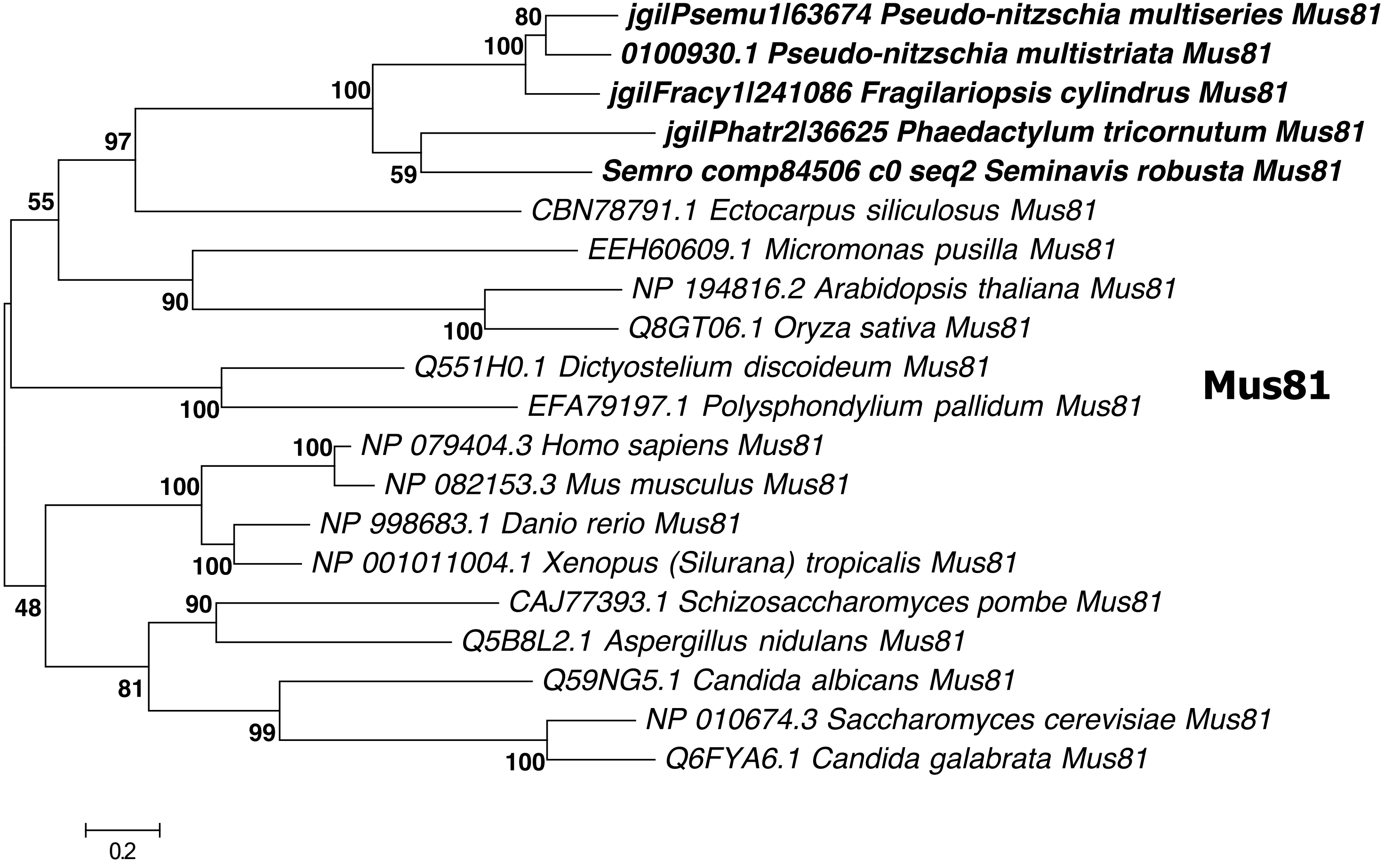

80
jgi|Psemu1|63674 Pseudo-nitzschia multiseries Mus81
100
0100930.1 Pseudo-nitzschia multistriata Mus81
100
jgi|Fracy1|241086 Fragilariopsis cylindrus Mus81
97
jgi|Phatr2|36625 Phaedactylum tricornutum Mus81
59
Semro comp84506 c0 seq2 Seminavis robusta Mus81
55
CBN78791.1 Ectocarpus siliculosus Mus81
EEH60609.1 Micromonas pusilla Mus81
90
NP 194816.2 Arabidopsis thaliana Mus81
100
Q8GT06.1 Oryza sativa Mus81
Q551H0.1 Dictyostelium discoideum Mus81
100
EFA79197.1 Polysphondylium pallidum Mus81
Mus81
100
NP 079404.3 Homo sapiens Mus81
100
NP 082153.3 Mus musculus Mus81
NP 998683.1 Danio rerio Mus81
100
NP 001011004.1 Xenopus (Silurana) tropicalis Mus81
48
90
CAJ77393.1 Schizosaccharomyces pombe Mus81
Q5B8L2.1 Aspergillus nidulans Mus81
81
Q59NG5.1 Candida albicans Mus81
99
NP 010674.3 Saccharomyces cerevisiae Mus81
100
Q6FYA6.1 Candida galabrata Mus81
0.2

Figure S13. Phylogenetic tree of Mus81 proteins inferred from maximum likelihood analysis. Numbers on the branches indicate bootstrap support from 1000 replicates. Among-site substitution rate heterogeneity was corrected using an invariable and two gamma distributed substitution rate categories and LG substitution model for amino acid substitutions (LG+G+I). Diatom sequences are indicated in bold. The codes before the taxon name are the corresponding sequence IDs (For respective databases refer to materials and methods section).

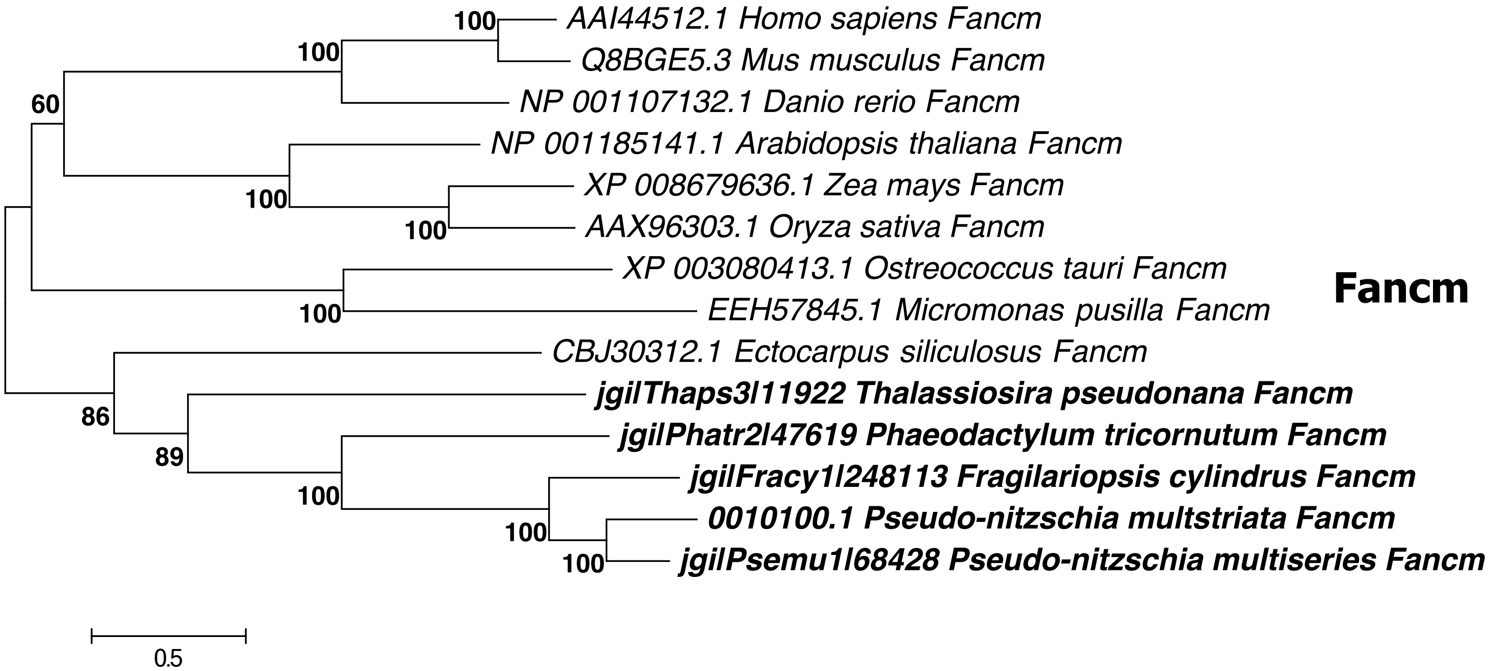

AAI44512.1 Homo sapiens Fancm
Q8BGE5.3 Mus musculus Fancm
NP 001107132.1 Danio rerio Fancm
NP 001185141.1 Arabidopsis thaliana Fancm
XP 008679636.1 Zea mays Fancm
AAX96303.1 Oryza sativa Fancm
XP 003080413.1 Ostreococcus tauri Fancm
EEH57845.1 Micromonas pusilla Fancm
CBJ30312.1 Ectocarpus siliculosus Fancm
jgi|Thaps3|11922 Thalassiosira pseudonana Fancm
jgi|Phatr2|47619 Phaeodactylum tricornutum Fancm
jgi|Fracy1|248113 Fragilariopsis cylindrus Fancm
0010100.1 Pseudo-nitzschia multstriata Fancm
jgi|Psemu1|68428 Pseudo-nitzschia multiseries Fancm
Fancm
100
100
60
100
100
100
86
89
100
100
100
0.5

Figure S14. Phylogenetic tree of Fancm proteins inferred from maximum likelihood analysis. Numbers on the branches indicate bootstrap support from 1000 replicates. Among-site substitution rate heterogeneity was corrected using an invariable and two gamma distributed substitution rate categories and WAG substitution model with frequencies for amino acid substitutions (WAG+G+I+F). Diatom sequences are indicated in bold. The codes before the taxon name are the corresponding sequence IDs (For respective databases refer to materials and methods section).

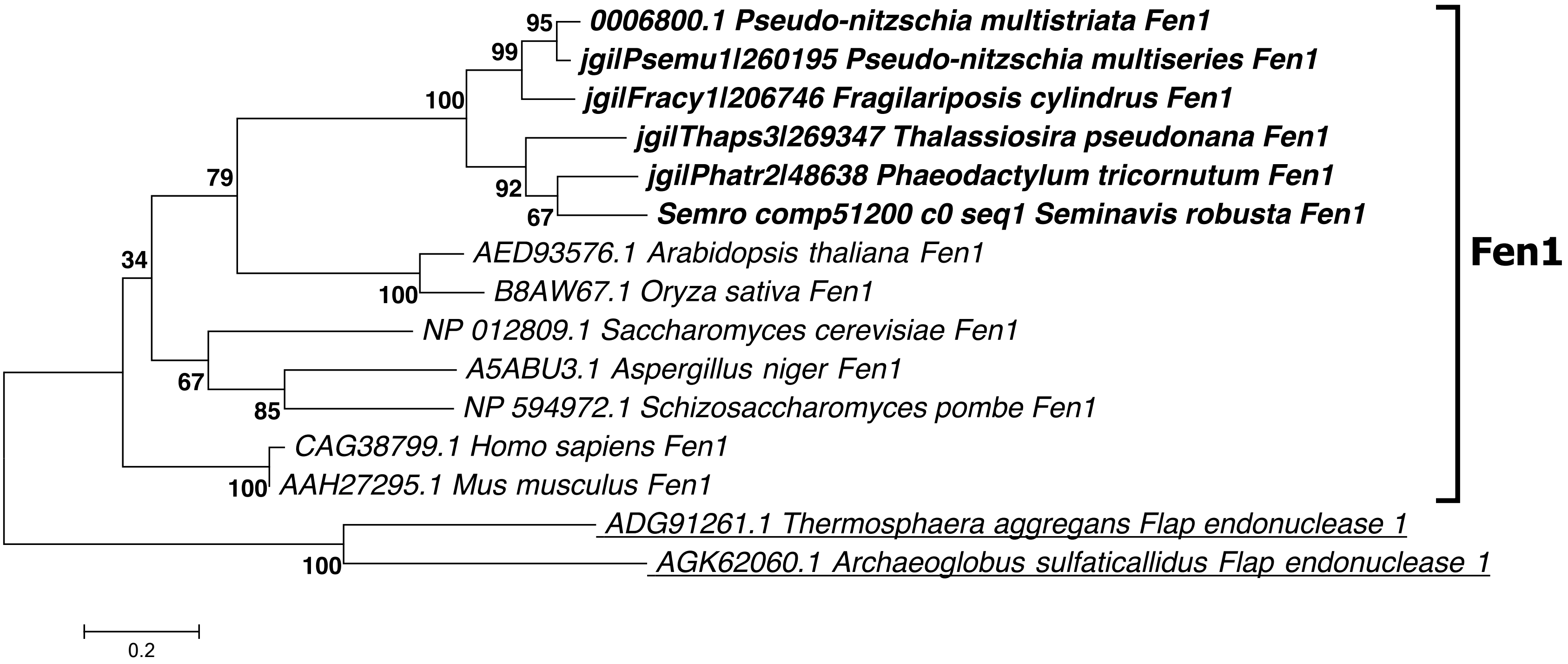

0006800.1 Pseudo-nitzschia multistriata Fen1
jgi|Psemu1|260195 Pseudo-nitzschia multiseries Fen1
jgi|Fracy1|206746 Fragilariposis cylindrus Fen1
jgi|Thaps3|269347 Thalassiosira pseudonana Fen1
jgi|Phatr2|48638 Phaeodactylum tricornutum Fen1
Semro comp51200 c0 seq1 Seminavis robusta Fen1
AED93576.1 Arabidopsis thaliana Fen1
B8AW67.1 Oryza sativa Fen1
NP 012809.1 Saccharomyces cerevisiae Fen1
A5ABU3.1 Aspergillus niger Fen1
NP 594972.1 Schizosaccharomyces pombe Fen1
CAG38799.1 Homo sapiens Fen1
AAH27295.1 Mus musculus Fen1
ADG91261.1 Thermosphaera aggregans Flap endonuclease 1
AGK62060.1 Archaeoglobus sulfaticallidus Flap endonuclease 1
Fen1
95
99
100
79
92
67
34
100
67
85
100
100
0.2

Figure S15. Phylogenetic tree of Fen1 proteins inferred from maximum likelihood analysis, rooted with archaeal Fen1 proteins as an outgroup (underlined). Numbers on the branches indicate bootstrap support from 1000 replicates. Among-site substitution rate heterogeneity was corrected using two gamma distributed substitution rate categories and LG substitution model for amino acid substitutions (LG+G). Diatom sequences are indicated in bold. The codes before the taxon name are the corresponding sequence IDs (For respective databases refer to materials and methods section).

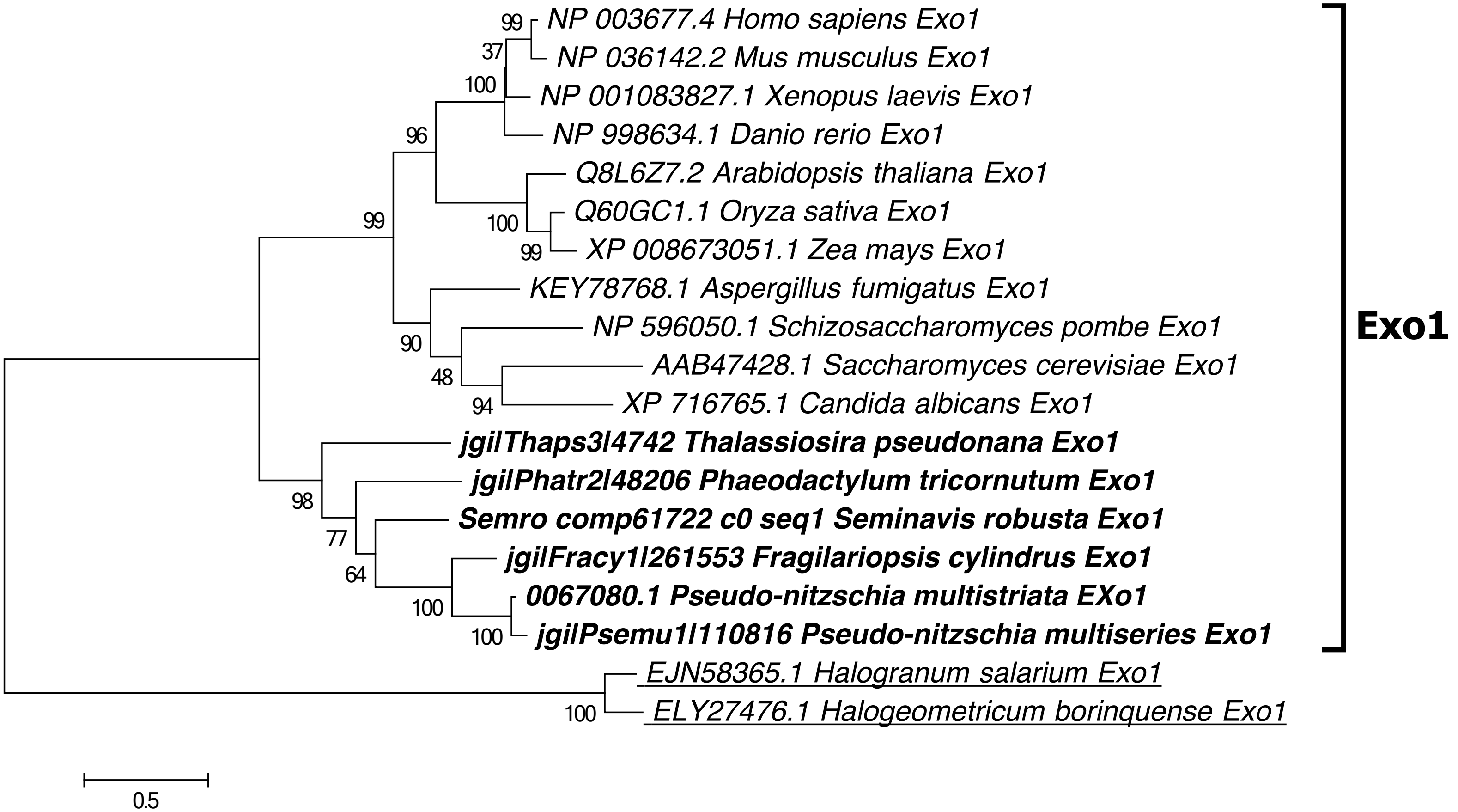

NP 003677.4 Homo sapiens Exo1
NP 036142.2 Mus musculus Exo1
NP 001083827.1 Xenopus laevis Exo1
NP 998634.1 Danio rerio Exo1
Q8L6Z7.2 Arabidopsis thaliana Exo1
Q60GC1.1 Oryza sativa Exo1
XP 008673051.1 Zea mays Exo1
KEY78768.1 Aspergillus fumigatus Exo1
NP 596050.1 Schizosaccharomyces pombe Exo1
AAB47428.1 Saccharomyces cerevisiae Exo1
XP 716765.1 Candida albicans Exo1
jgi|Thaps3|4742 Thalassiosira pseudonana Exo1
jgi|Phatr2|48206 Phaeodactylum tricornutum Exo1
Semro comp61722 c0 seq1 Seminavis robusta Exo1
jgi|Fracy1|261553 Fragilariopsis cylindrus Exo1
0067080.1 Pseudo-nitzschia multistriata EXo1
jgi|Psemu1|110816 Pseudo-nitzschia multiseries Exo1
EJN58365.1 Halogranum salarium Exo1
ELY27476.1 Halogeometricum borinquense Exo1
Exo1
99
37
100
96
99
100
99
90
48
94
98
77
64
100
100
100
0.5

Figure S16. Phylogenetic tree of Exo1 proteins inferred from maximum likelihood analysis, rooted with archaeal Exo1 proteins as an outgroup (underlined). Numbers on the branches indicate bootstrap support from 1000 replicates. Among-site substitution rate heterogeneity was corrected using an invariable and two gamma distributed substitution rate categories and LG substitution model for amino acid substitutions (LG+G+I). Diatom sequences are indicated in bold. The codes before the taxon name are the corresponding sequence IDs (For respective databases refer to materials and methods section).

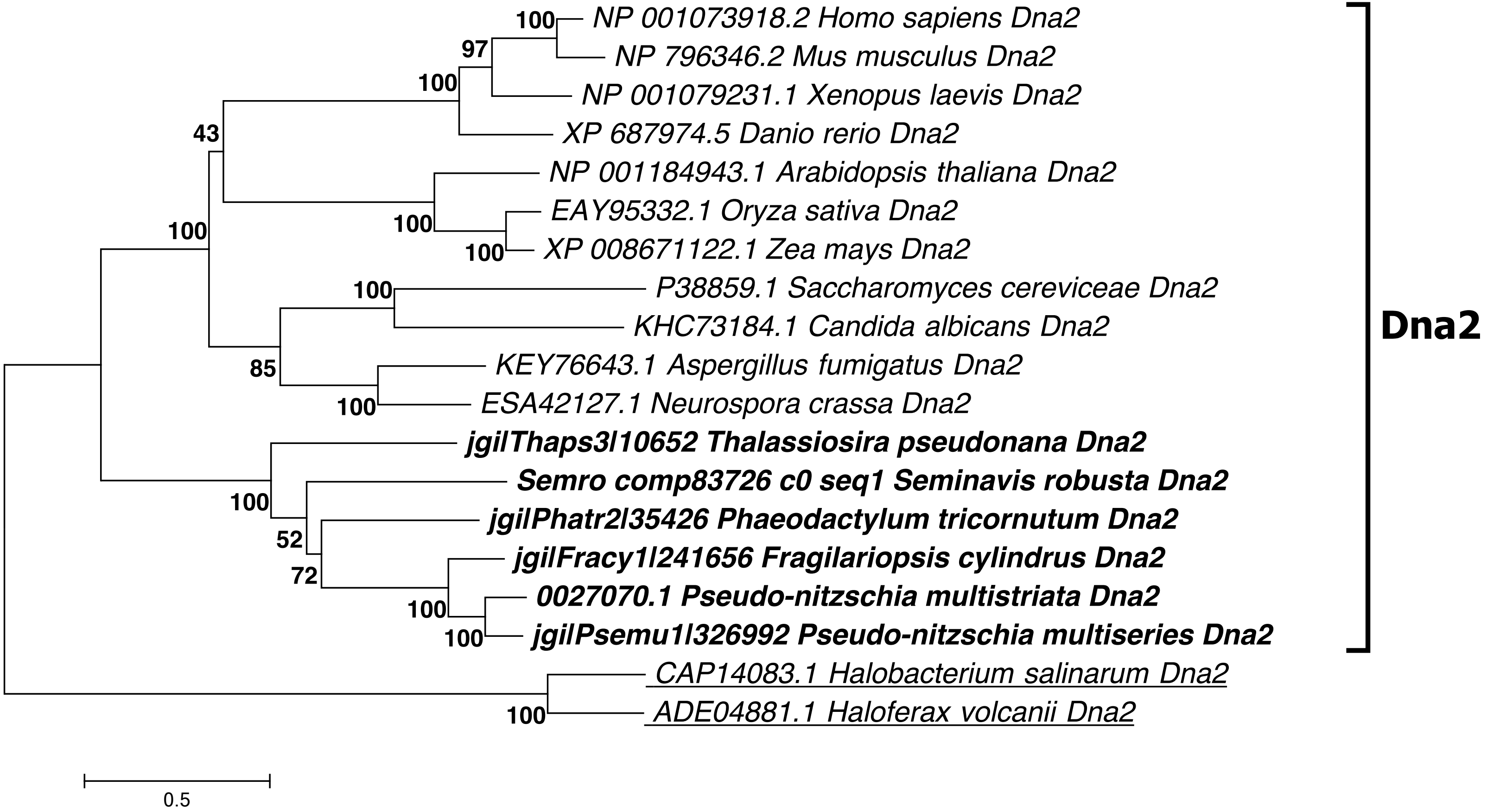

NP 001073918.2 Homo sapiens Dna2
NP 796346.2 Mus musculus Dna2
NP 001079231.1 Xenopus laevis Dna2
XP 687974.5 Danio rerio Dna2
NP 001184943.1 Arabidopsis thaliana Dna2
EAY95332.1 Oryza sativa Dna2
XP 008671122.1 Zea mays Dna2
P38859.1 Saccharomyces cereviceae Dna2
KHC73184.1 Candida albicans Dna2
KEY76643.1 Aspergillus fumigatus Dna2
ESA42127.1 Neurospora crassa Dna2
jgi|Thaps3|10652 Thalassiosira pseudonana Dna2
Semro comp83726 c0 seq1 Seminavis robusta Dna2
jgi|Phatr2|35426 Phaeodactylum tricornutum Dna2
jgi|Fracy1|241656 Fragilariopsis cylindrus Dna2
0027070.1 Pseudo-nitzschia multistriata Dna2
jgi|Psemu1|326992 Pseudo-nitzschia multiseries Dna2
CAP14083.1 Halobacterium salinarum Dna2
ADE04881.1 Haloferax volcanii Dna2
Dna2
100
97
100
43
100
100
100
100
85
100
100
52
72
100
100
100
0.5

Figure S17. Phylogenetic tree of Dna2 proteins inferred from maximum likelihood analysis, rooted with archaeal Dna2 proteins (underlined). Numbers on the branches indicate bootstrap support from 1000 replicates. Among-site substitution rate heterogeneity was corrected using an invariable and two gamma distributed substitution rate categories and LG substitution model for amino acid substitutions (LG+G+I). Diatom sequences are indicated in bold. The codes before the taxon name are the corresponding sequence IDs (For respective databases refer to materials and methods section).

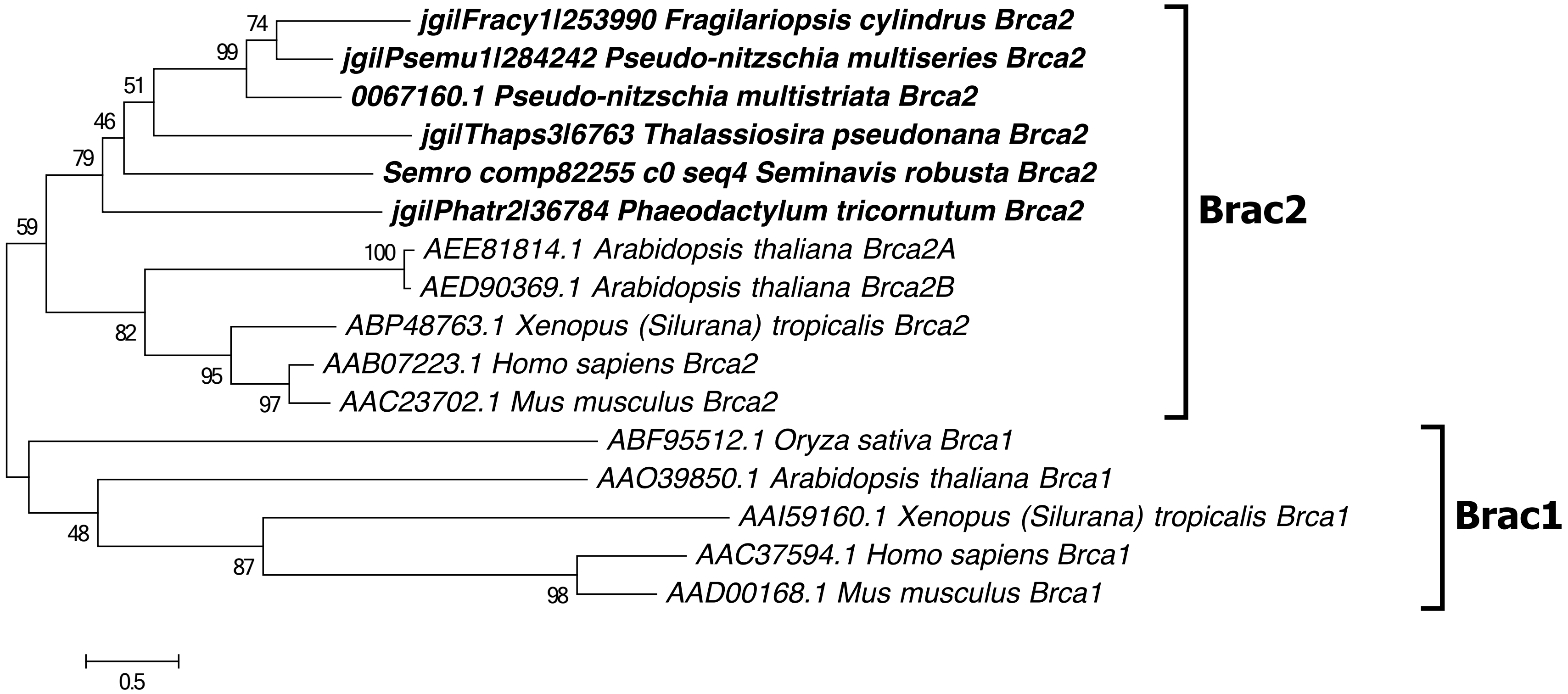
jgi|Fracy1|253990 Fragilariopsis cylindrus Brca2
jgi|Psemu1|284242 Pseudo-nitzschia multiseries Brca2
0067160.1 Pseudo-nitzschia multistriata Brca2
jgi|Thaps3|6763 Thalassiosira pseudonana Brca2
Semro comp82255 c0 seq4 Seminavis robusta Brca2
jgi|Phatr2|36784 Phaeodactylum tricornutum Brca2
AEE81814.1 Arabidopsis thaliana Brca2A
AED90369.1 Arabidopsis thaliana Brca2B
ABP48763.1 Xenopus (Silurana) tropicalis Brca2
AAB07223.1 Homo sapiens Brca2
AAC23702.1 Mus musculus Brca2
ABF95512.1 Oryza sativa Brca1
AAO39850.1 Arabidopsis thaliana Brca1
AAI59160.1 Xenopus (Silurana) tropicalis Brca1
AAC37594.1 Homo sapiens Brca1
AAD00168.1 Mus musculus Brca1
74
99
51
46
79
59
100
82
95
97
48
87
98
Brac2
Brac1
0.5

Figure S18. Phylogenetic tree of Brca proteins inferred from maximum likelihood analysis rooted. Numbers on the branches indicate bootstrap support from 1000 replicates. Among-site substitution rate heterogeneity was corrected using three gamma distributed substitution rate categories and WAG substitution model with frequencies for amino acid substitutions (WAG+G+F). Diatom sequences are indicated in bold. The codes before the taxon name are the corresponding sequence IDs (For respective databases refer to materials and methods section).
