# Supplementary material for: Identification of the meiotic toolkit in diatoms and exploration of meiosis-specific SPO11 and RAD51 homologs in the sexual species Pseudo-nitzschia multistriata and Seminavis robusta
Source: BMC Genomics. 2015 Nov 14;16:930. doi: 10.1186/s12864-015-1983-5 (PMC4647503; doi:10.1186/s12864-015-1983-5)
Supplement: Additional file 2: — Multiple sequence alignment of Rec8/Rad21proteins used in building the phylogenetic tree of Rad21, shown in Fig. 1 . (PDF 894 kb) [file 12864_2015_1983_MOESM2_ESM.pdf]

Additional file 2. Multiple sequence alignment of Rad21 and Rec8 proteins used in phylogenetic analysis of Rad21/Rec8 protein family (in figure 1).

```
                                               *        20         *        40         *        60         *        80         *       100
Arabidopsis_thaliana_Rec8_(SYN1)    : MFYSHQLLAR-KAPL----GQIWMAATL-----HAKINRKKLDKLDIIQICEEILN-------------P-SVPMALRLSGILMGGVVIVYERKVKLLFD :  76
Oryza_sativa_Rec8                   : GLLRSPLLSIASFRLLFSCAVLRMAATL-----HSKINRKRLDKLDIIKICEEILN-------------P-SVPMALRLSGILMGGVAIVYERKVKALYD :  81
Oryza_sativa_Rad21-4/Rec8           : MFYSHQLLAR-KAPL----GQIWMAATL-----HSKINRKRLDKLDIIKICEEILN-------------P-SVPMALRLSGILMGGVAIVYERKVKALYD :  76
Homo_sapiens_Rec8                   : MFYYPNVLQRHTGCF----ATIWLAATR-----GSRLVKREYLRVNVVKTCEEILNYVLVRVQPPQPGLP-RPRFSLYLSAQLQIGVIRVYSQQCQYLVE :  90
Mus_musculus_Rec8                   : MFYYPNVLQRHTGCF----ATIWLAATR-----GSRLVKREYLNVNVVKTCEEILNYVLVRVQPPVAGLP-RPRFSLYLSAQLQIGVIRVYFQQCQYLVE :  90
Schizosaccharomyces_pombe_Rec8      : MFYNQDVLTKEKGGM----GVIWLAATLGSKHSLRKLHKKDIMSVDIDEACDFVAF-------------S-PEPLALRLSSNLMIGVTRVWAHQYSFFHS :  82
Sordaria_macrospora_Rec8            : MFYSHEILTNQQYGV----ATVWLVSTFGLRSSNRKISRKAIQGVNVRKACETILQ-------------P-GAPIALRLQGSLLYGVSRVFSQQCSYVLT :  82
Neurospora_crassa_Rec8              : MFYSHEILTNQQYGV----ATVWLVSTFGLRSSNRKISRKAIQEVNVRKACETILQ-------------P-GAPIALRLQGSLLYGVSRVFSQQCSYVLT :  82
Arabidopsis_thaliana_Rad21          : MFYSHCLVSR-KGPL----GAIWVAAYF-----FKKLKKSQVKATHIPSSVDQILQ-------------KELDALTYRVLAYLLLGVVRIYSKKVDFLFD :  77
Oryza_sativa_Rad21                  : MFYSQFILAK-KGPL----GTIWIAAHL-----ERKLRKNQVADTDIGVSVDSIIF-------------P-EVPIALRLSSHLMLGVVRIYSRKVNYLFH :  76
Ricinus_communis_Rad21              : MFYSQTFLAR-KGPL----GTVWCAAHL-----QHRLKKSHYTSTDISSTVDRIMF-------------P-EVPIALRMSGHLLLGVVRIYSKKVDFLYH :  76
Theobroma_cacao_Rad21               : MFYSQTFLAR-KGPL----GTVWCAAHL-----QHRLKKSHYTAADIPSTVDLIMF-------------P-EVPIALRMSGHLLLGVVRIYSKKVDYLFH :  76
Zea_mays_Rad21                      : MFYSQFILAK-KGPL----GTIWIAAHL-----ERKLRKNQVADTDIGVSVDSIIF-------------P-DVPIALRLSSHLMLGVVRIYSRKVNYLFH :  76
Arabidopsis_thaliana_Rad21-3        : MFYSHTLLAR-KGPL----GTVWCAAHV-----HQRLKKSQYTSINIPDTVDNIMF-------------P-EVPLALRTSSHLLVGVVRIYSKKVDYLYN :  76
Arabidopsis_thaliana_Rad21-4        : MFYSQFILAK-KGPL----GTIWIAAHL-----ERKLRKNQVADTDIGVSVDSILF-------------P-EAPIALRLSSHLLLGVVRIYSRKVNYLFD :  76
Oryza_sativa_Rad21-2                : MSCSKVLLSK-KGVL----GTVWVAAVSG----VAALSRDQVVRTNVVACVDKILP-------------DDNDKTTYRVLGLLLLGIVRIYSKKVEYLCH :  78
Oryza_sativa_Rad21-3                : MFYSHTILAR-KSPL----GTVWIAAHL-----ERKIKKPQIDGIDIPSYAEFIMF-------------P-EVPIALRLSGHLLLGLVRIYSWKVNYLFH :  76
Homo_sapiens_Rad21                  : MFYAHFVLSK-RGPL----AKIWLAAHW-----DKKLTKAHVFECNLESSVESIIS-------------P-KVKMALRTSGHLLLGVVRIYHRKAKYLLA :  76
Mus_musculus_Rad21                  : MFYAHFVLSK-RGPL----AKIWLAAHW-----DKKLTKAHVFECNLESSVESIIS-------------P-KVKMALRTSGHLLLGVVRIYHRKAKYLLA :  76
Danio_rerio_Rad21                   : MFYAHFVLSK-RGPL----AKIWLAAHW-----DKKLTKAHVFECNLESSVESIIS-------------P-KVKMALRTSGHLLLGVVRIYHRKAKYLLA :  76
Xenopus_(Silurana)_tropicalis_Rad21 : MFYAHFVLSK-RGPL----AKIWLAAHW-----DKKLTKAHVFECNLESSVESIIS-------------P-KVKMALRTSGHLLLGVVRIYHRKAKYLLA :  76
Drosophila_melanogaster_Rad21       : MFYEHIILAK-KGPL----ARIWLAAHW-----DKKITKAHVFETNIEKSVEGILQ-------------P-KVKLALRTSGHLLLGVVRIYSRKAKYLLA :  76
Caenorhabditis_elegans_Rad21        : MFYAQFVLAK-KGPL----AKVWLAAHW-----EKKLTKAQIFETDVPQAIEEVIR-------------P-KVKMALRTVGHLLLGIVRIYSKKTRYLLA :  76
Schizosaccharomyces_pombe_Rad21     : MFYSEAILSK-KGPL----AKVWLAAHW-----EKKLSKVQTLHTSIEQSVHAIVT-------------EETAPMALRLSGQLMLGVVRIYSRKARYLLE :  77
Sordaria_macrospora_Rad21           : MFWSGTLLSA-TGPL----AKAWLSANQ-----ERKVSKVQILQHNLQDSVDAIIA-------------PNDAPLALRLSGQLLLGVVRIYSRKARYLLD :  77
Neurospora_crassa_Rad21             : MFWSGTLLSA-TGPL----AKAWLSANQ-----ERKVSKVQILQHNLQDSVDAIIA-------------PNDAPLALRLSGQLLLGVVRIYSRKARYLLD :  77
Aspergillus_niger_Rad21             : MFYSETLLSK-TGPL----ARVWLSANL-----ERKLSKSHILQSDIESSVSAIVD-------------QGQAPMALRLSGQLLLGVVRIYSRKARYLLD :  77
Thalassiosira_pseudonana_Rad21      : MFYSQIILAK-KGPL----GKVWLAAHWG----DKKITRPQIFSTDIATSVDSIVN-------------P-TVPLALRVSGHLLLGVVRIYSRKVRYLMH :  77
Phaeodactylum_tricornutum_Rad21     : MFYSQIILAK-KGPL----GKVWMAAHWG----DKKLGRPQIFATDISASVDSIVH-------------P-QVPLALRVSGHLLLGVVRIYSRKVKYLMH :  77
Fragilariopsis_cylindrus_Rad21      : MFYSQVILAK-KGPL----AKVWLAAHWG----DKKLARPQIFATDISQSCADIMN-------------P-SVPLALRLSGHLLLGVVRIYSRKVKYVLN :  77
Pseudo-nitzschia_multiseries_Rad21  : MEFG-------SSPL----GCLW--KDW---------------------ACCART------------------------------------TRRASSLLD :  30
Pseudo-nitzschia_multistriata_Rad21 : MFYSQVILAK-KGPL----AKVWLAAHWG----DKKLARPQIFATDISQSCTDIMN-------------P-SVPLALRLSGHLLLGVVRIYSRKVKYVLN :  77
Seminavis_robusta_Rad21             : MFYSQIILAK-KGPL----GKVWQAAHWG----DKKMNRVQIFGTDIAASVESIVH-------------P-QVPLALRVSGHLLLGVVRIYSRQVKYCLT :  77
                                      mf          gp        w aa                                           p     alr s  l  g  r
```

```
                                               *       120         *       140         *       160         *       180         *       200
Arabidopsis_thaliana_Rec8_(SYN1)    : -DVNRFL-----------------------VEINGAWRTKS----------------------------------------------------------- :  93
Oryza_sativa_Rec8                   : SNCVRSS----------------------SQMILRSTRHGG----------------------------------SSQSQTPPYFPRAKPKPSIVSERYM : 125
Oryza_sativa_Rad21-4/Rec8           : -DVSRFL-----------------------IEINEAWRVKP----------------------------------------------------------- :  93
Homo_sapiens_Rec8                   : -DIQHIL-----------------------ERLHRAQLQIR----------IDM---------------------ETE-----LPSLLLPNHLAMMETLE : 130
Mus_musculus_Rec8                   : -DIQHIL-----------------------EHLHRAQLRIR----------IDM-------------------------EEADLPSLLLPNCLAMMETLE : 131
Schizosaccharomyces_pombe_Rec8      : -QVSTLH-----------------------LRVRKELDHFT---------------------------------------SKPFKNIDIQNEQTNPKQLL : 119
Sordaria_macrospora_Rec8            : -DAEKIH-----------------------MHMRCFYNVLG-------GSGNAL---------------------DPQAGKAKRNQLILPDDPD---FEV : 127
Neurospora_crassa_Rec8              : -DAEKIH-----------------------MHMRCFYNVLG-------GTENAL---------------------DPQAGKAKRNQLILPDDPN---FEV : 127
Arabidopsis_thaliana_Rad21          : -DCNKAL-----------------------IGVKEFVAKER----NREKTGVSL------------------------PASIECFSIALPERFELDAFDL : 125
Oryza_sativa_Rad21                  : -VCSEAL-----------------------LKIKQAFRSTA----------VDL---------------------PPEESTAPYHSITLPETFHLDDF-- : 119
Ricinus_communis_Rad21              : -DCNVVL-----------------------VGLRKAFTSIE----------VNL---------------------PENATTAKFESVTLPPTFDLDALDV : 121
Theobroma_cacao_Rad21               : -DCNIVL-----------------------IGLRKVFASVQ----------VDL---------------------PEDARQAPFQAITLPQTFNLDAMEL : 121
Zea_mays_Rad21                      : -DCSEAL-----------------------LKIKQAFRSTA----------VDL---------------------PPEESTAPYHSITLPETFDLDDF-- : 119
Arabidopsis_thaliana_Rad21-3        : -DWNLLN-----------------------TWVAKAFVSTQ----------VNL---------------------PEDARQAPPESVTLPQALNLDEFDL : 121
Arabidopsis_thaliana_Rad21-4        : -DCSEAL-----------------------LKVKQAFRSAA----------VDL---------------------PPEESTAPYHSITLPETFDLDDFEL : 121
Oryza_sativa_Rad21-2                : -ECNELLGSYGSAHCNELSIPTGGATNRVSKQAKKPVRARRLVVRQEGAYKVKIPMQAVRTTRAETRATSQITEVRDTHATPDLPTFTIPKRFELDSFDL : 177
Oryza_sativa_Rad21-3                : -DCNRML-----------------------STIRTAFASVQ----------VDL---------------------PLHADHAPFDTITLPDTFRLDDINL : 121
Homo_sapiens_Rad21                  : -DCNEAF-----------------------IKIKMAFRPGV----------VDL---------------------PEENREAAYNAITLPEEFHDF---- : 117
Mus_musculus_Rad21                  : -DCNEAF-----------------------IKIKMAFRPGV----------VDL---------------------PEENREAAYNAITLPEEFHDF---- : 117
Danio_rerio_Rad21                   : -DCNEAF-----------------------IKIKMAFRPGV----------VDL---------------------PEDNREAAYNAITLPEEFHDF---- : 117
Xenopus_(Silurana)_tropicalis_Rad21 : -DCNEAF-----------------------IKIKMAFRPGV----------VDL---------------------PEENREAAYNAITLPEEFHDF---- : 117
Drosophila_melanogaster_Rad21       : -DCNEAF-----------------------VKIKMAFRPGM----------VDL---------------------PEGHREANVNAITLPEVFHDF---- : 117
Caenorhabditis_elegans_Rad21        : -DTNEAY-----------------------QKMKINFRNGF------------------------------------SFEVDIPENAEIEEDFSNFIDKY : 116
Schizosaccharomyces_pombe_Rad21     : -DCTEAL-----------------------MRLKMSFQPGQVDMIEPATALQSL---------------------KGKDAVTQSANLTLPETITEFD--L : 130
Sordaria_macrospora_Rad21           : -DCNEAL-----------------------MKIKMAFRSTG---------NHDI---------------------PTNLHVQNSESLMLPDQITPYDNLD : 123
Neurospora_crassa_Rad21             : -DCNEAL-----------------------MKIKMAFRSTG---------NHDI---------------------PTNLHVQNSESLMLPDQITPYDNLD : 123
Aspergillus_niger_Rad21             : -DCNEAL-----------------------MKIKMAFRLTN---------NNDL-----------------------TTTVVAPGGITLPDVLTESDLFM : 121
Thalassiosira_pseudonana_Rad21      : -DCHEAM-----------------------VKIKMAFRPEGGVNSMGGKGGEGMVVVDMDPNQIKGKRGGEGGDGSGNNVDPRFGEFYTQDMAGPLGGML : 153
Phaeodactylum_tricornutum_Rad21     : -DCHEAM-----------------------VKIKMAFRPSQ----------------------------------------EKNAAIDLPSNLKESSNMN : 113
Fragilariopsis_cylindrus_Rad21      : -DCTEAM-----------------------LKLQMAFTTTK------------------------------------QVGDEKGSSRELLSEKDAPMHQL : 117
Pseudo-nitzschia_multiseries_Rad21  : -KSN------------------------------------------------------------------------------------------------ :  33
Pseudo-nitzschia_multistriata_Rad21 : -DCTEAM-----------------------LKLQMAFCNQA------GGGKLGL---------------------RLDEHGKPVRGRELLSEKDAPTHQL : 126
Seminavis_robusta_Rad21             : -DCTEAM-----------------------VKIKMAFRNDAKNDGKSNSNNNS----------------------NTNTNSIDLVVIGDPTKPGGATTVN : 131
```

```
                                               *       220         *       240         *       260         *       280         *       300
Arabidopsis_thaliana_Rec8_(SYN1)    : -VPDPTL-------------------------------LPKGKTHARK--EAVTLPE------------------------------------------- : 116
Oryza_sativa_Rec8                   : LYGDEHLT-----------------------FSWVSVTGAKGAGLRAVWYEAVTLPE-------------------------------------NIM--- : 162
Oryza_sativa_Rad21-4/Rec8           : -VADPTV-------------------------------LPKGKTQAKY--EAVTLPE-------------------------------------NIM--- : 119
Homo_sapiens_Rec8                   : DAPDPFFG----M-----MSVDPR-------LPSP-FDIP---QIRHL--LEAAIPE------------------------------------------- : 165
Mus_musculus_Rec8                   : DAPEPFFGK---------MSVDPR-------LPSP-FDIP---QIRHL--LEAATPE------------------------------------------- : 166
Schizosaccharomyces_pombe_Rec8      : LAEDPAF--------------------------IP--------EVSLY--DAFNLPS---------------------------------------V--- : 141
Sordaria_macrospora_Rec8            : NMGLPAFH----------FDEEGNLATQ---FQSQ-ASRKTSSQFSPL--DRFNLTP--------------------------------DGNGSFII--- : 176
Neurospora_crassa_Rec8              : NMGLPAFR----------LDEDGNLASL---FQSQ-ASCKTSSQFSPL--DKFNLTP--------------------------------DGSGSFLL--- : 176
Arabidopsis_thaliana_Rad21          : GVLEDFHGGNVKPHEDITLKDGSQETERMDMYSMERFDMEEDLLFTFH--ETFSTNHNENKHES--------------FAHDMELDAENVRDTTEEA--- : 206
Oryza_sativa_Rad21                  : ELPEAAFQGDIDHHVSTKEQITLQDNPEKTGYSTSQFGLDERFGDGNSSHIGLDLEEELMLNKD--------------QSIQLEADDGIIIQGRSAVHPT : 205
Ricinus_communis_Rad21              : DFDIDAYG-------------------------------SPDNHMRSQ--EEITLQD------------------------------QIPTDRDPYV--- : 155
Theobroma_cacao_Rad21               : DGDMC-------------------------------TEGSSDNHLSNQ--EDITLTD------------------------------QIPIGGDPYV--- : 155
Zea_mays_Rad21                      : ELPEAAFQGDTDHHVSTKEQITLQDNPEKTGYSTSQFGLDERFGDASSSHIGFDLEEELILNKD--------------HSIHLQSDDGIIIQGRSSVPST : 205
Arabidopsis_thaliana_Rad21-3        : EDDTLDME----------------------------------FDNHTRSEEDITLTD------------------------------QIPTGIDPYV--- : 154
Arabidopsis_thaliana_Rad21-4        : PDNEIFQGNYVDHHVSTKEQITLQDTMDGVVYSTSQFGLDERFGDGDTSQAALDLDEAVFQDKDVIGSDDEGVPGIDHNAYLDAAAPGIKDSMEGVSEAM : 221
Oryza_sativa_Rad21-2                : GIPEDRDD----------DDVDHHQLP----HQGTMLEDENHHTSCLF--ESYKMMTCSYADLD--------------SACIMPVRVTIPTEMMSVI--- : 244
Oryza_sativa_Rad21-3                : DDAIRLID-------------------------------TPDNHRKSL--DQITLAE------------------------------------GDYL--- : 149
Homo_sapiens_Rad21                  : DQPLPDLDD---------------------------IDVAQQFSLNQSRVEEITMRE---------------------------------EVGNISI--- : 154
Mus_musculus_Rad21                  : DQPLPDLDD---------------------------IDVAQQFSLNQSRVEEITMRE---------------------------------EVGNISI--- : 154
Danio_rerio_Rad21                   : DQPLPDLDD---------------------------IDVAQQFTLNQSRVEEITMRE---------------------------------EVGNISL--- : 154
Xenopus_(Silurana)_tropicalis_Rad21 : DQPLPDLDD---------------------------IDVAQQFSLNQSRVEEITMRE---------------------------------EVSNINI--- : 154
Drosophila_melanogaster_Rad21       : DTALPELN----D-----IDMEAQ-------FSI---------NQSRA--DEITMRE-----------------------------------DYGSL--- : 152
Caenorhabditis_elegans_Rad21        : NITVPEF------------------------HDADYNEQLIMANVSRR--EDITMKE--------------------------------TVNFNVEF--- : 155
Schizosaccharomyces_pombe_Rad21     : LVPDSTFD----------FQWSQL-------LRTP-SRSSNTLELHSL--PISSSPS----------------------------------FPSSQL--- : 173
Sordaria_macrospora_Rad21           : LLPPPSADY----------------------LASQIEEVTGAPIMARK--VQVRASQ-----------------------------------RDINL--- : 161
Neurospora_crassa_Rad21             : LLPPPSADY----------------------LASQIEEVTGAPIMARK--VQVRASQ-----------------------------------RDINL--- : 161
Aspergillus_niger_Rad21             : NLDSSLL-------------------------------LPQTLNLEP---EGKRPGS------------------------------------------- : 144
Thalassiosira_pseudonana_Rad21      : VEPVLLMDPND-D-----LNDVGGGALGGGAFAIP-FSLDPAGNTGEG--ENWIVAD------------------------EDGDDASTTAGRGRNR--- : 217
Phaeodactylum_tricornutum_Rad21     : VANFGEYHEVVFE-----ITDAPTG------FQLP-FEINDDAAA-----EDWVPAD----------------------------------LDETHE--- : 159
Fragilariopsis_cylindrus_Rad21      : VSNFGEYDQ---------VHIVEG-------FCLP---------LPED--NEWILAE-----------------------------------DDDED--- : 152
Pseudo-nitzschia_multiseries_Rad21  : ---------------------------------------------------------------------------------------------------- :   -
Pseudo-nitzschia_multistriata_Rad21 : VSNFGEYDQ---------IHVVEG-------FCLPLPEDN-----------EWILAE------------------------------DEAGIGGGSV--- : 166
Seminavis_robusta_Rad21             : NSNFGEYQDFLLDPNAIVVDDDGDATG----FAVP-VDLNEMTEQELA--ERWVLAEQQHQQQQ--------------QSQGNTQERMNHESQSTTT--- : 207
```

```
                                               *       320         *       340         *       360         *       380         *       400
Arabidopsis_thaliana_Rec8_(SYN1)    : ---NEEADFGDFE----------------------------------------------------------------------------------QTRNV : 131
Oryza_sativa_Rec8                   : DMDVEQPMLFSEA----------------------------------------------------------------------------------DTTRF : 180
Oryza_sativa_Rad21-4/Rec8           : DMDVEQPMLFSEA--------------------------------------------------------------------------------------D : 133
Homo_sapiens_Rec8                   : ---RVEEIPPEVP----------------------------------------------------------------------------------TEPRE : 180
Mus_musculus_Rec8                   : --KTRKETLPEAT----------------------------------------------------------------------------------PDPRK : 182
Schizosaccharomyces_pombe_Rec8      : DLHVDMSSFTQPK-------------------------------------------------------------------------------------EN : 156
Sordaria_macrospora_Rec8            : GLDLPQSSPTNPL-------------------------------------------------------------------------------------YS : 191
Neurospora_crassa_Rec8              : GLDLPPSSPADPL-------------------------------------------------------------------------------------YS : 191
Arabidopsis_thaliana_Rad21          : SVRVVEAEPLDSN----------------------------------------------------------------------------------EPSRD : 224
Oryza_sativa_Rad21                  : DMEVDDNQNKDES---AEGYNMEDGPSSHNKLNPLSADGLVGNSLPNWHTYNVQTPDLNDMLLNNDGNAGPSASYYQPSPFPCDEPASPEFVSA-QAPAT : 301
Ricinus_communis_Rad21              : VISFDEDVMMDTL------------------------------------------------------------------------------------PPE : 171
Theobroma_cacao_Rad21               : AITFDEDIMRDAS--------------------------------------------------------------------------------------L : 169
Zea_mays_Rad21                      : DMDIDDNKSQDIIAEGYHNMDGGHSSHGKLGSLNLNAIDLGGNSIPNWTGYNVHTPDLNDILLHNEGIAGPSASYIQPSPYTCDEPASPEFISA-QAPAT : 304
Arabidopsis_thaliana_Rad21-3        : AVTFDEDIISESI------------------------------------------------------------------------------------PMD : 170
Arabidopsis_thaliana_Rad21-4        : PMDFNEEQVEDLA--------------------------------------------MNNEFIEDAQAPQTPGLVEVPNSSSVREQMACDDHMDVEDLNA : 277
Oryza_sativa_Rad21-2                : SEVNSLLCLSSIG---------------------------------------------------------------------------------GEPENH : 263
Oryza_sativa_Rad21-3                : MIDLDEDARVELS--------------------------------------------------------------------------------------A : 163
Homo_sapiens_Rad21                  : ---LQENDFGDFG------------------------------------------------------------------------------------MDD : 167
Mus_musculus_Rad21                  : ---LQENDFGDFG------------------------------------------------------------------------------------MDD : 167
Danio_rerio_Rad21                   : ---MTDNDFGDFG------------------------------------------------------------------------------------MDD : 167
Xenopus_(Silurana)_tropicalis_Rad21 : ---LQDNDFGDFG------------------------------------------------------------------------------------MDD : 167
Drosophila_melanogaster_Rad21       : SLSLQDDGFGDIG----------------------------------------------------------------------------------FEAET : 170
Caenorhabditis_elegans_Rad21        : NIDADFDGFGDEG----------------------------------------------------------------------------------ESWQL : 173
Schizosaccharomyces_pombe_Rad21     : SIEAGRNAQVESG------------------------------------------------------------------------------------FSL : 189
Sordaria_macrospora_Rad21           : QEDFNNSQFLNNT-------------------------------------------------------------------------------------MV : 176
Neurospora_crassa_Rad21             : QEDFNNSQFLNNT-------------------------------------------------------------------------------------MI : 176
Aspergillus_niger_Rad21             : SMDFGSQLLPDTG------------------------------------------------------------------------------------LRR : 160
Thalassiosira_pseudonana_Rad21      : GRDKKSTLSSNAA----------------------------------------------------------------------------------ANLTL : 235
Phaeodactylum_tricornutum_Rad21     : IVTGLQAELGEYG-----------------------------------------------------------------------------------SPRD : 176
Fragilariopsis_cylindrus_Rad21      : EVPQEQHLLVAAA------------------------------------------------------------------------------------VGQ : 168
Pseudo-nitzschia_multiseries_Rad21  : -LTTDDTDPTNAN--------------------------------------------------------------------------------------- :  45
Pseudo-nitzschia_multistriata_Rad21 : GEASQELLLAAHL------------------------------------------------------------------------------------PRK : 182
Seminavis_robusta_Rad21             : QQQTEEDSPNEWS--------------------------------------------------------------------------------------F : 221
```

```
                                             *       420         *       440         *       460         *       480         *       500
Arabidopsis_thaliana_Rec8_(SYN1)    : PKFGNYMDFQQTFISMRL-----------DESHVNNNPEP------------------------------------------------------------ :  160
Oryza_sativa_Rec8                   : RGMIVSIPLKSVMHVQRLEDL--------DDQYINVNLDD------------------------------------------------------------ :  212
Oryza_sativa_Rad21-4/Rec8           : TTRFRGMRLEDL-----------------DDQYINVNLDD------------------------------------------------------------ :  156
Homo_sapiens_Rec8                   : PERIPVTVLPPE-----------------AITILEAEPIR------------------------------------------------------------ :  203
Mus_musculus_Rec8                   : PDRTLATVQSPE-----------------VITLQEAEPIR------------------------------------------------------------ :  205
Schizosaccharomyces_pombe_Rec8      : PN---------------------------ISVLETLPDST------------------------------------------------------------ :  169
Sordaria_macrospora_Rec8            : QHSSYSLGPLSQHKPD-------------DDMMQFGEPEA------------------------------------------------------------ :  218
Neurospora_crassa_Rec8              : QHSSYSLGPLSQHKPD-------------DDMMEFGQPEA------------------------------------------------------------ :  218
Arabidopsis_thaliana_Rad21          : HQNASRHREDPESDDILLEPQMS------EDIRIAQEEDT---------------------------------------------------VRETICTIV :  267
Oryza_sativa_Rad21                  : PGLMEETVPSRVHESPVLSPQRKASPSSNDETAKTAENANDLVGAETTELALTKPVQIESSGAVQEIDSLSQQCATEELPPQAETSNLEATMDKSMINTD :  401
Ricinus_communis_Rad21              : EELNAGIRPTEDIVPPSGVDTDMASPHTIPSSRIEVTSET------------------------------------------------------------ :  211
Theobroma_cacao_Rad21               : PEVFQKSGSIQIN----------------DKLHGPPEDEH------------------------------------------------------------ :  193
Zea_mays_Rad21                      : PGLMEETVPSRVHESPVLSPQRKPSPSTNDETAKTDTPAA---------------------------PASDFLHSATGNANDVGAERTELGLAKHVQVES :  377
Arabidopsis_thaliana_Rad21-3        : VDQSTEPVSRHTGEID-------------VETAHETGPDN------------------------------------------------------------ :  197
Arabidopsis_thaliana_Rad21-4        : EEGIKSSGELNANEMPKRGEDLSSEYNAPESAVTPVEVDK-----------------------------------------------------SQIDENV :  324
Oryza_sativa_Rad21-2                : NAESACFTPVKDILPPEM-----------VDMMAEVNDPS------------------------------------------------------------ :  292
Oryza_sativa_Rad21-3                : PGQSSHMGPEPTDEERL------------PPFHIDFGPSD------------------------------------------------------------ :  191
Homo_sapiens_Rad21                  : REIMREGSAFED-----------------DDMLVSTTTSN------------------------------------------------------------ :  190
Mus_musculus_Rad21                  : REIMREGSAFED-----------------DDMLVSTSASN------------------------------------------------------------ :  190
Danio_rerio_Rad21                   : REMMREENAFGDIIHESSA----------ANLLLEAEPGP------------------------------------------------------------ :  197
Xenopus_(Silurana)_tropicalis_Rad21 : REMMREGSAFE------------------DDMLTTSAS-------------------------------------------------------------- :  187
Drosophila_melanogaster_Rad21       : PEIIRCSIPSNINDKIFD-----------NDVLENIESLD------------------------------------------------------------ :  199
Caenorhabditis_elegans_Rad21        : DHLYGSVEPLSLRPTPQ------------PESLMEVERDR------------------------------------------------------------ :  201
Schizosaccharomyces_pombe_Rad21     : GESFAHVGNDMQFHLP-------------ISNSGAATPRS------------------------------------------------------------ :  216
Sordaria_macrospora_Rad21           : DEEELALANNDDLGLE-------------LDFGMDIDDRP------------------------------------------------------------ :  203
Neurospora_crassa_Rad21             : DEEELALANNDDLGLE-------------LDFGMDIDERP------------------------------------------------------------ :  203
Aspergillus_niger_Rad21             : SASQEPARLEDHTLVD-------------LDLGEDETPLG------------------------------------------------------------ :  187
Thalassiosira_pseudonana_Rad21      : DSDMEGMGNQNPMEEEEE-----------EEGWGAFDPDA------------------------------------------------------------ :  264
Phaeodactylum_tricornutum_Rad21     : AAAAAVNNTLNDSELTDMLNRGQ------DEAWTAWNVDE------------------------------------------------------------ :  210
Fragilariopsis_cylindrus_Rad21      : GQQQQGGQPDHVMASPE------------EETWAAFDPDE------------------------------------------------------------ :  196
Pseudo-nitzschia_multiseries_Rad21  : ---------------------------------ATANNDA------------------------------------------------------------ :   52
Pseudo-nitzschia_multistriata_Rad21 : PTDGSGSGSPPAPVEE-------------EEAWAPFDPDN------------------------------------------------------------ :  209
Seminavis_robusta_Rad21             : GAFGGTAPTQSSQQQP-------------EEQWQEFDPEA-----------------------------------------------EDLGLPKDDDDND :  261


                                             *       520         *       540         *       560         *       580         *       600
Arabidopsis_thaliana_Rec8_(SYN1)    : ------EDLG----------QQFHQA---------DAENIT--LFEYHGSFQTNNETYDRFERFDI--------------------EGDDETQMNS---- :  209
Oryza_sativa_Rec8                   : ------DDF-----------SRAENH---------HQENIT--LADNFGSGLGETDVFNRFERFD---------------------ITDDDATFNV---- :  259
Oryza_sativa_Rad21-4/Rec8           : ------DDFS----------RAENHH---------QADAENITLADNFGSGLGETDVFNRFERFD---------------------ITDDDATFNV---- :  206
Homo_sapiens_Rec8                   : --------------------MLEIEG---------ERELPE--VSRRELDLLIA----------------------------------------EE---- :  228
Mus_musculus_Rec8                   : --------------------MLQIEG---------EQDLPEISRGDLELLIAEKDDAILLEERQRGRLLRQRRASL----------PLDESREEPR---- :  262
Schizosaccharomyces_pombe_Rec8      : --------------------SYLINT---------SQNYSL--RNNVSSFVYEDSRAFSTEEPL--------------------------DFEFDE---- :  208
Sordaria_macrospora_Rec8            : ----NAEIFG----------DWGIEI---------DADGNV--MATVDEPELPILPRPEEERAYATASGQV---------------HDEPEFHFDD---- :  274
Neurospora_crassa_Rec8              : ----NAEIFG----------DWGIEI---------DADGNV--MATVDEPELPVLPRPEEERTRVTAFGQG---------------HNESELSFDD---- :  274
Arabidopsis_thaliana_Rad21          : QRLVDSHE------------SSGDNL---------HRDGHTENLESEKTSKKTSCEEMQHDRSLPSECGIPEAIHGIEDQPSGAT-RINGEKEIPE---- :  341
Oryza_sativa_Rad21                  : GVAASGEATAAKETTEVSLVENSLEL---------CADGPTEPVIENQTQFNDGSVDVQGGGQHAPHAILATADSQV---------NVEASTQEMA---- :  479
Ricinus_communis_Rad21              : --------------------VDLQDS---------GPTNLTEVLMDFADLQETCPSNKTELQTQTLDFQEPGPSNQTEVLN-----SAINNDNFPPEIEV :  277
Theobroma_cacao_Rad21               : --------------------VRSQDP---------GQHNQI--EVPSASAAFQDPGRSNKSQVLESM-------------------SNDSSQDFPEIETI :  243
Zea_mays_Rad21                      : SAFWDADASGQQHTSEGLPSQGQADHLEPAADKLVNSDGIAASVETVTVNATIKDVPLAVNDSEPCVDGSTEPSVVENLVQINGPLIDAQDFQHEAQAMQ :  477
Arabidopsis_thaliana_Rad21-3        : -------------------EPRDSNI---------AFDTGTYSPRNVTEEFTEVQDPRQSNLTEERIPNSERNDAT----------SPGTVPEIERM--- :  256
Arabidopsis_thaliana_Rad21-4        : NTQNEPEEERAEHVHVTSPCCSHITT---------EMEDPGQVMNEAGANVVPDKPDAVPPLETPGEENRDHFAIATEVNQETDSSLQGDEQAYSR---- :  411
Oryza_sativa_Rad21-2                : --------------------DKSTRG---------KKPQRE--LNRDENGNSACHIPLSGSKEVQIPENIVENVTFPSRD------ANCPTIEESE---- :  351
Oryza_sativa_Rad21-3                : ----NMDE------------EISVDP---------SQSNNSEAVNTSNINQADKAPDLPEIMREAPFEGPELNLPDIDEVNNDPMDVTEESSPFVS---- :  262
Homo_sapiens_Rad21                  : ---------------------LLLES---------EQSTSN---LNEKINHLEYEDQYKDDNFGEGNDG-----------------GILDDKLISN---- :  236
Mus_musculus_Rad21                  : ---------------------LLLEP---------EQSTSN---LNEKMNHLEYEDQYKDDNFGEGNDG-----------------GILDDKLISN---- :  236
Danio_rerio_Rad21                   : --------------------AHLPDK-----------------TPNLDYDDFGDNNLENSDG------------------------GILMDKILSN---- :  232
Xenopus_(Silurana)_tropicalis_Rad21 : --------------------NLLLEP---------EQSTSQ---LNEKSNHLEYDDQYKDDNFGEGNDG-----------------GILDDKLLSN---- :  234
Drosophila_melanogaster_Rad21       : --------------------PHSLDA---------HADMPGSRLDGDGFGDSFGQPALFEDDLFGDPSQPVEQITKE---------STTVLNADDS---- :  257
Caenorhabditis_elegans_Rad21        : ------------------------DV---------AANGTEISRIDADSVIFSEGPTRPNLIFDNQEGGNFMPEMN----------LKVENQTLEN---- :  254
Schizosaccharomyces_pombe_Rad21     : ---VHSDNQS----------QISIEV---------GRDAPA--AAATDLSGIIGPQMTKSPASSVT--------------------HFSTPSMLPI---- :  268
Sordaria_macrospora_Rad21           : ------SKFM----------DTTIEM---------GRDAPD--ARAVEDDIFSELSAGPGKQRNTREP------------------SLGLDLDYGD---- :  254
Neurospora_crassa_Rad21             : ------SKFM----------DTTIEM---------GRDAPN--ARAVEDDIFSELSAGPGKQRGTREP------------------SLGLDLDYGD---- :  254
Aspergillus_niger_Rad21             : -------------------HDFSMEV---------GRDAPA--PRPVEEDLFSDGGKFNDVDNLQLD-------------------LGEDDAPLDK---- :  234
Thalassiosira_pseudonana_Rad21      : DEEEDMHVFQADA-------DLDEEG---------GVKGRE-SVASRVELVRGANDSIASDRQLQLQETPDQSAKAPG--------SPMSEPDFGG---- :  335
Phaeodactylum_tricornutum_Rad21     : ----EGEEV-----------PFQDDS---------HVSDIE--ITRAANDSISSEAQIGRASILDKPVNSSLDSSK----------MNLTAGGLTD---- :  270
Fragilariopsis_cylindrus_Rad21      : --------------------DDDDDK---------QSQVSDIEIVRAANDSMLSEDQTRRSTSMIGDKSAFTEDGTGA--------APGSAARAAA---- :  255
Pseudo-nitzschia_multiseries_Rad21  : --------------------QDGDDY---------DFNVPF---------------------------------------------GDDDEEEEES---- :   74
Pseudo-nitzschia_multistriata_Rad21 : ----EDDDDD----------EGLADG---------SRVSDIEVVRAANDSLLSDDQTRRASSLLDKSALTANSWNINNKNIDN---NENNNETITN---- :  279
Seminavis_robusta_Rad21             : NKQAQSDDEEDEPM------PLADDD---------DAAPPL-AMEDDDEEQLPDSSRVSEIEVARAADDSMLSENNAEISI-----QGDDASVIGKKEKA :  340
```

```
                                         *       620         *       640         *       660         *       680         *       700
Arabidopsis_thaliana_Rec8_(SYN1)    : ----NPREGAE-------------IPTTLIPSPPRHHDIPE----GVNP----TSPQRQEQQENRRDGFAEQMEE----------QNI------------ : 262
Oryza_sativa_Rec8                   : ----TPDGHPQ-------------VPSNLVPSPPRQEDSPQQQENHHAA----SSPLHEEAQQGQF---------------------------------- : 304
Oryza_sativa_Rad21-4/Rec8           : ----TPDGHPQ-------------VPSNLVPSPPRQEDSPQQQENHHAA----SSPLHEEAQQGGASVKNEQEQQ------------------------- : 260
Homo_sapiens_Rec8                   : ----EEAILLE-------------IPRLPPPAPAEVEGIGE-------------ALGPEELRLTGWEPGALLMEV-----------TP------------ : 275
Mus_musculus_Rec8                   : ----ALEGAGL-------------VSALSPPAPAQVEGIQE----ALPG----QVFPPEVQKMTGWEPGALLTEV-----------TP------------ : 314
Schizosaccharomyces_pombe_Rec8      : ----NGDIQEL-------------TKGTINSDPSLQAASQH-----SNL----GSVQREYNSEEQESRIHMFEID------------------------- : 257
Sordaria_macrospora_Rec8            : ----QDDLLIN-------------GGTAPPPEADEVPGL---------------VPDQVEQAQENQVMAEAEEEQ----------VIA------------ : 320
Neurospora_crassa_Rec8              : ----QGDLLMS-------------GGTAPPPDADEVPGL---------------VPDQVEQAQEDQLMEDVEEAQ-----------VI------------ : 319
Arabidopsis_thaliana_Rad21          : ----MSTLEKP-------------EPVSVTGSRDLQEGVEKCRDHNEAEMADFELFHGSHKEQSETSEVNLHGSEKGFLSDMTVSKDP------------ : 412
Oryza_sativa_Rad21                  : ----SNDIPSD-------------LPTLEFPEREKMLSAPDVELYQANDLGQVTAEKGTTESDGSNKVGSLTSRKRHLEDSLPALESATTEKLSSRPRGK : 562
Ricinus_communis_Rad21              : LRDHVDVFSSE-------------NLPPVFTHQQNDASEPNISLDQGLNEKKTPSPFKEDVIPSGGQSSPFQQRSESPNSASRDAPGIFDSHI------- : 357
Theobroma_cacao_Rad21               : RDAPRDFYTEN-------------LPPVFSDHRNDTPEMTGTLEEVLNE----KEAHTPSLNMLASGVPSMPFQQHSDPPTSAVSKGP------------ : 314
Zea_mays_Rad21                      : QVVASNDRLNE-------------LATSEFAEPEKMLSVPDSEFNHPIDLGLITAEKGTTESDGSNIIGSLASRKRHLEDSLPALESETTERLSSRPHGI : 564
Arabidopsis_thaliana_Rad21-3        : ----RDAAHDL-------------SPTSHPSFAAQQQDVRVERTESLDETLNEKEPTIPSIDEEMLNSGRHSAFELRSGSPGSAAGSE------------ : 327
Arabidopsis_thaliana_Rad21-4        : ----PDGQLNNAHETDEQLGNLTGFTDSDFPPPEKVLAVPNRQGDGNDFMVE-STPDKEDPGTCNDDAGNNNITGKKRTFTESTLTAESLNSVESVGLIQ : 506
Oryza_sativa_Rad21-2                : ----NGSLHGT-------------NTNPSCDGFEEPGSLEQ-----PTL----RCKTKLINELSPSTPEPMTEGGTGLPCSPKFMVTT------------ : 413
Oryza_sativa_Rad21-3                : ----KNITPPA-------------LERTISPGQGGLSGTSIPNARGSTS----TTYDNIEDVIPMDIGMPDFRIEPSPPRVQDEMNAQ------------ : 329
Homo_sapiens_Rad21                  : ----NDGGIFD-------------DPPALSEAGVMLPEQPAHDDMDEDDNVSMGGPDSPDSVDPVEPMPTMTDQTTLVPNE------------------- : 300
Mus_musculus_Rad21                  : ----NDGGIFD-------------DPPALSEAGVMLPEQPAHDDMDEDDNGSLGGPDSPDSVDPVEPMPTMTDQTTLVPNE------------------- : 300
Danio_rerio_Rad21                   : ----EDGGGIF-------------DDPPAITDSVMMPQDHGDDDDDFDNFSPAGGPDSPDSGPVEPLPNTTDQTDQTEQTTLVPNEDE------------ : 303
Xenopus_(Silurana)_tropicalis_Rad21 : ----DAGGIFD-------------DPPAMPEEGVAMPEQPVHDDLDDDDNVSMGAPDSPDSVDPVEPLPTMTDQTTLVPNE------------------- : 298
Drosophila_melanogaster_Rad21       : ----DEDAIDN-------------IHNVPSPATSLVNSIED----EKEE----NNLNGHASVSENVPMNEITLVQ-----------NE------------ : 309
Caenorhabditis_elegans_Rad21        : ----DGGVGPA-------------DMFSSMIHPVREHAVADVQNDDGMDFD--YQPFEPENVEPSRPQS------------------------------- : 304
Schizosaccharomyces_pombe_Rad21     : ----GGTSLDD-------------ELLAPVDDLNLDLGLDDLLGDEQGA----NAPAIEADEQAETSSIHLPSDIMEDDSSRPAAAGV------------ : 335
Sordaria_macrospora_Rad21           : ----GVRIHDD-------------EGDIQMGDDDLQFHVGDHSEIPEGL----STPGAPDMARARISESPLSDIDDEFAKEVEQEYSRH----------- : 322
Neurospora_crassa_Rad21             : ----GVRIADD-------------EGDIQMGDDDLQFNVADHSEIPEGL----STPGAPDMARTRISESPLSDIDDEFAKEVEMEYSRH----------- : 322
Aspergillus_niger_Rad21             : ----MDLGDDA-------------LPDNLLQTDDAMDLGDDHDE---------PAPNADDELERESALSSMADDDLDDLEKSQAFELD------------ : 296
Thalassiosira_pseudonana_Rad21      : ----QDHGDID-------------FDVDTSGQPKDVSGLGLSVDSKDDE----SLEHRRHSDLGSLEISGLDEEE----------GSE------------ : 392
Phaeodactylum_tricornutum_Rad21     : ----QNEFPIA-------------DDTEMIPFDDEDDTRHDIRNASLNL----SVSRDGSAGNATVAIGGLDDSQ----------DSA------------ : 327
Fragilariopsis_cylindrus_Rad21      : ----GGENPDD-------------YDFNVPFDDDESEDEQGVGDFTAGD----GGGLNLDLSRDGGTPGGNNSVANMSGLAMDDDDSA------------ : 322
Pseudo-nitzschia_multiseries_Rad21  : ----ADGRGGL-------------AAAAAAPLDDSALQLSR------DG----GTPLSKTGNADALLGLSMDDAS----------PQQ------------ : 125
Pseudo-nitzschia_multistriata_Rad21 : ----TNPQEED-------------YDFTVPFGEDDSDADEPVATTRLSD----AGLGDSALQLSRDGGTPLSTGNAGLGLGLDADEDS------------ : 346
Seminavis_robusta_Rad21             : APPATPGGMSE-------------ASFPIPGDDDDVAAMGQIPLGDVDDDDDNAAVNINDDEENAGDLSFSKDSTTGGGAAVGGLDES------------ : 415

                                         *       720         *       740         *       760         *       780         *       800
Arabidopsis_thaliana_Rec8_(SYN1)    : ---------PD------------KEEHDRPQPAKK------------------------------RARK--------TATSAMDYEQTIIAGHVYQSWLQ : 303
Oryza_sativa_Rec8                   : -----------------------KHMGQQPAKSSKRK----------------------------KRRK--------DDEVMMDNDQIMIPGNVYQTWLK : 345
Oryza_sativa_Rad21-4/Rec8           : -----------------------KMKGQQPAKSSKRK----------------------------KRRK--------DDEVMMDNDQIMIPGNVYQTWLK : 301
Homo_sapiens_Rec8                   : ---------PE------------ELRLPAPPSPERRPPV----PP-----PP-------------RRRR--------RRRLLFWDKETQISPEKFQEQLQ : 324
Mus_musculus_Rec8                   : ---------PQ------------ELRLPAPPSTEKRLPSLQRPLP--------------------RRHR--------RRQLLFWDKETQISREKFEEQLQ : 365
Schizosaccharomyces_pombe_Rec8      : ---------ED------------VLPLPVPLQSVMDSEHNENEPR-----AL-------------KRRK--------VQKLLEPDENIELSTRTLSQWRK : 310
Sordaria_macrospora_Rec8            : ---------EE------------EPVAEASAAPVR------------------------------RRQR--------RRVVLAPDEDTKISRHELKSWSN : 361
Neurospora_crassa_Rec8              : ---------AE------------EPVVEAVAAPVQ------------------------------RRQR--------RRTVLVPDHDTKISRHELKSWST : 360
Arabidopsis_thaliana_Rad21          : ---------SS------------EFNATDTPVTVTPKTPSRLKISEGGTSPQFSIIPTPAAKESSRVSR--------KRKCLIDD-EVIIPNKVMKEMIE : 482
Oryza_sativa_Rad21                  : RTIDGIPDDDDLLASILVGRRTPGLRLDSTPIPPKTSSLKRPRLT-----SK-------------STTP--------KRKVQMDD-AMVIHADTIRQQLI : 635
Ricinus_communis_Rad21              : -----------------------SFGRVSPELAIRSTPAVQQP----------------------RPRT--------RKRKHFFDEATVLTNKFMKKALE : 404
Theobroma_cacao_Rad21               : ---------PEV-----------SFGCASPQLVIQQSPPVQQP----------------------RQRR--------IPRRRFFDEKLVLPNRLMKRALE : 364
Zea_mays_Rad21                      : RTNDFVPHDDDILASILVGRRTPGFTLDSTPLPPRESSL--------------------------KRRRLGSKMGTLKRKVQIDD-AMVLHADTIRQQLI : 637
Arabidopsis_thaliana_Rad21-3        : ---------EERA----------DFVHPSPQLVLQPSPPPQ------------------------PQRR--------ARKRKNFDGVTVLTNKNISERLK : 376
Arabidopsis_thaliana_Rad21-4        : SKRTADSVPDDDDLLSSILVGKSSFLKMRPTPVLEPATT--------------------------KRLRSAPRSTATKRKVLMDD-PMVLHGDIIRQQLT : 579
Oryza_sativa_Rad21-2                : ---------PA------------KKEKHRVTRKRRRGLYNKDYIPTDRGDKR-------------KVRR--------RGTWVLYDENIVLPNETLRNTIE : 471
Oryza_sativa_Rad21-3                : ---------PVQ-----------GEINAQPVQDEMNAQPVQDEMN-----AH-------------PAQD--------KRRIRYDN-EIVFSNAYMKRQID : 382
Homo_sapiens_Rad21                  : ---------EE------------AFALEPIDITVKET----------------------------KAKR--------KRKLIVDS-VKELDSKTIRAQLS : 342
Mus_musculus_Rad21                  : ---------EE------------AFALEPIDITVKET----------------------------KAKR--------KRKLIVDS-VKELDSKTIRAQLS : 342
Danio_rerio_Rad21                   : -----------------------AFALEPIDITVKET----------------------------KAKR--------KRKLIVDS-LKELDSKTIRAQLS : 343
Xenopus_(Silurana)_tropicalis_Rad21 : ---------EE------------AFALEPIDITVKET----------------------------KAKR--------KRKLIVDS-VKELDSKTIRAQLS : 340
Drosophila_melanogaster_Rad21       : ---------DE------------GFALAPLDVSMYKGVT--------------------------KAKR--------KRKLIIDE-IKNISGEEMKAQLA : 353
Caenorhabditis_elegans_Rad21        : ---------PE------------SFALEPLDVEHMEGRK--------------------------KRQR--------KARKLIVDAETMISNDAFREQQE : 349
Schizosaccharomyces_pombe_Rad21     : ---------EE------------GQVVESATAPQQEKINPQ------------------------KTVR--------RQRAIIDP-VTELSSKQMKKQLA : 381
Sordaria_macrospora_Rad21           : ---------SN------------SDMYEPGEDPTASTIIAP------------------------QRSK--------KRKLLQPDEQTMLSNAQIKEQQS : 369
Neurospora_crassa_Rad21             : ---------NN------------SDMYEPGEDPTASTIIAP------------------------QRSK--------KRKLLQPDEQTMLSNAQIKEQQS : 369
Aspergillus_niger_Rad21             : ---------VN------------DVNDEDDEVTAKHAQP--------------------------PKRR--------KVVLMALDKETDQKVKDVREQYL : 341
Thalassiosira_pseudonana_Rad21      : ---------GQ------------KKRKGKETGPK-------------------------------RMKK--------RRKIIIDNNMTELSGEHMKSMLR : 432
Phaeodactylum_tricornutum_Rad21     : ---------VK------------GSVGNKRASVAP---------------KR-------------RTRK--------RRKVVVDNDDTELPNKHIKDMLA : 370
Fragilariopsis_cylindrus_Rad21      : ---------TQ------------ASAANKRPHEEKKP-------------RK-------------RRRK--------RRKVVIDNNATELTTEHIRDMIS : 367
Pseudo-nitzschia_multiseries_Rad21  : ---------QQ------------QMQMQPPSAAAAATTK-----P-----KK-------------RRKR--------RRKVVIDNHETELTNDHIRGMLK : 173
Pseudo-nitzschia_multistriata_Rad21 : ---------AGNA----------GGLSDPSKREQLQREEEPKPPP-----RK-------------RRRK--------RRKVVIDNHETELTNDHIRGMLR : 401
Seminavis_robusta_Rad21             : ---------PA------------APAQPSVSDEDEEEEKPKKKRTRQEGPKK-------------LRRT--------RRKIVIDNDSTELEGEHIKKMLR : 473
```

```
                                        *       820         *       840         *       860         *       880         *       900
Arabidopsis_thaliana_Rec8_(SYN1)    : DTS--DILCRGEKRKVR-----------------------GTIRPDMESFKRAN---------------------------------------------- :  332
Oryza_sativa_Rec8                   : DPS--SLITKRHRINSK-----------------------VNLIRSIKIRDLMD---------------------------------------------- :  374
Oryza_sativa_Rad21-4/Rec8           : DPS--SLITKRHRINSK-----------------------VNLIRSIKIRDLMD---------------------------------------------- :  330
Homo_sapiens_Rec8                   : TRA--HCWECPMVQPPE-----------------------RTI--------------------------------------------------------- :  342
Mus_musculus_Rec8                   : TGA--HCWEYPVAQPPK-----------------------RML--------------------------------------------------------- :  383
Schizosaccharomyces_pombe_Rec8      : -----NYVERMIALEAT-----------------------KYVRRRGASSAKK----------------------------------------------- :  335
Sordaria_macrospora_Rec8            : D----YLANAERTNNAA-----------------------RRAAATNPAAAKKHAYDLVFGRGVGGVGSFDDGLNSGNHRS------------------- :  415
Neurospora_crassa_Rec8              : N----YLANAERANNAA-----------------------VRGAAMNPTTAKKHAYELIFGRGVGRVGALESGLNSENHRS------------------- :  414
Arabidopsis_thaliana_Rad21          : DSS--KLLAKRRNVPHT------------------------------DCPERRTKRFA------------------------------------------ :  508
Oryza_sativa_Rad21                  : STE--DIRRIRKKAPCT-----------------------RSEIWMIEKGSLEDDIFHEPIFSCMCKELNDLQ--------------------------- :  683
Ricinus_communis_Rad21              : NSK--DILRKRREIPST-----------------------ALGIWKLRNSLRKEQVLSEPLLTGSC---------------------------------- :  445
Theobroma_cacao_Rad21               : DCS--DLVRKRRKLPSS-----------------------ALGVWKLNCS-------------------------------------------------- :  389
Zea_mays_Rad21                      : NTE--DIRRIRKKAPCT-----------------------RSEIWMIEKGSLEDDIFHEPIFSCLSEELNKLHNRTYEAIVHPAVQNMELQGQL------ :  706
Arabidopsis_thaliana_Rad21-3        : DPS--DTLRKRKKMPSS-----------------------KLKFWRMNNQSRKD---------------------------------------------- :  405
Arabidopsis_thaliana_Rad21-4        : NTE--DIRRVRKKAPCT-----------------------VPEIVMLQRQALEDGLFKEPIFTGMSVELVSLHTEPYDLRG------------------- :  635
Oryza_sativa_Rad21-2                : DAS--DLVQQRRKAPHT------------------------CLYTWKEGKIR------------------------------------------------ :  497
Oryza_sativa_Rad21-3                : GSELHRLVSKRRKLLQA-----------------------AVDVWKFNRIRQKDGFLLDPLVHGMC---------------------------------- :  425
Homo_sapiens_Rad21                  : DYS--DIVTTLDLAPPT-----------------------KKLMMWKETGGVEKLFS------------------------------------------- :  374
Mus_musculus_Rad21                  : DYS--DIVTTLDLAPPT-----------------------KKLMMWKETGGVEKLFF------------------------------------------- :  374
Danio_rerio_Rad21                   : DYS--DIVTTLDLAPPT-----------------------KKLMMWKETGGVEKLFS------------------------------------------- :  375
Xenopus_(Silurana)_tropicalis_Rad21 : DYS--DIVTTLDLAPPT-----------------------KKLMMWKETGGVEKLFS------------------------------------------- :  372
Drosophila_melanogaster_Rad21       : DTS--DILTTLDLAPPT-----------------------KRLMYWKETGGVEKLFSLPSRSIPARALFG------------------------------ :  398
Caenorhabditis_elegans_Rad21        : DFS--DTMRVVEMAPPT-----------------------RKMFNLCVSGDLQHLSR------------------------------------------- :  381
Schizosaccharomyces_pombe_Rad21     : DTS--SITSPLCLNTSS-----------------------IVFNATVNF--------------------------------------------------- :  405
Sordaria_macrospora_Rad21           : KRD--NILKPQTFGTQD-----------------------PYLMALLDL--------------------------------------------------- :  393
Neurospora_crassa_Rad21             : KRD--NILKPQSFGTQD-----------------------PYLMALLDL--------------------------------------------------- :  393
Aspergillus_niger_Rad21             : TRE--EIMKPSSWLPRD-----------------------PVLLTLMNM--------------------------------------------------- :  365
Thalassiosira_pseudonana_Rad21      : DTS--DIIMQNRFNIAD---------------------------MPEEEEEEDYTMVKINRLLET----------------------------------- :  469
Phaeodactylum_tricornutum_Rad21     : DTS--DIVLQNVVHPAT----------------------------WVPGQDKASHKRSARDLLFVH---------------------------------- :  406
Fragilariopsis_cylindrus_Rad21      : NTD--GIVRT-MIHPAS---------------------------IWDEEGLGRDYKTLVKERSAQDGITIINFITTSTGVNNKKKSST------------ :  425
Pseudo-nitzschia_multiseries_Rad21  : DTD--DIV-IPMIHPAS---------------------------LWDEDDTGKDYRTLVRERRARDEGTNSNTNTNTRSNKRRKKTVGFADGGAASISFS :  243
Pseudo-nitzschia_multistriata_Rad21 : STD--DIVRP-MVHPAS---------------------------IWDEEGTGKDYRALVLDRIAEQRGASRKRKKGVAFRDVPGPAS------------- :  458
Seminavis_robusta_Rad21             : DTS--DITLQNVAHPADWNVDGNNSNDNNTLDASLSQASPGNLTLGTQEQEAASHFSGLATLSAMTQGTEGMRPNSANGRILEFGEGEDDHLLRDT---- :  567

                                        *       920         *       940         *       960         *       980         *      1000
Arabidopsis_thaliana_Rec8_(SYN1)    : -----------MPPTQLFEK----------------------------------------DSSYPP---------------------------------- :  347
Oryza_sativa_Rec8                   : -----------LPLVSLISSLEKSP----------------------------------LEFYYPK---------------------------------- :  395
Oryza_sativa_Rad21-4/Rec8           : -----------LPLVSLISSLEKSP----------------------------------LEFYYPK---------------------------------- :  351
Homo_sapiens_Rec8                   : -----------RGPAELFRTPTLSG-------------------------------------WLPP---------------------------------- :  360
Mus_musculus_Rec8                   : -----------TSPAELFRTPTLSG-------------------------------------WLPP---------------------------------- :  401
Schizosaccharomyces_pombe_Rec8      : -----------KELNKFFDWESFHP--------------------------------------------------------------------------- :  349
Sordaria_macrospora_Rec8            : -----------HPLAALFAGVDFAA------------------------------EVLDIDIDIDE---------------------------------- :  440
Neurospora_crassa_Rec8              : -----------HPLAALFSGANFAS--------------------------------EALGIDIDE---------------------------------- :  437
Arabidopsis_thaliana_Rad21          : -----------NPFRSFLEPLIQYG------------------------------SSDLQSLFCQP---------------------------------- :  533
Oryza_sativa_Rad21                  : -----------YRTYEIVAHPTIHN------------------------------MEIHVRLDMSQ---------------------------------- :  708
Ricinus_communis_Rad21              : -----------ADLCNLLKDYISGK------------------------------PHLTLEREVTPEPRVTTSAPAI----------------------- :  481
Theobroma_cacao_Rad21               : -----------QRMGQVFDEPSFTG--------------------------------SCADICIMF---------------------------------- :  412
Zea_mays_Rad21                      : -----------DMPEAITEDSNIAGFGAATIDGPLHIPDGIQSDAMLPRANDAYGATAAFSLQVPPNNQVNGASNDFVIGTLFQGVTGPFIGNEKEVALA :  795
Arabidopsis_thaliana_Rad21-3        : ---------------QNFNEPLFTG--------------------------------------FSD---------------------------------- :  418
Arabidopsis_thaliana_Rad21-4        : -----------IMIIENDDRHASVGAVEDNECSVTAVEENKTEESSDPQAHPNDCEEQPGTAHTHP---------------------------------- :  690
Oryza_sativa_Rad21-2                : -----------SLPVTFMDPLIL----------------------------------------YPT---------------------------------- :  512
Oryza_sativa_Rad21-3                : -----------ATLRQTYERTFPHV--------------------------------------IDP---------------------------------- :  442
Homo_sapiens_Rad21                  : -----------LPAQPLWNNRLLKL----------------------------------FTRCLTP---------------------------------- :  395
Mus_musculus_Rad21                  : -----------LPAQPLWNNRLLKL----------------------------------FTRCLTP---------------------------------- :  395
Danio_rerio_Rad21                   : -----------LPAQPLWNGRLLKM----------------------------------FTRCLTP---------------------------------- :  396
Xenopus_(Silurana)_tropicalis_Rad21 : -----------LPAQPLWNSRLLKL----------------------------------FTRCLTP---------------------------------- :  393
Drosophila_melanogaster_Rad21       : -----------NYNRQLFSHSTFFEDFSSVVPMEILALEFYTKENENALIIFNKKGRKRKNDNMSN---------------------------------- :  453
Caenorhabditis_elegans_Rad21        : -----------EPGCKMFNR-------------------------------------------------------------------------------- :  390
Schizosaccharomyces_pombe_Rad21     : -----------TRNGKFNTSIFSSN--------------------------------------LNP---------------------------------- :  422
Sordaria_macrospora_Rad21           : -----------QKTGAFVSNVLLEN---------------------------------RSDSWADE---------------------------------- :  415
Neurospora_crassa_Rad21             : -----------QKNGAFVSSVLLEN---------------------------------RSDSWADE---------------------------------- :  415
Aspergillus_niger_Rad21             : -----------QKNGDFVSNVLGAG----------------------------------RGRGWAP---------------------------------- :  386
Thalassiosira_pseudonana_Rad21      : -----------VPMERLLARPCLGD-----------------------------------DGDLAP---------------------------------- :  489
Phaeodactylum_tricornutum_Rad21     : -----------LSYEKILARPALGD-----------------------------------DGQLAP---------------------------------- :  426
Fragilariopsis_cylindrus_Rad21      : -----------KIIVPSLTRPFLVD----------------------------ESEMNGSESRLHP---------------------------------- :  452
Pseudo-nitzschia_multiseries_Rad21  : SSSSSSKGAGSHAAVPSLTRPFLED------------------------------AADRGGPALHP---------------------------------- :  279
Pseudo-nitzschia_multistriata_Rad21 : -----------SGGVPSLTRPFLAD------------------------------ASESGGPGLHP---------------------------------- :  483
Seminavis_robusta_Rad21             : -----------LPFEELLIRPSLGD-----------------------------------DGQLAP---------------------------------- :  587
```

```
                                               *      1020         *      1040         *      1060         *      1080         *      1100
Arabidopsis_thaliana_Rec8_(SYN1)    : -----QLYQLWSKNTQVLQTS---------SSESRHP------------DLRA-----------EQSPGFVQERMHN----------HHQTDHHERSDTS :  400
Oryza_sativa_Rec8                   : -----ELMQLWKECTEVKSPK-APSSGYYTICSF---------------LSPF-----------WITKGTVAIFFKG----------GQQSSSPEQQQRN :  453
Oryza_sativa_Rad21-4/Rec8           : -----ELMQLWKECTEVKSPK---------------------------------------------------APSSG----------GQQSSSPEQQQRN :  385
Homo_sapiens_Rec8                   : -----ELLGLWTHCAQPPPKA---------LR-------------------------------------RELPEEAA----------AEEERRKIEVPSE :  399
Mus_musculus_Rec8                   : -----ELLGLWTHCAQVPQRM---------LR-----------------QRPQ-----------LETEETVEEERAA----------DEEERRKTEALSE :  449
Schizosaccharomyces_pombe_Rec8      : ------LLKPWIEKLKPS-------------------------------NNTP-----------SEIDDVLRNIDTS----------EVEVGRDVQGELG :  391
Sordaria_macrospora_Rec8            : -----GHEAGGRRGRRRSALE---------ALELEEEDAE---------RRVR-----------RRLSDENEEEHHA----------QSQQNVAEPQAEA :  496
Neurospora_crassa_Rec8              : -----EVEAEGRRSRRRPALE---------ALELEEEDAE---------RRVR-----------RRLSDENGEEHQA----------QAQPNTEPQTEAG :  493
Arabidopsis_thaliana_Rad21          : -----IKLKNWATTGTPKDTK----------------------------IARH-----------KEKSSLDTVRSPG----------VILSSDQTENTQE :  579
Oryza_sativa_Rad21                  : -----TMADGSNDVGTSGAKDSGNHQDHVVLPDGAESDA----------MHPE-----------ATDAADTRTDFDS----------HMPSDKQVNNVEG :  772
Ricinus_communis_Rad21              : -----EVIPEWRDATSPARSTVAILESIAATSP----------------TSAT-----------EMISESRTTTSPA----------PATENIPEPMFAQ :  539
Theobroma_cacao_Rad21               : -----NGQYISTKSHLVVPKK----------------------------VVPE-----------PTLLQSTAPTTDA----------ISELRIGQYDASI :  458
Zea_mays_Rad21                      : DRERAQVNTLYSDHFQDVPSDLHSTDANISSQDVTLDNSGQACAHATDGMTRELTHFVHSNANIFESNEVPASEITGVEYNQGASGFPQSTEDENAVGYN :  895
Arabidopsis_thaliana_Rad21-3        : -----DLRNVFEKDYVASKPH-------------------------------------------LAVSDETLPEPAS----------VSPTREAEVEINP :  460
Arabidopsis_thaliana_Rad21-4        : -----QEEQTINQQEELKDDNELAEKSDLEVLKEGNGAADEVNLVVIDDVSQI-----------PSEEKLDRVEDLQ----------VEESHENHDGEGG :  764
Oryza_sativa_Rad21-2                : -----SVYVRHTITADTPENS----------------------------CRES-----------VKSRRRLSLELSE----------SNNICDDAKNVEG :  558
Oryza_sativa_Rad21-3                : -----EAESGSVEHTPGVAND---------SIQD---------------THDH-----------QLSPKSPGNTDAQ----------PEHQFNQQAPRNS :  492
Homo_sapiens_Rad21                  : -----LVPEDLRKRRKGGEAD---------NLDE---------------FLKE-----------FENPEVPREDQQQ----------QHQ----QRDVID :  441
Mus_musculus_Rad21                  : -----LVPEDLRKRRKGGEAD---------NLDE---------------FLKE-----------FENPEVPREEQQP----------QQQQPQPQRDVID :  445
Danio_rerio_Rad21                   : -----LVPDELRKRRKGGEAD---------SLED---------------FLKE-----------LENPEVPREEVLG----------------HRSDVID :  440
Xenopus_(Silurana)_tropicalis_Rad21 : -----LVPEDLKKRRKGGEAD---------NLDE---------------FLKE-----------FENPEVPREELRP------------------QDVID :  435
Drosophila_melanogaster_Rad21       : -----LFLDHVPDSVVQSLEA---------PEVLRA-------------NHKS-----------LGVSTVSVEIVSK----------EQESISCQNELTF :  505
Caenorhabditis_elegans_Rad21        : -----ELLQRYRRCLVTREFD----------------------------LNYT-----------MQELSDSSSFTPS----------MEAQAEPWEDLNL :  436
Schizosaccharomyces_pombe_Rad21     : -----KVNELLQADFKQAIL-----------------------------RKRK-----------NESPEEVEPAKHQ----------RTDTSTENQETAE :  467
Sordaria_macrospora_Rad21           : -----LSDMLSLTTFRPFDLK----------------------------RKRD-----------SAIADMEVDEEDG----------TRKSPRLELPDDE :  461
Neurospora_crassa_Rad21             : -----LSDMLSLSTFRPFDLK----------------------------RKRD-----------SAIADMEVDDEDG----------TRKSPRLELPDDE :  461
Aspergillus_niger_Rad21             : -----ELRDLLSFDTIRKAGE----------------------------LKRK-------------------RDSG-----------IADMDVDAATAPA :  423
Thalassiosira_pseudonana_Rad21      : -----ELLALWGRNTVKITGK---------PG-----------------TQLP-----------FRMRGAKGEEQRG----------AAAEQLMEEEAEE :  537
Phaeodactylum_tricornutum_Rad21     : -----ELLALWAKNTAPVVGK---------PFPY---------------ELRV-----------GANEEDESDEDES----------IEEPRRRQSHEAD :  476
Fragilariopsis_cylindrus_Rad21      : -----DLQKLWQDNYWKALDQ---------PCPF---------------KRRD-----------VVAAEDEPVDDVE----------RVRRDDGSKADDD :  502
Pseudo-nitzschia_multiseries_Rad21  : -----LLKGLWEENYWKALGK---------PCPY---------------KRRS-----------NKSDNHNDNDNDD----------EATADDVERVRRG :  329
Pseudo-nitzschia_multistriata_Rad21 : -----VLQRLWEDNYWRANER---------PCPY---------------RRLD-----------PGGDGNDNDNDKD----------DKVVDDVEHVRRG :  533
Seminavis_robusta_Rad21             : -----ELLQLWRNNLAHVEGK---------PSPYPKLA-----------AKDD-----------AEEEEEEEEVEKA----------RADKDQESKDDQS :  641

                                               *      1120         *      1140         *      1160         *      1180         *      1200
Arabidopsis_thaliana_Rec8_(SYN1)    : SQNLDSPAEILRTVRTGKGASVES-----------MMAGSRASPETINRQAA-----DINVTPFYSGDDVR---------SMPSTPSARGAASINNI--- :  472
Oryza_sativa_Rec8                   : LPPQAFPTQPQVDNDREMGFHPVD-----------FADDIEKLRGNTSGEY------GRDYDAFHSDHSVTPGSPGLSRRSASSSGGSGRGFTQLDP--- :  533
Oryza_sativa_Rad21-4/Rec8           : LPPQAFPTQPQVDNDREMGFHPVD-----------FADDIEKLRGNTSGEY------GRDYDAFHSDHSVTPGSPGLSRRSASSSGGSGRGFTQLDP--- :  465
Homo_sapiens_Rec8                   : IEVPREALEPSVPLMVSLEISLEA-----------AEEEKSR---------------ISLIPP---EERWA-----------------------WPE--- :  444
Mus_musculus_Rec8                   : IEVLREAQEPSGPLMLSSELSLEA-----------AEDEKSR---------------TSLIPP----EWWA-----------------------WSE--- :  493
Schizosaccharomyces_pombe_Rec8      : LNIPWNTSSRSNSAINSKSHSQTG-----------SEH-------------------STPLLDTKYRKRLP-------------------------H--- :  433
Sordaria_macrospora_Rec8            : GRGLLTGEAEEDAEIGRRDGSALP-----------DPDIPSDAPWNRAPS-------LVPGSSVKGDSHMPGSSRQVSASPLHSRGSHLAHLPPIDR--- :  575
Neurospora_crassa_Rec8              : RGGLPTGGVEEDAEIGRR--DGSA-----------LPDIPSDAPWNRPSS-------LVPGSSVKGDNHHPGSSRQVSASPLHSRGSRLAHLPPIDR--- :  570
Arabidopsis_thaliana_Rad21          : IMETPQAAALAGLKVTAGNSNVVS-----------VEMGASSTTSGTAHQTENAA--ETPVKPSVIAPETP------VRTSEQTVIAPETPVVSEQV--- :  657
Oryza_sativa_Rad21                  : VTEQLTDNEKETAVVEKATTNMGDSAQVDSLDKEYLQDVPADLQRSTNTNTPLFVLDDMPGPDVVLDSSDP-----VSAQAVDDMKGELSDIVHDNV--- :  864
Ricinus_communis_Rad21              : PSVPEVEPDLEIERLRQHEGHDAN-----------ILLPELLPSPG-----------RFPPSPFRRDDFTPNSMKSLGSETAHWAGTSAGTEVLPTP--- :  614
Theobroma_cacao_Rad21               : PDPRVGFSPVSAPEIECLRRNESN-----------ATDNLFPEFGSIP---------AGSMPSPFRRDDSPFSTNSLQSELAPKAGATSTPDIATFSGTH :  538
Zea_mays_Rad21                      : QDVSGFPRPTEDENAVDYNQDASG-----------FSRPTEDVQDASGFPRPTEDENMVDYNQDASGFPRPTEDVNAVGYNQDASGFARPTEDVNAVGCN :  984
Arabidopsis_thaliana_Rad21-3        : VSPIPDSTNPDSTVQLSPAQQTED-----------VLDSAGPRPAHAESVAT-----EAQSPRTFDNDDMGIEHLRDGGFPVYMPSPPPRSSPFRTD--- :  541
Arabidopsis_thaliana_Rad21-4        : QDVCADPNEKSCTDVIEIAEGDTDINPIFNEMDLKVEDELPHEDEKTDA--------SAEVSELGRDDQTPCDNTVGSTETGCLEAGDLSNMALENC--- :  853
Oryza_sativa_Rad21-2                : ESIPDEPRKRKLDELTDSVQATVG-----------CYTE------------------SAQYHN---DEDYR---------------------FNDDT--- :  602
Oryza_sativa_Rad21-3                : DGQPEPELNPKSPVNAEAQPESQI-----------NPQSPRNADGQPEPELNPKSPGEAGTSHFDDMPEIP----------------RFSPQNIPSP--- :  562
Homo_sapiens_Rad21                  : EPIIEEPSRLQESVMEASRTNIDE-----------SAMPPPPPQGVKRK--------AGQIDP---EPVMP-----------------------PQQ--- :  493
Mus_musculus_Rad21                  : EPIIEEPSRLQDSVMEASRTTIEE-----------SAMPPPPPQGVKRK--------AGQIDP---EPSIP-----------------------PQQ--- :  497
Danio_rerio_Rad21                   : QTIMEEPSMLQASSMEGSRTALDE-----------SMMPPPSRQRGLKRKSLETLEKDAVLPPMGVLEQTL---------------------QPLDQSVL :  508
Xenopus_(Silurana)_tropicalis_Rad21 : QPILEEASRLQESLMEGSRTHLDE-----------TIMPPPPPQQGVKRD-------SLQMEP----EPVP---------------------VMQET--- :  489
Drosophila_melanogaster_Rad21       : FDNMRSPDLLSLNEMEQF-SSINE-----------LPLTPRNMNHEM----------GDDFNQ---GDSTP--------AGLDHGHATPQHGNIGEM--- :  569
Caenorhabditis_elegans_Rad21        : NEDIQEDIQAQGPAVDEFFNDVRM-----------DDDDDRQ---------------PAQEMDFGDNFDFP-----------------------QEV--- :  484
Schizosaccharomyces_pombe_Rad21     : VLDPEEIAAAELANITEA-----A-----------IATLPQ----------------ETVVQP---EGEAP------------------------EL--- :  505
Sordaria_macrospora_Rad21           : STVVGRAESINGNQSETAEGTEIE-----------LAANED----------------GAVFHD--EDQERP-------------------------G--- :  504
Neurospora_crassa_Rad21             : STVVGRAESINGNQSETAEGTEIE-----------LAANDD----------------GAVFHD--EDQERP-------------------------G--- :  504
Aspergillus_niger_Rad21             : LELGEEEAIVPVDEGVGLDSTLLQ---------------------------------------------------------------------------- :  447
Thalassiosira_pseudonana_Rad21      : VERARSQQQRDSIDGDGVQMEEED-----------IEFGGGGQDVDF----------GDAGKE---EEETP--------------------FDIQDD--- :  590
Phaeodactylum_tricornutum_Rad21     : PDRLLETRQVQLQDMHES---------------------------------------GSIVND---DEPTP-----------------------WDD--- :  508
Fragilariopsis_cylindrus_Rad21      : SDLGSQQSDLNVEKENQQ---------------------------------------PQEGGPPLDEDDFN---------------FPTGDDDEEEE--- :  545
Pseudo-nitzschia_multiseries_Rad21  : AGHADDESTLASQRLDEFNEAGYG-----------LDFDDREHEQRSVVST------GGPRKQTTGDDDVD---------------FPATMDDDDDE--- :  394
Pseudo-nitzschia_multistriata_Rad21 : TAASDDESTVGGRSELGVGAEKDD-----------DDARSRLSTGQ-----------EMPRDD---EMDFP-----------------VANDEEDEE--- :  588
Seminavis_robusta_Rad21             : GEVTVDGKPKVPAEVGGEDMQQQE-----------EDDEPMPLPDDD----------DEPPKPMPDDDVQNDEEGDMGIHGEDAPPLPFDDEEEEEV--- :  717
```

```
                                        *        1220         *        1240         *        1260         *        1280         *        1300
Arabidopsis_thaliana_Rec8_(SYN1)    : EISSKSRMP--------------NRKRPNSSPRRGLEPVAEERPWEHREY--------------------------EFEFSMLPEKRFTADKEILFETAS :  532
Oryza_sativa_Rec8                   : EVQLPSGRS--------------KRQHSSGKSFGNLDPVEEEFPFEQELR----------------------------DFKMRRLSDVGPTPDLLEEIEP :  591
Oryza_sativa_Rad21-4/Rec8           : EVQLPSGRS--------------KRQHSSGKSFGNLDPVEEEFPFEQELR----------------------------DFKMRRLSDVGPTPDLLEEIEP :  523
Homo_sapiens_Rec8                   : VEAPEAP----------------ALPVVPELPEVPMEMPLVLPPELELLS-------------------------------------------------- :  478
Mus_musculus_Rec8                   : EGQPEPP----------------ALPMLPELPEVPMEMP----PRPELSS-------------------------------------------------- :  523
Schizosaccharomyces_pombe_Rec8      : SPSMPSRVE--------------FLPALESSQFHETLNSELSLQLSDDFV----------------------------------------------LYKN :  473
Sordaria_macrospora_Rec8            : FSDTGSYAPLLHSGGVPDFSSDPVMPPGEDEPELPHLPGGNHGSQLLRRS--------------------------PSSEAGAATEVMAETSQVMKDALD :  649
Neurospora_crassa_Rec8              : FSDAGSYAPFLHSGGAPGFSSDPIMPPGEDEPQLPHLPGGPQGGRDLLRC------------------------TPSGEAEMTATQAAAETSQVMRDALD :  646
Arabidopsis_thaliana_Rad21          : EIAPETPVR---------ESMSKRFFKDPGTCYKKSRPASPFTSFEEHPS-------------------------------VYYVENRDLDTILMNDEVN :  717
Oryza_sativa_Rad21                  : NAFDNKDMPTSEITVLEFTQNASGFPQPTEDENVLSAMGENSGLQENHVG-SVMDLDNMGHDFSLKECSDFGSAIQGVDTDFLMYDDEVDCDEANDDEPN :  963
Ricinus_communis_Rad21              : DIAASTG----------------TYTTDLETPRTFLEEQFDAGHTGLSDI------------------------------PEFVNTAEAEDLYFLEADNSP :  669
Theobroma_cacao_Rad21               : GSGIDTPMT--------------LLEEQSHLENTGFSAIPEFGSSEADLY--------------------------------------------FLEEDGNTP :  583
Zea_mays_Rad21                      : QDASGFPRSMKDENAVEYNQDGSGFPRPAENENDVSAMGDNSGFHENNMGSLTDLDMVNDYELKESNDFGSAINGVDTDFLNYDDDDGGDFDDANDDEPN : 1084
Arabidopsis_thaliana_Rad21-3        : DFTTQSGNW--------------ETESYRTEPSTSTVPEDLPGQRNLGLS-------------------------------PVSERTDEELYFLEVGGNSP :  597
Arabidopsis_thaliana_Rad21-4        : NEPLVEANS--------------DGLNPETESYNKYEPHNEMSNEEASMQNALDGEHTSRDGLMGDNDEMDTMENAHDTGFLNVDDDEVDEDHEEDDIQY :  939
Oryza_sativa_Rad21-2                : VKEKDFSIG--------------GHESHSTELQERLNALKSKNPQLDEAL-------------------------------------DADIDSMEEDTHM :  651
Oryza_sativa_Rad21-3                : IRDDNSPFKTPGAGGTPKSRLGETPASGTPADMSYMSPGQDSDPQVSPFP--------------------------FNDELDGDLPEIPSLMSTPGVIS :  635
Homo_sapiens_Rad21                  : VEQMEIPPV--------------ELPPEEPPNICQLIPELELLPEKEKEK-----------------------------------EKEKEDDEEEEDEDA :  544
Mus_musculus_Rad21                  : VEQMEIPPV--------------ELPPEEPPNICQLIPELELLPEKEKEK-----------------------------------EKEKEEEEEEEDEDA :  548
Danio_rerio_Rad21                   : SHQLEMPQV--------------ELPPEDTTNLSRLIPELDLLDEKSKDK------------------------------------------DKDDSDEEGEEG :  556
Xenopus_(Silurana)_tropicalis_Rad21 : EPHIEMPPP--------------PLPPPVELPSEVSETPNINDLIPELNL-------------------------------LPEKEKEKDEEEEEEEEDA :  544
Drosophila_melanogaster_Rad21       : DHDSVIPTK--------------KTAVILNESVGTSVLSDNGVSKRTNNI--------------------------LKGWDNYEIPSFVGQNHGDEQME :  628
Caenorhabditis_elegans_Rad21        : EHQECAPIP-----------IQSGFAGENKENEDAEDWSDPFGSSNSSRR--------------------------------------------GQLEAYGFG :  532
Schizosaccharomyces_pombe_Rad21     : GSPMGFPVT-----------------ALESADDSLFDAPPVMLDEADLLG-------------------------------------SERLDSSVSEALPS :  552
Sordaria_macrospora_Rad21           : SRSANSPVP------------------NFDETIAPLVHPADSGPVSIGTK-------------------------------------------------- :  536
Neurospora_crassa_Rad21             : SRNANSPVP------------------NFDETIAPIVHPADSGPVSIGTK-------------------------------------------------- :  536
Aspergillus_niger_Rad21             : RSEIEFPGD---------------------EEDQALRLSDDEGMNQPLED-------------------------------------------------- :  476
Thalassiosira_pseudonana_Rad21      : FGQVEYKAD--------------DLDEGSVASDRSDRSSFSLGAVNDLEK-----------------------------------ELYQVDDDEDKPRQE :  641
Phaeodactylum_tricornutum_Rad21     : QDELPGPMD------------------DSRDEEHLMNMSGDGSDQPLIFL------------------------------GHTNAALDVEIEDEEDTPRQG :  561
Fragilariopsis_cylindrus_Rad21      : EQEEDAPVD--------------FGDDEEEQDLGVVNRSRDENDMLELGM--------------------------VNDMVLDSDEDDDDGDDDDDLANRQ :  606
Pseudo-nitzschia_multiseries_Rad21  : EEDVDAPVP--------------DFGEEEDDEEHLTAVAAERAGDDPLDL--------------------------------------------------GM :  432
Pseudo-nitzschia_multistriata_Rad21 : DDEMDAPVP--------------DFGDDEEDPTNLAAGGDDEDLLDLGMV---------------------------NDMVLDSDEDDRQDDGEDDEDRE :  647
Seminavis_robusta_Rad21             : QQDAKSTAT--------------KEADDESSRSSEEEKAQQEGENEYGLG--------------------TWGLVNDALGGKGVDDEDDDEDREEDMRQA :  783

                                        *        1320         *        1340         *        1360         *        1380         *        1400
Arabidopsis_thaliana_Rec8_(SYN1)    : TQTQKPVCNQSDEMITDSIKSHLKTHFETPGAPQ--------------------VESLNKLA-VG--MDRNAAAKLFFQSCVLATR--------GVIKVN :  601
Oryza_sativa_Rec8                   : TQTPYEKKSNPIDQVTQSIHSYLKLHFDTPGASQ--------------------SESLSQLA-HG--MTTAKAARLFYQACVLREE--------NKCAIA :  660
Oryza_sativa_Rad21-4/Rec8           : TQTPYEKKSNPIDQVTQSIHSYLKLHFDTPGASQ--------------------SESLSQLA-HG--MTTAKAARLFYQACVLATH--------DFIKVN :  592
Homo_sapiens_Rec8                   : ---------------LEAVHRAVALELQANR-----------------------EPDFSSLV-SPL-SPRRMAARVFYLLLVLSAQ--------QILHVK :  530
Mus_musculus_Rec8                   : ----------------EAVLRAVALKLQANK----------------------ELDFSSLV-PPL-SPRKLASRVFYLLLVLSTQ--------KILLVE :  574
Schizosaccharomyces_pombe_Rec8      : TQEENAHLMLSMEKECANFYEYAKTAIYENNG----------------------RITFSSLLPND--LKRPVVAQAFSHLLSLATK--------SAFLVK :  541
Sordaria_macrospora_Rec8            : RDGRNFLTYVNMVAKTRGETRSLGGGTPSQAVGDGDGNRR--------LRQ---WVAFDELLDEPRDRTRQVATQAFYNVLVLATK--------NAIKVE :  730
Neurospora_crassa_Rec8              : RDGHNFLAYVNMVAKARGETRSLGGGGAPPSSSHAHGDGN--------DRLLRQWVAFDELLDEPRDRTRQVATQAFYNVLVLATK--------DAIKVE :  730
Arabidopsis_thaliana_Rad21          : ADERQDLQQETWSSRTRNVAKFLEKTFLEQREREEEE-----------------KVSLLQLC-RG--RTQKESARLFYETLVLKTK--------GYVEVK :  789
Oryza_sativa_Rad21                  : PEEFQSLDNSGWSSRTRGVARYLKTLFDEESGLGRK------------------SVAIDHLL-SG--KTRKEASRMFFETLVLTTK--------DFISVD : 1034
Ricinus_communis_Rad21              : AGSPGSQGIDSLSVRTRAVAQYLKRNSPITPVSEDLSG----------------DLSLNRIL-QG--KTRKLCARMFFETLCVWFYNQENAWYLSMIKFS :  750
Theobroma_cacao_Rad21               : TESQGSQGVGSLSVRTRAVAKYLKKQSPITPISEDLHR----------------DLSLNKIL-EG--KTRKICARMFFETLVLKSY--------GLVDVR :  656
Zea_mays_Rad21                      : PNEFQSLDNSGWSSRTRGVARYLKALFDDESGLERK------------------SVAIDHLV-RG--KTRKEASRMFFETLVLTTK--------DYISVD : 1155
Arabidopsis_thaliana_Rad21-3        : VGTPASQDSAALTGRARALAQYLKQRSSSSPTTSSHPSG---------------DLSLSEIL-AG--KTRKLAARMFFETLVLKSR--------GLIDMQ :  671
Arabidopsis_thaliana_Rad21-4        : DDETRLLENSGWSSRTRAVAKYLQTLFDKETENGKN------------------VLVADKLL-AG--KTRKEASRMFFETLVLKTR--------DYIQVE : 1010
Oryza_sativa_Rad21-2                : DEQHARDEGLLRSTRTRTVARYFHQLLVDQKCQQRNN-----------------SVCLGQAL-EG--TKRKTSARFFYETLILKSG--------SLIEVN :  723
Oryza_sativa_Rad21-3                : TAGTGTTGLGSMSARTRAVAQYFKDQMASATSDDQPG-----------------KFILNRIL-EG--RHRKQAARMFFETLVLKSY--------DYIDVE :  707
Homo_sapiens_Rad21                  : SGGDQDQEERRWNKRTQQMLHGLQRALAKTGAE---------------------SISLLELC-RN--TNRKQAAAKFYSFLVLKKQ--------QAIELT :  612
Mus_musculus_Rad21                  : SGGDQDQEERRWNKRTQQMLHGLQRALAKTGAE---------------------SISLLELC-RN--TNRKQAAAKFYSFLVLKKQ--------QAIELT :  616
Danio_rerio_Rad21                   : QGGDQDQEERRWNKRTQQMLHGLQRVVAKTGAQ---------------------SIGLLELC-RN--NNKKQAAAKFYSFLVLKKQ--------QAIDLT :  624
Xenopus_(Silurana)_tropicalis_Rad21 : TGTEQDQEERRWNKRTQQMLHGLQRVLAKTGAE---------------------SISLLDLC-RN--TNRKQAAAKFYSFLVLKKQ--------QAIELT :  612
Drosophila_melanogaster_Rad21       : NETDEQFEERVLNKRAAHLFIDVRAHFIAKD-----------------------SLELSQLT-SG--NSRKQAAQKFYSLLVLKKF--------KVLHID :  694
Caenorhabditis_elegans_Rad21        : NTSTYKEDDGKWAKRAKHILKKVSADIETSG-----------------------QADFSSVTATA--KNRKQAAEQFYSLLTLAKS--------QAISVD :  599
Schizosaccharomyces_pombe_Rad21     : SQTAKDSLRNKWDPYTEGE-----------------------------------KVSFQTLS-AG--CNREEAVQLFFDVLVLATK--------DVISVK :  606
Sordaria_macrospora_Rad21           : --------------HAVHILRDLFGAEAADNADKRKKT----------------AVVFQDLLPEGR-TTKADATKMFFECLVLATK--------DAIKVE :  597
Neurospora_crassa_Rad21             : --------------HAVHILRDLFGAEAANNADKRKKT----------------AVVFQDLLPEGR-TTKADATKMFFECLVLATK--------DAIKVE :  597
Aspergillus_niger_Rad21             : ADDTIVADSGPVSIGTKHAVHILRERLGESAAEQKK------------------SVKFQDLL-PEKKASKADATKMFFEVLVLATK--------DAVQVE :  549
Thalassiosira_pseudonana_Rad21      : LGDELVSHTTKWHKHTVRVFGMLKRNMKSRNADEADAEEEE-------KQA---QLSYNKLS-QG--CSRRTASGVFFEMLQLKTW--------DFVELS :  720
Phaeodactylum_tricornutum_Rad21     : GGDSLVSSNSKWHKHTISVFNMLKKNIASENVDDQNSVGEPKTT----------ELSYTKLS-RG--VDRRTAAGVFLELLQLKTW--------DYIELN :  640
Fragilariopsis_cylindrus_Rad21      : ALGDVASSTTKWHKHTVRVFQHLKKCMKDPNQEHDDGDASTEDADDEPKKDLPEQISFKNLT-KNV-VSRRNAASVFFEMLQLKTW--------DFIDLD :  696
Pseudo-nitzschia_multiseries_Rad21  : AIGDVASSSTKWHKHTIRVFSHLKKCLRDSNNNNSNADADT-------DADLPDTVRFRELT-RNV-VSRRNASSVFFEMLQLKTW--------DFIELE :  515
Pseudo-nitzschia_multistriata_Rad21 : AIGDAASSSTKWHKHTVRVFRHLRKCLRDPNADTDEDGGIA-------NEGLPDSVGFADLT-KNV-VSRRNASSVFFEMLQLKTW--------DFIELE :  730
Seminavis_robusta_Rad21             : AGTELVSSNSKWHKHTVRVLQLLQRTMGDKGGTKTSSGEDKKD-----------HLVFGDLS-KN--CSRRTAAGVFFEILQLKTW--------DFVEVD :  861
                                                                                                              a   F   l 6
```

```
                                                *      1420
Arabidopsis_thaliana_Rec8_(SYN1)    : ---Q-AEPYGDILIARGPN-------M :  617
Oryza_sativa_Rec8                   : LFPE-LDKWATPSLLSHRQ-------M :  679
Oryza_sativa_Rad21-4/Rec8           : ---Q-LEPYGDILISRGPK-------M :  608
Homo_sapiens_Rec8                   : ---Q-EKPYGRLLIQPGPR-------F :  546
Mus_musculus_Rec8                   : ---Q-QKPYGPLLIRPGPK-------F :  590
Schizosaccharomyces_pombe_Rec8      : ---Q-DKPYSEISVSLNLK-------S :  557
Sordaria_macrospora_Rec8            : QDMEDFQPFGEIRVGVGVSEAEMLAMM :  757
Neurospora_crassa_Rec8              : QDMEGFQPFGEIRVGVEVSEAEMLALM :  757
Arabidopsis_thaliana_Rad21          : ---Q-NHPYSDVFLMRVSR-------P :  805
Oryza_sativa_Rad21                  : ---R-PNSFDFVSVKPGPK-------L : 1050
Ricinus_communis_Rad21              : ---E-TDAYSSAILSSFVA-------T :  766
Theobroma_cacao_Rad21               : ---Q-DDANGDITLKLTPT-------L :  672
Zea_mays_Rad21                      : ---Q-PNPFDLVRIKPGSK-------L : 1171
Arabidopsis_thaliana_Rad21-3        : ---Q-DRPYGDIALKLMPA-------L :  687
Arabidopsis_thaliana_Rad21-4        : ---Q-GKPYESIIIKPRPK-------L : 1026
Oryza_sativa_Rad21-2                : ---Q-EQTYGDIIVSATPR-------L :  739
Oryza_sativa_Rad21-3                : ---Q-EAAYG---------------- :  713
Homo_sapiens_Rad21                  : ---Q-EEPYSDIIATPGPR-------F :  628
Mus_musculus_Rad21                  : ---Q-EEPYSDIIATPGPR-------F :  632
Danio_rerio_Rad21                   : ---Q-TEPYSDIIAAPGPR-------F :  640
Xenopus_(Silurana)_tropicalis_Rad21 : ---Q-AEPYSDIIATPGPR-------F :  628
Drosophila_melanogaster_Rad21       : ---Q-SAPYADITITRGPT-------F :  710
Caenorhabditis_elegans_Rad21        : ---Q-SEPYGEIVIRPGAN-------F :  615
Schizosaccharomyces_pombe_Rad21     : ---Q-DVAIQNEITLTAKR-----GML :  624
Sordaria_macrospora_Rad21           : ---QKEGALGGHIRIRGKR--GLWGDW :  619
Neurospora_crassa_Rad21             : ---QKEGALGGHIRIRGKR--GLWGDW :  619
Aspergillus_niger_Rad21             : ---QRPETVGGPLKIRGKR--ALWGSW :  571
Thalassiosira_pseudonana_Rad21      : ---Q-DKSYGDITITPGLR-------F :  736
Phaeodactylum_tricornutum_Rad21     : ---Q-NESYGDIRISAGIK-------F :  656
Fragilariopsis_cylindrus_Rad21      : ---Q-EDSYGDITVSPGIR-------F :  712
Pseudo-nitzschia_multiseries_Rad21  : ---Q-EEAYGDIFISPGLR-------F :  531
Pseudo-nitzschia_multistriata_Rad21 : ---Q-ERAYGDIAISAGLR-------F :  746
Seminavis_robusta_Rad21             : ---Q-DEAYGDIRLLPGNR-------F :  877
```
