# Supplementary material for: Identification of the meiotic toolkit in diatoms and exploration of meiosis-specific SPO11 and RAD51 homologs in the sexual species Pseudo-nitzschia multistriata and Seminavis robusta
Source: BMC Genomics. 2015 Nov 14;16:930. doi: 10.1186/s12864-015-1983-5 (PMC4647503; doi:10.1186/s12864-015-1983-5)
Supplement: Additional file 3: — Multiple sequence alignment of Spo11 proteins used in building the phylogenetic tree of Spo11, shown in Fig. 2 . (PDF 553 kb) [file 12864_2015_1983_MOESM3_ESM.pdf]

Additional File 3. Multiple sequence alignment of Spo11 proteins used in Spo11 phylogenetic tree analysis (in figure 2).

```
                                                              *        20         *        40         *        60         *        80         *       100
Arabidopsis_thaliana_Spo11-2                    : ARARIEVSVLN-LLRILNSPDPAIS-------DLSLINRKRSNSCINKGIL--TDVSYIFLSTS-----------------FTKSSLTN--AKTAK--A :  68
Oryza_sativa_Spo11-2                            : VRARIEVAVLN-FLAALTDPAAPAI------SALPLISRGAANRGLRRALLR-DDVSSVYLSYA-----------------SCKRSLTR--ANDAK--A :  70
Zea_mays_Spo11-2                                : VRARIEVAVLN-FLAALASPSSPAI------SVLPLISRSSANCSLRSGLL--NDVSSVYLSYT-----------------FCKRSL----THNPK--A :  67
Thalassiosira_pseudonana_Spo11-2                : ------------------------------------------------------------------------------------------RTDR--T :   5
Phaeodactylum_tricornutum_Spo11-2               : VIARIERLIET-VVLALERGRMPIL-------------ETLWIPLENG-----SGGEDAVLGAQ-----------------QGDILRKTFHLHQCR--S :  61
Fragilariopsis_cylindrus_Spo11-2                : VLEKIEELILQ-TVIEPLIEEEPGP-------FLPE-----------------IDVDNPKT--------------------HFKRKLSFHHLTQAR--S :  52
Pseudo-nitzschia_multiseries_Spo11-2            : ---------------------------------------------------------------------------------------------------- :   -
Pseudo-nitzschia_multistriata_Spo11-2           : VVEXIEDFILQTVVEPLSRDPNPSV---------------------------PEINXTVDAI-------------------GFKRKLSLHHLTQAR--S :  51
Seminavis_robusta_Spo11-2                       : VIRRVESLTRT-LVQALANGELPEM-------------KSRSNQ---------------------------------------RRRFIFHNMTQCR--A :  44
Ectocarpus_siliculosus_Spo11-2                  : ---------------------------------------------------------------------------------------------------- :   -
Homo_sapiens_Spo11                              : VLASIENIIQD-IITSLARNEAPAF-------TIDN--RSSWENIKF------EDSVGLQMVSH-----------------CTTRKIKSDSPKSAQ--K :  64
Mus_musculus_Spo11                              : VLTAIENIIQD-IIKSLARNEVPAF-------TIDN--RSSWENIMF------DDSVGLRMIPQ-----------------CTTRKIRSDSPKSVK--K :  64
Xenopus_(Silurana)_tropicalis_Spo11             : VLAAIERIIQG-ITESLSKNEAPVL-------TLDN--RTNWANIEF------NHLVGLQIAKH-----------------CTTRKVRSNCAKSAQ--K :  64
Danio_rerio_Spo11                               : VLTRIEKVIER-IVIDVSKGEAPVL-------RLIN--RSCWNNVRF------QESVGLVLKDD-----------------VSPKYLHCGKPTTAT--K :  64
Saccharomyces_cereviceae_Spo11                  : VLAHIKHFLSL-AANSLEQHQQPIS-------IVFQNKKKKGDT---------SSPDIHTTLDF-----------------PLNGPHLCTHQFKLK--R :  63
Schizosaccharomyces_pombe_Spo11                 : VRSWIEQFVHD-FVEQLSKPTKDSV-------NVALKRRKH------------NSWNGSLDSKA-----------------NERQKVKVFSFPRNET-T :  61
Cryptococcus_neoformans_Spo11                   : ALKFLQSIVLS-FLEQISTSLGTIFSHETSKVHKKELKGLKRRKIYAAEAEQKKETDQCLETGKIGVTISLKNRKSGSYQAIMLPDSPLQSPDRQSSIMR :  99
Candida_albicans_Spo11                          : ------MNYHE-VTIIIKNSSNNSL--------------------------------------------------------RKLGSYCVLSIRETETRR :  36
Kluyveromyces_lactis_Spo11                      : CQRQITNNMRF-IRLALKEHAIPIT-------------VVFNGSITK------RKRCEEGSGGK-----------------LLRWRYPEFANTKKKCSK :  62
Neurospora_crassa_Spo11                         : ALTKIEALFET-IVDAISVGSPITI----------PYRRSTNAQVFDPT----ASASNPRDGRH-----------------MDSVRFPGRNPQELR--R :  65
Arabidopsis_thaliana_Spo11-1                    : LLQRIKDFTQS-VVVDLAEGRSPKI-------SINQFRNYCMN----------PEADCLCSSDK-----------------PKGQEIFTL-KKEPQTYR :  63
Oryza_sativa_Spo11-1                            : LLHRIRGLVRW-VVAEVAAGRSPTV-------ALHRYQNYCSSASAA------AASPCACSYDV-----------------PVGTDVLSLLHRGSHASR :  68
Zea_mays_Spo11-1                                : LLRRIKGLVRW-IVEEVAAGRSPSI-------VLHRYRNYCSSADSA------SPSPCACSYDI-----------------PVGTDVLSLLHKDYHTSR :  68
Arabidopsis_thaliana_Spo11-3/Top_VIA            : VQTEIETVIVQ-IARSILAGDGFSF-------SVPS--RAASNQLYV------PELDRIVLKDK-----------------STLRPFAS--VSSVR--K :  62
Oryza_sativa_Spo11-3/Top_VIA                    : VTSSINRLVLA-AARSILSGRGFSF-------AVPS--RAASNQVYL------PDLNRIVLVRR-----------------ESARPFAN--VATAR--K :  62
Zea_mays_Spo11-3/Top_VIA                        : VTSRINRLVLA-AARSILSGRGFSF-------AVPS--RAASNQVYL------PDLDRIVLVRR-----------------ESARPFAN--VATAR--K :  62
Thalassiosira_pseudonana_Spo11-3/Top_VIA        : VMEGIENVAVK-IAKQVLAKQGFQL-------EIPS--RSASNQVYV------PELDRIVLGDK-----------------TGTRSFLN--VKESR--K :  62
Phaeodactylum_tricornutum_Spo11-3/Top_VIA       : VLEGMEAMALH-IAQQVLERKGFSL-------EIPS--RAASNQIYV------KEWDRIVLGGK-----------------RSSRSFLN--VKESR--K :  62
Fragilariopsis_cylindrus_Spo11-3/Top_VIA        : VLEGIEGVALQ-ITRQVLQKKGFSM-------EIPS--RASSNQIYL------KDRDRLVLGGK-----------------RVGRSFLN--VRESR--K :  62
Pseudo-nitzschia_multiseries_Spo11-3/Top_VIA    : VLEGIEGVALQ-ITRQVLAKKGFSM-------EIPS--RSASNQIYV------KEWDRIVLGGK-----------------RTGRNFMN--VRESR--K :  62
Pseudo-nitzschia_multistriata_Spo11-3/Top_VIA   : VLEGIEGVALR-ITRQVLAKKGFSL-------EIPS--RSASNQIYV------REWDRIVLGGK-----------------RIGRSFTN--VRESR--K :  62
Seminavis_robusta_Spo11-3/Top_VIA               : VLEGLETIALR-VTNQVLTKQGFSM-------AVPS--RGPGNQIYV------AEWDRIVLGNK-----------------HLSRNFLS--VKDTR--K :  62
Ectocarpus_siliculosus_Spo11-3/Top_VIA          : VMDEIERIAVE-TALSIMEGNGFGF-------RVPT--RSSGNQKYV------EELDRIVLGDK-----------------VSSRNFVS--TAMAR--K :  62
Thermococcus_gammatolerans_Top_VI               : VLSKLEEYGRS-VLEAIKSGKNPYF-------DIPM--RGLNNVYFD------EKSRLIKMGDK-----------------LSRRYFLN--VAHAR--K :  62
Sulfolobus_acidocaldarius_Top_VI                : AGNVLRETFLK-LVEMVNNNEPPIM-------EIPK--RTLSNTVYD------EKRGLLILGKE-----------------KLKRNFLD--LNEAK--R :  62
```

```
                                                              *       120         *       140         *       160         *       180         *       200
Arabidopsis_thaliana_Spo11-2                    : FVRVWKVMEICFQILLQ-----------------------------------------------EKRVTQ-RELFYKLLCDSP-----------DYFSS-QIEV : 112
Oryza_sativa_Spo11-2                            : FVRVWKVMEMCYKILGE-----------------------------------------------GKLVTL-RELFYTLLSESP-----------TYFTC-QRHV : 114
Zea_mays_Spo11-2                                : FVRVWKVMEMCYKILGE-----------------------------------------------GKLVQQ-RELFYKLLSDSP-----------KYFSC-QRHV : 111
Thalassiosira_pseudonana_Spo11-2                : FTSITKVMAFIHQLLLS-----------------------------------------------NRTTTT-REVYYV----FV-----------THFRN-QKEC :  45
Phaeodactylum_tricornutum_Spo11-2               : FTSILLVLDFCHSLLRA-----------------------------------------------RRTTTT-REVYYY----HV-----------THYRS-QKEC : 101
Fragilariopsis_cylindrus_Spo11-2                : LTSILMVASFCHDLLAPSSSSSVMNNEDDNNEEDSDDDDDVDIEDGNYYYHRRRQNGRRRRRTTTT-REVYYF----FV-----------THFRD-QREC : 135
Pseudo-nitzschia_multiseries_Spo11-2            : --------------LQIP-----------------------------------------------QRTTTT-REVYYF----YV-----------TYFRD-QREC :  27
Pseudo-nitzschia_multistriata_Spo11-2           : LTSILKVAAFCYDLLAP---------------EDKEEHEDGHERDAANGKGDGCRVXYRTRKTTTT-REVYYF----YV-----------TYFRD-QREC : 119
Seminavis_robusta_Spo11-2                       : FSNILLVLAFCHNLLLS-----------------------------------------------QRTTTT-REVYYF----YV-----------THFRS-QREC :  84
Ectocarpus_siliculosus_Spo11-2                  : -------MSFSMRLLET-----------------------------------------------RKTATQ-REIYYT----FV-----------KHFAN-QTKC :  33
Homo_sapiens_Spo11                              : FSLILKILSMIYKLVQS-----------------------------------------------NTYATK-RDIYYT----DS-----------QLFGN-QTVV : 104
Mus_musculus_Spo11                              : FALILKVLSMIYKLIQS-----------------------------------------------DTYATK-RDIYYT----DS-----------QLFGN-QAAV : 104
Xenopus_(Silurana)_tropicalis_Spo11             : FALMLKVLSVIYKLVQT-----------------------------------------------DTYTTK-RDIYYN----DV-----------QLYGS-QTVV : 104
Danio_rerio_Spo11                               : FALILKILSIIYKLVQS-----------------------------------------------DSYATK-RDIYYN----DP-----------QLFGS-QRML : 104
Saccharomyces_cereviceae_Spo11                  : CAILLNLLKVVMEKLPL-----------------------------------------------GKNTTV-RDIFYS----NV-----------ELFQR-QANV : 103
Schizosaccharomyces_pombe_Spo11                 : IAQLFRVLDCVHEAVIS-----------------------------------------------DTVITK-RDIYYR----DV-----------DLFKR-QTVV : 101
Cryptococcus_neoformans_Spo11                   : MTCIMRVASTLYEAILD-----------------------------------------------RNVITL-RDVYYR----DK-----------QLFER-QPVV : 139
Candida_albicans_Spo11                          : FAAVCKLISILIKHLKA-----------------------------------------------QNQITTIRDIYYQ----DV-----------EVFNHCQNEC :  78
Kluyveromyces_lactis_Spo11                      : VPQLLQLLHILNERIKT-----------------------------------------------KKNTTTVRDIYYG----NI-----------ELFES-QQMV : 103
Neurospora_crassa_Spo11                         : FEALFRIIEISHEALLS-----------------------------------------------GTLVTK-R----------------------------S-QSVV :  94
Arabidopsis_thaliana_Spo11-1                    : IDMLLRVLLIVQQLLQE-----------------------------------------------NRHASK-RDIYYM----HP-----------SAFKA-QSIV : 103
Oryza_sativa_Spo11-1                            : LNVLLRVLLVVQQLLQQ-----------------------------------------------NKHCSK-RDIYYM----YP-----------SIFQE-QAVV : 108
Zea_mays_Spo11-1                                : LNVLLRVLFVVQHLLQQ-----------------------------------------------NKHCSK-RDIYYM----YP-----------SIFVE-VAVV : 108
Arabidopsis_thaliana_Spo11-3/Top_VIA            : TTITTRILALIHQLCLR-----------------------------------------------NIHVTK-RDLFYT----DV-----------KLFQD-QTQS : 102
Oryza_sativa_Spo11-3/Top_VIA                    : ATITARVLFLVHAVLRR-----------------------------------------------GIHVTK-RDLFYT----DV-----------KLFGD-QAQS : 102
Zea_mays_Spo11-3/Top_VIA                        : ATITARVLSLVHAVLGR-----------------------------------------------GIHVTK-RDLFYT----DV-----------KLFGD-QTQS : 102
Thalassiosira_pseudonana_Spo11-3/Top_VIA        : SAITTRVLQLLHAVLLK-----------------------------------------------RIHITK-RDLFYT----DV-----------KLFVD-QSES : 102
Phaeodactylum_tricornutum_Spo11-3/Top_VIA       : SAITLRVMQLLHAVLMK-----------------------------------------------RIHITK-RDLFYT----DV-----------KLFVD-QAES : 102
Fragilariopsis_cylindrus_Spo11-3/Top_VIA        : SAITLRVMQLLHAVLVK-----------------------------------------------RIHITK-RDLFYT----DV-----------KLFVD-QAES : 102
Pseudo-nitzschia_multiseries_Spo11-3/Top_VIA    : SAITLRVMQLIHAVLVK-----------------------------------------------RIHITK-RDLFYT----DV-----------KLFVD-QAES : 102
Pseudo-nitzschia_multistriata_Spo11-3/Top_VIA   : SAITLRVMQLLHAVLVK-----------------------------------------------RIHITK-RDLFYT----DV-----------KLFVD-QAES : 102
Seminavis_robusta_Spo11-3/Top_VIA               : TAVTVRVMQLLHAVLSK-----------------------------------------------RIHITK-RDLFYT----DV-----------KLFVN-QMDS : 102
Ectocarpus_siliculosus_Spo11-3/Top_VIA          : TAITTRVVQLVHQVLGK-----------------------------------------------GIHITK-RDMFYT----DV-----------KLFKE-QSES : 102
Thermococcus_gammatolerans_Top_VI               : FMQTLLIMAYVKRLVSE-----------------------------------------------GKHASL-REAYYANKHTIP-------GTRENTFED-QRES : 110
Sulfolobus_acidocaldarius_Top_VI                : FMQTTLMASIIYDALVN-----------------------------------------------DEYPTI-RDLYYRGKHSIILKDPNGRAYEENTWDE-QKES : 117
                                                                                                                        R  y                        q
```

```
                                              *       220          *        240          *        260          *        280          *        300
Arabidopsis_thaliana_Spo11-2                : NRSVQDVVAL-LRCS-RYSLGIMASSRGLVAGR--LFLQE-----PGKEAVD---CSACGSSGFAITGDLNL---------------------------- : 172
Oryza_sativa_Spo11-2                        : NQTVQDVVSL-LRCT-RQSLGIMASSRGALIGR--LVVQG-----PEEEHVD---CSILGPSGHAITGDLNV---------------------------- : 174
Zea_mays_Spo11-2                            : NQAIQDVVSL-LRCT-RQSLGVMASSRGALIGR--LVLHE-----PDGEQID---CSILGASGHAITGDLNL---------------------------- : 171
Thalassiosira_pseudonana_Spo11-2            : DGTILDVAKI-LNVP-RRALGLSASPKGELEGR--SVYME----------IS---CLVNGFADDGVEIKVTSK--------------------------- : 101
Phaeodactylum_tricornutum_Spo11-2           : DSAIQDTAIL-LQVP-RSSLGLKASPKGWFCGD--VQLVS-----NGQVVLD---GRHLQSIHGAPISGEWLAP-------------------------- : 163
Fragilariopsis_cylindrus_Spo11-2            : DKAIWDIVSI-LNLPSRQSLGLVASPKGWFCGS--IDVYN-----GHTNELKFNGRELDTHGMAITPSTYDNDYTTYNNSKPTNNDRDPMDDDDNNGNGN : 227
Pseudo-nitzschia_multiseries_Spo11-2        : DKAIWDLVCI-LGLPSRQSLGLVASPKGWFCGS--IDVYN-----AHTGELN-----------NNISSNHIR---------------------------- :  80
Pseudo-nitzschia_multistriata_Spo11-2       : DKAIWDLVCI-LGLPSRQSLXLVASPKGWFCGS--IDVYN-----ARTGELRFNGRDLDAHGMAITPSTYDSYSSDFNFSYLCKDDKXA----DDPLPIQ : 207
Seminavis_robusta_Spo11-2                   : DAAIVEAANI-LRVP-RSTLGLFASPKGWFCGC--IQILR-----EGRIAMD--GKALSSIQGAPITQEWLSSPTQRDFTIDCLVDAAQAENEDDSSTSN : 173
Ectocarpus_siliculosus_Spo11-2              : NDAILDCCSL-LGEP-RHCLGIVASSRGYLAGR--ISIRG-----EGFEDWQ-DPRSTGGSSGLAITSDWL----------------------------- :  94
Homo_sapiens_Spo11                          : DNIINDISCM-LKVS-RRSLHILSTSKGLIAGN--LRYIE-----EDGTKVN---CT-CGATAVAVPSNIQG---------------------------- : 163
Mus_musculus_Spo11                          : DSAIDDISCM-LKVP-RRSLHVLSTSKGLIAGN--LRYME-----EDGTRVQ---CT-CSATATAVPTNIQG---------------------------- : 163
Xenopus_(Silurana)_tropicalis_Spo11         : DNIVSDISCM-LKIP-RINLHILSTSKGCVAGD--LWFTA-----EDGSKVY---CG-GSSSGVLVPINVEG---------------------------- : 163
Danio_rerio_Spo11                           : DSIVDDISCM-LKVP-RRSLHILATSKGFISGD--LCYLE-----EDGTKVD---CN-SSSTAVSVSSNVNG---------------------------- : 163
Saccharomyces_cereviceae_Spo11              : VQWLDVIRFN-FKLSPRKSLNIIPAQKGLVYSPFPIDIYDNILTCENEPKMQ---KQTIFPGKPCLIPFFQD---------------------------- : 171
Schizosaccharomyces_pombe_Spo11             : DELLGDISNT-IGCS-RSDLNVEASAKGLVFGS--IHIAL-----ENGTVIT---------ATKPLISHHR----------------------------- : 155
Cryptococcus_neoformans_Spo11               : DKIVDDLVAT-AGLR-RRDFYVCASAKGLIASS-SLVIRY-----RTGDEII------LSATRANIVDPAEK---------------------------- : 197
Candida_albicans_Spo11                      : RFLLGQLVERGIGWSLCDDLNIHPTQKGLVYGD----YFQ--------------ELSIKAEPILIPINYTKFFNTK------------------------ : 136
Kluyveromyces_lactis_Spo11                  : EMLFQRIEAM-FNVE-KLFFNVVSTQKGLIYSG--PRLQI------------------GSQLFHAECQLVPYDQG-------------------------- : 156
Neurospora_crassa_Spo11                     : DDMVDNLAFT-LGVG-RNDLNIVAIAKGLVAGQ--VELIM-----RGGSKID---CAESSD---------------------------------------- : 143
Arabidopsis_thaliana_Spo11-1                : DRAIGDICIL-FQCS-RYNLNVVSVGNGLVMGW--LKF-R-----EAGRKFD---CLNSLNTAYPVPVLVEE----------------------------- : 162
Oryza_sativa_Spo11-1                        : DRAINDICVL-FKCS-RHNLNVVPVAKGLVMGW--IRF-L-----EGEKEVY---CVTNVNAAFSIPVSIEA----------------------------- : 167
Zea_mays_Spo11-1                            : DRAINDICIL-FKCS-RHNLNVVPVVKGLVMGW--IRFM------EGEKKVY---CITSVNAAFSIPVDIEA----------------------------- : 167
Arabidopsis_thaliana_Spo11-3/Top_VIA        : DAVLDDVSCM-LGCT-RSSLNVIAAEKGVVVGR--LIF-S-----DNGDMID---CTKMGMGGKAIPPNIDR----------------------------- : 161
Oryza_sativa_Spo11-3/Top_VIA                : DAVLDDVSCM-LGCT-RSSLHVVAASEKGVVVGR--LTF-A-----DDGDRID---CTRMGVGGKAIPPNIDR----------------------------- : 161
Zea_mays_Spo11-3/Top_VIA                    : DAVLDDVSCM-LGCT-RSSLHVVASEKGVVVGR--LVF-A-----DDGDRID---CTRMGVGGKAIPPNVDR----------------------------- : 161
Thalassiosira_pseudonana_Spo11-3/Top_VIA    : DGVLDDVATM-LGCT-RSNLHVVASDKGLVVGR--ISF-F-----EDGDFID---CTKMGVGGKAIPPYIDK----------------------------- : 161
Phaeodactylum_tricornutum_Spo11-3/Top_VIA   : DGVLDDVATM-LGCT-RSNLHVVASDKGLVVGR--IQF-E-----EDGDFID---CTKMGVGGKAIPPYIDK----------------------------- : 161
Fragilariopsis_cylindrus_Spo11-3/Top_VIA    : DGVLDDVATM-LGCT-RSNLHVVASDKGLVVGR--IQF-E-----EDGDFID---CTKMGVGGKAIPPYIDK----------------------------- : 161
Pseudo-nitzschia_multiseries_Spo11-3/Top_VIA : DGVLDDVATM-LGCT-RSNLHVVASDKGLVVGR--IQF-E-----EDGDFID---CTKMGVGGKAIPPYIDK----------------------------- : 161
Pseudo-nitzschia_multistriata_Spo11-3/Top_VIA : DGVLDDVATM-LGCT-RSNLHVVASDKGLVVGR--IQF-E-----EDGDFID---CTKMGVGGKAIPPYIDK----------------------------- : 161
Seminavis_robusta_Spo11-3/Top_VIA           : DGVLDDVATM-LGCT-RSNLHVVASDKGLVVGR--IQF-E-----EDGDYID---CTRMGVSGKAIAPYIDK----------------------------- : 161
Ectocarpus_siliculosus_Spo11-3/Top_VIA      : DNVLDDVACM-VGCT-RSCLNVVASDKGLVVGR--VTF-M-----EDGDLID---CTRMGVGGKAIPPYIDK----------------------------- : 161
Thermococcus_gammatolerans_Top_VI           : DPIIEDLERM-LGVL-REEMHITADRRGYIYGD--IVI-R-----DGEDEFN---ASKLGSGGWAVPGTVEH----------------------------- : 169
Sulfolobus_acidocaldarius_Top_VI            : DSVIVDIEVF-TSLL-REDMLILSKEKGKVVGD--MRI-R-----SGNDVID---LSKMGHGAYSIEPTPDL----------------------------- : 176
                                                                   6     G  g
```

```
                                              *       320          *        340          *        360          *        380          *        400
Arabidopsis_thaliana_Spo11-2                : -LDNTIMRT-DARYIIVVEKHAIFH----RIVE-DRVFNHI----------PCVFITAKGYPDIATRFFLHRMSTTF--PDLPILVLVDWNPAGLAILC : 252
Oryza_sativa_Spo11-2                        : -LSKLIFSS-DARYIIVVEKDAIFQ----RIAE-DRIYSHL----------PCILITAKGYPDIATRFILHRLSQTY--PNMPIFALVDWNPAGLAILC : 254
Zea_mays_Spo11-2                            : -LSKLNLSS-GSRYIIVVEKDAVFQ----RIAE-DRLYNQL----------PCILITAKGYPDIATRFILHRLSQTF--PNMPIFALVDWNPAGLAILC : 251
Thalassiosira_pseudonana_Spo11-2            : ----------DAKVIVVIEKEGVYN----KISE-ERIFDDF----------PCILVVTGKGFPDISTRALVNTLHKEL---DIPVVGICDSNPFGISVIA : 172
Phaeodactylum_tricornutum_Spo11-2           : -TRDFTIHSCAATCILVIEKEGVYN----RIVE-DRFFDRF----------PCILVTGKGFPDLSTRALVHVLHHTL--GLLPVRGLCDCNPYGVMVLH : 244
Fragilariopsis_cylindrus_Spo11-2            : GNDNIRIES-DARCILVIEKEGVYT----RISE-DKFFLNY---------LPCILVTGKGFPDIATRRWVRRMQKTL---KIPVYGLCDCNEYGVSVLD : 308
Pseudo-nitzschia_multiseries_Spo11-2        : -IES------DAKCILVIEKEGVYT----RISE-DKFFLRY---------FPCILVTGKGFPDIATRRWVKKMQKTL---NIPVYGLCDCNPFGVSVLD : 155
Pseudo-nitzschia_multistriata_Spo11-2       : SGSGIRIES-DAKCILVIEKEGVYT----RISE-DKFFLRY---------FPCILVTGKGFPDVATRRWVKRMQKTL---KIPVYGLCDCNPFGVSVLD : 288
Seminavis_robusta_Spo11-2                   : TQPAERR---RAKCVLVIEKEGVYT----RIAE-DRIFEKY----------PCILVTGKGFPDVATRSFVHTLHREL---KIPVYGLADCNEYGALVIN : 251
Ectocarpus_siliculosus_Spo11-2              : -TRPLELKS-DARIVLVVEKDGVEN----RMVE-DGFYDRANVSTLHYLRRIPSVLVTGQGFPPLAVRACVYRLAEAL---DLPVYGLCDCNPFGLSVFL : 184
Homo_sapiens_Spo11                          : -IRNLVT---DAKFVLIVEKDATFQ----RILD-DNFCNKL----------SPCIMITGKGVPDINTRLLVKKLWDTF---HVPVFTLVDADPHGIEIMC : 241
Mus_musculus_Spo11                          : -MQHLIT---DAKFVLIVEKDATFQ----RILD-DNFCSRM----------SPCIMVTGKGVPDINTRLLVKKLWDTF---HIPVFTLVDADPYGIEIMC : 241
Xenopus_(Silurana)_tropicalis_Spo11         : -IRHLST---QAKFILIIEKDATFQ----RILD-DNFCARS----------GPCIIITGKGVPDINTRLLTRKLWDSF---HIPIFTLVDADPYGTK--- : 238
Danio_rerio_Spo11                           : -IRNIVS---SAKFILVVEKDATFQ----RILD-DDFCARL----------SPCIIITGKGVPDVNSRLMVRKLWDAL---HVPVFALVDADPHGIEIMC : 241
Saccharomyces_cereviceae_Spo11              : -DAVIKLGTTSMCNIVIVEKEAVFT----KLVN-NYHKLST----------NTMLITGKGFPDFLTRLFLKKLEQYCSKLISDCSIFTDADPYGISIAL : 254
Schizosaccharomyces_pombe_Spo11             : -ISSITS---TAKWVLVIEKEAVFQ----TLTE-EALA-------------DTIIVTAKGFPDIMTRKFLVKLAKAL--PDAKFFGIFDWDPHGLCIYS : 230
Cryptococcus_neoformans_Spo11               : -IDVIEAPN-GLDWVLVIEKDAIFQ----SLCG-AGILQDE--------RIGHGVLITGKGFPDLATRQIIRLIADTF--PCVKMYALVDADPHGIEIMC : 280
Candida_albicans_Spo11                      : -ITQIKV---EKVIVVILEKDAVFQCLCTHLRQ-HNIINRF-------------LIVTAKGYSDNLTLRFLTWLQTNE---NCKFIGFFDSDVYGINIYW : 215
Kluyveromyces_lactis_Spo11                  : ---EMIVQQNQQLKVIVLEKDAIFQ----QLIQCQEMIKDW------------IIVTGKGYPDHLSRELLHKIDLSL-QGNVQFHIFTDSDPYGIDIVT : 235
Neurospora_crassa_Spo11                     : ---------------------------------------------------------SGKGYPDLATRKFLAAIHSVR--PRLLMFALVDFDPHGISVIR : 184
Arabidopsis_thaliana_Spo11-1                : -VEDIVS---LAEYILVVEKETVFQ----RLAN-DMFCKTN----------RCIVITGRGYPDVSTRRFLRLMMEKL---HLPVHCLVDCDPYGFEILA : 239
Oryza_sativa_Spo11-1                        : -IKDVVS---VADYILVVEKETVFQ----RLAN-DKFCERN----------RCIVITGRGYPDIPTRRFLRYLVEQL---HLPVYCLVDADPYGFDILA : 244
Zea_mays_Spo11-1                            : -IKDVVS---VAHYILVVEKETVFQ----RLAN-DKFCERN----------RCIVITGRGYPDIPTRRFLRYLVELL---HLPAYCLVDADPYGFDILA : 244
Arabidopsis_thaliana_Spo11-3/Top_VIA        : -VGDMQS---DAMFILLVEKDAAYM----RLAE-DRFYNRF----------PCIIVTAKGQPDVATRLFLRRLKKMEL---KLPVLALVDSDPYGLKILS : 238
Oryza_sativa_Spo11-3/Top_VIA                : -VSGIES---DALFILLVEKDAAFM----RLAE-DRFYNRF----------PCIILTAKGQPDVATRLFLRRLKVEL---KLPVLALVDSDPYGLKILS : 238
Zea_mays_Spo11-3/Top_VIA                    : -VSGIES---DALFILLVEKDAAFM----RLAE-DRFYNRF----------PCIILTAKGQPDVATRLFLRRLKVEL---KLPVLALVDSDPYGLKILS : 238
Thalassiosira_pseudonana_Spo11-3/Top_VIA    : -IENIQS---DAEFILLVEKEAAYM----RMAE-DRFYQRY----------PCIVITAKGQPDVATRMFLSRITSEL---QIPVLALVDSDPYGLKILS : 238
Phaeodactylum_tricornutum_Spo11-3/Top_VIA   : -IENIAS---DAEFILLVEKEAAYM----RMAE-DRFYHRY----------PCIIITAKGQPDVATRMFLARITSEL---KIPVLGLVDSDPYGLKILS : 238
Fragilariopsis_cylindrus_Spo11-3/Top_VIA    : -IENIRS---DAEFILLVEKEAAYM----RMAE-DRFYNKY----------PCIVITAKGQPDVATRMFLSRITNEL---QIPVLGLVDSDPYGLKILS : 238
Pseudo-nitzschia_multiseries_Spo11-3/Top_VIA : -IENITS---DAEFILLVEKEAAYM----RMAE-DRFYHKY----------PCIVITAKGQPDVASRMFLSRITNQL---KIPVLGLVDSDPYGLKILS : 238
Pseudo-nitzschia_multistriata_Spo11-3/Top_VIA : -IENIES---DAEFILLVEKEAAYM----RMAE-DRFYHKY----------PCIVITAKGQPDVASRMFLSRITHEL---KIPVLGLVDSDPYGLKILS : 238
Seminavis_robusta_Spo11-3/Top_VIA           : -IENIVS---DAEFILLVEKEAAYM----RIAE-DRFYNKY----------PCIIITAKGQPDVATRMFLSRAASQL---QIPVLGLVDSDPYGLKILS : 238
Ectocarpus_siliculosus_Spo11-3/Top_VIA      : -ITNIRS---DADFILLVEKEAAYM----RMAE-DRFYQKY----------PCIVITAKGQPDVATRLFLKRLKREL---NIPVMGLVDSDPYGLKILS : 238
Thermococcus_gammatolerans_Top_VI           : -IQFPEI---NVDYALVVETAAMAD----RLIE-EKFPKKE----------NALIIATQGQASRGVRRLIHRLHYEE---GLPIIVFTDGDPYGWYIYS : 246
Sulfolobus_acidocaldarius_Top_VI            : -IDFVDI---NAEFVLVVEKDAVFQ----QLHR-AGFWKQY----------KAILVTSAGQPDRATRRFVRRLNEEL---KLPVYILTDADPYGWYIYS : 253
                                                       a       ek                             G pd    r               p     D 1p G
```

```
                                                       *       420         *       440         *       460         *       480         *       500
Arabidopsis_thaliana_Spo11-2                    : TFKFG------------------------------SIGMGLE-AYRYAC----NVKWIGLRGDDL--------------------N-------------- : 283
Oryza_sativa_Spo11-2                            : TYKYG------------------------------SISMGLE-SYRYAC----NVKWLGLRGDDL--------------------Q-------------- : 285
Zea_mays_Spo11-2                                : TYKYG------------------------------SISMGLE-SYRYAC----NVKWLGVRGGDL--------------------HL------------- : 283
Thalassiosira_pseudonana_Spo11-2                : LYYCA------------------------------GERMGVDGRMKYTVP----MMWIGLRPSTV--------------------ESL------------ : 206
Phaeodactylum_tricornutum_Spo11-2               : TYQH-------------------------------TARKGVDGGHRFGVP----ISWIGLRPSQV--------------------QQL------------ : 277
Fragilariopsis_cylindrus_Spo11-2                : TYRYDKGVKICNV----------------------EKRRKKK-SDDEDDPL--EIQWIGLRPSQI--------------------EHM------------ : 351
Pseudo-nitzschia_multiseries_Spo11-2            : SYRHD-------------------------------QGNQHQ-SENRSDPLD-DLHWIGLRPSQV--------------------ETM------------ : 190
Pseudo-nitzschia_multistriata_Spo11-2           : SYRHDQGVRLHRKIKSSSXQRKSRSKIRIDEPSASGSETEHD-KTHDSPE---ALHWIGLFPSQI--------------------ETM------------ : 352
Seminavis_robusta_Spo11-2                       : TYQRG------------------------------SERLGMDGGSRYSVP----IRWLGLMPSQV--------------------ADIGSGTATDGDDDM : 297
Ectocarpus_siliculosus_Spo11-2                  : QYKEG------------------------------SSRQGRG-SAKFGVP---EMKWVGLRPSQL--------------------DDL------------ : 218
Homo_sapiens_Spo11                              : IYKYG------------------------------SMSMSFE-AHHLTVP---AIRWLGLLPSDL--------------------KRL------------ : 275
Mus_musculus_Spo11                              : IYKYG------------------------------SMSMSFE-AHNLTIP---TIRWLGLLPSDI--------------------QRL------------ : 275
Xenopus_(Silurana)_tropicalis_Spo11             : ------------------------------------LGDNVQ-KQSSILP---I---------------------------------------------- : 252
Danio_rerio_Spo11                               : IYKYG------------------------------SVSMAFE-AHSLTVP---SVLWLGLLPSDI--------------------QRY------------ : 275
Saccharomyces_cereviceae_Spo11                  : NYTHS------------------------------NERNAYI------CT---MANYKGIRITQV-----------------LAQNNE------------ : 286
Schizosaccharomyces_pombe_Spo11                 : CFKYG------------------------------SNAYSHEPHSQLR-----NLQLLGPLYEDI----------------------------------- : 260
Cryptococcus_neoformans_Spo11                   : TYMYG------------------------------SKANAYS-TDYGDLPLGDRVQWLGLRASHW--------------------SDL------------ : 317
Candida_albicans_Spo11                          : QYNQK------------------------------LPEMVYSGIYLLESQ---PHTWLSIASRDI--------------------AMM------------ : 250
Kluyveromyces_lactis_Spo11                      : KYMVK-------------------------------------------CP---RLIHKGVLLNQL----------------------------------- : 254
Neurospora_crassa_Spo11                         : TYQYG------------------------------SQRLDHE--ERTKVF---GLRWLGIRSSDVLLNTVMTQDCSANSQGSQSSQDLSSQESIAYSVDA : 249
Arabidopsis_thaliana_Spo11-1                    : TYRFG------------------------------SMQMAYD-IESLRAP---DMKWLGAFPSDS--------------------EVY------------ : 273
Oryza_sativa_Spo11-1                            : TYKFG------------------------------SLQLAYD-ANFLRVP---DIRWLGVFTSDF--------------------EDY------------ : 278
Zea_mays_Spo11-1                                : TYKFG------------------------------SLQLAHD-ANLLRVP---DIRWLGVFTSDF--------------------EEY------------ : 278
Arabidopsis_thaliana_Spo11-3/Top_VIA            : VYGCG------------------------------SKNMSYD-SANLTTP---DIKWLGIRPSDL--------------------DKY------------ : 272
Oryza_sativa_Spo11-3/Top_VIA                    : VYMCG------------------------------SKNMSYD-SANLTTP---DIKWPGVRPSDL--------------------DKY------------ : 272
Zea_mays_Spo11-3/Top_VIA                        : VYMCG------------------------------SKNMSYD-SANLTTP---DIKWLGVRPSDL--------------------DKY------------ : 272
Thalassiosira_pseudonana_Spo11-3/Top_VIA        : VYMSG------------------------------SKNMSYD-SASLTTP---DIKWLGLRPSDL--------------------NRY------------ : 272
Phaeodactylum_tricornutum_Spo11-3/Top_VIA       : VYMSG------------------------------SKNMSYD-SASLTTP---DIKWLGLRPSDL--------------------DKY------------ : 272
Fragilariopsis_cylindrus_Spo11-3/Top_VIA        : VYMSG------------------------------SKNMSYD-SASLTTP---NIMWLGLRPTDL--------------------DKY------------ : 272
Pseudo-nitzschia_multiseries_Spo11-3/Top_VIA    : VYMSG------------------------------SKNMSYD-SASLTTP---NIMWLGLRPSDL--------------------DKY------------ : 272
Pseudo-nitzschia_multistriata_Spo11-3/Top_VIA   : VYMSG------------------------------SKNMSYD-SASLTTP---NIMWLGLRPSDL--------------------DKY------------ : 272
Seminavis_robusta_Spo11-3/Top_VIA               : VYMSG------------------------------SKNMSYD-SASLTTP---DIQWLGLRPSDL--------------------DKY------------ : 272
Ectocarpus_siliculosus_Spo11-3/Top_VIA          : VYMSG------------------------------SKNMSYD-SASLTTP---DIKWLGVRPSDL--------------------DRY------------ : 272
Thermococcus_gammatolerans_Top_VI               : TIKQG------------------------------SINLAYL-SDKLATP---EARFVGMTMDDI--------------------KRY------------ : 280
Sulfolobus_acidocaldarius_Top_VI                : VFKIG------------------------------SISLSYE-SERLATP---NARFLGVSMTDI--------------FGDKGKKPY------------ : 293
                                                                                                           g

                                                          *       520         *       540         *       560         *       580         *       600
Arabidopsis_thaliana_Spo11-2                    : -----------------LIPEESL-VPLKPK-----DSQIAKSLLS---------------SKILQEN---------YIEELSLM--------------- : 321
Oryza_sativa_Spo11-2                            : -----------------LIPQSAY-QELKPR-----DLQIAKSLLS---------------SKFLQDK---------HRAELTLM--------------- : 323
Zea_mays_Spo11-2                                : ------------------IPEDAF-QELKPR-----DLQIAKSLMS---------------SKFLQES---------HRAELALM--------------- : 320
Thalassiosira_pseudonana_Spo11-2                : ---------------EDDLPKDVF-QSLTDL-----DYKRIDSLLD---------------EDNLFLN-------EERYEEIVKM--------------- : 248
Phaeodactylum_tricornutum_Spo11-2               : ------QRQPNTKHGQSKLPDQVF-QSLTAL-----DKRRLEHHLL------------SEQHGWTTFG-----PDERRVEELEEM--------------- : 333
Fragilariopsis_cylindrus_Spo11-2                : -----------------NLPSDVF-QELTNN-----DKKRLESLLISSKSKSRTTPKNKNSKSFAERG---GWNKEERVRELQAM--------------- : 410
Pseudo-nitzschia_multiseries_Spo11-2            : -----------------DLPSQVF-QELTTN-----DKKRLQSLLV---------PRSTHSGSFAERG---GRDKAERIRELKAM--------------- : 240
Pseudo-nitzschia_multistriata_Spo11-2           : -----------------DLPXQVF-QELTSN-----DTKRLDSLLA---------PKTPNSDCFVDLGGRN---RSRWIRELKAM--------------- : 402
Seminavis_robusta_Spo11-2                       : MDEEATSRSTGNRSNNPSLPPGVY-QQLTDL-----DRKRLAQLLD---------------EDHQFHG---CGTNQDCYEELELM--------------- : 358
Ectocarpus_siliculosus_Spo11-2                  : -----------------DLPDEVH-QTLSQQ-----DLSRAQSVANSTLVTDNPDTYGEEVEYWLDTE------NPGKGKGCERISPNTSIGKGDLPIVW : 289
Homo_sapiens_Spo11                              : -----------------NVPKDSL-IPLTKR-----DQMKLDSILR---------------RPYVTCQ-------PFWRKEMEIM--------------- : 315
Mus_musculus_Spo11                              : -----------------NIPKDSL-IPLTKH-----DQMKLDSILK---------------RPYITYQ-------PLWKKELEMM--------------- : 315
Xenopus_(Silurana)_tropicalis_Spo11             : ----------------------TL-FPYIFS-----FTFSCLCFRD---------------RNHVHLQ--------VWIY-IHVI--------------- : 285
Danio_rerio_Spo11                               : -----------------RVPEEAL-IPFAKA-----DERKISSLRS---------------RSYITCQ-------PTWEREVETM--------------- : 315
Saccharomyces_cereviceae_Spo11                  : -----------------VHNKSIQLLSLNQR-----DYSLAKNLIA-----------SLTANSWDIAT----SPLKNVIIECQRE--------------- : 334
Schizosaccharomyces_pombe_Spo11                 : -----------------FNKNQEFSLKLNKR-----DIKMITTLLQ---------------FEGFQKE-------PVVREQLQRM--------------- : 301
Cryptococcus_neoformans_Spo11                   : -----------------HISYDHL-IPLEQS-----DIQMAMSMLR----------------DHQTLP-------VEWKRELSHM--------------- : 356
Candida_albicans_Spo11                          : -------------------------IKICKN-----GTSKYNDIPY---------------------------------RELTRG--------------- : 272
Kluyveromyces_lactis_Spo11                      : -----ITRNT-------ESNSMLVLLPLSLR-----DATFAMGLIR------RLCKEEEEEEGSVRKH----TMRQNLKYEVQRQ--------------- : 312
Neurospora_crassa_Spo11                         : FEDERPAKRPRSRRTK-SHPSDYT-ASLSER-----DREKAVSVLR-----------DICGTARLDAT------EREHKLELQRM--------------- : 310
Arabidopsis_thaliana_Spo11-1                    : -----------------SVPKQCL-LPLTEE-----DKKRTEAMLL---------------RCYLKRE------MPQWRLELETM--------------- : 314
Oryza_sativa_Spo11-1                            : -----------------RLPDCCL-LHLSSE-----DRRKAEGILS---------------RCYLHRE------APQWRLELEAM--------------- : 319
Zea_mays_Spo11-1                                : -----------------CLPDCCL-LRLSPE-----DRRKAEGILA---------------RCYLHRE------APEWRSELEAM--------------- : 319
Arabidopsis_thaliana_Spo11-3/Top_VIA            : -----------------KIPEQCR-LPMTEQ-----DIKTGKDMLE---------------EDFVKKN-------PGWVEELNLM--------------- : 312
Oryza_sativa_Spo11-3/Top_VIA                    : -----------------RVPEQCR-LPMTDH-----DIKVGKELLE---------------EDFVKQN-------EGWVKELETM--------------- : 312
Zea_mays_Spo11-3/Top_VIA                        : -----------------RVPEQCR-LPMTDH-----DIKVGKELLE---------------EDFVKQN-------EGWVKELETM--------------- : 312
Thalassiosira_pseudonana_Spo11-3/Top_VIA        : -----------------DLPDQCR-LNMTEN-----DIKTGKELLQ---------------EAFIMKN-------PKWMKELQIM--------------- : 312
Phaeodactylum_tricornutum_Spo11-3/Top_VIA       : -----------------DLPEQCR-LDMTEN-----DIKTGKEMMK---------------EDFIQKN-------PEWMKELEIM--------------- : 312
Fragilariopsis_cylindrus_Spo11-3/Top_VIA        : -----------------DLPDQCR-LDMTEN-----DIKTGKELLN---------------EEFIQKN-------PKWMKEIENM--------------- : 312
Pseudo-nitzschia_multiseries_Spo11-3/Top_VIA    : -----------------DLPDQCR-LDMTEN-----DIKTGKELMT---------------EDFIQKN-------PKWMKELETM--------------- : 312
Pseudo-nitzschia_multistriata_Spo11-3/Top_VIA   : -----------------DLPDQCR-LDMTES-----DIKTGKELMT---------------EDFIQKN-------PMWMKELEIM--------------- : 312
Seminavis_robusta_Spo11-3/Top_VIA               : -----------------NLPEQCR-LDMTEF-----DIKTGKELLK---------------EDFIVKN-------PKWMKEVAEM--------------- : 312
Ectocarpus_siliculosus_Spo11-3/Top_VIA          : -----------------NIPEQCR-LDMTEH-----DIKTGKELLQ---------------EDFIKKN-------EPWCRELELM--------------- : 312
Thermococcus_gammatolerans_Top_VI               : --------------GLENVTEKLKGIPPNKKGGPTGDYKRILEEME---------------YPWFQNR--------EWQRQLKLA--------------- : 328
Sulfolobus_acidocaldarius_Top_VI                : ---------------LSDQERRNYIIKAKDA-----DVKRAMEIKN---------------YEWFKTK--------GWQHEISVF--------------- : 335
                                                                                    d
```

```
                                                           *       620         *       640         *       660         *
Arabidopsis_thaliana_Spo11-2                      : ----VQTGKRAEIEAL---YCHG--YNYLG-----------KYIATKI--------------------VQGKYI : 355
Oryza_sativa_Spo11-2                              : ----LETGKRAEIEAL---YSHG--FDFLG-----------KYVARKI--------------------VQGDYI : 357
Zea_mays_Spo11-2                                  : ----VERGKRADIEAL---YSHG--FDFLG----------K-YIARKI--------------------VQGDYI : 354
Thalassiosira_pseudonana_Spo11-2                  : ----KESGKKVELEAL---YWLG--SDYMS-----------------------------------------NWV : 272
Phaeodactylum_tricornutum_Spo11-2                 : ----LKNGYKMELEAL---NWLG--MDFIT-----------KWLGDIFHYQDRAGHGHE---------GNSCWM : 378
Fragilariopsis_cylindrus_Spo11-2                  : ----YK--YKVELEAL---HWKG--MDYMC-----------KFVYETILGHEKKHEEESVRRRRRRYRRQSDFS : 462
Pseudo-nitzschia_multiseries_Spo11-2              : ------RNYKVELEAL---HWKG--SDYLC-----------QFV------------------------------ : 262
Pseudo-nitzschia_multistriata_Spo11-2             : ----RK--YKVELEAL---HWKG--ADYLC-----------QFVYNAM--------------------LSEYER : 434
Seminavis_robusta_Spo11-2                         : ----QENGYKVELESL---NWLG--MDFMS-----------QWVHGALI-------------------EGGEQL : 393
Ectocarpus_siliculosus_Spo11-2                    : PSLAIEQSMYLVVSLL---SKTA--FSLFL----------MQIFPLRLY-------------------FLSSRI : 329
Homo_sapiens_Spo11                                : ----ADSKMKAEIQAL---TFLS--SDYLS----------RVYLPNKL--------------------KFGGWI : 350
Mus_musculus_Spo11                                : ----ADSKMKAEIQAL---TLLS--SDYLS----------RVYLPNKL--------------------RFGGWI : 350
Xenopus_(Silurana)_tropicalis_Spo11               : ----RSSSLDCPCNCM---AW-----------------------PPTI--------------------RFREVI : 309
Danio_rerio_Spo11                                 : ----QNLKRKAEIQCL---ASIA--PHFLT----------RVYLPNKL--------------------RYGGWI : 350
Saccharomyces_cereviceae_Spo11                    : ----IFFQKKAEMNEI---DARI--FEYK--------------------------------------------- : 354
Schizosaccharomyces_pombe_Spo11                   : ----LFIQKKAEIQAI---------LEFPS------------WIKGKLADAD----------------KSGKHS : 334
Cryptococcus_neoformans_Spo11                     : ----LHLHRKAEIEIIIASSWSS---DHVGSNGSGDREGDRQPLPDVE--------------------RLINYI : 403
Candida_albicans_Spo11                            : ----LYLFKKAEMNVV---QANNANQSYVN------------YMVSKIL-------------------GTPSYT : 308
Kluyveromyces_lactis_Spo11                        : ----LFFQRKGEMNVV----NNGNVQQYFK------------WMYSRI--------------------H----- : 341
Neurospora_crassa_Spo11                           : ----LMLNIKAEIQAV---DDFGDITNWLD----------ERLLKHSM--------------------DVNSH- : 346
Arabidopsis_thaliana_Spo11-1                      : ----LKRGVKFEIEAL---SVHS--LSFLS----------EVYIPSKI--------------------RREVSS : 349
Oryza_sativa_Spo11-1                              : ----LQKGVKFEIEAL---SACS--ISFLS----------EEYIPKKI--------------------KQGRHI : 354
Zea_mays_Spo11-1                                  : ----LQKGVKFEIEAL---SANS--ISFLS----------HEYIPQKI--------------------KQGMHL : 354
Arabidopsis_thaliana_Spo11-3/Top_VIA              : ----VKTKQKAEIQAL---SSFG--FQYLS----------EVYLPLKL--------------------QQQDWL : 347
Oryza_sativa_Spo11-3/Top_VIA                      : ----LRTRQKAEIQAL---SSFG--FQYLT----------EVYLPLKL--------------------QQQDWI : 347
Zea_mays_Spo11-3/Top_VIA                          : ----LRTKQKAEIQAL---SSFG--FQYLT----------EVYLPLKL--------------------QEQDWI : 347
Thalassiosira_pseudonana_Spo11-3/Top_VIA          : ----VKTKQKAEIQAL---SSFG--FQYIT----------EVYLPRKL--------------------KEGDWI : 347
Phaeodactylum_tricornutum_Spo11-3/Top_VIA         : ----VKTKKKAEIQAL---SSFG--FQYVT----------EQYLPRKL--------------------KEGDWI : 347
Fragilariopsis_cylindrus_Spo11-3/Top_VIA          : ----IKTKKKAEIQAL---SSFG--FQYIT----------EEYLPRKL--------------------REGDWI : 347
Pseudo-nitzschia_multiseries_Spo11-3/Top_VIA      : ----VKTKKKAEIQAL---SSFG--FQYIT----------EEYLPRKL--------------------REGDWI : 347
Pseudo-nitzschia_multistriata_Spo11-3/Top_VIA     : ----VKTKKKAEIQAL---SSFG--FQYIT----------EEYLPRKL--------------------KEGDWI : 347
Seminavis_robusta_Spo11-3/Top_VIA                 : ----VKTKKKAEIQAL---SSFG--FQYIT----------EEYLPRKL--------------------REGDWI : 347
Ectocarpus_siliculosus_Spo11-3/Top_VIA            : ----IKSKKKAEIQSL---SAFG--FQYLT----------QNYLPQKL--------------------QNGDWV : 347
Thermococcus_gammatolerans_Top_VI                 : ----LKWGVRIEQQAL---ANKS--LEFVA----------KEYLPEKI--------------------NSGDLL : 363
Sulfolobus_acidocaldarius_Top_VI                  : ----LDKKSKLEIEAM---ASKG--LRFLA----------FQYIPEKI--------------------KNSDFI : 370
                                                              e   6
```
