# Supplementary material for: Identification of the meiotic toolkit in diatoms and exploration of meiosis-specific SPO11 and RAD51 homologs in the sexual species Pseudo-nitzschia multistriata and Seminavis robusta
Source: BMC Genomics. 2015 Nov 14;16:930. doi: 10.1186/s12864-015-1983-5 (PMC4647503; doi:10.1186/s12864-015-1983-5)
Supplement: Additional file 4: — Multiple sequence alignment of Rad51 proteins used in building the phylogenetic tree of Rad51, shown in Fig. 3 . (PDF 1107 kb) [file 12864_2015_1983_MOESM4_ESM.pdf]

Additional file 4. Multiple sequence alignment of Rad51 proteins used in phylogenetic analysis of Rad51 protein family.

```
                                                 *         20         *         40         *         60         *         80         *       100
Arabidopsis_thaliana_Dmc1               : GINAGDVKKLQEA---------------------------------GIHTCNGLM-MHT----KKNLTGIKG--LSEAKVDKICEAA------------- :  47
Oryza_sativa_Dmc1                       : GINSGDVKKLQDA---------------------------------GIYTCNGLM-MHT----KKSLTGIKG--LSEAKVDKICEAA------------- :  47
Mus_musculus_Dmc1                       : GINMADIKKLKSV---------------------------------GICTIKGIQ-MTT----RRALCNVKG--LSEAKVEKIKEAA------------- :  47
Homo_sapiens_Dmc1                       : GINVADIKKLKSV---------------------------------GICTIKGIQ-MTT----RRALCNVKG--LSEAKVDKIKEAA------------- :  47
Danio_rerio_Dmc1                        : GINVADIKKLKSV---------------------------------GICTVKGIQ-MTT----RRALCNIKG--LSEAKVDKIKEAA------------- :  47
Ectocarpus_siliculosus_Dmc1             : GIKMAEINKLKEA---------------------------------NLATVGA------------RLLTIRG--FSDATVAKIQLAA------------- :  40
Oryza_sativa_Rad51-A                    : GIAALDVKKLKDS---------------------------------GLYTVESVA-YTP----RKDLLQIKG--ISEAKVDKIVEAA------------- :  47
Arabidopsis_thaliana_Rad51-A            : GIASVDVKKLRDA---------------------------------GLCTVEGVA-YTP----RKDLLQIKG--ISDAKVDKIVEAA------------- :  47
Mus_musculus_Rad51-A                    : GINANDVKKLEEA---------------------------------GYHTVEAVA-YAP----KKELINIKG--ISEAKADKILTEA------------- :  47
Homo_sapiens_Rad51-A                    : GINANDVKKLEEA---------------------------------GFHTVEAVA-YAP----KKELINIKG--ISEAKADKILAEA------------- :  47
Danio_rerio_Rad51-A                     : GISSSDIKKLEDG---------------------------------GFHTVEAVA-YAP----KKELLNIKG--ISEAKADKILTEA------------- :  47
Chlamydomonas_reinhardtii_Rad51-A       : GVAAADIKKLKEG---------------------------------GIHTVEALA-FMP----KKQLSEIKG--LSEAKIDKMQLVA------------- :  47
Micromonas_sp._Rad51-A                  : GVAASDIKKLLEA---------------------------------GIHTVEGLA-YAS----KKHLKDIKG--LSEMKVEKLKMAA------------- :  47
Ostreococcus_lucimarinus_Rad51-A        : GIAASDVSKLRDA---------------------------------GVHTVEGLA-AAS----RKHLQSIKG--LSEQKVEKLKQAA------------- :  47
Ectocarpus_siliculosus_Rad51-A          : GINRNDVQRLSEA---------------------------------GYCTVESIS-HCT----IRKLVEVKG--ISEQKAAKLKETV------------- :  47
Phaeodactylum_tricornutum_Rad51-A       : GIAANDIQKLNAA---------------------------------GYHTVESIA-HAT----IRKLSDVKG--ISEAKVLKLKEIT------------- :  47
Thalassiosira_pseudonana_Rad51-A        : GVAVNDIQKLNDA---------------------------------GFHTIESVA-HST----VRKLSEVRG--LSEAKVLKLKDIV------------- :  47
Fragilariopsis_cylindrus_Rad51-A2       : GISANDIQKLQSG---------------------------------GYNTIQSIA-HAT----VRKLSDVKG--ISEAKVTKLKELV------------- :  47
Fragilariopsis_cylindrus_Rad51-A1       : GIAANDITKLTTA---------------------------------GYHTVESIA-HAT----KRKLSDVKG--ISEAKVLKIKEIV------------- :  47
Pseudo-nitzschia_multiseries_Rad51-A2   : GIAANDLQKLQNA---------------------------------GYYTVESIA-HAT----TRRLSDVKG--ISEAKVQKLKEIV------------- :  47
Pseudo-nitzschia_multiseries_Rad51-A1   : GIAINDIQKLQNA---------------------------------GYHTVESVA-HAT----ARRLSDVKG--ISEAKVTKLKEIV------------- :  47
Pseudo-nitzschia_multistriata_Rad51-A2  : GIXTNDIQKLQNA---------------------------------GYYTIESIA-HAT----TRRLSDVKG--ISEAKVLKLKDIV------------- :  47
Pseudo-nitzschia_multistriata_Rad51-A1  : GIATNDIQKLQTA---------------------------------GYHTIESVA-HAT----VRRLSDVKG--ISEAKIIKLKGIV------------- :  47
Seminavis_robusta_Rad51-A               : ---------------------------------------------------------------------------------------------------- :   -
Arabidopsis_thaliana_Rad51-B            : GLHTKISNIFAAR---------------------------------NIITAKDAL-SMT----EFELMELLD--VGMKEIRSAISFI------------- :  47
Oryza_sativa_Rad51-B                    : RLPPHLAHILAAR---------------------------------RLTTAKDVL-SLP----EVELMGVLD--AGIHTARAAVAHV------------- :  47
Mus_musculus_Rad51-B                    : GLSPELCDRLSRY---------------------------------QIVNCQHFL-SLS----PLELMKVTG--LSYRGVHELLHTV------------- :  47
Homo_sapiens_Rad51-B                    : GLSQELCDRLSRH---------------------------------QILTCQDFL-CLS----PLELMKVTG--LSYRGVHELLCMV------------- :  47
Danio_rerio_Rad51-B                     : GVSADLCERLKRH---------------------------------QLETCQDVL-SVT----QVELSRLAG--LSYPAALNLQRLV------------- :  47
Chlamydomonas_reinhardtii_Rad51-B       : ALDPYLRDHLIAN---------------------------------NLTTARDVL-LLS----PLDLMELLG--LTWTAAHQLLADV------------- :  47
Micromonas_pusilla_Rad51-B              : GLDVDVLSRLEGH---------------------------------NFRTAEDVL-TRS----SLDLVELLD--VSLPTAERVVASV------------- :  47
Ostreococcus_lucimarinus_Rad51-B        : AVDQTLAAKLESR---------------------------------GCRTAEDAL-YRA----PLDVVELAD--VSMHRARQFIISV------------- :  47
Ectocarpus_siliculosus_Rad51-B          : GLDDDLVSRLASA---------------------------------GLHTAGDMF-SKT----ELQLVQSLD--ASREKVVELLDYV------------- :  47
Thalassiosira_pseudonana_Rad51-B        : GLPMQLFKDLISR--------------------------------------------------PRALA------LRSLTADGVATAA------------- :  31
Phaeodactylum_tricornutum_Rad51-B       : ATDRLQWCIVPNN---------------------------------VMSTADRILARL-----PLHLFENLATDGNTIPARELREQICASLDTLNTASRN :  62
Fragilariopsis_cylindrus_Rad51-B        : RLKSAICNRLKRF-VVRNNGRKNKNSRTAGGGGGRRISGISPSSTCSIKTIGQLL-RLS----KCTLLFALDPILTYEEVSIFLDRV------------- :  81
Pseudo-nitzschia_multiseries_Rad51-B    : SLKANICKRLEGFRVRVRNSARDRNRNKNNGGG-GSGISLSATTISSIRTIGQLL-RLS----KYSLMLALDPILTYEEVGIFLNRV------------- :  81
Pseudo-nitzschia_multistriata_Rad51-B   : SLKANICKRLKGF--RVRNNARGRSKNKNRGRGVGSGISLSTTTTYNIKTIGQLL-RXS----KYTLMLALDPILTYEEVGILLNRV------------- :  80
Seminavis_robusta_Rad51-B               : ELRRQMCAKLKKFASPHYKNNTRGNNYFE-----------------SVTTVGHLLTRTT----PWTLLKILDPLLTMEEVQLFLHRV------------- :  66
Arabidopsis_thaliana_Rad51-C            : PLSPSIRGKLISA---------------------------------GYTCLSSIA-SVS----SSDLARDAN--ITEEEAFEILKLA------------- :  47
Oryza_sativa_Rad51-C                    : PIATSHRANLLAA---------------------------------GYSSLAALS-AAS----PPRLARDLS--IEVHEAEEILKVAVGA----NKSKGA :  56
Mus_musculus_Rad51-C                    : PLSPAVRGKLVAA---------------------------------GFQTAEDVL-EVK----PSELSKEVG--ISKEEALETLQIL------------- :  47
Homo_sapiens_Rad51-C                    : PLSPAVRVKLVSA---------------------------------GFQTAEELL-EVK----PSELSKEVG--ISKAEALETLQII------------- :  47
Danio_rerio_Rad51-C                     : PLAPSVKVKLINA---------------------------------GFQAASDLT-DMR----PLQLCKEAG--ISQEEAVELLQML------------- :  47
Chlamydomonas_reinhardtii_Rad51-C       : PLPPHLRNRLLAT---------------------------------GFGTVADLERAGG----PMGLARETG--LSPDEAHEVLQLA----------GAA :  51
Micromonas_pusilla_Rad51-C              : PLAPSVRSKLQRA---------------------------------GFVSVRDVKKTRG----PVELALEAG--LTNEEASEVMKVV------------- :  48
Ostreococcus_lucimarinus_Rad51-C        : ALAPSTRSKLQRA---------------------------------GVHSARDVL-RISGNS-PSTLARECA--LTLDEANDALKAA------------- :  50
Ectocarpus_siliculosus_Rad51-C          : ---------MGSA---------------------------------AAAVAGGVL--------RGGLAGSQA---------------------------- :  22
Phaeodactylum_tricornutum_Rad51-C       : PLRPSTLQTLQKR---------------------------------GFYGTRELTESKQSG--MANLAAELA--LNLSQTSALYAEV------------- :  50
Thalassiosira_pseudonana_Rad51-C        : NQSNGQNERHDDN---------------------------------ATSQFGDFATELGC---SVSQAADYAHELNDALHSVGLSSVSSDRSEHNNHSAT :  64
Fragilariopsis_cylindrus_Rad51-C        : ---MHDPRRRSSH---------------------------------HHQAGGGITSTNR----------------------------------------- :  23
Pseudo-nitzschia_multiseries_Rad51-C    : LLRPSTLRRFTKR---------------------------------GFESTDEIE-ESRANGGINLLASELD--VSLQEAAGLIREVQGCLGSNNNNNNH :  64
Pseudo-nitzschia_multistriata_Rad51-C   : LLRPSTLERFSRR---------------------------------GFESTGEIE-ESRASGGINLLASELD--VSLGEAAGLIREVQGC----LGSFLP :  60
Seminavis_robusta_Rad51-C               : SLRPGTLKLFQQR---------------------------------GFTNTAEVE-RSRENGGLANLAAELG--CSLRQANDLFREI------------- :  51
Arabidopsis_thaliana_Xrcc3              : ---------MQNG--------------------------------------------------------------------------------------- :   4
Oryza_sativa_Xrcc3                      : -MRPAAPARHPSA---------------------------------AASSSSSTNSQQE----PR----------------------------------- :  27
Mus_musculus_Xrcc3                      : DLNPRITAAVKRG---------------------------------RLKSVKEIL-CYS----GPDLQRLTG--LPSHDVQCLLRAA------------- :  47
Homo_sapiens_Xrcc3                      : DLNPRIIAAIKKA---------------------------------KLKSVKEVL-HFS----GPDLKRLTN--LSSPEVWHLLRTA------------- :  47
Danio_rerio_Xrcc3                       : ELNPRIIAAVKKG---------------------------------NFRSAKEVL-CVS----GPDLQRLTR--LSKADVQRLHQAV------------- :  47
Micromonas_sp._Xrcc3                    : ----------------------------------------------MTTRENIIL--------------------------------------------- :   9
Ostreococcus_lucimarinus_Xrcc3          : ---------------------------------------------------------------------------------------------------- :   -
Ectocarpus_siliculosus_Xrcc3            : GISGVGCPPGPQY---------------------------------GVNRRQSPH-GQQ----PHRLSGKSS--KPDPWLAGTAPEC------------- :  47
Thalassiosira_pseudonana_Xrcc3          : SMPRKYQNPMVNN---------------------------------PYKKDPPSSSTTNANVNGSVRAERNFNTTTMSTKQKFIEKYRLQ----AMHHSH :  63
Phaeodactylum_tricornutum_Xrcc3         : KAPFSSCSK-------------------------------------GSASADTFPIRQEQSASRIPLTEIRNQYASTSTNRRQRRLS---------IPNA :  54
Fragilariopsis_cylindrus_Xrcc3          : MRSSVFCPE------------------------------------------------------------------------------------------- :   9
Pseudo-nitzschia_multiseries_Xrcc3      : --------------------------------------------------------------------------MRYSYVS------------------- :   7
Pseudo-nitzschia_multistriata_Xrcc3     : TXQHGGSLPVYSN---------------------------------GRKTTAAVA--------PVSALELFRIGVKRRXGTGMFSGG----------GNS :  49
Seminavis_robusta_Xrcc3                 : GLDKNHPSLQKEN---------------------------------FSCNNDGNDPSGS----RKRSWQDTNASSIAPDASHIRHPKTSDNDNNKRHAPS :  63
Sulfolobus_solfataricus_Rad-A           : GISQTVINKLIEA---------------------------------GYSSLETLA-VAS----PQDLSVAAG--IPLSTAQKIIKEA------------- :  47
Aeropyrum_pernix_Rad-A                  : GVGPTTAQKLMEA---------------------------------GYTTLEAIA-AAT----PQEVSQATG--IPILTAQKIVDAA------------- :  47
```

```
                                                  *        120         *        140         *        160         *        180         *        200
Arabidopsis_thaliana_Dmc1                 : ----EKIVNFG-------------------------YMTGSDALIKRKSVV---------------------------------------------KITT :  73
Oryza_sativa_Dmc1                         : ----EKLLSQG-------------------------FITGSDLLIKRKSVV---------------------------------------------RITT :  73
Mus_musculus_Dmc1                         : ----NKLIEPG-------------------------FLTAFQYSERRKMVF---------------------------------------------HITT :  73
Homo_sapiens_Dmc1                         : ----NKLIEPG-------------------------FLTAFEYSEKRKMVF---------------------------------------------HITT :  73
Danio_rerio_Dmc1                          : ----GKLLTCG-------------------------FQTASEYCIKRKQVF---------------------------------------------HITT :  73
Ectocarpus_siliculosus_Dmc1               : ----AKVDTSGS---------------------SGMFKIGLQCRQAREKVI---------------------------------------------KVHT :  70
Oryza_sativa_Rad51-A                      : ----SKLVPLG-------------------------FTSASQLHAQRLEII---------------------------------------------QVTT :  73
Arabidopsis_thaliana_Rad51-A              : ----SKLVPLG-------------------------FTSASQLHAQRQEII---------------------------------------------QITS :  73
Mus_musculus_Rad51-A                      : ----AKLVPMG-------------------------FTTATEFHQRRSEII---------------------------------------------QITT :  73
Homo_sapiens_Rad51-A                      : ----AKLVPMG-------------------------FTTATEFHQRRSEII---------------------------------------------QITT :  73
Danio_rerio_Rad51-A                       : ----AKMVPMG-------------------------FTTATEFHQRRAEII---------------------------------------------QIST :  73
Chlamydomonas_reinhardtii_Rad51-A         : ----FKMVPMG-------------------------FTTAAVAEQRKEVI----------------------------------------------SITT :  73
Micromonas_sp._Rad51-A                    : ----TKVVPLG-------------------------FTTASMVQAVRQDTI---------------------------------------------MVTT :  73
Ostreococcus_lucimarinus_Rad51-A          : ----NAIVPAG-------------------------FTTAKMIDQQRQDTI---------------------------------------------YITT :  73
Ectocarpus_siliculosus_Rad51-A            : ----YKLVPIG-------------------------FTTASQHLLQRQDLI---------------------------------------------TLTT :  73
Phaeodactylum_tricornutum_Rad51-A         : ----KSMVPMD-------------------------FKTAADALEDRKALV---------------------------------------------TLTT :  73
Thalassiosira_pseudonana_Rad51-A          : ----KTMVPLD-------------------------FKTAADALEDRKALV---------------------------------------------TLTT :  73
Fragilariopsis_cylindrus_Rad51-A2         : ----KGLVPME-------------------------FKTAADALLDRKAIV---------------------------------------------NLTT :  73
Fragilariopsis_cylindrus_Rad51-A1         : ----NTMVTME-------------------------FQTAADALEDRQGLV---------------------------------------------TLTT :  73
Pseudo-nitzschia_multiseries_Rad51-A2     : ----KQMVSME-------------------------FKTAADALEDRQSIA---------------------------------------------MLTT :  73
Pseudo-nitzschia_multiseries_Rad51-A1     : ----KQMVAME-------------------------FQTAADALETRKNIV---------------------------------------------HLTT :  73
Pseudo-nitzschia_multistriata_Rad51-A2    : ----KQMVSME-------------------------FKTAADALEDRQTIA---------------------------------------------MLTT :  73
Pseudo-nitzschia_multistriata_Rad51-A1    : ----KQMVAME-------------------------FQTATDXLEARKNIV---------------------------------------------QLTT :  73
Seminavis_robusta_Rad51-A                 : ------MVPMD-------------------------FKTAADALEDRKSIV---------------------------------------------TLTT :  24
Arabidopsis_thaliana_Rad51-B              : ---SEATSPP--------------------------CQSARSLLEKKVENEHLSG-----------------------------------------HLPT :  77
Oryza_sativa_Rad51-B                      : ---SEIACPP--------------------------YQTALALLEAFRARGDG-------------------------------------------RIAT :  75
Mus_musculus_Rad51-B                      : ---SKACAPQ--------------------------MQTAYELKTRRSAHLSPA------------------------------------------FLST :  76
Homo_sapiens_Rad51-B                      : ---SRACAPK--------------------------MQTAYGIKAQRSADFSPA------------------------------------------FLST :  76
Danio_rerio_Rad51-B                       : ---SKACAPA--------------------------VITALDLWKRKEEL----------------------------------------------CFST :  72
Chlamydomonas_reinhardtii_Rad51-B         : ---SAQISPP--------------------------YSTAYDVFTQQATAEAPA------------------------------------------PLRT :  76
Micromonas_pusilla_Rad51-B                : ----AKCVCPK-------------------------PQTAMALLRQRGGGGGGGGTARSSSGARASAS---------------GDGGGGGGASWPAFLPT : 103
Ostreococcus_lucimarinus_Rad51-B          : ---AKAVAPT--------------------------PTTALDALRRSQ------------------------------------------------YVPL :  70
Ectocarpus_siliculosus_Rad51-B            : ---STRIVPEQ-------------------------AKTAGDLLRERREAGASF------------------------------------------FVAT :  77
Thalassiosira_pseudonana_Rad51-B          : ------------------------------------DDDDVDVETSADD-----------------------------------------------DLPT :  48
Phaeodactylum_tricornutum_Rad51-B         : GSVTHRIRTVGPLLQCSAASLVRTLDPL--------LTYAFELLHSTTSSLNDCNRCEETFSRMR-------------------------------YLPT : 123
Fragilariopsis_cylindrus_Rad51-B          : ---CNQCSPK--------------------------PNSVLQLLKDTTNANADGISCSEQNNESQQQDRQQQQQRPSTSASTSTTTDPITFGSSMRDLPT : 152
Pseudo-nitzschia_multiseries_Rad51-B      : ---CKQCAPE--------------------------SRSVLKLYRATTANTNANANANTDLN-AIPRD-----------------ADPVAFGSRMRHLPT : 134
Pseudo-nitzschia_multistriata_Rad51-B     : ---CNQCAPN--------------------------PISALELFGATTLNTDNVLGMSXYRDEGKQHE-----------------NDPISFGSKMRYLPT : 134
Seminavis_robusta_Rad51-B                 : ---AKVCCPP--------------------------PQTALELLQQSQQHANAK------------------------------------------YLPT :  95
Arabidopsis_thaliana_Rad51-C              : ---NQSCCNGSRSLING-------------------AKNAWDMLHEEESLP---------------------------------------------RITT :  80
Oryza_sativa_Rad51-C                      : DGPSTSSVLKG-------------------------VQNAWDMLSDEQSRR---------------------------------------------HINT :  86
Mus_musculus_Rad51-C                      : ---RRECLTNKPRCAGTSVANE--------------KCTALELLEQEHTQG---------------------------------------------FIIT :  85
Homo_sapiens_Rad51-C                      : ---RRECLTNKPRYAGTSESHK--------------KCTALELLEQEHTQG---------------------------------------------FIIT :  85
Danio_rerio_Rad51-C                       : ---RDDAQPQQQRAAAD-------------------GVTALDLLHQEQTLG---------------------------------------------SIVT :  80
Chlamydomonas_reinhardtii_Rad51-C         : GGPASSNWRAG-------------------------TVSAADLLVAAAATP---------------------------------------------RIIS :  81
Micromonas_pusilla_Rad51-C                : RFGVDGTALAG-------------------------AKSASELLREETGKL---------------------------------------------PIYT :  78
Ostreococcus_lucimarinus_Rad51-C          : RFNGEGIALRG-------------------------ARSAAELLREERARW---------------------------------------------SVFT :  80
Ectocarpus_siliculosus_Rad51-C            : ------------------------------------GASARELIVRGRHKK---------------------------------------------PIIT :  41
Phaeodactylum_tricornutum_Rad51-C         : ----DSCLRTTTPV----------------------TKTAAALLEENVEGQG--------------------------------------------CIIT :  80
Thalassiosira_pseudonana_Rad51-C          : TVNETPITPSSSSHH---------------------AQTAASILRSTHSSSSSTQQHR--------------------------------------NIVS : 105
Fragilariopsis_cylindrus_Rad51-C          : ------------------------------------------------------------------------------------------------HVVS :  27
Pseudo-nitzschia_multiseries_Rad51-C      : NNNNNHNRYGGDSENANGANNATASSGGNTDNNDDGIVTAYDLLHHQHLTNTDAIGGVDRRRRK--------------------------------HIVT : 132
Pseudo-nitzschia_multistriata_Rad51-C     : RGRADASNENQSIGIGKDNEHNGTEAPPKSRDHDDGIVTAYELLHRSDPLSFPGSNTGRSQSQSRSGC---------------LPNG---------HIVT : 136
Seminavis_robusta_Rad51-C                 : ---SGAAQPMGQ------------------------AKTASQLLNVPNNNAKMGSR----------------------------------------KLVT :  84
Arabidopsis_thaliana_Xrcc3                : ------------------------------------KIKPENLLRRSPTNR---------------------------------------------KLST :  23
Oryza_sativa_Xrcc3                        : ------------------------------------PENPLLLLPSSRAG----------------------------------------------KLSL :  45
Mus_musculus_Xrcc3                        : ---SLHLRGSR-------------------------VLSALHLFQQKESFPEQHQ-----------------------------------------RLSL :  78
Homo_sapiens_Xrcc3                        : ---SLHLRGSS-------------------------ILTALQLHQQKERFPTQHQ-----------------------------------------RLSL :  78
Danio_rerio_Xrcc3                         : ---AASVRKSK-------------------------PVTALQLIQGECPVLEPGH-----------------------------------------RLSF :  78
Micromonas_sp._Xrcc3                      : ------------------------------------PKVTRERISADPSTWAP-------------------------------------------KLTT :  30
Ostreococcus_lucimarinus_Xrcc3            : ------------------------------------MRVAAAAAADARAA----------------------------------------------CVST :  18
Ectocarpus_siliculosus_Xrcc3              : -------------------------------------TTALAMSKDAARLD---------------------------------------------RLSF :  65
Thalassiosira_pseudonana_Xrcc3            : RYGNASRRRIIQHGNSRNGDRGEGGRNSNNSLFVVQSYSAMELYRKHESWRSWWRKHYKNQNLQQEKT---------------TKENA--------PLSR : 140
Phaeodactylum_tricornutum_Xrcc3           : ISVQSPLLPS--------------------------PTTALQLLQRHQQRTHTLR-----------------------------------------LIPN :  87
Fragilariopsis_cylindrus_Xrcc3            : ------------------------------------PSSSSSRSSRSHSSS---------------------------------------------ENDA :  28
Pseudo-nitzschia_multiseries_Xrcc3        : ---------------------------------------------------------------------------------------------------- :   -
Pseudo-nitzschia_multistriata_Xrcc3       : NGSSSXNRIGG-------------------------FETYNEYSGSNRSTRTSLSSSV--------------------------------------SAPP :  86
Seminavis_robusta_Xrcc3                   : RWTNKRKSKIQ-------------------------PMTAFSLLQKNQSARQ--------------------------------------------SLYL :  94
Sulfolobus_solfataricus_Rad-A             : ----RDALDIR-------------------------FKTALEVKKERMNVK---------------------------------------------KIST :  73
Aeropyrum_pernix_Rad-A                    : ----REALNID-------------------------FKTAYDLKIESMNIK---------------------------------------------KITT :  73
                                                                                 a
```

```
                                                  *       220         *       240         *       260         *       280         *       300
Arabidopsis_thaliana_Dmc1              : GCQA---------LDDLLGGG------IETS-------------------AITEAFGEFRSGKTQLAHTLCVTTQ--------LPT------NMKGG--N : 123
Oryza_sativa_Dmc1                      : GSQA---------LDELLGGG------IETL-------------------CITEAFGEFRSGKTQLAHTLCVSAQ--------LPI------HMHGG--N : 123
Mus_musculus_Dmc1                      : GSQE---------FDKLLGGG------IESM-------------------AITEAFGEFRTGKTQLSHTLCVTAQ--------LPG------TGGYS--G : 123
Homo_sapiens_Dmc1                      : GSQE---------FDKLLGGG------IESM-------------------AITEAFGEFRTGKTQLSHTLCVTAQ--------LPG------AGGYP--G : 123
Danio_rerio_Dmc1                       : GSLE---------FDKLLGGG------VESM-------------------AITEAFGEFRTGKTQLSHTLCVTAQ--------LPG------EYGYT--G : 123
Ectocarpus_siliculosus_Dmc1            : GSKQ---------LDALLGGG------IETG-------------------SITEFFGEFRSGKTQLCHTLCVTSQ--------LSK------ESGGA--- : 119
Oryza_sativa_Rad51-A                   : GSRE---------LDKILDGG------IETG-------------------SITEIYGEFRSGKTQLCHTLCVTCQ--------LPL------DQGGG--E : 123
Arabidopsis_thaliana_Rad51-A           : GSRE---------LDKVLEGG------IETG-------------------SITELYGEFRSGKTQLCHTLCVTCQ--------LPM------DQGGG--E : 123
Mus_musculus_Rad51-A                   : GSKE---------LDKLLQGG------IETG-------------------SITEMFGEFRTGKTQICHTLAVTCQ--------LPI------DRGGG--E : 123
Homo_sapiens_Rad51-A                   : GSKE---------LDKLLQGG------IETG-------------------SITEMFGEFRTGKTQICHTLAVTCQ--------LPI------DRGEG--E : 123
Danio_rerio_Rad51-A                    : GSKE---------LDKLLQGG------IETG-------------------SITEMFGEFRTGKTQLCHTLAVTCQ--------LPI------DQGGG--E : 123
Chlamydomonas_reinhardtii_Rad51-A      : GCKE---------LDTILEGG------IETG-------------------SITEIYGEYRCGKTQLCHTLCVTCQ--------LPV------EMGGG--E : 123
Micromonas_sp._Rad51-A                 : GASK---------LDELLGGG------FESG-------------------SLTEIYGEFRTGKTQLCHTLAVTCQ--------LPL------DQGGA--E : 123
Ostreococcus_lucimarinus_Rad51-A       : GSAK---------VDELLQGG------IESG-------------------SVTEIYGEFRTGKTQLMHTLAVTSQ--------MPI------EHGGG--E : 123
Ectocarpus_siliculosus_Rad51-A         : GSKE---------LDKLLEGG------IETG-------------------SLTEVFGEFRTGKTQLCHTLCVACQ--------MPL------DAGGG--E : 123
Phaeodactylum_tricornutum_Rad51-A      : GSIE---------LDKLLEGG------VETG-------------------SITEVFGEFRTGKTQLCHTLCVTCQ--------MAV------TEGGA--E : 123
Thalassiosira_pseudonana_Rad51-A       : GSTE---------LDKLLEGG------VETG-------------------SLTEVFGEFRTGKTQLCHTLCVTCQ--------MPI------SEGGA--E : 123
Fragilariopsis_cylindrus_Rad51-A2      : GSVE---------VDKLLEGG------IETG-------------------SITEVFGEFRTGKTQLCHTLCVTCQ--------MSI------DQGGA--E : 123
Fragilariopsis_cylindrus_Rad51-A1      : GAVE---------LDKLLEGG------IETG-------------------SITEVFGEFRTGKTQLCHTLCVTCQ--------MAI------DNGGA--E : 123
Pseudo-nitzschia_multiseries_Rad51-A2  : GSVE---------IDKLLEGG------IETG-------------------SITEVFGEFRTGKTQLCHTLCVTCQ--------MPI------SEGGA--E : 123
Pseudo-nitzschia_multiseries_Rad51-A1  : GSVE---------IDKLLEGG------IETG-------------------SITEVFGEFRTGKTQLCHTLCVTCQ--------MSC------GEGGA--E : 123
Pseudo-nitzschia_multistriata_Rad51-A2 : GAVE---------IDKLLEGG------IETG-------------------SITEVFGEFRTGKTQXCHTLCVTCQ--------MPV------SDGGA--E : 123
Pseudo-nitzschia_multistriata_Rad51-A1 : GSXE---------IDKLLEGG------IETG-------------------SITEVFGEFRTGKTQLCHTLCITCQ--------MSC------GEGGA--E : 123
Seminavis_robusta_Rad51-A              : GSVE---------LDKLLEGG------IETG-------------------SITEVFGEFRTGKTQLCHTLCITCQ--------MGV------NEGGA--E :  74
Arabidopsis_thaliana_Rad51-B           : HLKG---------LDDTLCGG------IPFG-------------------VLTELVGPPGIGKSQFCMKLALSAS--------FPV------AYGGL--D : 127
Oryza_sativa_Rad51-B                   : TLRG---------LDEALHGG------IPAG-------------------KLTEVVGPSGIGKTQFCLKLALLAT--------LPE------CYGGL--N : 125
Mus_musculus_Rad51-B                   : TLCA---------LDEALHGG------VPCG-------------------SLTEITGPPGCGKTQFCIMMSVLAT--------LPT------SLGGL--E : 126
Homo_sapiens_Rad51-B                   : TLSA---------LDEALHGG------VACG-------------------SLTEITGPPGCGKTQFCIMMSILAT--------LPT------NMGGL--E : 126
Danio_rerio_Rad51-B                    : SLPA---------LDRLLHGG------LPRG-------------------ALTEVTGPSGCGKTQLCMMLSVLAT--------LPK------SLGGL--D : 122
Chlamydomonas_reinhardtii_Rad51-B      : GLPT---------LDGALRLG------VPVG-------------------SITELVGPGGVGKSQLSHMLALAVA--------MPE------ALGGL--G : 126
Micromonas_pusilla_Rad51-B             : HLAA---------LDLALKGG------VPTG-------------------SISELVGPAGAGKTQFCLTLAVAAA--------APK------SVGGL--D : 153
Ostreococcus_lucimarinus_Rad51-B       : VIED---------VDKALGGG------LRVG-------------------AVTEVVGAAGAGKTQLCLAACASAA--------APA------RVGGR--D : 120
Ectocarpus_siliculosus_Rad51-B         : GIQP---------LDDALQGG------FPTG-------------------MITELVGPAGIGKTQTCLQVAAQAC--------LPA------KLGGLGED : 129
Thalassiosira_pseudonana_Rad51-B       : GLPT---------LDSSLLGG------FPLC-------------------SISELVGRAGVGKTHLAQQLCVLAA------------------KSGF--- :  93
Phaeodactylum_tricornutum_Rad51-B      : GLEP---------LDQALRGG------VRVG-------------------TITELVGPAGVGKTQLAMQLCIMAS--------RYA-------------- : 167
Fragilariopsis_cylindrus_Rad51-B       : CLPS---------LDRILRGG------VRLG-------------------TITELVGKSGTGKTQLALQLCIAAA--------KFN-------------- : 196
Pseudo-nitzschia_multiseries_Rad51-B   : SLPS---------LDRILHGG------VRLT-------------------TVTELVGRSGVGKTQLAFQLCVTAA--------KFN-------------- : 178
Pseudo-nitzschia_multistriata_Rad51-B  : SLPS---------LDRILNGG------VRLA-------------------TVTELVGRSGVGKTQLAFQLCVTAA--------KFN-------------- : 178
Seminavis_robusta_Rad51-B              : GWTA---------LDNCLRGG------IRIG-------------------SLTEFCGRAGAGKTQLAMQLCVTAA--------ARC-------------G : 140
Arabidopsis_thaliana_Rad51-C           : SCSD---------LDNILGGG------ISCR-------------------DVTEIGGVPGIGKTQIGIQLSVNVQ--------IPR------ECGGL--G : 130
Oryza_sativa_Rad51-C                   : GSAD---------LNNILGGG------IHCK-------------------EVTEIGGVPGVGKTQLGIQLAINVQ--------IPV------EYGGL--G : 136
Mus_musculus_Rad51-C                   : FCSA---------LDNILGGG------IPLM-------------------KTTEVCGVPGVGKTQLCMQLAVDVQ--------IPE------CFGGV--A : 135
Homo_sapiens_Rad51-C                   : FCSA---------LDDILGGG------VPLM-------------------KTTEICGAPGVGKTQLCMQLAVDVQ--------IPE------CFGGV--A : 135
Danio_rerio_Rad51-C                    : FCSG---------LDDAIGGG------VPVG-------------------KTTEICGAPGVGKTQLCMQLAVDVQ--------IPV------FFGGL--G : 130
Chlamydomonas_reinhardtii_Rad51-C      : MARD---------LDALLGGG------VAAG-------------------QVTEFCGVPGVGKTQLGMQLAVNVQ--------IPR------SLSGP--E : 131
Micromonas_pusilla_Rad51-C             : FSSE---------LDALLGGG------VAAG-------------------EITELCGCPGIGKTQMCVQLCASVQ--------IPH------AFGGY--D : 128
Ostreococcus_lucimarinus_Rad51-C       : FCEA---------LDDALGGG------AASG-------------------EITELCGCPGIGKTQMAMQLSVSAQ--------TPR------AFGGL--G : 130
Ectocarpus_siliculosus_Rad51-C         : FCRE---------IDGMMGGG------VPRG-------------------ELTEVCGTPGVGKTQFGMQLAVDVQ--------IPH------QFGGV--G :  91
Phaeodactylum_tricornutum_Rad51-C      : FCRH---------VDTLLGGG------IAMG-------------------ELTEIAGPPGVGKTQWGMQLAVDAR--------LPN------TFGGV--A : 130
Thalassiosira_pseudonana_Rad51-C       : FSHP---------LDSLLGGG------IPLS-------------------ELTELVGLPGCGKTQLAMQLCVDAR--------LPR------KNGGV--E : 155
Fragilariopsis_cylindrus_Rad51-C       : FCKS---------LDKLLGGG------VSLG-------------------QVTEISGLPGTGKTQLATQLSVLAR--------LPR------EFGGV--E :  77
Pseudo-nitzschia_multiseries_Rad51-C   : FCRS---------IDDLLGGG------IELG-------------------QVTEISGLPGTGKTQLATQLAVLAR--------LPG------AFGGV--G : 182
Pseudo-nitzschia_multistriata_Rad51-C  : FCRS---------VDDLLGGG------IELG-------------------SLTEVAGLPGTGKTQLATQLAVLAR--------LPP------SFGGV--G : 186
Seminavis_robusta_Rad51-C              : FCRS---------VDSMLGGG------IALG-------------------EVTEIAGVPGSGKTQLAMQLSVDAR--------LPI------QYGGV--E : 134
Arabidopsis_thaliana_Xrcc3             : GCEI---------LDGCLRGG------ISCD-------------------SLTEIVAESGCGKTQLCLQLSLCTQ--------LPI------SHGGL--N :  73
Oryza_sativa_Xrcc3                     : GCPV---------LDRLLSGG------LPPA-------------------SVTEIAGESASGKTQLCLQLALLAP--------LSP----------L--S :  91
Mus_musculus_Xrcc3                     : GCPV---------LDQFLGGG------LPLE-------------------GITGLAGCSSAGKTQLALQLCLAVQ--------FPR------QYGGL--E : 128
Homo_sapiens_Xrcc3                     : GCPV---------LDALLRGG------LPLD-------------------GITELAGRSSAGKTQLALQLCLAVQ--------FPR------QHGGL--E : 128
Danio_rerio_Xrcc3                      : ACPV---------LDGLMRGG------LPLR-------------------GITELAGESAAGKTQFCLQLCLSVQ--------YPQ------EHGGL--N : 128
Micromonas_sp._Xrcc3                   : ACPV---------IDKWLRGG------VPTR-------------------HVTELTGEAGAAKTQLALQLLLNAQ--------LPR------EVGGL--D :  80
Ostreococcus_lucimarinus_Xrcc3         : MCPR---------IDDALRGG------VRAR-------------------HVTEIAGESASAKTTLCAQLAVRAT--------ARG-------------- :  62
Ectocarpus_siliculosus_Xrcc3           : GCPS---------LDAAFEGG------VRVQ-------------------GVTEIAGEAGAGKTQLCLQLLLQAQ--------LAP------EAGGL--- : 114
Thalassiosira_pseudonana_Xrcc3         : GCGMKLMRPRLFLFSRCSGGGGSNEQSVPIGQSTPHHQQQPNEGVWELGTGITELSGEGGSGKTQISLSLCATTA--------MTPLINDNNSQGAT--H : 230
Phaeodactylum_tricornutum_Xrcc3        : G------------LESWLS--------LARP-------------------GIYELVGEAGTGKSQTALSVCVQAASSTTGPSREPPLIAAPTTGTDVSLH : 148
Fragilariopsis_cylindrus_Xrcc3         : AVKP-----------------------ISAL-------------------ALLQIAGPAGVGKTQIALSLCADCV------------------------- :  61
Pseudo-nitzschia_multiseries_Xrcc3     : ---------------------------LPLG--------------------LTEIAGPAGAGKTQVALSLCADCV------QQHPS-------------- :  40
Pseudo-nitzschia_multistriata_Xrcc3    : LCLL---------QNPHLS--------LPIG--------------------LSELAGPAGAGKTQMALSLCADCV--------QYS----WSDNXHQ--K : 135
Seminavis_robusta_Xrcc3                : IPPP---------ARNVIASNNLSWLKLGLG--------------------ITELAGEAGSGKSQLAMSLCVQAVTMQQQTASITQ------GRSHKTPP : 159
Sulfolobus_solfataricus_Rad-A          : GSQA---------LDGLLAGG------IETR-------------------TMTEFFGEFGSGKSQLCHQLSVNVQ--------LPP------EKGGL--S : 123
Aeropyrum_pernix_Rad-A                 : GSRN---------LDELLGGG------VETK-------------------TITELFGEFGSGKTQICHQLSVNVQ--------LPE------DKGGL--E : 123
                                                            gg                                   g    gK3q
```

```
                                                    *        320         *        340         *        360         *        380         *        400
Arabidopsis_thaliana_Dmc1                 : ---GKVAYIDTE--GTFR----PDRIVP--IAERF-------------------------------------------------------------GMDP-- : 151
Oryza_sativa_Dmc1                         : ---GKVAYIDTE--GTFR----PERIVP--IAERF-------------------------------------------------------------GMDA-- : 151
Mus_musculus_Dmc1                         : ---GKIIFIDTE--NTFR----PDRLRD--IADRF-------------------------------------------------------------NVDH-- : 151
Homo_sapiens_Dmc1                         : ---GKIIFIDTE--NTFR----PDRLRD--IADRF-------------------------------------------------------------NVDH-- : 151
Danio_rerio_Dmc1                          : ---GKVIFIDTE--NTFR----PERLKD--IADRF-------------------------------------------------------------NVDH-- : 151
Ectocarpus_siliculosus_Dmc1               : -----------E--GRVR----PRRGVQ--CLHARVSFL--------------------------------------------------------------- : 139
Oryza_sativa_Rad51-A                      : ---GKALYIDAE--GTFR----PQRLLQ--IADRF-------------------------------------------------------------GLNG-- : 151
Arabidopsis_thaliana_Rad51-A              : ---GKAMYIGAE--GTFR----PQRLLQ--IADRF-------------------------------------------------------------GLNG-- : 151
Mus_musculus_Rad51-A                      : ---GKAMYIDTE--GTFR----PERLLA--VAERY-------------------------------------------------------------GLSG-- : 151
Homo_sapiens_Rad51-A                      : ---GKAMYIDTE--GTFR----PERLLA--VAERY-------------------------------------------------------------GLSG-- : 151
Danio_rerio_Rad51-A                       : ---GKAMYIDTE--GTFR----PERLLA--VAERY-------------------------------------------------------------GLVG-- : 151
Chlamydomonas_reinhardtii_Rad51-A         : ---GKAMYIDTE--GTFR----PQRLSQ--IAERY-------------------------------------------------------------GLAP-- : 151
Micromonas_sp._Rad51-A                    : ---GKAMYIDTE--GTFR----PQRLIA--IAERF-------------------------------------------------------------GMDP-- : 151
Ostreococcus_lucimarinus_Rad51-A          : ---GKCLYIDTE--GTFR----PQRLIQ--IAERF-------------------------------------------------------------NMDP-- : 151
Ectocarpus_siliculosus_Rad51-A            : ---GKAMYIDTE--GTFR----PQRLTA--IAERF-------------------------------------------------------------GLNG-- : 151
Phaeodactylum_tricornutum_Rad51-A         : ---GKAIYIDTE--GTFR----PNRLQA--IAERF-------------------------------------------------------------GLDP-- : 151
Thalassiosira_pseudonana_Rad51-A          : ---GKAIYIDTE--GTFR----PNRLQA--IAERF-------------------------------------------------------------GMDP-- : 151
Fragilariopsis_cylindrus_Rad51-A2         : ---GKAMYIDTE--GSFR----PERLKL--IAERF-------------------------------------------------------------GLDP-- : 151
Fragilariopsis_cylindrus_Rad51-A1         : ---GKAMYIDTE--GSFR----PERLKA--IAERF-------------------------------------------------------------GLDP-- : 151
Pseudo-nitzschia_multiseries_Rad51-A2     : ---GKAIYIDTE--GSFR----PERLRA--IAERF-------------------------------------------------------------GLDP-- : 151
Pseudo-nitzschia_multiseries_Rad51-A1     : ---GKAIYIDTE--GSFR----PERLKA--IAERF-------------------------------------------------------------GLDP-- : 151
Pseudo-nitzschia_multistriata_Rad51-A2    : ---GKAIYIDTE--GSFR----PERLRA--IAERF-------------------------------------------------------------GLDP-- : 151
Pseudo-nitzschia_multistriata_Rad51-A1    : ---GKAIYIDTE--GSFR----PERLKV--IAERF-------------------------------------------------------------GLDP-- : 151
Seminavis_robusta_Rad51-A                 : ---GKAIYLDTE--GSFR----PARLQA--IAERF-------------------------------------------------------------GLDP-- : 102
Arabidopsis_thaliana_Rad51-B              : ---GRVIYIDVE--SKFS----SRRVIE--MGLESFPEV----------FHLKG--------------------------------------------MA-- : 162
Oryza_sativa_Rad51-B                      : ---GRVLYIDTE--SKFS----SRRMIE--IGEKSFPQI----------FRQEG--------------------------------------------LA-- : 160
Mus_musculus_Rad51-B                      : ---GAVVYIDTE--SAFT----AERLVE--IAESRFPQY---------------------------------------------------------FNTE-- : 158
Homo_sapiens_Rad51-B                      : ---GAVVYIDTE--SAFS----AERLVE--IAESRFPRY---------------------------------------------------------FNTE-- : 158
Danio_rerio_Rad51-B                       : ---SGVIYIDTE--SAFS----AERLVE--MAQSRFPEF---------------------------------------------------------FSVK-- : 154
Chlamydomonas_reinhardtii_Rad51-B         : ---AGVVYIDTE--RKFS----APRLQE--MVHARVAEA-----AAAAGPQAAHVLQP--------------------------------------LAVQ-- : 172
Micromonas_pusilla_Rad51-B                : ---GGVVFIDTE--QKFS----GVRLAE--IARAKFPSV--------YGDGDGDGAPSEADA----------------------------------RASL-- : 200
Ostreococcus_lucimarinus_Rad51-B          : ---GGVIYVDAE--RKFS----GARLAE--IAREKFPGA--------FEDEE----------------------------------------------SV-- : 155
Ectocarpus_siliculosus_Rad51-B            : ---AGVVYLDTE--RKFS----PDRLVE--IASERHPGH-----YGEFSTE---------------------------------------------KGSM-- : 168
Thalassiosira_pseudonana_Rad51-B          : ---GGAVYIDTE--KKLS----LVRLRE--LAMER------------------------------------------------------------------- : 117
Phaeodactylum_tricornutum_Rad51-B         : ---QGCVYVDTE--KKLS----VARLRE--IALQRSSRV-----PDTNTHGEFLYPSDTTLVESTVDISATSRNC---------------------FRSP-- : 230
Fragilariopsis_cylindrus_Rad51-B          : ---QGAIYIDSE--KKLS----LPRLRE--MSERWKAHG-----LQERRRQREQSQSNTIGVGGVGVGTDHHHPGRQFANKAAANSFSYNQGSYGNSSYP : 280
Pseudo-nitzschia_multiseries_Rad51-B      : ---QGAIYIDTE--KKMS----LERLGEPSTSEELLEVL---------------------------------------------------------DALE-- : 212
Pseudo-nitzschia_multistriata_Rad51-B     : ---QGAXYIDTX--KKMS----LERLGE--MSEQRRLIG-----NNHESRDEDDMGM---------------------------------------YKST-- : 223
Seminavis_robusta_Rad51-B                 : ---QGTVYLDTE--QKLS----LTRLQE--IVQHQHGNG--QLRKRPRTHEYDDGTTTNMEPTTV-------------------------------AEQQ-- : 196
Arabidopsis_thaliana_Rad51-C              : ---GKAIYIDTE--GSFM----VKRALQ--IAEACVEDM-----EEYTGYMHKHFQANQV------------------------------------QMKP-- : 178
Oryza_sativa_Rad51-C                      : ---GKAVYIDTE--GSFM----VERVYQ--IAEGCISDI-----LEYFPHCHDKAPAGQE------------------------------------KLKP-- : 184
Mus_musculus_Rad51-C                      : ---GEAVFIDTE--GSFM----VDRVVS--LATACIQHL-----HLIAGTHTEEEHQKALK-----------------------------------DFTL-- : 184
Homo_sapiens_Rad51-C                      : ---GEAVFIDTE--GSFM----VDRVVD--LATACIQHL-----QLIAEKHKGEEHRKALE-----------------------------------DFTL-- : 184
Danio_rerio_Rad51-C                       : ---GKALYIDTE--GSFL----VQRVAD--MAEAAVQHC-----TLLAEDTEQKGALE--------------------------------------ELNV-- : 176
Chlamydomonas_reinhardtii_Rad51-C         : ---GQAVYIDTE--GSFM----AERCAD--IAEGAVRHV-----QSILEKKASMGQPELLHDGER-------------------------------PFTL-- : 184
Micromonas_pusilla_Rad51-C                : ---GEAVYVDTE--GSFM----AERAEE--IAEATARHL-----RSVSNASPEDAGMSDAIA----------------------------------SFTA-- : 178
Ostreococcus_lucimarinus_Rad51-C          : ---GEAVYVDTE--GSFT----AERAMD--MAEALAEHL-----GRCAKRCEDEDARREMEAALE-------------------------------TCAP-- : 183
Ectocarpus_siliculosus_Rad51-C            : ---GGALYIDTE--GSLT----VERLSQ--LCSAVVEHL-----QKIARNKRKQGVPDLESA----------------------------------VPTQ-- : 141
Phaeodactylum_tricornutum_Rad51-C         : ---GETVYVDTE--GSFS----PERCHD--MATSLVQHI-----EAGRRRRQEKGQKLQPMPA---------------------------------WFAP-- : 181
Thalassiosira_pseudonana_Rad51-C          : ---GCSVVVDSE--GSWTGVHGGERLWT--MAKSLVEHV-----RNSAGRRMEARRARAADFGGGAVDEELVPA----------------------WFTP-- : 221
Fragilariopsis_cylindrus_Rad51-C          : ---GRTLYVDAE--GSFV----PERAYS--MADALCDHV-----RATTRKRRRRQQQQHSQRSQR-------------------------------NHHV-- : 130
Pseudo-nitzschia_multiseries_Rad51-C      : ---GRTLYVDAE--GSFV----AERAWA--MAGALCRHV-----GGTAQRQRKRRLRHNNNNNSSSNHPA--------------------------DFTT-- : 240
Pseudo-nitzschia_multistriata_Rad51-C     : ---GRTLYVDAE--GSFS----ADRAWA--MAGALCGHV-----GGTARKRRQKRRGSKTKTKRSSQSATTSDEKEHEHEHEHEHEHETTCLPP--GFTP-- : 266
Seminavis_robusta_Rad51-C                 : ---GTAVYIDTE--GSFS----PERCYT--MASALVDHV-----KKSAEKRKTQCPPTPQ------------------------------------WFQP-- : 182
Arabidopsis_thaliana_Xrcc3                : ---GSSLYLHSE--FPFP----FRRLHQ--LSHTFHQSN-----PSIYANYNDNPC---------------------------------------------- : 113
Oryza_sativa_Xrcc3                        : ---ASCLFLHSD--LPFP----LRRLRG--LA----------------------------------------------------------------PKSR-- : 116
Mus_musculus_Xrcc3                        : ---AGAVYICTE--DAFP----SKRLWQ--LIAQQRRLR---------------------------------------------------------TDAP-- : 160
Homo_sapiens_Xrcc3                        : ---AGAVYICTE--DAFP----HKRLQQ--LMAQQPRLR---------------------------------------------------------TDVP-- : 160
Danio_rerio_Xrcc3                         : ---SGAVYICTE--DSFP----IKRLRQ--LITQQ--------------PRLRPDLPPA-------------------------------------LIHS-- : 166
Micromonas_sp._Xrcc3                      : ---GAAVYVHTE--GRAP----LARLRQ--MISKRQIYS--------AH-----------------------------------------------LPPD-- : 114
Ostreococcus_lucimarinus_Xrcc3            : ---GAAVYVHTD--GPAP----TALMRR--IASSA----------RFVAEACDGD-----------------------------------------EDAA-- : 100
Ectocarpus_siliculosus_Xrcc3              : ---GGKSYVLTCGEGDFP----SRRLRQ--MASTYQSRH---------------------------------------------------------GVKP-- : 148
Thalassiosira_pseudonana_Xrcc3            : ---CTSIYITMG--EGIPSSKIAMRLEQ--MVRARRENM-----HEQCNNAHKVNISNNGNFFNTG--------------------------RE--EEEI-- : 290
Phaeodactylum_tricornutum_Xrcc3           : LIPCRAIYISLK--AQNNVVQIVKRLEQ--MVLSRQEQR-----PASTPDQMKAPT----------------------------------------RSPP-- : 199
Fragilariopsis_cylindrus_Xrcc3            : --------------LQHP----SRRLQS--MIQSRILTT-----TGNNEDRRQRNHHQEADD----------------------------------DRII-- : 102
Pseudo-nitzschia_multiseries_Xrcc3        : ---AKAVYVVALGGSGRSSLHKAAKRLRD--MLEARTRHQ--ACGGSANGNGNGKGNGNSNSNTNSNTNSNT-------------------------NEWV-- : 108
Pseudo-nitzschia_multistriata_Xrcc3       : ---KKAVYIQLGGSSRFL-QTASRRLKA--XLESRIAMR-----DPCGGGIHPSGSPGDATDATAHNPAARHKRHHHRQRQRQQQRQQQQPDVSEDRV-- : 222
Seminavis_robusta_Xrcc3                   : ---CRAVYISLSG-GQASLSRIAYRMEQ--IANAQKQQSCKDNSATNYPICNQQLPGSALHGPTTTSEDQT-------------------------NAAS-- : 228
Sulfolobus_solfataricus_Rad-A             : ---GKAVYIDTE--GTFR----WERIEN--MAKAL-------------------------------------------------------------GLDI-- : 151
Aeropyrum_pernix_Rad-A                    : ---GKAVYIDTE--GTFR----WERIEQ--MARGV-------------------------------------------------------------GLDP-- : 151
                                                        e            r
```

```
                                               *       420         *       440         *       460         *       480         *       500
Arabidopsis_thaliana_Dmc1              : --------------------------------GAVL------DNIIYARA--YTYEHQYNLL-LGLAAKMSEEP-------------------------- : 184
Oryza_sativa_Dmc1                      : --------------------------------NAVL------DNIIYARA--YTYEHQYNLL-LGLAAKMAEEP-------------------------- : 184
Mus_musculus_Dmc1                      : --------------------------------EAVL------DNVLYARA--YTSEHQMELL-DYVAAKFHEEA-------------------------- : 184
Homo_sapiens_Dmc1                      : --------------------------------DAVL------DNVLYARA--YTSEHQMELL-DYVAAKFHEEA-------------------------- : 184
Danio_rerio_Dmc1                       : --------------------------------EAVL------DNVLYARA--YTSEHQMELL-DFVAAKFHEEG-------------------------- : 184
Ectocarpus_siliculosus_Dmc1            : ----------------------------------FC------RRWWWWWKMSCLPLPPEKRTFQSIAALCLTEG-------------------------- : 173
Oryza_sativa_Rad51-A                   : --------------------------------ADVL------ENVAYARA--YNTDHQSRLL-LEAASMMIETR-------------------------- : 184
Arabidopsis_thaliana_Rad51-A           : --------------------------------ADVL------ENVAYARA--YNTDHQSRLL-LEAASMMIETR-------------------------- : 184
Mus_musculus_Rad51-A                   : --------------------------------SDVL------DNVAYARG--FNTDHQTQLL-YQASAMMVESR-------------------------- : 184
Homo_sapiens_Rad51-A                   : --------------------------------SDVL------DNVAYARA--FNTDHQTQLL-YQASAMMVESR-------------------------- : 184
Danio_rerio_Rad51-A                    : --------------------------------SDVL------DNVAYARA--FNTDHQTQLL-YQASAMMTESR-------------------------- : 184
Chlamydomonas_reinhardtii_Rad51-A      : --------------------------------EDVL------NNVAYARA--HNTEHQMRLL-QEAAGMMADSR-------------------------- : 184
Micromonas_sp._Rad51-A                 : --------------------------------NAVL------DNVAYAKA--HNTEHQSELL-VAAAGMMAEAR-------------------------- : 184
Ostreococcus_lucimarinus_Rad51-A       : --------------------------------SAVL------DNVAYAKA--HNVEHQSELL-LAAAGMMAETR-------------------------- : 184
Ectocarpus_siliculosus_Rad51-A         : --------------------------------DDVL------ENVAYARA--HNSEQQMDLL-KMASAMMAEDR-------------------------- : 184
Phaeodactylum_tricornutum_Rad51-A      : --------------------------------TVAL------ENVAYARA--HNSEHQAELL-KLAAAIMSQDR-------------------------- : 184
Thalassiosira_pseudonana_Rad51-A       : --------------------------------SVAL------ENVAYARA--HNSEHQMELL-KMAAAIMSQDR-------------------------- : 184
Fragilariopsis_cylindrus_Rad51-A2      : --------------------------------VATL------ENVAYARA--HNSEHQMELL-KVAAAIMSQDR-------------------------- : 184
Fragilariopsis_cylindrus_Rad51-A1      : --------------------------------KLTL------ENVAIARA--HNSEHQMELL-KVAAAIMAQDR-------------------------- : 184
Pseudo-nitzschia_multiseries_Rad51-A2  : --------------------------------AVVL------ENVACARA--HNSEHQMELL-KVAAAIMSQDR-------------------------- : 184
Pseudo-nitzschia_multiseries_Rad51-A1  : --------------------------------AVAL------ENVACARA--HNSEHQMELL-KVAAAIMAQDR-------------------------- : 184
Pseudo-nitzschia_multistriata_Rad51-A2 : --------------------------------AVVL------ENVACARA--HNSEHQMELL-KVAAAIMSQDR-------------------------- : 184
Pseudo-nitzschia_multistriata_Rad51-A1 : --------------------------------TVAL------ENVXCARA--QNSEHQMELL-KTAAAIMAQDR-------------------------- : 184
Seminavis_robusta_Rad51-A              : --------------------------------NLAL------ENVAYARA--QNSEHQMELL-KMAAAIMSQDR-------------------------- : 135
Arabidopsis_thaliana_Rad51-B           : --------------------------------QEMA------GRILVLRP--TSLANFTESI-QELKNSILQNQ-------------------------- : 195
Oryza_sativa_Rad51-B                   : --------------------------------QKMA------GRILVLRP--TSLSEFTKSL-EQMKVTLLQHD-------------------------- : 193
Mus_musculus_Rad51-B                   : --------------------------------EKLL---LTSSRVHLCRE--LTCEGLLQRL-ESLEEEIISKG-------------------------- : 194
Homo_sapiens_Rad51-B                   : --------------------------------EKLL---LTSSKVHLYRE--LTCDEVLQRI-ESLEEEIISKG-------------------------- : 194
Danio_rerio_Rad51-B                    : --------------------------------ERLL---EMAARVHLFRE--LTCQDVLKRL-ERLEEDIIACR-------------------------- : 190
Chlamydomonas_reinhardtii_Rad51-B      : --------------------------------GEVL------RRVAVSTP--GSTEQLMQTV-ENLQHTVLQYR-------------------------- : 205
Micromonas_pusilla_Rad51-B             : --------------------------------TTLT------SRVLVLTP--STLSEILQRL-NGLEEALIDHG-------------------------- : 233
Ostreococcus_lucimarinus_Rad51-B       : --------------------------------HALA------RRVHVVTP--TSLTDLNKRL-DALEEAIIDHK-------------------------- : 188
Ectocarpus_siliculosus_Rad51-B         : --------------------------------ERLL------NQVTVFPV--DNSAALVDRL-ETLQARMIESN-------------------------- : 201
Thalassiosira_pseudonana_Rad51-B       : ---------------------------------------------------------------NTMQRLP------------------------------ : 124
Phaeodactylum_tricornutum_Rad51-B      : --------------------------------QEVL------DNVTVHSP--SNIDELFGAL-SEVEDELFSRN-------------------------- : 263
Fragilariopsis_cylindrus_Rad51-B       : TPNSQQSFHSNTNYDNDECNNDEDAMCTFKSTELVL------GNLTLHTP--GSSSELLDVL-DALEDEILQRN--------------QTAGQAFPVSTA : 357
Pseudo-nitzschia_multiseries_Rad51-B   : --------------------------------YEILIRNQTAGQAFLESG--AESAGTTTNA-TSTHTHTHTHT-------------------------- : 251
Pseudo-nitzschia_multistriata_Rad51-B  : --------------------------------QLVL------NNLTVHQP--ENTDELLQVL-DGLEYEILIRNQNAGQAFFQSGASTDINT-------- : 274
Seminavis_robusta_Rad51-B              : --------------------------------QQVL------DNVTVLSP--TSMPDLMTAL-LQIEELVLDAQ-------------------------- : 229
Arabidopsis_thaliana_Rad51-C           : --------------------------------EDIL------ENIFYFRV--CSYTEQIALV-NHLEKFISENK-------------------------- : 211
Oryza_sativa_Rad51-C                   : --------------------------------ESFL------ADIYYFRI--CSYTEQIAVI-NYLEKFLGEHK-------------------------- : 217
Mus_musculus_Rad51-C                   : --------------------------------ENIL------SHIYYFRC--HDYTELLAQV-YLLPDFLSDHP-------------------------- : 217
Homo_sapiens_Rad51-C                   : --------------------------------DNIL------SHIYYFRC--RDYTELLAQV-YLLPDFLSEHS-------------------------- : 217
Danio_rerio_Rad51-C                    : --------------------------------EKIL------SNLFLVRC--HDYVKLLAEV-YLLPDFLSEHP-------------------------- : 209
Chlamydomonas_reinhardtii_Rad51-C      : --------------------------------ENVM------RGIYLFRV--HDHVEQLGLV-NMLPRFLEQYS-------------------------- : 217
Micromonas_pusilla_Rad51-C             : --------------------------------ERML------ERVHLFRC--HEVTELLAVL-EALPAYVKKHR-------------------------- : 211
Ostreococcus_lucimarinus_Rad51-C       : --------------------------------EAML------RGIHLFRC--HEVTELLAAL-ETLGEFIAERP-------------------------- : 216
Ectocarpus_siliculosus_Rad51-C         : --------------------------------EAFL------GGIHVWRL--HDHAEQLAAV-RTLPEFLVAHP-------------------------- : 174
Phaeodactylum_tricornutum_Rad51-C      : --------------------------------DTIL------QGIHVYRV--HDEAAQTSVL-YSLPKFLQDRQ-------------------------- : 214
Thalassiosira_pseudonana_Rad51-C       : --------------------------------ESIL------EGIHIFRV--HDEPSQTCTL-YSLPQFLMEQE-------------------------- : 254
Fragilariopsis_cylindrus_Rad51-C       : --------------------------------EDIL------DSIDVFRV--HDETALLATL-YSLQKYNNSTA-------------------------- : 163
Pseudo-nitzschia_multiseries_Rad51-C   : --------------------------------EGIL------GSIDVFRV--HDETALLATL-YSLQHHIEIAN---------------------ENNAN : 278
Pseudo-nitzschia_multistriata_Rad51-C  : --------------------------------EGIL------ASIDVFRV--HDEAALLATL-YSLGDRIGEAS-------------------------- : 299
Seminavis_robusta_Rad51-C              : --------------------------------NTIM------QGIHVFRA--HDEAAQMSIV-MGLPDFLRDQE-------------------------- : 215
Arabidopsis_thaliana_Xrcc3             : ------------------------------------------DHVFVQNV--HSVDHLFDIM-PRIDGFVGNSK-------------------------- : 142
Oryza_sativa_Xrcc3                     : --------------------------------PDLL------DHVLVAAA--HSPSDLISLL-SRAQRLLAHPG-------------------------- : 149
Mus_musculus_Xrcc3                     : --------------------------------EELIEKIRFSNHIFIEHA--ADVDTLLECVSKKVPILLSRGM-------------------------- : 200
Homo_sapiens_Xrcc3                     : --------------------------------GELLQKLRFGSQIFIEHV--ADVDTLLECVNKKVPVLLSRGM-------------------------- : 200
Danio_rerio_Xrcc3                      : --------------------------------LRFS------DNIYIEHA--ADLEALQVCVSQRVPVLLKRGL-------------------------- : 200
Micromonas_sp._Xrcc3                   : --------------------------------HDPL------DYVYLVKTLEEDPESLWDAL-ASVADVLTDPP-------------------------- : 149
Ostreococcus_lucimarinus_Xrcc3         : --------------------------------TAAL------ERVYVVKAL-GDADALRETL-TAVSAVLRAPV-------------------------- : 134
Ectocarpus_siliculosus_Xrcc3           : --------------------------------EKLM------EGVCIQNA--KTLDDQMMIIMEHLPHIMRSQN-------------------------- : 182
Thalassiosira_pseudonana_Xrcc3         : --------------------------------KQIL------SRIWLLSI--RNEEEFIELVESHLPQMLEQQR----------QYQQQLQQQTAMNNHN : 340
Phaeodactylum_tricornutum_Xrcc3        : --------------------------------CTIL------QRILTRAA--WNAEQLTQVL-DELPVLLKSGT-------------------------- : 232
Fragilariopsis_cylindrus_Xrcc3         : --------------------------------HDCL------TRVLVRWI--CNSDELMDLLTTNLDRLLHHHP-------------------------- : 136
Pseudo-nitzschia_multiseries_Xrcc3     : --------------------------------HECL------TRVLVHWV--CNTEELTEVLQTRLPALLRKHH-------------------------- : 142
Pseudo-nitzschia_multistriata_Xrcc3    : --------------------------------HDCL------SRIIVHWI--CNSEDLMELLRTKLPRLLRMNN-------------------------- : 256
Seminavis_robusta_Xrcc3                : --------------------------------ASVL------PQILTHSV--RNHEDLFALLREDLPHCLKGDS------------PDTTNPASFQPDNA : 276
Sulfolobus_solfataricus_Rad-A          : --------------------------------DNVM------NNIYYIRA--INTDHQIAIV-DDLQELVSKDP-------------------------- : 184
Aeropyrum_pernix_Rad-A                 : --------------------------------DEVM------KNIYWIRA--INSHHQIAIV-DKLFTMVKNDN-------------------------- : 184
```

```
                                                          *        520         *         540         *         560         *         580         *         600
Arabidopsis_thaliana_Dmc1              : -----------------FRILIVDSIIALFR------VD---------------FTG----------RGELAD-----RQQKLAQMLSRLIKIAEEFNVA : 231
Oryza_sativa_Dmc1                      : -----------------FRLLIVDSVIALFR------VD---------------FSG----------RGELAE-----RQQKLAQMLSRLTKIAEEFNVA : 231
Mus_musculus_Dmc1                      : ---------------GIFKLLIIDSIMALFR------VD---------------FSG----------RGELAE-----RQQKLAQMLSRLQKISEEYNVA : 233
Homo_sapiens_Dmc1                      : ---------------GIFKLLIIDSIMALFR------VD---------------FSG----------RGELAE-----RQQKLAQMLSRLQKISEEYNVA : 233
Danio_rerio_Dmc1                       : ---------------GVFKLLIIDSIMALFR------VD---------------FSG----------RGELAE-----RQQKLAQMLSRLQKISEEYNVA : 233
Ectocarpus_siliculosus_Dmc1            : ----------------PIRVIIVDSVIALFR------VD---------------FSG----------RGDLSV-----RQQKLGRHLSSLLQMASDLNVA : 221
Oryza_sativa_Rad51-A                   : -----------------FALMIVDSATALYR------TD---------------FSG----------RGELSA-----RQMHMAKFLRSLQKLADEFGVA : 231
Arabidopsis_thaliana_Rad51-A           : -----------------FALLIVDSATALYR------TD---------------FSG----------RGELSA-----RQMHLAKFLRSLQKLADEFGVA : 231
Mus_musculus_Rad51-A                   : -----------------YALLIVDSATALYR------TD---------------YSG----------RGELSA-----RQMHLARFLRMLLRLADEFGVA : 231
Homo_sapiens_Rad51-A                   : -----------------YALLIVDSATALYR------TD---------------YSG----------RGELSA-----RQMHLARFLRMLLRLADEFGVA : 231
Danio_rerio_Rad51-A                    : -----------------YALLIVDSATALYR------TD---------------YSG----------RGELSA-----RQGHLGRFLRMLLRLADEFGVA : 231
Chlamydomonas_reinhardtii_Rad51-A      : -----------------FSLVVVDSATALYR------TE---------------FNG----------RGELSI-----RQILLGRFLRALQNIADEYGVA : 231
Micromonas_sp._Rad51-A                 : -----------------FGVIIVDSVTNLFR------TE---------------YEG----------RGELSA-----RQMHLGKFLRHLTRLADEFGVA : 231
Ostreococcus_lucimarinus_Rad51-A       : -----------------FSLMIIDSVTNLYR------TE---------------YEG----------RGELSA-----RQMHLGKFLRQLARLADEFGVA : 231
Ectocarpus_siliculosus_Rad51-A         : -----------------YALLVIDSATALYR------TD---------------YCG----------RGELSE-----RQMQLAQFLRQLTRMAEEFGVA : 231
Phaeodactylum_tricornutum_Rad51-A      : -----------------YALLVVDSATALFR------TD---------------YTG----------RGELSE-----RQMQMAQFLRQLTRLAEEFGVA : 231
Thalassiosira_pseudonana_Rad51-A       : -----------------YALIVVDSATALFR------TD---------------YSG----------RGELSE-----RQMQLAQFLRQLTRLAEEFGVA : 231
Fragilariopsis_cylindrus_Rad51-A2      : -----------------YALLVVDSATALYR------TD---------------YTG----------RGELSE-----RQMQMGQFLRQLTRLAEEFGVA : 231
Fragilariopsis_cylindrus_Rad51-A1      : -----------------YALLVVDSATALYR------TD---------------YTG----------RGELSE-----RQMQMGQFLRQLTRLAEEFGVA : 231
Pseudo-nitzschia_multiseries_Rad51-A2  : -----------------YALLVVDSATALFR------TD---------------YTG----------RGELSE-----RQMQMGQFLRQLTRLAEEFGVA : 231
Pseudo-nitzschia_multiseries_Rad51-A1  : -----------------YALLVVDSATALYR------TD---------------YQG----------RGELSE-----RQMQMGQFLRQLTRLAEEFGVA : 231
Pseudo-nitzschia_multistriata_Rad51-A2 : -----------------YALLVVDSATALFR------TD---------------YTG----------RGELSE-----RQMQMGQFLRQLTRLAEEFGVA : 231
Pseudo-nitzschia_multistriata_Rad51-A1 : -----------------YALLVVDSATALYR------TD---------------YQG----------RGELSE-----RQMQMGQFLRQLTRLAEEFGVA : 231
Seminavis_robusta_Rad51-A              : -----------------YALLVVDSATALFR------TD---------------YTG----------RGELSE-----RQMQMGQFLRQLTRLAEEFGVA : 182
Arabidopsis_thaliana_Rad51-B           : -----------------VKLLVIDSMTALLS------GE----------------------------NKPGAQ-----RQPQLGWHISFLKSLAEFSRIP : 239
Oryza_sativa_Rad51-B                   : -----------------VKLLVVDSMAALMS------SE---------------IEK----------SA-TGL-----RQHPLRWALSFLKSIAEFSQIP : 239
Mus_musculus_Rad51-B                   : -----------------VKLVIVDSIASVVR------KE---------------FDP--------KLQGNIKE-----RNKFLGKGASLLKYLAGEFSIP : 243
Homo_sapiens_Rad51-B                   : -----------------IKLVILDSVASVVR------KE---------------FDA--------QLQGNLKE-----RNKFLAREASSLKYLAEEFSIP : 243
Danio_rerio_Rad51-B                    : -----------------AGLVILDSVASVVR------KE---------------FDT--------SLPGNLTH-----RSNFLGQEAAVLKYLSQEFCIP : 239
Chlamydomonas_reinhardtii_Rad51-B      : -----------------ARLVVVDSIAALAR------TEYGNPSSSGASGSVAGGGG--------GLVGSIMD-----RQQVLGRIAASLKALAESLRIP : 269
Micromonas_pusilla_Rad51-B             : -----------------VRLLVVDSMAALAR------AE---------------FG-----------RGQLQQ-----RQELLGQIASVLKQQAERLHLA : 279
Ostreococcus_lucimarinus_Rad51-B       : -----------------VRLVIIDSIAHLAR------AE---------------FG-----------REKVVQ-----RQSALGAVASTLKRHAEKHALA : 234
Ectocarpus_siliculosus_Rad51-B         : -----------------VRLIVLDSIAALAR------RD---------------FAR----------EDTLS------RQELLTRQAAVLKSLAYTFSAV : 247
Thalassiosira_pseudonana_Rad51-B       : -----------------VKLVVIDSIAAPVR------RD---------------FDMMSSSSTSKFSPNAATQ-----RASFIFQVARKLKQLTHDHHLA : 181
Phaeodactylum_tricornutum_Rad51-B      : -----HQSVGSSNAKFPVRLLVLDSIAAPAR------RD---------------FGA-----------GSAPE-----LASTVIKIAQTLKRLADQHHLV : 321
Fragilariopsis_cylindrus_Rad51-B       : SVVENINTRNNVRGKYPVRLLIVDSIASPMR------RD---------------FGT-----------DSAPQ-----RASAIFQCAQKLKRLADQLHLA : 420
Pseudo-nitzschia_multiseries_Rad51-B   : ------HNKTAVRGKYPVRLLIVDSIAAPMR------RD---------------FGT----------DS-APQ-----RAAAIFQCAQKLKRLADQLHLA : 308
Pseudo-nitzschia_multistriata_Rad51-B  : -----SLSNTAVRGKYPVRLLIVDSIAAPMR------RD---------------YGT----------DS-APQ-----RAASIFQCAQKLKRLADQLHLA : 332
Seminavis_robusta_Rad51-B              : --------HDNSNNKLPIGLVIVDSIAAPAK------RG----------------------------AESAPE-----RAAALFQCAQRLKQMADQLQLA : 282
Arabidopsis_thaliana_Rad51-C           : ----------------DVKVVIVDSITFHFR------QD---------------YDD-------------LAQ-----RTRVLSEMALKFMKLAKKFSLA : 256
Oryza_sativa_Rad51-C                   : ----------------DVRIVIIDSVTFHFR------QD---------------FDD-------------MAL-----RTRVLSGLSLKLMKLSKAYNLA : 262
Mus_musculus_Rad51-C                   : ----------------KVQLVIIDGIAFPFR------HD---------------LED-------------LSL-----RTRLLNGLAQQMISLANNHRLA : 262
Homo_sapiens_Rad51-C                   : ----------------KVRLVIVDGIAFPFR------HD---------------LDD-------------LSL-----RTRLLNGLAQQMISLANNHRLA : 262
Danio_rerio_Rad51-C                    : ----------------EVRLVVIDSIAFPFR------HD---------------FED-------------LSQ-----RTRLLNGLAQQLIQLATQHRVA : 254
Chlamydomonas_reinhardtii_Rad51-C      : ----------------QVRLIVIDSVTFHFR------QD---------------FPD-------------MAQ-----RTRVVTGMAQQLISLAQTHNVA : 262
Micromonas_pusilla_Rad51-C             : -----------------VRLVVVDSVAFHFR------QD---------------FRD-------------MAL-----RTTILAKMTQRLQQLASENALA : 255
Ostreococcus_lucimarinus_Rad51-C       : ----------------KVRLVVIDSVAFHFR------QD---------------FQD-------------MAL-----RTTILSKMTNRLMSIATTQQVA : 261
Ectocarpus_siliculosus_Rad51-C         : ----------------EVKLVVMDSVAFHFR------HA---------------FQD-------------MSV-----RTRMLSRMAQQLNEVAQAHSLA : 219
Phaeodactylum_tricornutum_Rad51-C      : ------------EAGTPVRLVVVDSMAFHHR------AA----------------PP----------NSDFVG-----RTRSLTSQAAFLTNLAAQSGIA : 265
Thalassiosira_pseudonana_Rad51-C       : ------------EKGHPVKLLVIDSLAFHYR------VANTSTSSNNNGGGGGSNNN----------RNNSLS-----NTHNLTRMSTFLTELASEFDMA : 321
Fragilariopsis_cylindrus_Rad51-C       : ------NNNSSSSSSLPVKLIVIDSIAFHFR------AV--------------TPTD----------SSYYVQ-----RTKTLTALAAYLGDLASKHDLA : 222
Pseudo-nitzschia_multiseries_Rad51-C   : NASTTSSTSTTTTNYLPVKLIVIDSIAFHFR------AV--------------TPTD----------SSYYLQ-----RTKTLAALASFLGDLAHTHDLA : 343
Pseudo-nitzschia_multistriata_Rad51-C  : ----------STKGGLPVRLVVVDSIAFHFR------AA--------------SPTD----------PSYYLR-----RTKTLAGLAGYLGDLARRHGLA : 354
Seminavis_robusta_Rad51-C              : ------------QQGSPVKAVVVDSIAFHYR------CA--------------PPGG----------N--YLA-----RTRSLSTVASLLSDMATKFDLA : 266
Arabidopsis_thaliana_Xrcc3             : -------------TRFPLKLIVLDSVAALFR------SE---------------FDN---------TPSDLKK-----RSSLFFKISGKLKQLASKFDLA : 194
Oryza_sativa_Xrcc3                     : -------------RLPPVRLILVDSIASLFR------AD---------------FDA---------SPADLKR-----RSALFFRISAKLKELAHRHRCV : 201
Mus_musculus_Xrcc3                     : -----------------ARLVVVDSIAAPFR------CE---------------FHL-----------QASAI-----RAKLLLSLGATLRRLSSTFRSP : 246
Homo_sapiens_Xrcc3                     : -----------------ARLVVIDSVAAPFR------CE---------------FDS----------QASAP------RARHLQSLGAMLRELSSAFQSP : 246
Danio_rerio_Xrcc3                      : -----------------VRLLVVDSVAALFR------SE---------------FQA-----------DEAVQ-----RSRHLLAFSSTLHRLSHTYAAP : 246
Micromonas_sp._Xrcc3                   : -----------GGPRRPVRLIVVDSVSSPFR------ET--------------DAST----------KEGVFE-----RTGILSRVAALISEYAHRADVA : 203
Ostreococcus_lucimarinus_Xrcc3         : ------------SEDAVVRMIVVDSIAAPFR------DD-------------ATNGG----------WTYAAK-----RAGALHKLTMLLKEYATKHDVA : 188
Ectocarpus_siliculosus_Xrcc3           : -----------------VKLVVIDSIAALFR------SD---------------MGR---------GRGDIGE-----RSRLLGRLSQQMKRLGDRHGAA : 230
Thalassiosira_pseudonana_Xrcc3         : HHHQNEQQPADPISSARIGIIAFDGIAGFFRF-----SD-----------PLLSFTT----------SKSSNSLFHRQRSSKLFQISSHLRRLSDVYDIP : 414
Phaeodactylum_tricornutum_Xrcc3        : -----------------VRVLVLDSIADMFRTS----ED---------------TDG---------TRSQQSSHHHAARSAILFGLAARLKKLSDVFDVP : 287
Fragilariopsis_cylindrus_Xrcc3         : ----------------TISMVVLDGIANLFRFLPEYESQSSSQQQQQQQQQQQNYNN----------NNPWHH-----RAITFFQISNLCKELSSKYEVP : 205
Pseudo-nitzschia_multiseries_Xrcc3     : ---------------PAVSVVVLDGIANLFR------LRPEHGSHGNDNGSGSGNGI----------ASPWHD-----RAVALFRLSNACKAVSSRFGVP : 206
Pseudo-nitzschia_multistriata_Xrcc3    : ---------------RTVSLVVLDGIANLFRLLPE--QD------HFCSTRGYDIGD----------KNPWHH-----RAVTFFQISNLCKELSSVFEVP : 318
Seminavis_robusta_Xrcc3                : QLPGGFPGDTTQQPQHPVALVVLDSIADLFR------LG---------------NSD----------ESDVEKGAILKRSYLMFELSAILKALSDQYQVP : 345
Sulfolobus_solfataricus_Rad-A          : ----------------SIKLIVVDSVTSHFR------AE---------------YPG----------REILAV-----RQQKLNKHLHQLTRLAEVYDIA : 232
Aeropyrum_pernix_Rad-A                 : -----------------IKLVVVDSVTSHFR------AE---------------FPG----------RENLAM-----RQQLLNRHLHQLMRLADIFNVA : 231
                                                            6  Ds                                                    r
```

```
                                                  *        620         *        640         *        660         *        680         *        700
Arabidopsis_thaliana_Dmc1               : VYMTNQVIADP---------------------------G----------GGMFISDPKKPAGGHVLAHAATIRLLF----RKGKGD-------------- : 276
Oryza_sativa_Dmc1                       : VYITNQVIADPG-------------------------------------GGMFITDLKKPAGGHVLAHAATIRLML----RKGKGE-------------- : 276
Mus_musculus_Dmc1                       : VFVTNQMTADP--------------------------GA----------TMTFQADPKKPIGGHILAHASTTRISL----RKGRGE-------------- : 279
Homo_sapiens_Dmc1                       : VFVTNQMTADP--------------------------GA----------TMTFQADPKKPIGGHILAHASTTRISL----RKGRGE-------------- : 279
Danio_rerio_Dmc1                        : VFVTNQMTADP--------------------------GA----------GMTFQADPKKPIGGHILAHASTTRISL----RKGRAE-------------- : 279
Ectocarpus_siliculosus_Dmc1             : VVLVNQCMSDP--------------------------------------GAMAMFATVKPVGGHVLAHASTTRVHL----KKGRGE-------------- : 265
Oryza_sativa_Rad51-A                    : VVITNQVVAQV--------------------------DG----------SAMFAGPQIKPIGGNIMAHASTTRLAL----RKGRGE-------------- : 277
Arabidopsis_thaliana_Rad51-A            : VVITNQVVAQV--------------------------DG----------SALFAGPQFKPIGGNIMAHATTTRLAL----RKGRAE-------------- : 277
Mus_musculus_Rad51-A                    : VVITNQVVAQV--------------------------DG----------AAMFAADPKKPIGGNIIAHASTTRLYL----RKGRGE-------------- : 277
Homo_sapiens_Rad51-A                    : VVITNQVVAQV--------------------------DG----------AAMFAADPKKPIGGNIIAHASTTRLYL----RKGRGE-------------- : 277
Danio_rerio_Rad51-A                     : VVITNQVVAQV--------------------------DG----------AAMFSADPKKPIGGNILAHASTTRLYL----RKGRGE-------------- : 277
Chlamydomonas_reinhardtii_Rad51-A       : VVVTNQVVANP-------------------------DGA----------GAMFAGPQTKPIGGNIMAHATTTRLSV----RKGRGE-------------- : 278
Micromonas_sp._Rad51-A                  : VVVTNQVVANP-------------------------DGN----------AMFAGANALKPIGGNIMAHASTTRIAL----RKGRGE-------------- : 278
Ostreococcus_lucimarinus_Rad51-A        : VIVSNQVVANP--------------------------EG----------GPFAGANALKPIGGNIMAHASTTRLAL----RKGRGE-------------- : 277
Ectocarpus_siliculosus_Rad51-A          : VVLTNQVVANP---------------------------D----------GMSFAKDSTKPIGGNIIAHASTTRLRL----RKARGD-------------- : 276
Phaeodactylum_tricornutum_Rad51-A       : VFITNQVVANP---------------------------D----------GMSFAKDSTKPIGGNIMAHASTTRLRL----RKGRGD-------------- : 276
Thalassiosira_pseudonana_Rad51-A        : VFMTNQVVANP---------------------------D----------GMSFAKDSTKPIGGNIVAHASTTRLRL----RKGRGE-------------- : 276
Fragilariopsis_cylindrus_Rad51-A2       : VFITNQVVSDP---------------------------G----------AMSFAKDSTKPIGGNIIAHASTTRLRL----RKGRGD-------------- : 276
Fragilariopsis_cylindrus_Rad51-A1       : VFITNQVVSDP---------------------------G----------AMSFAKDATKPIGGNIIAHAATTRLRL----RKGRGD-------------- : 276
Pseudo-nitzschia_multiseries_Rad51-A2   : VFITNQVVANP---------------------------D----------GMSFAKDATKPIGGNIIAHASTTRLRL----RKGRGE-------------- : 276
Pseudo-nitzschia_multiseries_Rad51-A1   : VFITNQVVANPD-------------------------------------GMSFAKDSTKPIGGNIIAHASTTRLRL----RKGRGE-------------- : 276
Pseudo-nitzschia_multistriata_Rad51-A2  : VFITNQVVANPD-------------------------------------GMSFAKDAXKPIGGNIIAHASTTRLRL----RKGRGE-------------- : 276
Pseudo-nitzschia_multistriata_Rad51-A1  : VFITNQVVANP---------------------------D----------GMSFAKDSTKPIGGNIIAHASXTRLRL----RKGRGE-------------- : 276
Seminavis_robusta_Rad51-A               : VFITNQVVANP---------------------------D----------GMSFAKDSTKPIGGNIIAHASTTRLRL----RKGRGE-------------- : 227
Arabidopsis_thaliana_Rad51-B            : IVVTNQVRSQNRDE---------TSQYSFQAKVKDEFKD----------NTKTYDSHLVAALGINWAHAVTIRLVL----EAKSG--------------- : 301
Oryza_sativa_Rad51-B                    : VVVTNQVRSQSNDD---------GYRYSFEVEKKYD-SN----------NAEGFESHLVAALGIQWAHAVTIRLVF----EAHSG--------------- : 300
Mus_musculus_Rad51-B                    : VILTNQITTHL------------------SGALPSQADLVSPADDLSLSEGTSGSSCLVAALGNTWGHCVNTRLIL----QYLDSE-------------- : 307
Homo_sapiens_Rad51-B                    : VILTNQITTHL------------------SGALASQADLVSPADDLSLSEGTSGSSCVIAALGNTWSHSVNTRLIL----QYLDSE-------------- : 307
Danio_rerio_Rad51-B                     : VVLTNQITTHV--------------GEKLHCPQWNQTDA----------SFEEDSGFVTAALGNTWSHSVNTRLIV----QYEDSE-------------- : 297
Chlamydomonas_reinhardtii_Rad51-B       : VLVTNQVTTRI------------------GGGGGGGPGP----------PGSAANGTLTAALGAKWAHCVNLRLVL----QRLQE--------------- : 322
Micromonas_pusilla_Rad51-B              : VFVTNQVTTRIGAAAGHDTGPGAGREEGDGGGIGGGGGGAERDAGSRDAGSGSGSGSVTAALGTKWAHCVNTRLVL----EGAAGGGDADAAGGG----- : 370
Ostreococcus_lucimarinus_Rad51-B        : VLAVNQVTTKI----------------GTFARHASDGGD----------DVADESSGITAALGTKWAHCVNTRIAL----EVLED--------------- : 289
Ectocarpus_siliculosus_Rad51-B          : VLVTNQVTTSF------------------YPTSGLQGGS----------RQSIASHTNSSSREGKSAFETETRMVM----RRG----------------- : 298
Thalassiosira_pseudonana_Rad51-B        : VVVVNQVGSGG----------------SDFRNNNPQRNN----------TLDIRDGEFTASLGTAWQYCVTTRIVM----EHEDDPHKLAQEQDQCH--- : 248
Phaeodactylum_tricornutum_Rad51-B       : VVVINQVGSSI-------------------------LGT----------DAVIDQTGIRPALGTSWHHCVSTRVLF----EFEADLVSSSSLSNSDGPLR : 382
Fragilariopsis_cylindrus_Rad51-B        : VVVINQVGSDS-----------------------------------------------RAALGTSWHHCVSTRLML----ETATATSDANKIDLDRSNGN : 469
Pseudo-nitzschia_multiseries_Rad51-B    : VVVINQVGAAGGGGVGVGD----GRAGNGIGIGTNAPGM----------SSEQQRIPVRAALGTSWHHCVSTRLLL----ETSTVGVVPTAPELSGFDNG : 390
Pseudo-nitzschia_multistriata_Rad51-B   : IVVINQVGAASVDSNAM------KNRSSENKNDISGFGL----------NAEQHPSNVRAALGTSWHHCVSTRLLL----EALNIPNFMPSPELSSLGNT : 412
Seminavis_robusta_Rad51-B               : ILVVNQVALER--------------------------------------SNFGSGVRVKAALGTSWHHCVSTRIMM----EQQQQQLEQQPQQQSGSPFV : 340
Arabidopsis_thaliana_Rad51-C            : VVLLNQVTTKF----------------------------------------SEGSFQLALALGDSWSHSCTNRVIL----YW-NGD-------------- : 297
Oryza_sativa_Rad51-C                    : VVLLNQVTTKF----------------------------------------TEGSFQLTLALGDSWSHSCTNRLIL----YW-NGN-------------- : 303
Mus_musculus_Rad51-C                    : VILTNQMTTKI----------------------------------------DKNQALLVPALGESWGHAATIRLIF----HWEQK--------------- : 303
Homo_sapiens_Rad51-C                    : VILTNQMTTKI----------------------------------------DRNQALLVPALGESWGHAATIRLIF----HWDRK--------------- : 303
Danio_rerio_Rad51-C                     : VVLTNQMTTRV----------------------------------------SNGQSKLVPALGESWGHAATQRLIL----HW-EGQ-------------- : 295
Chlamydomonas_reinhardtii_Rad51-C       : VVLMNQVTTKV---------------------------------------LEGGGSKLVPALGESWGHAASTRVML----TWGPDN-------------- : 305
Micromonas_pusilla_Rad51-C              : VVTVNQVTVKP--------------------------------------DPRGGGARLVPALGESYAHACTTRIIL----SWEDD--------------- : 298
Ostreococcus_lucimarinus_Rad51-C        : VVTVNQVTVKP---------------------------------------QRDGSARLVPALGESYAHACTTRVIL----SWEND--------------- : 303
Ectocarpus_siliculosus_Rad51-C          : VVLVNQMTTKV---------------------------M----------TGHRGESSLVPALGESWAHAATNRLLL----LW-KGQ-------------- : 263
Phaeodactylum_tricornutum_Rad51-C       : VVAINQMTTKM---------------------------------------TTSEASKQVPALGESWAHAVTTRILL----SRPISDTNG----------- : 311
Thalassiosira_pseudonana_Rad51-C        : VVALNHLTTKI-------------------------EKD----------ENGNSTTKLVPALGESWAHSVTSRLMIDHYHRVNNGSTLPIEMEE------ : 380
Fragilariopsis_cylindrus_Rad51-C        : VVVINQMTTKVSN----------NYNNNSHQSSFSSSSI----------PSSSSSTVLVPALGESWAHATATRLIL----SNEEQYVVPVTATGADGNSD : 298
Pseudo-nitzschia_multiseries_Rad51-C    : VVAINQMTTKVVMD---------GNNYGNGTGNGNGNGN----------GTGLSVSSVVPALGESWAHATATRIIL----SRDEGSHDNEHNDGDRGDDE : 420
Pseudo-nitzschia_multistriata_Rad51-C   : VVAINQMTTRMRH----------GHGHGHGQSSNGSGSG----------SSNPSDALVVPALGESWAHATATRLVLSREXQWQGGTRGRNENENENHNDC : 434
Seminavis_robusta_Rad51-C               : VIVINQMTTKV------------------------ATTA----------NTTDTESVLVPALGESWAHATTTRLLI----ASETNNNN------------ : 316
Arabidopsis_thaliana_Xrcc3              : IVITNQVTDLV-------------ETSDGLSGLRIGNLR----------YLYSSGRRVVPSLGLAWANCVNSRFFI----SRSDGSIVKDRSEKDENCSS : 267
Oryza_sativa_Xrcc3                      : VVVTNQVVDVV---------------------EGEAGNT----------VAWSSGRRVSPALGIAWANCVNTRLFL----TRDADGRGCA---------- : 256
Mus_musculus_Xrcc3                      : VLCINQVTDMV--------------------EDQQSVSR----------SLGASEERLSPALGITWANQLLMRLMV----DRTHEDDVTTGLPRSP---- : 308
Homo_sapiens_Xrcc3                      : VLCINQVTEAM--------------------EEQGAAHG----------PLGFWDERVSPALGITWANQLLVRLLA----DRLREEEAALGCP------- : 305
Danio_rerio_Xrcc3                       : VLCVNQVTDVV--------------------DGPNPGRC----------DYGLVGSKVLPALGIAWANQVMVRLML----RRLAGQVKSDSRSCA----- : 307
Micromonas_sp._Xrcc3                    : VVVTNHVSDAV--------RDDGRGGFDSRQRRLFGPSG----------DLRSSGRPVQPALGLFWANCVNTRVFL----SRTGGSAGGYDNDGRVGLVD : 281
Ostreococcus_lucimarinus_Xrcc3          : VVVTNHVVDSV---------RENGTGAHGDGAVATRAMG----------EFTTSGRRVVPALGLMWSNCVNARLFLTRRATRGQGYVVGGDDGDDGNAGV : 269
Ectocarpus_siliculosus_Xrcc3            : FVVVNQVTARF----------GSSTIGGGGGRGQGTMGV----------VGGSGGGGSVPAMGLLWSQCINASPSS--IPSWDVTTLDGN---------- : 298
Thalassiosira_pseudonana_Xrcc3          : MLITNQVTASI------------------AEGGGDGGGV----------VASTQEHGVVPALGLIWSNCVTTRYIL----QRKEATVATGANPTINYDGK : 482
Phaeodactylum_tricornutum_Xrcc3         : VLVINQVA--------------------------------------------LSGVWTKPALGLSWAHCIDVRYILTRQERGGDAGVVFG---------- : 333
Fragilariopsis_cylindrus_Xrcc3          : FVVINECTTKIPNDTTT------TTTTANTGGSTFRRCQ----------QQQQQLPVLEPALGLAWSQCVNCRFFV----RRLGVMTTCNNNNNSSSDQQ : 285
Pseudo-nitzschia_multiseries_Xrcc3      : VLVLNGCTSRI-----------------PAAVAPTTTSG----------YNGGGRAVLEPALGLAWSQCVNSSWFV----VRKPGTETGTGAARAAYEDG : 275
Pseudo-nitzschia_multistriata_Xrcc3     : FVVLNECTSRI--------------------------SS----------ESTTNSPVLEPAMGLAWSQCVNCSFFI----CRESRNNLNTAEXATXTTTS : 378
Seminavis_robusta_Xrcc3                 : ILVINQVTCDL------------------------------------------NRKQNLPALGLSWANCVNTSYVI----RRSRSAAGGNSSSTRADVNQ : 399
Sulfolobus_solfataricus_Rad-A           : VIITNQVMARP---------------------------------------DMFYGDPTVAVGGHTLYHVPGIRIQL----KKSRGN-------------- : 275
Aeropyrum_pernix_Rad-A                  : VVITNQVMARP---------------------------------------DVFYGDPTQAVGGHVLGHAPGVRVYL----KKSRGN-------------- : 274
                                              N                                                         g         r
```

```
                                              *       720         *       740         *       760         *       780         *       800
Arabidopsis_thaliana_Dmc1                : ----------------------------------------------------------------------------------------------TRVCKV : 282
Oryza_sativa_Dmc1                        : ----------------------------------------------------------------------------------------------QRVCKI : 282
Mus_musculus_Dmc1                        : ----------------------------------------------------------------------------------------------LRIAKI : 285
Homo_sapiens_Dmc1                        : ----------------------------------------------------------------------------------------------LRIAKI : 285
Danio_rerio_Dmc1                         : ----------------------------------------------------------------------------------------------LRIAKI : 285
Ectocarpus_siliculosus_Dmc1              : ----------------------------------------------------------------------------------------------ERVAKL : 271
Oryza_sativa_Rad51-A                     : ----------------------------------------------------------------------------------------------ERICKV : 283
Arabidopsis_thaliana_Rad51-A             : ----------------------------------------------------------------------------------------------ERICKV : 283
Mus_musculus_Rad51-A                     : ----------------------------------------------------------------------------------------------TRICKI : 283
Homo_sapiens_Rad51-A                     : ----------------------------------------------------------------------------------------------TRICKI : 283
Danio_rerio_Rad51-A                      : ----------------------------------------------------------------------------------------------TRICKI : 283
Chlamydomonas_reinhardtii_Rad51-A        : ----------------------------------------------------------------------------------------------NRVVKI : 284
Micromonas_sp._Rad51-A                   : ----------------------------------------------------------------------------------------------NRIAKI : 284
Ostreococcus_lucimarinus_Rad51-A         : ----------------------------------------------------------------------------------------------NRVMKV : 283
Ectocarpus_siliculosus_Rad51-A           : ----------------------------------------------------------------------------------------------NRICQV : 282
Phaeodactylum_tricornutum_Rad51-A        : ----------------------------------------------------------------------------------------------NRICTV : 282
Thalassiosira_pseudonana_Rad51-A         : ----------------------------------------------------------------------------------------------NRICTV : 282
Fragilariopsis_cylindrus_Rad51-A2        : ----------------------------------------------------------------------------------------------NRIMQV : 282
Fragilariopsis_cylindrus_Rad51-A1        : ----------------------------------------------------------------------------------------------NRIMTV : 282
Pseudo-nitzschia_multiseries_Rad51-A2    : ----------------------------------------------------------------------------------------------NRIMSV : 282
Pseudo-nitzschia_multiseries_Rad51-A1    : ----------------------------------------------------------------------------------------------NRIMTV : 282
Pseudo-nitzschia_multistriata_Rad51-A2   : ----------------------------------------------------------------------------------------------NRIMSV : 282
Pseudo-nitzschia_multistriata_Rad51-A1   : ----------------------------------------------------------------------------------------------NRIMTV : 282
Seminavis_robusta_Rad51-A                : ----------------------------------------------------------------------------------------------NRICTV : 233
Arabidopsis_thaliana_Rad51-B             : ----------------------------------------------------------------------------------------------QRIIKV : 307
Oryza_sativa_Rad51-B                     : ----------------------------------------------------------------------------------------------HRYIKV : 306
Mus_musculus_Rad51-B                     : ----------------------------------------------------------------------------------------------RRQILI : 313
Homo_sapiens_Rad51-B                     : ----------------------------------------------------------------------------------------------RRQILI : 313
Danio_rerio_Rad51-B                      : ----------------------------------------------------------------------------------------------RRQIVI : 303
Chlamydomonas_reinhardtii_Rad51-B        : ----------------------------------------------------------------------------------------------RRFLKV : 328
Micromonas_pusilla_Rad51-B               : ----------------------------------------------------------------------------------------------TRVVKV : 376
Ostreococcus_lucimarinus_Rad51-B         : ----------------------------------------------------------------------------------------------RRALKI : 295
Ectocarpus_siliculosus_Rad51-B           : ----------------------------------------------------------------------------------------------QRAATF : 304
Thalassiosira_pseudonana_Rad51-B         : ----------------------------------------------------------------------------------------------QRKATI : 254
Phaeodactylum_tricornutum_Rad51-B        : GRAP------------------------------------------------------------------------------------------SRQLSV : 392
Fragilariopsis_cylindrus_Rad51-B         : NEFGINLNSRQSMQSDHHRV--------------------------------------------------------------------------TRKIAV : 495
Pseudo-nitzschia_multiseries_Rad51-B     : NGNGSHNDSISRQTTTLPTHRV------------------------------------------------------------------------TKKIVV : 418
Pseudo-nitzschia_multistriata_Rad51-B    : NRNGFQWEKAIEPTHLI-----------------------------------------------------------------------------TKKIAV : 435
Seminavis_robusta_Rad51-B                : REN-------------------------------------------------------------------------------------------ARTATI : 349
Arabidopsis_thaliana_Rad51-C             : ----------------------------------------------------------------------------------------------ERYAYI : 303
Oryza_sativa_Rad51-C                     : ----------------------------------------------------------------------------------------------ERYGFL : 309
Mus_musculus_Rad51-C                     : ----------------------------------------------------------------------------------------------QRFATL : 309
Homo_sapiens_Rad51-C                     : ----------------------------------------------------------------------------------------------QRLATL : 309
Danio_rerio_Rad51-C                      : ----------------------------------------------------------------------------------------------RRLASL : 301
Chlamydomonas_reinhardtii_Rad51-C        : ----------------------------------------------------------------------------------------------ERHAQM : 311
Micromonas_pusilla_Rad51-C               : ----------------------------------------------------------------------------------------------TRTAYL : 304
Ostreococcus_lucimarinus_Rad51-C         : ----------------------------------------------------------------------------------------------ERVAFV : 309
Ectocarpus_siliculosus_Rad51-C           : ----------------------------------------------------------------------------------------------ERFAEL : 269
Phaeodactylum_tricornutum_Rad51-C        : ----------------------------------------------------------------------------------------------VRTCTL : 317
Thalassiosira_pseudonana_Rad51-C         : ----------------------------------------------------------------------------------------------ARVCTL : 386
Fragilariopsis_cylindrus_Rad51-C         : DGEDYNRGPAVLQQ--------------------------------------------------------------------------------VRTCTL : 318
Pseudo-nitzschia_multiseries_Rad51-C     : TEHDRDPTSSVRTRTR------------------------------------------------------------------------------TRTCSL : 442
Pseudo-nitzschia_multistriata_Rad51-C    : ENDNDYDRPAVLRP--------------------------------------------------------------------------------VRICSL : 454
Seminavis_robusta_Rad51-C                : ----------------------------------------------------------------------------------------------NRTCTL : 322
Arabidopsis_thaliana_Xrcc3               : SVSRSA----------------------------------------------------------------------------------------KRRLDI : 279
Oryza_sativa_Xrcc3                       : ----------------------------------------------------------------------------------------------RRRMNV : 262
Mus_musculus_Xrcc3                       : ----------------------------------------------------------------------------------------------VRTLRV : 314
Homo_sapiens_Xrcc3                       : ----------------------------------------------------------------------------------------------ARTLRV : 311
Danio_rerio_Xrcc3                        : ----------------------------------------------------------------------------------------------PRKLEV : 313
Micromonas_sp._Xrcc3                     : SGGTAVV---------------------------------------------------------------------------------------RRHASV : 294
Ostreococcus_lucimarinus_Xrcc3           : ----------------------------------------------------------------------------------------------ARTLHV : 275
Ectocarpus_siliculosus_Xrcc3             : ----------------------------------------------------------------------------------------------LAFASF : 304
Thalassiosira_pseudonana_Xrcc3           : DGSENGRCLEPLRNSTVLEQLDLSLVGRHESPDIQRPVVLSDIVIPILESIIGTEVNSVKNLQLPMKWRWGVDLHSNECPRLLDFLEMLPQLLNSREQSC : 582
Phaeodactylum_tricornutum_Xrcc3          : ----------------------------------------------------------------------------------------------RRVTLD : 339
Fragilariopsis_cylindrus_Xrcc3           : QQHQHQHQHQKSSSSTATSTTMLTPTTNGSNNNKSNSNNNKVYY--------------------------------------------------RRLLHC : 335
Pseudo-nitzschia_multiseries_Xrcc3       : ANAARATTTTTTTATATNGGRGC-----------------------------------------------------------------------PRVLRC : 304
Pseudo-nitzschia_multistriata_Xrcc3      : TTIAESIQTSDSVQV------------------------------------------------------------------------------CRRVLQC : 400
Seminavis_robusta_Xrcc3                  : G---------------------------------------------------------------------------------------------LRHIFL : 406
Sulfolobus_solfataricus_Rad-A            : ----------------------------------------------------------------------------------------------RRIARV : 281
Aeropyrum_pernix_Rad-A                   : ----------------------------------------------------------------------------------------------KRIARV : 280
```

```
                                                      *        820          *        840
Arabidopsis_thaliana_Dmc1              : YDA-PNLAEAE------------------ASFQITQGGIADAKD : 307
Oryza_sativa_Dmc1                      : FDA-PNLPEGE------------------AVFQVTSGGIMDAKD : 307
Mus_musculus_Dmc1                      : YDS-PEMPENE------------------ATFAITAGGIGDAKE : 310
Homo_sapiens_Dmc1                      : YDS-PEMPENE------------------ATFAITAGGIGDAKE : 310
Danio_rerio_Dmc1                       : FDS-PHMPENE------------------ATFAITAGGITDAKD : 310
Ectocarpus_siliculosus_Dmc1            : FDS-PSMPEAE------------------CPFKITNGGITDTD- : 295
Oryza_sativa_Rad51-A                   : ISS-PCLAEAE------------------ARFQIASEGVADVKD : 308
Arabidopsis_thaliana_Rad51-A           : ISS-PCLPEAE------------------ARFQISTEGVTDCKD : 308
Mus_musculus_Rad51-A                   : YDS-PCLPEAE------------------AMFAINADGVGDAKD : 308
Homo_sapiens_Rad51-A                   : YDS-PCLPEAE------------------AMFAINADGVGDAKD : 308
Danio_rerio_Rad51-A                    : YDS-PCLPEAE------------------AMFAINADGVGDAKD : 308
Chlamydomonas_reinhardtii_Rad51-A      : IAS-PSLPERE------------------ANFAIGQEGVTDAKD : 309
Micromonas_sp._Rad51-A                 : ACS-PVLPESE------------------AQFSISELGIEDAKD : 309
Ostreococcus_lucimarinus_Rad51-A       : VCS-PVLPESE------------------AQFSISEFGIEDAKD : 308
Ectocarpus_siliculosus_Rad51-A         : FDS-PTLAESE------------------CQFSIGPVGVEDPKD : 307
Phaeodactylum_tricornutum_Rad51-A      : FDS-PTLPEAD------------------AQFAVGAQGVCDAQD : 307
Thalassiosira_pseudonana_Rad51-A       : FDS-PTLPEAD------------------AQFAVGPSGICDATD : 307
Fragilariopsis_cylindrus_Rad51-A2      : FDS-PSLPEAD------------------CQFAISSSGIADATE : 307
Fragilariopsis_cylindrus_Rad51-A1      : YDS-PSLPEAD------------------ASFALSGAGVCDATE : 307
Pseudo-nitzschia_multiseries_Rad51-A2  : YDS-PSLPEAD------------------CQFALSNAGVCDATD : 307
Pseudo-nitzschia_multiseries_Rad51-A1  : FDS-PSLPESD------------------AQFAVSGAGVCDATD : 307
Pseudo-nitzschia_multistriata_Rad51-A2 : YDS-PSLPEAD------------------CQFALSNAGVCDATD : 307
Pseudo-nitzschia_multistriata_Rad51-A1 : XDS-PSLPEAD------------------AQFAVSAXGVCDATD : 307
Seminavis_robusta_Rad51-A              : YDS-PTLPEAD------------------AQFAIGPQGVCDPTE : 258
Arabidopsis_thaliana_Rad51-B           : AKS-PMSPPLA------------------FPFHITSAGISLLSD : 332
Oryza_sativa_Rad51-B                   : AKS-PMTPAVA------------------FPFTVESSGIILLSD : 331
Mus_musculus_Rad51-B                   : AKS-PLAAFTS------------------FVYTIKGEGLVLQGH : 338
Homo_sapiens_Rad51-B                   : AKS-PLAPFTS------------------FVYTIKEEGLVLQAY : 338
Danio_rerio_Rad51-B                    : AKS-PVAPFAV------------------LSYTIQKEGIRLEEN : 328
Chlamydomonas_reinhardtii_Rad51-B      : AKS-PSCANVV------------------LEYVIGPTGLQEVPR : 353
Micromonas_pusilla_Rad51-B             : VKS-PRCALAG------------------FEYEVRAGGVVVDGD : 401
Ostreococcus_lucimarinus_Rad51-B       : VKS-PLAPLTS------------------FEYRVDASGIRVSGK : 320
Ectocarpus_siliculosus_Rad51-B         : V---PRALP--------------------YFARASLPHLFGLEV : 325
Thalassiosira_pseudonana_Rad51-B       : TKS-LVSKRAE------------------VAFQLTSQGLCEV-- : 277
Phaeodactylum_tricornutum_Rad51-B      : IKS-NLTGPGKP-----------------IVFDLTLFGIVIKA- : 417
Fragilariopsis_cylindrus_Rad51-B       : VKS-NRTGFSE------------------AGFEISTMGIVEDSI : 520
Pseudo-nitzschia_multiseries_Rad51-B   : VKS-NRTALGE------------------TRFAITSTGIVED-- : 441
Pseudo-nitzschia_multistriata_Rad51-B  : VKS-NTTALGE------------------TQFAITTAGIIEDRP : 460
Seminavis_robusta_Rad51-B              : VKS-NLVGLQS------------------VKYQIVGAGVVDAVE : 374
Arabidopsis_thaliana_Rad51-C           : DKS-PSLPSAS------------------ASYTVTSRGLRNSSS : 328
Oryza_sativa_Rad51-C                   : DKS-PSLPVAS------------------APYAVTVKGVRDAVN : 334
Mus_musculus_Rad51-C                   : YKS-PSQKEST------------------IPFQITPQGFRDAVV : 334
Homo_sapiens_Rad51-C                   : YKS-PSQKECT------------------VLFQIKPQGFRDTVV : 334
Danio_rerio_Rad51-C                    : YKS-PSQMEAT------------------VQYQITVQGFRDSPD : 326
Chlamydomonas_reinhardtii_Rad51-C      : IKS-PHLPLGD------------------AAFAVTADGLRSLPR : 336
Micromonas_pusilla_Rad51-C             : YKS-PRLPQGR------------------ARYTVTEGGIRDVRG : 329
Ostreococcus_lucimarinus_Rad51-C       : TKS-PRLPQAR------------------ARYAVTAGGIRDVRG : 334
Ectocarpus_siliculosus_Rad51-C         : LKS-PRLRRKT------------------VPFSVVGVGIRDTQS : 294
Phaeodactylum_tricornutum_Rad51-C      : VKS-PRLASGS------------------ADYQILQCGIRGVDA : 342
Thalassiosira_pseudonana_Rad51-C       : VKS-PHKPPGT------------------ALFAITNKGIRGVPS : 411
Fragilariopsis_cylindrus_Rad51-C       : IKS-AHRPTGT------------------AQFHILEMGIRDAPD : 343
Pseudo-nitzschia_multiseries_Rad51-C   : IKS-SHRATGT------------------ATYRILEDGIRDVPR : 467
Pseudo-nitzschia_multistriata_Rad51-C  : VKS-SHRPTGR------------------ALYRILEEGIRDVVR : 479
Seminavis_robusta_Rad51-C              : TKS-PNRPSGV------------------STFKILEAGIRDIDR : 347
Arabidopsis_thaliana_Xrcc3             : VFS-PYLPGSS------------------CEFMITREGICAVQA : 304
Oryza_sativa_Xrcc3                     : AFA-PHLPERA------------------CEFVIRRDGVFGVEP : 287
Mus_musculus_Xrcc3                     : LFA-PHLPLSS------------------CCYTVSGEGIRGMPG : 339
Homo_sapiens_Xrcc3                     : LSA-PHLPPSS------------------CSYTISAEGVRGTPG : 336
Danio_rerio_Xrcc3                      : VFA-PHLPRSS------------------CLCGVWEEGVRGIPD : 338
Micromonas_sp._Xrcc3                   : VFS-SHLPSTAPAWGGALDDGWGSSAVRPCEFEVREDGVWGVEA : 337
Ostreococcus_lucimarinus_Xrcc3         : VYA-PHLPESS------------------VDFVVREDGAWDVAA : 300
Ectocarpus_siliculosus_Xrcc3           : ATC-CTVPSVS------------------YFY------------ : 317
Thalassiosira_pseudonana_Xrcc3         : SLC-RRQPLETEQLIGTNNLGDCHRIQHKTCY-VCVEHVCNTCN : 624
Phaeodactylum_tricornutum_Xrcc3        : ASS-SHATGQH------------------KAFFIRADGVVAG-- : 362
Fragilariopsis_cylindrus_Xrcc3         : LKA-PHISSEHAK----------------LEFTIDHSGVIPIQN : 362
Pseudo-nitzschia_multiseries_Xrcc3     : RKA-PHVSDSSG-----------------AEFWIDRSGAVATAF : 330
Pseudo-nitzschia_multistriata_Xrcc3    : LRA-SHISSEYSR----------------AEFWIDRSGVHPYEH : 427
Seminavis_robusta_Xrcc3                : AKSANHAVDQN------------------ASFVIDSGGVRMQET : 432
Sulfolobus_solfataricus_Rad-A          : VDA-PHLPEGE------------------VVFALTEEGIRDAEE : 306
Aeropyrum_pernix_Rad-A                 : VDA-PHLPEGE------------------TVFAITEWGIRDPE- : 304
                                                                             g
```
