# Supplementary material for: Identification of the meiotic toolkit in diatoms and exploration of meiosis-specific SPO11 and RAD51 homologs in the sexual species Pseudo-nitzschia multistriata and Seminavis robusta
Source: BMC Genomics. 2015 Nov 14;16:930. doi: 10.1186/s12864-015-1983-5 (PMC4647503; doi:10.1186/s12864-015-1983-5)
Supplement: Additional file 5: Table S1. — Overview of phylogenetic distribution of core meiotic proteins among eukaryotes [11, 72, 133–142]. (DOC 235 kb) [file 12864_2015_1983_MOESM5_ESM.doc]

**Table S1. Overview of phylogenetic distribution of core meiotic proteins among eukaryotes**

|  | |  | | **Diaphoretickes** | | | | | | | | | | | | | | **Amorphea** | | | | | | |
| --- | --- | --- | --- | --- | --- | --- | --- | --- | --- | --- | --- | --- | --- | --- | --- | --- | --- | --- | --- | --- | --- | --- | --- | --- |
|  | | **Excavates** | | **Alveolates** | | **Stramenopiles** | | | | | | | | | **Archaeplastida** | | | **Opisthokonts** | | | | | | |
|  | |  | | Ciliates | Dino-flagelates | Diatoms | | | | | | Eustigmatophyceae | Brown algae | Oomycetes | Red algae | Plants | | Animals | | | | Fungi | | |
| **Name of the Species** | | *Giardia* | *Trichomonas vaginalis* | *Tetrahymena thermophila* | *Symbiodinium minutum*  [11] | ***Thalassiosira pseudonana*** | ***Phaeodactylum tricornutum*** | ***Fragilariopsis cylindrus*** | ***Pseudo-nitzschia multiseries*** | ***Pseudo-nitzschia multistriata*** | ***Seminavis robusta*** | *Nannochloropsis gaditana* [132] | *Ectocarpus siliculosus* | *Phytophthora sojae* | *Cyanidioschyzon merolae* | *Arabidopsis thaliana* [133, 134] | *Oryza sativa* [135] | *Homo sapiens* [136] | *Danio rerio* | *Drosophila melanogaster* [137] | *Caenorhabditis elegans* [138] | *Saccharomyces cerevisiae* [139, 140] | *Neurospora*  *crassa* | *Schizosaccharom-yces pombe* |
| **DNA replication and chromosome maintenance** | Smc1 | P | P(2) | - | P | P | P | P | P | P | P | P | P | P | P | P | P | P(2) | P(2) | P | P | P | P | P |
|  | Smc2 | P | P | P | P | P | P | P | P | P | P | P | P | P | P | P(2) | P | P | P | P | P | P | P | P |
|  | Smc3 | P | P(3) | P | P(4) | P | P | P | P | P | P | P | P | P | P | P | P | P | P | P | P | P | P | P |
|  | Smc4 | P | P(2) | P | P | P | P | P | P | P | P | - | P | P | P | P | P | P | P | P | P(2) | P | P | P |
|  | Smc5 | P | P(2) | - | - | P | P | P | P | P | P | P | P | - | - | P | P | P | P | P | P | P | P | P |
|  | Pds5 | - | P | - | - | P | P | P | P | P | P | P | P | P | P | P | P | P(2) | P(2) | P | P | P | P | P |
|  | Scc3 | - | P(2) | - | - | P | P | P | P | P | P | P | P | P | P | P | P | P(3) | P(3) | P(2) | P | P | P | P(2) |
|  | **Rec8*** | **-** | **-** | **P**  [72] | **-** | **-** | **-** | **-** | **-** | **-** | **-** | **-** | **-** | **-** | **P** | **P** | **P** | **P** | **P** | **P** | **P(3)** | **P** | **P** | **P** |
|  | Rad21 | - | P | - | P | P | P | P | P | P | P | P | P | P | P | P(3) | P(3) | P | P | P | P(2) | P | P | P |
| **DSB**  **formation** | **Spo11-1*** | **P** | **P** | **P** | **P** | **-** | **-** | **-** | **-** | **-** | **-** | **-** | **-** | **-** | **-** | **P** | **P** | **P** | **P** | **P** | **P** | **P** | **P** | **P** |
|  | **Spo11-2*** | **-** | **-** | **-** | **-** | **P** | **P** | **P** | **P** | **P** | **P** | **P** | **P** | **P** | **P** | **P** | **P** | **-** | **-** | **-** | **-** | **-** | **-** | **-** |
|  | Spo11-3 | - | - | - | - | P | P | P | P | P | P | P | P | P | P | P | P | - | - | - | - | - | - | - |
|  | |  | | **Diaphoretickes** | | | | | | | | | | | | | | **Amorphea** | | | | | | |
|  | | **Excavates** | | **Alveolates** | | **Stramenopiles** | | | | | | | | | **Archaeplastida** | | | **Opisthokonts** | | | | | | |
|  | |  | | Ciliates | Dino-flagelates | Diatoms | | | | | | Eustigmatophyceae | Brown algae | Oomycetes | Red algae | Plants | | Animals | | | | Fungi | | |
| **Name of the Species** | | *Giardia* | *Trichomonas vaginalis* | *Tetrahymena thermophila* | *Symbiodinium minutum*  [11] | ***Thalassiosira pseudonana*** | ***Phaeodactylum tricornutum*** | ***Fragilariopsis cylindrus*** | ***Pseudo-nitzschia multiseries*** | ***Pseudo-nitzschia multistriata*** | ***Seminavis robusta*** | *Nannochloropsis gaditana* [132] | *Ectocarpus siliculosus* | *Phytophthora sojae* | *Cyanidioschyzon merolae* | *Arabidopsis thaliana* [133, 134] | *Oryza sativa* [135] | *Homo sapiens* [136] | *Danio rerio* | *Drosophila melanogaster* [137] | *Caenorhabditis elegans* [138] | *Saccharomyces cerevisiae* [139, 140] | *Neurospora*  *crassa* | *Schizosaccharom-yces pombe* |
| **DNAdamage sensing** | Rad50 | P | P(2) | P | P(2) | P | P | P | P | P | P | P | P | P | P | P | P | P | P | P | P | P | P | P |
|  | Mre11 | P | P | P | P(2) | P | P | P | P | P | P | P | P | P | P | P | P(2) | P | P | P | P | P | P | P |
|  | Xrs2/Nbs1 | - | - | - | - | - | - | - | - | - | - | - | - | - | - | P | P | P | P | P | P | P | P | P |
| **Homology searching/**  **Crossoverregulation** | **Dmc1*** | **P(2)** | **P** | **P**[141] | **P(2)** | **-** | **-** | **-** | **-** | **-** | **-** | **P** | **P** | **P** | **P** | **P** | **P** | **P** | **P** | **-** | **-** | **P** | **-** | **-** |
|  | **Hop1*** | **P** | **P** | **P** | **-** | **-** | **-** | **-** | **-** | **-** | **-** | **-** | **P** | **-** | **-** | **P** | **P** | **P** | **P** | **-** | **P** | **P** | **-** | **-** |
|  | **Hop2*** | **P** | **P(2)** | **P** | **P** | **-** | **-** | **-** | **-** | **-** | **-** | **-** | **P** | **-** | **P** | **P** | **P** | **P** | **P** | **-** | **-** | **P** | **-** | **-** |
|  | **Mnd1*** | **P** | **P** | **P** | **P** | **P** | **P** | **P** | **P** | **P** | **P** | **P** | **P** | **P** | **P** | **P** | **P** | **P** | **P** | **-** | **-** | **P** | **-** | **-** |
|  | **Mer3*** | **P** | **P** | **-** | **-** | **P** | **P** | **P** | **P** | **P** | **P** | **P** | **P** | **-** | **P** | **P** | **P** | **P** | **P** | **-** | **-** | **P** | **P** | **-** |
|  | **Msh4*** | **-** | **P** | **P**[140] | **P** | **P** | **P** | **P** | **P** | **P** | **P** | **P** | **P** | **P** | **P** | **P** | **P** | **P** | **P** | **P** | **P** | **P** | **-** | **-** |
|  | **Msh5*** | **-** | **P** | **P** | **P** | **P** | **P** | **P** | **P** | **P** | **P** | **P** | **P** | **P** | **P** | **P** | **P** | **P** | **P** | **P** | **P** | **P** | **P** | **-** |
|  | **Zip1*** | **-** | **-** | **-** | **-** | **-** | **-** | **-** | **-** | **-** | **-** | **-** | **-** | **-** | **-** | **P** | **P** | **P** | **P** | **P** | **P** | **P** | **P** | **-** |

|  | |  | | **Diaphoretickes** | | | | | | | | | | | | | | **Amorphea** | | | | | | |
| --- | --- | --- | --- | --- | --- | --- | --- | --- | --- | --- | --- | --- | --- | --- | --- | --- | --- | --- | --- | --- | --- | --- | --- | --- |
|  | | **Excavates** | | **Alveolates** | | **Stramenopiles** | | | | | | | | | **Archaeplastida** | | | **Opisthokonts** | | | | | | |
|  | |  | | Ciliates | Dino-flagelates | Diatoms | | | | | | Eustigmatophyceae | Brown algae | Oomycetes | Red algae | Plants | | Animals | | | | Fungi | | |
| **Name of the Species** | | *Giardia* | *Trichomonas vaginalis* | *Tetrahymena thermophila* | *Symbiodinium minutum*  [11] | ***Thalassiosira pseudonana*** | ***Phaeodactylum tricornutum*** | ***Fragilariopsis cylindrus*** | ***Pseudo-nitzschia multiseries*** | ***Pseudo-nitzschia multistriata*** | ***Seminavis robusta*** | *Nannochloropsis gaditana* [132] | *Ectocarpus siliculosus* | *Phytophthora sojae* | *Cyanidioschyzon merolae* | *Arabidopsis thaliana* [133, 134] | *Oryza sativa* [135] | *Homo sapiens* [136] | *Danio rerio* | *Drosophila melanogaster* [137] | *Caenorhabditis elegans* [138] | *Saccharomyces cerevisiae* [139, 140] | *Neurospora*  *crassa* | *Schizosaccharom-yces pombe* |
| **Recombinational repair** | Rad51 | - | P(2) | P[141] | P | P(5) | P(5) | P(6) | P(6) | P(6) | P(5) | P(4) | P(7) | P(3) | P | P(9) | P(7) | P(6) | P(6) | P | P | P | P | P |
|  | Rad52 | P | - | - | - | P | P | P | P | P | P | - | P | P | P | - | - | P | P | - | - | P | P | P |
|  | Rad1 | P | P | P | P | P | P | P | P | P | P | P | P | P | P | P | P | P | P | P | P | P | P | P |
|  | Msh2 | P | P | P | P(2) | P | P | P | P | P | P | P | P | P | P | P | P | P | P | P | P | P | P | P |
|  | Msh6 | P | P | P(4) | P(3) | P | P | P | P | P | P | P | P | P | P | P(2) | P(2) | P | P | P | P | P | P | P |
|  | Mlh1 | P | P(3) | P | P(2) | P | P | P | P | P | P | P | P | P | P | P | P | P | P | P | P | P | P | P |
|  | Mlh2 | P | P(2) | - | - | - | - | - | - | - | - | - | - | - | P | - | - | P | P | P | - | P | P | P |
|  | Mlh3 | - | P | - | P | - | - | - | - | - | - | - | P | - | P | P | P | P | P | P | - | P | P | P |
|  | Pms1 | P | P | P | P | P | P | P | P | P | P | P | P | P | P | P | P | P | P | P | P | P | P | P |
|  | Mms4 | - | - | P | - | - | - | - | - | - | - | - | - | - | - | - | - | P | P | P | P | P | P | P |
|  | Mus81 | - | - | P | P(2) | - | P | P | P | P | P | P | P | P | P | P | P | P | P | P | P | P | P | P |

Proteins highlighted in bold and marked with an asterisk are meiosis specific genes. For each species, the presence (P) or absence (-) is shown, while numbers in parentheses indicate number of homologs/orthologs and numbers in the square bracket correspond to the respective references
