# Supplementary material for: Identification of the meiotic toolkit in diatoms and exploration of meiosis-specific SPO11 and RAD51 homologs in the sexual species Pseudo-nitzschia multistriata and Seminavis robusta
Source: BMC Genomics. 2015 Nov 14;16:930. doi: 10.1186/s12864-015-1983-5 (PMC4647503; doi:10.1186/s12864-015-1983-5)
Supplement: Additional file 6: Figure S19. — Expression profiles of SPO11-2 and SPO11-3/TOP VIA homologs in Thalassiosira weissflogii during spermatogenesis compared to purely asexually-dividing cultures. (PDF 248 kb) [file 12864_2015_1983_MOESM6_ESM.pdf]

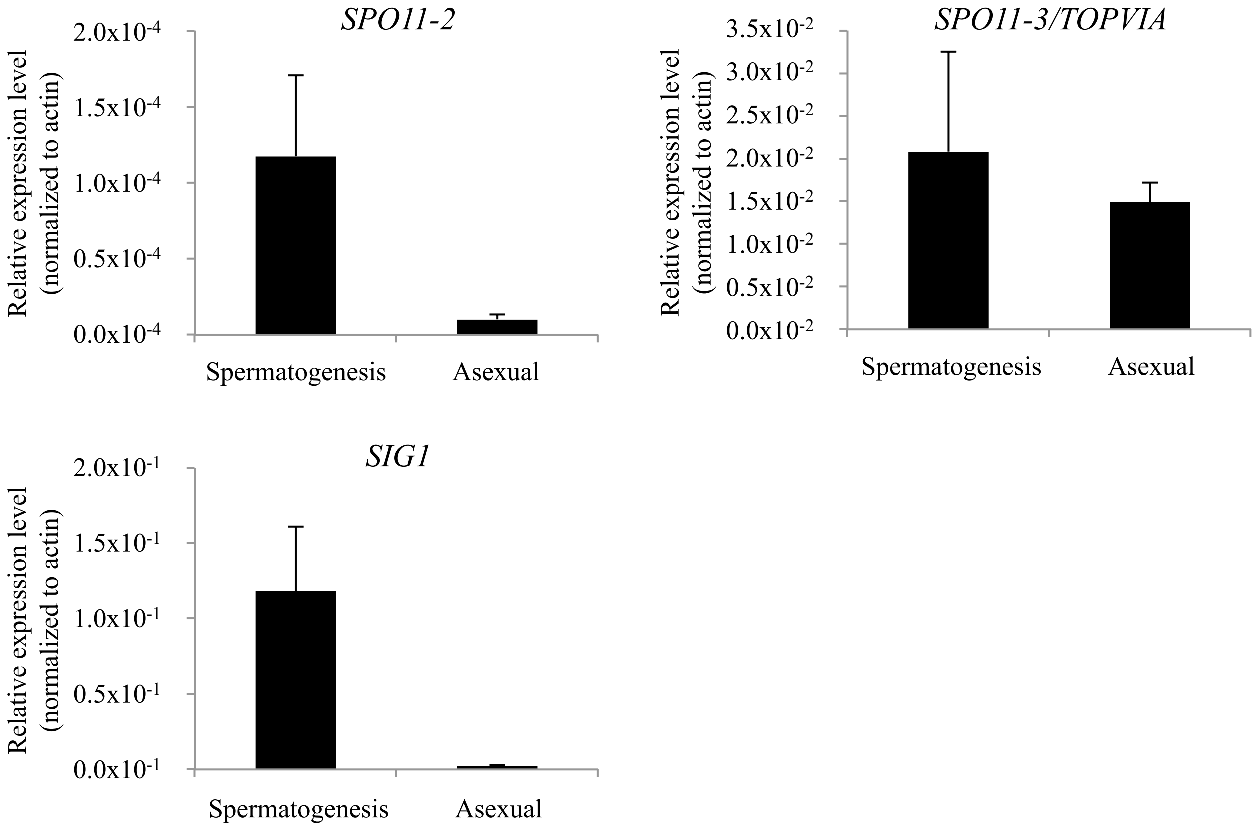

Figure S19. Expression of *SPO11-2* and *SPO11-3/TOPVIA* homologs in *Thalassiosira weissflogii* during spermatogenesis compared to purely asexually-dividing cultures. For comparison expression of the sexually-induced gene *SIG1* was analyzed in parallel. Small-size sub-clones (below size-threshold for induction of spermatogenesis) of *T. weissflogii* strain CCMP1587 were grown in f/4+Si medium, 20˚ C, continuous light (120 µmol photons m^{-2} s^{-1}). Exponential cultures were exposed to 10 h of darkness to trigger entry into spermatogenesis and harvested 14 h after return to light, the time when spermatogonangial cells enter meiosis. The non-induced control was a sub-clone with cell size above the threshold for induction of spermatogenesis, kept in exponential growth, to ensure absence of meiotic cells. RNA was extracted using the Trizol protocol, purified with a QIAGEN RNA columns with DNase treatment. Quantitative PCR was performed on an iCycler Real Time PCR Detection System (Bio-Rad) and expression was normalized to the expression of the actin gene. Absence of contaminating genomic DNA was verified by lack of amplification in RT- control reactions performed on the same RNA samples.
