# Supplementary material for: Identification of the meiotic toolkit in diatoms and exploration of meiosis-specific SPO11 and RAD51 homologs in the sexual species Pseudo-nitzschia multistriata and Seminavis robusta
Source: BMC Genomics. 2015 Nov 14;16:930. doi: 10.1186/s12864-015-1983-5 (PMC4647503; doi:10.1186/s12864-015-1983-5)
Supplement: Additional file 7: — Gene models for meiotic genes from the Pseudo-nitzschia multistriata genome. (DOCX 142 kb) [file 12864_2015_1983_MOESM7_ESM.docx]

Color codes:

1. Three prime UTR:
2. Exon:
3. Intron: No highlighting
4. Five prime UTR:

>PSNMU-V1.4_AUG-EV-PASAV3_0061270.1 class=Sequence position=[PsnmuV1.4_scaffold_29-size_260220:12817..16215 (- strand)](http://gbrowse255.tgac.ac.uk/cgi-bin/gb2/gbrowse/maplesod_psnmu_v1_4_gbrowse255?name=PsnmuV1.4_scaffold_29-size_260220:12817..16215) *MCM2*

CGAAAGGACGCAACGGCCACAATCCCAATCGGAGGAAGGAACCCAGAAAACAGCCAAGCATGAGCGATCTCGAAGACGAC

AGCGACGACCTCTCGTCGCCTCCCGCATCGGCCAAGAAGCGCCGCCGCATCCTTGGCGCACGGGACGACGAGGAGGACGG

GGAGGAGGGCGGAGGCCGTGGGGCTTCTAGGGACAACGACGAGGACAACGACGAGGACAACGACGAAAACCCCGCCGCCG

TCCGCCGCCGACGGATGGAAGCCCTGAACGCCGAACGCCAGCGCAGGCGACAGGATCGTCTGCGGAGGGAACTGGCGGAC

CTGCAGTCGGAGGAGCTCCTGCCCCTCGAGGAAGACGAAGATCGCCGCGCGGACGACGTGGACGACGAGGTCGACCGGGA

GTACGACTACGAGGACGGAAACCCCGACGCGGATGCCGATGCCGATGCGGACGCGGACGCGGGCGCCTACCCCGGCGACG

AGGACGAGGACGACGGGGAGGACCTGCTGGACAACGCCGAGCGGGACTACCAGCGCATCGCGGCCCTCGATACCTACGGC

CGGGAGGGGATCGACGACCGGGAGTACGAGGGCCTGAACGCGGACGCCCGCCGCGAGGTCGAGGCCAAGCTCCGGGAGCG

CGACCGGAGGCTGGGCCTGGGGGGGAGGCGCCGGGACGCCGGGATCTACGGGGCGGCCCTGGACGCCATGGAGGCGGAGG

AGGACGCGGACGCCCGCCGGGCCCGCCGGGGCGTCTTCCGGAGGGGCGAGGGGGGAGACAGCGATCGGGAAGGGGCCGAG

AGCGCCGGCGACGAGAGCGAGGACGACGAGATCGACGAGGCCGAGCTGGACGGGGAGGACCCCATCAACCTCGAGGCCTT

TGACGTGCCGCTGCGGGAATGGATCGCACAGGAGCGGACCCGGAGGGAGGTCCAGCGCAAGTTTCGGGCCTTTCTCCGGC

ACTTCCGGCCCGGGGCGACGGAGGAAGAGCAGCAGCAGCAGAAGCGCCGCAGGAAGGCGAACGGGATCTACGAGCAAAAG

ATCCGGTCCATGTGTGCCTCCAACAAGGCCACCCTGCAGGTTTCCTACCACCACCTCATGGAGGCGGAGCCCCTCCTGGC

CGTCTGGCTGGCCGATGCCCCCAAGGACATGCTGGACCTGCTGGACGAGGCGGCCACACGCCACACGCTCATGATCTTTC

CCTCGTACGGGGCGATCCAGGAGGACATCCACGTCCGGATCGCCGACATTCCCATCATGGACTCGCTCAGAGACCTCCGC

CGGACCCACCTCGACCACCTGGTCAAGGTCCACGGGGTGGTCACCCGCCGGGGAGAGGTCTACCCGAAGCTCCTCATCGC

CTACTACCGCTGCCTCAACAAGGACTGCCGGGCCGTCGAGGGCCCCATCCGGATCGAGGGGGTCGGCCAGGACGTGGAGT

CCCTCATGATGCCCGAGGAGTGCCCGCTCTGCGAGGGAAGCCGCTTCAAGCTAGACGCGAACCTCTCCGTCTACGGGAAC

CTCCAGCGGATCAACCTGCAGGAGACCCCCGGATCGGTCCCCCCGGGCCGGGTCCCCCGCCAGAAGGAGGTCTTTCTGAC

CCACGACCTGATCGACGTGGCACGGCCGGGGGAGGAAATCGAGGTCACGGGGGTCTACGAACAAAAGTACGACTCGGGCC

TCACCCTCAAGTCCGGCTTTCCGGTCTTTAGCACCTTTCTGACGGCCAACCACGTCCGCAAGCGGGAGGACGCCTCGGCC

GCCGCCAACCTGCAGGAGGCGGACGTCCGGGCCATCATAGAGCTCTCGAGGGACCCCAAGATCGGGGACCGCATCGTCAG

GAGCATCGCCCCGAGCATCTTTGGCCACGACAACTGCAAGATGGCCCTGGCCATGAGCCTCTTCGGGGGCGTCCCCAAGA

ACGTCAACGACAAGCACCGGATCCGGGGGGACTGCAACGTCCTCCTGCTGGGAGACCCCGGGACCGCCAAGAGCCAGCTG

CTCAAGTACGCCGAGGCTACGGCGCCGCGGGCCGTCTACAGCACCGGCAAGGGGGCCAGCGCCGTGGGACTGACGGCCGG

CGTCCACAAGGACCCGATCACGCGGGAGTGGACCCTGGAGGGCGGGGCCCTCGTGCTGGCGGACAAGGGGGTGGCCCTGA

TCGACGAGTTCGACAAGAGTGAGTGCGGTGGCAGAAGCCCGGAAGCGACCGAATCGTTTCGTTGCTTTTGTTTCGATCCG

TTGCGAGGAAAACCCGCCCTTTCTTGATTTCCATCCACACATTACTGTTTTCTCACGTCTCCCAACTTTCATCGATGTTT

TTTCACAGTGAACGAACAGGATCGAACATCCATCCACGAAGCCATGGAACAGCAGTCGATTTCTGTCTCCAAGGCCGGAA

TCGTAACCTCTCTGCAGGCCCGGTGCTCCGTGATTGCCGCTGCCAACCCCATCGGTGGCCGCTACGACAGCTCCAACACG

CTTTCCGACAACGTCGAACTGACCGCTCCCATTTTGCAGCGATTCGACATTCTGTGCGTTCTCCAGGACACCGTGGATCC

CGTCCTCGACGAACGACTGGCCAAGTTCGTGACCTCGAGCCACATGTCCGCGGTAGCGACACGGGATCTCAACAACGGGG

AGGCCACCCTGCCCCCGAGCACCAGTGTCGCCCAGCAACAGGAGGGCATCATCGACCAGGAGCTTCTGCGGAAGTACATT

CAGTACGCCCGGACCAACGTCAAGCCCACGCTCCGGGGGAATGCCTTTGACCAGGAAAAGATTGCCTCCCTCTATGTGGC

CCTCCGCAGGGAATCGAAGAATTCGGGGGGGGTTCCCATCGCGGTGCGGCACGTCGAGAGCATCATGCGCATGTCGGAGG

CCCACGCCAAGATGCACCTGCGCGACTACGTCCGAGACGACGATGTGGACGCCGCCATCAAGATGATGCTGGAATCCTTC

ATCGTGGCGCAAAAATTCTCGATCCGGCGGTCCCTCCGAAGGAGCTTTGCCAAGTACATCACCAGCGGGGAGGACCGGGC

CCATTTGCTGCTGCACATTCTGGGCGACATGATGCGCAAGGAGCAAATGTACCAGGTCATCCGGCTGCGCCAGAAGGGAC

AATCGGAAGACCTCCTGGGAAGGCTCGAGGTGCCCGTGGACGAACTGGAAGCCCGGGCCCGCGAGCGCCGCATCTACAAC

GTGACGGATTTCTGCAAGGGACCCAGCTTTCAAGAAGCCGGTTATTTTCTGGACGAGAAGAGGGGCCTGATCAGCCGGTC

GGCCGTCATTTCGACGTAGGAAAGAAACGATCCCATACCTGCCAATGGTATTGTTTTGGAAGAAAGCAGGTACAGCAAAG

CACTGCATTGAAGTGCCCTATACGCAGGAACCATTTCAA

>PSNMU-V1.4_AUG-EV-PASAV3_0004850.1 class=Sequence position=[PsnmuV1.4_scaffold_106-size_142667:51350..54495 (- strand)](http://gbrowse255.tgac.ac.uk/cgi-bin/gb2/gbrowse/maplesod_psnmu_v1_4_gbrowse255?name=PsnmuV1.4_scaffold_106-size_142667:51350..54495) *MCM3*

AGGAACAAATCGGACAGCAGCAGCAACAGCAACAACACGATACAATACAACAGAGAAGCAACAGGATAGCATTGGTTGGT

GGTTTCTTAGGTTAGGACTTCGATAGCGGTAGCAAGAAGCACGATGGCGGCAACCGACTTTTCCTCGTTGAGGGACGAAT

ACGATAACTTTTTGCGGCAAGAGGTGAGTATGCGCCGGGGTTCTTTCGAGGTCAGCAAGACAGCACAAAGATAGCGTCGA

CGAAGAACCTTACAACCTTTTCCTAACCCTTGTTCTCATGGCTCTAATTCTACCGGCGACACGAAACGATTCGAAACAAA

ACAAATAAAACAAAACACAACAACCACCAATCCGATGAAACGGATCCAACCCAACGAACCCGACCAATGCAATAACAAAC

ATAACAACAACAACAACAACCACAGCGCTTCCAGTACGTCGACAAAATCAGCACGGTCCTCGACGACTACGCCCACCGCA

GCAAGTCGCTGAACGTGGAGGGATGGGGCTGCCCCCGGGTGGAGATCCGCATGGCGGAGCTCCGGTCGATGGGAAACGGC

GGGCTCTCGCAGCGCCTGCGCACGGACCCCCTCCCCCACATCCGGGCCCTGGAATCGGCCTGCCACGAGGTGGCGGTGGA

GGCCCGGCCGGGATACGACAAGAACGGTACGAACAGATCAACGGGACGCGACGCGACGCGATGCTACGCGAGTGTAGTGT

AGTGTAGTGCGGTGCGATCTTGCACGATTTCTCACTTTCTGTTTCTTTGTTTGTTTCATCGAAAACGTTGATTTCACTAC

CCATTTCCATTCCCATTTCCATATCAATGCCAATGCCAATGTCAATGTCAATCTCGCTGCAAATATCAATGCCAACGACT

CTTTAGGAATCAAAATCAAGGTCGCCTTCTCGGGCCCCGTGGGTGCAAAGCCCATGAGCCCCCGGAGCCTCACCTCGTCC

TCCCTGAGACAACTCGTATGTGTTGAGGGAGTTGCCACCAAAGTGTCTGCGATCAAACCAAAGATTGTTCGTTCCGTACA

CTACTGCCCCGAAACAAAACAGCACCTGGAGAGAGAATACAGGGATGCCACCGATCCCGAGCTCGGCCTCCACGCCATCG

ATGCCCAGGGAAGAGAACTTCCGGACCGAATGATCGGAATCACCCCCACAGCCTTTCCCCAGAAAGACAAGGACGGGAAC

CCACTCGAGACCGAGTTTGGTCTGTCGGAATTCAAGGACCACCAAACGGTCGTCCTTCAGGAGATGCCGGAGCGGGCACC

GATGGGACAGCTGCCCCGATCGGTCGAGCTCATCCTGGACCACGATCTGGTGGACAAGATCAAGCCCGGGGACCGTGTGC

AGATCGTGGGCGTCTACCGAGCCCTGTCCAGATCGGGAAACAATCCGTCCGGAGGCGGGGGGGCCTTCAAGACGGTGGTC

TTGGTCAACAACGTGCAGATTCTCGGGCGGGACACCAGCCAGCTGACCTTTTCGCCACAGGACGTGCGCATGATCAAGGA

ACTGGGAAAGAATCCGGACATCTTGTCGATCCTAGGAAGGAGTCTGTCGCCCTCGATCCACGGCCACTCCGTCATCAAGA

AAGCCCTGGCGCTGCAGCTGCTCTCGGGATGCGAGAAGAACCTGAAAAACGGCACGCATCTGCGTGGAGACATCAACATT

CTCATGGTGGGCGATCCATCGACGGCAAAGAGCCAGCTGCTTCGATCCGCCATGACGATTGCCCCACTCGCCGTGTCGAC

CACCGGAAAGGGAAGTTCCGGTGTTGGTTTGACCGCCGCGGTCACATCCGACGCGGACACCAACGAGCGGAGGTTAGAAG

CCGGAGCAATGGTCCTCGCCGATCGTGGTCTCGTGTGCGTGGACGAATTCGACAAGATGGGGGAAAACGATCGGGTTGCC

ATCCACGAAGCTATGGAACAGCAGACGGTGACCATTGCCAAGGCCGGGATCCATGCCAGTCTCAACGCGAGGTGTTCCGT

CCTCGCCGCTGCCAACCCCGTCTACGGGCAGTACGATCGCTCCCGAAGGATCCAGGAGAACATCGGGCTCCCGGATTCAC

TGCTGTCTCGTTTCGATTTGCTCTTTGTGGTTCTCGACCAGATGGACCCGGAAACGGACCGCAAGATTGCCAACCACGTC

ATCCTGGGCCACCGGTACCGGTCGGACCGCGGAGGTGCCGGGCACGATAGCGACTACGACGACGAAGACGAGGACTCGGA

CGACGACATGGACGACGGGGACAAGGTCCATTCGATATGGCAGCGGAATCGACAGGGCTCCGACCCGAGCGAAGCCGCGG

ACCCGCACTCGAGCGATATTTTGCAGCACGACTTTTTGCGGAAATACCTCCACTTTGCTAAGACGCGGATGAAGCCCCAC

CTCACCGAGGGCGCCCGCGAGTTCATCGCGAACCGGTACGCCGAGATGCGGTGCCGCCAGGACGAGAGGACGCTGCCGGT

AACCGCCCGTACGCTCGAAACCGTCATCCGCCTCGCCACGGCCCACGCCAAGGCGCGGCTGAGCGGCGTGGTGGAGGCGG

CGCCGGACTGCGAGGCCGCGATGGACGTGCTGGGCTTTGCGCTGTACCACGAATCCGGGGGAGACAAGAACCAAAACGAC

GAGCCGAGCGAGGGGCTCACCGACGGCAACGAGCAGGCGGCCGCCGAGGTGTCGGACGACGGGGAAGCGTCGTCCGCTTC

CGGGGAACCCTCCGCGAAGCGAAGGCGCACGGCCGTGACCGACGACGACAACGACGACGACGACGGGGAGAACAAACTGA

AGGGCCAGATCCTCGCCGAGCTCGCGCAGAACGATGGATACCTGCCCATCGACGAGATCTTGCCGAGCCGAGACGACCGC

GACGAAATCCTCAGGCTCGTCGAAACGCTCGTGTCCGAGGGGAAGGTCATGACGGAACAGAACGACGAGGGGACGCTGGA

CGTCTTTCCCGTGTCCGATTAGGCATTGGCCTGCGTGCGTCCTCTGTCTCTTTTTCTATGCATTTGCTGGCTCTCGCGGG

CCCACGGTGGGCACCTGCGCACAAAATGCTCGAATTCAACATCCTAAAAATATGTACTCGTTAAACCAATCCATCGAACC

GGACCGAATCAAATCAAATCAAATAG

>PSNMU-V1.4_AUG-EV-PASAV3_0078600.1 class=Sequence position=[PsnmuV1.4_scaffold_387-size_48713:39316..42163 (+ strand)](http://gbrowse255.tgac.ac.uk/cgi-bin/gb2/gbrowse/maplesod_psnmu_v1_4_gbrowse255?name=PsnmuV1.4_scaffold_387-size_48713:39316..42163)*MCM4*

TAAGATTTTTTTTTTAGATGATAGCTTTATGATACGGAAATTGGTAAAAATCGAGTTTGCATGCATTACAGTGTAAATCC

TTGAAAAACAACTGAATCGAGCCGAGCGCATCCAGGTACTTCATGTTTGCTGGTATGTTTCTTTCGGGGCCAAGCTCCTA

CGGAACCTCGACCATTTCGATCCTCCCCGTCTTCGGATCCGTGGAAGAGGCAAGAGTGGCCACACTCAATAGGCGGGCAG

CCTCCCTGGCGTCGGCCCCCGAGATGAGGGGCGCATGTCTCATCCTGGCCAAGGCCTCGCTGGTGCGAATGAGAGACTCG

AGCTGACGGGGCGTCGCCGCCAGGGTTCCGTTGTTTCCGGTTCCGATCTTTCGAAGCTTCAGATAGCGCTCAAGGAGCTC

TTCCTCCGCGGCGTCGCCCAGGCGCGGGTGGACGGTGGCCCGTGCGTAATCGATGTAGTCCCTCAACAGGGCGGTGTCCA

AGGGGGGCTCACCAGCGGTGGGGGTCTCCTGGTAGAGGCCGACCAAGTGCCTCGCGAGCCTGCGGTCCGCGTCGGGGGAT

GCATTGTCGAGGACCAGGTAGACGAGATCAAACCGGCTGAGGAGGGTAGGGGACAGGCGGAGATTTTCCACCACACTCCT

GCGGGGGTTATATCGCGACTCCGAGGGGTTCGCCGAGGCCAGGATCGAGGCCCGGGCGCGCAGGCTAGATACTATCCCGG

CCTTCGCAACCGAGACGGTTTGTTGCTCCATGGCCTCGTGCAGGACCGACCGCGTCGCATCCGACATCTTATCGAACTCG

TCGATGCAGCAGATTCCCTTGTCAGAGAGGACCAGTGCCCCGGCCTCTAGAACGGGATCGCGGGTCTCAGGGTCGCGTAC

CACCGAGGCCGTGAGTCCGACCGCGGAGCTGCCCTTTCCCGAGGTGTATATGCCGCGGGGGGTGAGCTTGTGGACGTAGG

CAAGGAGCTGTGACTTGGAGGTCCCGGGATCACCGCAGAGAAGCACGTTGATATCTCCGCGCTTACGGGGCCTGGTGTCG

TCATGGTCGTGGTTCTCGCCGTCGTTCACCCGCCGCACCTGAGTTCCGCCGAACAGCATGCACAGTACGCCCTTCTTAAC

GTCGTCTAGGCCCCAGATGGAGGGCGCTAGAGATCGGGTGAGGATGTCGTAGATGTCAGGCCTCCTGGAGAGGGCTACAA

GTTCCTCCTTCCGCTCCGATGTAAGAAGCTTGCACCTTCCAGAAGCGGTGCCCACCCTCCGTGGTAGTGGTTGTCTTCGT

TGGGGGTCTTGTATTTTCAGTTCTCGTCGCTGTGATTGCAGTCGCGGTGGCTCCGACGAATCCCCCGCCATCCCTGTGAT

CCTTCGAAAGTGCAGGACATCAAGATAGGTTTTGTAGACAGACCTGACCCGGGTGGCCCTCGGGTTGACCCGCACCGACT

GGGCACGGAGGATCCCCGTCGCCTCGATCCGGTCTCCCGGAACGGCAGCGTCGACCAGGTCGTCGAAGCAATAGACCGCT

AAAGAGGCCGGGGTCCGGCCAGCCGGCACCGCTTCGGGCGTCTCTTGCAAGCGAACCAGCTGCTTGTTGGCAAAGGAGCA

GCGGTTGTGCTCTAGCCGGTGTGAGTCCCTCGTTCCGCAAGAGGGGCACACCGCGGGCTCGGAGATCCTCCCCCGGTCGA

CCGCTACGTTGTGAGCGTGCCCACAGACGCAGCAGGAAAAGTGGGCAATCCGGAGGTCCGGGACAACCGGGCTGCAGCGC

ACAACCATGCCCTGGATTGCGACAAGGGTGTCGACTGTGATGGGATCGAGGCACCGGAGGCCCTGGACCTCCCTAAGGTT

AAAGGGCCGGATCTGGACGACGGGGGGTTCGTGTGGGAGGTGTGATTCGTGTTGCTCTTGGTGGCCCGCCCTGAGTTCCT

CCATCTCGCGCCTCACAACGAGGTCCAAGAGCGGGACGATTTCGGAGGGGTAGTGGAGTAGCTGGTGATAGAGCTTCTGG

CAGGCCGCGTCGAAAAAGCACAGGTGCATCACGTCTACGTCGAGGCTGGCCGGACCGGTCCGTTGAACCATGGATTCCAG

CTCGGAGCTGTAGACCGGGGGTGGAAGCGAAGAGTTGTCATCGATTTCGTTGTCGCTGTCGTCATGGCCGCGCAGACCGC

GAAAACCCCGTAGGAATCCCCGAAAGGCCTGGGCGGCCTCAGCGACGTCTATGTCGGTCCCCCGCGGCGTGGCAAAACCA

TCTTGGGCGTCTAGAAAGTCACCTTCGTGGTTTTCGTCATTAATATCGTTTTCTGTCTCTTCGTTTACGTCGGCGTCACC

GTCACTCATGACCCCTCCGAATCGCGGCCGCCGGCTCCCCTGGAAAACTGATCCTCCACCGACTTCCAGAGGCCTGGAGG

GAGTAGGAGGGGCAAGGGGCGATAGAGCGGAACCTTCGTCCGTCGGCGTGGTTGGGATACCGTCTTCGGTGCCTCGTTCG

TCGACGTCCATCTGGGCCGCGGCGAGGGTGGTAGCGTTGTGTGCTTTGCTACGCGTTGTGAAAGGAAGCGAAGAGAGAGT

TTACAGTGGCTCGGAAGACCCCGCTAGGCTTCGGCTTCCCGGAGTTTCCGTCGAAGCGGACGGTCCGGGAGCTGGCCTTT

TTTTTAGGAAGATGGCTTTAAAACGACTCTCGGGCTATGTGCGGTGTCTGGATTGGGGGGAAGGCTACAGCTCGAACCTC

GAAACGAAAGGGAGGGAACGGAAGTCCTACTCCGACCTCTCGTCTGGTTGGGGTTTGGATTCGGTTCCGTTGCTGATGAT

TTACTACTACGTGACTTTTCTTCTTACTAATACAAGCTGGTTTGTACC

>PsnmuV1.4_aug-pasa-abinitio_v2_0126930.1 class=Sequence position=[PsnmuV1.4_scaffold_387-size_48713:39311..41895 (- strand)](http://gbrowse255.tgac.ac.uk/cgi-bin/gb2/gbrowse/maplesod_psnmu_v1_4_gbrowse255?name=PsnmuV1.4_scaffold_387-size_48713:39311..41895)MCM4 Corrected

GGTCTTCCGAGCCACTGTAAACTCTCTCTTCGCTTCCTTTCACAACGCGTAGCAAAGCACACAACGCTACCACCCTCGCC

GCGGCCCAGATGGACGTCGACGAACGAGGCACCGAAGACGGTATCCCAACCACGCCGACGGACGAAGGTTCCGCTCTATC

GCCCCTTGCCCCTCCTACTCCCTCCAGGCCTCTGGAAGTCGGTGGAGGATCAGTTTTCCAGGGGAGCCGGCGGCCGCGAT

TCGGAGGGGTCATGAGTGACGGTGACGCCGACGTAAACGAAGAGACAGAAAACGATATTAATGACGAAAACCACGAAGGT

GACTTTCTAGACGCCCAAGATGGTTTTGCCACGCCGCGGGGGACCGACATAGACGTCGCTGAGGCCGCCCAGGCCTTTCG

GGGATTCCTACGGGGTTTTCGCGGTCTGCGCGGCCATGACGACAGCGACAACGAAATCGATGACAACTCTTCGCTTCCAC

CCCCGGTCTACAGCTCCGAGCTGGAATCCATGGTTCAACGGACCGGTCCGGCCAGCCTCGACGTAGACGTGATGCACCTG

TGCTTTTTCGACGCGGCCTGCCAGAAGCTCTATCACCAGCTACTCCACTACCCCTCCGAAATCGTCCCGCTCTTGGACCT

CGTTGTGAGGCGCGAGATGGAGGAACTCAGGGCGGGCCACCAAGAGCAACACGAATCACACCTCCCACACGAACCCCCCG

TCGTCCAGATCCGGCCCTTTAACCTTAGGGAGGTCCAGGGCCTCCGGTGCCTCGATCCCATCACAGTCGACACCCTTGTC

GCAATCCAGGGCATGGTTGTGCGCTGCAGCCCGGTTGTCCCGGACCTCCGGATTGCCCACTTTTCCTGCTGCGTCTGTGG

GCACGCTCACAACGTAGCGGTCGACCGGGGGAGGATCTCCGAGCCCGCGGTGTGCCCCTCTTGCGGAACGAGGGACTCAC

ACCGGCTAGAGCACAACCGCTGCTCCTTTGCCAACAAGCAGCTGGTTCGCTTGCAAGAGACGCCCGAAGCGGTGCCGGCT

GGCCGGACCCCGGCCTCTTTAGCGGTCTATTGCTTCGACGACCTGGTCGACGCTGCCGTTCCGGGAGACCGGATCGAGGC

GACGGGGATCCTCCGTGCCCAGTCGGTGCGGGTCAACCCGAGGGCCACCCGGGTCAGGTCTGTCTACAAAACCTATCTTG

ATGTCCTGCACTTTCGAAGGATCACAGGGATGGCGGGGGATTCGTCGGAGCCACCGCGACTGCAATCACAGCGACGAGAA

CTGAAAATACAAGACCCCCAACGAAGACAACCACTACCACGGAGGGTGGGCACCGCTTCTGGAAGGTGCAAGCTTCTTAC

ATCGGAGCGGAAGGAGGAACTTGTAGCCCTCTCCAGGAGGCCTGACATCTACGACATCCTCACCCGATCTCTAGCGCCCT

CCATCTGGGGCCTAGACGACGTTAAGAAGGGCGTACTGTGCATGCTGTTCGGCGGAACTCAGGTGCGGCGGGTGAACGAC

GGCGAGAACCACGACCATGACGACACCAGGCCCCGTAAGCGCGGAGATATCAACGTGCTTCTCTGCGGTGATCCCGGGAC

CTCCAAGTCACAGCTCCTTGCCTACGTCCACAAGCTCACCCCCCGCGGCATATACACCTCGGGAAAGGGCAGCTCCGCGG

TCGGACTCACGGCCTCGGTGGTACGCGACCCTGAGACCCGCGATCCCGTTCTAGAGGCCGGGGCACTGGTCCTCTCTGAC

AAGGGAATCTGCTGCATCGACGAGTTCGATAAGATGTCGGATGCGACGCGGTCGGTCCTGCACGAGGCCATGGAGCAACA

AACCGTCTCGGTTGCGAAGGCCGGGATAGTATCTAGCCTGCGCGCCCGGGCCTCGATCCTGGCCTCGGCGAACCCCTCGG

AGTCGCGATATAACCCCCGCAGGAGTGTGGTGGAAAATCTCCGCCTGTCCCCTACCCTCCTCAGCCGGTTTGATCTCGTC

TACCTGGTCCTCGACAATGCATCCCCCGACGCGGACCGCAGGCTCGCGAGGCACTTGGTCGGCCTCTACCAGGAGACCCC

CACCGCTGGTGAGCCCCCCTTGGACACCGCCCTGTTGAGGGACTACATCGATTACGCACGGGCCACCGTCCACCCGCGCC

TGGGCGACGCCGCGGAGGAAGAGCTCCTTGAGCGCTATCTGAAGCTTCGAAAGATCGGAACCGGAAACAACGGAACCCTG

GCGGCGACGCCCCGTCAGCTCGAGTCTCTCATTCGCACCAGCGAGGCCTTGGCCAGGATGAGACATGCGCCCCTCATCTC

GGGGGCCGACGCCAGGGAGGCTGCCCGCCTATTGAGTGTGGCCACTCTTGCCTCTTCCACGGATCCGAAGACGGGGAGGA

TCGAAATGGTCGAGGTTCCGTAGGAGCTTGGCCCCGAAAGAAACATACCAGCAAACATGAAGTACCTGGATGCGCTCGGC

TCGATTCAGTTGTTTTTCAAGGATTTACACTGTAATGCATGCAAACTCGATTTTTACCAATTTCCGTATCATAAAGCTAT

CATCTAAAAAAAAAATCTTAATATT

>PSNMU-V1.4_AUG-EV-PASAV3_0118810.1 class=Sequence position=[PsnmuV1.4_scaffold_85-size_162413:15589..18505 (- strand)](http://gbrowse255.tgac.ac.uk/cgi-bin/gb2/gbrowse/maplesod_psnmu_v1_4_gbrowse255?name=PsnmuV1.4_scaffold_85-size_162413:15589..18505) MCM5

GTTCGAAGGCACTTCAAGGAATTCCTGAGTAAGTGATAGAGAGACGATTTGAATCATAGTCGAGGCGACGTCGACAGTTG

ACTGATTCCAGACATGCATGCGATGGATTGGTTCAAAAACAAAACCAGAGTTCTCACAGACACTTTCAATCCCTCGTTCG

CTCGTCTCTAGGACACTATCGTCTGGGCAATACGAGATATGTGTACCGGGAAAAGCTTTTGAGGATGCATCGGCGTTTGT

CGAACCACGGGAACAAGTATCCGACCGCCGAATCGGAGAACAATTACGAGGAAAACGAGGACGGAACTACAAACGCAACA

AGCAGCAGCAATAGTAGCCGGAAGAACCAGATGATGAACCACAAGAACATGATGCTAGAAATCGACGTTGCTCATCTCGG

GGAATACGATGGATCGCTCCTGGGGTATTTGCGGGAATCTCCGTCTGCGATACTGCCGGAGCTGGAGGTTGCAGCTGGGA

AAGCCCTCGATTCATTGCTTTACGATTTAAGGGAGTCTTCCTCCACATCGCATGGAAATACCCAAAACGAGGACGACGAT

CTAGATAACGACGAAGGGAACAGGAGCGAGGCAAACGAAAACGGAAACAACAACAGGACTAACAACAACGATGGCAGAGC

CTCTTCCGAGTGGTCGATCCAGATCTTGCTCAAGGGAAACCTCACTTCCACGCCGCTGCGCCAGATCCAGAGCCAGCACA

TCAACCGCCTCATCAAATGCCCGGGTATTGTCATCAGCGCAACACCCATCCAGGTTCGCGCCGTGAAACTCACGGTTCGG

TGCAACAGGTGCATGGACACCCATTCGATCAGCGCCACGGAGGGCCCCTACGGAAACGTTCCCCTCCCGCAGCGTTGCCA

GGGCGTAGAGCCGAAGGAATGTGGGCCCTACCCATACTCGATTGTAGCGGACGAGTCCCTGTTCAGCGACCGGCAAAAAC

TCAAGCTCCAAGAGTCGCCGGAAAGAGTTCCGACGGGAGAAATGCCACGGAGCGTCATGGTCGCCGTCGAAAAGGGACTG

GTGGACAAAGCCCCACCGGGGACCCGTGTAAGCCTCTTTTGTATTCCGACGCTCTTCAACGGTGGTGGTGGAGCCCCTGG

ACAGAGGGGAGGAGCGGGTGCCGTCCAGAAAGTATACCTGAGGTGCGTTGGAATGTCCAAGGATCAGGCCTCTGGCGAGG

CCGCAAGATTCACACCCGCCGAGGAGGAAGCCTTYCAGCAATTCTCCAAGCGGGCGGATGCCTACGAGATTTTGACCCGC

TCGGTCGCTCCAAGCATCCAGGGCTCGTACACGGTCGACATCAAGAAAGCTCTGGTGTGCATGCTAATGGGTGGAACCCG

CAAGCGGCTGCCGGACGGCATGCGGCTCCGTGGGGACATAAACGTTTTGCTGCTGGGGGATCCATCGACTGCCAAGTCGC

AGTTTCTCAAGTTTGTAGGCAAGACCTCCCCGATCGGAATCTACACCTCCGGTAAGGGTTCGTCTGCAGCGGGTCTCACG

GCGAGCGTGGTGAAGAACGCGGCCGGTGAGTTCTACCTGGAAGGCGGGGCGATGGTTCTGGCAGATGGCGGAATCGTCTG

CATCGACGAATTCGACAAGATGCGACCCCAGGATCGGGTAGCAATCCATGAGGCCATGGAGCAGCAGACGATCAGTATCG

CAAAGGCCGGAATCACCACCGTTCTGAACAGCAGGTCCGCGGTCCTGGCCGCKGCAAACCCCGTCTTTGGACGCTATGAC

GACTTCAAGTCGGCGTCGGAAAACATCGACCTGATGACCACAATCCTGTCCCGGTTCGACATGATCTTTTTGGTGCGCGA

CGTGCGAGAGGAGGAGCGTGACCTCATGATYTGCCGACACGTGATGGGGGTCCACATCCGGGGAGGTGCCCCCGACAGCA

ACGCTCCCAGTGGGGGGCCCTCCGGGACGGGAGGCCTGGGTTTCCTGGCCGGGCTATCGATGGGAGGGGCAGGCGGTGAA

GGCGGAGGAGACCGGGCCAGGTGAGTTTGGTGGCTAGGTGCCGRAACATCTTTTCAAGCAGAGGAGAACTACTGCGAAGC

GACAATTRTGCGCATTTCGTTGTTTTGCAAATCGATTGYATGTTACCTAACGCTGTTTCYGCTTCCTCGTTTCTTCGGGG

TTTTGCATCACTCAGATCCGAAGCCCAGAGCAACACCCCYGAGGCCATTGCAGAAAACGTAATGCGGCTGGCCACTACGG

GGCAGGGCGAGCTAGACGTTCCTTCCATGAAGAAATACATCCAATACTGCAAATCGAGGTGCAAGCCAACCCTGTCGGAA

GAAGCCGGCCAAGTGCTGACCAGCTCGTACGTGAAAATTCGGGACGACGTGCGCAAATCGACCCTTCAGTCGCTCGGTGA

GGACGCACAAGCCACCATCCCCATCACGGTGCGCCAGCTCGAGGCCCTGGTCCGCATCTCCGAGAGCCTGGCAAAAATCC

GGCTCGATTCGGAGGTTCGGATCGAGGACGTGAACGAGGCCCTGCGGCTGTTCCGGGTGTCGACCATGGCCGCTAACTCG

GCTGACCAGGCCAACGGCGCCGGTGGAACGGGTAGCGGAAATCCCTTCCTAAACGGAGCGGGCGGGAACAACCGGGAGGA

ATTTGACCGTGCGCAGGGCTTTTTGCGGTGCCGGCTGGCCATCGGAAGCACGGTCAACAAGCAAAAACTGATCGAGGAAG

GATCGGGGCAGGGATACGACTTCATGACCATGGTGCGTGTGCTTTCTGCTATGGCGCAGCGGGGTGAACTGCTGGAGMGA

AACCAAGGCCGGCTCCTGAAGAGAATAAAATAGTCACTATTAATCAGGGCACTGCCAAGTCTATTGGAATCAGGGCACTG

CCAAGTCTATTGGGAGCAATAAAAGAWCAGTTTTAGA

>PSNMU-V1.4_AUG-EV-PASAV3_0022580.1 class=Sequence position=[PsnmuV1.4_scaffold_15-size_312907:251606..255857 (+ strand)](http://gbrowse255.tgac.ac.uk/cgi-bin/gb2/gbrowse/maplesod_psnmu_v1_4_gbrowse255?name=PsnmuV1.4_scaffold_15-size_312907:251606..255857) MCM6

CAAAACACRGCCCACCACACCAGTTGATCCGTTGATCGAAAAATCACCACGAGCGCAAGTTCGGYAGCACCACGACCCAC

CGTTCGCCGCCACGATGGACGAACACGACGACGACATGCTGTACGACGACGACTACCGGCCGCCCACGAGCGGGGGGGAT

GGTCCGGGCCTTAGGGGGGAAGGTCCCGGTGCCGTGGTGGCCACCCCGGAAAGCCGGGCCACGGGCGCGGCTCGCTACGA

CGGCAAAGACGACGACAACGACAACGACGACGATCCCCTGGCGCAGGCCGAGGCCGCGGCCGCCGCCGCGGAGGTGGCCG

CCATGGAGGGAGGAACCGGCCCCTCCGCAAGCGSCCGGGGGCGGGACGCGACGGCCGACCGGGCCCTGATGGGGGCCCAC

CAGGACGCCGACGGCGGGGACAACGCCGTGGCGGCCTACGAGGTCCTGGACGAGAGCGGGGAGCTGGTCCGGGGGGCCTT

TGAGGAGTTTCTGCAAAACTAGTGAGTCCGGCATGGGATGGCGTGGCACGACATGGCAGAACATGGCATTGCATGGCACC

GCGGGAGTAGAGTGGGCGTAGCAAGGCTGTGTTTGTGTTTCTCTTTGTCGTTGATGTTGTTTGGTGTTCTTTTTGCTGCG

TTGTGTGCTGCGCTTTGTGCTGCCGCTATGATGCGCCCAGGAAAGACATGATAGGAAACACACGGCAACTCAAGAGRGAT

TATTTGGTCATTACATGAAACAAAAAGCAAATCGATCGATCGATACTTACTAGATTCGGTTCATTCGAAATGGTCTCAAA

CCGCACGAGACTCAAATTTTCGAATGCATTTTCATTGTCTCTCACACACGCCTTGAATACCAAATTCACTCTAATTTTGT

CGAAATCAACTCAACTTCGTTCTACGAAATCCAATCCAATGCAACGCAATCCAATACAACTCTGGAACTAACAGCAAACA

AGCAGTCAACACGGACGATGGTGAACAGCAGCAGCAGAGCCACGATGACAGCGACAGCGAACACAGCACCGAATACATCT

ATCCCTACCAGGAGCAAGCCGATTTGCTGGCCAAGCGACAACTGAAACGCCGGCAATACAACGCCAACAATGCCTTTCAS

GAGGACAGCGACGACGAGGACGAGGAGGACAACCGCCACAATTCGTCCCACGGATTCGCCAAYACGCTCTTTGTCAATTT

CGCGCACTTGCTCAGGGACGATCCGGAACTCGCCGAGGCCATCCAGGGGGAGTTCTGCCGGTTCGAAATGCACCTTCGCA

GGGCCGTTTTGGGCTTTGTTGTGGACCGGCACCCGTCCCTGAACCGGTACATCGAGCGGGGGAACGGCGGGGGGGGAAGG

GGCAACAACACCCCGACGGATCCCCTCGTCTTTTTCGTCGCCTTTTACAACTGCCTCCCGAGCCAAATGAAATCGGTCCG

GGGCCTCCGGATGGAACTGGTGGGGTCACTGGTGAGCCTACAGGGAACCATCACCCGGACCTCGGACGTCCGGCCGGAGC

TGCTGGTGGCCTCCTTTCGGTGCGATAAGTGCGGTTTGCTGGCCGAAAAGATCCCCCAGAACTACCACTTTACCCGCCCT

ACGCTGTGCCGGAACCCCCGCTGCAAGAACAAGTCCCCCATGCGTTTTACGCTCGAGGCCACCCGATCGGAGTTTGTCGA

CTGGCAGAAGCTCCGGGTGCAGGAGTCCGCCCACCAGATCCCTTCGGGATCGATGCCCCGGTCGACGGACGTCATCCTTC

GGAACGAAATGGTCGAGCGCTGCAAGGCCGGGGACAAGTGCGTCTTTACCGGGACGCTGGTGGTGCTACCGGACGGGAGC

GCCCTGGCCCGGKCCGGGGACTCGGTGACCTCCACCAAGGCACCGGGCTACAAGACGAGCGACGCGGCCTCGGGGGGCGG

CGGCGGGGTCAAGGGCCTGTCCGCKCTGGGGGTCAGGGAGCTGACCTACCGGACCTGCTTCGTGGCCACTTCCGTCATGA

GCCTCGAATCGACCAAGCAGGCCAACACCAACGAGCAGGTCATGGCGGGGGCCCTGTACGGAACCACCAGTGACCGGTCC

GCCTCCCTGGACGACGACCCGACCACCAAGCAGGTAGCGATGGAGATGACGACCGCCCAGCGGGCCGAGCTCCGGGCCAT

GCGGAACTCCCCGCGCCTGTATGAGCAGATGGTCGATTCGTTGGTCCCCGCCACCTTTGGGCACCGGGAGGTCAAGAAGG

GGATTCTCCTCATGCTGCTGGGGGGTGTCCACAAGACGACGGGCGACGGGATCAAGCTGCGGGGGGACATCAACTGCTGC

ATCATCGGGGACCCCTCCACCGCCAAGTCGCAGTTCCTCAAATACGTCCATTCCTTTCTGCCGAACCGGACCGTCTACAC

CTCCGGAAAGGCCTCGTCSGCGGCCGGTCTCACGGCGGCSGTCCAGCGGGATTCCGACACGGGTGAATTCTGCRTCGAGG

CCGGGGCCCTCATGCTGGCCGACAACGGGATCTGCTGCATCGACGAGTTTGACAAGATGGACGAGCGCGACCAGGTGGCC

ATCCACGAGGCGATGGAACAGCAGACGATCTCCATCACCAAGGCCGGGATCCAGGCCACCCTCAACGCCCGGGCCTCGAT

CCTGGCGGCCGCCAATCCCATCTATGGCCGTTACGACCGCACCAAGACCCTCAAGGCAAACGTGACGCTGTCGGCCCCCA

TCCTRAGCCGATTCGATTTGTTTTTCGTAGTGCTCGACGACTGCAACCCGGAAACCGACCGTCAGGTCGCCAAGCACATT

CTGAAGGTYCACCGCGGCGAGGACCACGACGATCCCGCCAAAAAGGCCCCCTTTACGATGGACCAGATRCGCCGGTACAT

CAAGTTTGCCCGGACCCTGCACCCAAAAATCACGCCGGAGTCCCAAAGGGTCATGGTGGAGTGCTACCGCAAGCTTCGAC

AGGGGGACTCGCTCGGSCGGTCCCGGTCGGCCTACCGGATCACCGTCCGCCAGCTAGAGTCGATGGTCCGGCTGTCCGAG

GCCCTTGCCAGGCTRCACTGCTGTGACGAGATCACCCCGGCCTTTGTCCGGGAGGCCTACCGGCTGCTCAAGACTTCGAT

CATCCAGGTGGAAACCAGAGACATAGAAGTCGACGACGACGACGATGATTATGGCGATTACGGCGGTGGCGATGGGGTAC

TGGAGCAAGGGGACGACGGCGACGASGACAGCGACAACGGCGGCGGACCGGACTCGCAGATGCCGGACAGCCAGGGGCTG

GAAACACAGCAACCGCAACACGTCTACCGACCGGGAGAATACGTTGGAGAGGAGCGACAGCCAGCGGAGCCACCAGCCGC

CAGCGATGAAGGAGCGGGAGACACGGCCCCTGGGATGGATGCGACGGGGGTGAGCCCKCCAGAACCAAAGGCCGAAACGG

ACGACTCGGGAGCAAAAGCGGGCAAGAAAAAGAAGAAGAAAAAGAAGAAAACCAAGATTTCCTTCGAGGAATACGAGGCC

ATTACGAACGCCATTGCCTGCCACCTCCGGGCCCTGGAGGAGGAGCAGATCGAGAAGTCGGTTGGGGGCGCGATAACCGA

CCAGACCTACTGGACCTGGAAGCAGGCCTCGRACTGGTATCTSGAGCAGGTCGAGCGCGACATTGGGGACTCTGTGGAGC

AGCTCGATGCGATGCGGAAAAAGGTAAACCTGGTGATCAAGCGTCTCATCGAGGTCGACCGGATCCTAGTAACCGTGGGG

GACGTTCCCAGGAGCAAAAAGGAAGAAGGGAAAACACTGCTGGCGGTCCACCCAAACTACGAAATCAGCTAGTGGAAGTC

TTTCGGAAGTTAAAAGAAACTGGTACTTGTTTTGTCCCTCAAGTCCTTACACGATGCGTGTGGGTTTCTTCGACTTCGCT

GTTGGTCCGCAACTCATTGCTCTTTGCTTGTTTATCAACACAATAAGATTCCGAAGTCAACTWTTGGCGCTCTAAKTATT

CGTATTAGTGTCTACTAGAAAAAAATTCTCAAAAWCAGGGGAAGACGACACGGGAAAATGGCGCTAGACCAGCAGATTGC

CAAGTCAGCATATTCCACATTCGTACTAGTCTTCGCAAGCAAAATGGCATCCAAAACCGATGAAAACTCGTTTTTGTATC

GACACTCAAGGAACAAAACGTTGTGTCTGCCGTAGGTATGAATATTGTTTGAATACTTTTTTTTCAATGAAGAATAAAGA

CTAGAAGAGTAA

>PSNMU-V1.4_AUG-EV-PASAV3_0109820.1 class=Sequence position=[PsnmuV1.4_scaffold_70-size_199570:77787..81305 (- strand)](http://gbrowse255.tgac.ac.uk/cgi-bin/gb2/gbrowse/maplesod_psnmu_v1_4_gbrowse255?name=PsnmuV1.4_scaffold_70-size_199570:77787..81305) *MCM7*

AGCGTACTGTACAGTACGATAGAACTTGTACCGTACCACTCTCTGCCATGCCCGCACTGGCGTTCACGACAAGAACAGGA

TACGATACGGCGTGATAGATTACGATACGTACGAACACCAAACAAACACCGATAGGGAACGAATCTCTTGTGTTGCGATA

CGATACGAAACAGCAAGGGATTTTGGGTTTGCGGAACGAAACGAAACGAAACGAAACGAAACGAAACGAAACGAAGCGAA

ACGAAGCGATCAGCTAGCATCCAGAACACCGGCGTCAAAAGAAGGGCGAGCGCAGCGGCACGATGGCGTCGCAAGATACG

ACCGCAACGGCCGCGGAAATAGTGACGGGCAACCAGTATCCCGATTACGACGGCGATTCCGGTGAGTGTTTGTGAACGCA

AGATCCATTKGGAGACTTCGGTGTCCGATCCTGTTTCGGAGGAGGTTTCGAATCGCCTGTTTTTTGGTCTCTGCTTTGGC

CGACGGTCTCGCATCGTGCACAACCCAACCCCGTACCACTCACCATCGCAACGCAATGCACCGCCCTTGTTTGATTTCTT

GGGTGGGTCCGTCCGCCGCGCAACCCAACGCAACCCAACGCAACCCAACGCAACCCAACCCAACGCAAAACAACGCAGCC

ACCTTTGCGAGTTTCGTGCTGGAGCACACCATCCGGCGGGTCGTCCGGGGCAGCGGGGACGGCTCGGATTCCGAGGGCGA

GGACGGCGACCAGCGGCTCTTCGAGCCCACGGGCGAGACGGGAGCCTCCGGCGAGGCGATCCGCGTCGAGCGGGTCCCGT

GCTACCCCCGCCTGCTGCAGAAAATCGCCGACAGGGACTACCCGACCGACACGGCGACGACCTTTCTGGTCGAGGTCCCC

CTCTCGGAGCTCGTCGCCTGGGACTCCGTCCGGGGGGGGGACCTCTGCGAGCGGGCGGCGGCGAACGGGCAGCGGTACCA

CTCGCTCTTCTGCACGGTCCTGGAYGGCATCCTCAGGGACAGCTTTCGGCCGTCGGGGGGGGTGACCCGCAACCCACGCG

GCCGCGGCTCGGTCCTGCTCAGGGACGCCATGGACGTGCTGCAGGAGCAGCGCATGGCCCTCCACCGGCAAGCCCAGCAG

CAGCAGGACCAACAGCAGGAGGCCGGCGTGGAGGGGGACGCCGCCAATGCGGGCGGCGGCGACGAGCGAAACGCCTTTCC

CCCCCTGCTCATGCGGCGGTACGAGCTCCGCATACTGCCYCTCGGCCGCCGGGGGAGCCTCTTTCCCTTTGAGGACCAGT

ACCACCCCTCCAAGGCCGCGGCGGCGATCCCGAGGGGGGTCTCCCTCCGGCACGTCCGGTCCAAGAGCATGGGCCGGCTC

GTCACTATCACGGGGATGATCGTCAAGGCCTCGGACGTCCGGCCCATGCTGCAGGTCGCGGCCTACACCTGCGACGTCTG

CGGTTCCGAGCTCTACAAGGAGACCCACCGGCAGCGGGACTTTGTCCCGGAAAAGGTCTGCCCGGTCTGCGTCTCGGGGG

GGACCAACCCCGGGGGCGGGGGCGGCCGCCGCGTCGTGGGCGTCCTCAAGCTGGAAACCCGAGGGAGCAAGTTCACCAAG

TTCCAGGAGCTCAAGCTCCAGGAGCTCCCCAGCCAGGTCCCCATGGGGCACATCCCCCGGTCCCTCAGTGTCTACTGCCG

CGGGGAGCTGACGCGGTTGGCGAGCCCGGGGGACGTCGTGACCCTCGACGGGATCTTCCTGCCCCAGAAGGTCAACGACG

GACACGGGGCCGCGGGCCGCAGGGCCGGCCTCATGTCGACCCTCTACGTGGACGCCCAGAACCTCTCGGTCCACAAAAAG

TCCTTCGACGACGCCGCCACGAAGCGAGCAGGAGAGGAGAAAGCCGGCGGGCTGGACGCCGACGTCCGCAGGGTCGCTTC

GGCCGAGGATCCCGTGGGGGTCCTGAGCCGTTCCATCGCGCCGGAAATCTTTGGCCACGCCGACATCAAGCGGGCCCTGC

TTTTGCAGCTCACCGGGGGGGTCTTTCGGAAGATGGCCGACGGGATGAAGATCCGGGGGGACATCAACATCTGCCTCATG

GGGGACCCGGGGGTCGCCAAGTCGCAGCTCCTCAAGCACGTGGTGAGCGTCGCCCCCCGGGGTGTCTACACGACCGGAAA

GGGGTCTTCCGGCGTCGGCCTCACGGCCTCCGTCACCAAGGACAACACGACGGGGGAACTCTCCCTGGAGGGGGGCGCGC

TGGTGCTAGCGGACCGGGGCATCTGCGCGATCGACGAGTTCGACAAGATGGACGAGGCTGACCGGTCCTCCATCCACGAG

GTCATGGAGCAGCAGACCGTGTCGATCGCCAAGGCGGGAATCGTGAGTCCTGGTTTGCGACGCGCTTGCCGTGCCGTGCC

GTGTGCGTTGGGTATGCGTGTGGTGCGGGAACACAGTGCTCTGGAATTCCGTGAACCGATGTGATGCGATSCTAGGACGC

CATGATTCGACAGCAACCCGCGAGCAATCGGTGCTGCGTGCCTGCGTTGCAAGGGTTTGCAATCAGTGATGATCACTGTC

CAACTCACCGACTAATTCGTTCGTTCGTCTTTCTTCTATCTCTCCTGCTCGCAGGTTGCCACTCTAAACGCACGGGCGTC

GGTTCTTGCTGCCGCGAACCCGCTATACAGCCGCTACAATCGGCACAAGTCTCTCTCCGAAAACATCAATCTGCCCAATT

CGTTGCTGAGCCGTTTCGATCTGATGTTTTTGATTCTGGACACACCGGACATGGACCGCGACATGGCACTGGCCCGCCAC

GTCACGTTTGTGCACCAAAACGAGGGCCTGGAGGCGCAACCGGAGGACCTCGACTCGGACGACGACGACGATTCCGGCAG

GGCCGCAGAAGAAAACTCGGATTTCAACGAGGAGAAGGGGATCGTCTCCCCCCGGCTTCTTCGGGAGTACATTTCTCGAG

CTCGCCGCCACGATCCAGTGGTTCCTCCCGAAGTCGCACCCTACATCGTGGAAGCGTACGTGTCTCTCCGAATGCAGGAT

CGTCCCGGMAAAAGTAACAACGGGAACAAAGTCGGGGATCAGACCGTCATGACCGCTCGACAGCTCCTTTCTATACTGCG

ACTCGGCCAAGCTCTGGCTCGTCTGCGCTTCAGTGATTATGTAGCCAGGGAGGACGTAGACGAAGCCATCCGATTGACAC

ACATGAGCAAGGCTAGCCTGTCCGAGGAGACAGGCGAAGACGGAAAGATAGTTCGACGGGAAGACGTCATGTCACGAATA

TTTAACATCATCAGAGACTATTCTGCGACGTCGAACAGTACCACGGTCGAAATGAAGCTCGCCGAAGCCATGGTTATTAG

AAAGGGTTTCACAGTTCAACAGATGCAATCCTGTTTGGAAGAGTACGAAGCATTGGAGATTATCCAAATCAATCAAAATC

GTACCCAAATACATTTTATCTCTTAATACTGGGTGCCTGATCCTGGACGCTTCATTGAACCCATAATTTACATCTGACT

>PSNMU-V1.4_AUG-EV-PASAV3_0068370.1 class=Sequence position=[PsnmuV1.4_scaffold_32-size_254018:145057..147795 (- strand)](http://gbrowse255.tgac.ac.uk/cgi-bin/gb2/gbrowse/maplesod_psnmu_v1_4_gbrowse255?name=PsnmuV1.4_scaffold_32-size_254018:145057..147795) *MCM8*

GCATGCCTTCTTTCTGGCCTTTTTGAAGAAGCACAGTACGGTCCAAGGCAGCCGCCAAGGAACGAACAATCATAAAGGAC

CAGACGAGGAACGAATTGACTTCTTGAAACAAAATCTGCGAGAGAGTTGTTTGGAGGTTTTTTATTTGAAATTTATCGAG

AAAAAAAGTCGAATGAACGAAAGCAATAAAGGAAAAGTACGGCTGAATGGAGGAAGAACAAGAATTACATTGCATCACGA

AGAGTTCTTGTCCGAGTTGAACGAATACGAGTTTCGTGGCCAATTTCGGAGCGACAGTAGCACCTGCATGGCTAATTGCT

ATGGAGCGTACTTGTATCACCGTCCACTAGAAGCACTACCTGCCGTGAATGTATCCATGGCTCTAGCTGTCGTAACATTG

TGGAGGCTTCGCGAGAATGCGTTTGTGGCGCCTGGAGTAGACGCAACGTATAGCAACACGAATGATACTTCCAGCAAAGT

GAACACGTTTCTGGATTCTTGTCAAATAGATGTTCGATTTGTACATGTTAGCCCGCAAATTCCCATGGCGGACATAAAGA

CCGGACTCGTGAAGAAATTCATCGCGGTCAAGGGCCACGTCGTAAAGGCACGACCGAGACGGCTCCGCGTTTCAACTGCC

GATTTGTAAGTTTTCTCTGGACAACGTACGTTTTATTCAATTTTGCCTGGATATTACATTTCCCATGTGGAACAACTTTT

CTGACAATCAATTAATCTATAGTGGGTGCCAAAAATGTGCAGCTATAGTTCACCACGTCTTTGCGAAGGGTCGATTCTCT

CTACCAACAAAATGCTCGGATCCAAAGTGCAAAAGTAGATCTTTCACTTTGATTCGGCCAACAGCTCGGTATACAGTAAG

TACATTCTCGTACTTGCTATTCATTCCGGTAAGCTTGCTCTTTCCCTTCTTACTATTTTTTCCAATATATTTGTCAATTG

AAAGAACGTCCAAGAACTAAGGCTTCAGGAATCTCAAGAAGAGAGTACATCGCATGCTGGACGAACGCCGCGACAGTTTG

AAGTCGAATTGAATCACCATCTAATCGACTCCTGTCGACCAGGTGATAGCATCTTGCTTGCGTGTCAAGTAGATGCCGTG

AACTCAGCCGTCGCTGCTGGAAGAGCAGGGAAGCGTGCCCAAGAGACAAGTACGTACAAGCTATTCCTCCAGGGTCATTC

TATCACCACGCTAAGCGAATCCAACAACCAAGTGCGTGACGACAAATTCAGTGCCGACTCTGCACAGGAAGGCGGCTCCC

AGGTCACTTATACGCAACAACAACTCCAGCAAATCACGCAACTCTGTCATGCTGATCATCGGTGTTTGGGCATGGTGGAA

CGACGAGCCTTCCCATTCGATTTGCTTGTTAGATCGCTTTGCCCGTCAATTATTGGTCATCATGCAGTCAAAGCGGGTAT

CCTTTTATGTCTTCTGGGAGGTACGCCTTCCGTCTCGGAGCAAATCGATAGGGGAAATACGATACGGAGCAATTCTCACA

TTTTGATCGTGGGCGATCCGGGGATGGGGAAATCCCAGATGCTGCTTGCTTCCACGCAGTTGGCGTCGCGGTCTGTATAC

GTTGGCGGCAACACTGCATCTACTACCGGGTTAACCGTTACTTTGACGAAAGAAGAAGGGGGAGAAACTGGAATAGAAGC

AGGTGCGCTCGTTTTGGCAGATCAGGGTGTGGCTTGCATTGACGAGCTTGACAAGTGCAAACACTTGGATGGGCTTTTAG

AAGCAATGGAGCAACAATGTGTTTCCATTGCAAAGGCGGGAGTTGTAGCTAGCCTGCCGGCAAGGTGCAGCATCGTGGCG

GCAGCCAATCCAAAGCACGGCAGCTACAATATGAGCAAAAGCGTCGCTGAAAATCTCAACATGGCACGACCCATATTGTC

ACGATTCGATCTTGTATTCATTCTTCGGGATCGAGCAGATAAAGATCAGGATCGTCTTGTTTCTAGCAACATCATGAATC

TCTACCGGAAGCCTAATGGAACGGGTAAAGCTGAAACTAGAGATGAATTCGAGCGCCTGTCAAATGAGCTTTCTTCTAGA

AGCAATGCTCAAAACGAAAACACAGGTCAAATCACCGAACCTAATCGAATTCCGTTGGAAAAGCGCCTTGCTTGGGTTAC

AGGTTTTAACGAGCCTTTGCCTGCGGGACTCGTCAAAGACTATATTGCATATGCCCGTGAATACTGCAAACCTAAGCTAA

CATCAGAAGCCGCTGTCATCCTTAAGGAGTACTACATGACTCTTCGCTACCCAGCGAACGGCCGCCACCACAGAGATTCT

GTTCCGATAACCACGCGTCAATTAGAGGCCCTGATCCGTCTATCACAAGCCCGAGCCAAGGCTTGTCTGAGAGAATTCGT

CTTGAAGGAAGATGCTCTAGATGTTGTCGAGCTGTTGAAAAGATCCGTGGAACAAGTCCACACCGACGAATTTGGTATGG

TAGATAGGAGTCGCGCTGGAGCAAGGGGCCAAAGCAATCGCAAATTGAGAAGAGAATTTGTACGAGAGCTCCACAATATT

GTAGGTATTGGGGCCGAATGTACATACGATGACTTGCTTCGCGTTTCCAATCGAGTGAACTGCCCTCTTAGTGATTTCAA

CACTATAATTGATGATATGCGAAACAATGGAACTTTGATAAAAAAGTCGAATATGAAGTATCAAATTGTATCTTAAATTT

ATAAATCTCCGATACAGTT

>PSNMU-V1.4_AUG-EV-PASAV3_0056900.1 class=Sequence position=[PsnmuV1.4_scaffold_269-size_70619:31206..34345 (- strand)](http://gbrowse255.tgac.ac.uk/cgi-bin/gb2/gbrowse/maplesod_psnmu_v1_4_gbrowse255?name=PsnmuV1.4_scaffold_269-size_70619:31206..34345) *MCM9*

AAGCAGCACACCGGCAGGACACACCGGGAGCCGTGCCCATGGATCCGGTGGACCGGCCCAGGACGACGAAGCGGAAGCAA

GAAAGAGGAGTCCACTGTTCACGGCAGGCGGCCAACACGCAGGGATGCCGGGCCCGTCCGGTCCGGCTGGAAGCGCCCGG

GGCCATCCCTGGACCGCGGAGCGTCTCTGCCGGGCGTCCTACCAGGAGCTTGGGACTCCCGCCGAGGTGGAGAACCTGTT

CAAGCAGCACCTCCTCTCCTCGTGCCGGTCTTCGCTAGAGGTCCTGCTGTCGTCGGAGAGCCGGACCACCCGCGACTACT

CGCTGGAGATCGACGCCTTTGATCTCTTTGTGGCCAATCCCGTCCTCGGCCACCTCCTGCTCAAGTTTCCCAACACCCTG

GTGCCGCTGCTCGAAAAGGCCATCGTCGAGGCCCAGAACGACCTCCTCGGCCAGCTGGAAGCCGGGGAGGAAGAGGAGGG

GCTGGCAGCAACCGGGGACGCGAACCAAAGCCACCGTTCCGTCAAGGGCAGGGCAGGGTCGCGGGTCCACGCCCGGATCT

TCCACCTCCCGCCGACCTGTTCCAGGACGTCGGTCGCCTCCCAGGCCACCGACGTGGGGAAGATCGTACAGCTCTCGGGA

ACGGTGGTGCGGACCTCGGCGGTACAGATGTACGAGTCGGCCAGGACCTACAGGTGTGCGGGGAAGAACGGCTGCTCCGG

GACCTTTGTGCAGGAAGCCGACCTCGAGCAGCAGAACAACGCCATGGTGGTCCCGGAACGCTGCCCCCTCTTCTCAAAGG

ACGGCAAGCGGTGCAGCGGGACCAACCTCAAGGCCACCAAGGACGGGTCCGTCCACACCGACTACCAGGAAATCAAGATC

CAAGAGTCGGCCTCCAAGCTCGGCGTGGGCAGCATACCCCGGTCGCTCCTGATCAAGCTGCAGCACGACCTGGTGGACTC

GTGCCAGCCGGGGGACGAGGTGGTGGTGGTGGGGATCCTGCTGGCCCAGTGGCAACAGGCGGCGCAGCCGGGCCTCGAGT

GCCAGGTCGGCATGGCCCTCAAGGCGCACTCGGTGCGGGTCACACAGGAAAACGGGGGCTCGGCTTTTGTCGAGTCGTCC

GTGGGGGAGCTGGACAAGTACAAGAAGGAATTCGACGTCTTTTGGGACGCCAGGGGGAGGCGGGACTACCCGATCGCGTC

GAGGGACTTTATTTGCACGGCGGTGTGTCCCAAGCTCTACGGCCTCCAGGTCATCAAGCTCGCCCTGTTGATCACCCTCA

TCGGCGGGGTGTCGTCGGGTTCGCTGGACGGTGCCAGCAGCGGCGACATGAATGACAACGACAACGGCGATAACAACAAC

GACGACGACGACGACGACGACGACGACGACGAAACCGATGCCTGCAGGGGAACGGGGATGCCCCCGCTGTACCACCACGC

GATGCGACCGGAATCCTTTCGGGTCCTCTCCACCGGGGATTCCTCGTCGCACAACGGGAACATTTACGGCGAGAACGCCG

GCAGCGGCCGGTCCGGGACCAAGACCCCTTCCGCCGGGGGTGAGACCATTGTAACGACCCGGCGGAGGGACATGTCCCAC

ATGCTCCTCATCGGAGACCCGGGGACGGGCAAGTCCCAGGTGCTGAGGTTCGCGGCGGCCCTGTGCCCGCGCTCGGTGCT

GACGACCGGCGTGGGGACGACATCGGCCGGGCTGACCTGTGCCGCGGTCCGCGAGGGCAACGGAAAAGAGGTGCGTGGGA

GCGTGGGTACCGTTCCACCCGAAGCAGAACCCGACACGCCTGCTTTCCGCGGCGGGCAGAGGCACGAACCGATCGCACCG

GTTACGGCATTCCAATGTGGCCTTTCTTTGTTGCTGACTCTTCTCTTTCTCTCTCGACTTCTCGATCTCTCTGTGCCATG

TTTTGTGTCTCAACACCGATAGTTTGCTCTGGAAGCCGGTGCGCTCGTATTGGCCGACAAAGGCATTTGTTGCATCGACG

AATTCGGTTGCATTCGGAACGAAGACCGGACGACCATTCACGAAGCCATGGAGCAGCAAACACTCTCGATAGCAAAAGCG

GGCATCGTCTGCAAACTCAACTGCCGCGCCACCGTGATTGCCGTCATGAACCCACGGAACTGCATCTACGACAACCACAT

GTCGCTGGCCCACAACACCGGGCTCGGGACGCCGCTCTTGTCGAGGTTTGACCTGATCTTCAAGCTGGTGGATTCCTCCG

ATGCAGAAAGGGACAGCAATGTGACGACGTATCTCCTGAACCGTGCCATCCAGGGCACAGGTTTTGAATGCTCGAGCACT

TCGGCCGGAATCGCCGGGGAAGACACCCCCTGGTCCATGGAAAAGCTGAGAGCCTACATTGCTATTGTCAAGGACCGCTT

CCAGCCCTCCATTAGTGACGAAGCTGCACGCCTGCTCGAGGCGCACTACGAAAAAGTGCGATCCGCACAGTCCTCTTCGA

TTCCCGTCACCGTTCGCTTCCTCGAGTCCCTGATTCGCCTGTCGCAGGCCCACGCCAGGCTCATGTACCGCAACACCGTT

CACCTGGAGGACGCCGTCGCCGTGTTGCGGGTCATGGAATGCAGTGCGCATGCCTACGGAGGTTTTGACGGAAACGTGGA

CGACCCGGAAAACATCTTGTACGGGGATCCCATGCACATGGATACATGTGTTTACGCCCCGGACGAAGACTTTGTCGTCT

TCGAGTACAGCATACTGAAGCGCTACGACATGCTGGAATACATGTCAAACGATCTGCGAAACCAGGCACTGGCGCTGCTG

GAAGGAGAAGACGACAACGATGCGATGGGATTCGGGCCAACGACATGGAACAATATTGCGAATCCGCACGATCGGCCGGG

GACGGTTGGAGAAGACCACTACGGGCGTTCGTACTTTTCGCCGGGTCCACCGAGCCAATCGCAAACCCAGACCCCCAACA

CAAAACGGCGAAAAGTGATGTGAACAACGGGATTCTCCCATTACATTGTCCGTGATGGTTCCCGGAATTTCAAGACCAAA

AGCTTGTGAACGGTGTGCAATCGATCGATCGAAAGAGAGGTGGCAGGACAGTAGTGAGCGCAACCCAGCTGTACCACTAG

AATAGAAAGAACAATTTGCT

>PSNMU-V1.4_AUG-EV-PASAV3_0116990.1 class=Sequence position=[PsnmuV1.4_scaffold_81-size_176161:113406..118305 (+ strand)](http://gbrowse255.tgac.ac.uk/cgi-bin/gb2/gbrowse/maplesod_psnmu_v1_4_gbrowse255?name=PsnmuV1.4_scaffold_81-size_176161:113406..118305) *SMC1*

GGAACACAGGGCACGTCACGCTTTGCACGGCACCGCACGGCACGGCACCGCACCAAACGACGGACACCACCATGCCGGTC

ACCTACCTCGAACTCGAGAACTTCAAGTCGTACGGAGGCCTCCAGCGGATCGGTCCCTTCCAGTCGTTCACGAGCATCAT

CGGCCCGAACGGATCCGGCAAGAGCAACTGCATGGACGCCCTCAGCTTCGTCCTGGGCGTCCAGTCCCGGGACCTRAGGT

CCAGCCAGATGAAGGACCTGATCTTTCGGCCCCCSCACGGGAAGTCGAGGGGGAGGAACAAGCTCAGCGCGAGCGCCGCC

ATCTACTTTGAGGAGGAGGAGGAGGAGRRMRRCGRSGAGRRSRRCGRSGRRGAARRMAGMRRARGMRGCRRMGARRRSSR

MSMSRARGMRMRASSSARMSSMMGRRRCMGYSGCCGSAMMCSWGSCMGCASMMSMRMCARCAGCAGCAGCRAGACRCCAA

CGACCAAAACCACCACCAAGTTCCAGCGGACGATCCATCCCAACGGCAGCGGCGACTACCGCATCAACGACCGGGTCGTC

TCCTACAAGCAGTACGAGGAGAAGCTGGCCAGCATCGGGGTGCTGGTGAAGGCCCGGAACTTTCTGGTCTTCCAGGGCGA

CGTGGAGTCCCTGGCCCGCAAGTCGCCGAAGGAATTCGTCGMCCTCCTCGAACAAATCTCGCAGAGCGCCGATCTGAAGG

ACCCCTACGAGGCGGCCCTCGTGGCCAAGGARGAGGCCGAGGCGGAGGCCCTCTTTTCCTACAACAAGCAGAAGGGGATG

AAGGGGGAACGCCGGCTCCTGAAGGAGCAAAAGGAGGAAGCGGAGCGCTTCGACCGGCTCCGGGCGAACAAGCAGCAGCT

GCTGACGGACTACTACCTCTGGCAGATCTACCACATGGAAACCGACCGAGAAGAGCGGGAGGACCACCTCTCGGAGCTCC

GGGCCGAGGTGGAGCAGGCCGAGTCCGCCGAGAAGGARCAGATCGCCGCCCTCAAAAAGGCCAAGAAGAAGGCYTCCAAG

GCCCGGCGGRCCCACCAGGAGGCCGACTCGGAGCGCGTCGAGCTGGCGGCGAAAGCCGACGCCCTCGAGCCCTCCCTCAT

CAAGGCCGAGGAAGAAATCAAGACCTTCCAGAAACAGATCGCGRGCGACGAGGCGCAGATCGCCAAGCACCGCAAAAAGG

CCGAAACCCACGAGCAAACCCTGGCGGACCTCGACGAGGCGATCGAGGAGCAAACCAAGAACCTGAAGGATCTCGGCGAC

GAGTACGAGGCGGCCAAGCAGGACGCCCTGCAGAACGACGACCASCCCACCCTCACGCAGGCCCAGGAGGACGAGTACGA

GGCGGTCAAGGAGGCGGCGGCCGCCGCCAGCGTCCGGCCCCGGCGGAGGCTYCAGCAGATCGCCCGGCAGCTGGAGTCCT

CCCGGGCGGCCGCCTCCGAGGCCCAGAGGCAGCTGGACGAAGCCAGGGCCCACACCAAAAGTCTGACCCGGGACCGGACC

GAGCTGCAGACGCGCCGGGACAAGATCCAGAGCAGCATCCAAAAGACCGAGGCCGACCGCAAAAARGCCCAGGACGAGCT

GAGGGAGGCCAACCGCCAGAACGAACAGGCCAACCAGCGCAAGGCCACCATCGACCTGGAGCTGGAGCAGATCGCCGCGA

CCCTCCGGGACGCCAGGGACAACCGACGCAAGAGCCGGGACGAGGAGCGCCTCCAGGACGCCATCAAGGCCCTCAAGMTG

CACTTCAAGGGGGTCTACGGGAGGCTCGTCGATCTGTGCCGACCGACCCAGAGGCGGTACAACCTTGCSGTTACGGTGGC

CGCCGGGAAGGACATGGACGCCCTCGGTGCGTTTTCGACTCGTTGTGCACTGCGCATCGCATCGTGTGGCATTGCATTGT

GTGTCGAGATGTTGCTGTCTTGTTTCGATGGCTTTGGTGTTGTATTTGTATTTGTGTCGCGATTTTATCTCGTGGCCTCG

TGTCTCTCTATCACTCATCTCAAAGTTTTTTTTGTCGTTGTGATTGTCGTTGTTATTATTGTTGTTGCCCGGTGTAGTTG

TTGACACCAAAGCCACGGGCATCGAATGCATCCGGTATCTCCGGGAAAACCGCATCGGAACCGCGACCTTTTTGCCCCTG

GACAGCCTCCAGGCGCCCTCGAGGGAATCCACGGAACGCATCCGTGCCCGCCTGGCCCAGGACGGGCGCTTTCGGTTGGC

CGTGGACGTGATTTCCAGCGATCCCGCCATCCGCAAGGCCGTCCTCTACGCCGTGGACAACACGGTGGTTTGCGACAACC

TCGACTCGGCTCGGCAGCTGTGTTTCGGTTCCGGGCGAAGAGGCGGTGGAGGAGGACAAGACGGCAGGTCTTCCATCAAG

GCGGTCACGCTGGACGGTGCCGTGATTTCCAAAGCGGGAACCATGACGGGYGGTGTCACCAGCGAGGAATCCAACAAGGC

GGGACGGTGGGACAACCAGGCGATGGAAGACCTCCGCCAGAARAAAGAAGCCCTCGAAGACGAACGAAACAACCTGGATC

GAAACCAGAACACGGCGGGACGTCAATCGATGGGTCGGTCCAATCGCATCGAGGAACTGCGGAACAACTACGATTCGCTC

AACAACCGCGCCGAGTATTCCAAGAGCGACATGGAGTTTACCMGCAAGGCACTGGCCGAGAAAAACACGCTGCTCAAGTC

GATCGAGCGCAAGGTCCCGCAGCTCGAGGAAAAGGTGGCTCAGCTCGAGAAGGAACTCGAATCGCTGAATGCTACCAAGG

AGAAGGCCATCGCGGAGGTCCACGCCGCCAAGGACGAGCACCTCGGCCCCTTCCTGGCCGCCACGGGGCTGAAGGACCTC

GAGTCCTACGAGCGGGCCACCYGCGAGACACGCGACGAGTTCAACCGMAACAAGCGGCAGCTCGTTGAGCACATTGCCCA

GCTGGAAGAAAAGAAGAGCTACGAGTCCAAACGCGACCTGAAGAAACCGATCGCCGCGGGAGAGAAACGGCTCAAGAACC

ACCAGAAAAAACTCACCGATGCCAAGGCCCGCCAGAAAAAGCTCAAGAAGGAAGGAAAAGCAGCGAGGGAGAACCTGGAA

GCCGCGGAGAAAGCCGTCACCGAGGCGCAGGAGATCGAGCTGGAAACCGAAGAGACGGCGAAAAAGCTCCAGAGCGAGTT

CAAGGAAGCACAGAAACAGAGGAACAACGTGAGCAAGTCGGTTGCCACGGAAGAGTCTGCGCTGGAACAACTCCGCGGGC

GTCTCGCCGAAATTCTTCAGCGGGCTCGCTTGGAAGAAGTCTTCCTTCCGGTCAAGGGTGCGGCGACTTCCGGCAGAACC

ACTCGGTCCGGCCGCCAGATTGGTGGAGGGGACGATGCCAACGAGGAGGAGGASGACGACGACGAGGACGAAGAGATGCY

CGAAACCCAGGAAACCGACCACCGAATTCCGTCCGCCACCCAGTATTCGCAGGAAGACAACCCCAACGTGGTTGCCGACA

AGAACGAGGCCGCAAAGCTCGATTTCGGCAAGCTCCGCTCGGACCTGAAAATGCGTCTGTCGAGTCGGGAGACGGCGCAG

GTCAAAAAGGACTTTGAAGAAAAGCGCCTGAAAGTCGATGCCGAAATCGAGGGCATCGTTCCAAACATGAAGGTGAGCAG

TAGGCAACGTTGTTGTAAAATCTTGGACTGTCGTTGAGAGTGTCGGTCGTTTCACAAACTCTTGCTGTCTTGTTTGTCTT

TTCATACTTGTRTTGTGCAGGCCCATGGCGCTTTTTCGGCCATCACAGAAAAGCTGAAAGGAACTGATTCCGATTATCAG

CAGGCCAAGGAAAAGTCGCGAAAGGCTGCCGCAGAATTCCTAAAAATCAAAAAGAAACGCACAAAGAAATTCCTCGATGC

CTTCAACCACATCGACAAGGCTCTCAAGACGATCTACACGGACATGACGAAATCCAGCAAGCATCCCCTGGGTGGCAATG

CTTACCTGACGCTGGACGACACCGAGGAGCCTTTCAAGGGAGGAATGAAATTCAATGCCATGCCACCCATGAAGCGATTT

MGGGATATGTATCAGCTGAGTGGGGGAGAGAAGACGGTCGCGTCTCTGGCTCTTTTGTTTGCCATTCATTCGTATCACCC

CGCCCCGTTCTTTGTGATGGACGAAGTGGATGCTGCTCTGGACAATAGTAGGTTACGYTAATGCCTTGCATGGTGTTGTG

TCTTMRAATGGATCAATKTGTGGCTTCGTGTAGGTTTGATCCCTTGTGCACGGCACGTCTTTCTCACAACCCCTTTCTCK

CTTTCTCCTCAGTCAACCTGCGAAAAGTTTGCAACTATATCCAACAACGCAGCCAGGTAGACTTTCAGTGCATCGTCATT

AGCCTGAAAGACATGTTTTACGAACGAAGCCAGTCCCTGGTGGGCATCTGCAAGGACGTGGGGACCAATTCGAGCCGCAC

GCTGACCCTGGACCTTACTCGGTTTGACAAGCGAAAGCAAGACGAGGASGAAGAGCGAGCCGCGACCGACGGGAAACGAC

GGACCAAGCGCAAGGCGAAGAGTGAGGGCCAGTCGTCCTCGCGGAAACGCCTGGCTTCGGACAGCCCCGGAACGATCACA

ACTCAGTAGTTTCGCTTCGTTTCGTTCCGCACAGCTTGTTTCACAACAATCGCTTGGCACCGCCCTTTCGAATGCGTAGG

TAGCGTACAGTTGCGACTGTGGGGATCGTCGCAACTAGCATCAGAAGGCAACGCCACTCCAGTGTTTACAATGTAGTGTT

TTGCTCTTGCGTTCAAACGCCATGCAATGYAAATGCAATACGACACAACCCTTTACATTATCACGAGTGCATAATACTAC

AGTAAATTGTATTCTAAGAA

>PSNMU-V1.4_AUG-EV-PASAV3_0096310.1 class=Sequence position=[PsnmuV1.4_scaffold_52-size_208590:166339..169825 (- strand)](http://gbrowse255.tgac.ac.uk/cgi-bin/gb2/gbrowse/maplesod_psnmu_v1_4_gbrowse255?name=PsnmuV1.4_scaffold_52-size_208590:166339..169825) *SMC2*

TGGAGACTACAGTATCATACAACAGGTGTTGTTGCGGACACTCTGTCTACCTATTTCATTTACATGTCACGTGACACAGC

CACTTTTTCTGTGAATAAAAACAATGCACATCCAGCAGGTTGTGATCGACGGATTCAAATCCTATGCTCGTCGAACAGTG

GTGGAAGGGTGAGTCGATCTGACGATTTGCTTGACAGGATTTAGTACATGGCTCCGCCATCATCTCATATATTTTATGGC

AAACTTCACGTCTTCTTTCTCGCAGATTTGATCCACATTTCAACGCAATCACGGGTTTGAATGGTTCGGGAAAATCAAAT

ATTCTAGATTCGATATGTTTCGTCTTGGGGATTACAAATTTAAGCCAGGTTCGAGCTGGTAATCTCTCCGAACTTGTTTA

CAAGCAAGGGCAGGCTGGTGTTACCAAGGCTAGTGTTACTGTCGTATTCAACAACGAAGATGAAGCGAGCTCACCAGTTG

GTTACGAACAATGCAAGGAAGTCACCGTTACTCGTCAGGTTTTGCTTGGAGGAAAATCGAAATACCTCATCAATGGGCGC

AATTCACCTGCTGGTCAAGTTCAAAATTTATTTCATTCCGTACAACTCAACGTCAATAATCCTCACTTTCTCATTATGCA

AGGTAGAATCACCAAAGTACTAAACATGAAACCACACGAGATCCTAGGAATGGTTGAAGAGGCTGCTGGGACACGAATGT

ACGAAACGAAAAGAATTGCTGCAATCAAAACTATCGATAAAAAGCAAGCTAAGGTGGACGAATTGAATTCTGTCCTGTCG

GAAGAAATTACTCCTACGTTGGAAAGGCTGCGAGGTGAGAAGCAAAGCTATCTTATGTGGAGTAAAAATAACGCTGATAT

CGAACGTATTGAACGATTCGTGGTTGCAAGTGATTTTCTCAGAGCGCAACAAGCATTAGACAATAATGTGGAGGGTTCCT

CTGAGATGGAGGATAAAGTAACTGAATTAGAGACTCAGGTAGAAACATACGAGGGCCAAATAGAGGAACATGACAACGCT

GCGAGCGAATTATCATCCAAACTCAAGGGGGAGTTTGATCAGTCACATTCGAAGCTGAAAAAGGAGGAGGAGAAACTATC

AAAACAACTTGTTAAGGTAACCTCCACATGGCAAAATAGTCAGGAATTGACAAAGAAAGCAGAGACAGATCTCGCAGATG

CTAGAGCGCTTGTAACTGAAACAAAGAACTTATGTAATGAAAAAGAAAAGCAGATTTCTCAAGATGCGAAAGCTGTTGAG

GATATTGTTTCTGCAAAAGAGGAAGCAGAGCGTACTCTAGAGGACCTCAGCTTGAAGTTTCAGAATATGTCGGCCGGAAT

ATCCATGTCTCAAGGAAACGAAGGTATGACTCTTCCGGACCAGATAGCAAAGGCTCATTCCGATTCTAAAACAGCCGAAG

CCAAGGTCAAACAAGCGACGATGAAGATCAAACATTTGACAAAATCACTGACGGTAAGTATTGTCGTGATCAAAATATGC

ATCGAGATTTGTAATTTCTCTCTTGAAGGAACTCCTACTTGCTAGACATTTTATTCGTGCCCTAACCTAACTGTCTGGAA

CGACTTTGTAGTCTGTCGAAAAGCAAATGAAGAAAGAGCAAAAGAGCGCAGATACGCTAAACAGACGACGTGAAGCATCA

ACTCAAAAGGCTGAAAAATTTCGAAAGCTGCTTTCAGAACTTAATTTTGATCAAGACGAATTCGCGAAACTAGATCAAGA

AAAAGATCACTTGACTTCTTCTGTGGAAGAACTCAATGGCATCGTTGAAACTCTAACAGCTCAGCTTGGAAGTCGTCTTG

CATTTAACTATTCAGATCCCGTACGTGGGTTTGACCGCTCCAAGGTCAAAGGTTTAGTGGCCAAGCTAATCCAAGTGCAG

AACCCAGTTCATGCTACAGCTCTCGAAGTTGTTGCTGGAGGAAAGTTGTATCAAGTAGTGGTTGATGAGGCGATCACTGG

AAAAGCTATTCTTGACCGAGGGAATCTAAAGCGCAGAGTTACAATCATTCCACTCGACAAAATTCAGCCACGACGAGTGA

CTGCTGCTGCGTGCAACAAGGCAAACCAAATTGCAAATAGTCTTGGTGCGAAGGCTTGGCCCGCAATCGAGCTTGTAGGA

TTCGACGAAGAAATTCGTTCTGCTATGGAATATGTTTTTGGTGCATCTATTGTCGTGGACGGAGCGAAAGCTGCGAATCA

GATATGTGATGCGACAAAAACCAGAACTGTGACATTAGATGGCGATGTCTATGATCCATCTGGGACTATTACCGGAGGGT

CTAAGAACAACCTTGGGAGTACACTTGTAGAACTTACGAAGCTACTCGAAGCCTCAAAGCAGCTTTCTGCGGACAAGGAA

CGGCTTTCAACCGTGTTGACGGCCCTTCGTGATTTGAAAGGGAAGTCGTCCGAATTCGATAAATTGAGTCATAAGCTTTC

ACTCGCAGAAGCAGAACTCGAAGGAATTAAAAAGCAGGTCTCCCAGACATCATATGGAGTCCTAGAAGAGAAATTTACCT

CCATGTCAAAAGAACTGGAAGAAGCCAATGAAGAATACACTTATATGCAAGATGAGCAAAAGGAAAAATGGGACTTGTAC

CACGAACTGAAAGAAAAAGAAGTAGAACTTACTCAGCAACGCGAGGACATGCTTGGTCAAATCGAAAGTGATATCAAGAA

GGCCAAGAAGGCAGCTGCCGATGCAGTTAAAAGAGCTCGTGAAGCGGAGTCGTCCAAAGACACTCTTCTTATGGAACTTG

AGAGCTTGAAGGCCGACGTAGTTTCAGCAGAAGTGGCTGTCTCAACTGCAGAGGCGAGCTTGAAGGAAGCCAACGAAGAA

GAGATAAGCAAGGAAATTGTAGTTGGAGAAGTGAAGGCATTATGGGACGAAGCAAAGGCTGCACAAACAAAATTTGAGGA

ACGAATTACACAATTCTCGTCAGAACTGGCTGAAATCAAGCGAGAGAGAGCTAATTTAACGAAGAAGGCAGAGAAATGCA

CCCTCGAGGCTAAAAAGCTTACGGTGTCGATTTCTCGTATTCGGAAAGAAAGACAGAGTGCTGAAAAAGTTGTCATAAGC

TTATTAAAGAATCATCCGTGGATCGAAAGTGAGAAGAGTGCTTTCGGTGTCGAAGGTGGCGACTACGACTTTACAGCTGC

GAACCCCGATGATATGAGTGCACAACTGCACGCATTAAAGAGCGAACAGGAATCATTGGTACGTTTTATCATTCTTTATC

ATAGTTTCACATGACCCGACCGGCAAGTGTACAAGACCTGTTGCAAAAGCTAACTAATGAATTTCAACTTTCGTTTTCCT

AGAGCAAGAAAATCAACAAAAAGGTTATGGGAATGATTGAGAAGGCCGAAGGAGAATATACCGAGCTTCTCCGAAAAAGA

AAGGTGGTTGAGAATGATAAGAAGAAGATTAAAAGCGTTATTGAAGA

>PSNMU-V1.4_AUG-EV-PASAV3_0079810.1 class=Sequence position=[PsnmuV1.4_scaffold_396-size_40476:1469..5935 (- strand)](http://gbrowse255.tgac.ac.uk/cgi-bin/gb2/gbrowse/maplesod_psnmu_v1_4_gbrowse255?name=PsnmuV1.4_scaffold_396-size_40476:1469..5935) *SMC3*

GGCACCAGTCAGGACAACCACGAAAATCCGATAATCCGGAAACCAACTAGAAAAAAATACGTAACAAAAAGGAAGCCAGG

AAGCAACCACCCAGAAACCACGGCACCATACAACAATGCACATCAAACAAATCACGATATCGAATTTCCGGTCCTTCCGC

CAGCAACCGGAAATCAACCCATTTGCMGCGACCACCAATTGCGTCCTGGGGAGAAACGGAAGCGGAAAGTCGAATCTATT

CGATGCCGTTCAGTTCGTCTTGTTGGCTCCACGKTTTGCAACGCTGAGATCGGTGAGTGGTGGAAGAGTGGTGGAACTGT

GTACTCGTAGTGTGTTGGAAAATATTTATCACCAGATGAAAGCGCGGTGAGAGCGTTTTGCATTCATTTGCCGTTGTTTT

GCGTTGTTGTACCATGTTTTATCACGCCATGTGATACCAAAAGGTATACGTAGGCCGCTTCGTTTCCAGCGGTACCCATT

GCGGTGACGATCGCTTCTTCGCCCAGTCTCGTCTGTTGTATCATTGATCTTTCACCGGCTTTCCTTCTCGGAACTTGATC

GAAGCGTGCTCACACATTATTCGCTTGCGCTGCTACGAACCCAACCGAACGGAATCGAACACAACACAACACAACACAAC

ACAACACAACACAACACAACACAACACACCAGGAAGAGCGCCAGGCACTGCTGCACGAGGGATCCGGATCCGCCGCCGTC

AACGCCTTTGTCGAGGTTGTCTTTGACAACTCTGACAATCGCTTTTCACTGGAACACTCCGACGAGGTCGTCCTCCGCAG

AACCGTCGGCAGCAAGAAGGACGAGTTCTTCCTCCAGAGAAAACGGGCCACCAAGCAAGAAGTCCAGTCCCTGCTCGAGG

GGGCYGGCTTCTCCAAGTCCAATCCGTACTTTATGGTCCAGCAGGGAAAGATCCAGAGCCTCTGCCTCATGACGGATGCC

CAGCGCCTGGAGCTGCTCCAGCAGGTAGCCGGCACCACCCTCTACGAAGACAAGAAATCGGAATCCCTYGTCAAGATGCA

AGAGAATTCGCAGTCCATCGAAAAGATCGACTCCATCCTCGGCGACATCGATGAACGCCTCAACGAATTGCAGAGCGAAA

AGGAGGAGCTCACCATCTACCAGTCGCTGGACCGGGACCGCAAGGCCCTCGAGTATACCCTCTACGACAAGGAGCTGCGC

AAGGCGCGCCTYGTTCTAGACAAYATCGAGCACAAGCGGGTGATCCACCTGAGGGATCTCACGGAACTCCACGAGCACTG

CAAGAAAACACACGACGACATCCAGAACGCGGACGCCGTTCTCAAGATAAAGTCGGAGAAACTCAAGCGGAACCGGAACA

ACCGAGCCCTGCTGGAGCAAGACCGGCGGACCGCCTACAAGCTCAAGGAAGATCTTGTCCTGCAGTGCGCGGAACTGGAG

GGCACGGTGGGGATGATCCGAGACACGGCTCAGCGCAACCAGGCCGAGCTGGAAACACTCGAGGGGGAAATCGAGTCGAC

GAAGGARAGACTCGAAGGAAAGAAGACGCTCCTGAAGGATGAAGAAGATTCCCTCCGAAAACTGGAACAAGAACGGGACC

AGGCCWYCCGCAAGATCGAYGGTCTCTACGCCCGGCAGGGCCGGGGAAAGGAATACAACTCCAAGGAGGAACGGGACGAA

TCCATACGGGTGCAAATCAGGGAATTGGAGGGCCAGCAATCGCAGAAACACCAGACTCTTTCGCAACAAAGGGACTCGCT

TGCAAATCTACGGCGCCAGGCTGTCGACCTCGAGGCCTCCATCGAATCCACGAAGGCCGACGTGGGCACCAAGCGCGAGG

CTCTCCAGGCGCTCCACAAGCGACTGGACGACCAGCAAAAGCAGCGCCTGAAGCTCATGGACCTTCGGAAAAACGCCTGG

CGAAAAGCCGAAGCCCTCACGGAAACACTCGCAGAATGCCGGGAAGCCAAGGGAAGCACCTACGCCAACCTCTACCGAAG

CATGCCACGAAATACCTCGCAGGGGCTAAGATCGCTCCAGACCATTGTGGAGCGAGAGGGCCTGATCCGCGGAGAGCAGT

ATTTTGGCATGGTCATGGAAAACATGAAACTCAAGGATCCCAAGTTTCAAACGGCGGTGGAGGTCGCGGCACAGAACGCC

CTCTTTCACGTCATTGTCGATACGGACGCGACCGCGGCCCTGTTGATGAAGAGGCTGGAAGAGGGAAAGCTCGGGCGCGT

GACGTTCATGCCACTCAACCAGCTTCGCGTACAGGACAACATCCAGTATCCACAATCCGATGATGTTGCCCCGCTCTTGT

CGACCTGCATCGCGTACGAGCCGAGAGTGAAACGTGCCATGCAGCAARTATTCGACCGGAGGCTGCTGGCCCGGAGTCCC

GAGGTTGCTTCGGAATGGTCGGCAAAACTCAGGATGGACTGCATCACCCTCGACGGTGATCTKTGTTCTCGGAAGGGAGC

TCTCTCKGGGGGATATGTCGACGTTCAGAAGTCCCGCCTTAAGGCCTACAGCGCAAAAACGATTGCCGAAGAGGCCTACC

GCAAGGCCAGGGAAGAACACCGCAGGGTGGATGCCGAAGCCAAGCAAGCCGAACAGGACGTCACAAACGTCTCGGAAGAA

ATATCCCGTCTTCAGCACAAACAAGCCCAACTGGCACGAATGATCCAAAACCAGGAGGGCGGACTATCCCAGTCGCAATC

CCAGATGGGGCAATATAGTAAGCAGGCGGATAGATTGGAATCCCAGATAATTCCAAGCCTAGAACAGAACCTCGAAGGCT

TACAGTTCGACATTGCCCGCTTGGGAGAAGAGGTCGGGACGGAGCTGACCTCGAGTTTGTCGGAGGAGGAACGGRCATCC

CTCAATGAGYTAAAAGCTGTCAAGGAAAAGCTTGTGGAAAAGATTGCGGATCAGCGCGATACTGTGGAAGAACTCCGTAC

CGAACGCACCCGGCTAGAATCCGTCTTGGAAAACAACCTTTACAAGCGTCGGACGGAACTTACACAGGTCAACGACACGC

TTGATACCAACGACGGCGGCGCAGCCATGGGAGGCGGCGTCACGACGAACGCGATGCTGCAGGAGCAAACGGAACGCCAG

CTCGAGGAACGCCGATTGGCATCGCGGGAAGCGATAAGGGACGCCGACGAGTTGGAAGCCAAGCTGGAGGAAATACGAAA

AACAGAAGAGGAACTCAAAAAAGAGCTCATTGCTGCCAAGAATGAGCTCGAACGSCTCAAAACCGCAGACAACAAAAACA

CGCGGGACCTCGACGATGCGCAACGCCGTGCCGAAACGCTCATGGGGAAGAAATCGCTGAACATTGGACGCCGTGATAGC

TACATGAGAAAGATACAGGAACTCGGCTCTCTTCCCCCTGATTCAGAACTCCGAAACTACAAGAATCAAAACATTAGCGA

CCTCGAGAAATCATTGGAGCGCGTGAGCAGGAAACTCAAAAAGTACTCCCACGTAAACAAGAAGGCGTTCGATCAGTACG

TCAATTTTTCGGAAAAACGAGAGTCACTGATGGAACGCAAAGACGAGTTGGATCGAGGTGCMGAAAAAGTGAAAGAACTG

GTTGAGAGTTTGGATCGAAAGAAGGACGAAGCTATCAACCGTACCTTCCGAGGGGTGAGCAAGCATTTTGCAGAAGTCTT

TAAGGAACTCGTGCCACTGGGAGATGGTGAACTCATCATGCGGACAGCCATCGACGAAGCATTGGAGAGGGACGACRCCG

AAGATGATAACGGCAGCGACGATGAAACTTTCCGAAAGAAACAACTCGACAATCCGGACGTCAGCCTTTATCGTGGTGTC

GGTATCAAGGTTCGTTTCTCTGCAGTCGGAGAGAACTTTATAATGAGCCAATTGAGTGGAGGSCAGAAGGCTTTGGTGGC

AATGGCTCTCATCTTTGCAATTCAACGCTGTGACCCTGCACCCTTTTACATTTTCGACGAACTCGATCAGGCTCTCGATT

CCACCTATCGCCAGGCGGTTGCCAATGTGATTTATAACCAGGCCAATAATCCAGACAATCCAACGCAATTCATCACCAGT

ACCTTCCGCCCAGAGCTTGTTCGCATTGCGAAGAATTGTTACGGCATAAGCCACCAAAATAAGGTGAGCAGCATCCACTC

GATGACCAAGAATGATGCCTTGAAGTTCATTGCGAACTTGATGAATGAGGAAGAGGCGGTCGGGAATGTTTCTACCACTC

GTTCGTCGTTGTCGAAGAAGGGGAGCAAAAAGCGCAAATCAAACGACATMGAGACCGCTGWCGAAACAAGCCAGATGTCT

TATGGACTCCAAACCCAACCTKTGGAAGAATAATGGATGAATAATCCAGAAGAATTGTACTATTTATGCGGTGTTTCAAG

TAATTGCTTTCGGGTTGCAATTACCATYATGGGAAAAAGTAATTGTAAATGAACTATGATAAATGAA

>PSNMU-V1.4_AUG-EV-PASAV3_0030660.1 class=Sequence position=[PsnmuV1.4_scaffold_172-size_109320:92596..97202 (+ strand)](http://gbrowse255.tgac.ac.uk/cgi-bin/gb2/gbrowse/maplesod_psnmu_v1_4_gbrowse255?name=PsnmuV1.4_scaffold_172-size_109320:92596..97202) *SMC4*

AAATACAATCAAGCAGAAAGTAATTACAAGAGACTCTGCTTCACGTTGAAATCTGCTATTTCGTTGTTCTCTTCCGACAG

CACCGTGAATTGCAACTAGAGGCAGGAAGATTATAGCATAGCGACTATTGAACTTCTCGGAGAGCAACTTGCCCAATCTA

CGATAGTCGAGATGGAAGACGCCGATCCTCTTCGTGCTGAGAGGGACGGCGATGTTGATATGGGTACGAACAACGAAAAG

GAGGTAGAAGAACCTCCGCCTAGACTAATGATTACAAAAATGGTAAGCTGTTGGATCGTTGATTCCGTAGCCGAATAGAA

CTTGATGCGGAAATATTCAATATTTTTTTCTCATTTCTTGATCGTCCTCTACTTTGCAGACGCTGGAAAACTTCAAATCG

TATGCTGGAATAAAGGAGATTGGACCTTTTCACAAATGTTTTTCTGCTGTGGTGGGACCAAATGGCTCGGGAAAGTCGAA

TGTGATTGATGCTATGCTTTTTGTCTTCGGAAAACGTGCTAAGAAGCTTCGGCTAAACAAGGTGTCCGAGCTCATCCACA

AAAGTGACGGTGTCAAAGACAACCCTCCTCAGTTCGCGCGAGTTTCTGTTCACTTCCAGGACATTATTGATACGGGCAGT

GGTGATGAAGATTACCGTGTACTTCCTGATACCGAGGTAGTCGTTACCCGCATTGCCAAAAAGGACAACACATCAACCTA

CAAGTTAAACGGAAAGAATTCATCTTTTAAAGACATCGCGAAGTTTTTAGATTCGAAAGGAATCGATCTAGATAACAACC

GCTTCCTTATTCTACAGGGGGAAGTAGAAATGATCAGTATGATGCCACCAAAGGGRAAAACAGAATCTGATGAAGGTCTC

TTGGAGTACTTAGAGGATATCATTGGGTCTTCGAAATTCGTCGAGGCAACCAATGAAGCCGCAGAAAGAGTGGATGGTCT

CTCTGAAGTAAGACAAGAAAAATTGAATAGAGTCAAAGCAGTTGAACGGGAAAAGGAAAATCTAGAAGGGGCAAAACAAG

AGGCAGAGGCCTTGTTGGGGAAGGAACGAGAGATAAGGCGCAAACAAAATATTCTTTACCAAATTCATGCTATGCGAGCC

GACAAGGATTCGACAAAGTACACACAGCAGCAGGAAAAACTTTCGGAACAGCTAGAAATCGAACGTGAGAGAGTAGCTCA

AGCCCGAAAGCGAATCGAAGAAATTGAAAGTGGTCTGACAGAGCAGCGCAAAGACTACGATGCTTCTTACCAGGAACTGA

AGCGAACCAAAGACGAGTTTAGTGCATATGAGCGACGGGATATTAAGTTACGAGAAACCATTAAGTATGAACGGACAAAC

AAGAAGAAATTACAGGACAAGATAAAAGCGGAAGAAAAGAAGGAGACTAAGGCAGAAAATAAAAAAGCCAAGGCGGAAGA

AAGTGTTCCAGAATTAGAGGACGAAATCGAACGTCTCGCTGAGTCAAAAGCTGAAGAAGATTCTAAGCTGGAAAGAATCG

AAGAAGAAACAAAAGTTGTCACCCAGAATATTCGTCGAGAGCTAGAAGCAAAAGCAGCCGAGTTAGCACCTGTCAAACAA

GAAAGAGCTGTGTTGCAAGCTGACTTAGATACGGCCGAAACAGAACTCAAATTGTTGGAGGACTCTTCGAAACGAGTTAA

GGAAAGACTGGCTTCTTCCGAGTTAGAACTCGAAGCTCTCGATGCCACACAAGAAAGTAAACGCCAGAAGCTGAAAGAAC

AAGAAGTAGAATTAGCTTCGACTAAACAAAGAATCGTTGACTTAAAGCAAGAAGATCGTGAACTGTCTGCAAGAGAAGGA

AGTATGGGAACACGCCACAAGCAATTACTGGTATGTTACGAGCATAATCGCGAGATGGCAATCCGAAGCACTACGATTTC

TGTTCCTAATGCGTTTTCTTTCTCTTCTTTTTATTCATTGTCTTCATTCCATCTGATCATATCCAAAGGCCCAAGTCGAG

GATGCAAAGTCAGCTCTTAGAGCTTCAGGCGGAGGAAAGTCACGTTCCAAAGCAGTACAAGGAATTTTGGACGCTTCCAA

AAGAGGAGGCGAACTATCGAAAGTTGGAGTTTTGGGCCGTCTTGGTGACTTGGCCACCATCAACGAACAATATGATGTGG

CTGTTTCGACGGCGTGCGGTATGCTTGACAACGTTGTTGTGCAGACGACTGCTGGTGCTCAAAGATGCCTTGAGTTTCTT

CGAAAGTACAATCTCGGACGCGCCAGTTTCATTCCATTGGATAAGCAAAAGAAGGGTGCTCACGATCGTGTCGTCGAGAC

CCCTGAAAATGCACCTCGTTTGTTTGATCTCATCACACCTTCAAACTATGCCATCACACCGGCCCTCTATCTTGCTATAG

GAAACACTCTTGTAGCTCCTGATCTAGAGACTGCATCACGTTGGGCCTATGACTATTCTCGGCGATGGCGTGTGGTCACT

TTGGACGGAAAACTAATTGAAACTGCAGGGACAATGTCAGGAGGAGGAAGAAACGTCCGTCGKGGCGGGATGCGTCTTGG

GGTAAGTTTGCCAGCGCGTGATTATCCTCGCTCCGATTATCATTTGTAAATCAAACCAGCTCACACTTTTCGCATACCCT

CTCGTTCCGCAGAACTCTCGTCGTCCAGCCATCACGGTGGCACCCGGAGAAGACGAGGAAGAGATCATCGAAAACATGAG

TCATGAAGTCAACAATTTGGGTGCAAAGATTCAGGAGTCTCGTAAGCGACGACTAGCTATTAAAGACGAGCTACGAAAGT

TGACGAAAGCTGTGAAGCTGTTGGAAACAAGTTTGCCGAAATTGACGCTAGAAATTGAAGGGTGCGATACGACTAGAGAA

GAACTGACAAAACTTATTCCCGAACTTCGTGTGCAGAGCGAAGTGAGCGCTGAAGATTTGGAGAAGGCAAAAGAGCTTGA

AGCCAGAGTCGAAGAATGTAAATCGAATATGAARTCTTGCGTCGAACTTGCAGATCGGCTTGAAAAAGAAGTATCAAAAC

TACAGAAGAATATCCTTGACGCTGGAGGTCCTAGGTTGAAGAAGCAGAAGGCGAAATGTGGAAAGATCCTTTCAGAGCTT

GACGAAAGGGAGAAGGCTTTGAAGTCTGCCAAAGTTGGAATTGTTTCGTCCGAGAAGGCTTTGGCGAAAGCAAAAACAGC

TAAGGATGTTTTGTGTCGTCAGCTTGAAAAATGTGAGGAACTCCTGAATGAAAAAACGACAGAATTCAAGACTCTTGAAT

CCGATGCGTTGGCCGTGATCCAAGCGTATGATAAAGTGAAAGAAATTGAGGAGGAAAAAAGGACCTTACTGGAAACGGCC

ACGAAAGAGGCTGAAGATTTGAAGAAATCTGAATCTGAAGTCCAGTTGATTGAGATCGATTTGATAGGACAATTGGATGC

TCTGAAGAAACAGATTGCAGAATGCCAGAAAACTAAAGCTCATTGGGAAAAAGAGATCTCTCGTCTCAACAAAGTTGAGG

ATGATTTTGAAATTGATGATGCGAACGAAGATGGAGATATTGAACAAAGTGACAGTTCTGATGWTGACGACGACGACAAT

CATGCTTCGTCAGCTGATATCGAAATGACTGATGCAGAGGAAAGATCATCGGAGAAAAAGGCATTAACGAAATCTCCATC

ATCTACGTCTTTAGTGCTCCCTTATGAAGCACTCGAAAAATACGATCGCGAAGAACTGAAAGAAACCATTAGTGTTCTAC

AGAATGAGCGAAATGTTCTTGCAAAAAATGCAAACATGGGAGCAATCGCTGAATACCGTAAGAAGGAATCGGACTACCTT

GCGAGAATTGATGAGTTGGATCAAGTCACAGAGCAACGAAACGATGCAAGGAAAGAGCACGAAGAACTTCGTCGTCAACG

TCTCGAAATGTTCATGGATGGGTTTGGAATCATAACTCTGAAGCTAAAAGAAATGTATCAAATGATTACACTTGGTGGAG

ACGCGGAACTAGAATTGGTTGACTCGCTGGATCCGTTTTCCGAGGGTATTGTGTTCTCTGTGAGACCTCCGAAAAAGTCT

TGGAAGAACATCAGTAACTTGTCTGGAGGTGAAAAGACCCTTTCTTCGCTGTCTCTTGTATTCGCACTCCATCACTTCAA

GCCAACACCACTTTATGTCATGGATGAAATCGATGCTGCCCTGGATTTCAAAAATGTTTCTATTGTTGCGAATTATATTA

AGGAGCGGACAAAGAATGCGCAGTTTATTATTATTTCACTCCGAAATAACATGTTTGAATTAGCAGATCGCCTCGTAGGG

ATCTACAAGACGCAAAACGCAAGTAAAAGTATCGCAATCAACCCAAAATGCTTCGTATCTGGACATAAAACACGCAGCTC

TACTACATCATCAACAGTTCCGCTTGCTGATGCATCAAATAATTTTCCATCTATTCATTCTTCGTCAACTTACAAGAGAA

CAAACCAAATCGATCACTGAAAAATCAGCGTTGGCGGGATAAAATGTTGGATACTTTAAGAAAAGAATTTGAAGTTGGGG

GACGGTTTCTTATTTTTGTTCAGGTACAATAATAAATCATCGAGTTG

>PSNMU-V1.4_AUG-EV-PASAV3_0102810.1 class=Sequence position=[PsnmuV1.4_scaffold_6-size_405735:157906..162340 (+ strand)](http://gbrowse255.tgac.ac.uk/cgi-bin/gb2/gbrowse/maplesod_psnmu_v1_4_gbrowse255?name=PsnmuV1.4_scaffold_6-size_405735:157906..162340) #*SMC5*

CACCGCAAGACCACCCCGGAAACCCGAACGAGACCTGCCAGATTGCACTCCGTTGTTGGTTGCAAAACACCTTAAGAGCA

GACACACGACACCGCTTGCCCGCCAAAACAACGCAACGGAACGCAGCGCAGCGACGCCTTTGCCCCGCACCGATCGATCG

ACCGCAGCACAGAACAGAACAGAAGAGAACAGCGCGGCACAGCATGCCATCGTCGCCCCGGTCGGACCTGGAGCAGATGG

AAGAACAGGCCGCCCTGGCGCGGCGCCTGAAGACGGTCGGCGACTACAAGCACGGTTCCATCAAGCGGATCAAGCTCCGG

AAGTTCCTGACCTACAGCGCGGCGGAGTTCAGTCCCGGGCCCCGGTGCGTTTCTGTAGTTTGTGGTTGTTGTCGTTGTTG

TGGTTGTGGTTGTTGTCGTTGTCGATGGAGTGGCCGGAGGATTTTTGGGGTTGGTCCGAGGCTTTCCAGGCGCTCCCTAA

CGATTTTCGCATCTGTCCTCACACTTTTTTTCCCTGGTTTGGTCGCAACTCAATTCCTTCCCTGGTTTCTGTTTCTGTTT

GCGTTTCGTTTCGTTTCGGATCGGTTCGTTTCTGTGTTTTTCGCAGCCTAAACATGGTGGTGGGGCCCAACGGAACGGGA

AAGTCCTCGATTCTCTGCGCCATTTGCCTCGGACTGGGGGGTGAGCCGCGGCTGCTGGGAAGGGCCGACCAGGTGACCTC

GTTCATCCAGAACGGGGAAACCGAGGCCCGGATCGAGCTGGAGGTGGTGAACGAGCACGGGGAAAACGTGATCGTCACCC

GCACCATTCACGGAAGCGGGGACGGAAGCAGCGGCAGCAACCGGAGGGCCAACAGCAACAAATCGTCCACCTTTACCTGG

AACGGCGAGATCGTTTCGGGGAAGAAGGTCCAGGAGCGGGCCTCGAAGGACTTCCAGATCCAGCTCGACAACCTCTGCAC

CTTTCTGCCGCAGGAAAAGGTGGGAAACTTTTCGGGAATCAACAGCAAGGACCTCCTCCTCGAGACGGAGAAGACCCTCA

GCGACAACAAGGATTTGTACGAGAAGCACCTCAAGCTCATCGAGATGCAGACGGAGCTCCGGGGGGGATTCAGCAAGGTC

GACAACCTCAACGAAAAGGCGGCCTTTCTCGAGGCCGAGGTCGCCAAGTACAAGGCCGACGTCGATCGCATGGAAGAGCG

GAGGAAAGCCGAGGAGCAAGCGGACCTTCTCCGGAAAAAACTCATGTGGCTCAAGCTGGACGGCGTCCGCGAGACGTGCC

TGGCACTCAAGGCACAGAAAGAAGAGGCGAAACAGAAGGTGGAAAACCTCGAGGACGAGCTGGAGCCACTCCTGCAAGCC

AGGGACCAGGCGAAGGAGTGGCTGGAGAAAGCCAGGAACGACGTCGACGCCTTTGAGAAAAAGATCAAGAACGAGGAAAA

GAACATGGCGAAGCAGAAGCACAAATTCGACAACCACGACGACCAGATCGAGGAGACCCTGGCCGAGCTGGCGACCGTGG

ACAACACCCGCGCCAAGTACGAGAACGACGCGCGGCTGCTGCGGGACAAGGTCGGGGGCCTCCAGGAGGCGCTGGACGAG

GAGCCGCCGATGGAGGACCTCGAGGAGGCCTTTGCGAGCGCCCGCCGGGAGCAGGATGCCATCAAGCCGCGGTACAACGA

CTCGAAGACCGCCCTGCTGGAGCTCAACCACGAGATGTCCTCCGTGAAGGACGAGAGGAACAACGTCCAGAGAAAACTGG

CCAGGCTCCAGAACGAGAAGGAACAGCGCCGCGAAAACGTCTTTCGGTACTTTGAGGACGTGCGGAACGCCTACCACTGG

ATCAACAACAACCGGGGCCTCTTTCGAAAAGAAGTCATTGGGCCCGTGGCCTGCGAGATTTCTCCCAAGTCCAACGACAG

CGCGGCCTTTCTCGAACAGCACGTGGCAAACTCCGTCCTCAAATCCTTTGTGGTACAAGACAAGTCGGACTACGACCTCT

TGTACAACAAGGTCCGTCGCGAACAGAACATCCCCATCAACATCAACTTTGTGGACCGGATTTCCCAGGGCGAACCCCGC

ATGTTTTCCGAACAGAAAATGGCCGTCCTCAAGCGAGACCACGGCGTGATGGGCTACCTCGACGAATCCTTCGATGCACC

GGACATTGTGGTCCAGGTGCTGAAAACCAATTCCGCGATCCACAAGGTGCTGGTGGGAAGCGACCGAACGCAGGACAGCC

TCGACGGCCGGGACCTGGGACGGATCCTCTCCGAGTCCGAACACCACCACGGCAAGCTACAGTCGTACTGCATTTTCGCC

AGCAAAGACGGCGATTCGTTCAAGTACACCTCCCAGGTCTCGAGGTATTCCGGGAAACCCTCGCTGCGGTAAGTCTTCTC

GGATTTCTGTGTGGTTGCAAGCCGCGATGCTCCAAGGCTAAGGCTTGCGACCGCAGCGGTCTCGTGTTTGGAATACTCAA

TGCATCGTTGTTGCTGTTTGTTTTTGTGTTCTTGCGGTACCACAGAGTGGACGACGTTCGGGTCGCGAGGTTTTTGAGCC

GCGGGGCAAGCGACGACGCAAAGCAGCGAGTGACCCAGAAACTGAGGGAAGTAAAGGACCGCGAGAACGAAATCCAGCCA

AAAATCGAACAGTGTGCCCGCGAGCAGGAGGATCTTTTGCTCCAGGTCCAGCAAGCGCAGCAGCGTTCCAAGGAAATCAA

GGCCAAGATCCAGACGATCAAGAAACTAGTACAAAAACTCCAGAATTCCAAGCGCAAGCTGCGGGAAGCCGAGGAAAGGC

TCGAAACCGACGACGAAGAAGAAAAGAGGAATCTGGTAAGGAAACTGAAGCAGCGACTCGAGGCCTCGTTGAAAGCGATG

AATGCCTATTCGGAAAGCAACAAGAAAATGGTAGAATTGACGGTAGAGGTAGCGGGGTTCAAAGTAGACAAGGAATTCGC

GTTGGTCCGAGCCAGACTCAGCGAGTAAGTATCAACCCAAAGCATTCTTTCCAGGGATGCGATGCGATGCGATGCGATGC

CCTAATTTGCCTTTTGAAACGTTTCCAACACGTGCTGCTTTCCCTTTCTGTTTCCTTAGAGAAAAAGCGGGAGATGCCGA

AACCGCAATCGATACGATGAGAGACGAATTTCGCAAGGCAAAGCAACAGTTCGCGCAAGAAAAGAGTCGATACAAGGAGC

TTGCGCAACGCGCATCGGAACAAGCCCCTCTTGCGGACGAGAACGGGGAAGAAACAGATCTCAAACGAAAGCTAGAGGTG

GACCTTGCCCAATTCGACCGAGAAGATCTCGCGCAGAACGCGCTCGAAGAAGCCGAAGCCAAGATCAACAGCATCCAGCG

AGACGATAGTGCGCTGCGGGTCTACGAAGAAAAGACGAGAGAGCTCGAGGAAGTCAGAGAAAGCCTCGATGATCTGAAGG

ACCGAAAGGAGAGAGGCCAATCGGAACTGGAGCGCATGAGAGTTCCGTGGCACAGCACGCTGAAAGAAATCATCGAGACG

GTGGACAAGCGGTTTACGAAATACATGAGCGAGCTCGGATGCATCGGAAGCGTTTCGCTCAAGGACGGAGACAACGACGA

GGACTTTCTCTTTGAGGATTACGCCGTCGAGATCAAGGTCAGCTTTCGAGAGGGCGTCAAACCGACGGTGCTTTCCAGTC

GGGTCCAATCCGGGGGCGAGCGGTCGGTGTCTACTATTATGTACCTGATGGCCATGCAGGACATGATGGTTTCCCCCTTT

CGGTGTGTCGACGAGATCAACCAGGGCCTGGACGACCGAAACGAGCGCTTGGTGTTCAAGCGCATCGTGGAAAATTCCAC

GCAAGCCCCAGGCCCGAACGGACCAACGGACCACTGTGGCCAGTACTGGCTGATCACGCCCAAGCTCCTTCCCAATCTCA

CCGACATGGAGGTTGCTGCAATGAACGTCGTTTGTATCTTCAACGGTGAGTGGAACAAAACAAAACGGTTTTCCCTTCTT

GCTTTGCGTTTCCGCAACTTTTTCGAACTCCTAACCGTTGTCATCTTCTTCCTTCCAAAAGGCGCTTACAACTTCAAGAA

TCCCACGGACTGGTGCACGAGGGAACTGATTGCCATTCGAAAGCGTCGCCACGAAGAAATTGGTGACCACGACATTGGCG

ACGACGATGACGACGACGACGATAACACTAGAAACAAGATGTCTAAAACTGGGGAATAAAACGCTGCAGCAGTCTCATCA

GAATTTGCGGGAATAGGCATTGCGGGGAGGACGGCTGGCGACTTTTTTGGGGATTGCAAACCCACGGCCGATGATGATGC

TACGCAATATCTGCTGCGATTCCATTGACTCGCGACGGAAGAAGCACAGTCATCGTTGATCCATAACAACAACATTACTT

CTAGTATAATAGTCGCAGCCTATATCCTGAAAGCA

>PSNMU-V1.4_AUG-EV-PASAV3_0090050.1 class=Sequence position=[PsnmuV1.4_scaffold_47-size_226086:163759..168095 (- strand)](http://gbrowse255.tgac.ac.uk/cgi-bin/gb2/gbrowse/maplesod_psnmu_v1_4_gbrowse255?name=PsnmuV1.4_scaffold_47-size_226086:163759..168095) *SMC6*

TTCTCTGTTTACTGTACGTCGCGCAAAAAAAMGTATCGCATTGCACCAAGTTACGAGCCGCAACGTAGAAATGAGGTTAC

CGGTCGAAGCAACAACGAAGTTTTTTTGGGGTCAATCATGCTGTACCGTACTTCTACTACCTTACCGGCGGAACAAGACA

TCGACGGAAAACAASACCTTCGTGYTGAACACTGCTCGTCTTTCTTCATCATTACCATCCTTCCGACGCAATATATCTCA

AGGAGCAGCTTCATTTCGCTGCTTTTGGCACAGCTTGGTATCTTTTCTAGGTATWAATATTTTAACTGGGCAATTCCTCC

GTYCCTRTAAAACTTCGTGCCAACGATGGCCGCCATGGACAATCTTTCCGTAGAACCGAACGCATACGTCGAAGCGGAGT

CGACCGAACACGTCGACGAGATCGAAAACGAAGACGAAKCGTCGGTGTCCCCCCGCAAACGGGGATCTGGGTTGAAGTCT

TCCTCCGAAAATCCCCGGCGGAAAAAACAAAAGGCCGGCAAGCCACTGTCCCATGAAAGYAGCGAAAACGACACGACCCA

AGACAAGGAAAATAACGGGCGCCGTACCTCTTCTGCCTCCCGCCGCAGAACGACSAACGARTCTTCGACCGATGCCACTG

ATTTTCTTGCAGGCCGCAACATAAACAAACCGAACAAACCTCCCGAGGCGGGAGTAATTACAAGGATTTACGCCGAAAAC

TTYATGTGGTAAGTAACAACAAAACGGACCAGGATGGAATGGAATGGAAGAGGGAACGAAGCGTATGGAACGGGGATGGC

CAACTAATTTGCGAKATGCCTTCAAGGTTCCATATTCRAACAKCTTACCCCATCGCTCGATCTTAATTCCATTGCAATRA

ATACAAAGCCATCGCAAATTGACCGTAGACCTGAACCGAAACGTCAACTTCATCTACGGCCAAAATGGATCGGGAAAGTC

GGCCATCTTGGCAGCYATTCAGATATGCCTGGGCGCCGGAGCCCGTCGTACACAYCGAGCACGMAATCTGAAGGATTTAG

TTCGCAAGGATACTTCMTCMAATTGCGCMAAGATTCGTGTCACCTTGCTCAACAAGGGCGATGATGCGTTTCAACACGAA

AGATATGGGGACCACATTACCGTAGAAAGGACAATTGCACTTCGGGGAGGCTACAATGGATATAAGTTGTACAGTGCGAA

AAAYATTGAGCAGTCGAGGTCGAAGAAGGACCTCGATGAGATGCTGGACAAGCTCAATATCCAGGTTGAGAATCCGGTGG

CCATTTTGGATCAGGAAGAAGCCAAGAAGTTTTTGACCGGSAAAGCCAAAGACAAATACAAATTCTTCGTAAAGGCAACC

GAACTGGAGCGAATCGATAACACCTTCCGAAACACGAAGGAACAGCTAATGGATATGGAAGTCAAATCGGATAGACTTSA

GGAGTCACTCGATCGAGACMGYGAACTGGTCSAGGAGACCAAGAAGGCATACAAACAGCATCAGGCTATCGGGAAACTAG

AGGGGAAATATTCGAAGCTCCAGATCAATATGGGGTGGGCAAACTACAAAMTTGTCACTGAAGACGAGCATACAAAAACA

TTCGTACGTATTGCTTCATTTGTGTYAACACAATTCTGAATTTTCTTCATGTTTTCATCTCATCATTGTATCTTTCTGTT

GCTTTRAATAACCGTMACAGAAATTTGAAAAATTTAAGGAAAAAGCCGAGAAGAAAAGACAAGAATTGACGCAAGCTGAG

GCTGTTACCCAGGAGGCCGACGACCCAAACGATGAGCGTCGCAACCGTCTAGATACSCTCACGGCCGAGGCAGATACCAT

GGCGACTCTGAAACGGGATTTGGAACAGCAACTCAAAAAAGCAACCGAACCCCAGAAGGCATTGGCTAGACAATTTAAGC

TCCTGAAAAAGGAGGAAGAAAGTATTAACAGAGCTTTGTTAGAGGCGAATCAAGCCCTTCAAGCCAAGCGTGACGAGATT

GTGGCGAAGGCTGGATCGGCCGAGTCGGAACAAGCACGCAGAAATCAACGAMTGCAAGCTGCCGAGAAGAAGATTGCAGA

GGAAAAAAYTCGCCGCAACGAAATCAAACAGGCTGTGACGGATGCGTTGAACTCCTACGAAGATTTGGAACCGGAAGTAA

TGGGAGCAAAACAAATTGTCTCGCAACTMGAAAATCAACTGAGAGGAATCGAAGGCAAAATTCGATCGATGGAATCCTCT

TCGGGAAATTCCCTCGARATATTTGGCAGGAACTGTGGCATAGTGAAACAAATGGTAGACAAAGCAACTCAACAGGGTAG

ATTCAAGGCACCCGTTATTGGACCAATAGGCTTTTACTGCAAAATCCAACCGGGAAAAGAAGAGTTCGCCGCGTTGGCAG

AACAAGCCATCGGGAATGGTGTATTGGATAGATTCATTGTGTTCAACGATGCCGACCGAAAGACCTTTCAGAGTATACGT

AACCGAGCTAGGTGTAAGATGGACTGTGGGATTTTCCAGCAGTCCCAGCATGCTCGATACAATATTCCGGCACCGCCAAA

GGGTGTCGAGACAGTCGCAACCGTTGTTTCTATTCAGAATGATTCGGTGTTCAATTGCCTCATCGACTCGGCTAAAATTG

AAACGAAAGCCCTGTCTCGTGATAAGAAAGAGAGTGAAGACTTGCTACTTGTCAAAGACAACAACAATCGCAATGCCATT

CGGGGAGGAAATATCAAAGAAGTCTACTTTCTTCCGAATGGAGATAATTGGAAAGTCACCAAAGGTATTTTGCATATGAC

TTCCAATACCCGCAGATTGAAGAAGACAATTGGTGTCGACATGACTGCGGCCGTTGAAGATGCAAGAAACGAGTTCCAAA

GCATCAAGGAAAACTTGAGGCAAAAGAATCTCGATTTCAATAGGTTGAACCAAAAGCACACAGACTATAAAAAGCAGTGG

AACATTAAGAGGCGAGAATTAAGAAACAATGAACGTGAGATTGATCAAGCAACGAAGGAAATCGATGAGATTAAAGCAGA

GGAAGCAGCTTTTGTCGACAACGACATCGACACTTCGGAGGAGGAGGAGGAAGTATCCACAGCCCAAGCCCATCTAGAAA

AGGTCAAGGAGAATCAAGAGAAAGCGCAGGACTTGATGAAAGAAAAAGTTCCAGAGATTCAACAGATCAAAGATAAGCTT

CATGAAATTACAAACCGAAATGAAATGATTTTGAATGATTTAGATGAAGCACAACAAGCTTTAAGTCAACACTATCAAGC

GATAGAAATTCAAAAGGAAAAATTAGAGAAGAAACGGCAAAAACTTGAGCAATACGAAGAGCTTGTTTCCCAGCATACAG

AAATAGTCAGAGATGCGCAGGAGGTATCAATTAAGTTCTTGGATATGGCAAGGAAGGTCCAATATGTCAACGACCAGGCG

GAACGACGCCGAAAGCAGAGAGAAGACGGAGAAAATGGGGATCAAATGGGTGAAACGCAGATGTCGGTGTACAATGAAGA

ACCAACAGATGAAGATCTTGCGATGATAGAGATTCCACAGAATCTTGATGGTTTGAAACATCCTGACTACTACAAAGGCA

GAATGGAACAAGTTGCAGCGAGAATCGAAGCTGAGAGGCAACGACGTCTTAAAAATGGGGACGACGAAGCAACTGCCTAT

GCCAAATATACTCGTGCTCTGTCCATTTTTCAAGCGAAATCACATCAAATCGAAAAAATCGAAGATACAACCAGCAAATT

GAAGCAAGATATGGAACAAAGAGAGAGACGTTGGGGACACTTTCGTGATCACATCAGTGATTTCACGGGCATGAAATTTA

ACGAAACTCGTAAGTATTATGGTCTGCATCCATCTTGGTGAAAATTTCGGCACCAAATCGTCGGCAATTCTCATCTCAAT

CTTACCATCTGATCTTTTGTTTTAAAAAAATAGTCCATATCAAAGGCTCCGCCGGAACGATCGAATTCGATCACCACAAA

GAAGAACTAAATTTGAGTGTTTACAAGGATTGTAACGATAACGGGACTGAGCAAAAAGACGTGAAGGCTCTCAGTGGTGG

GGAAAGGTCATTCACGTAAGTTGTCATATCGCAATCAAAATGACTTGTATTTGGTATGAAACTTTTTTGTCCTGTACTCT

TGCGCTTGCAACGAAGTGGATCCTGAATGTTTTCCTCTTTCTTGGATTGTTTGTCTTGTGTAGGACCATTGCCCTTCTAT

TGGCCTTGGGAGAATCATTGGAAACTCCTTTCCGTGTGATGGATGAGTTCGATGTGTTTCTCGATCCGAACGCAAGAAAT

AAAGTCATTGACTTACT

>PSNMU-V1.4_AUG-EV-PASAV3_0089060.1 class=Sequence position=[PsnmuV1.4_scaffold_462-size_41819:12317..19035 (- strand)](http://gbrowse255.tgac.ac.uk/cgi-bin/gb2/gbrowse/maplesod_psnmu_v1_4_gbrowse255?name=PsnmuV1.4_scaffold_462-size_41819:12317..19035)

GAGGCACCGGCTGCGGCAGCCCCTGGAGGAGAACAAAGAGCGGTCCGGGGGAAGAACGCGTTCCCACGCTGGGTCGCAAC

CACCACACCGCAGAGCGCCATGCCGCCACCGTCCCCCCGGGGGGCCTCCGCTTCCGCATCCGTTGCCGCCACCCATGCCC

CCGCGATCGGGCACTCGGGGCGGATCCTGACGGCAAAATACCTCCGCAAGGCCTCGAGCAAGGTGCGTTGCGTTGTGTCT

TTTTTTCTGCCTGGCGTTTTTCTCGTTTTGTTCTGTGTTGCTTTGTGTTGCTCTTGTATGTGTTGTTCTGTGTTGCTCTT

GTATGTGTTGCTCTGCCCCGTTCCGTTCTCACAAAACAAAAACCTGTTCGTTTTCACGGTTGGCGTTGCTTCTGAACATC

CCTTGCGGCGATGGAACAAAACGAAACGAAACGAAATGGATGCGCATCCAACACCACGCGACAACTACAATCAATGCACC

ACATCACATAACCCGCAACGCATCGCATCGCATCGCATTGCATCGGACCAACCACGAACGGCCGATTGCGTCCCGCTTTC

GGCAACGAAACGCGATGCATTTGCAAACACTACACTACACTACACCACTCCACACTACACTACACGCCACACCACGCCAC

ACCACCGTCCCTCGGCTTCCGCTCCGCTCCGCGTGCACGCCCACACAGAAGGACCTCGTGGCGAGGCTCCGGAGGGTCCT

GGGGTGCCTCCGGGACGACCCGCACCTCGAGGCCGAGGACACCTCCGGCTACCCCGGCCTGGCCGGCCTCTGCGGGGCCC

TCACCGGGAGCGGGGGCGACCCGCAGAACCGTGGCCGGAGGGCCGGCCCGATCCACCACAGAGACAAGGAGGTCCGCCTC

TACGCGGTGGCCGCCTGCATGGAGCTCTTTGCCCTCTACGCCCCGGCCGCCCCCTGGAACGAGGCCGAGACCCTGGACAT

TTTCGGCCAGACCATCCGCCAGCTGGCCAACCTGGGGCACACGCACACGCACACACACACCGAAACCACCAAGGGAGCGC

ACTTCTACGAGTACTACCGCATCCTGGAACTCCTGGCGGAGGTCAAGATCGCCGTCCTGCTGGTCGATCTCTCCAAGAAA

AACGACGACGACAACGACAACAGCGGCGGCAACAGCAGCAGCCATAGCACCACCAACCACAACGCCGCCCGCCCTTCCGG

GAACCAGACCAACCGGGAGGCCCTCCTGGTCCTGACGGAGCTCTTCCGGACCCTCCTGCAGTCCGTCCGCAACGGGCACC

CCCCCGAGGTCCTGGACTGCTGCCGAAAGACCCTGACCTCCTGCATCGAGGAATTCTTCGAGACCACCTTCCTCCCGGTC

CCCCTCCTCGACGAGCTCCTGGTCTGCATCGGACAGGGCCCGCGGGTCCTGGTCCTCAAGCAGCAGCAGCAGGAACAGAA

ACAGAAACAGAAACAGGAGCCGCCGCGCGGGAAACCCCGGGCCGGGTCCGGGGCTTCCGGGCGGCAGCCCCTTCCGGCCC

CGGTCGTCACCGTCCGGCAGAACAACCCTTCCTACGCGGTCGCCTCGGCGGTCGTCCGCGCCAGCGTCGAGCGCCTGTCG

ACCCCCATCGCGAGCCTCCTCAACGGCCTGGTCCAATCCGATCCGCGGGCCGTAGGGGCCTCGACGATCTCGAACCACGC

CTGCGAGGCAAGCGCCGATTCCAACGCCGGCGCCGGAAACGAGCCCCCCGTCCCGCGGGGGGTCCTGGAAATGGCCGACG

CCCTGGGCCGGCCCCAGAGGCAGACCCAGGACCCCCACGGGTCCAGCAACGTCTACGGCGTCATCCTGGAGCTCCAGAGG

GTCGCCCCGGCGATCCTTACCACCGTCTTTGGAAACCTGGCGAGCCACGTCGAAACCACCGACGTCGGCCAGAGGCTCCT

GGTCGTCGAGACCCTGGGCAGGCTCTTTGCGGGCAGCGGAAGCGGCGACGCCGCCGGTGGGACCGGCCGCTCCGGCCTTT

CCGTTGCCGCCAAGAACAGGGAGAGCTTTTTCCAGTGGCTCCAGCGCAGCGGGGACAGGCGCATCGAGATCCGCCGCCGG

ATGCTCCCCCACCTGCTGGCCCTGACCCGGGCCGGGAGCTCCTTTCTGGAAGAAGCCGGGGCCTCGTCGCTGGAGGCGAG

CCTGGCGCTCAGGGTCCAGGAGGCCCTCCTCCGGAGGCTTTCGGGGGATCCCTCCCCGGCGTTCCGGACCGAGGTGGTCC

AGGGCCTCTGCACCCTTTCCTACAACCACCGCAAGATACTGACCCGGAGGGCCATGGACCAGCTCGGGGAGCGGGTCATG

TCCAGGGACCGGGCGGAGCGGAAGGACGCCCTCACGGGCCTCGTCCAGCTCCACTTTCGGCAGTACACCCTGCACCACCT

GGCGGCGGTCCTGGAGGGGGGGGACGACTGTCCCATCGGGTCGGTCCTGGACGTTCTGCGCAGGTGCTCCTTCCCGGGAG

ACCGGGCTTCCGCCGCAGGGGCGTTCTCCGCGGAGACCGCCGCGAGCCTGATCGCCGCGAGGTCCCGAAACGCGGCCCCC

GAGTCCTGCGGGAGGCGGCGCGGGCCGGAGGAGACCGACGACGACGACGAGCCCGGGCCTCTGGAGTCCCCTGGATTCTC

CGACCGGGGCGGCAGGGACGACTTTTCCTATTACCAGTGGATCCCCTGCGTCCTCTTTGAGAGCGCCAGCTACTCGGACG

CCACCGACGCCGACATGCACTCGCGTGTCGTACAGCTGGTCGACGAGCTCCTCCTGGGGTGTTCCTCCCCCCATCCGGAC

AACCGGCGCAGGCTGACCTCCACCGGGAGGGCCACCGCGATGGCGATCGTCGTAGACGCCGTCCGCAAGCAGTCCCATCT

GGCCGGCCTCTGGATGACCAAGCTGCAGTCCGTCCGGGCCAGGCTCCAGAACGCTCTGAAGGCCTACCTCGAAGCGAGGG

CGGAGATCCGGAGGCACGAAACAGGTACGTCGACGGAAACGGAGCGGAAGGCAAAAAACGTGGCGCGAGGCGTCCGTGGA

AGCGCCGCCGTGGCCTTCCGGGAGAAAACGGAGAGCGCGGCATTCCGATCCAAACCACAATCGCTGCCGTCCTTTCTTTC

TCTGACCAATTCTCTGCTTCCTTTGCGTTTCAATGTGTGTACGACAGGTTCCGAGGAGTATTTTGCTGCGGACGCGAAAG

CCAAAGATCTATTGGAAACGGTAGCTTCGATGATCCCCCCGCCAAGCGGGGCTTCTCCTGCTCCCGGAGAACGGCACGCC

GTCCTGGAAAAATTTCATTCCAGCAAAGACAAGCGCGTCTTTCTCGTCTTGGGCACGATCACGAACCCGAGCCATTCTTC

AAAGTGTCGTGCCAAGGCCATCGACGACTTGCCGAAGCGGGTGAAGGCAACGGCCGGGGATGCCGTGTCTGCATGGGTCA

AATCGCTGGCAAAACGTTGCGCCATGGGAGATTTCGTAAACCTCGATGTCGTTCATCACTGTGTGCTGCTGGCCCAAGAA

TGTTTTCACGAAGGAGACTTGGATGCCACCCTCAAATTTTTGGTATGCGTTCAAATGGCGGTAGAATCCTTTCCTTCCCT

CTGCGCATCCGGGGAAGTCTTTGAGAACCTGAGCGAGTTGTTCCAGGATTGCAACGGTTCTTCTCAGAAAAATCAAACGG

AAGGACCCGCGATTGTTACCGCACTCAGCGCCATTCTTGCCTCCGTAGCGCCGTATAGAGACCCAAGCGACGATTCGTCC

TTGTTGGAGGATGATCTCTACAAAAAGCTTGTGAACCTATGTCGAAATGGCACCCCGGAACAAGCTCGGCATGCGGTGGC

AACCATTACATCGCTCCTCAAGCCGAAGAATGGTGCGGAACTCACCCAGGAAGAAACCAAAACTTTTCTTCCATTGCTCG

AAACTCTTGCCACTCCCTCGCGATTGGCCATAGCGTCGACTGGATCTTCCACGAAGCTGGTGTGTGTGTTGGCTGCTCTA

ACGGAGCTGGCAAACAATGCCCCCCAGGTGTTTGAATCTTCGAGTCGTGGAACGAAGGCGCTGAAGTTTGCCCTCGAAAT

GGTGTTGATGGGCCGCGCCCGTGTCGACGTAACAAGTGGTGACGACGACGACGATTACGATGAAGCGGACGATACAAGAA

CTCCCAAGCGTGGTCGAAATCGAAAGTCATCAAGCGCCAAGAGCAGGCATCTCAGCCCCAAGGCTACAGATACGAGTCTA

GTCGAAGACCAGAATCTATCGATTCCTTGCCGCACGATCTGCGCTGCAATTGAACTCTTGTCTGCGTTTATCCGATCTAG

CGTTTTCGTTGCCAAGAAATCTCGTTCGACCTTTACTCAAACGACTCAGGACATCATCGAACAATCGTTCAAGATTTTCT

CGCAAATATTGCGAGACCAAGGTTTGCCTCCATCATCAAGAGACCGCGACGTGTGTAGTTTTCGTCAAGATCGAGCCGCT

CTGAGGCAATGTGCGGCGATCCATCTCTTTCGTCTATGCGATACCCGCCTAGGACTCGATCAGAAACACCTTACAACGGA

GCGATGGCACACTTTGGCTTCGAGCTTACTCGACGACGAACCTGTTGTAAGAAAAGCGGCTATGGAAGAGTTTGGACTGA

TGATCACGGGCCATGGGAAGTTTTCTACGGCATACGGAATGGGCGTTATGGCACCCCGATTGCGCTTCGTTGCAATGTCG

GTGTTTTGTATCGATGGAAGTCAAGGCTCGCATTCCAAGGCGAATGGCAATTCAGCGAACATAGGAAAAGCGATTCACAA

TCAGAAAGGGAACATCGCTGGTTGTATTACTGACCTGAGGAAAGTATACGAATCATACGCCGTACAGTGCCGGGCGCAGG

GGCCAGAAGCTGAGAAACAGTTTGAGACATTTACCAAGCTTACCATCATGCCAGAATATGCTGTGCCCTATTCATTTCAT

CTTCTGACTTGTCGACAAGAAACACCCTCGTCGACTGGACCAAGCACCGGGAAATCTAGGAAGGCGAACGACGATGATGA

GAGTGGACAAAGAATCCTTCGAAAACGCTTGAAAGCTCTGTACGATCCACTCGTTCTCCAACTCGGTACATCGGCCGATA

ACATCAGCTTTCTTCTCCGCATGGCAGAAATGCTAGCAAAATCATTCCAACCTATAGGTTGCTCGCTTAGCTCCCCAGGA

AGTGACAACAGCTCAAGGGACGGGGATAAATTGAAAAATATATGTGCGACGGCACGAGAGGTATTGTTGTCGTACGTGAA

GACAGACGCGAATCTTGACACGCATCCCGGTGCGATACGGATGCCTGGCAATCTCTTCCGCAAACGACAAAGTCGCAAGA

GACCAGTACAGGAAGACTCCATCACGACAGATACATCTCCAATCATTGACATGATGATTACTACGCAAGACAGTGAGATT

CAAAACCAAAAAATAAGTTCAAACCTGAATCGAAAAAGACCAGCTAGCTCTAAGGAACACGAGAAGCACACCCAGGATCA

TCCTTTTGGCAACAATAATACCAAGCAAGAGCAAACCCGAAGATCTACAAGATCCGCTACAAAGCACGATGTCGATGGTT

CAAAAACGACTGCAAAAACTGATGAAGCGTCTAGAAACTCCCATGACACTCAAGCAAGCATCGCAGATACGAGTCGGAGG

CGAAGATCTCTTCGAATATCGACAAGAACAGCATCCATTGGTTCAACTAATACTTACGGAGATGGTGACTCGAGAGTGTC

ATCTAGCACAGACGAAATATCTGGATATTCAGTTGACGGAAATGTAGGTATTACAAGAAAGAATACCCAACAAATTGCGC

CTCGAAGATCCACAAGGTCAACTAGATCGACTAAATTCGACGATTCGAGGGACTCAAGCACATCAGGTAGCATCAGTATT

GAACATGGAAATGAATCAACCGAGAATGGAACAGGAACTATTGAATCAACCCCGTCGTCGAAGGATGTCGAGAGCACAGT

TACCTCTACTCGTGGTATTATGCTTTCGACTGAAGGTGAAGACGACCCTTCGAGTTCTCGACTTGGAAGCGGCTCCAATA

AAAAGCGACGATCAGATGCTTCCAAAAGTATCTCCGCCGACAGTCGCGTACACTTTTCTCCTGAAATTGATTTTGGAGGG

TTGTCTCCCATCAACAGGAGGAGTTCCCGTAACAGTAAAAATGAGGAGCTGTTACTGAGTTCGTCGGAAACAAAAACAAG

AGGCACAACACCTCCATCGTCCCTCCGGAACGCCTCGTTCCCAGCTACTGCATCTGCGTCAGTTGCTATTGGGTCGCCTC

GGCCCCATGAAAATAGAGACTCCAAAAGCCCAACAACTGTGAGTGAATCCACTGCCAAAAGCTCAATTGGACGTGTTTCA

TCTCAAGGAAATGAAAAGACGAAGCCTCTAGGGGACAAAAAGGCAGAAGTTGCGCATAATCGACGAAGAAAATCATCTCG

GAAACTTGCTGTCGACGACAAGGAAAATGCGACTGCAATTAAGAAAAAAGGAGGCCCGAAAGATATCAAGATTGTCCGAT

CGAAACAAAGCTCAAAGTTCAAAGCCAAGAAAGTTGTCAAGAAAACGGCGCTCGCCAGCGTTGGAAAGCGCGGCAAAAGG

AAAAGTTCGAAACCAATTGATTCCTTTGACTTTGAGGGGTAAAATACTGGATGAGAAATAGTCGATTACATTGCACTGG

>PSNMU-V1.4_AUG-EV-PASAV3_0089060.1 class=Sequence position=[PsnmuV1.4_scaffold_462-size_41819:12317..19035 (- strand)](http://gbrowse255.tgac.ac.uk/cgi-bin/gb2/gbrowse/maplesod_psnmu_v1_4_gbrowse255?name=PsnmuV1.4_scaffold_462-size_41819:12317..19035) *PDS5*

GAGGCACCGGCTGCGGCAGCCCCTGGAGGAGAACAAAGAGCGGTCCGGGGGAAGAACGCGTTCCCACGCTGGGTCGCAAC

CACCACACCGCAGAGCGCCATGCCGCCACCGTCCCCCCGGGGGGCCTCCGCTTCCGCATCCGTTGCCGCCACCCATGCCC

CCGCGATCGGGCACTCGGGGCGGATCCTGACGGCAAAATACCTCCGCAAGGCCTCGAGCAAGGTGCGTTGCGTTGTGTCT

TTTTTTCTGCCTGGCGTTTTTCTCGTTTTGTTCTGTGTTGCTTTGTGTTGCTCTTGTATGTGTTGTTCTGTGTTGCTCTT

GTATGTGTTGCTCTGCCCCGTTCCGTTCTCACAAAACAAAAACCTGTTCGTTTTCACGGTTGGCGTTGCTTCTGAACATC

CCTTGCGGCGATGGAACAAAACGAAACGAAACGAAATGGATGCGCATCCAACACCACGCGACAACTACAATCAATGCACC

ACATCACATAACCCGCAACGCATCGCATCGCATCGCATTGCATCGGACCAACCACGAACGGCCGATTGCGTCCCGCTTTC

GGCAACGAAACGCGATGCATTTGCAAACACTACACTACACTACACCACTCCACACTACACTACACGCCACACCACGCCAC

ACCACCGTCCCTCGGCTTCCGCTCCGCTCCGCGTGCACGCCCACACAGAAGGACCTCGTGGCGAGGCTCCGGAGGGTCCT

GGGGTGCCTCCGGGACGACCCGCACCTCGAGGCCGAGGACACCTCCGGCTACCCCGGCCTGGCCGGCCTCTGCGGGGCCC

TCACCGGGAGCGGGGGCGACCCGCAGAACCGTGGCCGGAGGGCCGGCCCGATCCACCACAGAGACAAGGAGGTCCGCCTC

TACGCGGTGGCCGCCTGCATGGAGCTCTTTGCCCTCTACGCCCCGGCCGCCCCCTGGAACGAGGCCGAGACCCTGGACAT

TTTCGGCCAGACCATCCGCCAGCTGGCCAACCTGGGGCACACGCACACGCACACACACACCGAAACCACCAAGGGAGCGC

ACTTCTACGAGTACTACCGCATCCTGGAACTCCTGGCGGAGGTCAAGATCGCCGTCCTGCTGGTCGATCTCTCCAAGAAA

AACGACGACGACAACGACAACAGCGGCGGCAACAGCAGCAGCCATAGCACCACCAACCACAACGCCGCCCGCCCTTCCGG

GAACCAGACCAACCGGGAGGCCCTCCTGGTCCTGACGGAGCTCTTCCGGACCCTCCTGCAGTCCGTCCGCAACGGGCACC

CCCCCGAGGTCCTGGACTGCTGCCGAAAGACCCTGACCTCCTGCATCGAGGAATTCTTCGAGACCACCTTCCTCCCGGTC

CCCCTCCTCGACGAGCTCCTGGTCTGCATCGGACAGGGCCCGCGGGTCCTGGTCCTCAAGCAGCAGCAGCAGGAACAGAA

ACAGAAACAGAAACAGGAGCCGCCGCGCGGGAAACCCCGGGCCGGGTCCGGGGCTTCCGGGCGGCAGCCCCTTCCGGCCC

CGGTCGTCACCGTCCGGCAGAACAACCCTTCCTACGCGGTCGCCTCGGCGGTCGTCCGCGCCAGCGTCGAGCGCCTGTCG

ACCCCCATCGCGAGCCTCCTCAACGGCCTGGTCCAATCCGATCCGCGGGCCGTAGGGGCCTCGACGATCTCGAACCACGC

CTGCGAGGCAAGCGCCGATTCCAACGCCGGCGCCGGAAACGAGCCCCCCGTCCCGCGGGGGGTCCTGGAAATGGCCGACG

CCCTGGGCCGGCCCCAGAGGCAGACCCAGGACCCCCACGGGTCCAGCAACGTCTACGGCGTCATCCTGGAGCTCCAGAGG

GTCGCCCCGGCGATCCTTACCACCGTCTTTGGAAACCTGGCGAGCCACGTCGAAACCACCGACGTCGGCCAGAGGCTCCT

GGTCGTCGAGACCCTGGGCAGGCTCTTTGCGGGCAGCGGAAGCGGCGACGCCGCCGGTGGGACCGGCCGCTCCGGCCTTT

CCGTTGCCGCCAAGAACAGGGAGAGCTTTTTCCAGTGGCTCCAGCGCAGCGGGGACAGGCGCATCGAGATCCGCCGCCGG

ATGCTCCCCCACCTGCTGGCCCTGACCCGGGCCGGGAGCTCCTTTCTGGAAGAAGCCGGGGCCTCGTCGCTGGAGGCGAG

CCTGGCGCTCAGGGTCCAGGAGGCCCTCCTCCGGAGGCTTTCGGGGGATCCCTCCCCGGCGTTCCGGACCGAGGTGGTCC

AGGGCCTCTGCACCCTTTCCTACAACCACCGCAAGATACTGACCCGGAGGGCCATGGACCAGCTCGGGGAGCGGGTCATG

TCCAGGGACCGGGCGGAGCGGAAGGACGCCCTCACGGGCCTCGTCCAGCTCCACTTTCGGCAGTACACCCTGCACCACCT

GGCGGCGGTCCTGGAGGGGGGGGACGACTGTCCCATCGGGTCGGTCCTGGACGTTCTGCGCAGGTGCTCCTTCCCGGGAG

ACCGGGCTTCCGCCGCAGGGGCGTTCTCCGCGGAGACCGCCGCGAGCCTGATCGCCGCGAGGTCCCGAAACGCGGCCCCC

GAGTCCTGCGGGAGGCGGCGCGGGCCGGAGGAGACCGACGACGACGACGAGCCCGGGCCTCTGGAGTCCCCTGGATTCTC

CGACCGGGGCGGCAGGGACGACTTTTCCTATTACCAGTGGATCCCCTGCGTCCTCTTTGAGAGCGCCAGCTACTCGGACG

CCACCGACGCCGACATGCACTCGCGTGTCGTACAGCTGGTCGACGAGCTCCTCCTGGGGTGTTCCTCCCCCCATCCGGAC

AACCGGCGCAGGCTGACCTCCACCGGGAGGGCCACCGCGATGGCGATCGTCGTAGACGCCGTCCGCAAGCAGTCCCATCT

GGCCGGCCTCTGGATGACCAAGCTGCAGTCCGTCCGGGCCAGGCTCCAGAACGCTCTGAAGGCCTACCTCGAAGCGAGGG

CGGAGATCCGGAGGCACGAAACAGGTACGTCGACGGAAACGGAGCGGAAGGCAAAAAACGTGGCGCGAGGCGTCCGTGGA

AGCGCCGCCGTGGCCTTCCGGGAGAAAACGGAGAGCGCGGCATTCCGATCCAAACCACAATCGCTGCCGTCCTTTCTTTC

TCTGACCAATTCTCTGCTTCCTTTGCGTTTCAATGTGTGTACGACAGGTTCCGAGGAGTATTTTGCTGCGGACGCGAAAG

CCAAAGATCTATTGGAAACGGTAGCTTCGATGATCCCCCCGCCAAGCGGGGCTTCTCCTGCTCCCGGAGAACGGCACGCC

GTCCTGGAAAAATTTCATTCCAGCAAAGACAAGCGCGTCTTTCTCGTCTTGGGCACGATCACGAACCCGAGCCATTCTTC

AAAGTGTCGTGCCAAGGCCATCGACGACTTGCCGAAGCGGGTGAAGGCAACGGCCGGGGATGCCGTGTCTGCATGGGTCA

AATCGCTGGCAAAACGTTGCGCCATGGGAGATTTCGTAAACCTCGATGTCGTTCATCACTGTGTGCTGCTGGCCCAAGAA

TGTTTTCACGAAGGAGACTTGGATGCCACCCTCAAATTTTTGGTATGCGTTCAAATGGCGGTAGAATCCTTTCCTTCCCT

CTGCGCATCCGGGGAAGTCTTTGAGAACCTGAGCGAGTTGTTCCAGGATTGCAACGGTTCTTCTCAGAAAAATCAAACGG

AAGGACCCGCGATTGTTACCGCACTCAGCGCCATTCTTGCCTCCGTAGCGCCGTATAGAGACCCAAGCGACGATTCGTCC

TTGTTGGAGGATGATCTCTACAAAAAGCTTGTGAACCTATGTCGAAATGGCACCCCGGAACAAGCTCGGCATGCGGTGGC

AACCATTACATCGCTCCTCAAGCCGAAGAATGGTGCGGAACTCACCCAGGAAGAAACCAAAACTTTTCTTCCATTGCTCG

AAACTCTTGCCACTCCCTCGCGATTGGCCATAGCGTCGACTGGATCTTCCACGAAGCTGGTGTGTGTGTTGGCTGCTCTA

ACGGAGCTGGCAAACAATGCCCCCCAGGTGTTTGAATCTTCGAGTCGTGGAACGAAGGCGCTGAAGTTTGCCCTCGAAAT

GGTGTTGATGGGCCGCGCCCGTGTCGACGTAACAAGTGGTGACGACGACGACGATTACGATGAAGCGGACGATACAAGAA

CTCCCAAGCGTGGTCGAAATCGAAAGTCATCAAGCGCCAAGAGCAGGCATCTCAGCCCCAAGGCTACAGATACGAGTCTA

GTCGAAGACCAGAATCTATCGATTCCTTGCCGCACGATCTGCGCTGCAATTGAACTCTTGTCTGCGTTTATCCGATCTAG

CGTTTTCGTTGCCAAGAAATCTCGTTCGACCTTTACTCAAACGACTCAGGACATCATCGAACAATCGTTCAAGATTTTCT

CGCAAATATTGCGAGACCAAGGTTTGCCTCCATCATCAAGAGACCGCGACGTGTGTAGTTTTCGTCAAGATCGAGCCGCT

CTGAGGCAATGTGCGGCGATCCATCTCTTTCGTCTATGCGATACCCGCCTAGGACTCGATCAGAAACACCTTACAACGGA

GCGATGGCACACTTTGGCTTCGAGCTTACTCGACGACGAACCTGTTGTAAGAAAAGCGGCTATGGAAGAGTTTGGACTGA

TGATCACGGGCCATGGGAAGTTTTCTACGGCATACGGAATGGGCGTTATGGCACCCCGATTGCGCTTCGTTGCAATGTCG

GTGTTTTGTATCGATGGAAGTCAAGGCTCGCATTCCAAGGCGAATGGCAATTCAGCGAACATAGGAAAAGCGATTCACAA

TCAGAAAGGGAACATCGCTGGTTGTATTACTGACCTGAGGAAAGTATACGAATCATACGCCGTACAGTGCCGGGCGCAGG

GGCCAGAAGCTGAGAAACAGTTTGAGACATTTACCAAGCTTACCATCATGCCAGAATATGCTGTGCCCTATTCATTTCAT

CTTCTGACTTGTCGACAAGAAACACCCTCGTCGACTGGACCAAGCACCGGGAAATCTAGGAAGGCGAACGACGATGATGA

GAGTGGACAAAGAATCCTTCGAAAACGCTTGAAAGCTCTGTACGATCCACTCGTTCTCCAACTCGGTACATCGGCCGATA

ACATCAGCTTTCTTCTCCGCATGGCAGAAATGCTAGCAAAATCATTCCAACCTATAGGTTGCTCGCTTAGCTCCCCAGGA

AGTGACAACAGCTCAAGGGACGGGGATAAATTGAAAAATATATGTGCGACGGCACGAGAGGTATTGTTGTCGTACGTGAA

GACAGACGCGAATCTTGACACGCATCCCGGTGCGATACGGATGCCTGGCAATCTCTTCCGCAAACGACAAAGTCGCAAGA

GACCAGTACAGGAAGACTCCATCACGACAGATACATCTCCAATCATTGACATGATGATTACTACGCAAGACAGTGAGATT

CAAAACCAAAAAATAAGTTCAAACCTGAATCGAAAAAGACCAGCTAGCTCTAAGGAACACGAGAAGCACACCCAGGATCA

TCCTTTTGGCAACAATAATACCAAGCAAGAGCAAACCCGAAGATCTACAAGATCCGCTACAAAGCACGATGTCGATGGTT

CAAAAACGACTGCAAAAACTGATGAAGCGTCTAGAAACTCCCATGACACTCAAGCAAGCATCGCAGATACGAGTCGGAGG

CGAAGATCTCTTCGAATATCGACAAGAACAGCATCCATTGGTTCAACTAATACTTACGGAGATGGTGACTCGAGAGTGTC

ATCTAGCACAGACGAAATATCTGGATATTCAGTTGACGGAAATGTAGGTATTACAAGAAAGAATACCCAACAAATTGCGC

CTCGAAGATCCACAAGGTCAACTAGATCGACTAAATTCGACGATTCGAGGGACTCAAGCACATCAGGTAGCATCAGTATT

GAACATGGAAATGAATCAACCGAGAATGGAACAGGAACTATTGAATCAACCCCGTCGTCGAAGGATGTCGAGAGCACAGT

TACCTCTACTCGTGGTATTATGCTTTCGACTGAAGGTGAAGACGACCCTTCGAGTTCTCGACTTGGAAGCGGCTCCAATA

AAAAGCGACGATCAGATGCTTCCAAAAGTATCTCCGCCGACAGTCGCGTACACTTTTCTCCTGAAATTGATTTTGGAGGG

TTGTCTCCCATCAACAGGAGGAGTTCCCGTAACAGTAAAAATGAGGAGCTGTTACTGAGTTCGTCGGAAACAAAAACAAG

AGGCACAACACCTCCATCGTCCCTCCGGAACGCCTCGTTCCCAGCTACTGCATCTGCGTCAGTTGCTATTGGGTCGCCTC

GGCCCCATGAAAATAGAGACTCCAAAAGCCCAACAACTGTGAGTGAATCCACTGCCAAAAGCTCAATTGGACGTGTTTCA

TCTCAAGGAAATGAAAAGACGAAGCCTCTAGGGGACAAAAAGGCAGAAGTTGCGCATAATCGACGAAGAAAATCATCTCG

GAAACTTGCTGTCGACGACAAGGAAAATGCGACTGCAATTAAGAAAAAAGGAGGCCCGAAAGATATCAAGATTGTCCGAT

CGAAACAAAGCTCAAAGTTCAAAGCCAAGAAAGTTGTCAAGAAAACGGCGCTCGCCAGCGTTGGAAAGCGCGGCAAAAGG

AAAAGTTCGAAACCAATTGATTCCTTTGACTTTGAGGGGTAAAATACTGGATGAGAAATAGTCGATTACATTGCACTGG

>PSNMU-V1.4_AUG-EV-PASAV3_0079350.1 class=Sequence position=[PsnmuV1.4_scaffold_39-size_249521:219728..225655 (- strand)](http://gbrowse255.tgac.ac.uk/cgi-bin/gb2/gbrowse/maplesod_psnmu_v1_4_gbrowse255?name=PsnmuV1.4_scaffold_39-size_249521:219728..225655) SCC3

AACCGAAAACCGGAGCCAAAGCATCCAATGACAAGAAGCGGCAGCTCACCAACGGATTCCCCCACGGTCCGGAGATCCTC

CCGTGCCAGACGCCCGGTCACTACGATCTACGACTTGGCGGCGGCCAAAACAAACACCGGGCCGGACCGAAGAAAGAGGG

ACGTCGACGGCAGCAGCAGCAGCAGCATCGACGGGGATGAGTGAGTGGTGCCGGAGCGTTCGTTTCTTTGTGTGCGGTGT

TTGTGTGTGTGTGCAGTATGGCGTGTGCAATCCGGGGGGGGACGAACCTTGCGTTTCTCGAAGCAACGACGATGTCGGAG

GCAAGTTCGGACGGAAACCGATCCACGATGCAGAGCATGCAGAGCACCAGCCTTTGTATTTTCATTCGCATTCGAATTTG

CATTTTCATTCGCATTCGCTTTCCCACTGACCCTCGCCCTTGCCCTCGCCCTCGCCCTTGCTCTCGGCCTCAGGGAGAAC

TACGGGGACAGCGGCGAGGACCATCAGGATTCCTCGAGCGAAAACGACAGCGACGAGGATGCCTCGGCCGCGAGGACTCC

GGCACGAAAGAAATCCAGAACCACCGGGGGCGTGGCCACGAACCGGCGGCGGTCCTCTGCGGGTGCGTCCCCCGCCGCCC

CGGCGAAGCGAAAGAATCCCGCCGCGGGGGGCAGGGGCAGGGCCCGSGCCGCGGGAACTCCCGGAGGAAGACGCTCAAAG

ACGGGCCCAAAGACGGGCCCTTCTCGCCCGGTGTCCACCCGGTCGGCCTTCACCAAGGCCCGCATCGCGCAGGCCCTCGG

CATGCTCTCGAAAAAAGCCCTCAACAACCACGGCACGGACGAGGACGACTCCGGGAACAACGCCGACGCCGACGTCGACG

TCGACGAAACCCCCGAGAACAGCCTCCTSGCGGCGCTCCTGGCCTCGGCCAAGCCCATTCCCGGGATCCCATCGACGGAG

CGCCCCAAGACCTTTTACTGCCAGCAGCAGAGACAGCAGCGGCTGCAGCTGCWGCTGGAATCCCACGGGGAATACGGGAA

GCCTCCCAACCGGGAGAAGCGCACGGCCGCTATTATGGGGGTTTGCCTGCCCCAGCTGGACGGGATCGTTCGCCACCTGA

TCGCCACAACGGACCAACACGGGGACAACGGGGGACCGCACGAGCTSCACGTCAGGCTCCTCAACCTCTTGTTTCGAAGC

GTCGGGGGATCCACCCGCTCCAACATTCCGGAGCACGGCATTCCCAAGAGCCACGACAGCGACAACGAAAACGAGAACGA

AAACGAGAACTACAACGAAAACAGCCATGCCTACACCGACCTGGACGAGCTCGAAGACAACGACTGGGACGATCTCGTCT

CGAGAGTGGTGACGGTCATGAAGAACGAATCGGATGCCGACCAAACCCTTTTGGTGGTGGCCGACGACCACGACCACGAC

GACGACGACGACGAGGAAAWCTATCTCGACGGCGCACCCCCGGCGCCACCCGTCCTGACGACACGAGCCATCGGGATCGT

TGTGTACAGGACGCTCTACAAGGAGTTCTGGTACCGGCTCGGCCACGTCCTGCTGGCGCATTCGCGTTCCCAGCCCCCAC

CGATGGTCAGCGACGAAGACGAAGACGAGGACAACGAGTCCGACGAAGACACGGGGGACAGCAGCGATAGCGATGCCGAC

AGCGATGCATCGCCCCGCAGAAAAAAACGCAAGGCCTCCAAACCGAACAAGAAGGCCCCCTCCGTCGGAACCGATGCCGC

CACGCGGGGATTTTCGTCCGACCGCTTCCAGCTGGAAAAGGTCCGCGACCTGATCCTCCGCATGACCGAGCTGGTGTCCG

TGGGACAGCCCGACCTGCGATCGGCGGCCACGACGGCGGTCTTGCAGCTGGCCACCGCCTGCGTCGAGCGGACCGTCGAG

CTCCAGCAAAAGATCGCCGTCGCGGCGCGCCAGCTGAGGGTTGCTTCCCGGCAGGGGTCCCGGGACAAGCAGCAGACGCT

CAAACACCGGCTCGACAACTGGAAGCGCCACAAGGCMGAGCTCGAAGAAATCGTGGAGGGACCAGTCTTCCAGGGGGTCT

TCATCCATCGGTACAGAGACGCAAACGAGCGGATCCGGAGGGACTGCCTGCGGATGCTCTCTGGAATCTCCCTGATCCGG

CCCGACATTTTTCTGGTCGACACCTACCTCAAGTACTTTGGGTGGATGGCCTCGGACAAGGCCCCGTCGGTCCGGATCGC

GGCACTAGAGGGGCTTTCTGCGCCCTTGGCGGAAGCGCGGTGGTCGGCCCAGGGGGTGGCAAAGCCGCCCTTCTCCATCG

ACATACGGGCCATGCACAACGTCCTCTTCAAGTTCCTCGACCGCATCGTWGACTGTACCGGCGACGCCGACAGCACCCGG

GTGCAAGAGATTGCCATCAAGACCATGCTCCGCATGCTCCGGGAAGAACTCCTGGACGAGTGGGACGACGACGTGGCCTG

GGACAAGGTCAACCTCAAGGCACTCGATGCCCTGTCCTCTCCGAGGGTCCGGAGGGACGCTCTCTATTTTGTGATGGACC

AGCTCGATCCCTTCGATGCGGACGACGACGAAGAACGCAAGACCGTCGGCGACCGCAAGCAATCGGACCGGTTGGTGGCA

ATTGCTAGGTGGTGAGTCGTTCCGGGGTGCGGGGGTCCGCCTCACACTGGTTTGCTTTGATTGTCGATTGTTTGTAACAT

TCCATCTCCCTTTCTAATCGTTGGTTTCTCTCTCTCTCTCTCTCTCTCTCTCTCTTGATTCCCTCCTTTTGCAACGACGA

CAAAAAACAGGTGCGCAAACAAGCTCTGCGACGGATTTATTCCGATCGACAAGCTAAACATTCAACTGGTGGACTGCATC

GTTCATTCGATTCTGTCCATGCCGGAGCACCGAAGTCTCGTTCTCAATTGGCCGATGATCCTCCGGGCGATCCGGAGCGA

GAACCCCCAGCAGGGATCCGAGACCGAGCGAGAAGATACCGCGACCCAGCGAATCCTCCTGCGGATACTGGCCACTTCCG

CGGAACTCGAGCGCCAGACCGTGGCAGAAATCGAGAACCGCGATTCCACCATGACGGGGACGACCAAGGCGCTCACCTCG

AGAACACGCAAGAAATCGTCCAAGCACGCCGCGACCGAGGCGAGCCTCGACCAGTTGTCCACCGCGCTGCTGAAAAACCT

TCCGCACCTCCTCGAGCTCTTCAAGAGCGACGTCGTGTCGCTYCGCGACGTCACCAAGCTCCCCCCGACCATTGGCCTTT

CGGCCCTCGGACTCCCGTCCCGGAAGAGCGACYTCCAAACCCTTGTCAAGACGCTCTGCCAGCTGTACCTCGACTCGACS

GACGAACGCGTCCTGCAGAACATTGCGCAAACCCTTTCTCGATGGGTGGAGGGCGACCACACCCGGGTGTCGGAGGTCAA

GATCAACCTGAAGCGATTGTCGCGTGCGATGCAGGACAGATTGATGGACCTCTTTCGGGAGAGCGATCCCCAGAATAACG

AGTACGGCGAGAAGAGCAGATCCAGCCGGCGGCGCAGCAGCAAGACGCGTCGCCGTTCGTCTTCTAGCCAAACGGACGGT

GCKAGCACCGCTGGAGGATCCACCATCTTTTCTGCCTCGCCACAGGCCGACCTGGAGCACTCCATTTCGTTGCTAATGCT

TCGCTGGAAYATTCTTTTGATGCAGTGCCAGGCAARGTATCTCTTCGAGACCCCAGATGAGGACGAAGACGAAAACGAGG

TGGACGGACTATTCTTCACTATATCCGAAGCCATGGGAAAGAGGCTCTCGGACAGAATGCCAACCCTCGATCGCGACACA

AGCGACGATGCTACTGCCGTAACGACCCCTCCAATCTGGTCSGAGTACGATCCGGACGTCCACGAGGCAGTTTCCAGAAC

CATCGGGCGGTCGCTAAAAGTATTGCTGCTCATCCTATCTTACGAATTACACGATACGCTGATGGATCGGAAGGAATTCG

AGTCCTCCGAGAGCGAAACGACAGACGACGTKGAAGTCGACGAGTACAGCCTTCCTGTCCTGAAACTAAGAGACAACCTC

ATAAAGCTTCTTGGTCTTTGTTTTGATCAACACCTTCCACGGATCGAGGGGGTGGCGTACAGTAGCGAGCAGCACGACTT

TGCCACCGCGGTCCAAGCCGGYGCAGGAGAAGTAGCGTCGGACCTGCGGACTCTCTTTCCCTGGGACTGGTCGAAGGCTG

CCGATCCGGTGCGCAGATCACTGGCACTGACTAACGGCGAGGACTTTACCCTGATGCTCTCCGGGTTCGCTCGCTGGTTT

CAGTCCAGGGAAGAAGAGATTTCTGAAGACACGGACGACGCTTCGCCGGATTCTCTGCTGAGAGAGGCCATCCTCCCACT

GGCGCGGGTGACCAACATGAATTTCGAGGGCTATTTCCGCAAGGAAGCCGCCATGATTATGCAGCACATTAGTGGCAGCG

GAAGCTTGGCGTCACAGACCATACTCGCCTTGTCGCGCTCGCTGAAGAAGGTACGCTTTTTTCGGCACGGTCTGCGCCGC

TTTTATTCATTTTTCCTTTCTGTCGCTCTTTCCTCCAGCTCTAAGCCGTTTTTGGTTTTGTTTTATTCTATGTTGTTGCG

TCGTATTCATTACACCCACAGACCAACCCCGTTCGCATGCTCGAATCTCAGATGGCGTGTTTGCGGCTTGCTTTTGAAAA

CTGGTTGGATAACGARCCAGATATTCCTGAAGARACCTCGCCAWCGGAAGAAGAGCTACAGGCGTTCGAAGAAGCTGAAA

AGTATCACTTTGATTCATTTCTTTCGATGGAACAAGTTGCTTCAAGGCTTTCGTCAACCCTTGGTGTTGCTGGAAGAATC

AGCAACGCAGGTCTGAGAAAGTCGATTTTTGGTTTCATGAGAGAAGGTATTCGATACGCTTTTGATGGCATCGAAGGGAA

TCAGCAGGACGACAACCTGATGATTGGCAGTCGTTTGGCTTTCTTATCGATTCTYTCGAAGTAGGTTTCTCAATTCGTTG

CATCGTTTCAGCATAGCTCCATTCAAGCCATCGTGCTTGGGTCGTTTGTGTTTTGATTGTAATTCTAACATCTCCGTTTC

ATTGCTTTCACAAAGATACGCAATCTGGATAAAGAAAGAGAAGGAGCATCTAGAACACGTTTCCGATTACCTGATCGAAA

AAGAATCTGAACTTCGTCGTCACCCTGAATTCGATGAGGTACACGAGGAAGATCTGAACACGCTCGCCGACTTCAAAAAG

GCTCTGGGGATTAAACGAGTCACCGAAATTTCCTACGAGACCGACGATGGAGCCTCTCGCGTGGATGCTGCTTCCTACTC

AGTGGCTACGCCAACCACTACAGCCTCCGGAGGATCGTCTCGCCGCAGGGTTTCTTCTGCTACGAGCCGTGGGAGCGTAT

CTTCTCGTCGGTCCGGAACTTCCGTACAGAGCGAGTTGTCACCATTGTACGAAGAAGACAATACAATGGTGGGAGAAGAC

GACACAGAGTCGTCCCCGACTCAACAGAAACGGAGACRATCCCGTTCGCAGTCGAGCCGCAGAGAGAGATCGGGAAGAAG

TGGTACGAAGGGCGCACACGGCCGAGAAACAATTCTAGAAGAAAACGAGCAAGAGAGCAGAGCTTAGAGATCGCTTTTTG

GTCAATTGCTACATTTTCTTCATGTTCACTGCATCRTACATTTCTATTCAGAACACTTGTGCGAGACATCGTCTTCCCTG

YTGTGTCGAATGGGATTTGTTGGTATAAAGAATATCGATTCTCGTGCAGCTGCACGGTGTTCTGTCGTGAGATAGTCCAT

CAAGATTTATTTTGTTTCGGCCGTTACTACMAGGGATAACTACTTTCTTTGAAAATATGATTGAAAACATTAAATATACG

GTATTTTA

>PSNMU-V1.4_AUG-EV-PASAV3_0072170.1 class=Sequence position=[PsnmuV1.4_scaffold_343-size_50805:31467..34095 (- strand)](http://gbrowse255.tgac.ac.uk/cgi-bin/gb2/gbrowse/maplesod_psnmu_v1_4_gbrowse255?name=PsnmuV1.4_scaffold_343-size_50805:31467..34095) *RAD21*

CCCTTCTTCGTTCCGGTTTCAGTTTTCCTTTTCGATCCATCTTCCTCCGCAATCTTGCAGCAACATCGGCAAGAGGAACC

AACGCGAACGCTGGTTTAGGTCCATCTATAGCGATAGCCACCAAAGCACGCAGAGGCCATGTTCTACTCGCAGGTGATCC

TCGCCAAGAAGGGCCCCCTCGCCAAGGTGTGGCTCGCCGCCCACTGGGGGGACAAGAAGCTCGCCCGGCCGCAGATCTTC

GCCACGGACATTTCCCAGTCGTGCACCGACATCATGAACCCCTCGGTCCCCCTGGCCCTGCGGCTGAGCGGGCACCTGCT

GCTGGGGGTCGTCCGGATCTACAGCCGCAAGGTGAAGTACGTCCTGAACGACTGCACGGAGGCCATGCTGAAGCTGCAGA

TGGCCTTTTGCAACCAGGCCGGGGGAGGCAAGCTGGGGCTCCGGCTGGACGAGCACGGGAAGCCCGTCCGGGGCCGCGAG

CTCCTCTCGGAGAAGGACGCCCCCACCCACCAGCTGGTGTCGAACTTTGGGGAGTACGACCAGATCCACGTCGTCGAGGG

CTTTTGCCTCCCGCTCCCGGAGGACAACGAGTGGATCCTGGCGGAGGACGAGGCCGGGATCGGGGGCGGGAGCGTCGGGG

AGGCCTCCCAGGAACTCCTGCTGGCGGCCCACCTGCCCCGGAAGCCCACGGACGGGAGCGGGAGCGGATCCCCCCCGGCC

CCGGTGGAGGAAGAGGAGGCCTGGGCGCCCTTTGATCCCGACAACGAAGACGACGACGACGACGAAGGACTCGCCGACGG

CAGCCGGGTGTCGGACATCGAGGTGGTCCGGGCCGCCAACGATTCTCTCTTGAGCGACGATCAGGTGGGTTGCTTCGGAA

GTCTTTGTCGATCTGTGTTTGTTGTGTTCTTTCGTGTCGTGTCGTGTCGAGTTGAGTTGAGTGGACTTGAGTTGCATACA

GTAGTCTTGCGTTGACCTTCTCGTTGCGTTGCCCTCTCACCCGCTCGGATTCTGTTTTTTCCATACGTTCGGTTCGGTCC

GGTTTCGCATCGCAGACCCGGCGTGCGTCTTCCCTGCTGGACAAGAGCGCCCTCACGGCCAACAGCTGGAACATCAACAA

CAAAAACATCGACAACAACGAAAACAACAACGAAACCATCACCAACACCAACCCGCAAGAAGAGGACTACGACTTTACCG

TTCCCTTTGGGGAAGACGACTCCGATGCCGACGAGCCCGTCGCCACCACCCGCCTGTCCGATGCGGGACTCGGCGATTCC

GCCCTGCAGCTGTCCAGGGACGGGGGCACCCCGCTCTCGACCGGCAACGCCGGGCTCGGGCTGGGCCTGGATGCGGACGA

GGACTCGGCCGGGAACGCGGGGGGCCTTTCCGACCCTTCGAAACGGGAGCAGTTGCAACGGGAAGAGGAGCCGAAGCCGC

CCCCCCGCAAGCGCCGCCGCAAGAGGCGCAAGGTCGTCATCGACAACCACGAGACGGAGCTCACCAACGACCACATCCGG

GGGATGCTGCGGTCCACGGACGACATCGTCCGGCCGATGGTCCACCCGGCCTCGATCTGGGACGAGGAGGGCACCGGGAA

GGACTACCGGGCCCTGGTGCTGGATCGCATTGCCGAGCAGAGGGGCGCGTCCCGGAAGCGCAAGAAGGGCGTTGCCTTTC

GCGATGTTCCGGGGCCGGCGTCGAGCGGGGGGGTCCCCTCGCTCACACGGCCCTTTCTGGCCGATGCCTCCGAGTCCGGC

GGGCCGGGCCTCCACCCGGTCCTGCAGCGGCTGTGGGAGGACAACTACTGGAGGGCGAACGAGCGGCCCTGCCCGTACCG

ACGCCTGGATCCCGGCGGAGACGGCAACGACAACGACAACGACAAAGACGACAAGGTCGTGGACGACGTCGAGCACGTCC

GCAGGGGAACCGCTGCCTCGGACGACGAGAGCACGGTTGGGGGCCGGTCCGAGCTCGGGGTCGGGGCCGAAAAGGACGAC

GACGACGCCCGTTCCCGGCTGTCCACGGGCCAGGAAATGCCCCGGGACGACGAGATGGACTTTCCCGTCGCCAACGACGA

GGAGGACGAGGAAGACGACGAGATGGACGCCCCCGTCCCCGACTTTGGAGACGACGAGGAAGACCCCACCAACCTCGCCG

CCGGAGGAGACGACGAGGACCTACTGGACCTCGGCATGGTCAACGACATGGTCCTCGATTCGGACGAGGACGATCGCCAG

GACGACGGCGAGGACGACGAAGACCGCGAGGCCATCGGGGACGCGGCCTCCTCGTCCACCAAGTGGCACAAGCACACGGT

CCGGGTCTTTCGGCACCTCAGGAAGTGCCTGAGGGACCCGAACGCCGACACAGACGAAGACGGCGGCATCGCCAACGAGG

GGCTCCCGGACTCGGTCGGCTTTGCGGACCTGACCAAGAACGTGGTGTCCCGGCGCAACGCCTCGTCCGTCTTTTTCGAG

ATGCTCCAGCTCAAGACCTGGGACTTTATCGAGCTCGAACAGGAACGGGCCTACGGGGACATTGCCATCTCGGCCGGCCT

CCGCTTTGGGGAGGACGCCCCCGAACACTAGGCGAACGAAACGAAAGCGAAAGCGAAACGAAAGCAAAC

>PSNMU-V1.4_AUG-EV-PASAV3_0108120.1 class=Sequence position=[PsnmuV1.4_scaffold_67-size_186386:107180..108785 (- strand)](http://gbrowse255.tgac.ac.uk/cgi-bin/gb2/gbrowse/maplesod_psnmu_v1_4_gbrowse255?name=PsnmuV1.4_scaffold_67-size_186386:107180..108785) ***SPO11-2* Incomplete gene model**

GTAGAAGACGAATAACCGTCGGTAACGGTTCTGTTCGAAGAAGCGGAAAACCGAAACTGGAAACTCCTCACAATTTCCAA

AATAATTCGTACATTCAAGACCATACTGTAACTCAAAAACCTACAGTTGCAATACCGGACAAGCTCCTTCTTGCTTYCCT

CGAGKCTATGATTGCTTCCACAATCGCGAGACAACAAGCTAAGYAGTTCCTGTCCCCAACCAGACGAACTCTATCGTACC

TTGCACTCGAAACAGAAAAAGGTCACCGGTCACACCTCGCAAAAGAAACCATCATGTACGGCAAGCACCAGCGATATCGT

TGTGGTTCTCACGAACAGCTTCGCAATCGATTCCGCGTCCAAGATTGCGGAATCTACAGCAAAGGATTCGACCCCCGCAT

CTTGTCCAACGACACTTTCAAGAGCAAGGACAAGGACAAGGACAAGGACAAGGACAACGACTCGTTGTGGGACGAAGACG

ACGCAATTCCGGAAAAAGCCGCCATGTATCGCCTCGACCCAAGCCGGAGCTTCCATCCGTCTTCTTCTCCCTCTTCCCAA

TCGGACAGCGAGAGCGACGAAGAGCAGCCGAACGATTACCTTTCCCAAGGGAATCGCAACAACGATAGCTATGGCCGCAA

AGACCACCGGGGTGACGCATCTTTCTCGATTTCTTCCTCTGCTTCTTCGGACGAAGAAGATTCCACGGTTTCGGTCCGAT

CGGTAGTGGAAATYATCGAGGATTTTATCCTACAGACAGTGGTTGAGCCATTGTCTCGGGATCCGAATCCATCCGTACCG

GAGATCAACGYCACCGTCGATGCCATTGGGTTCAAGCGCAAGCTGTCCCTGCATCATCTCACCCAGGCACGATCGCTGAC

CTCCATTCTCAAGGTGGCAGCCTTTTGCTACGACCTGCTGGCTCCCGAAGACAAAGAAGAACACGAAGACGGACACGAGC

GTGACGCTGCGAACGGGAAGGGGGACGGTTGCCGGGTTYGGTACAGAACGCGAAAAACCACGACCACGCGAGAAGTCTAT

TACTTCTACGTGACCTATTTTCGAGACCAGAGAGAATGCGACAAGGCTATTTGGGACTTGGTGTGCATCCTTGGATTGCC

CTCGAGGCAATCCCTGGGYCTGGTGGCATCGCCAAAGGGCTGGTTTTGCGGTTCGATCGATGTCTACAACGCCCGCACCG

GCGAGCTCAGGTTCAACGGGAGGGATCTCGATGCGCACGGAATGGCCATTACTCCCTCGACCTACGACAGCTACTCCAGC

GACTTCAATTTCAGCTACCTCTGCAAGGACGACAAGCRCGCCGACGATCCTCTCCCCATTCAAAGCGGTAGCGGCATTCG

AATCGAAAGCGACGCAAAATGCATCCTGGTCATCGAGAAGGAAGGTGTGTACACCCGATTGTCCGAGGACAAATTCTTTT

TAAGATACTTTCCTTGTATATTAGTTACCGGTAAGGGCTTTCCTGATGTGGCAACTCGTCGTTGGGTGAAGAGGATGCAA

AAGACACTCAAAATACCGGTCTATGGATTGTGGTAAGTTGCTTTGGGTTGGGTTCGGTCGGTGCGGTATAAAGCAAGGAA

GGTTTG

>PsnmuV1.4_aug-pasa-abinitio_v2_0049780.1 class=Sequence position=[PsnmuV1.4_scaffold_67-size_186386:106351..108555 (- strand)](http://gbrowse255.tgac.ac.uk/cgi-bin/gb2/gbrowse/maplesod_psnmu_v1_4_gbrowse255?name=PsnmuV1.4_scaffold_67-size_186386:106351..108555) ***SPO11-2* Corrected gene model AUGUSTUS V2**

CTATCGTACCTTGCACTCGAAACAGAAAAAGGTCACCGGTCACACCTCGCAAAAGAAACCATCATGTACGGCAAGCACCA

GCGATATCGTTGTGGTTCTCACGAACAGCTTCGCAATCGATTCCGCGTCCAAGATTGCGGAATCTACAGCAAAGGATTCG

ACCCCCGCATCTTGTCCAACGACACTTTCAAGAGCAAGGACAAGGACAAGGACAAGGACAAGGACAACGACTCGTTGTGG

GACGAAGACGACGCAATTCCGGAAAAAGCCGCCATGTATCGCCTCGACCCAAGCCGGAGCTTCCATCCGTCTTCTTCTCC

CTCTTCCCAATCGGACAGCGAGAGCGACGAAGAGCAGCCGAACGATTACCTTTCCCAAGGGAATCGCAACAACGATAGCT

ATGGCCGCAAAGACCACCGGGGTGACGCATCTTTCTCGATTTCTTCCTCTGCTTCTTCGGACGAAGAAGATTCCACGGTT

TCGGTCCGATCGGTAGTGGAAATYATCGAGGATTTTATCCTACAGACAGTGGTTGAGCCATTGTCTCGGGATCCGAATCC

ATCCGTACCGGAGATCAACGYCACCGTCGATGCCATTGGGTTCAAGCGCAAGCTGTCCCTGCATCATCTCACCCAGGCAC

GATCGCTGACCTCCATTCTCAAGGTGGCAGCCTTTTGCTACGACCTGCTGGCTCCCGAAGACAAAGAAGAACACGAAGAC

GGACACGAGCGTGACGCTGCGAACGGGAAGGGGGACGGTTGCCGGGTTYGGTACAGAACGCGAAAAACCACGACCACGCG

AGAAGTCTATTACTTCTACGTGACCTATTTTCGAGACCAGAGAGAATGCGACAAGGCTATTTGGGACTTGGTGTGCATCC

TTGGATTGCCCTCGAGGCAATCCCTGGGYCTGGTGGCATCGCCAAAGGGCTGGTTTTGCGGTTCGATCGATGTCTACAAC

GCCCGCACCGGCGAGCTCAGGTTCAACGGGAGGGATCTCGATGCGCACGGAATGGCCATTACTCCCTCGACCTACGACAG

CTACTCCAGCGACTTCAATTTCAGCTACCTCTGCAAGGACGACAAGCRCGCCGACGATCCTCTCCCCATTCAAAGCGGTA

GCGGCATTCGAATCGAAAGCGACGCAAAATGCATCCTGGTCATCGAGAAGGAAGGTGTGTACACCCGATTGTCCGAGGAC

AAATTCTTTTTAAGATACTTTCCTTGTATATTAGTTACCGGTAAGGGCTTTCCTGATGTGGCAACTCGTCGTTGGGTGAA

GAGGATGCAAAAGACACTCAAAATACCGGTCTATGGATTGTGGTAAGTTGCTTTGGGTTGGGTTCGGTCGGTGCGGTATA

AAGCAAGGAAGGTTTGTCATCGATTTGCTAGGTTTCTTAATCGTTTGCCTTTTTGTTTCCACAATCATTGGGCCAGTGAC

TGCAATCCTTTCGGAGTTTCGGTGTTGGATTCGTACCGGCACGACCAGGGAGTCAGACTCCACCGAAAAATCAAAAGTAG

TTCCAMGCAAAGAAAAAGTAGGAGCAAGATCAGAATCGATGAACCGAGTGCAAGCGGGAGCGAAACCGAACACGATAAAA

CTCATGATTCCCCCGAGGCACTACATTGGATCGGCCTATTCCCTTCTCAGATCGAAACAATGGATCTTCCGCCYCAGGTG

TTTCAGGAATTGACCAGCAACGACACGAAACGATTGGACTCCTTACTAGCGCCAAAGACACCGAACTCGGACTGCTTTGT

TGACTTGGGTGGGAGAAACAGATCCAGGTGGATCCGTGAACTCAAAGCGATGCGTAAGTACAAGGTTGAGCTCGAAGCAC

TCCACTGGAAGGGGGCAGATTACCTATGTCAATTCGTATACAATGCGATGCTTTCCGAGTATGAGAGATTTCGATAGTGA

CAGAGACAGCCAAAACGAATCGACACATACATCTGGATCCTCAGGATGCTRTTACTTTAATCTTGCGAATAGCGATARAA

AGCTTCAACAACAATCTAAACACGGGATACACATTGATACACAAGCAGAGATGAATCTCGCCCCTACACTCGTACTGGAT

ACGTGAGAGCCATCTTAGTTCAGCAAAGCATATACTGGAACATTGAAGTCCTTGTATGTAGTTACACATGTTGCAGCCCC

>PSNMU-V1.4_AUG-EV-PASAV3_0081370.1 class=Sequence position=[PsnmuV1.4_scaffold_4-size_463035:272510..274375 (- strand)](http://gbrowse255.tgac.ac.uk/cgi-bin/gb2/gbrowse/maplesod_psnmu_v1_4_gbrowse255?name=PsnmuV1.4_scaffold_4-size_463035:272510..274375) *TOP VIA/SPO11-3*

CGCGAGTTTCGCGGGCCGCGATTCCGAAATCATTTTGATGATCATTTCATCGAGTGTCAGCCTTGCCTCCCATCGATCCG

AGTGTCACGAACACAACGCAATACAACACAACGCCCGGACTAGCACGGCGTACCACTTCTTCGCAGCCAACAACGCACAA

AACGCGGTGCCATGTCGACCAGGAGAAAACCGCGAAAGGCGTCGGCGGCGACTTCGGTTCCCTCCACACCCACGCCCGTC

ACGTCTGCCACCACCGGAAGCAGAAACGCCGGGAAACGCCGAAAACGCGAACCCGTGAAGCTCGCGGCCGATTCCGATCT

GGTCCTGGCCAAGTGCCGAGCCCTCAAGTCGAGAATGCGCCGAAAATCCGAGGAAAACGACGGATCGGATCCGACTTCTT

TCGATGACAGCGTCCCCGATGCCGTCGGCAGCAACAACGGCGACCAAAACATCCCGAGCGACATTGTGGAGGTCGCCGAG

CTCTCCGCCACCGAGGTCCTGGAGGGCATCGAGGGGGTGGCCCTCCGGATAACGAGACAGGTCCTGGCCAAGAAGGGCTT

CTCCCTGGAAATACCGAGCCGGTCCGCCTCGAACCAGATCTACGTGAGGGAATGGGACCGCATCGTGCTGGGGGGGAAGC

GCATCGGCCGGAGCTTTACGAACGTCCGCGAGTCACGCAAGTCGGCGATCACGCTCAGGGTGATGCAGCTCCTGCACGCC

GTCCTGGTCAAGCGGATCCACATCACCAAGCGTGATCTTTTTTACACGGACGTCAAGCTCTTTGTCGACCAGGCCGAGTC

GGACGGGGTCCTGGACGACGTGGCCACCATGATCGGGTGCACGCGGTCGAACCTGCACGTGGTGGCCAGCGACAAGGGGC

TGGTGGTCGGGCGCATCCAATTCGAGGAAGACGGGGACTTTATCGACTGCACCAAGATGGGGGTGGGCGGGAAGGCCATC

CCGCCGTACATCGACAAGATCGAGAACATCGAGAGCGATGCCGAATTTATACTGCTCGTGGAGAAGGAGGCTGCCTACAT

GCGAATGGCGGTGCGTTTTCTATTGCGTTGCTTGGCCTTGTGTTGCATCCGAGCGATTCGATTCCGCCCCGTCGCAGTGT

TTTTATGGGGCGGGGGGGAATTGTGTGGGAGATGGACCTCGTTTGTTCCATCGAAAAATTGTTTGTTCGTTTCCCATCTC

ACAACCGCGCATTTCTTGCCAATTGAACTATCGATGTGCCATACCCGCTTGAAAACGGAACAAAAACACGCAACAACAAC

AACAACAAACACCAATTGCTCGCTTCTTGTTGTGTACTGAAACAGGAAGATCGGTTCTACCACAAATACCCCTGCATCGT

GATCACGGCGAAGGGTCAGCCCGACGTAGCCTCCCGGATGTTCCTGTCCCGGATCACCCACGAACTCAAGATTCCCGTCC

TGGGGCTGGTGGACTCGGATCCGTACGGCCTAAAAATTCTGTCCGTCTACATGTCGGGGAGCAAAAACATGAGCTACGAC

AGCGCCTCTCTGACGACGCCCAACATTATGTGGCTGGGGCTGCGGCCCTCGGACCTGGACAAGTACGACCTCCCAGACCA

GTGCCGACTGGACATGACCGAGAGCGATATCAAGACGGGGAAGGAACTCATGACGGAAGACTTTATCCAGAAGAATCCAA

TGTGGATGAAGGAACTGGAAATCATGGTCAAGACCAAGAAGAAGGCCGAGATCCAGGCCCTGTCTTCCTTCGGGTTTCAG

TACATTACGGAAGAGTACCTGCCAAGGAAACTCAAGGAGGGGGACTGGATTTGATTCTGAATCAATAAAAGCACCGAGTC

GTGTGTAAGAAAACGAACAAATATGT

>PSNMU-V1.4_AUG-EV-PASAV3_0001820.1 class=Sequence position=[PsnmuV1.4_scaffold_1-size_679566:204986..210308 (+ strand)](http://gbrowse255.tgac.ac.uk/cgi-bin/gb2/gbrowse/maplesod_psnmu_v1_4_gbrowse255?name=PsnmuV1.4_scaffold_1-size_679566:204986..210308) *RAD50*

GTCAAGACTGGTGGAACCCAGGGCCAAGTTCAGAGTTTGAGGAAGCTTCCGATCCAATTAAGGTCAGAGAGGAGTATTGA

CCCAGTTATCTATTGAACTAACGCAACAATTCCATAGACGCAGAACAGTGCAGATCGCAAAAAAACAGGATCCAACAGAA

GCAGGCCAAAAGTAGAACACCGGGACTCGAGGGCAAACCCCCATACAAGCGGAATCAATAACTTTCAACAGCAATGGCGT

CCATCAACAAGCTTTCGGTCMGTGGGGTCCGATCCTTCTCTCCGGAAGACGCCGAGCAGGTCCTGGAGTTCTACATGCCC

CTCACCGTCATCGTGGGGGCCAACGGCTGCGGGAAAACCACCATCATCGAGTCCCTCAAGTTCGCGGTCTGCGGGGCACT

CCCCCCGGGGAAAACTGCGGGCCAGGCCTTTGTCCACGATCCCCGGAGCATCGGATCCTCGGTCGTCAAGTCGAACATCA

AGCTGCGCTTCAACAACGCGGCGGGGCACACCATGGTCATCGTCCGGTCCATGGAGGTCAAGCAGTCCAAGACGAAGCTC

AACTTCAAGCAGCTCGACGGGACGATCCGGGCCACCGACCAGCACGGCCAGAGGGTTTCGATGAGCCACAAGTGCACGGA

GCTCGATCGGCAGGTCCCGCTGATGCTGGGTGTTTCGAAGGCGATCCTCGAGAACGTCGTCTTCTGTCACCAGGAAGACG

CCTCCTGGCCGCTGCAAGAGGGTGCCGTCCTCAAGAAAAAATTCGACGACATCTTTGACTCGACGCGTTACACCAAGGCC

CTCGACGTCTTTGTCAAGGCAAAGAAGGAGTACATCCTCAAGGCCAAGGACCACAAGGCGGTGGTAGCGGAACTCCGATC

GCACCGGCACGCCGCCCAGGGATTCCGGGCGGAGATTCGAAAGCACAACGAACAGATGGAGGAGCTTGAGGACCAGCTGG

AAAGCGGCCGCCACGCCATCCGCGAAAACAAGGAAGAACAAAAGATGGTCGATGAGCTCCTCGATAAGATCAACGAAATC

GAGGATCGATTCGATATCAAGGAGCAGGAATACGTCAACGCAAAAGAGACGGCGCTCACCCGGCGGGGATTGCTTGAGGA

GGACCTCACCGATCGGTTCAGCGAGGATGAACTCAGGGACCAGCTCCGGGCCTTCGACGGGAAAAAGGAAGCACACCAGG

ACCGGAAGCGGGAACTAGAATCCCGGCAGAGGACCCTCAAGCGCGAGCTCGAGGCCATCCGGCACCAGCAATCGGAACTG

CAATCCGAGGTGGGCCGCCTCCAGGCCTCGAAGGAAGCCGATACCGAGAACCGAAAGAAGCGCTACGACAAAATGTGCGC

GATCGGTACCACTTACGGCCTGGAAGACGTGGTCTCGCAGATCACCCAGAATTCACAACTGGCCGCTGGTTCCACCCAAG

ACTCAAGCGGGTCCTACCATTCTCGGCACCACACACAGGGCGACGACGACGACTCGGCCGACGGCCGGGATATGGCGGCA

CCACCCGCCATTCTCGACATCCCCAAAAAGGACCTGGACGAGTACTTCCGGGTTGTTCGGAAGAAAAAAGACGAGCTGGA

GGACCAGGTGGCTGCCGAAAAGAGCAAGGCTCGGGAGCAAGAAGATGCCTTCAACAACGAACTGTCCGACCTCAAGGGGA

AACTAAGCTCCGTAGAGCTTCGAGCGGACGAGCTGCGCACGGCACAGCTCAAGCTGAACAACGATATAGCTGAGCGAACA

CGAAAAGCAAGGCAGGGTGAGCATCGAAGAAAAGCTCTCATTGCTTCCATTATGCCTCGGAATCCATTTCCATCCATGCT

CATCCATACTCCTTATCTGTTTCCTTCTTCAACCTCCACATTTATTCATTCTTTTCTTAATGATTTCCCTCGCCCCGGCA

GTTCCAAAAACTCGCAAAACCAATATTGATGAGGCGAGAGCGAACGCTGAAAAAGCAGCCAGAGCCAGAGACGAGGCCAA

CAATAACTCACGGCAAAAGGAAATTCCTATCGAAATCGGTAGTCTCAACAACAAGATCGAGGATCTCAAAAAGAAAATCG

AAATCGACAGGGCCGTACAGGAAGACCTCCGTAGTACATTCAAGGCGCAAAGCGCCATCGAACAAATGAAGGCAAAGTGC

AAGACTGACCTGGAAGAGCTCAAGATCAACGTTTCGGAGTACTCTTTTCTTTGCCAGTCCCAAAGGATCACGCCTCCGCC

CCGTGAACTTCCGGGAAATGACACAGACGGGAATGGCGAGGAGCTCAAGAGGATTATGGAAAAGGTCAACAACGAAATCA

ATAGCAAGAAGGAGGACAGAGAACTAGAGCTCGGGAGACAACAAGACGCGATCCATCGGCTCGAGGCAATTGTGTCGGAG

AAATCTGCTCTGCACAACCACTCCGTCCAATCCATCCGCCAGAAACAGAAGCGCCTTGGAGCACTACAACCAAGCATCGA

TGGGACGAAAAAGGTAGTCGAAGATCTTCGTTTCTTTGAGCAGCACGAACAAAAAATCCCGACCCCGGTTGCGATCACGG

ATGGCCGCCCTGATGAGCTTCTGTCCTACGTGACGAAACGCTTGGACGAGATCGAGGACAGATCCACGGAGAATATTCCA

CCAAAGATGGTTAAGCAAGTGCTTCAGCAAATATTCTACCTGGTAAGTTTGAGATTGAAAATCCTAGTGATGCTACTAGG

AAAGACTTGCTACCTTCGTTCTCATTTTTCTTCGACTTCATTCCTCTTTTCGAAAGTCAAAACCCGCCAAGGTCGGGGCT

GGTGATGACTTTGTTTGCCCTTGCTGTGAACGCAGTATGCCAGACGAGGCCGAATTCAAGAGGTTTCAGGACGCCATGAA

AAAATTATGGTCCGACGGATCCCCATTACTCCAGGTTGACGACTGGACTAAGACTGCCAAGGTAAGTCAACCGACAAGAA

ATAAATCGATTTGCATACGCTAGTATTTGCGTTCCTCATCGAGTTTTTCCCATGCCTTCTCGGTCAACAACAAAACTCTA

AAAATAGGCCAAGTATCAAGAATGGCAACGCCTTGTCAAGGGAGCGGTTGGTGATATTACGGAGCATCAGCGCCTGCAAA

AAGACCTGGTCGAGCTTCAAAAGAACGTTTCTGACTTGGATAGGATTCTCATAGACCGCAAGGGGGAGCGAGACAAAATA

AAATCAGACATTCAGAACGTCGAGTTAGATTTGAAAGAACTCCGTGAACTCTCCGACGCTTCGAAACGCTGGGTGGGAGA

TTCTTCGCGCATCGCTATGGACCGCATTCAAGTCAATGAAAAGGAGTCGAATTTCCGTCTGATGAATTCCGATAGAGAAG

GACGCGACCTGAAACAAGTTGAGGGAGAACTATCCGACTCGCTTCAAAGGAAAGACGAGTATGCTGGTACGTGTTTAGTC

CTATTTTTGAGATGGAAATCAAATGGCAATTACTAAGTCCGGAAGAAGTCGCACACTCTCATCGTTCGGTTTTCCACCGC

TTCTCTTACTTATGTACTCATATACGAGTAGATAAGATCACAAAACTTAACACTGAGCTAGCTTCTCTCAATGACCGCGT

ACAACAAGCATCCACTAATGCAACTCGTCTCGAGCATCTATTCAAAAGAATGGAAGAGAAATACCAAGAGCAAATCAAAG

ATGAAGAAACCTTAGCTGTGTTTCAGACAGAGTACTTGAAGAAAAGAGCCGAACAGGAGAAGGTACGTCGAACAGTTCTG

TTCTTTTAAAAATTAGAATTTTTACGCGATTCACAGTGCATATAATGCAGACTGAAATTGCTTCTTTTGTCCACTTGTCT

GGATGTATCTTCGCTTCACGCAGCTCTCCGACCAGATCAAGCCATTGAAACAACGTATCGAAAAGAAAGAAAGTGACCGG

ACTCGAGCTCGAAATCTGGCCGCAGAGGAAGAAAGGCGAATAAGCAGCACCCTGGCTTTATTCAATAATGACATTGCAGG

ATTACAAAACATTGTTGATGCAATCAATGAATACGAAAAGTCAAATTCTAAGGACGATGTCGATCGAATGGAAGCTCAGA

GAAACGAGTTGATTCGAAAAGCCAAAGAAAAAGATGAAGAGATCGAGAAAATGACTCCTGATCTGAATCGCATGGAACGA

ATGGTTGATGACCAGGAACGTCAGCGCAAAAACCTTGAAGAGAACATCAATTTGATCCGGTCCACGGTAAAAATCAAAGC

ATTAGAAAAAGAAATTGGCAGGATAGAAAAAGAAAAGTCAAGCGTAGGTGACCGCCAAAGATTGCAAGAAGATCATGAAA

GTCTTCAAAATCGGAACGAGCAATTGATTGAAAGCAACGCACGATTGGAAGGTAGACGCGCGGAGATTAATGACACAATT

CGTGGATTGAAAAGAAAGTTGTCGCAAAATGAGTATAGAGATGTCGAAGAAGATTTCAGGAAAGCTTCGATCAGACAAGA

TACTACAGAAATGGCCGTCAAGGACATTGAAAAATATCACTCGAATCTTGACAAAGCTTTGCAACGCTACCACGGCATCA

AGATTACAGAAATCAACGCAATTATTAGGGATCTTTGGAACCTTACGTATAAGGGTGAGGATATTACCAACATTGAAATT

ATTTCTGGAAATGACTCTGCTGGTCGAACCACTCGCTCATACAACTACCGAGTTGTCATGTCGAAAGGTGGAGGAAGTCA

AATGGATATGAGAGGTAGGTGCAGTGCTGGACAGCGTGTTCTTGCAAGCATCGTCATTCGTTTGGCGCTCGCGGAAACAT

TTTGTGTGAAATTCGGGTGCATTTCACTGGATGAGCCCACGGTGAATCTGGATTACAACAACAAAAAGGGGCTCGCCGTC

GCCCTCGCTCAGATTATCGCATCCAGATCGCAGCAAAGTAATTTTCAGTTGGTTCTGATTACTCACGACGAAGACTTTGT

CTTGATGATGAAAAACGAGTTGTCAACACAATCAAACGTAAGCATGCCCGAGAAATACTTTCAGATCAGGCGAGAAGAAG

GAGTCGACGGGAAATACTACTCGAAGATTGATGCGATTGATTGGGAAGAATTGCTTTAACGGTTTTTGGACGAGTACCGA

GTGCCAACAAACAGAGAAAACTCGCTTGTTCTTGCATCAGTTTCTCCAGCGCCTTGAGGCTACCTCTACTAGATATCCAA

AGTACTATGTTTTACTCCGTTTCGTTCACAGAATCCGACGTATTTGAAATCAATTCCAAAAACATAGAGAAATATCTATT

TGAGAGTAGAATTTTTAATCTTCAATTAACGTTTTAATGTTCC

>PSNMU-V1.4_AUG-EV-PASAV3_0086370.1 class=Sequence position=[PsnmuV1.4_scaffold_44-size_221303:113260..117015 (- strand)](http://gbrowse255.tgac.ac.uk/cgi-bin/gb2/gbrowse/maplesod_psnmu_v1_4_gbrowse255?name=PsnmuV1.4_scaffold_44-size_221303:113260..117015) *MRE11*

ATTCCATCACCTTCGATTGCACTACCGTAGCAATATTACACGGTGAACACGGAGCAGCAGCAAACAGGTAACAAAACAAT

AACGAGTTGCCACTCGCCGAGATCCAAGCAAGACTCAGTCGAGTGTTGTGGTTGAAATCCAAGCAAGTCAGAGTCGCGTG

CTGTTGTCGAAATCCAAGGACGACAGATATTGATTGTCAATGCTGAATTGAACTGAATTTCTCCCCGTTCAAATCCTTCG

TTCCCACCGTTATTACCGGTTTGGGCAGGTGCTCCTGGTTCAGTCGATTGTAAATTTTTGATTTTTGATTTGCCCGTTTG

TGGATCGATCGATTCTGATCGACCCTTTCGGTTCGCGTACACCATGCGTAATCCTTCATCCTCAGACGACGAGGCGGTGG

GCCCTTTGTCGTCCACCGGCGGGGCAACAGGAGGCACAGCGTCAACTGCTGCCACCAGACGGGGAAGGCGACCGGCTGGA

GGAACCCTCCGTCGCCGCCGAGGCGAAGTGGAAGTCGATTCGAGCGACGAGGACAGCRCCGGAAGCCAGGACCTCCGTGA

AAACTTCGATCGTCGAAGAAACTCTGATTCGAGATCGAGTTTGAGCTCCAGTCTGAATACGACTTTGAATTCGAATTCTC

AATCCTCGAGGCAGCGGGCACCGGCTTCAAGCGACAAAAATACTTCCGGGAACGCTGGAAACGCGGATGAGAGTCTCAGT

GGTGACTACGGTTCCTCTCCATCCGGCTACAACGGAAGAGAACCATCTTCGGCAGCGCCGTCGCASTCGCAACGAGACGT

CATTCTAGAGGACGCAGACTCGAACACGCTGCGGATCATGATCAGCACAGACAACCACGTGGGATACGCCGAAAACGACA

ACATTCGGGGGAACGATTCGTTCGCTGCTCTCGAAGAGGTCCTGTACCTCGCAAAAGAATACGGATGTGACATGGTGCTC

TTGGCAGGGGATTTGTTCCATGAAAATCGTCCGAGTCGAAGGACACTGATCAAAACAAGTGAGTGGGWATCTAATTGATA

TMAAAAAATTGTTGCCCTTGGGCTGGCTCCRTTTCTTTGGTTCCAGAAACGAAACCTGTGGCGATTTTATTGCTTTCTCT

TATTTTCGTTCTTTATTTCTCCGCAAAYATCTTTTTGGTTTCTCAATTGTGGACGAATCGCACATTTCCAGTGGATATAT

TTCGTCGGTACTGCATGGGCCCTGGAGCAATCCAGGTGCAGGTTGTTCCTGCGTCAAGAAAAGGAAGCAATGGCAACAGC

AAGAGCAACGAGAACAAAAGTCCTTTCGTCCGAGGCTACGTCAACTACGAAGATGCCAATTATTCCGTCGATTTGCCTGT

CTTTTCGATTCACGGGAATCATGACGATCCGAGCCGTGATGGCGGTAACTCGGAGTTGTATGCTGCACTCGATCTGTTGG

ATGTTGCRAACCTCGTCAATTATTTCGGCAAGCAGGAGGACACACAGAGGATTCGGATCGATCCCGTTCTGTTGCGAAAG

GGAAACACTAAGGTCGCACTGTACGGACTAGGCAGCATGCGCGACGAACGACTCAACMGAATGTGGCGGGGGGAAAACCT

GACCTTTACGCGCCCCGCGGAGAGCACCAGCGACGAGAACGTTCCGGCATGGTTCAACATTTTTGCYCTGCACCAAAACC

GCGACCGCGGCCGGGGAGCCAAGAATTGCATCAAGGAAGACATGATCCCGGACTGGATGGACTTTGTCTGCTGGGGCCAC

GAGCACGAGTGCGACATCGAGCTCGAGGAATCCGTTGTCGGGACCTTTCGCATTAGCCAGCCGGGATCGAGTGTAGCGAC

GTCGCTCGTKGAAGGGGAATCCGTCCGCAAGAAAGTTGCGGTGTTTGATGTTCGTGAGGGGCAGTTCCGGTTGACGGCGA

TCCCGCTGACACAGGTCCGCTCCTTTGTGGTCTCGACAGTCCGGTTGGCGGAGGAGCGCCGGCTCGATCCAGACGATCCC

AAGGTCGACACCAAAGTCTCYAAGATCTTGGAAGACAAGGTACGAATTTTRATCCACGATGCAAGAGAAAAACGAAGGGA

GCTCTTGGAAGACGCCAAGAACGGGGGCAACGGCTTGGCGAGGTACTGTGCAGCCGCTGGTGGCAACCCCGATGRCATGC

CACTGAAACACACCATGAACAAGGAGGACGAAGTCCTGGTTCGCCTGAAGGTCGATCACACGGGTTTTGCAGCGGTGAAC

AACCAGCGGTTCGGGGCAAGATTTGTTGGGGACGTTGCCAATCCGGTGAGWACAGTATTAGTTGGTGTGTTTTGAGTTCT

TTCTCGTGGAAATTATCCGAACCGAAAGTCCCGTTCTTTGCAAAAAATCGAAATCTCTCACACTCCACGTTTCCTGCCCC

GAATGCTGCAGACTGACATTTTGTTGTTCACCCGTAAAAAGGCAGAAGGCGGTRGATCGCGCTCGAGCAAGTCGATGAAG

GGCATTGCCCCAATCGAGCCTAGCGAGATCGAAGAAATGAACATCGAGGATTTGATTGTCGAGCAGTTCGAATCCAGCAA

CACCAAACTCGAACTCTTTGACGACAAGAAGATTTCTGCCGCCCTAGATTCTTACGTTGGAAAACAAGAGGCGCAGGCCA

TCAATGAAACGCTCGAGAAGCTCCTGGGCAAACAACAGAATCGATTGATTAAAAACGGGGCATTGTCGGTCGCGGAGGGA

ATAGAAATGGATGGTGATGRAGAAGTTGGTAGAAGGAACAACGGCAAGAGACGCAACAAACGTGGAMGAGACGACGAAGA

GGACGAGRACGATGCCATGCAGGATTCACCACCCACCAGAAGTCGATCCAACAAATCGTCCCAGAGATCCATAAGGTCGA

GATCGACGAGAAAACGGGCGGATTCTTACGATAGCTTGGACGATGGGGATTCCATGCAGGAGTCACCGCCCACCAGGTCG

CGATCCAACAAATCGTCCCAACGATCCACCAAGTCTAGATCGACAGGCTATGACGACGACGACAGCTACGAAGCAGCGCC

TTCCCAAGTCAGGTCCAAATCGTCCAGAACCACTTCGTCTCGATCAACCGGTCGCAAGGCTGCTGYGCGCTATGAAGAAA

GCGAAGACGAGTACGACGACGACGTCCGGGTGGTCGACCCACCACCGCGCAGCCGCAACGCCAAGCCCGCCAGACAACGC

GCAACGAGGAAAACYATAGACTACTCGGTCGACGACAGCGAAGACGACAAGGTCGACGATTCCCTATCGATCGACGATCC

CACTCCGCCGAGAAAAAAACCCACGGGACGGGGCAGAGCTKCCGCGAAACCGACGAGAAAGACCGCCACTAGTAGGAGAG

ACAGACGCAGCACCGACACGGCTAGCAGTGGGTTTTCCCAGTCTCAGCTGTCCTTTCAGCCCATCAAGCGCAAGACAGSC

ACAACACGAGCCAAGAGACCCAGGCAMAGCTACGACAGCGACGATGACGACGACATGGGTCCAGCGGGACGTAGCTATGA

TGACGATGACTGGGGGACCGCGAAATCCGGTCGGTGAACCGAATCATGGTGCATGTAGCGATTCCACGACCGGTTTCAAG

GATGCTCAAAACGTCGTCTTSGAAACGAGCAACATCACTTGCTATGTTGCCAGCAGTATAATTGCACGAAACAAAGAGAT

CACGACACCATATTGTTGAGTGTTCATCGAAAAGAAAACCTTCAATTTACAATTCAAATAGAGCAAAAAGCTATTG

>PSNMU-V1.4_AUG-EV-PASAV3_0087420.1 class=Sequence position=[PsnmuV1.4_scaffold_45-size_270500:150591..155625 (- strand)](http://gbrowse255.tgac.ac.uk/cgi-bin/gb2/gbrowse/maplesod_psnmu_v1_4_gbrowse255?name=PsnmuV1.4_scaffold_45-size_270500:150591..155625) *MER3*

CGACTACGAAGTGGGTTTCAGAACCGAGCGCGCGTGCATCAACGAATGGCTCTTTTTTGAAGCGGAAACAAGAAGCTTCA

AACTTCAGATTTTGAGTTCCCGACAAGGCTGAAATCTTGCTCACGCCGATCTGGCTCGATGCGACGTCGAACGATCCTTT

TCCATCTTGCTTCTCGTTTCCCCCCACGTTACCACYTAGCTTTCCTCTTGTCAGCGTTGCTACAACATTCGCTTCCGCAC

TCTTGGAATCATTGAATTATGGCGGAATGGTACGAAGAAAACGAAGACGAAGCCGCTTCTTACTTCAGTGACGATTCGTC

CACTGAAGAAGAGCTGCTTCGCGTAGCAGAAAAGCTCGAGACGATGCACAAGGAAAAAGAGCGAAACAAGAAAGAAGCAA

AGAAAGACGAGGCCATGATCCGAGATATCGACGACCGCGAGGCATCTCAATGGTCGGTTTCTGAACCTCGTCGTCTCTGC

CGTTCCCAGTCGATCACGCGGCCGGATCAATCGCCGGAGTCCGATTCTTTGTCCTTTCCCGCAAACACCGAGTCCCATTT

GCACAAGGAAAGTCCAGCTGCCAGCAGCCCAACCAATGCAAGCATTCACGGCTCGGATAGTGACACGCGCGTCGGAACGA

AAAGTACGGTCGACCCCGATCGAAACGGCGGTGGGGGCGATGGAAAGCGATCGCTGGCCTCGCGAAGCGATTGTAACGAC

GGGAAMAGCAGTAGATGGCAATTTCAAGCATTGCGGCCCCCGCAATCCGGTCGGAAAAGCAGCCATGAACTGGTGGTTTC

GAACCCAGGACCTTGTAATGGTGCATCGCGAAGCKATAGGTTTGTCCGCAGGGGTGAATCGGGAACGAAGAAGGACGAGC

AAGATATGCTGGGGAAACGGCAAGAATATGCTTTGCCTCATCCATCTCGACAGCAACAATACGAGAGCATCCATGAGACC

AACACGAACGGCAATGGCGATGACGAAAAATCCATCGCTAGGAATCATCGTCGGGCTCATGACGATGAACCGACGAAGAA

GGAGGGTATCGTGGGTCTCACCCGGAAAGAGACAAACGATTCTACGGATAGCAGTAGCAGTAATAGCAGCAGTAGCAGTA

GCAATAACAGCCGCAATGGTGAATGTGAGACTGATCTCAGCGACGACGATTCTACTTCAATTTCCTCGACTTTGATTTCC

ATCGACAAGGCATCTCTGATAGTAGGGAAAGTTACGACACAAACAAAAAAGATAGCGAACATGGATGTTGACACCCGTCG

TTCGAAACATTCGTCGGAATCGGAAACCCCGTCTACAACTTCTACCATGTCTTCCTCGTCTCCCCCTTGGTCTCAGAACA

GTTTTGGAGCCACACATACTTCGAATGGAGCTGTTCCGAACGATCCTAACTACGAGAGAAAGACGACTGCCAAAAAATTC

AAATCTGATAGGGGAGTTACGAATCCTTTGGGAAGGAGTACCCTATTGCAAAGATCCACACCTACTCTTTTGCGAAATCC

ATACCTCCCTAATGCCATTGCAGATCCAAACAAAACACGAGAATCTTTGTCATTGGAGAAGATGGGGAATAATAATCAAC

AAAGTCAATCGATATTGAAAAAAAGGAAGGAGTTAAAGAGATTCGACGATGGTCATTGCGATGGCCTCCCTATTGATATT

ACCATCAGCGATCAGAAAGATGAAGGAGAACAATCGAAACCGCTCACTTCAAAAGGGGCCGAATCGTCGGCCTCGATGGA

GCAGAAGACTCGGCACTCCGTTCCAAATGCTGAAATGATGAAGGTATTCGAAGGTTTCGAAGCCAAGGGCGATACCAACG

ATAGCTGCGAGACAGTTGTCGTCACCGATGATCATCGAGACGAGCCCGAAGAACCAGAGGTGTCGCATGTCCTCTATGCT

CCACCCGTTTCTAGAGACGGCCGGCACACAGTTCCAAACGTACAGAAATTCGATTGTCGCAGCCAGCCCCTCCATTCCAG

AAAAACCATCCCAGTCCGAACCTCCTATCGTTTCTTCACGCCTCCGATTGCCAAATCAATGTGGCCAAAGTTTGATCGAT

TCAATCATTTTCAGTCCGCAATGCTCGATGTATTGTCCAATTCCGACGACAGCGTAGTCGTGTCCGCACCAACGGGAGCC

GGGAAGAGCACGATTTTTGAAATGGCGGTGGCACGGTTTCTAGCCATGGACCTCGAAGCCCAGGAGCAGCAGGTGCACAG

CAATGGATCCAGCTCGAATCCACCTGTTCGGTCGCGATCGCAAGAAATCTCGAAAGCGCGAAAGATCGTTTACATCGCTC

CGAGCAAAGCCCTTTGCCAGGAACGATACGAAGATTGGTCGCGAAAGCTTCAACAGATGAATTTAGGGCTGGAGGTGGCT

TTGATCACGGGGCAAGATGCAGACAACGAGCACGCCAACAATGCGTTAGCTGACTTAATCGGCGGTCACCTCATCGTGAC

GACGCCCGAAAAGTGGGATTCGATGACCCGGCGGTGGAACGAAAAGTTCTTTCTCTTTGCTTCCGTGAAGCTTCTTCTGC

TGGACGAAGTGCATCTTCTCGGTGACGAAAACCGAGGATGGTGTCTGGAGTCTGTTATAACCCGGATGAAAACCATTCAC

CGCGCTGCCACAGTGCTCCACACCGCCCCCCAAGTGATCCGTACCTCCAGCTACCCCGAAACGAACCCAAACGCAATCAA

ATCAAGCTTTCGAATGGTGGCCGTATCAGCAACCCTCCCAAACATCGCGGATGTTGCAGATTTCTTGCAAGCTAGAGAAG

CATACACCTTCGATGACAGCTATCGGCCAGTTTCTCTCACCAAGCACGTGAACGCAATGGGAAGAGTAGGGAAGAACGAA

TACAGGTTCTGGAACACTCTTATCGACCACGTTCCTGAAATCATACGAAGATTTTCTCACGGAAAGCAATCCCTGATTTT

TTGTCACTCCAAAAACGAAACCGAACGACTAACGGAGTTACTCATTCAAAAAAAGCTTGGGAACGAAGCTAATGTAGTGG

MCGGTCGACGTTGGAGTGAACCGGTCGGCAGAATGCTTGCCCATGGCATTGCATTTCACCATGCAGGATTGAGCAAGACC

GAGCGCGAGAGAATTGAAGAGGCGTTCCTCAACAAAACTATCAAGTGCCTGACCGCTACATCCACGCTCGCTGTAGGAGT

AAATCTTCCTGGTATGTTGCGTCGGAATGCTTGCTAACCTTGATTCTTTGATATCCCATATAATCCATTACCTAACTTCT

GTCTTGCTCGTCGTCAGCTCATTTGGTGGTGGTTGTGGGAACAAAGGCATATCGTAGAACCAAAATAGGCAAGGGGTCGA

AGGCCGGCTACGAAGATATTGAAGTTAGTACACTGCTACAAATGGTTGGACGCGCAGGTCGGCCRGGATTGGATTCCACA

GGCGTTGCAGTTATTTTGACGGATGTCGATTCGAAGAAAAAGATTGAAAACATCATGCAGGCAGGAATTGGGCCGGCTAA

ATCAAAACTTGTCCCAAGATTACCCGAGGTGATAAACTCGGAAGTATCGCAACGAGTCATCACCTCGAAAGAGGGGGCGA

TGCGTTGGTTACAAACAACATTTCTTTTCTCTTGTATGAAACACGACAGAGATGCTGTTTCTTCTGCTCAAAACTTGACC

AACGAAACCTTGGTGTGTCTGCGAGAGATTGGCTTGGTAGAGGACGAAAAGACGGGTTTAAAACCCCAGGCAGGATCTTT

CATAATGAACAAACGCCTTATTTCTTTTGAGGACATGAAAGCAATATTTGCGTTCCCATTTGATGTGACCCAGTGTCAAG

TGCTCAAGTCCATATCAAAGCTGAAAAGCCTCCAAAGTCACGTGAGGAACGACCAAAAGAGGCAGCTCAAAGAGTTTCAC

AAAACTGAACTCATGAAATACAAGTTACCAGGGCGGTTGTCAGAATTCAGAGTCAAGGACGAAAGTGAAAAGGCTTTCAT

TCTTCTTCAGAGCTACGTATCACGACATCGATTCGCAAACAAATTGCTAGATGAGGAGCAAATTACAGTTCGCAACGAGG

CCATCAAATTTTTGGAAGCGGCACAAGAATACAGTCTGAAAGCATCGAAGCACGGAAAGGTAGCATTTGAATGTCACAGA

CTCCAAAGATCGCTTGTCACATGCCTTTGGGGTGAATCTTCGGGCGTGTTCAACCAGTTCAGCTGGATAGGTTCTTCCGC

AACCGGTTCAAATACGCTAGCATTTGGTGGGATAAAAACTTTTCAAGATGCGTTCGATTCTTCGGAACAAAAGCTGAACG

ATCTTTTCTCGAAAGCCAAGATCTTGTGGTTGCCAGAAAATCCTGGCCGCGTAGTGAAGCATACAGTCAATGATTTATGT

CGTCGAAGGCTCAGGTTATCTGCAAAGATTGAGTACACCAGGAACTCAAACTTACCAACATATCTACGTTGTACTTTGAA

GTACAACGACCCAACTCTGACTATAACAAGAAATGAAGGAGGATTGCAGGACTTCAAGTTTAGTCTCATGGCATACACAG

ATAATTCAAAGCAGTCGTGCTTAATATTTGAAGATGATGTTTACTCTCCATCATCCTTATCTGTGCCGTTGCCTTCGTAT

ACGTTTAAAAAAATTTACGTTCATTTGATGGGTACATGGATAGGATTTGATGAAACTCAAATCATTCATGCAAGAAAAGG

CTGTATGAATGCTGCAAAGGCAGATAATTCCGCGGCGCGGCGTCCCCAGATCGAGAACGAAGAAGACACTACGACTACTA

AGCGCAAGATTCGTCAAACAGAGAGATTGATGTACCCGAATCCGCAGCCCCAAAAAAGACAAGATGAAACAACAAAGAAA

TCATCTAAAGAACCATCTCCAATAACACCTCCGAATGACTATCTAAGATCTATACCTAACCAGCAGTCTTTGCAAGAGTT

TTGGAAGGAATCGAACAGGAATGGCCAAGCCGTTCATCAATCTCGGGCAGCTGACAATATAGTTCTGTCAAACCG

>PSNMU-V1.4_AUG-EV-PASAV3_0080640.1 class=Sequence position=[PsnmuV1.4_scaffold_40-size_264869:241826..244420 (+ strand)](http://gbrowse255.tgac.ac.uk/cgi-bin/gb2/gbrowse/maplesod_psnmu_v1_4_gbrowse255?name=PsnmuV1.4_scaffold_40-size_264869:241826..244420)*MND1*

TCCACAACGCACTGATCGACAAATTGTAACATAGCCACTTAAAGAAGAAACCAGGAAGAATCGTCAAGATGGGTGGAACG

AAACGAATGTCGGCCGAGGAGAAGCGCAAGGTGATCTTGGGGTAAGCGCCACGAGACTGCACAGCTGCGTGCCCTATTGG

TTCGTCTTCTTCTTTTGTTCGCAGTCCTCACCCATGGACGATTGATTTCGCCTGTTCCTCCATACTTTCACTCTTCTTCC

TTTCGCAACGCAGTATTTACCACAAGGAGCAACTGGTCTACACCGAGAAGGAAATCACATCGCTGGCGGCCAAGGCGGGC

GTCAACGCCAACTCGATCCCGGACATCCACCAGGGAATGATCGACGACGCCCTGGTCGAAAAGACCAAGATCGGCGGAAG

CAACTATTTCTGGAGCTTCAAGGCCAAGAAAGACCGGGCGGCGCAGGTCAGATACGAAACTACGCTGAAAGCCATAGAGG

AACTCAAGCCCAGGGTGGCCGAGGCCGAAGCCAGGCTTGCGGACGCGAAGCGCGGTCGCGAGGAAGACGACGACGGTGAC

GGAAGGGAAAGCGGATCCTCTGAGAAGGATGGCGGCGACGAAGGATCCGGAGCGGTTCCCGTGCGTGGACGAGCCAAGAA

ACTCGCACGCCTGGAAGAGCTTGGCAAGGAAAAGGCCGCCCTCCAGACGGAACTCGACAAGCTCAAGGAAAACGATCCAG

CAACCCTCGCAGACCTCGAAAAGGAACTCAAGCTTGTCACACAGGTGCGAATAAACGAAGGGACATGCACAAATTTACAG

GAAGCAAGCATGTTGCATCATTTTGTTTCCGTTTGCATCCCTCTGCATGAAGCATGTCTTTCCTTCCCGTCTCTCACACG

GTGTACCCCGCGTGCCAAAATTGCTTCTTCTCTATTCCATTCTTTTTGACAATGAATGAATCAAATATGCATATGGCGAT

TGTTATTCAGGCCGCCCATCGCTGGACGGACAATATTTTCGAGTGCAAGAGCTACCTTGTGAAGAAACGTGGAATGCAGA

AAAAGGAGGCGTGCAAGTTTCTTCAAATCTCTTCCGACTTCGATTGTGAGTGCCATGGCTTGTCTAATATTATTATATTG

TGTTTTAACCGTTGATGACAAACTTGATGGACAATGTTTTCCGTTTCTATGGTCAACCAACAATGCATCTTCTTCATTTC

AATCTTGTTGCTTCTCAACAGATCCCGAGGAGACGAATTAGCAATTTTTTCGATGGCGCTATGAATATGGAAATATATGG

GAACGTCCCAAAATGCATTCCGTCGCTTGCAAGCGAGGATTGTGGGATATCGCAGTGCAGTCAGAGTCCTCATCGCCGCC

AACGGCAACACCATTCCCAATTCTTTTGTGAAACAGCAATGAGCACGAAACAGAACTCCTGGTGGAGAGATATGCCACTG

TTGAAGGATGCGATATCGCCTCCCACCGCGTTCTAGAACTTATTTGAATGATGGTTGTTGTAATACACATTGATAAGATT

GACGACATCGGAATTTTTCTACTCGTCAGACAGTCGTTGATGCTTGAAGAAAAGAAGACTGATTTGCATGAACATATCAA

TTACACGATAACATTAGGTGTCCAACTCTCAGGTTGGGACCAACATCTGTACTTGCGATCACCCAAGATCCTATGAAAAA

ATACTCTCCGCAAAGAAACATATATTTTTTCTGTAAAATAATAATGCCTGTAATGGGCGCAAACAATTTTTTCAACTGAA

TCAAGATACTAACAAGATCTTATTGCACGATTGATCCCTTTGCAAATGATTAGTTTTTCTGTGATTTATTTCGATCCACT

CGCACCAACTATTTTTAATACTTTGAACACGCCGTGCCATGCTAAGGTTTGTATGGATAGAAAGCTGATTGCATAATAGT

GATTGCATTCAACTCCAGCCTGCTTGTGGATAAGTGGCCAACTTCAAATTATAGCGTTTTGTACAGTTTTGAAAGACATG

TCACACCTGAAGATGCTTCGGGGAAACTTTCATATCCAGGTTCGAGTCAAAATCCATACCATCTCCTCAAGTTCGAGTAT

GGGTTTGTCCGTATGACGGATTGATCAGTATGTCTAGAAACAGGCAGTGCGACAAGATCAACTCTGGAAAGGATGTCGCG

TCAAAGTACAAGTATTGATAGAGGCCAACAAGCTGCTGCCAGGACCAATCTGCGTTCAATCTTGAAAACCAGCGCTCGCC

ATGTGACACATCCGAAAATAGATCGATCGATCCACTTTGCTTAGCTTTTCACTTTACTCAACCATCTATCCCATCAAAGA

GTCCAGAAATAATCATCAAGGAGAAATAAGGAAGCAAAGATGGTCCTGCGAAAGTTCTGCTGCCCACACACCAACACTCG

GTTCGGTTCTATCGTCTCTCCTTCTATTGCTGTGGCCGTTGCTGCTCGTTCTGGTGGCACTTGCGAAAGACCTCCCACTC

GCTTGCACAGGAGGAAACCCTACCACCGTTCTTGGTCATGCAGGCGGCAACGCAATCATACACCTCGGCGCAGGGCCGGC

CCGGTTGTGACACCGGATCGCTCGCAGGCAGGTCC

>PSNMU-V1.4_AUG-EV-PASAV3_0116300.1 class=Sequence position=[PsnmuV1.4_scaffold_8-size_368192:244046..249880 (+ strand)](http://gbrowse255.tgac.ac.uk/cgi-bin/gb2/gbrowse/maplesod_psnmu_v1_4_gbrowse255?name=PsnmuV1.4_scaffold_8-size_368192:244046..249880) *MSH4* wrong gene model (Ubiquitin-activating enzyme E1 with MSH4 domain)

CTCCCGATCAGAAGCAGAAGCAAAAGCAAAAGCAAAAGCACAACACAGGACACAAAACGGCTCGTTGTCTCTCCGACCGG

CAACGAAACCCAGAGCAATCATTGCCATGCCGAAGCCAACACCAACGAACACGCGGGGAGGCATCAAGCGGCAGCGATGC

CCGAGCGGCGGACCACGGACACCGGCCCTCCGCCTTTCCCCTTCGATCCTCCTCTTCCTTTCCGCGGTATGGCTGGCGGC

GGCCTCCGCCGGGGCCAGGCGGGCGCCCTGGTCGCTGGCCCCGCTGCCGTCGTCGTCGTCGTCGTCGTCGTGGGGAAAGA

ACCGAGCCACGCCACTGGCGCTGAGCGCCCGCGGCGGGGGCGGTGACCGGACGGAACCAAGCGCATCCAAAACCAGCAGC

AACCATTGCAACGAGGACGACGAAGAGGACGACGAGGAGCGGTACAGCCGGCAGGTCTACACGATGGGGGCGCGGGCGCA

CGGGCTGGTCCGGTCGTCCAGGGTGTACCTCGACGGGCCCGCCCGCTCGGGGCTCCTGTGGGAATCCGCCAAGAACCTGG

CGCTCTCGGGGGTGGGGAGCCTCGTGGTGGTGGTGGACGACGAGAACGACAGCGACGATGCCCGCAACGATGCCAGCGCA

CCGTGCCCGCACTACCACGATCCGGCCCTCGACGACCTCGGGAGGACCTACCTCCGCGGGGCCCTGGCAGAGCTCGGGGA

CCTTGCGGCGGAGGACCCTTCCGCCGTCCTGGTCGGCTTTCTCCGGCGCCTCAACCCGGCACTGGCGGTGTCCACGATCT

CCCGCAGGAAGCTGCTCTCCGGCGAGGACGAACACGAAGAGGAAGAACGAGGAGAAGGGGTCCTCCTGTGCGTCGATCGC

CCCTGCCAGGAGCAGGCCGAATGGAACGAGGCCTGCCGGAAAGCCGGCCTCGCCTTTGTGGCGGTGGAAACCGCCGGGGT

CTACGGCCGCGTCTTTTGCGACTTCGGCCCGACCCACGCGGTCCACGACGCGGACGGCGAGGCCCCCCTGGTCGTCCCGC

TGGACCGCGTCGAGGCCCTGCCGCCAGAGGAAGGAAGTGGCGAAGAAGCAAGGACGGCGACGACGACGACGACGACGATC

CTGGTCCGGAGCGTCGAGGGGGAACACCACGACGTCTCGGGGGGCGACACCGTCGTCTTTTTGCGGTCCGACGGATCCGT

CCTCGAGGACTGCCGCGGGACGGTCGCCCGGGTCGAAACCCCCGAGCGCATCCGGGTTCGGATCGACGCCACCGAAACCC

AAAACAGCGAAACCGAAACCATCGACGCGATCAACGCGGAAGCCGTCGCCTTTAGCCGGGAGAAACAGATCGAGGAAATC

TCCTTCGAGCCCCTCGCCAAAGCCGTCGGCGAGGCCACCGACAAAGGGACCGCCCACRCCGTCTTCACCCCCTGCGACCT

CGACAAGTCCTTCGACGAGACCCGCCGGGGGGCCGTCTTTGGGTGCTTCCAGGCCCTCGGCTCGTACGCGGAAACCCACG

GTCGCATGCCCGTGCCGGGAGAGGAGGACGAGCCGGCCTTTCGGGAGCTGGCCACGGAGGCCGCCGGCGAGTRCGGAAGC

GAAAGCGAAAGCGATGACTGGAAGAAGCACTGCGCGACCTTTCTGAAAACCTGCCCCGCCAAGTTCGTCCCACTCCAGGC

CATCTTTGGGGCGATCGCCTCGCARGAATGCCTCAAGGCCGTGTCGGGCCTCTACAACCCCGTCCGGCAGTTTCTCYTGT

ACGACTGCGACGAGATTCTGGACACGACCGAAACGAAGAAARCGAAAAAGACCGCTGCCGAAGCGRCGTCGGAAGACGAC

GAAGAAACCACCGGCCTGTCCTACCTCCTCGGACCAGAGGTTGCCCGCCGCCTGCGGGCCCAGAAGCTCTTTGTCGTGGG

GTCCGGCGCAATCGGGTGCGAGATCCTCAAGAACGYCGCCGCCATGGGACTCGGCACCTTTGGATCCGGGAGCCTCGTGG

TCACGGACATGGACACGATCGAGAAATCCAACCTGAGCCGGCAGCTCCTCTTCCGGGACGAGGACATCGGAAAGTTCAAG

AGCAGGGCCGCCGAGGAGGCCGTCCTCCGGATGAACCCGTCGGTGAGGGTGGAATCCCACACGAGCAGGGTCGGGGACGG

GGAGGATCCGGGTCCCTTCGATCCGGCCTTCTGGTCCGGAGGGGTCGACGTGATCCTGAACGCCCTCGACAACATCGAGG

CCCGCCTCTTCATGGACGGYCAGTGCGTGGCAAACAAAAAGGCCCTGGTCGACGCCGGGACCCTCGGATCGAAGGGGAAC

GTCCAGGTGGTGGTTCCACACCAGAGCGAATCCTACGGGGCAAGCGCGGACCCCCCGGAACCGGCGATCCCGGTGTGCAC

CCTCAAGAACTTTCCCTACGCCATCTCGCACACGATCCAGTGGGGCCGGGACCTCTTCGACGGCCTCTTTGTGCGGAGGC

CCGTGCAGGCSAACCAGTACGCGCMGCTGTTCGCAGGCTCGGGCACCGGGGGGCTGGCCGGTGCGCTGGACGAGGAGCTG

GGCGACGAGGCTGCCCTGGAAGCCGCCAGGGAGCTCGCGGAGGACCTGGCGGTGCTGCACGGAGAGGACAACGGAGCCGG

CAACGAAGCYCTCCTCGAGCAAAAGGCCATCGAGTGGGCGGTTGCGCTGGGGAAAAAATTGTTTCAAACCGCCATCGAGG

AACTCCTTCTGGAACACCCCCTCGACAAGCTGGACGAAGACGGAGAACGCTTCTGGTCTGGATCGAGAAAGCCGCCGCAA

CCGCTGTCGTTCTCGCTGGACCCAGAGGAACAGGCGGCCGAGGGCGCCGCATCCCAAAGAGAAGAGATCAACAAGAACAT

GATCGACTTTGTTCGAAGCGCTTCCCGGCTTCGGTACGAGACGTACGCCGGGATTCCATCGGATTCCCGGGAAAACGCCG

GGATCGTTTCGCGCGAGACAGCAATGSAGGCCCTCGCTCATGCCACCGAGAACGAGAACGCTGCCCGAAAGGAAGAGGAC

TCCGAAACAACCAAGCGATCGAAAATCCAGGGTCTTTTGTCCCCGCTCGAAGGGCTTTCCTCGAGTGACCAAGCGGCACC

ACGATCCCTGTCCCCGGCCGAGTTTGAAAAGGACGACGAGTCGAACGACCACATTGCTTTCATYACCGCTGCTAGCAATC

TCCGTGCCATCTGCTACGGAATCGCCCCGGTAGACGCCATGGAAACCCGCAAGGTGGCGGGRAAAATCGTGCCAGCGATG

ATCACGACCACCGCCTTTGTATCGGCTCTTTCGTGCATCGAACTGGTGAAGCTGACGCAGGGAATGCCGTTGAATCGCTA

TAGAAACGCCTTCATCAACCTCGCACTCCCGTTTTTTGCCTTCACTTCTCCCCTTCCTGCCGAGGAGTTTCCCGGCGTGA

GAGGAGAAACCCACACGCTGTGGGACCAAATAAACATCAAAGAAGGAAAGAAGGCGGCGAAGGCAGGTGGACTGACCGTC

CGGCGCCTCCTCAGAAGAATTCAGAAAAAGGCGCATGCCGAAGACCCCGACGCTATTCAGGTTTCYAATATTTCTATTGG

GCCCATTATGATATACGCTAATTTCTTGCATGAGGACGATGAAGAACTTTTGAACAAGAGCATATGGAAAGTTATTGAGG

AAGCTGTACAATCTGGGGCARAATTCGACGAAGAATTTTCTCGGGATGGCCCAGTGGCCAGCGATGATAGCGCGGCTACG

GTATCTATCTCCTCGGCCTCCTTTGTCGACCTCGCAGTTTCCGTTGAAGATACCGAAACATACGAAGAAGTAGAACTTCC

CCCGGTTCGAGTCTTCCGCTCGTGATTAAAATAAAGATATAGAGAACGCCTAATCTCCGACTCAAGGATCAATGTGTTTC

AACCATGCTCTTCTGTTGAAARCTAGGCCGGAGTGTTTTCTATATCATCGTTGCTATTTCCGGTCAACAATCTTGCAATA

GCTGATTCTGGAACCGCTTCTAGTGTGGCAACCTTCAAGGCCTTCGCAAAAAGAGATGGGAAAGAATTGAGATCGATGTG

CGAAGTAGATAATGACTCTTTCCAAACTCGTTCATGCAAATACTTGCCTGGAGAGCTGTGCGCAGGGACTCCAACGATGG

TTCACAATCTGTGGTAACGCAATCGACCAGTCTCCGAGTCGTTTCTTTCAATGTTTTCTTATGACGGATTTCTGTCTCGA

TATCTGTGTCTTCGCCGCAAAGAGGAGATTCTGCAAGTTTKTCCTTGAGACTATTTTCAATGCTTCGCGCCTTCACAAAG

AAATTCTGAATGAGAAGCTTTGTTTCGGAGAAATGCAGCAAATAGTGGAGAGTCTTCGATACTTACCTGTTGGACAACAT

CAGATGGCCAGCCACAAGAAAGAGCCATTTCTACGCCATAACTCGATGTTGCATCACACGGCCCGATCCCTATCTTGTGG

CTATATTCRATTTCTCCGTTCCCGTCATCTGGTATTGACGCTATGAGGTGCTGATTCTGAACAGCAGGGTAGGCACGGCT

CATTTGGCTCATCTGTGGGTAGTGGGAGACGAAATAGGTCATGGCACCCTTGGCCAGGAGACTCTCTGCAACAGCCCATG

CAATGGCTACTCCATCTTCGTTACTAGTGGCCCTGCCTAGCTCATCTAGAAGGATCAAAGACCGRGAAGTCGCATTATTG

CATATGAAAGCAACCTCTTTCATTTCTAACATGAAAGATGATATGTTGTTCTCTGTAAAGAAGAAACGAATGGAATCGAA

AAAAAAAGCAAAAGCGAAAAACTGAAATGAGAACATGTCTCGAAAAGAAAGCAATAAATGTCAGTAATTGCTTTGTACTC

GTTACCTTGATCATCCGCAGTCATCATCCTAGCACAGAGTCGGTTGGTGAGCTGTGAATAATATCAGAGTGGAATATGAA

ATCGACGAGATAAATAGAATATGGTTGGATGACGYCTCTGATCGTAGCACTACGGAAACAATCTATTCTATCAGCTAGCA

CGGTACAAACTTACGGGGATCAAGGCTTCTTCTGCGGGCACGTAGCTTCCACAATGCGCTAAAAGAACAATGATTGCGAT

TTGTTTGAGATATGTAGACTTTCCACTTCCGTTGATTCCTGAAATAATAGTTAGATTTTTGGACAAGCTTGCGTAAGTAT

CGTTTGCAAYCCATTCTCCAGGCCCACCGTCGGAAGGAAAGACTGAATCATCCACGTCGATTCCGTATCTTCCGTTGCAA

ATAGCAATCGCATAGGACTCCCCTGGGTTGGCAGCAGGAGGGACTGCGCTGATGTGATCGTCACCCTCGTCGCTGGTTTC

GGTTGATTCAGCCGCTGGTACTGCTCCATCGGTTAGCAACGGGCGTGCCCAAGGGAGTTTGCTCAGCGTGACTTTGTCAG

CAAAGCTGTGGCAGAGGTCTAAAAGAGCGACAGCATCCGACAGACGAGCCAGGGCATCGTACTTAGACCTAGCCACGTCG

AGGACCTCCTGGATTCTTTCGTGAGTAAGAATCAACAAGTCTTGGATATTGTCCTGGGATCGGGTGTTGAGGGACTGAAC

CTCCTCGGTAGTGCAGTATATATGTTTTCCCGACTTGGATGGCTGAATGAATTCATTGGGCAGGTTAGATGCCATTTCGA

GGGGCAGCGATAGATAGTACCCCCTCGCTGCGGAATACCTGACAGCCACGTGGGAGATTCCGTGGATCTCGGCATACTCG

TCGGCTTTTTTGTAAATATCGTCCACGTTGCTTAGAAAAGCCTTGATATTTTTAAAGGGGTAACCAGATTTTGTA

>PsnmuV1.4_aug-pasa-abinitio_v2_0010060.1 class=Sequence position=[PsnmuV1.4_scaffold_8-size_368192:247937..252155 (- strand)](http://gbrowse255.tgac.ac.uk/cgi-bin/gb2/gbrowse/maplesod_psnmu_v1_4_gbrowse255?name=PsnmuV1.4_scaffold_8-size_368192:247937..252155) ***MSH4* AUGUSTUS V2 Corrected gene modeel**

AATCATAATGTGGTATTCATCTTCCTTCGCAATAKTAGCAATATCGAATCAAATTTACTATTCAAAATTACTTACTGTGA

GTAGAGTTGTGTTCTCTTTATCTTGATTTCCAGTTCCCTCTTTCCTCCATCAAACACCTTCGTACTGCTATGGATCATCA

TCGGAACGCAACTCGGGATAGYCAGGGAGATATCTCGAAGAAGACGAAGAAGCAGCGCATTGGTGGTAGTAACAACGGAA

GATTCAATGTCGTATGCGCGARCAACAGTGCTAGATATCATTGTCATTCTTCGGGTAGATCCGTTTCGAGTGCCGCTTCT

CGCTCCAGCAATGCCCGCAATCGTCTTCGACGCCGACAMAATCTCGGCATTGCAAGCAGYAGTAGCAGCAACCGCAATCA

TGCCGGTGGTCGCAGCCGCTGTAGCCGTCGTAGCAATTCGTGCATTATGCAAGGAAATGCCTCGTCGTCATCTCCCCATA

TTCTTTGCGCGATTGGAGAGAACCTAGCTCGGGAAACCTGCGTYGTGTCCCTGGATTTGTCGGCTCCCTTCTTGCTCAAT

GTCACCAAGCAGAGCAATGGACAGAGTTATTCGGAAACAATTGCCTATTTGTCGGTCTTGTGCCCGGACGAGGTCTTGAT

GAACGAAGGCCGCTATCATTCTCCCCTCGCCCGCAAAGTCCTCCAGCACTTCGATGTTCAACGAAAGCATCAGCAGCAGC

AACAGCAAWACGACTCGATGGYAACAGGATCMACGGGAGAGACAGGGCAAGCTGCTATCTCTTCCGAGGAAGAGGCGACC

GTTGTCAARTTCATTTCGCGGTCCTACTTTGACCAAACCAAGGGGGCCGATCTTCTCCGACGACTCGCAAGGCAAGACAC

ATACGATTCTACTCTAGTGGAAGAATACATTTTGCTGTCCAGCTGCCATGCGGTTCTTCATTATACGCAGCTTACCCTTG

GGGCTGTGACCTTTACCAGACATTCTCTGGATGTTCGAGTACACACAGGTGGTCACAACAACCACCGAATGGAAATAGAT

CGCTCCACCTTGCTGCAGTTGGAACTGTTGACAAATAACAGCCACATGGCAACMGCAGGATCATCCATGTCCAATCACCG

CAAGCATTCGCTCATTTCGACCATGGATTGTACAAAAACTAGCGTTGGACATCGATTGCTGCGAACGACTCTAATGGCGC

CACCAAGCAGGTACGTTAGATAAACCGACTATTGCCCATCGTCCGATGCGAATGAATCATTTTAGTCCCTCCAATTTCGA

ATGCTCATCGATCAGATGCTAAAAAATGCGGATTCATAGCAAGCTTTCGTCGTTCTTGGTTCTCATTTGATATTCCGCTT

TTACTATTTTTCTCCAACGCACCACAGACTAGATACTATAACGGCACGGCTAGATCTAGTTGATACATTTCTACACAACG

AAGATCTGTTTTACGCGACGCTGCAACAACTCCAGAATCTAGCTAGTTTGGATAATATGCTAACAAACATCGTTGTTGTT

CCTTCGAGGAAGCAACAGGAACAACTACAGCAACGAAAGCAACATCAAGAGAACAGGGGAGGATTTGGAATTTCTTCGAC

TTCCGCTTCAACCAGTAATAATAATGCTTCGAGCCACCTAGCAGTGCCGTTAGCACCTRCTGCAGSTCCTGCCACGGGCA

AGAGCAATGCTGCGAATGCGCGCATCGCCAGCAAGGGTATWTCTGCGTTGGTCTGCATCAAGTCTACACTAGCATTCATT

CCCGTCCTCGCATCGATCCTGAAAAATCACATGGAGCGCATCGAAAACGAAAGCAAGAGCAGAGATACGGAAGCGCAACG

GAAACAGCAGCAGCAGCAGCAACAAAACAACAGAGAAAGAATTGAGGAAAACGAACAAGAAGCAAGGGACGAAGCTTCCA

TTGCTACCGCCAAAACTAGTTTGTTGATAGGTCTAGGCGTGGGCAAAGGGCACGGATCAAACGCTTCCATTGGTTCGTAT

CCGTCCTCCGATTCCTCAAGCAAAGACCACCAGCTGCTTCGTGCCATAATATTTGCGCTAAGGCAACCAGAATTGTGCAT

TGTTCGGAATGCCATTGACGAGGCTTTTACCGAAAGTACGTCATATACCCGTAATGCCAATGCGATGAAGCATCAGGAAT

GCTTCGCCCTCAAAAGCTCTGATGACAATGGAATGATGGACGTTCTTCGAAAGGTACGTAAGCACAACAAGAACAATTCA

TAACTCTTTGTTTTAAAAAAATTGTGACTTCAACTTACAAAATCTGGTTACCCCTTTAAAAATATCAAGGCTTTTCTAAG

CAACGTGGACGATATTTACAAAAAAGCCGACGAGTATGCCGAGATCCACGGAATCTCCCACGTGGCTGTCAGGTATTCCG

CAGCGAGGGGGTACTATCTATCGCTGCCCCTCGAAATGGCATCTAACCTGCCCAATGAATTCATTCAGCCATCCAAGTCG

GGAAAACATATATACTGCACTACCGAGGAGGTTCAGTCCCTCAACACCCGATCCCAGGACAATATCCAAGACTTGTTGAT

TCTTACTCACGAAAGAATCCAGGAGGTCCTCGACGTGGCTAGGTCTAAGTACGATGCCCTGGCTCGTCTGTCGGATGCTG

TCGCTCTTTTAGACCTCTGCCACAGCTTTGCTGACAAAGTCACGCTGAGCAAACTCCCTTGGGCACGCCCGTTGCTAACC

GATGGAGCAGTACCAGCGGCTGAATCAACCGAAACCAGCGACGAGGGTGACGATCACATCAGCGCAGTCCCTCCTGCTGC

CAACCCAGGGGAGTCCTATGCGATTGCTATTTGCAACGGAAGATACGGAATCGACGTGGATGATTCAGTCTTTCCTTCCG

ACGGTGGGCCTGGAGAATGGRTTGCAAACGATACTTACGCAAGCTTGTCCAAAAATCTAACTATTATTTCAGGAATCAAC

GGAAGTGGAAAGTCTACATATCTCAAACAAATCGCAATCATTGTTCTTTTAGCGCATTGTGGAAGCTACGTGCCCGCAGA

AGAAGCCTTGATCCCCGTAAGTTTGTACCGTGCTAGCTGATAGAATAGATTGTTTCCGTAGTGCTACGATCAGAGRCGTC

ATCCAACCATATTCTATTTATCTCGTCGATTTCATATTCCACTCTGATATTATTCACAGCTCACCAACCGACTCTGTGCT

AGGATGATGACTGCGGATGATCAAGGTAACGAGTACAAAGCAATTACTGACATTTATTGCTTTCTTTTCGAGACATGTTC

TCATTTCAGTTTTTCGCTTTTGCTTTTTTTTTCGATTCCATTCGTTTCTTCTTTACAGAGAACAACATATCATCTTTCAT

GTTAGAAATGAAAGAGGTTGCTTTCATATGCAATAATGCGACTTCYCGGTCTTTGATCCTTCTAGATGAGCTAGGCAGGG

CCACTAGTAACGAAGATGGAGTAGCCATTGCATGGGCTGTTGCAGAGAGTCTCCTGGCCAAGGGTGCCATGACCTATTTC

GTCTCCCACTACCCACAGATGAGCCAAATGAGCCGTGCCTACCCTGCTGTTCAGAATCAGCACCTCATAGCGTCAATACC

AGATGACGGGAACGGAGAAATYGAATATAGCCACAAGATAGGGATCGGGCCGTGTGATGCAACATCGAGTTATGGCGTAG

AAATGGCTCTTTCTTGTGGCTGGCCATCTGATGTTGTCCAACAGGTAAGTATCGAAGACTCTCCACTATTTGCTGCATTT

CTCCGAAACAAAGCTTCTCATTCAGAATTTCTTTGTGAAGGCGCGAAGCATTGAAAATAGTCTCAAGGAMAAACTTGCAG

AATCTCCTCTTTGCGGCGAAGACACAGATATCGAGACAGAAATCCGTCATAAGAAAACATTGAAAGAAACGACTCGGAGA

CTGGTCGATTGCGTTACCACAGATTGTGAACCATCGTTGGAGTCCCTGCGCACAGCTCTCCAGGCAAGTATTTGCATGAA

CGAGTTTGGAAAGAGTCATTATCTACTTCGCACATCGATCTCAATTCTTTCCCATCTCTTTTTGCGAAGGCCTTGAAGGT

TGCCACACTAGAAGCGGTTCCAGAATCAGCTATTGCAAGATTGTTGACCGGAAATAGCAACGATGATATAGAAAACACTC

CGGCCTAGYTTTCAACAGAAGAGCATGGTTGAAACACATTGATCCTTGAGTCGGAGATT

>PSNMU-V1.4_AUG-EV-PASAV3_0023810.1 class=Sequence position=[PsnmuV1.4_scaffold_154-size_117545:45016..48810 (+ strand)](http://gbrowse255.tgac.ac.uk/cgi-bin/gb2/gbrowse/maplesod_psnmu_v1_4_gbrowse255?name=PsnmuV1.4_scaffold_154-size_117545:45016..48810) *MSH5*

ACATTCTTTTCTCCGACTACGACGTACCATAAACAATCGTACAGTCCCTGCCATTGTACGAGTATTTACGTGAAAGCTAC

GTATTCACTTGTTTTCTGTCTTTCGATTGAAAACGACGGAAAAGGAGCTAAGTGGGACTCGGATTTTTCTGTGCAGACAC

GCGCGTTGTGGAAGTAGCTCATCGGATTGTTTTTTTCGTTCAATCTTTCTCTCTCTCGAAGCCATTGTTCATTGTCATTT

TGAACCAACCGTCCATCGGAACAATGAGAAGGACCTCGATTGGTAGCAGTGGTAGTGCTGGCGGTAGAAGCCTTGCGAGT

GTGCTTAGCCACCGGAGTTGCGATCCGGGGAGAGAATCTTCTGTCTCTTTGGCGTCGCACCACGACAACGACAACGACAA

CATCGACGAGAGAAGCAGGAGATCTGCTTATTCGTTCGAAAGCGGGAGTGTTCGCACTTTGACGAGTCGTGGCAAATGTG

CACCCTCCAGCAGCAGAGGCAGCAGCAGAAGGAATCGAAGCCGTTCTAGTGGGAGACAAACGCGAACGCGAGTTGGTGTC

GACGACGAGGACACAACCACCACCAGCGGCAACAGACGAGTCCTTTTGCAAAACCAGAGCAGTCCGGGGCAAATTCCCGC

CGCGGCAGCCGCTCCACTGGAACCAAGCCTTTGCGTCATGTCGGTGGTCGAAGAAGGATCTAATATATCGTTTTGCTGCT

ACAACGAAGATCGAAACGAAATCACGACGGAAACCTGTGCGGCCGTGACTGGATACGAGACAGAATCCTTGGTGGAGCGC

TTCTTGCAAGCGACGCGACCCAACATGGTCTTGGTGGGGTAAGTACGAAAATAAAGAGCAACGCAGGAAATATCGTTTTC

AAAAAGCCATTCCGAAGGATATTATTAGTTGTTTTACACTCTAACAGTCGTATTGTTTTGTCAATTTTACAATTCACAAC

TTGGTTCGTTCCCGATATATTTTCCCTTCAATGTCGGCTCCAAACCGACTACCGAATAGAAATCGGATCGTCAACAACAT

TCCTCTTCTTGAAATGGTAACGCGACAACCTCAACCTCTTCTGGAAACCGAACATGAGCAGCAGGGGCACCATCACCGTC

AAGAAGGAGGAAACCCCTCGCCTCGGTCTTCCATTCCGTATCGTCTGTTGAAATCAGGAATCTTTGACGTTCGTGCTTGC

AGAAGTATTATTTTGCAAAAATTGCAGGTGCTCTCCATGCTCAAACAAAGAGCCATGGACGAAGCACGACTATCAGCAAC

CGCAGCAGCAATGGGAAGGAGCGACAGTAGATATCGAAATCACCCGGACAACGATAGGCATTTTCCCCGGGATGGGTCAT

CCACACAGACCCTTTATCGCCCTTCCAGTTATCATTATTTGGCAGCCGTCATCGATTTCGATTCAAAGGTTCTGGTGCAA

GCCCTGGGAGCTCTTTTGTCGTATCTGCAGAAAACAGTCTTCCAACTCGAGGAAGGGAACACCATCACCGTTAACAGGAT

CATCGAGGCTGGAATATCGTCGTATTTGATCCTTTCTCCGTCGACGTTTTCGGCGCTCAATATCTTTTCCACCGAACGCC

ATCCACTCATTGCCAAGGGACACGGGCATTCCAAAGAAGGTTTTAGTCTATACTCCTTGCTCGATCGTACCAAGAGTAAG

TGGGAACAGCGAAACGGAAAACTGTTCGCAAGGGTATTTCCAACAATCGACCAATGACCACACGCAATCCCATATCTTAG

TGACCGATTTAGACTTCGTTGTTCCGAACTCGCACGTTGACTAACAGCTCGACGTTCCTTTTGGTTGCCTTGTTTTTTCG

TTTCCGTTTCGCTCCGTCCAAACATTAACAACGGCGATTCAACTTTTTGCTTTGTTAAAACAAACAACCAGGTCGAGGGG

GGAAGCAATTGTTGCGGGAGTGGATGCTCAAGCCATTGACCAATCTGGAAAACATACAGACACGCCAAGACGCTATTGAA

CTATTCCTGCAGCCAAGCATGCAGGCATCGGTTGGTGTTCTCATCGGACTTTTGCAAAAGGTCGGTCCGGTGGACAAAAT

CTTGATGCGAATCCAAAAATGCACCGTCAAGCCAACTGATTTCCTGGTCCTAACCACGACACTCTCCGCAGCTATTTCGA

TTGGCAACGTGCTCCGGAACGATCTTCTGGCGTCAATGAATCATACAACAGCTCCGCCACAACAGGGCCCCAACAGTTTC

TCGTGTGATCACCAGTACTCCTTCTTCTCGGAACTTTGTATCGAGTGCAATGTGGAAGTTATGATCGATCTGAGGGAGCG

CATCACAAATACCATCGATAGTGAACTAACCTTGGAGGAAAAGGGAGCCTCGGTCGTGATTCGTCATGGCTTTCACGAGC

AGTTGGATGGCTGGAAGGAACAATACGAATGTCTCGAAGAAACCCTGGCAGAAGCAGCAAAGGATCTCTACCATAAATAC

GGTCAGCAGCTCGATGGGCTGACAGTAATATTCATACCACAGGTTGGATACCTGGTAGGCTTAAACGAAACCCTTGTTCT

TCGGACACTCAAAGACCCCACGTCCCCATTGCCCCCAGACTTTGAACAGATTTTTGTTCAAGACGGTGAGGTTTTTTTCA

AATGCGACGAGATGAGAAGGATGGACGAAGAAATTGGTGACTTGGATGGCTTGATTAAAGACACAGAGCAAATGATCGTA

ACAGAGCTAGAGGAAAACATCCTCGACACGGAAAACGAACTTCGAGAATGCTTCAGGGCTTTGTCAACCTTGGATTGTTT

GCTTTCGTTCGCCGATTGCGCTGCTGACTTGGGATTCACTCGACCGCAGCTGATTGACGACGATGAAGATCTTCAACCTC

GAGGTCACCAGCAGCAACACCATCCGCAACGGAAGCAGCTGATATACATCCGTGACGGCCGTCATCCGCTCCAAGAGATT

ATTTGCGACACCGATTTCGTCAGCAACGATGTCCAGATTGATGATTCCAAGCAAATATTGTGCGTTACCGGACCGAATTA

TAGTGGAAAGAGCTGCTACATGCGACAGGTTGGACTCCTCGTCTACATGGCACACCTCGGATCGTTTATTCCGTGCACGC

GAGCAATGATTTCCATCACAGACCAAATTTTCGCACGCGTCTCGACTATCGAGACCTGCAGTAGACCCCAATCGAGCTAT

CAATTGGAGCTCACCGAAATGGCTGCCGTTCTTCTAAAGGCAACGCCAAAATCGCTCGTCCTCGTCGATGAATTTGGAAA

GGGGACCAACCCCGCGTCGGGAATCGCAATACTGGGGGCCGCTTTGAAAAGGCTTTCGACCATCCGATGCAAAACGGTAT

GCGTAACCCATTTTCTGGAGATGTTTACCATGAATGTCATCGAAGACGGCGAAGAGGGCATTCAAGCTTCTCGAATGACG

ATTCATTTGAAGGAAGGGGAAGAGGACGGTGCTTCGCCCTTGTTCAAGCTGGAAAAGGGTGTCGCCTCTTCGTCAGCCGG

CCTTGTTTGTGCAAAGAATGCCGGAGTGAGCCATGCCGTCATCGATCGTGCCAAAGAAATTATCCAAACCATGAGGGCGA

GGAAATTTATTCGGCCGTTGCCAGAAGCAACAAAGCAAATCCTGAAATTGGTGGGAGTAGAACGGGAAATGCTCGCGCAT

TTTCTATCAGTGGATTCCTGGGAAAACGCCAGCGATGACAGTATCATGGGTCTGCTGCAACTCTTAGCAAAAGTGAGCAC

TCAGGAATAGTTCATATACCGGCGCGAAATCCTTT

>PSNMU-V1.4_AUG-EV-PASAV3_0056780.1 class=Sequence position=[PsnmuV1.4_scaffold_268-size_73268:57989..59935 (- strand)](http://gbrowse255.tgac.ac.uk/cgi-bin/gb2/gbrowse/maplesod_psnmu_v1_4_gbrowse255?name=PsnmuV1.4_scaffold_268-size_73268:57989..59935) *RAD51-A1*

CTTGGAAGCTACTGGCAGCAATGCCTCCAGAAACTTTTACCAACAACYAAACAAGAAGTGCACAAGAAGGTACCAATCTA

ACAACAACACAGCAAGCTCATTCTATTACGGTAGTCTGCAATCATKATGGAAACCGAAGYATCTCCGRATTTTGCCGAGG

AAAATCATCATGGCGAGCAAATGGGGGAAGAGGAAGAAGAGGTGCGACCTAGCTTTTTATTGATACAATGGCTGCTTGYG

TCTCGTTGTTGTCGACCGAGCACTGCACGGACTACCGCATTGCGTCACAYGATGCCGACACACCCGGGAACGACGAGCAA

CATACCGAAGGATCTTGTCGGGGAGATGGTAGAATCAAATTCTGTATCCTCTACCGACTGCTTCTTCTCTGCAGGTTGGA

GGCGAATGACGAATTAAGTCGGCTAACCCAGCGTTTCTGTTTGCTTTGAATCGGTGCTCCTTTTCTACAGGCCAATCCCA

TTTATCAGCCCCTTGACCTACTCCAAGAGCACGGTATTGCCACCAATGATATCCAAAAGCTTCAAACGGCCGGGTATCAC

ACAATCGAATCGGTACGTTCGGCACGAGGAGTCGTTGGSTACCGGTGCTGTGTTTCGGTCCCTGTTGTCAGATTCACCCC

ACTCACTGCGTATATTGCTTCCACTTCCAATTCTTCACATCGAACTTTGATTTGAATTCTTTCTGAATCACTTTTCGTTC

CTCCGTCGCTCGCTCCTTCGCATTTTCTGCTCGCCTCCCAGGTGGCCCACGCCACGGTCCGCCGTTTGTCGGACGTCAAG

GGAATCTCCGAAGCCAAAATCATCAAGCTGAAGGGGATCGTCAAACAAATGGTGGCGATGGAATTCCAAACCGCCACGGA

CGYCCTAGAAGCCCGCAAGAACATCGTTCAGCTCACAACTGGATCGGTSGAAATTGACAAGCTGCTGGAGGGCGGAATCG

AAACGGGATCTATCACCGAAGTCGTAAGCCACCGATGCCGAAACGAAGCCACGYTTGTCCGCCTCTTGCCAGAATCTTCC

TCTCAATCGTCGCCCTCACACCTTGCTCTGTGTTTTGGAAACTCTGTTTGACTTAGTTCGGAGAATTCCGAACCGGAAAA

ACCCAGCTCTGTCACACCCTCTGCATTACCTGCCAGATGTCCTGCGGGGAAGGCGGCGCCGAGGGAAAGGCCATCTACAT

CGATACCGAGGGTTCCTTTCGCCCCGAACGCCTCAAGGTGATTGCCGAGCGGTTTGGTCTTGATCCTACGGTGGCCCTCG

AAAACGTGGCSTGTGCGAGGGCCCAAAATTCGGAGCACCAGATGGAGCTCCTAAAGACCGCGGCGGCAATCATGGCCCAG

GTGAGTTTCTRGCTGCTACTAGATGGAGTTCTTTGGCGCATGTCTACCGAYGCTTTTGTTTCTCACAGTGTCCCTCGTTC

TCTTGCTTCGTCGTTCCGCGAATCCTTAACCCAGGACCGATATGCCCTGCTGGTGGTGGATTCTGCCACCGCGTTGTACC

GGACGGATTACCAGGGCCGCGGTGAGCTCTCCGAACGGCAGATGCAGATGGGCCAGTTCCTCCGCCAGCTCACTCGATTG

GCCGAGGAATTCGGAGTAGCCGTGTTCATCACCAACCAGGTCGTGGCGAATCCGGACGGAATGAGTTTCGCTAAGGACTC

CACCAAGCCCATCGGCGGGAACATCATTGCCCACGCCTCGACSACCCGTTTGCGCCTCCGCAAGGGACGTGGCGAAAACC

GCATCATGACCGTCTTYGATTCGCCCTCGCTCCCCGAAGCCGATGCCCAGTTTGCCGTCTCGGCCGCYGGAGTTTGCGAT

GCTACCGATGCGTAGTGGAGCGAMTTTTGTGACAGAGGGTTTTGTAGTACGGATGCATTGAAATTGTGATTGCCCCAATA

CCCAACAAATAGAAGAATATCTGCACA

>PSNMU-V1.4_AUG-EV-PASAV3_0086180.1 class=Sequence position=[PsnmuV1.4_scaffold_44-size_221303:46086..47878 (+ strand)](http://gbrowse255.tgac.ac.uk/cgi-bin/gb2/gbrowse/maplesod_psnmu_v1_4_gbrowse255?name=PsnmuV1.4_scaffold_44-size_221303:46086..47878) *RAD51-A2*

TTGCACCACACACTACAGTAATAGTAAAGGATTAACTGTTCGTCGGATAAAGACAAAGTAAACAAACCATACCATGATGG

CGACAGAGACAGCCGCTCCTATGGAGCAACAACAGTATGACGAAGGAGAAATGGAACAAGAAGAAGTGAGTTCTCGAGCT

ACGATTGTAATGTCGATGATTGAAACATTGGATCCCAACGTTTGAAGCTTGTCGGCACGCAATTTCACGACAAACTTATT

CCGCTCAACKAACATTCTAYCGYGATCAAACCKTGGTGCTTTTCGAATCTTACAACAAATGTTTCCAATTTTTTGTTCCT

GCCTCGAAGTCTGCCACTACCTACCAACCGATCGATCTCCTCGCAGAACACGGAATTGCMACAAATGACATTCAAAAGCT

TCAAAATGCCGGGTATTACACGATCGAATCGGTGAGTCCGTTGCCATAGACAAAGGAATGACTGCGAACTRTGTTCCAGA

TTCGTGGGTAATGACCGTATGAAAACTGTAAGTGTGCGCTGTCCCTCACCGATATTCCTGCCATTTGTGCATACCGTACG

GCACATCGTTCCTCGCGTTTTGTTCTATTATTCTTCCATCTTTCATCAACGTTGCTCCCAATCCAAAAGATTGCCCATGC

CACTACCCGTCGACTCTCGGACGTCAAGGGAATTTCGGAAGCCAAGGTTCTCAAATTGAAAGACATCGTGAAACAAATGG

TATCTATGGAATTCAAAACTGCGGCGGATGCCTTGGAAGACCGACAGACAATAGCCATGCTCACGACAGGAGCCGTCGAA

ATAGACAAATTACTGGAAGGAGGAATCGAGACCGGATCGATTACCGAAGTCTTTGGCGAGTTCCGAACCGGAAAGACCCA

GCTRTGCCACACCCTATGTGTGACATGCCAGATGCCGGTCTCGGACGGCGGGGCGGAAGGAAAAGCCATATATATTGATA

CTGAGGGGTCCTTTCGACCCGAGCGCCTCCGGGCCATTGCCGAACGATTTGGCCTCGATCCGGCCGTAGTCTTGGAAAAC

GTGGCCTGCGCTCGGGCACACAACTCGGAACACCAAATGGAACTCCTCAAGGTTGCCGCGGCCATCATGTCGCAGGACCG

ATACGCGCTATTGGTGGTGGATTCCGCCACGGCTTTGTTTCGAACAGATTATACGGGCCGCGGAGAACTGTCCGAGCGGC

AGATGCAAATGGGACAGTTTCTCCGGCAATTGACGCGATTGGCAGAGGAATTCGGAGTAGCCGTCTTTATCACCAACCAG

GTCGTTGCCAATCCCGACGGAATGAGTTTTGCCAAAGATGCCACRAAGCCAATTGGGGGAAACATCATTGCGCACGCCTC

AACCACGAGGCTGCGTTTGCGAAAGGGACGAGGCGAGAATCGGATAATGTCGGTCTACGATAGCCCCAGCCTGCCGGAGG

CCGATTGCCAGTTTGCCCTCTCGAACGCCGGAGTTTGCGATGCCACGGATTGAGCATATAGATTTTGGAATATTACGCCT

TCCCATAGAGAAAAGCGATGTGAAAAAAATGCATCTTTCGTTACATCTTCACCTTTMAAAGTTTGTGGTTTGTACGGTGC

TGTAATTGGTAGATCTACGAWTCAGTTCAGAAGAGATAGCAATGAATCCTTGCTGGCACTTGAATCAATGAAAAAAAATC

ACTGTAGAAATTTTATTTCTGACCCTAAATCCGTCGGTATTGCATTACTACAGACAGAAGWACTCAGTAGTGTTGTGACA

AATTGTCGGAAGGAATATAATTTAAAACTTTTT

>PSNMU-V1.4_AUG-EV-PASAV3_0105810.1 class=Sequence position=[PsnmuV1.4_scaffold_634-size_14067:7925..9985 (- strand)](http://gbrowse255.tgac.ac.uk/cgi-bin/gb2/gbrowse/maplesod_psnmu_v1_4_gbrowse255?name=PsnmuV1.4_scaffold_634-size_14067:7925..9985) #*RAD51-B*

GGGCACAAATGCGATGCTSRWGKWTGCTGATTCTAAGTTTWTATACTTTTGTTCTGTCGATTGTTTCCYTAGACACGACT

ATTTGTTTCAGTATCGTACCATAGCACCGAACGTTCGCGAGTACGAGTATTCCTCATCAATTTCCTGGGATTGGTTGCTT

TTCTATTGTTTTCATGCTCGAGAACTCTGGTCCGATCAAGCGAATATGATTGCGCATGAACCATGAACAATACCGATGAG

CGCTTCTAATAGAAAGATTTCACGGCTGCCCCTTTCGGTGTTTCAGGATCTCATTACGCGAGCCCCTCTGGATGGCYGTG

ACGATGGTCCTGAAAGTCTCAAAGCCAACATCTGTAAACGCCTGAAAGGTTTCCGTGTTAGAAACAATGCTAGGGGAAGA

AGCAAGAATAAAAACCGTGGCCGTGGAGTTGGATCGGGAATATCATTGTCTACCACGACAACTTATAACATCAAGACCAT

CGGGCAGCTTCTCCGCMTATCCAAGTACACTCTAATGTTGGCATTAGACCCGATATTGACCTACGAAGAAGTCGGAATAT

TGCTCAACAGAGTGTGCAATCAATGCGCACCGAATCCAATTTCTGCACTCGAACTATTCGGTGCTACAACTTTAAATACA

GATAATGTTTTGGGCATGTCGRGWTACAGAGATGAGGGGAAACAACATGAAAACGATCCCATATCTTTTGGTAGCAAGAT

GAGGTATCTACCGACGTCGCTGCCGTCACTGGATCGTATTTTAAATGGGGGAGTTCGGTTGGCGACGGTAACTGAACTGG

TGGGGAGATCAGGAGTTGGAAAGACGCAGCTCGCTTTCCAGCTTTGCGTGACTGCGGCAAAATTCAATCAGGGGGCGATM

TACATAGACACGGRAAAAAAAATGTCTCTGGAACGACTAGGAGAAATGAGCGAACAACGGAGATTGATCGGTAACAATCA

CGAGAGCAGAGATGAAGACGATATGGGCATGTACAAATCGACCCAGCTCGTTCTCAATAATCTGACCGTCCACCAGCCAG

AAAATACCGACGAACTGTTACAAGTTTTGGATGGACTGGAGTACGAAATTCTGATTCGAAATCAAAATGCAGGCCAAGCT

TTTTTTCAGTCTGGTGCATCTACGGACATCAACACATCTCTCAGCAACACCGCTGTTCGAGGAAAATACCCGGTTCGCCT

GCTGATCGTGGATAGTATCGCCGCACCTATGCGACGTGACTACGGAACAGATTCTGCGCCGCAGAGGGCTGCATCTATCT

TTCAATGCGCACAAAAGCTCAAGCGATTGGCAGATCAGCTCCATTTAGCCATCGTTGTGATCAATCAAGTCGGTGCTGCT

AGTGTCGATAGTAATGCAATGAAGAATCGTTCCAGTGAAAATAAGAATGATATCTCCGGATTTGGACTAAATGCCGAACA

GCATCCAAGCAATGTACGTGCAGCTCTAGGAACTTCTTGGCATCACTGTGTGTCTACAAGGCTCTTGTTGGAAGCCTTGA

ACATCCCTAATTTTATGCCATCACCGGAATTATCTAGCCTAGGCAATACCAATCGCAATGGTTTCCAGTGGGAAAAAGCG

ATAGAACCAACTCATCTTATCACAAAAAAGATTGCTGTTGTCAAAAGTAACACAACAGCGCTCGGTGAAACTCAATTTGC

AATCACGACTGCGGGGATCATCGAGGATCGACCGTCTGCTAGTCCACATAAGGACCAACAATTGTGATGGTTCAGTCCCA

ACGCAATGCCAAATCATTTTTTCGTGCGAATCAAATTGGGACGGTGCTTGTGCTCAAGTTATGGGATATGGCATTAGCCT

GCCCCTGATGGGAAATAGAATTACTCGACTTCTCGACTTCTCGACTTCACCATAGTAATAAACATAAAAGAAAACAACAA

ATAGAAGCCCTACTTTCTCTTTTTGAAGTAAGAGTTGAGCATTAAGCTCTCGTGATTTCCTAGTTTCTAACTGTGTGTTG

ACTTTTGAAGATGATGATCAAAACAATTTTTTGCTAGCAGAAATAAAATGCATTGCATTGC

>PSNMU-V1.4_AUG-EV-PASAV3_0104040.1 class=Sequence position=[PsnmuV1.4_scaffold_61-size_202280:88426..90290 (+ strand)](http://gbrowse255.tgac.ac.uk/cgi-bin/gb2/gbrowse/maplesod_psnmu_v1_4_gbrowse255?name=PsnmuV1.4_scaffold_61-size_202280:88426..90290) *RAD51-C*

CGCGAAGCAAAACAACCCGGCCCGTCGGAAGAAAACAAAACCAAACCAAACCAAACCAAACACCACATTGCATGGCAAAG

ACCGCACCACACCACAAAACGACACGACACAAAACGACACGACACTGCCCGATATGGAAGCGAGGGGCACAACGGCGGGT

GCCCGGCGACCCCCCTCGGTCCCCCTGTCGGAGCTCCTCCTGCGGCCGTCCACCCTGGAACGCTTTTCCCGGCGGGGCTT

CGAGTCGACCGGCGAGATCGAGGAATCCCGGGCCAGCGGCGGGATCAATCTCCTGGCCTCGGAGCTCGACGTCTCCCTGG

GGGAGGCGGCGGGGCTGATCCGGGAGGTCCAGGGCTGCCTCGGGTCCTTTTTGCCAAGGGGCCGGGCCGATGCGAGCAAC

GAGAACCAGAGCATTGGCATTGGCAAGGACAACGAGCACAACGGCACCGAAGCACCGCCAAAGAGCCGGGACCACGACGA

CGGGATCGTCACCGCCTACGAGCTCCTCCACCGGAGCGATCCCTTGTCCTTCCCGGGCAGCAACACCGGCAGAAGCCAAA

GCCAAAGCCGAAGCGGTTGCCTCCCAAACGGCCACATCGTCACCTTTTGCCGGTCCGTCGACGACCTGCTGGGCGGGGGG

ATCGAGCTGGGAAGCCTGACGGAGGTGGCGGGCCTCCCCGGGACCGGAAAGACCCAGCTGGCCACCCAGCTGGCGGTCCT

GGCCCGGCTCCCGCCTTCCTTTGGGGGCGTCGGGGGAAGGACCCTCTACGTCGACGCCGAGGGGTCCTTCTCGGCCGACC

GGGCCTGGGCCATGGCGGGGGCCCTGTGCGGGCACGTGGGGGGGACCGCCCGCAAGCGGAGGCAGAAGAGGCGCGGGAGC

AAGACCAAGACCAAGCGAAGCAGCCAGAGTGCCACCACCAGCGACGAAAAGGAACACGAACACGAACACGAACACGAACA

CGAAACAACCTGCCTCCCCCCGGGCTTTACCCCCGAGGGCATCCTGGCCTCGATCGACGTCTTTCGGGTCCACGACGAGG

CCGCCCTCCTGGCCACCCTCTACTCCCTGGGGGACCGCATCGGAGAAGCATCAAGCACGAAAGGCGGCCTCCCCGTCCGG

CTGGTCGTCGTCGACAGCATCGCCTTTCACTTCCGGGCGGCCTCCCCCACCGATCCCTCCTACTACCTGCGGCGGACCAA

GACCCTCGCGGGCCTGGCCGGCTACCTGGGGGACCTGGCCCGGAGGCACGGCCTGGCGGTCGTGGCGATCAACCAGATGA

CCACCAGGATGCGCCACGGCCACGGCCACGGCCACGGCCAAAGCAGCAACGGAAGCGGCAGCGGCAGCAGCAACCCCTCC

GACGCGCTGGTGGTCCCGGCCCTCGGCGAGTCCTGGGCCCACGCCACCGCGACGCGCCTGGTCCTGTCGAGGGAGCKGCA

GTGGCAGGGGGGGACCCGCGGAAGGAACGAGAACGAGAACGAGAACCACAACGACTGCGAGAACGACAACGACTACGACC

GGCCCGCCGTGCTCCGGCCGGTGCGGATCTGCTCGCTGGTCAAGTCCTCCCACCGGCCCACGGGACGGGCCCTTTACCGC

ATCCTGGAAGAGGGGATCCGGGACGTGGTCCGCAACAACGACAACAGCAGCAGCAGCAGCAGCAGCAGCAAGCGACCAAG

AACCGGTTAGCCATCGAGGCCCTGCAACGATGGATCCGTTCGGATCGGGTTGCTTGTTAGCTTCGGCACAATACAGTACT

CTGCCGAACCAATACGGTGAACGGCAGAGAACCAACCACCGCCGTTCCACCTGCCGGTGGCAAGACGACAACCAAACAAC

CAACCACTAAAAAACTGCAAGCTAC

>PsnmuV1.4_aug-pasa-abinitio_v2_0067170.1 class=Sequence position=[PsnmuV1.4_scaffold_108-size_153887:9585..11015 (- strand)](http://gbrowse255.tgac.ac.uk/cgi-bin/gb2/gbrowse/maplesod_psnmu_v1_4_gbrowse255?name=PsnmuV1.4_scaffold_108-size_153887:9585..11015) #XRCC3

CGCGGTTGATCCTGTCACGAGCCCGGACGAATTTCATTTCGTTTCGTATGAGGCAATCCTCTTCGACACWACAGCATGGG

GGATCCCTCCCGGTGTATTCGAATGGAAGAAAAACCACTGCTGCTGTTGCGCCGGTGTCTGCCCTAGAACTATTTCGCAT

CGGAGTGAAACGACGTCRAGGAACAGGAATGTTCTCTGGTGGTGGTAACAGCAATGGCAGCAGCAGCAWTAACAGAATCG

GTGGCTTCGAAACTTACAATGAATATTCCGGGAGCAATCGTTCGACGCGAACCTCTTTGTCGTCTTCGGTTTCGGCACCA

CCTCTTTGCCTACTTCAAAATCCGCATCTGTCTTTGCCGATCGGGCTTAGCGAATTGGCAGGGCCCGCTGGTGCAGGAAA

GACACAAATGGCCCTGAGTTTGTGTGCGGATTGCGTTCAATATTCTTGGAGCGACAACARCCACCAAAAGAAGAAGGCAG

TCTACATTCAATTGGGAGGTTCCAGCCGGTTCCTGCAAACCGCGTCGCGGCGGCTTAAGGCTATRCTGGAATCCAGGATA

GCAATGCGTGATCCTTGTGGTGGGGGGATCCATCCTTCCGGTTCTCCCGGTGATGCCACAGATGCAACTGCTCACAACCC

AGCAGCAAGACACAAACGACATCATCATCGTCAACGACAACGACAACAACAACGACAACAACAGCAACCAGATGTCTCGG

AGGACAGGGTGCACGATTGTCTGAGCCGTATAATCGTCCATTGGATTTGCAATTCCGAGGATCTGATGGAGCTTCTCCGC

ACCAAATTGCCTCGATTGCTTCGAATGAACAACCGCACTGTGTCGCTTGTGGTATTGGACGGAATTGCCAACCTGTTTCG

ACTACTGCCAGAGCAGGATCATTTCTGTAGCACTAGAGGCTACGACATTGGCGACAAAAATCCATGGCACCACCGAGCCG

TCACATTTTTCCAAATATCGAATCTTTGCAAAGAGCTATCCTCTGTGTTTGAGGTCCCATTCGTCGTTCTGAATGAGTGC

ACGAGTAGGATATCGAGTGAAAGCACTACGAACTCGCCTGTCTTGGAACCGGCGATGGGACTCGCTTGGTCGCAATGCGT

CAACTGCAGCTTTTTCATTTGTCGGGAATCACGAAATAATTTGAATACCGCCGAGAGSGCGACGAGRACAACAACGTCAA

CGACAATAGCGGAGTCTATTCAGACTTCGGACAGCGTCCAGGTATGCCGTAGAGTGTTGCAATGTCTCAGAGCATCGCAC

ATTTCATCTGAATATTCCAGAGCAGAATTCTGGATTGATCGAAGCGGTGTCCATCCATACGAACATGACTTTGGACAAGA

AAATCAACGACGAAAATACAGTACATAAACCTAGTTTTAGAAATGACAACAGATAAATTATGGATGGAATA

>PSNMU-V1.4_AUG-EV-PASAV3_0063260.1 class=Sequence position=[PsnmuV1.4_scaffold_30-size_255779:77087..78745 (- strand)](http://gbrowse255.tgac.ac.uk/cgi-bin/gb2/gbrowse/maplesod_psnmu_v1_4_gbrowse255?name=PsnmuV1.4_scaffold_30-size_255779:77087..78745) *REC-A*

AAAGAAACGAGCCAGGCTTCATCGGTTTCTTTCAGTCGTCTACACCAGAACCAACCACAAGATGAAATTGTCCCTGTTCT

TCTTCCTCTCGAATCTTCTAATTGCCGATGCCTTTGTGGTTCCCCGGCACCACGAGGCAAACCCGCGGCAATCTCCGGCA

GGGATTCTCCGGCCCCGGTCGCTGGCCCGAGCCGCGAGAAAGAAGGCGGCCTCTCGGGAAGAGGAGGAAGGCAGCGGCAC

CACCGGTGCCGAGGACGCCTCCAGGAAGGCCGCCCTCCGGGGCGTCCTGGGCCAGATCGAGCGGACCTACGGCCGGGGAT

CCGTGGTCCGCCTGGGGGACGCCTCCCACATGGCGGTCGAGGCGACGCCGACCGGCTCGCTGACCCTGGACGTGGCGCTC

GGCGGGGGGGGATACCCGAAGGGGCGGGTGGTGGAGATCTACGGCCCCGAGTCCTCCGGGAAGACGACCCTGGCCCTCCA

CGCGATCGCCGAGTGCCAGAAGAGGGGCGGGACGGCAGCCTTTGTGGACGCCGAGCACGCCCTCGATCCCGTCTACGGTG

CGTTGCGATTGCGATTGCGATTGTGTTGCGTTGCGTGTTGGCATCCATTCGTGGGGTTTGTGTTGCGTTGTGTTGCATCG

GGACTGCCTTTTGCAGCGTTTCGCGTCTTGCGTGGGATTGTATGAGTGACACTCTATAAACATTTCTTTCTCGACTAGGA

TTCACAAACACAATTCTGCTTTCGTTCTGCGCAACGATGTTCGCATTCCAACCAACCGACCAACCCATTGCCGTGTGCCC

CGTGTTGTTGTTTTTTTTCCTTCTCTCTCTCTCTCTCTCTCTCTCTTCCCACAGCCAATTCCCTGGGCATCGACGTCGAC

GACCTGCTGGTGAGCCAGCCCGACTGCGGGGAAATGGCCCTCGACATTGTCGACAAGCTGGTGCGGTCGGCGGCCGTCGA

CGTCATCGTGGTGGACAGCGTCGCCGCCCTGGTCCCGCGGGCCGAGCTCGAGGGGGACATGTCCGACTCGCAGATCGGCC

TGCAGGCCCGGCTCATGTCCAAGGCGATGCGGAAGATCACCGGGTCGCTCGCCCTCAGCAAGTGCACGGTGATTTTTCTG

AACCAGCTGCGCTCCAAGGTCGGGGTCATCTACGGGTCCCCCGAGGTGACTTCGGGGGGGAACGCTCTCAAGTTCTACTC

GTCCGTCCGCCTCGACACCCGCCGGAAGGAGGTCCTCCCCGAGAACGCCGGGATCCGCGTCAAGGTCAAGGTCGTCAAGA

ACAAGGTGGCGGCGCCCTTCCAGATCGTCATGCTCGACATACTCTTCGGGACCGGGATCGATTCGGAGGGGTGCGTCCTC

GACGCGGCCCTCGACCTGGGGATCGTGGAGCGGAGGGGGAGCTGGTTTTCCTACCGGGGGGAGAACCTGGCCCAGGGCCG

GCACAACGCGCTGGAATGCCTCAAGGCCAACGCCGAGCTGTATTCCTCCATGGAGGGGAACGTCCGCACGTCCCTGGCCG

GCCTCGGAAGCGAGGATCCGGAGACCGAAACCGGTGCTTCCGACGACCTTGTGCCCGGTGGGGCTTCCGGTGTGCCCCCG

GTAGAGATTTCGGCGGAGGGATTCGAGTAAGCGGGCGCAACGAATCTCATCGACGTCCG

>PSNMU-V1.4_AUG-EV-PASAV3_0088620.1 class=Sequence position=[PsnmuV1.4_scaffold_46-size_234273:104466..107137 (+ strand)](http://gbrowse255.tgac.ac.uk/cgi-bin/gb2/gbrowse/maplesod_psnmu_v1_4_gbrowse255?name=PsnmuV1.4_scaffold_46-size_234273:104466..107137) *RAD52*

GCGACCAGATTGGTTCCATCGCAGCAAATCGCAACCATCAGGACTAGGACTGCGAGAGAAGTTTGGTGAATCCATTCTCT

CACTAAATCCTGAATATCGCCCTGCGTCGGAGGTTCTTTTTGGGAGTCATGGCATCCTCTACCTATTGCAACACCCGGCA

GATCCGGCACCAACACAACGAGCAACATCAATTTTTACAGCAGCATCAACAATCCGCTTCTTCTTCCCACTGTCTTCCAA

AAGCGAACGCAGCAGCCTCTCCAGTGATGTCAAGAAATACTTCGATAGTGGACGAAAACGCCATTGTCAACGATTACAGA

CCGCCGGGTATAATCTCGTCGCCTTATCACCCCCTCAATGACAACGAAAACAACAAGAAATGCGAGGCTGCTTCCACTTC

AAACAAAAAAGTGCAATGGGTCTACAACCCCATCGACGGGAGTGTTCTGCACGACCACAATGGRAACCCACTGAGCGTGG

ATAGATTGCTCGCTACAAAACCCCTCAGGCACGAACTTTCGACCCGCCCCGGGCCGGGAAACAAAAAGCTGACCTACATT

AGCGGGGACGACGTCAGCAGGACGCTGAACGACATCTTTGGCTTTGACGGCTGGGATCTGGACATCAAGGACGTATCGAA

GGTGGACTCCGAAAGAGATCCTAAAACGGGGAAGCACACCATTGTGTACACGGCCCAGGTCCGATTGACCCACAGGGSGT

CCGGGGCCTACAAGGAAGACTGCGGGGCAGGGGATTCGACCGATCGATCCTATGCCACGGCGACCGCTCACGCGCTGAAG

GCAAGCATCACGGATGCGATGAAGCGGGCGGCTCGCCATTTCGGGGACAAGCTGGGAAACTGTGAGTRCAGARAGATTYT

GTTGTTGTTTGTTTCGTTTCCTGGTGGGGTTCGTGCATTGGCATCGATTTGCTCTGTGTGTTTTACTTTATGTGATGGTG

ACGCTTGATCGTTCTGGATCCATGTCTCGTTGGCATCTAATTACGTTTGTATCTCAACCACACAAAACGCAAYWCATTGC

AACAGCGCTCTATTCCGGGAACTTTAGCATCAACAAGGCTCCGGTCTCGCTGAAGGATGCACTCGACAAGTACGACATTC

ATAGATGCAACACCAAGTTTGGATTTCAGCGGTCCAACAAAAAGCCCAGTTCGTCAGTGGCCAACACAAAGGCAGTCGAT

GCTTCACATATACCCATGCCTCCTCCTCCGGCTCAACAGAATGTGCAGCAGCAGCAACAACAACAAAATATGCACCAAAG

ATATCCCCCTGCTTCGATTGTTTCAAACGAAAAATCTGGAGTTGGAAAGCAAGAAGAGAGCAGTCTTGCTCACCAACATC

AACACCACAATGAGCGAAACTCGCACCGGCCTCAGAATTGCCACCATACCCATCCCAATGCTCCGAGTATTGCCGRGCCG

TCCGATGGTCCTCCTCCTCAGCAACAGCAAAGGACGAGCATCAGTACTCTATCCTTTGGTGCTCCAGCAAACTCCAATTC

CGAGACAAATATACYTGCCCAACAATACCAACTCCAAACGCASCGACAGCAACATCCACCCCAGAAAWCATTTCGAGCAA

ATACTAATGCTCCAAACGTTTCGGGGAYTTCGAACGGTCCTCGGCCGCCGCAGCAGAGCAGAAACCCCAATTTTCCGTCT

TCYAACGGACCCGCACAACCAGAATCTCGTTTCACACCGGAATCGACGAATTCGGCAACCGCTTTGGATCGACCATCCTC

RTCGTATGGGCGGCGCCCCGCCCTCGGATCCATTTCYTCTTCCACCACGAGTGCGGCAACAACAGGCCAATCTTTCGATA

CCAACAACAATCATARCGACAAGAACAGWAGTAACGACGTTCGCAGGGTGTCCTTGGGCAATCAACACCCGCAGCACATG

CGCCAATCGATGCCAGCGCAAAACCACCATTCAAACGCGAGCATAACAAATAATGTTCCTGCGATTAGCCAAACACCATC

GGCCTGTAGCGGTGGYGGCAACCATAACCACTACAATAAACCACRGCACAAACGGCCTCCTCTCGGGAACATAGTACCGA

ATCCCGAATTTGCCCCAAAGCGCCCAAAGCCACAGCACCACAATCCCTACCAACAACCCTACAACAGTGGAAGAAAATCC

ATCTAGAATGGGTAACAAAGTGTTGCATTGGCACCGCGTTCGTAATGCATCTGGAAGATGCCAAAGTTCCACTCCCAACC

TTGGCTTCTTACACAGTGCTTCCCGCAAGTTCTGCCCGTTGATTTTGCATGACGAAGGCAGGCTCCCAAATGAAAAAATT

AAATATGGGTTGATCTCTTAGCGTCTCCGAAAACGCTGTTGCCATTTCCTTTGTAGCACTCTTCATTTGTTAGACGAACT

TATACGACCAAAAATGCTAACAATGGGTCTCAGAGAGCACTATGATTTTAMACGGATCCTAAACACAAAATTGGAGCGAT

TCAAACCATTTTTTGGAACTGAAAYCAACACCAAGATTTTCACCCAAATGCAAACGGGGTGCATTCGTTATGATGTAGTG

CACTTCTCGAAGACTTCTGAAAATTACTTTTTGTKACATTCGTATTGCATTCGACGGAATAATGGTACCATATAGTGAAA

GGAGTCTTGGTGAACAAAAATACTTATCAACG

>PSNMU-V1.4_AUG-EV-PASAV3_0074360.1 class=Sequence position=[PsnmuV1.4_scaffold_358-size_46621:30017..32855 (- strand)](http://gbrowse255.tgac.ac.uk/cgi-bin/gb2/gbrowse/maplesod_psnmu_v1_4_gbrowse255?name=PsnmuV1.4_scaffold_358-size_46621:30017..32855) **#*RAD1* No ERCC4 domain found**

TCGGGAAAAATGGAAAAATTAGACCATCAAGGTTTTGATTACCGGTAGAGACTCGTGATGGTCCTCAGAAGAAAGCGAGC

TGAAGGGTTGAAAAGAATCGAGAAATCGTCTCGGCGTCAAGTTATTGGCACCACTTTTGAAACTGGAACGTATGGCGAAA

GAGGATGCCTCGAACAAATGACGCAAACGATCGCAAGAACAATGGAGAATCAATGATACCAGACGGCCTTTTACCATCGT

ATTTGGCCACTGCTTTTGGAGATATCTACGACAGAGATGGACTTGCGATATTTGGTAGTGGGCTTGGATGGTTGGATCTT

ATGGCCGCATTCGTTCGATTCTATGCAGACACCGAGGAGGGCCATCTTCCCATTGTTCAAGAAGAGGAGAAGGATGCTAC

TGGCCGGATCAAGACAACGAAGAAACCTCTCGTGTTAGTCCTAGGATTGCGCGACGACGAACACGAGACGCTTACTGCGA

TGTTGGAAACGTGGGGCACGCCAGACGAGATGATGCCGACCATCATTACAAACGAGAGTGGACAAGGAAAGGATCGAATG

CAAATGTACCAGCGGGGTGGTGTATTTTGTATAACATCTCGCATACTGATTGTCGACTTACTTGACAACACTCTCTCATC

GAAGGATGTAGATGGCATGCTGATAGGACACGCGGATCACGTAAACAACGATTCAACCGAAGCATTCATAATCCGGATCT

ACACGAGTCAAAAGCGAAGAGATCAGTGCTTCATCAAGGCGTTTACTGACAACCCCGAATATTTGCTCTCTGGATTTGCC

AAAATTGACAAGACACTTAAGGCACTCCAGGTTCGTCACCTGTATCTTTATCCGCGATTTCACGATTCCATTAAAAAAGA

ATTGGAACAAAATACGCCTCACGTACAGGAGCTTCATCAAGATCTATCTCCTTCCATGAAGGACATTCAGAGTGCCATTG

TTGCAGCTGTCCAGACCTGCATGAAAGAACTGAAAAGCTCAACACCTTTTCTCGAGTGGATAGGCGATGATCTTTCCATT

GAGAACTGTGTCACTTCAGCCTTTGACCGCGCGGTTTCTAGGCAATTGGAAAAGGACTGGCATCGTCTGAAACCACAAAC

CAAACAGCTGGTTCAGGACTTGAGAACGTTGAGGGCCCTCTTTGCGTCCTTGATACAATACGACTGTGTTAGCTTTTGGA

AACTGATCTCTTCAATCAAAACCATGAGTGCGGCATCGAGACACCCAGCGATGTGGCTGTTGACACCCGCAGCAGATTTG

CTCTTTCGACGTGCCAAAGAACGAATCTATAAGGTTCATCGTCCAAAACCCACAGAAACTATACCCAATCCAGTGGGTAA

GTTGATACCTGTGTTGGAAGTAAATCCGAAATGGAAACTCCTGAAAAACGTTTTGTTGGAGATCAAAGAAATCGAGTCCA

AGGGTGAGCAATCGAGTACTCTTCGTGAACAACCAGCAACAATCCTAGTTGTTGTTAAAGATGAGCGAACTGTAGATGCG

GTCAAGTCGTTTCTAGTTGATGGGAGAGATAGAACGATGGTGAGTCTGGTTTTGATATCTTTGTTTCGTATTGCAATGTA

TTGTAAAACAATCTGATTTGTGTTGATCTTTTGGAAGACTTTGCGCTGGCACAAGTATCTAGAGCAGTGCAATGATCGAT

CGCGGTCGATCATCAATGGAAAAATGGCGGAAGAAAGTCGTCTTCTTTTAGAGGAAGAAAGTCGAGTGTGGAGAATTCTC

TATGGAAACAAGGGACACAAAAAGAAACCTCAACAAAATGGAAAGGACCGAGCGAGGAAACTCAACGAGGTTCCAGAGTA

TCTCAGAAAGCGACGCAGGATTGCTGTTGAGCAGGGCAGAGGACGAGATATGACTGGAACCAACGAAGATAGGGAAAGGC

AGGCAATTTTAGAGGAAGCAGTAGAAGAGACGGAACAAGACCTAGAAAAATTTCGAAAGACCGCCGAGGAGGAGTTGAAG

GAGGAAGAGGAAGAGGAAGATATAATATTCAATACGATGTTCCAACCGTCATTTATTGAGGAACCAAGAGTGGTCATCAA

GAGCTTATCGGGAATTGATAAGTCTGCTGTCGGATTAGTTCTCGCTGATTTGAACCCCGATTATGTAGTCTTGTACGATT

ACGACATAAGCTTCGTCCGATCTGTGGAAGTCTTTGCAGCATTGAATCCTCTGAAAAAAGAAAATCGCCTCAAGGTTTAT

TTTCTGATGTTTGCGGCAAGTTCCGAGCAAAAGGTTTTTCTTAAAGCCCTGGAGCGCGAACAAAGTGCATTCGAAAGACT

TATTCACCACAAGAAGACGATGCCTCCACCGGTGATACAGGTGGAGGGCACACAGGAAATGCAGCATGCTCTAGCGACCG

GCAGCGTTGCGGGAACATACAGCAACGGGAGCTTGCCCCTGTCGATGGATACGAGAAGGGGAGCTGGGAAATCCAAGGCA

GAAAAACGCGATATTGCGGTGGATGTTAGAGAATTCCGGTCCGCCTTGCCGAGCATTTTACACAGAGGGGGGATGCGAAT

AGCTCCGGTAACGTTGCTCGTTGGAGATTTCGTACTGAGCAATGTGCACTGCGTCGAACGAAAGAGCATCAGTGACTTAT

TTGGTAGTTTCGCAAGCGGTAGATTGTACGTTCTTCCGATTTTACTTCGATCAAAATATCGAATAAACTGTCCATCATCA

TTCGATTAAGTCACATTCAATTTTCCCTAACGAATCTTTATTTCCTATCTATTGTGATTTTTATCCGTTGTGTGAAATAG

ATACAACCAGGCGGAGCAAATGTCGAAATATTACACCTG

>PsnmuV1.4_aug-pasa-abinitio_v2_0123850.1 class=Sequence position=[PsnmuV1.4_scaffold_358-size_46621:29345..32855 (- strand)](http://gbrowse255.tgac.ac.uk/cgi-bin/gb2/gbrowse/maplesod_psnmu_v1_4_gbrowse255?name=PsnmuV1.4_scaffold_358-size_46621:29345..32855) ***RAD1* Corrected gene model V2 AUGUSTUS**

TCGGGAAAAATGGAAAAATTAGACCATCAAGGTTTTGATTACCGGTAGAGACTCGTGATGGTCCTCAGAAGAAAGCGAGC

TGAAGGGTTGAAAAGAATCGAGAAATCGTCTCGGCGTCAAGTTATTGGCACCACTTTTGAAACTGGAACGTATGGCGAAA

GAGGATGCCTCGAACAAATGACGCAAACGATCGCAAGAACAATGGAGAATCAATGATACCAGACGGCCTTTTACCATCGT

ATTTGGCCACTGCTTTTGGAGATATCTACGACAGAGATGGACTTGCGATATTTGGTAGTGGGCTTGGATGGTTGGATCTT

ATGGCCGCATTCGTTCGATTCTATGCAGACACCGAGGAGGGCCATCTTCCCATTGTTCAAGAAGAGGAGAAGGATGCTAC

TGGCCGGATCAAGACAACGAAGAAACCTCTCGTGTTAGTCCTAGGATTGCGCGACGACGAACACGAGACGCTTACTGCGA

TGTTGGAAACGTGGGGCACGCCAGACGAGATGATGCCGACCATCATTACAAACGAGAGTGGACAAGGAAAGGATCGAATG

CAAATGTACCAGCGGGGTGGTGTATTTTGTATAACATCTCGCATACTGATTGTCGACTTACTTGACAACACTCTCTCATC

GAAGGATGTAGATGGCATGCTGATAGGACACGCGGATCACGTAAACAACGATTCAACCGAAGCATTCATAATCCGGATCT

ACACGAGTCAAAAGCGAAGAGATCAGTGCTTCATCAAGGCGTTTACTGACAACCCCGAATATTTGCTCTCTGGATTTGCC

AAAATTGACAAGACACTTAAGGCACTCCAGGTTCGTCACCTGTATCTTTATCCGCGATTTCACGATTCCATTAAAAAAGA

ATTGGAACAAAATACGCCTCACGTACAGGAGCTTCATCAAGATCTATCTCCTTCCATGAAGGACATTCAGAGTGCCATTG

TTGCAGCTGTCCAGACCTGCATGAAAGAACTGAAAAGCTCAACACCTTTTCTCGAGTGGATAGGCGATGATCTTTCCATT

GAGAACTGTGTCACTTCAGCCTTTGACCGCGCGGTTTCTAGGCAATTGGAAAAGGACTGGCATCGTCTGAAACCACAAAC

CAAACAGCTGGTTCAGGACTTGAGAACGTTGAGGGCCCTCTTTGCGTCCTTGATACAATACGACTGTGTTAGCTTTTGGA

AACTGATCTCTTCAATCAAAACCATGAGTGCGGCATCGAGACACCCAGCGATGTGGCTGTTGACACCCGCAGCAGATTTG

CTCTTTCGACGTGCCAAAGAACGAATCTATAAGGTTCATCGTCCAAAACCCACAGAAACTATACCCAATCCAGTGGGTAA

GTTGATACCTGTGTTGGAAGTAAATCCGAAATGGAAACTCCTGAAAAACGTTTTGTTGGAGATCAAAGAAATCGAGTCCA

AGGGTGAGCAATCGAGTACTCTTCGTGAACAACCAGCAACAATCCTAGTTGTTGTTAAAGATGAGCGAACTGTAGATGCG

GTCAAGTCGTTTCTAGTTGATGGGAGAGATAGAACGATGGTGAGTCTGGTTTTGATATCTTTGTTTCGTATTGCAATGTATTGTAAAACAATCTGATTTGTGTTGATCTTTTGGAAGACTTTGCGCTGGCACAAGTATCTAGAGCAGTGCAATGATCGAT

CGCGGTCGATCATCAATGGAAAAATGGCGGAAGAAAGTCGTCTTCTTTTAGAGGAAGAAAGTCGAGTGTGGAGAATTCTC

TATGGAAACAAGGGACACAAAAAGAAACCTCAACAAAATGGAAAGGACCGAGCGAGGAAACTCAACGAGGTTCCAGAGTA

TCTCAGAAAGCGACGCAGGATTGCTGTTGAGCAGGGCAGAGGACGAGATATGACTGGAACCAACGAAGATAGGGAAAGGC

AGGCAATTTTAGAGGAAGCAGTAGAAGAGACGGAACAAGACCTAGAAAAATTTCGAAAGACCGCCGAGGAGGAGTTGAAG

GAGGAAGAGGAAGAGGAAGATATAATATTCAATACGATGTTCCAACCGTCATTTATTGAGGAACCAAGAGTGGTCATCAA

GAGCTTATCGGGAATTGATAAGTCTGCTGTCGGATTAGTTCTCGCTGATTTGAACCCCGATTATGTAGTCTTGTACGATT

ACGACATAAGCTTCGTCCGATCTGTGGAAGTCTTTGCAGCATTGAATCCTCTGAAAAAAGAAAATCGCCTCAAGGTTTAT

TTTCTGATGTTTGCGGCAAGTTCCGAGCAAAAGGTTTTTCTTAAAGCCCTGGAGCGCGAACAAAGTGCATTCGAAAGACT

TATTCACCACAAGAAGACGATGCCTCCACCGGTGATACAGGTGGAGGGCACACAGGAAATGCAGCATGCTCTAGCGACCG

GCAGCGTTGCGGGAACATACAGCAACGGGAGCTTGCCCCTGTCGATGGATACGAGAAGGGGAGCTGGGAAATCCAAGGCA

GAAAAACGCGATATTGCGGTGGATGTTAGAGAATTCCGGTCCGCCTTGCCGAGCATTTTACACAGAGGGGGGATGCGAAT

AGCTCCGGTAACGTTGCTCGTTGGAGATTTCGTACTGAGCAATGTGCACTGCGTCGAACGAAAGAGCATCAGTGACTTAT

TTGGTAGTTTCGCAAGCGGTAGATTGTACGTTCTTCCGATTTTACTTCGATCAAAATATCGAATAAACTGTCCATCATCATTCGATTAAGTCACATTCAATTTTCCCTAACGAATCTTTATTTCCTATCTATTGTGATTTTTATCCGTTGTGTGAAATAGATACAACCAGGCGGAGCAAATGTCGAAATATTACACCTGTCCGTGCCTTTTAATTGAGTTCGATCCGGCAAAGAGTTTCTGTCTCCAGAATCCCAACGATCTTGGGATGGATATAAAAATAGATTCGGTGGGTTCGAGAAAAATTGAACAATTAAGTGGA

GTGACATGTCGAGCTAGGCCCAATTACCCTGTGTTCGATCTGACAGAGTTTCTAACACCCATTTTTTTTCCTTTGGCCAA

AGATCTGCTGCAAAATGGCGACCCTTACGGCTCACTTTCCAAAACTTCGCATTCTTTGGTCGAGGAGCCCCCACGAGACC

CTGAGACTCTTCAAGGCATTAAAGGCGAACCACGAGGAAGTGGATGTTGCGAAAGCGGTCGACGTTGGGAAAAAAGACTC

CCTCGAATCGCTGCTCAAGTCGGACAAGACAGACGAAGGGGGTGACGAGGAGAACGAAGAAGACGAGATCAACGAGGCCG

GACGAGATATGCTACTCCAGCTCCCCGGGGTCACCATCCAGCTTGCCCGCAAGATCATGCACGAATGCGATTGCTTGGCA

GATTTGATAGCCCTGAACCGAGATCAGCTTCGCGCCTTGGCGGGTCCCTCCGTAGGGCAGAAACTGTTTACGTGAGTTGT

TTATTCGCACTGTGTCTTCTAGATTTGGAACTTTTGTATCTGTATTGGCAAAATCAACCATAATTTTCAAC

>PSNMU-V1.4_AUG-EV-PASAV3_0083140.1 class=Sequence position=[PsnmuV1.4_scaffold_41-size_230178:160616..163740 (+ strand)](http://gbrowse255.tgac.ac.uk/cgi-bin/gb2/gbrowse/maplesod_psnmu_v1_4_gbrowse255?name=PsnmuV1.4_scaffold_41-size_230178:160616..163740) *MSH2*

CGCAACGCAACGCAAGCCCACCCCAACGCAGCTCTGGCACCACGCAACACGATCCGCACCGATACAATGTCGAGCAGCAC

CGGCAGCAGCAGCGAAAGCGGAAGGGTGGTTTCGATCGCCGTCCTGGTCGGTTCGTCGCCGCATTCCGGCGGCGGCCCCG

ACGCCACGACCGTTCCGCTGGTCGTCTGGACGCTCATGGAGAAGGCCCGGGCCGCCGCAGGGGAAGCCGGGGCACCGAGC

AAGCACACCCACGACCTGGTCTACTACGAGTTCCTGGACGACACCGCCGGCTGGAACCACCTCGACCGGTTGCTCCTGCA

GCTCTGCGACACGAACGACGAAGACGGCGACACCGACGGCGGCGATTCCCCCGGCCCGCCCGACGTCCACCTGGGGTCGA

CCGAGCGCCTCGAGAAGGACACCCCGGGCAAGCCCGACAAGCCCGGGGCGGCCCGGGCCCGCAAGCTCCTCGGCTCCCTC

GAGCGCTTTCTCGGGGGCCGCCTGGGGTACTCGCCGGACGGAAGCACTCCCGGCAAGGTCCACCGGCACCCCTCGGTACC

GGTGGACGCCTCGAGGGTCGAGGACTCCCTGTCGGCCCTCCTGATCGACGACGAGGGCTGCCGGCTGGCCGTCCGGGGCG

ACGTCAAGCTCTCCAGGCCGGCCGTCCGGCAGGGGCTGGCCCTCTGGCTGCGGTCCCAGGGGCTCTACGGCGAAGCCAAC

CACCACCGAGAAAGCGCCCGGGACCTGGAGGGATCCCTGCGGGTCCGGTCCGGGAGCCTCCGCAGCTGCCTGGCGATGGA

CGCGACGGCCGCCAGGGCCGTCCACCTGCTGCCCCCGGCCAACGAGGGGGAGGCGGTCGTGGTGGGGGGGACCCCCTCCA

CCAGCTCGCTCTGGGGACTCCTCTCGGGGCCCTGCCTGACGGCGATGGGGAGGAAGCGCCTCCGGGTCTGGCTCCGGCAG

CCGCTGGTCGACGCGGGGGCCATCGCGGACCGCCAGGACGCCGTCGGGGCCCTCCTGGCCGGGACGGCCCGGGACTCGCT

CCGGGACGGCCTCCGGCCCCTGGGGGGGAACCGGGGAAACGGCCCCGCGAAACTGGGGCCGGCGCTGGCCCGGTACCGGC

GGGAGGCCGGCGGCGATGACGGCGACGATGCCGATGCCGATGAAACCAATGCGAGCGACGGGACGGCGGCCGTCGGGAGG

ATCGTGGACACCAAAAAGCCCCTCGAGGTCCTCTACCATCTCTACGTCCTGGCCTCGCACCACCTGCCCCACCTGCTGGA

ATGCATGGAGCCCCTCGCGCCAGGGAACGGCGGAGGCGGGCAAGGGTCTTCCCTCCTGGCCGGCCTCTACGAGCAGACCG

CGGCGCTGGCGGGGCAGCTGGAGCGGTCGGTCGGCCTGGCCGAGGCCGTCCTGGACCTGGCCAAGGCCCCCGACGACTTT

CTGGTCCGGGCCGGTTACGACGAGGAGCTCGGGGAGGTCCACCGGGAGCTGGAGGCCGTCCGGTCCGCCGTCAAGGACGA

GCACGAGCGCGTCCAGGACGCCTGGAACGAGGTTTCCCCCGGCAGAGCCTCCCAGGTCCGCCTCGAGCGGGTCGAGGACC

AGACGGCCTGGCAGTTCCGCCTGCCCGACGCGAACGACAGCAAGAACGTCCGGGAGGCGGGGCGGAAGATCCAGGTCCAC

CGGATCCTCAAGAACGGCGTCTACTTCTCCAGCCTGGAGCTCCGGGAGCTCTCGGCCCGGTACCGGGACCTCCGGTCCGA

GCACGCACGCCTGTCCCGGAGGATCGTCCGGGACGCCATGGCCATCGCGGCGACCTACGCGGGGGTCGTCGAGCGGGTGG

CGGACACCATCGGGACCCTCGACGTCTTTTGCGCACTGGCCCACACGGCTGCCATGAGCCCCCACGGGTACTGCCGGCCC

ACCGTCACCGACAGCGATGAGGACGGCGCCGGGATCCGGCTGAGAGAGGCCCGGCACCCCTGCGTCGAGCTCCAGGAGAA

CATGGAGTACATCCCCAACGACATCGACCTGACCTTCCCGGGGGCGCAGCTCCTGGTGAGCGGCCCCAACATGGGGGGAA

AGAGCGTCTACATCCGGTCGCTGGGGGCCATCGTCTGCATGGCACAGATCGGGAGCTACGTCCCCTGCGAGTCGGCGACG

ATCAACGTCTGCCACTCCATCCTCGCCCGGGTCGGCGCCGGGGACCTCCAGGAGCGGGGGATCAGCACCTTTATGGCGGA

AATGCTCGAGTCGTCGTCCATGCTCCGGGCCGCCACCAAGCGCAGCCTCGTCATCATCGACGAGCTGGGCCGCGGGACCT

CAACCTTTGACGGGTACGGACTGGCCAGGGCCATCCTGGAGTACCTGAGGGACCGCGTCGGCTGCATGGTCGTCTTTGCG

ACGCACTTCCACGAGCTAACCCACCTCGAGGGGGTCAAGAACTGCCACGTGACGGCCCGCAAGGGGAGCCAGGGACTGAC

CTTTCTCTACAAGGTCCGTCCCGGCCCGTGCCTCGAGTCCTTCGGGATCCAGGTGGCCGAGATGGCCCACGTCCCGAGGG

CCGTCATCGAGGACGCCAGGAAACGGGCCAGGGAGCTCGAGCGCTTTGTCGATGCCGGGGCCGAGGGGGGAGCGGCCGCC

GGTGGCGACGCCAAGGCGGCCCTCCGGCGGTTTGCCGGGCTGGACCTCCCGTCGGTGGTGGAGGAGCTCTCCACCCTCGC

CCCGGAAGCGAAACGGGCCAGGCTCCTGTCGCTCGTGTCGCCGTGAGCGAGGAAACGAAGGTGCAGATGCAGCGCAGCAG

CCCCTTTCCGGAACGCGGGGCCCGCTGGAGCAGCGCCTTCCGCCTGTGGCCCGGACCCGGCGGTTGTTTCTGTGCGGGAC

AGCCGGGGGGATTTCCCGGCCCACAAACAAAGACGGACTCGGGTTTTGATGGAACGAATCAAAATCAATCCATCAATCAA

TCGCCCGAAAGCAATCATCGTACGGTACTACCGTCCTCATACGTGGTAAAGTACATACTACAGTAAAGTAGAAGAATAAT

TAGTGATCCTAGTACAAAAACCTACAGTACGAGCTACTCCACTTCGTACGGTATGATGAGAGTCAAATAAATATCTTTCG

AGATC

>PSNMU-V1.4_AUG-EV-PASAV3_0084190.1 class=Sequence position=[PsnmuV1.4_scaffold_42-size_240916:172036..179299 (+ strand)](http://gbrowse255.tgac.ac.uk/cgi-bin/gb2/gbrowse/maplesod_psnmu_v1_4_gbrowse255?name=PsnmuV1.4_scaffold_42-size_240916:172036..179299) *MSH6*

GCTGTGTTSGGTGTTTGGTTCGGGTGGATCGATCGACAATGGTATTGCACCCTTTGTAACAAAACACTGAACACAGACGC

GTTTGTCAATCCGTACTATAATTATCGTTTTCAACATTGTCTTCGTCGTTGCTGATACCACTCATCGTGCCCAATACAAT

TGATTCCACTCAGTCTCTAATCCATTTTCGATTCGGCGCCATCGAAAAAAAGCAATCCGAGCTGCTATATATCTTTTTCC

CATTCGTTCTTGGGGTTGGTGGCTCACCATTCTTATTCCATCGAGGCATCGGCACAGATACGAGCACACCGCAAATCGCA

TGCAACGGAACCACACATCCAAGCCCCTGGTATATAATATGCCGCTGTGGTTCGACCTACCTACGGGAGCGGTTGTGTTG

CACTGGATTTCGTTGCGCTGCGCTGCACGGGGTTGTCCTCCGCCCCCCCCCCCCTTCCGTTCCACTAGCTGCCGTCGTTC

TGCGTTGTTCTCGCGAGCCTGTCGAATCCCGGGTGTTCCTGGACCAGCCTGGCGAGGTGCCTCTGCCTCGGGTCGCGCTT

TCGGTTCCTGAGGACCTTTTTCTGTTTTGCCTCGGCGGGAAAGAGCGAGGCCGCCAGCAGAGCCGCGAGGAGGAAGAGGA

AGAAACTGCGGTTCGGAGCGATCATGGTTKTGGTGGTGTCTTGTCTTCTGTTGTCTTGCGTTGTGAGGAGTGGTGGTGGC

GTTTRTTGAAGCGCGGCGTGGTACAAGCGACGAGATCCGACACGAGTGGTGTGTGTTTGTGTGTGCAAAAGGAAAGAAAT

AATAACAATGTGGGAACAATCGGATGTTTTTTAAAAAAAACAAAAAAATTGTGGAGGACTATCGCAAAAAAAACGTACGA

GCACGAACTGCCAATCTCTGGTGGCGCAACGCAAAACACGGTGTGCAGAGCCCCATCCATCGGTGCGGAGTGAACACGAG

TCAATCCAATTCAAAACCAGGATCATTATGGGTTGCTCGTGCCGGATCCGGCGGGTGTTTTGGTTCGAATCGAATCGAGT

CAATCGAGTCAATCCTCCCTTCCTCGGATTCGGATCGGATTCATTCGAATCAAATAATCCATCGTAGAGGGACCGTCCGT

GCACCCATCCGTCCGCGGGGCCTTGGTCGATCGGTTCGCGCCAAAACTCGTCGTACGAATGCATGCCAGATAGACAGAAC

AGACGCTCCGAGTGGGAATTTCCTTCCGGCGAGGGAAAATGACGGTCGAAGCGCGCCGGTATGGTACGTCGACGGCGGCA

TCGGTGCCGTATCGTCTGGTACGCTACGGGCGTGAGACTCGACTAGATGACCGTACGAGTACGGTACTACGGTAGGACTG

GCATGACAAAATGATGATGAATGATTCCTTGTCGCGCCGCGAACGCAACGAGAGAGGTGCAAAAGGGATTCCCCCGAACC

AGACTCTTTTACCGTATGAATCAACGAAGGACAAGACAACATAGTACGTTACCAAGTACGGTACGAGCTACGGTACTACG

ATACGATTGCTCGTTGCTTACCGTACGGCACTTTCCTTGTCCATTTTCAATGCATTGATTTTGYTTCCTAACGTCGGCAT

CCATTTCGAAGGCATCCATCTCGAAAGCATCGSTGGATACGTCCTCTCTATTTGACGAAGCGAGTCCGAAGAGAGCCGAG

TTGTTAACGAAACCGATCCCAAGGCGAGAACGACTCCAATTGCATATCACACAAACGAAACAAGCACCAACACCGTCTGC

GCAWCAACCTTTGACAACCTTCGCAACGGCAACCGTCGGCGACAATGGCGCCACCGAGCAGCAGCAAGAAGAAGGGCCTG

AAACAGGGCAGTCTCTTTTCCTTTTTTTCCAAGTCTCCCGCACCGAAAAAGGCATCGGCATCGGCATCGGCTGCCGCTCC

TGCCAATGCAAAGGCCAAGGGAAAAGGTGCTTCCGGCAAGCCCGAAAGCAGCAAGAAGCAGAACCCCACCACCACCAACA

ACAACAACAGCAACAGCAATGACAATGGCAAGGAACGCAAAGCAACACCGACAAGGCAGGCACCCGCCCCTCCGTCGAGA

GCAGGGTCGTCCGCGCCAACACCCACGCCCACCCCGTCGTCCTTCGGGCGGGTGTCCGGGAACACGCTGTGGAGAAAACT

CCGAGAGGGGTCCCGGGTGGGGATCTACTGGAAGGACGACAGGTGCTACTATCCCTGCACGGTCGAGAAGCGGGCTCCGA

GCGACAAGGACGGGAAGGCCTCCTCGCGGTTCTTTGTCCGCTACGACGACGGGGAGAGCGAGTGGACGGACATGGCCACC

GAGAAGCTGCGGTGGTGCGAAGACGACGACGAATTCGAGCGAGAGGAAGAGAACGAGAACGAGAACGAGGAGGAGGAAGA

CGTCCCGGAAGCATCCGGTGGTGCCGGCAGCGGCAGGAGAAGCAACCGCAACAATCACAGCAGCAGCAACAAGCGACGAA

GAAGCATCCAGGAGAGCGACGACGACGAGGAAGAAGAAGAATGGCAGGACCCGGGAGAGGACGAAAGCGACGACGACGAC

GGGAGTGTCTTTGAAGCCGCCGTGGACGAGGAAGAAGACAACGAAGACGAACTGGCGGAGGAAGACAACTGGATGGTCAC

GGACGACGAGAACGAGAACGAGGACGAGGACCGTCCTTCCGCAAAGAAGAACAAGAGGAAGAGAAAGACCACCAAGAGCA

AAGCCGGGGGCACCCTTACGGTGACCCGGCACAACGACAACTCCTCGACGCCACCGCCCTCCAAGCGGCCGAAACCGGGC

ACTGGCACTGGTAGCAGCGGCGGTCCTTCGGCCACCAACACCAACACCGGTAGCAACGGCAACGCCTTCAAGACCCCCCT

CAAGCAATTCTCCCACACGGTGTCCCCGCAGACGGCCTCGTCCCAGAAATCAAAAACAAAGACCGCGAGGCCCGCAACCA

CGCCCTCTCCGTCCCACCCCCACAAAAACCTCCAGCTCTCGTCGCCGTCGCCGTCGCCGTCGCCGTCGCCCCCCACCTCC

CATCGCACCGGCAACAAGCGGCTCAACCCCCACCTCCCTTCCGAGGCCCTTCCCTGCAACACCGAAAAGGGGGCCCTCAA

CGTCCGCGGGGCCCACGTGCACAACCACCTCGGCTTTTTGCGGGTTCCCAGGGACGCCATGGGCCGCTACMGGGACCACC

CCGACTACGACTGCCGGACGCTCAGGGTGGACGAGGCCGAGTGGAAAAAGGTCACCGGGTCCGACATGACGGCGGCCGTC

AAGCAGTGGTGGAAACTCAAGTCCCAGTACTTCGACACGGTGCTCCTCTTCAAGACGGGAAAGTTCTACGAGATGTTCCA

CACGGACGCCGACGTCGGGGTGGAGGTCCTGGGGTTCATCTACATGAAGGGCAAGTCGGCCCACTCGGGCTTYCCGGAGA

TTTCCTACGGGCAGTTCGCCGACAAGCTGGTCCGTGCCGGCTACAAGGTGGCGAGGGTCGAGCAGACGGAAACCCCCGAC

ATGCTCAAGTCCCGAAAGGCCAGGACGCCGCGGTCKCAGAAAAAGCCCCAGGTCGTCAACCGGGAGGTCTGCAGCGTCAT

GTCGCTCGGGACCAGGACCTTCTGCGCCCTCGACGACAAGTCGGCACTGCTGGCGTCGGAGGCCAACGGAACGGCCCACG

AGTCCTGCGGTCCGCTCCTGGCCATCCGGGAAACCCTCCTCGGCAGCGGTGCCGAAGGAGGAGAAACCAGCGGCGAGGGC

GACAACGACGACAACGACAACAACGACAACCACGACGACGACGACAACGACGATGGGGGCGTCCGCCCCGTCTGCGAGTA

CGGCATCACGGTGGTGGACGCGGTCCGGGGCACCCTCACCCTGGGCATGTTTGCCGACGACGTCCTCCGCAACCGCATGC

GGACCCTCCTGGCCTGCTTCGAGCCGTCCGAGATCCTCTACCAGCCCAGCGGMRAGGCCTCGGGGGAGCTCGTCTCCCTC

CTCAAGGCCCACCGGGCCGGGACCCGCAAGCCCTGCCGCCTGGAGGTCGTCCGGACGGAGGAGTCCTTCCCGAAGTCGAC

CGCCCTCGACGAGAACCACGCCCGGGACCTRGAGCGGGGGACCGGGCCCCACCGGACCGTCCGGCCCTGGGACCCGGAGG

AAACCCTCGAGGAGCTCCAYCGGAGGGCCTACTACCCCCGGGCCTCCCGGAGCAAGACAACCCCCCATGGGGGCAAACCC

AGCGTGGCCCGGTGGCCCGCGGTCCTCCGCATGGTGGTCGAGGGMRGGGCCGAGCTCTGCCTCTCGAGCCTGGGGGCTGC

CCTTTTCTACCTGCAGCGGAACCTCATCGACCGAGAAATCCTCAGCATGGGGATCGTCCGGGCCTACATCCCGCAGGAAA

GCAAAACCATCGCCCGCAGCGAGAACAAGAGCGGAAGCGAAGCCCCGGCCGGAACGGACGGCTTTTGGTCCACGCAGCAG

CAGCAGCAGCAGCAACTGCAGCAGCCGGTGGAGGTGTCCCAGGACGAGAGCGTCCTGGACGGCTTCGAGGAGAACCCTTT

GTTCAAGATTCCGTCGGCAACCCGTGCCCACACACCGGATTCCGGCGCATCGGAAGAAGCAAAGGCCATTGCCCGCGCCG

AGGAACAGATCACCCACATGTCCCTGGACGGGACCACCCTCCACAACCTCGAGATCCTGACCAACGCGGTCGATTTCAAG

ACAACCGGGAGCCTATGGAGCAAGGTCAACTCCACCAAGAGCCCCCACGGGGCRCGGATGCTCCGGGCCTGGCTCCTCCG

GCCGCTCTTCCAGAAGGCCGACATCGAACGCCGGGCCGATGCCGTCCAGGAACTGGCGGGCGGSGCGGGGGCCCTGGCCC

TCCGGGAGGCCCGGGCCAGCGTGCTGGGAAAGATCGGGGACCTCGACCGGCTCCTCAGCCGCGTCCACAGCATGAGCGTG

GGGGTCTCGGCGGGGGAAGCCGGTGCGTCGGGCCCCGGCGGGGCCATCGAAGGGGCCGGCGAGGGCTGCGCCTCCCACGA

GTACCACCCCAACGAGCGTGCCGTCCTGTACGAGGGCCCGGCCTACACCAAGCGCAAGGTGACGGACTTCAAGAACCTGC

TGAAGGGCCTCCAAAAGGCCTGCCAGATCCCGGAGATCTTTGCCGACCTGGACCTCGACCCGGGCTGTCTGTTGTACAAG

CTCGTCCGACAGGGCTGCGACGGCGGCCTGTTTCCGGACATGGTGGAGTCCCTGGATTGGTTCTTTGAGAACTTTGATTG

CGACAAGGCCGCCAGCGGACAGTTCGTCCCCGGCCGGGGGACCGATCCCATCTACGACGAGGCCTGCGACACGATCGACC

GGATCGAGGCGGACCTCGAGAGCTACAAGCAAGAAATGATCCGGGAGGAACTCACGCCGCGACACGTCGCCGGCCGGGCC

TGGAAATACATCAACACCAAGCCGGACTCCAAGGACAAGTACCTCATCGAGCTCCCGGTCACCGTGAAGGTCCCCCACGA

CTTCCACGTCAAGGCCAAGCGGGGGTCCGGCCAGAAACAGGTCAACAAGTACCGGACCCCCTTGGTGGCAGGACTCGTGT

CCGAGCTCGAGCGGGCCCTGGAGRTCCAGCAAACCCGCCGGGCCAAGGGGATGCAGATCATCTTTGCCCGGTTCGACKCC

CAGCGATCCCTGTGGGCCGCGGCCGCCCACGCCACGGCGATCCTCGACGCACTCGGATCGCTGGCCGTCCTGGCGGGGAG

CCAAGGCTACTCCCGGCCGGAGATCCTCTCCTGCGATGCAGGMACGCAGCCGAGCCTGAGCATCGTCCAGGGCCGGCACC

CGGTGGTGGARAACACCTTCCACAGCTCGGAATTCATTCCCAACGATCTCGAGCTCGGGCCCGGGGAAACGGAGGCGGGC

AAGCCGGCGCGGTTGCTCCTGCTTTCCGGTACGTCTTGTGTGGTTCTCTGTGTGTGTGCCTCGTTGCTCTGGTTCTTGTT

CTCTCTCTTTCTTTGCCGATAGATGTGGRAAGCAACAACGTTTGTTTTGGGTTCTTTTGTGGTTCTGATCCTTTTTGTTT

CTCGTCTCCYTCTCATCCGCACAGGTCCCAACATGGGTGGTAAATCGACCATGCTGCGCCAGACCTGTCTCATCTCGATC

CTTGCCCAGATCGGGAGTTATGTACCCGCAGAGAGCTGCCAGCTGACGCCGATCGACTGCATATTTACTCGCCTCGGAGC

ATCGGATCGCATTCTTCTCGGCCAATCGACGTTTTTTGTGGAGGTAAGAAGAACGCCGAACCAAAATTGCGTRTTCTCTT

TGACTTGGAACCTCTTTCCTGTGCGTGACCCAATTGTTCTACAAAAAACAGAGGTCTTACCAAAYATTTCTTTCCTTCTT

CTCCCTCCTTCAGCTGGCGGAGACCGCAGCAGCTCTCCGAGGGGCAACAAGGAGAAGCTTGGTGATCATGGGTGAGTCGG

CTTKAAAACAACCAAATTCGTTGCTTGCCGTTTCTGATGGCCTTGGCCAAATCTYACAAACTACTCCGTGCTTTTGTTTG

CAGACGAACTCGGAAGGGGAACCAGCACATTCGACGGGACTGCAATTGCCAGCGCCACCGTRAAGCACCTCGTGGAACGC

AGCCAATGCCTGAGTCTGTTCGCTACGCACTACCACTCTCTCTTGGACGAATGGAAGAACGCCCCTAACGTSAGACTCGG

TCACATGCAATGCATCGTCGACGACGAAACCGATGCTGGTTCCTCGATCGGCAACAGCAATATTACCTTCCTTTACACAC

TGGGACCCGGGGCGTGTCCAAGGGTACGTTGCCATTKCATTTGCTTGCGTCGAGTTTTGGGTTGATATTGGAGATGAATT

GGTGGCTCACTCACCTTTTTTGGTTTCGTCCGCTTTTCTCAGTCCTTTGGAATCAACGTCGCAAAACTAGCGGGCCTRCC

AAACGAAGTCCTTGCGAACGCAAAACGGGTCAGCGCGGAGTTCGAAGAAGAGATGGCTTGTGGTGGCAGTGGGGCAGAGG

CAAACTCGAACGAGGACGACGCCAAAGAAAAGATGCAGAATATTTTAAAACTAATCGCTGACGAAAATTACAGCGATCTT

CTAACCCTCTGGCAGAGTGTCCAGGCAAAATCCGATTGATTCGAATAATACATCCTACACCAWGGAATCCTGATTTTTAA

AAATATATTCGTTTCTCTCATCGTTGTCTGGAGTAGCGCACTCAAATACAAAGGGCTAGTGAAA

>PSNMU-V1.4_AUG-EV-PASAV3_0125040.1 class=Sequence position=[PsnmuV1.4_scaffold_98-size_175496:95026..99218 (+ strand)](http://gbrowse255.tgac.ac.uk/cgi-bin/gb2/gbrowse/maplesod_psnmu_v1_4_gbrowse255?name=PsnmuV1.4_scaffold_98-size_175496:95026..99218) *MLH1*

CTCTATGAAGTATTGAATGGCTTTTTGAATTCGTTTTAGACTGTGTTGCGGTACGCAGGTCTTTCGCGATGGTGCAGTGG

TTGCAGCAACACGTATCTTCTGTGAGCGCAAGATCGTAACAGTTTCTGCTCTGCTTGATGTTTTGATTTTGACCTGCGAG

TATGAACTCACCTGTTTTCTGGCTACAGGGGCAATAGTATTGTTGTTTCCTGAGGTTTGGAAAAATTGGAATGAGTGCGA

AAAAGAAGCGCGCTACTCCACCCACATTTTTTATTTTTCGGCCAGTACACGTACAAGTGCCTGGTACAAGTTGCGATTCT

GAAAATGAGGACCCTACCGTAGTGAAGATGTGAAAGCAGCTTTCCCAGCATTGTTTGACGAAGACACATAAAATAATAGA

AAAAGTAAGCAACAGCCGAAGCAAAGATTAGAACACAAATGTCAGCAAGACTCTTCTAGCGAAAGAACGACGTGATGGAA

TCTTCCAACGACGAAGTGACGAAAGCAGGTGCAGATAAAAATACAGCTGGGTCAGCAACAACAGATCGACGCCAGCAGTT

GTCACAGACCAAAATCCCTCCGAAACGACGACCAAAGATTGCGGTCCTCCCTCGAGCTGTGGTGGATCAGATCGCCGCGG

GAGAGGTCGTCCAGCGCCCCGTCTCGGTAATCAAGGAACTATTGGAAAACTCGCTCGATGCCGGGGCTACCCAAATTGTG

ATTCAGTACGACGGGCTCTCGAAGTTTTCCATTGCCGACAACGGGCGCGGAATCCCCAGTGCCGATCTGAACCTCTTGTG

CACTAGACACGCCACCAGCAAGCTTGCCTCCGTAGATGACTTCTCCGACCTTTCGACCTTTGGGTTTCGGGGGGAGGCCC

TGGCGTCCACCAGCATGGTCAGCCGGCTCCTGACGGTAGTGACGCGCCTCAGGGTCGGGGAGTACGACGACGACGACGAG

AGTGCCTCGGTGGTTGCTTACGCTCAATCCTACCAGCACGGAAAGCCCCTCCAACTCAAACCAAAGCCGTGCGCTCGAAA

AGTGGGAACCACCATAACGGTGAACGACCTTTTCTACAACGTCCATCATCGGCAAAAGGCCTTTTCGAATTCCAAGCGAG

AGGCGGAAGAATACGGACGCATCCACAAGCTAATCCAGGACTACGCGGTTCACTATCCGACCGTCGGGTTCGTTTGTCAG

CGACAGATGGCCACCAGAGCGAAAGCAAAAGCCAACAAGCAGCTCGTTGTAGACTGCAACACGGGGCAGATCCCGGCCGT

CAGGGCCTTGCTACAGAGCCGTGCCTCGGCGGCAAAACTGGCGGCCGCACAAAAAACCAACAAAGACAATTCTGGTCAGA

AGGCAAAGGTCGTCGAAGCCACCAAACAAATCTTGACACACGTCCTAGGATCGAATCTCGCGAATCATTTATTGTATATG

GAATGCAGCAGCTCGGACAACGACAAGGTTCCCACTGAAAACGACTGTGGAAATGGGAGTGGCAATACCAAAGAAAACGC

CGACAGATACAAATTCCGCTACGCTGCCGAAATCTACTTTACGACTCCAACCTACAATAGTGTCGGAGGTGGTGGCAGCG

CCAGGAAGGGAAATAGCAGCAAACAACAAAACACCAAGACGACTCAGGGGAAATTCGTTTTGTTTCTGAACCACCGCCTC

GTCGACCTTCCACCCCTCAAGCGGGCTCTGGAGGACATTTATGCCGATCTGGGCAATCCCAATCGCAGCAAATCCAGCAC

CAACAACAGCAACGTTGGGAAACCCGTCTTGGTGGTCAACCTGAGCATTCCAGGATGCCAGGTCGACGTAAACGTTCATC

CGTCCAAGCGACAGGTGGCCTTGATGTACCAGGAAGATGTGATCAGGGATCTAGCTGTGAACGTAAAGGAACGCCTCGAA

GACCACGGGCAGGCCTTTTTGGCTCAATCGGTGGCCACGCCCGCCGCCAAAAAAGCGAATGTTCGTAATCCATACGCCAA

AACTCCAAGCAGCCAGAAGCGAAAACGATCGTCCCCCAACAAAGAATCATCAGAGGAGGCGGTCGACATTGACGATAAAG

AGGAGATTGAAACCATGAGCCACTGCGAGAACGACATCGCCGAGGAAAAGAAACGTTCCAAAAGAGACGAAAACTTCGAT

TCTCCTCCTCCACATGAGAATGCTCGAGGGAAGATCGATGGGAAGAAGCGGAATCCTCGTAGCGGCAGCAGCGAAAAGAA

AATAGCACCGTCCAAGCTTGTTCGAACGAACTTGGCTGCCAGGGCAGGAGCTATAGAGCCATTTCTGGTCTCCACACAAG

GCGCGTCGCAACTATCTCAATCACAGTCGACCCAAGATAACAATGATGAAAACGATAATCCTTCGTCATCTCAACGACCA

CTATCCCAACCAGAGCAATCGTCTATGGTCCACATTCCTCCTTGCCCCCTTGCGAATCCACTGACCTTGAAAGACATTGA

TATAACGCAACCAGGAGCCTTTGCTTTAATACTGAAGCAAGAAAAATGCACCTGTCCGACCGATGTGATTGACATGAGCC

GACGTACCGTGTTCGTACCAAACACGGTGGTCCGGCCTAAGCGCGTCGTTCCGACCCCCTCCAAGTACACATCGATCGCA

TCCCTCCGCAAGCGCGTGGGCAAACAGCAATGCCACGAAACGACAAAACGGATACGGACGGCCTTTTTCATGGGCGTCCT

GTCGCACCAGCGGTCGCTGGTCCAGTGTGGCGAAGAAATGGTGATGATCAACCACTTTGAGCTCGCGAAGGAGCTCTTCT

ACCAGCTAGCGCTCGCGAGATTCAATGGTGGCGCCAAACTAGCTCGACTGGGGGGCGGTGGCATATGCGTACAGACCGTG

GTAGCACAGGCTTTGCAACACGAAGACGAACTGACACTAGCTAGTAGCACCAAAGCCACAAGTAAAACAAGCGTTAACAA

TGGTGCGAGTTGCAGCGACATGCTGCAGGTTAACGACTCGAATCAGAACCTAGCAGAACACATCGCGGCACGCCTGGTAG

AGAGTTCTGCGATGCTTGAAGAGTACTTTTCAATACGCATCGAGCTCCAACCGTCTATCGGCGGAAACGACATGAAAAGT

GATGATAACAAAGATGGAAAGAGCGATCCTTGCGACTATGACCATCGCCATGAAAATGCTGGCAACGCAATTTTAACCGG

TCTCCCAGTATTGCTGGACGGTCACTCTCCACAACCTCGTAAGTGCTTTCGAAATGAAACAACTTTGTTCGAACGTTATT

ATATTGTGTCGTCTTCGCTAAAACTTCCTCCCGCCTACACCAGACGCATTGCCCATTTTTTTGCTTCGACTTGCAACCCA

GGTCGACTGGACCAGAGAAAAGCACTGTTTCCACGGAGTTTGTAAAGAAATCGGAAATTTTTATGCTATGTTGCCTAGTG

AATGGGGCGACCTAAACCCCTACGTCCAGCACACAATCTTTCCTGCACTTTCGTACTTGTTGTTGCCATCAAAGAATCAA

GCAAGCAATGGCTATTTCACGGCTATGACCAAACTGTCCACCCTGTACAAGGTGTTCGAGCGATAAGTAACTTTTCCTAC

CTCCGTCCCACCCCCTTTTTGGCAAAACATTGGAATACCACTCTCTGAACCCGTGCAATGCAGTGAAAATCCTACACAAA

ATAAGATTAAAATGTAGGAAACAGTAAAAGATTTTTTTCATGTTTCTTCCCGCATCATTTCCAAGAACATATTAACCATC

TCCAACCACAAAGTCAAATCATGGCATTTACCATGATGGATGATTGGTGGTGACAAGAACATATCTGAAACTACGACTTG

CACACCTTGAGCTAATTGCAGGAGTCTCCAAAAATGATACTAGTGGCAGTGTTTCCAGTAGTGACATTAATGGTTCTTTT

GGCATTGTGTATCATTCATCCTACTCTAATTTTTCCTTTACTAATAACATTCAAGAACTCCTTCGCCAAGACCTGACTCA

TCTGATCTCAAGCAAGAGAGAAGGATGGAACCATATATATCACCAAAGCCCCTCGTTGGAACTACATGCATCATATTGTT

GGATGCAGTTTTGTTGTTTTTACCAAAGAATCCACTCCACAACCTCAGGGCTCTAATGGTGAAGATCATGAATGTCTTTG

TCTTGAATGGTATACTAAATATTGATCAATCTG

>PSNMU-V1.4_AUG-EV-PASAV3_0117080.1 class=Sequence position=[PsnmuV1.4_scaffold_81-size_176161:137837..142265 (- strand)](http://gbrowse255.tgac.ac.uk/cgi-bin/gb2/gbrowse/maplesod_psnmu_v1_4_gbrowse255?name=PsnmuV1.4_scaffold_81-size_176161:137837..142265) *PMS1*

GCCGATCCGAAGAATAGACCGCACGGAGCGGCGCGGCATGGACGCTGCACAACACAACCCAGCGCGCCAAGAGAAAAAGA

ACATACTCGCTGCGAGTACAAGTACGCATTCTGCTCGACGACGAGCATAGCACAGCACAGCACAGCACAGCACAGCATTG

CATTGCAGCAGAACACGGGATAGCACGTTGCATTTGCACCATTTACGGCCCGCCCCGGAACCATGGACGATTCGGACCAC

CACGAGCCGCGACAGAAAACACAAGGAGGGCTGCTGCAAGCTACAGCTGCTGCTGCTGCTGCTGCTCCTTCTGCCTGCTC

GCAAAAGCCGCAAGAACCACCGCCGCTGCCGGAGACMTCGGAAATCCAGCGCCTCGACGAGCACACCGTGCGACGGATCA

CGGCAGAGCAGGCGATCTCCGATCTGACCAGTATCGTCAAGGAATTGTTGGACAATGCACTGGATGCGGAGAGCACGACC

ATAAAGAGTGAGTGATTGTGCTTTGTTTTGTCGTACAACGAAATGCGGTACAAAACCAATTGGATGGGCCATTTTGCTTT

GCTTCGCTTTCTCTCTGAGGCAAACAATTAGTTTTCGGTCTGCTCTCATCGAAATCGATGTTGTTTCATTCCGCGCTCTT

CGATCCCGTACCATTCATTGCATTGCATTGCATKCCCTTCCGTAGTCCGCTTGTTCGGGCAGGGACTCGAGATCATCGAG

GTGTCCGACGACGGCACCGGTGTGCCGGTGGGATCCCTCCCCTACCTGGCCACGAGGCACGCCACGTCCAAGATCAAAAC

CCTGGGTGACATCTACGAGGGGACCGGGATGACCATGGGATTCCGGGGCGAGGCACTCTTCGCCATGGCCTGCGTCAGCG

ATTCGCTGGTGGTCGCCTCCCGCACCGAGGACGACGAGCTGGCCACCAAAGTCGTCTACGGAACAGACGGGCWGCCGGTG

GCGGGGGATTCGGGTGCGGACGCGGATGCGGATGCAACCCCCGGCACCCACCACGCGGAATCTCCTTCGTTTGCCCCCRA

GCCACGGCAGCCGGGGGGACAAACCTTTCCGAAACTCGCCCGAAAGGTGGGAACGACCGTCGCGGTGGTAAAGCCCTTCG

GGAACCTCCCGGCCCGTCGGGCGGACCTGGTCCGGAGGATCAAGCAGGAGCGCACCAAGCTCTTCAAGCTGGTGGAATCC

TACGGGGTGTTCAATGTCGGCGTCGGCATTCGGCTCATCGACATGGCCAAGAGCAGCGGCGGCGCGGTGCACCGGGAGGA

AGTCGGCCTGGCTACCAGTTCGTCCTCCCGGACCCTCGAGGAGACGGCGAGCACCTTGCTGGGGCCGGCCTTTGTCAAGG

GGATGACAACGGTGGAGGTCTCGCTGGAATCCTTCTTTCGGAAAACGGAGGGGAGCCGGAAGAACGACGACGGGACGTGC

TACAACTGGGGGATCCGGGGCCTGGTGTCACGGGAGCACTCGCAGCTGGCCGCCAAGCAGCGGCAGCACGGGGGGAAACA

CGGCAGCGGCAGCCACCAGGCCCGTGTCCGGTCCGTCCAGTACTACAGCATCAACGGCCGTGTGGTGGAGCTTCCGGGAG

TGACGGCCCTTCTCAAGAAACTGTGGACGGAATCCTTGTCGTCGGCAAAGGCGTCGCTGTCGTCGKCTTCGGGTCAGTAY

AAGCCCCCCAAGAAGCCCTCGGCGATTCTGGCGTTCACGCTGCCGAACGATGCCTTCGACATCAACCTCTCRCCCGACAA

GAAGACCGTCTTGTTCACCCACGGGGACGACCTCCTGGAGATGGTTCGGGAACACGTCTCGGGGCTCTGGGGCGGATCGG

CCCGATCGTCGGCCGTCTTTGCCGAGGGTTCTCCGGAAGCAAACCTCGGTCGGAAARCAGCGGCACGGGCCATCGGAAGC

GACCGCGAAAGCCCCCGGAGCGATGCGGCCGCAAAAGCACCGCCAGCGAYGTCGGAACACCAAGACGTGCGGAAGGAGAC

ACAAACACCAGGCATCGAACCGGAAGCGACGCCGCGAGGGGACGGCGATCCCGGGCACGGATACGAATCCGAGCACGAAT

GCCAACAAGGCGAAAACGGCGAWACCCCCCAGCACAAGCGAAGGTTTGCCTTTGTCCACGACATTTCAAAAGCCAAGATG

CAGCACGAATCCGCCGGCCGAGAAAACGGCGGGAGGCGGTTGCGCCGCCGCCGATCCACTGACACGGAGGCAACCAGGGC

GACGAAGACGATCGTGCCACCACCAAACGAGCCAGGGGGGCAAAATCTCGGCCCCCCAAAGGAACAATTCACCGAGCCGA

GCCCGGTCGCAGCCGCAGCCGCATCCCATCCACCGACCCCCGAACCTCAAGGCGAGAGCGAAAGTGAGCGGGAATCACCC

ACAACTGGAGAGGATGGTGGTGATTGTTCCTCCACGGAGAACGAAACGGGCCAYCTTCCTACGTCAGCCCCCCTGGATCA

AATTTCCGATTCCGACCGCCGGAAATGGATGGAAGTCCAATCCAGGTTTGCGAGGGGCGATCGCGACGAAGATCCTCCGA

GTGGACGAGAAATTGAAGACGAAACCCATCAAAGCGACGACCAGCATATCCTTTCGAGATCGGTTTCACTAGGGGGCTAT

GCCCGGAATACGACGGTGGAAACAAACCCACCGGTCACCCCCGAAGAAGTCATTGATGCTTCTGCTTCAACTRCAAATAC

TGCTACGGCTAGCAAGACAAGCGGGGGCGATAGTTCTCCTCCGAGAGYGCCTTCCTCTYCCACAAGATCGCGGCTCGATC

TCAACAGTCACCTGAGGAAATTCGCCCACAACGCATMTACCGATGGAGCCGGCAACAACAATAGCAAGCAAAACCAGAGA

GGTGGATTTCTGAGTCAATTTGCGTGCCAGGCATCGGCGGACAGTGGAGTCGYAGACRTAAACAGCRGCAGCAGYAGTAG

CAGCAATAGAAACAACCTTGCYCACAAACGCCGCCGTAAACCCGCTGTAAGCCCACCGGATATCAASGAAGCGCCACCTT

TGAAAAGCTCCGCTAGCTCTAGGGATGGAGCAAGTAAAAAGTACGCGGCCGCCCATGTTAACAACTCGTTTGCTGGTGAT

GGAAAAGAATCACCCCGCGTATCATCCARGAAACGAAAAAAAAGGGACGAGGACGAATCGATGGAAATTGAAACCGAAAG

RACGAKTCCCTCTTCCATTGCACCAGAGGAGTSCAACCCAGATGAGGGAGATAGTACCACCGATGCGGGCACCGAGCCCC

MGGTAGTATGGGAAGCCTTTTCTTCAACAGAAAGGATCTGCTCGGATTCCAGAGCGGAAAGGCTTCTGATGCGCAAACGA

GTGCAGGACGTCCATGGCAATMGACGATTGCTCAAAGAGTCAATCTCAGACAGGAACAGTAAATCTGCTCGACAAGCAGA

YATCAATACCARACSTAATGAATCCTCCCAMGGGGCCAACGCAAGCGAGGAAAAAGACTATGGTAGGGATRACRACGACG

AGGATTCCTCTTCGAGCAGTCCCTTCATTCGAATCTCCAAGTCCACGTTTCGCAGCGGCATGCAGGTGATTGGTCAGTTC

AATCTCGGTTTCATAYTAGCGAGATGCCCGCAGAACCACCTCTGGATCATGGACCAGCACGCTTGCGACGAAAAATACAA

CTTCGAGCAGCTCTGTAAGAAGACGGTCATGCACGTCCAGCCMCTSATACGGCCCCTGCCCCTCGAGCTGAATCCGTCCG

AGGAAGCCTGCGTTCTGGACCACATGGACGTCTTTGCCTCGAAYGGATTCCGCTTCCGATTCGACGAAACCGCTCCCATC

CGCCACCGGCTGTCGCTGACCTCCCTGCCCCACTCGGGCGCCCACGAGGGCCGCAAGGCCGTCGCCTTTGGACCCTCGGA

CGTATCGGCCCTCTGTGCGATCCTCACGGAGGGTTCTTCCTACGAGGCCGGTTCCGGCGGGACGGGGACCGATGGCACCG

GGCTCTACGGGAACAACGCGGTGCGGCGGCACGCCGGCGGAAGTCAGAGCGAGACCCCCGACCGCCTGCTGGCCCGCCTG

CCCAAGGCCATCGCGATGTTTGCCAGCCGGGCCTGCCGGACCAGCATCATGATCGGGACGGCCCTTTCCCGCAAGGAAAT

GGAGGCCGTCGTGCAGAAGCTGTCCGAKACCGATTCCCCCTGGAACTGCCCCCACGGGCGGCCCACCATGCGGCACGTTG

CCAACGTGCTGCCGTTCCTGGAGGAGGACGAGCGCCGCGCAGAAACCTACGYTTCGCTTCCCGCGATCTCCGTCGTTCCR

GCCGAGGAAGCAGAGCGGGAGTCCTGAGGGAATCGGATTCCACAGAGGAATGGATGGGAAGTCAAGGCAACAGCAGAGTT

TGAAAACCCACATCTAATCTTGCCACCTT

>PSNMU-V1.4_AUG-EV-PASAV3_0100930.1 class=Sequence position=[PsnmuV1.4_scaffold_58-size_221033:91116..100613 (+ strand)](http://gbrowse255.tgac.ac.uk/cgi-bin/gb2/gbrowse/maplesod_psnmu_v1_4_gbrowse255?name=PsnmuV1.4_scaffold_58-size_221033:91116..100613) ***MUS81* Might be wrong gene model contains extra SEC5 domain not part of *MUS81***

ACCGGTGCATTGCGCAACATCCACCACCACAACCGCATCGCGAGATGCCCTACAACCACTATTCCAAGAACACCACGGTG

ACGGTGAAAAAGGCCAGGGCCAGGCACCCTTCCAACCAGCCCGGCCTCGATCGGCTGCAGGAACTCCTGGTCAAGGCCAC

CGCGAACAGCGCCTCGGGAGCGGGAGGAGGAGGACAGAACTACGCGACCACCATCCAGCGGGCCCTCCGGTCCCTCAAGG

ACTGCAGGCACCCGATCGTTACCCAGCAGGACGCCGTCAAGCTCAAGTACGTCGGCCCGGCCATGGCCAAGAAGATCTGT

CCCCCCGAAGCAAGCTCGGGCCCGGGCCCGCTGCCCMAAAAGCGCAAGAGGGATGCCCCTACCGAACCACGGCGYTGGGA

ASCATGCGTGGACCGGGGGGTTCTCCCGGGCCGGCCGGACGCACCACCGCCTGTCGCCCTCTGCCCGGTGCCGGCCCTCC

GGTCGGCTCGTTCCGCGCCGTGTGGACCGAAGAACAACACCCAACGACGGGACGAACGCCACCCAACCGCCAAGGAAGCC

GCCTACGGGGCCGCCAGGAAGCACGCCGAGGCCCTGGCGGTCGCCCCAAAGGCCCTGGAGGGGACCTGGAAGGTCGTCCT

GCTGGTGGACAACCGGGAACACAGGTCCAAGCAGGTCGTGAGCTCCTGCAAGCAGGCGGGGATTCCCTGCGAGGAGCGGC

AGCTGGCAATTGGGGACATGGCCTGGATCGCCCGGAAGATCCCTTCCAGCAAGAAAAAGGACCGCGCAATCGGCAAAGGC

GAAACCGAAACCGGCGCGGGCGAAACCSCCCTCGAGGTCCTGGTCGGGACGATCGTCGAGCGCAAGGAGGTCGGCGACCT

GGCCTCGAGCCTCTTCGGGACCCGGTACGCCGAGCAGCGCCTCCGGCTGAGCCAGTGCGGGCTGCCCCAGGTCCTCTTTC

TGGTGGAGGGCGACCTCCACGCCCTCAGCAACTGCCCGGCCGAGACGCTGGAGATGGCCATGATGGAAACCCGCCTCCAG

CTGGGCTTCTCGGTGGTCCGGACCAGGAACCTGCCCGAGACGGTGGGAATCCTCAAGACCCTCCACGCCCGGATCGTCCA

GCGGACCTTTCCGTCCGCCTTTGGGAGGGGGGCTCCSAAGGGCAACCSCACGGGCAGCACCAGCGGCACKATCGCGAMRC

CCCAAAAGCAGGCCCTCCCGAGCTTTGGGGGGGGGAACCGGAGGAGACCCGGGAGCCACCGGAGGCCGGCCTCCCTCCTG

GAGCTGGTCTTTGACACGCCGCCCGTCCCGGCCTTTGGGGGGAAGCGCTTTGTCACCTACCCAGAGCTCCGGGCCAAGGT

GGAGGTCGACCGCGAGCGGGGAACCAAGAGCGTCCGGGCTTTGACCCTGGCGATGCTCAAGCAGATCCCGACCCTCTCCC

AGAAGAAATGCACGGCCCTTGCCRGTCGGTACCCCACCCTCCGGAGCCTGATGGAGGCCCTCACGCATCCGGGCCGCGAC

AACGACCACCAGAGCGGAAACGACCACAGCGAAAGCAACGGCCACAACGGCAACAACGACGGCGACAACCAACCCCCGCA

GCGAAGACCGCGACCCCGACTCTTGCACCACCCCAAGCAGATCGTTGGGGGGATCGAGATCGAGGGCGGCCACGGCCGCA

CCATCGGCCCGAGCTCCGCGGCGGAGGTGTACGCGGCCTGCTGCACCCTGCCGGATGGTTCCACGGTGGATTGCCACCGG

CCCCACGAGCCAAAGGTGCAAGGAGTGGCGAGAGGCAGAGGCAGAGGCAGAAGCAGAACCACGGCCACCACCACAAACGC

AACGACGACGACGACGACGACGACAGCAATGGCAGCAACGACCGCAACGAGAACCGTGGTTTCGTCTTCGGTCCTTGCTG

CTTCCACGGCCAGGGGCTCGCAAGCACCACCAACGGGRCCACAAGCACTCCTGCCTGCTAGAGGGACCGTGCAGAGCGAC

TTGGAGGAATGCGYTGCGGTGGTTGGAAAGCAGCCGAGCAGAAGCAAGGYGTCGATCCCGGAAGCCGTAGCAGCAGTGGC

AAGGAAGGATTCGCTTCTMTTTTCCTCGTCCCCTATTGGCGGCGACGACGACGAGGATCTCCCTTTTCGGAGCAACCGCA

TCCCTGCAAACCGCAAAGCTACTTYTGTTGCTGCTCGTCATGCCGATGCTACTCGCCACACCAGCACACAACAGCAAGAA

WYCGTCGATCTCTTGACACCCGAGGCAYCCAGGCACGAACCAAAACGTGCGGCCRATATCGGCCGACTATCGAGCAGCTC

CGGTTGGTCCAGCGACAGCAGCAACGATACTCCCGCTGCCACGGCCGTCACCAAATTTTCTGCAAATGCCAACGACCAAA

ACAACAAGAAGACTTCTGCCACAAGGGAGACGGCACAATCCAATTCAGAGAAATCCGCAACTTCCATCACAAACGCGGTG

CGAAAACCACCAACGGCCCAATCGATTCTTCTCTTGTCGTCCTCTTCGGAAGACGAAACGGACAACGACAACGACAACGA

CAACGACAACGCTTTTTCCCTGGGTGGCGAAACCCGAAAGAAGCCACCACGCCTCCAGGACATGGACGGCAATGCCCAAA

AACGCAGCAACCATTCGGATATTTCGAACGGAGAGAAGACGACGGCTGCGATTCCCTCCAGAAACGGGCCTTCGTCCGGG

GTTGGTCGCCCGGTGGCAGAAAGGGGGCTTCCGACCACCCCTCCCGTCTTTTCCGGTCGCCAAAGGGAAGCCCCAGCGGC

GGCGGCCGAATCGTCGGCCTCCCCTTCCTCGGACGGCTCCTCCGTCCTCTGGTCCCCCGAGGACGGGGGGTTCACCCCGG

TGTCGCWGCGGGGAGGCACCGGGGCCGGTGTCTACCGCCGCCGGCCGGATCCCGCCGTGGCAGGAGTCGTGAGCCTCCTG

GACGACGACGGCGACGACGATAGCGATACCAACCATGGCGGAAACGAGCAACCCAAACCAATGACCGCAGCCGGCAGCCA

CAAGACCAGCCACAGCCAGAACACCAGGGGCACAGACAAGGACCGAGAAGACACCAACGCAACGAACCGTCGTCGGTGGT

CGTCCCCGCGGAAGGAAAGTCTCTCCAAAGACAACGACGACAACGACGACAACGACGACGCGTTCCTTTCGAAGCCTGCA

GTGGCTGGCGCCGCCAATGCTTCAGAGCAAGGTGCCTCCCTGCTGCTGCTNNNNNNNNNNNNNNNNNNNNNNNNNNNNNN

NNNNNNNNNNNNNNNNNNNNNNNNNNNNNNNNNNNNNNNNNNNNNNNNNNNNNNNNNNNNNNNNNNNNNNNNNNNNNNNN

NNNNNNNNNNNNNNNNNNNNNNNNNNNNNNNNNNNNNNNNNNNNNNNNNNNNNNNNNNNNNNNNNNNNNNNNNNNNNNNN

NNNNNNNNNNNNNNNNNNNNNNNNNNNNNNNNNNNNNNNNNNNNNNNNNNNNNNNNNNNNNNNNNNNNNNNNNNNNNNNN

NNNNNNNNNNNNNNNNNNNNNNNNNNNNNNNNNNNNNNNNNNNNNNNNNNNNNNNNNNNNNNNNNNNNNNNNNNNNNNNN

NNNNNNNNNNNNNNNNNNNNNNNNNNNNNNNNNNNNNNNNNNNNNNNNNNNNNNNNNNNNNNNNNNNNNNNNNNNNNNNN

NNNNNNNNNNNNNNNNNNNNNNNNNNNNNNNNNNNNNNNNNNNNNNNNNNNNNNNNNNNNNNNNNNNNNNNNNNNNNNNN

NNNNNNNNNNNNNNNNNNNNNNNNNNNNNNNNNNNNNNNNNNNNNNNNNNNNNNNNNNNNNNNNNNNNNNNNNNNNNNNN

NNNNNNNNNNNNNNNNNNNNNNNNNNNNNNNNNNNNNNNNNNNNNNNNNNNNNNNNNNNNNNNNNNNNNNNNNNNNNNNN

NNNNNNNNNNNNNNNNNNNNNNNNNNNNNNNNNNNNNNNNNNNNNNNNNNNNNNNNNNNNNNNNNNNNNNNNNNNNNNNN

NNNNNNNNNNNNNNNNNNNNNNNNNNNNNNNNNNNNNNNNNNNNNNNNNNNNNNNNNNNNNNNNNNNNNNNNNNNNNNNN

NNNNNNNNNNNNNNNNNNNNNNNNNNNNNNNNNNNNNNNNNNNNNNNNNNNNNNNNNNNNNNNNNNNNNNNNNNNNNNNN

NNNNNNNNNNNNNNNNNNNNNNNNNNNNNNNNNNNNNNNNNNNNNNNNNNNNNNNNNNNNNNNNNNNNNNNNNNNNNNNN

NNNNNNNNNNNNNNNNNNNNNNNNNNNNNNNNNNNNNNNNNNNNNNNNNNNNNNNNNNNNNNNNNNNNNNNNNNNNNNNN

NNNNNNNNNNNNNNNNNNNNNNNNNNNNNNNNNNNNNNNNNNNNNNNNNNNNNNNNNNNNNNNNNNNNNNNNNNNNNNNN

NNNNNNNNNNNNNNNNNNNNNNNNNNNNNNNNNNNNNNNNNNNNNNNNNNNNNNNNNNNNNNNNNNNNNNNNNNNNNNNN

NNNNNNNNNNNNNNNNNNNNNNNNNNNNNNNNNNNNNNNNNNNNNNNNNNNNNNNNNNNNNNNNNNNNNNNNNNNNNNNN

NNNNNNNNNNNNNNNTGGATATTGGATATTGGATATTGGATATTGGATTGGTTCGTATCCGGAGTGCCTGCTGCCAACGT

TTTCGGGTGCTTTTGGCTTTGTGCCACTGTATAACAATTGTTGCCTCTGTTGCCTTTGTTGCCTTTGTGGAGCTGTACSG

TAKGATGCTACAGTWTTAGTASTAGTACGTACGGTGCGATGCCGTATGGACTTCGTACTTGGTAGTTAACGAACGTGAAC

GACGGGTAGGAAAGAAAAGAGATCAGTGACCCCCCACAAAACCAAARCCGACCGTTTCGCGAGGCGAGGAGCCAAAACAA

TGATAAAAATGAAAACCGCAAAATGCCTTTCTTGTTAKTATTACTATTACYATGTCCCGAATCACTCGCGACTCTTTTTC

ATTCAAAATATAATATATTCCAGTGTTTTTGCATAWTTTTCGAAGAAATGTTGCAWAATCGCGCGATCATTGCCGATTTC

CGAAGCATTTTTTTATTTTTCACTCCTCTACYGGTACTGTAATTCGATCTTTCCAAAGACTTCAAAAACCCTAACGGATA

ACGGCAGTACCCKGTACGAGTACGAGTAGTACGGTACTCGTACTCGTASTCGTAGTCGGCTCGTTTCGATCATTTGCTCC

CTCCCCCTGACAAATCAAGCAACAAATGCTGTGCACGGCAAACGTTGAGGTTGTTGCCCACAAAGTCTAACTAGATAGCA

TAGCATAGCATCGCATCGCATATATCTTTCGTATAGAAACCAAAGAACAACCCAGAGGAGGGAAGACAGACACCAAACCG

AAGCGAAGCGACACCGGCAACCACACACACAAATACAAAAACACACAAACACAAATACAAACACACAAAAAAAAACACAA

ACACACAAAAACACACCAAACCAGCCATGGCCGAGGAGGACGCCGCCGAGCGGGCCACCCAGGAAATCCGGGCCCTCCTG

AGGCGACTCGATCCCAAGGACTCCTCCCACAAGGACCGCATCCGCCGCCTGAACAAGTTCCGAAACTTTGTGACGGGGGA

ATCCAACGCGGGGACCCCCGAGTTCTACGACGACGACATTCCCCTGCTCCTCCTGGGGTCCTCGGCGCCGCCGGCCCTCC

TGGCGGCCTCGGACCTCCTGGAGGAGATGGACTACGGCGGAGGAGGCAGCGGGAACGAGATCTACGGGCTCCTCCAGGCC

TGCGGGACGCCCAGCGTGGAGCACGACCACATGCTGAAGCGKTCCGCCCGGCACGCCATGTCCCTCCTCAAGTTCCTGGT

CATCGATTTCGTGGAGGAGGGAGGGAGCGGAACGGCGCCGGACCTCAACCTCTTTGCGCAGGCCTTTTGCTCCTTCCCGG

TCGACAAGTACGGCTACATGAGCCTGGACCTGCACCTGATCGGGGACCAGAGGGGGGGCGCCCAGGAGGACGCCTGCGAG

GTCATGGTCCTCCTGCTCACCAAGCACCTGCAGGAGGACGGGGAGACCCCCGGCCCCCTCTCGAAGCAGGACCTCCTGGC

CTCCCAGCAGGCCCAGAAGGCCTTCGACCTGTGGGTCTCCCAGCACGCCACCAAGTCCCAGCAGGACCTGATCCGGTCCA

ACGCACAGAAACGGAAGGACTTTCTCGAGGCCGAGGCGGTCCGCCTCTCGGGGGGCCTCACGGCGGAGGAGAACGCCGAG

CTGGAGCACCTCGCCAACGACGACTCGGACGACGACTACGACTCGGACGACCCGAACGACCCGCTGGCGGGGATGAAGCG

CCGCCAGAAGGCCCACAAGCGGGAGCTCCTGGAGGCCAAGCGGACGGGGGAGTACGACGAGGAGCGGGACGGTCCGGCCC

TCCGCTGGGAGGACTCCCTCCTCTACCGGGAGCAGCARTCCCGGCTGGCGGCCCAGGAGGCGGTCGGGAAGGGCGGAAGC

GGCAGCGCCGGAACGACCCTCAACGTCCGCAAGTCCCAGGAGGCGGCCGCCGAGCTGGCGGAGCGGGAGGAAGAAAAGTC

ACGCTTTCTGCGCCGGGACCCCCTGGGGCTRCACGGGGAGGACTTTGACCTGCAGGCGATCGAGACCAACCAGGTCGAGC

AGCTCGAGAGCGCCCTCGGACAGCTCCAGGAAGAGCTCTCCAAGGCCGAGGCGGGCGGGGAGGACGACCAGGCCCTCCGG

GCCAAGAAGGAGTCCCTCGAGATGGTCCTGGACGGCGTGGTGGGGATCGGCAAGGGGCAGAGCAAAGGCCTCGGGGCGGC

GGCCCTCGACCAGGGCGCGACCGGGAAGAGCGTCCTGCCCACCGACGGGAACTTTGATCCCATCCTCTTTCTGACCCTGG

TGCACCGGGGGGCGTCCTACGAGGAACTGGTCGGTTCCATGAACCGGCTGAGCAGTGAGTACAGCGTCGTGCCTGCAGCG

TCGTGTCGTGTCGTGCAGCGTCGTGCATGCTTTGTGTGTGCTTTGTGTGTGTGCTTTGTTTGTTTGTCCGTCTAGTTGTC

TCACCCCGAGTTCGACCATGCGACCTGGTTTTGCCCAACACCAACCATCCGGTTTGCCTTGTTTTCTTCTCGTAGCCGAC

ACGCAAAACCAGGTGAAGCAGCTGCAGGACATGGTGCGGGAAAACTTTGCGCTCTTCCTCCGGTGCGCCGATGGCATCGA

CACGTTCAACGAAAAAACCCTGTCCCAGTCGGGACCGGGGGTCACGGATCGCCTCAACCGGCTGGACGCACTGGCGGAAT

CGTGCTCCCACCAGGCCAAGAAATCCTTCAAGCCGCTGCTRGACAACGCCAACGAGGTCCGAAAGGTGCAGTCGGCCCTC

AACGTCCTGCAGCGCGTCGAGGCCGTCCTGCAGGCACCCTACCTGATGCGCCAGCACGTGGAGAACGGCCGGTTCTCGGC

CGCCCTCAAGGCCTACCGGCTCGTCCAGGTCATCGACGATTCCAACAAGATCGAGATCCTGATCCACGTCAAGCAGCAGG

CGATGGAGTGTGCCCGGGAGGCCCGGCGGGAACTCGAGGGCCGGCTGGCCCAGGACAGCAAGATCGGTGTCACCGGTCTG

CTGGATGCCATTCGAGACCTGGGAGAACTCCTCGAGCTCAACATCCCGGAGGACCCCGGGGAGGCCGAAAAGGTTTCGGG

AACGGCGCGCTTCCGCCAGCAGCTCGTGGGAACCTACAACATCGGGGGGATCACGATCAACGTCCGCGACTTCCCGCCCG

CACTGGCGTGCCTGCTGCTGCAGGCGGCCCACTTTACCTCTTTGGTATCGGGTGCCATCCAGGACGCGGACAGCATCACC

ACGCGCATCTTCCAGGGCGAATCGCTGTCGGCACAGAACACGCTGGATCCGGACARCAACAACAACAACAAGGGCGAAGA

GAAAGAACACGAGGGAAGCAAGACGCCGTCGCGATCCAAGCAATCCTCTTCCGCCAACAGCAACCAGTGGAAATACGACG

TTCTCGAGGCGCGCAGCCTCGTGACGATCCGCGCCGTGGAGATCATCTCCAAGTGGCTCCCGCGTCTCCTGGAGGTGGCC

ATTGCCGCGAGGGAGGACGAAAAACGACGGGCCGCCCGGGTGCGGTCCAACCCCGGCGGCACCAAGGAAACCTACCAGCT

GACGCCCTTTGAGGTCTTTCTGACCAACGTCGCTCCAGTCGTTACCAAACTGGTGGAGCACGCCGCCTTTTGCGGCCTCG

GAAGCGCCCCCCGCGGAAGCGGCATGGACATCAAGATGACCTTTGGAAAGAAATCTTCCGAGAAGCTCCGGGCGCTCCTG

CGCTCGCCCCTCCCGCCGTCCCAGAGCGCCCGCGTCGGGAAGGAACTCGCCGACATGGTCGCGGTCCTGGCCCAGAGCAG

CATCACGGTCAACCAGCTGAGGCCGCTGCCGGGAGAGTCTACGTCTACGGCGAAGAACGTGTACACGCTGTCGCCCCTGG

ACGAAAGCCGAACGCTGGGGGAGCAATCCGTCATGACCATCGAGCGGAGGAGGTGCATCTATGCCTTTGACATTTGTGCC

CGGGGATGTTCCAGCCGGGCCACCGGAAGCGGGAAATTCGATGCCGACTCCCTGCTGWCCTGCCTCCGCACGCTGTCCGA

GGAACTGACCCGGCCCGAGGAATGCTCGACCGAGGTCGAAAAGGGCTGCGAGATCGTYATTCGCAAGTGCTGCGACGGGC

TGGCGTCGTACGTGCGAGACCGGGGAGACACGGCCCGGCTGAGCGCCGTGGCCGAGTGCGCCGACGTCCTGCAGACCCGC

ATGACCGACGTCGTACGGGAGATTGGCTACCTCACGAACAACCACGAAGCGGTCGAGGGGGTCATGATGGAAGATATTAT

GGGTCTCGAGGGCGCCATGTTCGACGAGTTCCTGGACAGCATTCGGGATTCCACGTCGTCGTGCTGCCGGATGGGCTGGC

TCGACGTCCGAACGTCGGATCTGACAAAGGATTCTCCCAGCGACAGTCCCTCGGGAGGCTTTCCGCCCTACCTCTCGGCA

TCGCTGCTRTCGATTGTTCGGTGCAGGGCCCAGGTGGAACAAGCCCTGGGGTCAAAGGTTCGTCGGTCCGAGGGCCAGAC

CTACCAGCACATCGCCATGGCCATCGTGGCCGAGGGGATTGCCGAGGGGATTTGCGAACAAATCCAGCGGCGCAAAATGA

CCCTCAAGGTCCGCCAGTCCGATCGGCTGGCRAACGAGCTGCAGTTTCTGATGAACACGCTCAAGACGTACCTTTCCAGC

GAGGCCATGTCCCTGCTCGACGGCACCCGGAGGATGCTCTGCAGCAAGGCGGGACGCGGAAACGGAATGCAGGGCGAYGG

ACCGGACGGCCTGGCCGCCCTAGAGGAACTGGAACGCCTRGGTCGCGTGTACGTGCTCTGTCTGGGAGTGTAAATACCAA

CGCTGTTGTTTCTAGTCATAAATTGGGCTGCATGCCATAATAGAATACGATTCAAGAA

>PsnmuV1.4_aug-pasa-abinitio_v2_0044970.1 class=Sequence position=[PsnmuV1.4_scaffold_58-size_221033:91116..93500 (+ strand)](http://gbrowse255.tgac.ac.uk/cgi-bin/gb2/gbrowse/maplesod_psnmu_v1_4_gbrowse255?name=PsnmuV1.4_scaffold_58-size_221033:91116..93500) *MUS81* Corrected model V2 AUGUSTUS

ACCGGTGCATTGCGCAACATCCACCACCACAACCGCATCGCGAGATGCCCTACAACCACTATTCCAAGAACACCACGGTG

ACGGTGAAAAAGGCCAGGGCCAGGCACCCTTCCAACCAGCCCGGCCTCGATCGGCTGCAGGAACTCCTGGTCAAGGCCAC

CGCGAACAGCGCCTCGGGAGCGGGAGGAGGAGGACAGAACTACGCGACCACCATCCAGCGGGCCCTCCGGTCCCTCAAGG

ACTGCAGGCACCCGATCGTTACCCAGCAGGACGCCGTCAAGCTCAAGTACGTCGGCCCGGCCATGGCCAAGAAGATCTGT

CCCCCCGAAGCAAGCTCGGGCCCGGGCCCGCTGCCCMAAAAGCGCAAGAGGGATGCCCCTACCGAACCACGGCGYTGGGA

ASCATGCGTGGACCGGGGGGTTCTCCCGGGCCGGCCGGACGCACCACCGCCTGTCGCCCTCTGCCCGGTGCCGGCCCTCC

GGTCGGCTCGTTCCGCGCCGTGTGGACCGAAGAACAACACCCAACGACGGGACGAACGCCACCCAACCGCCAAGGAAGCC

GCCTACGGGGCCGCCAGGAAGCACGCCGAGGCCCTGGCGGTCGCCCCAAAGGCCCTGGAGGGGACCTGGAAGGTCGTCCT

GCTGGTGGACAACCGGGAACACAGGTCCAAGCAGGTCGTGAGCTCCTGCAAGCAGGCGGGGATTCCCTGCGAGGAGCGGC

AGCTGGCAATTGGGGACATGGCCTGGATCGCCCGGAAGATCCCTTCCAGCAAGAAAAAGGACCGCGCAATCGGCAAAGGC

GAAACCGAAACCGGCGCGGGCGAAACCSCCCTCGAGGTCCTGGTCGGGACGATCGTCGAGCGCAAGGAGGTCGGCGACCT

GGCCTCGAGCCTCTTCGGGACCCGGTACGCCGAGCAGCGCCTCCGGCTGAGCCAGTGCGGGCTGCCCCAGGTCCTCTTTC

TGGTGGAGGGCGACCTCCACGCCCTCAGCAACTGCCCGGCCGAGACGCTGGAGATGGCCATGATGGAAACCCGCCTCCAG

CTGGGCTTCTCGGTGGTCCGGACCAGGAACCTGCCCGAGACGGTGGGAATCCTCAAGACCCTCCACGCCCGGATCGTCCA

GCGGACCTTTCCGTCCGCCTTTGGGAGGGGGGCTCCSAAGGGCAACCSCACGGGCAGCACCAGCGGCACKATCGCGAMRC

CCCAAAAGCAGGCCCTCCCGAGCTTTGGGGGGGGGAACCGGAGGAGACCCGGGAGCCACCGGAGGCCGGCCTCCCTCCTG

GAGCTGGTCTTTGACACGCCGCCCGTCCCGGCCTTTGGGGGGAAGCGCTTTGTCACCTACCCAGAGCTCCGGGCCAAGGT

GGAGGTCGACCGCGAGCGGGGAACCAAGAGCGTCCGGGCTTTGACCCTGGCGATGCTCAAGCAGATCCCGACCCTCTCCC

AGAAGAAATGCACGGCCCTTGCCRGTCGGTACCCCACCCTCCGGAGCCTGATGGAGGCCCTCACGCATCCGGGCCGCGAC

AACGACCACCAGAGCGGAAACGACCACAGCGAAAGCAACGGCCACAACGGCAACAACGACGGCGACAACCAACCCCCGCA

GCGAAGACCGCGACCCCGACTCTTGCACCACCCCAAGCAGATCGTTGGGGGGATCGAGATCGAGGGCGGCCACGGCCGCA

CCATCGGCCCGAGCTCCGCGGCGGAGGTGTACGCGGCCTGCTGCACCCTGCCGGATGGTTCCACGGTGGATTGCCACCGG

CCCCACGAGCCAAAGGTGCAAGGAGTGGCGAGAGGCAGAGGCAGAGGCAGAAGCAGAACCACGGCCACCACCACAAACGC

AACGACGACGACGACGACGACGACAGCAATGGCAGCAACGACCGCAACGAGAACCGTGGTTTCGTCTTCGGTCCTTGCTG

CTTCCACGGCCAGGGGCTCGCAAGCACCACCAACGGGRCCACAAGCACTCCTGCCTGCTAGAGGGACCGTGCAGAGCGAC

TTGGAGGAATGCGYTGCGGTGGTTGGAAAGCAGCCGAGCAGAAGCAAGGYGTCGATCCCGGAAGCCGTAGCAGCAGTGGC

AAGGAAGGATTCGCTTCTMTTTTCCTCGTCCCCTATTGGCGGCGACGACGACGAGGATCTCCCTTTTCGGAGCAACCGCA

TCCCTGCAAACCGCAAAGCTACTTYTGTTGCTGCTCGTCATGCCGATGCTACTCGCCACACCAGCACACAACAGCAAGAA

WYCGTCGATCTCTTGACACCCGAGGCAYCCAGGCACGAACCAAAACGTGCGGCCRATATCGGCCGACTATCGAGCAGCTC

CGGTTGGTCCAGCGACAGCAGCAACGATACTCCCGCTGCCACGGCCGTCACCAAATTTTCTGCAA

>PSNMU-V1.4_AUG-EV-PASAV3_0010100.1 class=Sequence position=[PsnmuV1.4_scaffold_119-size_146336:68086..77730 (+ strand)](http://gbrowse255.tgac.ac.uk/cgi-bin/gb2/gbrowse/maplesod_psnmu_v1_4_gbrowse255?name=PsnmuV1.4_scaffold_119-size_146336:68086..77730) *FANCM*

TACGAATTAAAGATTTTCTTGCGATCGTTCCTGCTCGGAGTTTACTCCATAGAATTGTGAGAGATTTTTTGGCGCGGTTT

TCCACAGCAAGTCTAGGAGTACAGTACGGTACGTTCCGTATCGTAGTCTATCCGACTATCCGACCATGCATGGCATCCGC

ACGACCAGCTAGATTGAAAACATGCACGACAGGTACGGTGCTTGTACGAGTACTACCGTCACTACGGTACGTACGAGTAC

TACGATATTCGGACTCGTACTGGTACGTACCATCACTATTCTCTACAAGCAACCAAACATGGAGCATAGGATCGGTGTCC

TCCCAAACGGACTCGGATTGGATCCCGAACCACCACGAACGGCACGCCGCACAACCCAATAGCACACACAGACAGACACC

GCGGAAGACCCGTCCCAACCCCACTCCCTGCCGCCATGGCGACGCGGTCCGCCTTTGGGTGGAGCTGCCGGAACTGCACC

TTTTTCCACAGCGTCCCGGCCCCGAGGTGCAGCATGTGCGGGGAGCTCCGGGGGGCCTCCCGCAGGGAGATGCACGACTT

TGTCCTGGGGAAGAGGGTGGCACCGGTGGCGGAGGGAAACGTGGTGGAACTCCCGGAGAACGACGAGGGGGAACGAGGCG

CGGGTGGCACCAACCGCAACGGCAACGGCAACACCGACGGAGGCGGGGCGTCGTTCTCGAAAGAGAACCGGCACAACCAC

CGGGCACCCACCCCGCTCCCCCGGACCACCGGAATCAGTGCTGCGGCTGCGGCTGCGGCTTCCAGGAATCCGTATTCGAA

ACACCCAAAGCCCGTCCGTGCGGCAGCAGCTGCAGCAGCAACAACCACAGCCACAGCCACAACAACGGCAGCGAGAACAA

AGAATGCCACCAACCACCCCTTTGTCACCACTCGGAAACCTCGCAACACAAAAAATGCATCCGTTCGAAACCCGTACTCG

ACGGGTCGATCGATGACGAATAAGGGGCATCAGCAGAAACAACAACAGCAGCAGCAACAGCAGCAGCAGATGTCCGACGC

GGCCATGGATTCCACAGATTCCAACGCGCAGGCACCCCAGCGGCTGCCGAATCCGCAGCCAGAAAAGCCCCCGCCTCCCC

GGCCGGGACCGCAAAAGCAAACCCATGTCCGGCCGCACTCCTTTTTCGACAAGAACCCGTCCTCCCATGCCTCCAAGCGG

AGAAAGGGCCAGCAGCAGCAGCAACCAAAGGTCCACCACTTTGGCTTTTCGGACGGCCCGGTCCCCCACAACCCCGACGC

GGCCCTCACCTACATCTACCCCAAGCACCCCGACTTTCCGACCCGGGACTACCAGCTCGAGATCACCGAAACGGCCCTCC

GGTACAACACACTCGTGTCGCTCCCGACGGGCCTCGGAAAGACGCACATCGCCGCCGTGGTCATGTACAACTTTTGGCGG

TGGTTCGCACCGCCCGGGGCCTCCCTGCAAGGCCGCAACCACGGCGGTGCGTCGTGCCGCGGGGGCAAGGTCGTCTTTCT

GGCGCCGACGCTCCCGCTCGTCAACCAGCAGATCGAGGCCTGCTACAACATCATGGGCATCCCGGGGCACGAGACGGCCG

TCCTGACGGGCCGCCTCAAGGCACACGAGCGGAGGGAGCTCTGGAGGACCAAGCGCGTCTTTTACTGCACGCCGCAGACG

GTCCAGAAGGACCTGGTGTCGGCCTGCGGGGGCGGCAGCGGAGAGAAGAACGAAAGAGGCAACGGCCAGAACCACGATCC

GAACGGAGGCACCGTTGGGGTCGACCACGAGACGGCCCTGGCCTTTTCGAGGGTCGTCTGCCTGGTCCTGGACGAGGCGC

ACAAGGCCACCGGCGACTATGCGTACACAAAGGTGATCGAGCTCCTCGAGAAACACGCCGGGGCCAAGTTCCGGATCCTG

GGCCTCTCGGCGACGCCGGGGACCTCCATCAAGGCAATCCAGGGCGTCATCGAAGCCCTCCGGTCCTGCAAGATCGAGGC

CCGGACCGACGCCGATCCGGGGGTCGCCCCCTACCTGCACGAAAAGAGGACGGAGATCGTGGTGTGCCCCCGGAACACCC

ACCAGAGGGACATCGGGAGGAGGCTCTCCCACATCGTCGAGCCCCTCCTGAAGCGGCTCAAGGACGAAAAGCTCTTCTCC

TACGGAACCCCCGAGACGCTGACGCCCTACGCGGTCTTCCGGGCCAAGCAGCAGTACGAAACGAACTTCCGAGGCCGGAC

CAACGGGGGCATCCTGTCCTGCTTCCACGCGACGCACGCGCTGATTCAGATCAAGAACGACTGCCACCAGGGCCTGGGGG

TCGTACGGACCAAGCTGCTGCGGCTCAAGAGCGGCCCACAGAGGGGCATCCTGAGCACGATCGTCAAGTCGGACGACTTT

GGGGCCCTCTTCGAAAAGGTCATGGAGGCGACCGGTGGCGGGCCGGACGGGAGCAACAGCAACAGCAACAGCAACAACAA

CGCTTTCGCCGTTGGAAGCGTGGTCGACCCCAAGCTCTCCAAGCTCTGTGAGCTGCTGAAGGTCCATTTCGAGCGCAGCA

ACGCCGAGAACCACTCCTCCCGGGCAATCGTCTTTGCGCAGTTCCGGGACTCGGTCCAGGAAATCGTGGGGTGCCTGGAA

CAAACGCTCAAGCCGCTGGTCCGATCGCGGTACTTTGTGGGGCAAAACAAGGGCGGCGGGGGAGGAACCGGCCAATCGAA

GCAGCAGCAGCAGCAGACGACGACGACGACGACGACCGGCAACGACAAGGTCTCGGGCATGAAGCAGGCCGAGCAGCAAA

AGGCCATCCGCGACTTTCGGAACAACGTCTTCAACGTGCTGGTCTGCACCTCCATCGGGGAGGAGGGCCTCGACATCGGG

GACGTCGATCTGATCATCAACTACGACGTGATCCGGTCCCCCATCCGCACCATCCAGCGGGCGGGCCGGACCGGCCGCAA

GCGCGACGGCCGGGTGGTCTCGCTCATCGCGGAGGGCCCCGAGGAGCAAACCCACAAGAAGCGGGTCGAGGGAGAGCGGA

CCCTGGCCAACGCACTCAAGAACCCCAAGAAGTTTGTCATGGCCAGCCACTATCCCATGCTGCCCGGCCACGGCAGCCAC

CACCAGGCCGGCCCCGCTCTGGAATACCAGGCCATGCAGCCCAAGGCCCGGCTGAACATGAAAGAGGTGGCCGGGGCCCA

GCTCACCACCCCCGGTTCCAAGAAAGCGGCGGCGGAGACCAAGAGGAACGCATGGAGGCTGACGCTCGAGCAGGAGGACG

AGCGCAGGCACTTCATGGGGGGCGGCAACGGCTCGGACGCAATCGCGTGCCTCCCGGATGCCGTGGCCTGGAAGAAACTC

AAGCGATTCTTTCTCAGGAACCGAGCCGATCCCTCGAGGCTTCCACCCTCTCTTTTCTACCGCGGGAAAGGCAAGCGGCT

GGTCCCACACGAAAAAGAGCATCTCCTGCAGCGACGAAAGGAACTCCGGAACCGCCTGGTGCAAAAGCGGGCCCATCTGT

TCCGGGGGAGGAACGCCTCGATCCTAGAACAAATCCAGCAAAAGCACGGGCCGGTGTACCTTGTTGGTGCTGCCGCGGCA

AAGAGGGGGCACGAGGCGATCCTTGAGCTGTTTCCGGTGCACCAGGTCCGCGACGTGGAATCCGACACGATCAACAAGGC

CGGAGGGACCGGCCCCGGTTCCGGGATCCGCAAGAACATGTGGCCGAAAACCAAAACGGACCAAGGCGGACCCGCGACAA

CATCGCCCGGTGTTGCCAACAGAAATGCCCGTGCATCGGAATTGCCAAACACTACGGTAGATGAGACAAGACAGGGAAGC

GAAGCTTCCGTCGTGGATGGGAACGATGCAACGGGCGCTCCATTCTCGGGAGCAGATTCGCTTGCGAAAGAGAGTTTAAA

TGGACGTGGCGATGCTCCGCCTGCAATCTCCAGACAAAGACCACCTTTGAACGAGGGTGCGGAACCCGGCAAAGAAAGCC

TATACAGAAGCAGTATTTCGATGTCGGAAAAACATCGTCGTTCGAGTGGCAATGCCAGCGGAAGTACCACCCATGACATT

GAAAACGCTGGGGCACTGACGGGTGGAAAGAAGTTAACCGATTCAAAAAGAAACCAGGTCCCAATGTCGCTGCAACCTCC

AGTACCGCAAAATTCTCGATCGAAAGCTGAAGTCGACGAATATGTTGACCGTGAAAGCGAAAATACCAGTACACTGCAAG

GTGGGAAGAAAGTGGCTGATTCCGAATCCGATCCCGTTCCATTATCACCACAACCGGAGAGGGCTTTGTTTCGGCTCCCT

ACCCCCCCGCCAACCTCAGATTCCAGCAGGAGTGACTTTCCTCGCTTGTCCGAAAATCTCGAAAAGGCTGTCTTTCGGTT

GCCAACGCCTCCGCCCTCATCGGATTCCAGCAGTGGCGAAGACGAGGACGAAGGGCGAAGCCAGACCGTGTGCGAGACAA

ATCATCCAACGACCTCCTCTGGCGTTGCTGTGAATAACGAGTTTGAATTCGAAGAAAACAAGAATGCTTCCGGGGGGATC

CCTCAACAAATTGCCATCCAAGCCGCAGAAGAAAGTGACAACCTGAGGACGACAGCGAGTGACACCAAACCAGTCCAAGA

AGAATCGGACAACGATGAGCGTGGTGGCGACCCGAGGTTTTCCCAATCCAACGCTGCGGGGTTGGACACCAAACCAGAAA

TTAAAGCCATCTTTCGACTGCCGACTCCGCCACCATCATCAAGTTCAAGCAGTGACGAGGACGACGAGAGCAATGACGAC

ACTGAAGTTGAACTTTTGCCACAATCTGGAACCATCGAAGGTCGTTTCTCGCACGCAGCTCCTCTGGTCAAAGCAAGTAC

TTTTCATGAGGAAGACATGAACCGCGGGGAGGAAGAAGTTCAAAACCCAATCGACAATCTGGATCACAATGATCACACTA

TGATTAACCTTGAGGATAAAAAATTCAGAAAATTGAGGCTGCCTGCAAGAAAAGGTCAATTAGGTGGAAGTTCTCCCTAT

TGCGAACAAGAGAAACTTGTTTCCGGCGTCAGCAGTGATAAAGATCTTGCACTGAGTTCGCTGAAACCCAAACCGAGTGT

TGCCAAGGAAGATAAAGCGCTCACCCTTTCAAGTGTCGAAAAAGGAAATCATTTTCTTGGCTTCGAAGGTGATGACGATG

TTGCGTTGAGCACTTTGAAAAAATCTAGCATTACCAAAACGATGAGCAGAAACGATGGGTATCATATGGATGGAACCGCG

TGCGGAAAAGATATCACTTCCGATAGCCCTTTGAAGATATTGGAAGGAATCACGTTGAAAACCTCCCAAAACGACGTTTC

TCTCTCTTCATTACGGAAGAAGAATGAGAGGAAGAACCCGGCTGTTGAAAACAATGTCTCGTCGGGTAAGAATAGATACG

TTGAAGGCGAGAGATTTAGAAACGATTCCGAACAAAATAAGAAAAAAAGGAAGAAAGATGCAGGGGATGATACAGATAGT

GATGAAGATGTACCTTTGACCTACTTTAAAAAGACAAGGGAAAGAAGTCATGACGCGGAGAAGTCAAAAAAGGATTCTCC

AGATATTTTGGATACTCCATGTGACAGGCACGATGGTTCAGACAAAAGCCACAATCAGAATTCTACTGTTGCAAAATCAG

AGAGATGCATTCGTCCTCGACTACTGGAGACACCCGAAGGTTCCGCTACTGACAGTTTGAAGGAAAGTTCCGTACCAACG

CAAAGTCCAAGTCAACAGGAAATTGTTAATCACTTGAAATCAGGCAAGAATCGGAAGCGGCTACGCATAGGAACCGATGA

CAGCGACGAAGAAAACGGCGAGGAGGAACGTTTAGTCACGGGTTTATCGTATCAAACTATGACTCCTTCTTCTTCTTCTG

TGAATAATTCGACTTTACGCACAGTTGAAGATACACCAAAGACGGCCTCTGATCCCATCTCTCGTTTCTTGAGGGATACC

CCGAACAATGCTGATGCGTCGGCCTTTTTGACTGATACTCCTGCTACAAAGAACGGTGACATCGCAACGGAGATTGTTTG

CCAAATTTGTACATCGGGCGATACGACGGATAAAGACCCGATAGTATTGTGTGACGGCTGCAACCTTGGATTTCACAAGC

TCTGCTACCGAGCAGAAGTAGACGTTGAGTCGGCAGATCCTTGGTTTTGTGACGCTTGCAAGCATCCTTCGCGATGTCTC

TCTTCAACTACATGCACCTTTTGCTGCCAGCACAACGGTATACTGAGAAAGGATGGAGCGACATTCTGCCATCCTCTATG

CCTGGCATTGTCGTGCAAAATGACGTCTGCTAATTGTTCTGTTTGCTCCCTTTTCGGTGCGGTACAGTGCAATTTGTGTT

CCGACGCTGCCCATCCGCATTGTGCACTAGACTCCGGTTGGACTATCGTGCATTGTGCAGCAACCAAAGGGCAGCCCATG

AAGCATTCTATGTTTTGCCCGAAGCATAGTGGCAACGCAGACCAGCTATCGTCGGAAAATACTCGAATCATACGCAGCAA

GAAGAAAACAAACATGAATGGTTCACAAAGGCCAAAAAAACTCAAGCGGAAAGGGAAAAATACATACGAGCGAGAACGAA

CGCAGTCCGAGAAGGTCTTTGGAACTAACAATTTTCGGGAACAGGAAATAGAGATACTTGATGATACAGATGACGACGAA

GAAATAAAAAAGAAAGAAAAGGAAAGCCTCAAAGAACGCCGACGTCGTGGTCTCGCTCGCTTTGTCCTAGATGAGGCAGA

GATCGGATCCGATCAAGACCATGACGACGAGATCGAGAATGAAGAGCTCCGCTTACTCGAAGAGGAGGAAGGGACTGGAT

CCCAAGACAGTTTTATCAACGACAATCCGGATCTGACGCAGCATTTTTCCCAAGATGTTCTTGGCGATGTCGATCCCGAC

GCCGCGTCTACTGATTTCGTTTACGGTGCTTGTAAAAACCGCGATGAAAATGACAGCGGCGGCTCCAGCAACATTCACCG

TGCGTTGGATGCTCGTCGGGAACGCGAGCACCAATTCCGAACCCCGAACTTCAATCGTCGCAGCATGAGAGCACCTGATT

CTTCATCCCCTTCTTACACCGAAAATGGTTGTTCAATACCTTCCTCGGAACGCGGACTCGGCCGAATGCATTTCATTCGC

AGCGTCTTAGAACACCACCGGCAGGGTGGGGACTGTGATGAGATCGAAAGCGAATATAAGCGCTTGGAGGCTTCTGCTGG

TGCCGATGTTGATGCCACACTCTCTCAAATTAGATCGGAATCGACGCCAGCCAATGATCGCAATGCTCTGATTGATCTGT

CGCGTTCACAAACCAACGGGACAGATTCTCCAATCCAACTGATGACTGATGGTTTGCGTCCCGCCATCAGCAACGGCGAA

GTGAACCCCATTCGCTCTTCTGTCGAAAGATTGAATGCGTCTAGCACACGTGGTGCATCGTTGTGCTTTCTCGGCAGTGC

AACCGACACCAAAACCACGGAAGCTCCCCCTTCTCAGCAGTTCCATTCTCCCGGTCGCGTCAATGGAACAGCCACAACTT

TGACAGCGGAACAACTGGCCCGGATAGAAGCTAACCGGCAGGCGGCGTTGCGTCGGAGAGCTCAATTCCAAGCGAATCAG

AAAGCTTGACAATTTCGCAAACATGATGTAATATTACAGTAAAGAATAGAATAGAACCAAACAATTATGCATCATGCGAT

ACATAAATCGTAATAGTACGTACCTTGTTGAAAATACTACTGTGTCGCACCCTTTCTTCCACAACCATCACCCTTTGGTT

TGCCAGGAGAGGCGAAGAACCCTGCTTCGACGCACTGCTGAAACATGTCGATGATCGTTATTCAGAGCGGCGTGTAGTCC

CCCAGTTTGAGTTCGCAGGCTGGTTTCTACGCGTCGAATTCGTGGCATAACGATATTATTCTTTACGGACCGGGACACCA

TCCTATGGATCATCTCGAGTTGCGGCGCCACTAGCCAGGCGACATTATTTGATGGATTTTCCCCAATATTCCGATTGCGC

TCTATCATAATTGTTTTTGTACCAATAGTACGGGCATTGTAAATGGTATTATGACTGATGTGCAGAGTATCTTATAAAAC

TACTGTAATTCTGTATTACGTTACTATCCATCACCTGATTTCCCGTAATTAATTTTGCTCATTATCTTCTAAGATCCGTG

GAATACGTCGCGCTCATTTCGCTGTCAGTCATCTATTCACATTTTCTAGATAGCATTCTTCCCCGAACCGTACGTTAGAA

CAATCAACTCATTACAACACTGGGATTAAGTAAGTATCAGTTTGTCCGCCGATTGTTGTTGCTTTCTGTCGCCATCGTCC

ATGACCTATGTTGGTGGTGGGCGGTGGTGCTATTGACGTTGCTGGCATTCGTTCTCGTGTCGCTGCTCGTTCCGCTGAGG

TCGGACCCGGCAATGATTGATGGAACACTGCATCATACCGCAACCCACCGCCTCCGCTTGTCGTTGGAAGAAAAATTGAT

GATTACAAGAAAGACTATTGGAGTGCAATGGTATTTGAAGACAAGTATACGAAACATAACAGGTAGATGATTTCATTCGA

TCGTCTTCTTTTGGCTAAATAGTTTTTGGTTTTAGCTCTCAATCCGAGAGGCGGCGCTGAAATCAAAATCCCCAAAGCGT

TGTGAAAAATATAACGTGCACCACGTTATAATTATTCACTTTAGGTCACAAAATCACCTAGATATTTGTTCTTCTGCCGT

CAACTTTTCAAAGAAGGGGTGCATCAGTGCTGCTGCTGTATCTGGGCGTTTGTACGGGTCAGGAATCATCATGTATTCCG

CCAATTCGTATGCTAGGGCTGAATCCAGGAGCCTTGACCTCGCTTCTTCGGATTTGTTGCTCGTGTCTAATTTTTTCAGG

GACTTCCGGAGCTTTTTCAACGTAACTCCACAATTTTTTTCCAGAAGCATTTTCAATAGTCGACCAATCGTGTAAACGTC

GAAGGCATAGACGGAGGTATGCACAGACGAGGTATTATGTTCGGCCTCGGGTGGAAAAAGTTGGTCTGGTGCGCGATCGT

AATGGATGGGCACCCTGTTTGGCTCATATTTAAAGGCGGCGTTCCAGTCAAAGAAAGAGACTTTGTTGCCGTCCCAAAAG

TAGTTGTTACCGTGAAGATCGCAATGCATAATGTTGCGAGAGTGGACGAATTGCATCGTTTCCAACATGGACTTCATCAA

TCTCCGAACATCGTCGAGACCCTTCGGATCTACCCGATTCTCCAACAGGTCAATTACGAGCACACTGATTCGTCGCTTCT

TGGTCAAGCGCCTGGCACTGTTGGAAGGAATTTTCAGGTCCTTTTTCAAGTAGGTTTGATTTCCTGCAAAAAAGGGATTA

GGCATGGATCGAACTGCATAATATAATTTGGGAATATTCGAAATGCTGGGAGGTGCATTGAGCTCGCGGAAAAAATCTAT

CTCTATATCGGAAAAGTACAATTCATCGTCTGCGGCAATTTTGGCAACTACCGTCTGGTTTTTGTACCGGGCCTCAAAAG

CGTAGCTGACGTCCCCCTCTCCAATCATCTTAATCAGTTCGAGAT

>PSNMU-V1.4_AUG-EV-PASAV3_0006800.1 class=Sequence position=[PsnmuV1.4_scaffold_11-size_341144:148287..150415 (- strand)](http://gbrowse255.tgac.ac.uk/cgi-bin/gb2/gbrowse/maplesod_psnmu_v1_4_gbrowse255?name=PsnmuV1.4_scaffold_11-size_341144:148287..150415) *FEN1*

AACTATTGTTGCACCACCCGCAGCCTTGACCCACCAAGAAGGAACGGGACAGACCATCCGCTCTCCCACCGACATACGGT

ACGATACGATACCCGTGTTCCAAGCAGTCAGCACTCTCATTTGACACGAAACTAGGAAGAAAGCAACAAATTATTTTGCC

ATGGGAATCAAAGGTCTCGCCAAACTGCTTTCCGACGAAGCACCCGACGTGAGTGAAAGGAAAAGAAAAGAACGAACACG

AGACGGTTGTGTTGATGTTCAGCGGTGCAACCCATTCCATGTGGCACGTGTGCTGGATGGATTGTTTCCAGAATTGCCAC

CGGTATCGTTTTCTGTTGTCACCGGTAGGATTTCGTTCCATCGATTCGTGCTCTTTCGTAGTCTCGTCTCATGTCTTCTC

AACCCGTCTCGCCCCGCCAACTATGACGAACGAACTGTGCTTGCAACCAACAGTGCATTCGAGAGGTCGAATTCAAATCT

CTCCACGGACGAAAGATCGCCATCGATGCCTCCATGGCCATATACCAGTTTCTGATCGCCGTCCGGAGCGGTGGGCCAAA

CCAGCAGGCAATGATGCTTACCAACGCGGACGGAGAGACCACGAGTCATATCCAGGGAATGTTCAACCGAACTATCCGCT

TCTTGAACGAGGGCCTGAAGCCCGTCTTTGTCTTTGACGGAAAGGTACGCAGCATGTATCTTGTATTTTGTTGTCCCTTT

CTCTTTTTGTGTCTTCAATGTGTATTCGATGCGGCAAGGCGTTGTATCTAACCCGACGTACGCACAACAAATTCAATCCA

CACATCGTCGGTGATTCCACAAACCACTTCTACTGACAACAAAAAAACAACGCGATCTACTCCGCAGGCCCCCAACATGA

AATCAGGGGAACTGGAAAAACGCAGGGAAAAACGGCGCAAGGCCGAGGAGGAGCTCAAGAAAGCCAAGGAAGAGGAAAAC

ACGGACGAAATCGACAAGCACTCGAAGAGGCTAGCGAGGGCCGGACGAAAGGAAAACGAAGACTGCAAGCGACTGCTGCG

GCTCATGGGGGTTCCCGTGATACTGGCGCCTATGGAGGCGGAGGCCGAGGCCGCGGCCCTCTGCAAGGCCGGCAAGGTCT

GGGCGACCGGGACGGAGGACATGGACGCCCTGACGTTTGCGACCCCCATTCTGATTCGAAAACTAACCTTTGCGAATCAG

TCCTCCAAGGGGGCCCAAATACAGAGCATGAACTACAAAAAGGCGATCGAGGGACTCGGGTTGACCCACGATCAGTTTGT

CGATTTGTGCATCCTTTTGGGATGCGATTACTGCGACAACATCAAAGGAATTGGGCCAAAGACCGCGTGAGTGAGAAAAC

CATACTCAGTGCCGATGTTCGTCTGTTTTGCTCACCAAGGCTTCCAGGCCGGAATTCTCGAACAAGGAATCCGTTTTGCT

CACCGTTGCTTCTTGTTTTGCTCGTTGTCTGTTCTGTCGCAACAAATGATCACCGCAGCCTCAAGCTCATTCGCGAGCAC

GGCAGCATCGAAAAAATACTGCCGGTGGTGAAGGATGTTCCGAAATACACGGTCCCCGCGAACTGGATCCCCGACGAGAA

GGACAAGGACGAAACCACGGACGAGGAGGCAGAAGCAGCGAAAGAGGCAACCACCCCGGCCTACGTGCTTGCCCGGGACC

TCTTCCACAACCACGAGGTCGACACGGACGTCGAGCTCAAGTGGAAGGAGCCACAGGCGGAGGAGCTCACCAAATTCCTG

GTCGACGAAGCGGGCTTCAACCCCGAGCGGGTGAAGCTCCAGATCGAGAAGCTCAAAAAGGCGCACGCGGCCAACCGCAA

GCCCCAGGCCAGGATGGACTCCTTTTTCAAGGTCGTTTCGAACCCGGCGGCCGACGCCAAGCGCAAGAAACGACTGGAGA

ACGAGCGGGCCAAGAAGAAGCAAAAGGCGGCCGAGGATCGCAAGAAAAAGGGCAAGAGCAAGAGATAAACAGGCAACAGC

ATCAACAAGTCAACAGTTCGAAAGCATGTATGCACTACCATAAGCGCATGAGGCATTGTTGTATCAAACATATCTGATTC

GTTGGGAACGAACAATGAAAAGCATAGAAAATAATAAAAACCAAATACT

>PSNMU-V1.4_AUG-EV-PASAV3_0067080.1 class=Sequence position=[PsnmuV1.4_scaffold_311-size_61866:7576..12434 (+ strand)](http://gbrowse255.tgac.ac.uk/cgi-bin/gb2/gbrowse/maplesod_psnmu_v1_4_gbrowse255?name=PsnmuV1.4_scaffold_311-size_61866:7576..12434) *EXO1*

GACACAAAGCTTGTCCGGTACGGCACTCGTTCCACTGTGTCCAGCGTTTCGAACTCAATCCTCTCGTCCACCATCGTCGT

CATCGAGAAAGGTAGCGATCAGAGTTGATCAGCGTTTCACAGAGATCGCGATTGGAAGGAGATGGAGTGGATATTGTTTT

GCCCCCCAGTGTCCAGTGCAAAAATTGAAGTCGCTGATCTTCACAACGTCAAAGGATTCTGCACCGGAGACAGGACGCGG

GAATCAGTCTCTGATTTAGTGTAGTTTGCCATCGAAAGTGCAGCTGAATATTTTCCTGAACTCAGACCGCGTGCACTGCG

ATTCCCCAAGCCAGAGACAAGATGGGGATCAAAGGCTTGCACAAGGCGCTCTCGTTCTGTACCGTCAAGGACAATCTTCG

GAACCATCGGAATTCTATCATTGCTGTCGACACGAGTTCGTGGATGCACAAAAGCGTTTATTCCGCCTCGGAAAAGTTCG

TTGAGGCCACGAATAATGGACGTGTGGACCACGGGTGCGTTCGTGTTTCTGCGAGATACATCATCACGAGGTGCAAGGAG

TTGATCGAGGCCTTCGGGATCAAAGCGGTGTATTTAGTGATGGACGGCAAGCGGATCCCTCTCAAAGCGGAAGAAAGCCA

GGATCGCGATCAAAAACGCCTACAAAACCTAGCCGAGGCAAGAAGGCTCAAGAGGGCCGGCCAGAGATGGAAGGCAGAAG

ACAAGTACAAGTCGTGCATACGGATCAAAGACAACTTCACGGAAGCCGTCATCCGCGAGGTGGAAAAGGCCTTTTCCAAT

TACGGTCGCGTATTTTTTGTGAACAGTCCCCACGAAGCCGATAGCCAGTTGACTCGACTGGTGCTAGACGGTGTCGCCGA

CGCCGTGATTACGGAGGATTCGGACGTCTTGGTGTACAGTGCTGCCGCTCACAAGCCATTTCCGATCTTGTTCAAACTCG

ATCGCAGGACAGGAGCTTGCGACAGCATCAACATGAAGTGGCTGATATCACCATCCTCACAGGAAACGGCAAGGGCTGTG

ACAAGCAAGAACACACTGGAGTTCATTCTTCGGCGTTTTGCTTCGAAACAAGTAAACACGAAAGGACTTGGGGTTCGTCT

TTTCGTTCAGGGGTGCATATTGTCGGGTTGTGACTATAGGAAAAATATCGAAGGCATAGGAACAACCAACGCCTTCAAAC

TGGTAAGTGAGAACGCATTTCGCGGTGCTTCCGTGCGATTTCGAAAGATTCTCGAGTCCCTTCCGAAAAAAGTGCGACAG

AAGATCGAAATCAACGAGCATGAAGAGATCCTCGCCAAGAGCGAGGCGATTTTTTTCTATCATCCTGTCTTGCACAAAGA

TTGCCAAATCAAACCGCTTTTAGAGCCTCGCATTTCGCCAGAAGAAAGCAGCGTCGGAAAATGTCATTCGGATCACTATC

CGTGCATGTCTCGATTCAAAGGAGATTGGTCATTTTTGGGCAATATTGACTCGTCTTCGAGTCGTGTCATTCCGAATAGT

TACGACGCGAACAACTGCAATACTGTTCAGAAATCAATGGCGCCGTCCATTCTCAGTAAACCCAACAGTATGTCATCAAC

TCTCCCGACGAAAAACCCAGATTTTTCACACTCATCAGCGTCGAATCGATCACAAACACTGCAAAATCCGTACAAGAAAT

TCAAAATATGCGACAACAACAGACAAAGGCCACTAGGAGAAAGGAATATCAACATCCGATTCAATACCGAGAAGAAGCTC

GAAACTCAGAAAAATATGGGTACTGGCAGTATCACCAAATTCTTAGTGAAACCTGATCCGAGGTATGCTAAGAGAACATT

TTCTTCTACCACGGTAACAAAGAGAGACACCCAATGTTCTAGATTGCGGAATTTCTCAGTTGCAGGCTCCAGCTTTCAGC

TTTCATCTCGTTCAAACGCATCCCAAAAACCCCCCGGCCAGTCATTCTTTGAAAAAAGAGCAACGAAGAAAACACATCAT

ACCACTTCTAGTAAGACTACCAACTCTAGAATAGAACAGCGCCGTTTCGATTACTCTCCCATCGAAAGCAATGGAAAGAA

GGAAATCTCCTTTTCGTATCCATCTTGCAACTTGCCGAGCCCTTCCGAATGCGAAAGCATACGGACGAAACGTGCAAGTT

CAGACATTAAGCGTGACGGATTGAAAAATCTTGATCGAAATCTTCAAGATTTTTTCGATTTGACCAATTCCAATTCTTCC

GACATGGACAACTCAGAAATCCGGACGAAAATCGCAGAGACGAAAATGGAAGCAACGCTCTGTGACGACCAGGCTGTTTC

TGTGGTCCCATCGAATGACACATTCGAGGAAACAGAGAAAGAATCTAATGCAATCGGAGTTAAGATTTGTTCGACGTTCA

CTTCGGAAGAACCGAACGATAATGAGCGAGACCATACCAAGAATGCTGGGAAAGCAACCAGCAAGTATTTTTCGAAGAAA

CGGTCGGACCCCCGCAGAGTGACACTCGATTCTTCTATCAGGCGTCCGTGCGATGGACTTCCGAGTGTCGCATGCACAAC

GCCGGTACGACCTCAATCTATAGACGATGAAATGAATTTACCAGCAACACGAAAGAATGAGTTGCGAGACTATGAGTGGG

CACTGGACGAATGCATCGATGATCCGGAGGAAGAAGTTCAAGTGGCGAAACCAAAATCTGGTTTCGAAAGCGGCTCCTTT

GCCAACAAGTGCAAGATAAGGCATCCATTACCACAAGAAAACAGCAATAAATCATGTTCCGAGTCTAAAGGATCGATCCG

ATCCTTCTTTAAGCGCCAAGAAATATTTGCGAATCAGTTGAAGAGAAGCATCGAACCCAATAAAAGATCACAGCCAAAGA

GGTCGAAACCAAACGACTTTTTTTCTCCCGACAGATATTCGAAAAAAAAGAAATCGAATAACAACAGCTCTCACCGTTTC

AAACAGAACCCTTCTACTGATATCCTCTCGAAAACGAAAAGATTCGCTCTCTCTCAGTTGGACTTGAAAGGGGGACAATC

GCAGTCTTCGGACGACTATTTATGGAACGGTACGCCGTGAGAATTGTGCGGTACAAACAGCCGTTCAAACCTCACAGAAA

AGTTCAAAATCGGCACAAAAAGTCGAACACAAAGATTCGACAAACTACAATAGAGTAGACTAGAGAAAAGCGCATCCGTG

CAACACAGATTGTAGCAATGAGTGTCAACAAGCAAACAATAGAAGTATTAGAAGAGAGATCGCCTTCTGTTGATTCTCCG

TCGACTGATTCGCTCGCTACCTTCCTCGTGGTTTTCTAAGCCGATTCTTGTCTGAAGAGCTTCCAAAAATTCGTTTGCTT

CTTCACCCTGCATGACAATGCGATCCACAACCGTATTGAACGATGAACAATTGCGACACTGGACACCGAGGAAATGCCAA

GCCCGGGCATCCTCCGATTGTTCGCAATCTATGCAAGAGATGTTCACTACTCGTGCCAGTTCCGCTGGAACGGGTTGCAT

TGCCACGGCCTGGGCCATCGCATTCCACGTCGGAATCATTCTCTCTTTCGTCTCTGCTGTTTTCTTGCAAATGGGACACC

GCGAGTCGTGGGCCGCTAGCTGTCGAAAGCACTCCCAATGAATTGCATGGCCACAGGGCATCTCGTGAGAAGCGCTCCGA

GAACTAAAGAGAAACTCCTGACAAACTGGGCAGTTCGATTTGTACTTCCCGCTTTTGCAATTATGATTGTCGTACAATGT

CTTGTCGATGCACATACCACAGTCGTGACAATGCTTAAAGTTTTCGGCACCCCCAACCCTACAAAAACCACAGTCTGGGC

AATGATAAGGTTTTTCCTCGTTCGACATCCACAAGTTGCAAATACTGCAATGATATTCTCCAAATTCAATGCCACAAGCG

GAGTTGACGCATTTGTTTCTTTGGTTCGAGACAAGACGAAGTGGAAAGGAGGAAAAATTGAGCAAACCAATCAAACGAAG

CACGAACATTGCGGATCCAAGCGCTGTTCTAGCAAGAAAGTCTTGTTGTGATAAAAATTCAAAGATGCATAGGAGCGCGG

CGCAAGTTACATACGTTTTGGAACTCTGTTTGGTATAGCATTTGCGACAAATGATGTCCTTGACAGCAAATCGATCGATT

GTGTGATGCGTCTCTTGAGGTGCGGGATTGCCATCGTCCATGCTGGTCCATCCTCCGGGCATGGACGAGCTTCTTGTGGA

ACGCCGCGATGCCCGCTTGGCAATCAATAAAGGCGGCAGAATAGGGCACTCGTCGTGGCAAATGCGACAACCAAAGGCCG

CTCCGCAACAGGGAGCTATCATGGTACAATTCCTGTCGTAGTGGTTGCAGGGCTGGCGAGTAAGCTCGGCGCGCTTGGTG

TTTTCGTTAGAAACCGGGATGCAAATTGCATTTCCCGAGTCACCGAGGATTTTGTTTTGACTACTGTTGGTATTGGCGTT

CATTTCGGTCTCGTATCCATCTGGAGCCCCATCGCCATAGCCATATATATCTGCGCTCTCTGAACTCATAGAAGCTATTG

ACGCGGTTCCGATACTGCTCCTCCTACCGTCCATCAGACTCTGGATCGATCGTCGTCGCTCCTGTGGAGTGATGCTGCAG

TCTGCGAGAATTGCTTTAATGTTGGCTCGCCGTTCCTTCTCTTCTTCGTGAGACATGGCTCGACGCCTCCTGCCAACCAT

TTCTTCGCCATCGCCATCGCGATCGCGGAGCCTCTCGGTCGCATCCGATTCCGACGCTATTCGCACCTCTATAATTTCGC

TATCACTTTCCTCGTCTTCCATATCGATATCCATCTTGAAATCAATCTCAACAACCCCA

>PSNMU-V1.4_AUG-EV-PASAV3_0027070.1 class=Sequence position=[PsnmuV1.4_scaffold_162-size_122010:85016..90960 (+ strand)](http://gbrowse255.tgac.ac.uk/cgi-bin/gb2/gbrowse/maplesod_psnmu_v1_4_gbrowse255?name=PsnmuV1.4_scaffold_162-size_122010:85016..90960) *DNA2*

CGTTCGATATATTTGGTTTGGGCACCAGACTTGGGTGTTGCATTTGAATCTAGACCATAATGGATGGAGACGGATATGAC

GATCCAGAAATTCGTGCTACTGTCGACAACGACGAGTCAGATGCATGTCTCTATTTGAATGAGATGACCCAAGATGTGAT

CGAATGGGACGAAAGCAACAACAAGGATTTGGATCAAATGTTCAAGTCTCCCATGCCGCCCAGCAGTGGAAGGAAAGGCG

CTAGGAGCAGCACAAGGAAACGAAGCAACAGCACGTCTAGTACGAAATGCAACAAGAACGCAAACAAGAGGAGTACTCCT

TCGTCGAAAGCGACGACCAATTCCCCCCTGTTTTTGGGTGTAGGACGACAACTCGATCCTTCTTTCCTAAGCACAGTCAA

TACTGATGGAAATAGAGCAGAAAAATGCGCAGAGCGAGAAATCGATGGGACCATAAACTCTCTCAAAATGAAAACTGACT

CGAAACCACTTGTGAACGCGGACACAAAAAGAACTATCCACTTGTCATCCATGTTTTCAGATCCTCTCGGAACAACACCA

ATACGACAGGGGAGCAAACGACCGGCAAAAACAACACTCAATAGACACCACACATATCCAAAAACGAAGTCTTCCGCTAC

CTCTCTGGCCCAACGTCACCGACGTCGACGGACGGATTATTCACGAGCGCAGAAGGAGCATACAAACAGTGCCGACGCAC

TCCTGCCATCGGCTTCCATGGCTGATTCTGCCTTGTCACAACCAAATAATGCGCTTACAATATGCAAACATTCAGGTCCA

ACATCCAGGGATGGAAGGAATACACAAAATGTTTTAGTACCCAAAGAGGCAACTGCACCGGACGATTTTGCGGAGCTTCT

CGAAGCAATCCGAACGCCCCATTCGCCCAATGAATCAAGGCCGCTTGGACGTTTACCTCTTTCTTCGGTAGACGAAAATG

CAATCGCTATTCGCACGACAACTGTGAATAGTATAGAGAGGAAAGACACGGATTCGTGTTCCACTGAAGATCAAACGAAG

GGTTCTTTGATCGATTCTGTCGATACKTCATCCGAGGTAGTACACAGGCCACAATCGGCGCTTTCGAAGCAAGAACCTTC

CGCCAATATAATACCGCAACTACGACACTRTAGTCAATTTGAACATAAAAAGACGCAACCAAAAATATATTCTGGAATCT

CAAATCAACACGAGTCGCCCATGGTTCTTCAAGTAGAAGCAAGCCATTCGCGGCCTGTAAATCGCATTTCGTGTCCAGAA

AGAAGGGGAAACGACGGTGTAAGGGACGCAGGAAAGATGAATGGTAGCGTTGACATATGCAAACACAACAAACTGGAAGA

GTTTGACAATATTGATTTTTCTGAAGATGACCTAGCGAAAATCGATTCCCTGACACAACCAGCGCTAGGGCAAAACAATA

AGCAGATGTTGCAAGTGCAATCATCAGAAAGAAAGAATTCCGTGGAAAATATGACGAAAAGGTTTAATACGAACGAGAAT

GCGCCCGCATTGCAAGGAAATACATTGGATACAAGCACGAAAGCCAAACCATTTGATTCGCACACCACGAAGCGTGATGT

TGATGAATTTGGTGACTTTCCATCCGATATCGACTTTGCCCAAATTGATGCTATGGTTTCTTCTCAAGCATGCAGATCAA

GAACAGAAAAGAATACATCTCTTGCAAAGGCCTCGCCACAAGCGTGGGAAGCATCGTCTACCGAAATTCTTGCGTCTTCG

GCGACTCATATGTTGACATCGAGGTGCGAAAAAACACAACTTGTTACCTCTTCCAACATGATTCAATTACCGAAACAGAA

TTCGACACCCGAAGAGGAAGACGAATTTGGTGATTTTCCTACCGATATCGATTTTTCTGAGATGGATGCTTTAGTTGCTA

CTCAGTGTTGCAACATAAAACATGATGATTCGAAATCATYTGTACGCTCTTCTATCTCAACAAAGCATTCAAACGAATGG

AAAAATGATCCGTCAAAATCACAGAATCTGACTTCGACAAAAACAGAGGATGACGACTACGGTGACTTTCCAGAGGATAT

CGACTTTGACTCCCTTGATCAGGCTGTCACTCAAAAAATGCTCTCGAAACCCGAAGCATCGGTGGACCCTACGGGTATTG

TTCGCAACCGGAGAAGAAGCGGTATCAGACCTCGAGAAATGTCGTTCATGAAATTTTCAAGGTATAAAGTCTTGGCCGTT

GAAAACGATCAAAAAAATTGTATAAAAATACTGAAAGTTGCTGGATGGGTAGAGTCAATGATGAAGATACATGAGGAAGA

AAAGAAGATTCACAGAGATGGCAAGGTCAAAAGATCGGATGACTATGTTCTCGACGAAGACAATAGTTGTTCATTTAGCC

TCCCTCAAGATATTGAGTACCCCGAAGATGGAATGATATACCTCTGCGGAGAATGGACCTTCACTCCAGTCTCGCCAGGT

GATTTTATTCATGTATGTTCACTGACAGGCCAGTTCGAAACTGATACTGCAGCTCTTCCCATTGTCCTCAACTCTTACCC

TCCGCCGGGATCTGACATTGATGACCTGATTCTGGTGTTACACCCAGATATGCTTTTATCACCGAGTATAATCAGCGAAG

CTTCCTCTTGTAACCGACGGGCTGCTTTGAAATCTAAATTGGGTTCGACCGGATTGTCATCGAAGTCAGCTGTTGTTGGT

ACGATGAGGCACGGACTGTTCGAAGGATGTATGAAGGCAGCAGAATTCAGTCCATCATTTGCACAAAAAGTGATGAAGAA

GTTGATTAGAGAAAAAGCAGAGATGCTGATCGGCTGCAATGTTTCCGAATCCGAGGCTGAAGTCGAAATTCTTAAYATAC

TTCCAATGATTCAACATTTCGCAGGGCAATATACAACGTTGAGAAAGGATGTAATGGTGCTATCTTCTCTCGGAAAGCCT

GTAGGGGGTGCAGCCTGTCATCCAGACATTCGTTTATTAGGTACAAGAGTCCATTCCGTAGAAGAGAACATTGTATCAGC

AGCAATAGGACTCAAAGGATCAATTGATGCTGTTCTCGAAACCCAATCCGAAATAATAGGTCGCAAACAGCAAAGCGCAT

CATCGCCAACCGTGGTTGCTGCTCCACAGCATTCGTTGATGTGCTTTGAGTTAAAAACAGGCCACAATCAGAATATTCAA

AATGTGCATATGGCTCAACTTTCTCTTTACACTTTTATGCTTCAGAGCCGATATGGTGCAGATATTAAACTCGATAACAA

TAAAATGATTGGGTCACAATCCGAAACAAAGTTTGGAGTTAGAGGGGCTGCCCCAGGCGGTATTYTGTTATACCTTAACG

AAAAGTCACAGCAGATTTCTCATGTTTCGCCCCAGCTAAATGAGGTAAAAACCTTGATGACTCAAAGAAATGTCGTGGCA

AGTGATTCCAAGCGTTCATCACGCCTTAGAGGAATTGCCCTTTCCTATGACGAGGGACACCGAGAGGATGACAAAAAGTA

TCTGAAGGCGAAACTGCTTCCTGCTCCGCCCGCTGATCTGCCGGAATTAAAAGCTAGTTCACACCCCTGCAAACGGTGCT

TTTCTAACAGGGAGTGCATGGTTTATGCTGCTAGTGACCCAGCGACTGATACAGGAAGGCATCACGAATTATTGTCACAG

TTTACTGGACATTTAAAAGAAGAAGATCTAACATACTTTCGGAAATGGGATAGGTTAATTGATATCGAAGCAGACGCATC

CAACCCAAAGATAGTGGCACCATGGCTTGTAGATTCGCGCATTCGAGAAATTGAAAATGGTGAATCAATATCTCGTCTTG

TTTTCGATGCAAATACGTCTTTCAAAGTGGGAACTTCGCATGCATTGGTCTGTTTTCGACGAAAGCTCGAGACATCGTCT

CAATCTCCCCTTGAAAGACTTAACATAGCACCTGGCTCATACGTCATATTGAGCACAGATGGAAACATTTTTGATGACTT

CAGCAGAACTACTCATGGACCTAGGAAACATCGGAATCAAATGCATGTCACAAAGGGTTATCTTGACCGCATCGAGGACG

ACAAAGTTTTTCTATCGATGACCTCCGATGAATTGAACCAGGTAGAAATGCTCGTGAGTCGATATCAAGACGAGGTGCAA

AAAAATTCCAACGGAAATAATTTATCCTCGTCTGTATTGTTCCGTCTTGACATGAACACGCGTTCTGTGGGGACTGGAAC

GTTGAGGTGGAATCTCGTCAATTTCTTATCTGGCGATTATAGCCAGAAGAATAGAGAAGAATGCACCGAGTTAGAGCAGA

GACGACAGCGGAGGCTGGTATGGCTTCGCGATATTGTCATCAGGTTGAAAACGCCTGAATTTGCCGATGAATCTGATGCT

TCTTTTTTTGAGGGCATTGACCTTCATATTTCTGGATGTAACCTACAAGAATTGTGTAGAGAATTTCATCTACTCAATGA

CGATCAAAAACTGGCAGTGAAAAAAGTACTGAGTGGTAGGGATTATACACTTATTCAAGGATTGCCGGGGACTGGCAAAA

CTTCAATTCTAGGGTTCTTGGCTCGTCTTCTGGTTGGACGAGGACGTCGTGTCCTTATCACCGGTTACACTCATTCCGCT

GTGGACAACATTATGTTGAAGCTTATGGAAAAAGGAATGAAGCCTTTAAACTCAAATCCGGGAGTATCGTCTTTGGTTCG

AATTGGACAAAATCGGTCATGTCATACAAGTGTGAAACCAATTATGCATTCACATCTAGCTCTTGAACAAGACAGAATAT

TGAATAATCGAAATAGTTGCGAGGAGTTGGCTCACGACGATGTGGAACATCCATCGGCAACAGCATTGAAGACAGTAATT

ACAAATGCCCGCATTGTGGGTGTCAGCGCTTTGTCCCTTCCTCGATCTGCTCTTCTAGAAAATCAGTTTTTTGATGTTGT

CATAATTGACGAGGCAGGCCAAATGAATGAGCCCACGGCACTTGGAGCCCTGGCGGCAGCTGACTCGTTTGTTCTTGTCG

GAGATCATAAACAACTTCCTCCATTGGTTAATAGCGCAATTGCAGAGAGTGGTGGATATGGAGTCTCCTTGCTTAAAAAG

TTGGCAGAGAAGCATCCCCATGCAATTGCCCCATTGACAATGCAATATCGAATGAATGAAGACATTTGCAAGATCAGCAG

TGAGTCAATGTATGGTGGTAGGATGAAATGCGGWGATGAAAAAGTGAAATCGCAGCAACTACACCTTCCTGGTTTTCCTT

CGTTACTACCGAAATCTGTCAAACAACAACCCACGGACTGGCTACGTACAATAATTGATCCAGATCGCCCTGTCGTTTTT

GTCGACACGGACAACTTGAGGACAAAATTCTCAGAGTTTGACGGATCCACTGAATCAAGCAATCGAAAAGAGAAGTATGA

AACTCTTGAAGGAAAAATCGGGGGTAAGGGTAGTGGAAGTATTATCAATAGGGCAGAAGCTACACTAGTTCGGTATATAG

TGGGAGCGTTGCAGTCTTGTGGCCATGATCTCTCCGAAATTGGAGTCATTAGTCCATTCCGCGCTCAAATAAGGGTTCTG

GGAGAAAATCCTACTTTGTTGTCATGGAAGAAGGAAGGCCTAGAATTGAGCACAATTGACAGGTATCAAGGCCGCGACAA

ATCTACAATCATCCTTTCATTAGTACGCAGTAATGAAAAGAACAACACCGGGCGCCTACTCCAAGATGCGAGAAGGCTAA

ATGTTGCTTTTACGCGAGCCAAGTACAAACTAATTGTCATAGGGTCTTATCGAACCCTGACTCTTGGCAGCGCTCCCCTG

AGACCTATTTTGAATCGAATGAATATGAGGGATCAACGGGTTGAACCTCCAGAAAATGCTTTGGGATGCTACGACATGAA

CTAAAGACTTCTCAAACGTGACTTT

>PSNMU-V1.4_AUG-EV-PASAV3_0067160.1 class=Sequence position=[PsnmuV1.4_scaffold_312-size_67119:12307..20415 (- strand)](http://gbrowse255.tgac.ac.uk/cgi-bin/gb2/gbrowse/maplesod_psnmu_v1_4_gbrowse255?name=PsnmuV1.4_scaffold_312-size_67119:12307..20415) *BRCA2*

NNNNNNNNNNNNNNNNNNNNNNNNNNNNNNNNNNNNNNNNNNNNNNNNNNNNNNNNNNNNNNNNNNNNNAACGAGAACGA

GAACGACCACAACCGCGACCATGGCGGACCGGAACTGGAACTGCGGGGCCTGCACCCTTTTGAATCCCCCGTCCAAGCGC

CGGTGCCTGGTGTGCGGAACCCGCCGGCCGGTGGCCAAGGACGGCACCACCGAGGGATCCCTTCCCTCGCGGAAGCGGCC

GAGGAACCCCTTCCCCCCTCCGAAGCGAGAGCAGCAGCAGCCGCAGTTACAGCTACAGCCGCAGTGGCAAGAGCCCCAGT

GGCGAGAGCGGCAGCGGCCGCAACGACCGGCGTCGCAATGGCAACAGCCCCAGTGGCAGCAGCGGCATCGCAAACGGCCC

CAGAAACAGGCGTCCGTGGCCAGCTACTTTTCCCCGGCACCCAAAAAAGGGCGAGCAACGGGGAGCGGAAGCAGCGGCAG

CAGTAGTGGAAGCAGCAGTGGAAGCAGCCCCCCTTTCGTTGCGGTGCGGCACAAGGTCACCCCGGCGCAGGCGTCTCCCG

CCGGTGCCAACGAACACGCCCTTGCCTCGGCTTGGATCGGAACCGACCCCGGCACGAAGACGAAGACGAAGACGAATGCT

AATGCCAATGCCAATGCGAATGCGAATGCGAATGCGAATGCGAATGCGAATCCAATTGCAATTGCAACGGAAAAGACGAG

CACGAATGCGGATGAAAATACGAGCACGAATGGGGATGCGAGTACGAATATGAATATGAATATGAATGCAAATACAAACA

CTACTAAGCCTGCAAATGAGTCAAACGCCACAGACACCAAACCATTGGATCGCGTCACCGAGGCAAACGAAGAACTGGAA

TGTGCCAGCGCTTCTGATGGGATTGTCGAAGAGAACGAGAATGACAACGAGAACGCGACAGAGATCGAGGTCGAGATCGA

GAACCACAACAACACCGACAGAAGTTTCAGGAACACCACCGATAACCAAAAGGATAACCAAAAGGAGCAAAACAGCGACA

AAAACAGCGACAACGACAACGTCGGGAAGAAGAATGAGTGCCAGGATGCGGGGGCAGAAGGGTCGTTGGCGTCGCTGCCA

TCGCTGCCGTTCCCGTCTCCGCCGTCCGAGCCCAGCCCTTTTGCCTCTCCCGTTGCATCCCAATCGGCGGTCGGTCCCGG

GCCCGGGGAGGGAGAGGTTTCCGTTCGGGAACAAGGGACGAGAGAATCTCCGGCGCCCCCCCCGGCTTCCCAAACCAAAG

GGGACCGGCGGGTTCCATTGGTGCCCCACGGATCGCCAGCGCCAGCGCCACCGCCACCCCCAGCGCGGTCCGTTTCCCAC

CCCGGTGGCGGTGCCGAGGTCGGTTTCGGTACCGGAGGCAGCAGCCCGGATGCCAGCCTGGACGGCAGCCCCGAAGGGGT

CCGAACCCCGGCGCACCCGGCAGCGGTGGATCTCCCTCTTCCGACCGCCGGGTTCGGCTTTGCCTACACGCCGCCCTGCT

CGCAGTCGCAATCGCAGTCGCAGGCCTCCGCGGGGGACCTCCTCCCCGAGACCCAGGAACCGGGCGGTGCCCAGCACCAC

ACGCCCCGCCMCTACCGAACGGAAGCGGGGAGATGGTTGCCGGCCAGCGGCGGCAGCAGAAGCAGTGGCAGTGGCAGTGG

CAGCAACGGTGGGAACAACAGCGACAACACCGACGCATCCTCTTTGTCGACCAACCGGGAACGGCCGAAATGTTYGACCG

GGGACCGAGCGAGCTTCGCGTCCGCGGCAAGAAAGAGACAGAGGCCGATGCCTGCGAGAACCACGTCAGCGACGACGGCA

ACCACCACACCGGCACCGGCAGCGGGAACGACGMCGACCACGAAACCATCCGCGGCAATCTTTCACACCGCTGGCTCCGG

GGCTTCCATCGAGGTTTCCGAGGAAAGGATCGAGGAGATGGGGAGACTCCTCGGGAGGCCAACCCCCTCGTCGGTCAGGG

AAGCACCGGYGCCCGTTCCGCGGAGCGGTGTTTCACCCCCACCGGCAGGSYCCCCCCCGCCGSCCACCACCCGGKCGGST

WTCCCGCCCGGGGTAGCTGTTTCTGCGGCGGGGGAGACAGACATTCCCSAAGAGCTGCCGCTTCCGAGACCGGGACCGTC

GCSGGCGGAGCACTCCGGGTTTCGGCCTCTCGAGAATCCTCTGGCAGTGTTCCWGACCGCTGGRTCTGGAGCYTCGATTA

CGGTATCAGAAAAAAAGATGAAGGAGATGGATAAAATGCTAAACGAACCTTCTTTGCCGAGCAATGGAGGACAAAGACCG

CCTACAGTTCAAAAGCCTGGTTTACTTCCCTCAAAGTCTCCTCTGGCAGTGTTCMAGACCGCTGGGTCCGGAGCTTYGAT

TATCGTATCAGAAGAAAAAGTCAAGGAGATGGATAAAATGCTAAATGAATCTTCTTTGCCAAGCAATGGAGGACAAAGAC

CGAATACAGTTCAAAATCCCATGGTGCAACCCTCAAAGANNNNNNNNNNNNNNNNNNNNNNNNNNNNNNNNNNNNNNNNN

NNNNNNNNNNNNNNNNNNNNNNNNNNNNNNNNNNNNNNNNNNNNNNNNNNNNNNNNNNNNNNNNNNNNNNNNNNNNNNNN

NNNNNNNNNNNNNNNNNNNNNNNNNNNNNNNNNNNNNNNNNNNNNNNNNNNNNNNNNNNNNNNNNNNNNNNNNNNNNNNN

NNNNNNNNNNNNNNNNNNNNNNNNNNNNNNNNNNNNNNNNNNNNNNNNNNNNNNNNNNNNNNNNNNNNNNNNNNNNNNNN

NNNNNNNNNNNNNNNNNNNNNNNNNNNNNNNNNNNNNNNNNNNNNNNNNNNNNNNNNNNNNNNNNNNNNNNNNNNNNNNN

NNNNNNNNNNNNNNNNNNNNNNNNNNNNNNNNNNNNNNNNNNNNNNNNNNNNNNNNNNNNNNNNNNNNNGGAGATGGATA

AAATGCTAAATGAATCTTCTTTGCCAAGCAATGGAGGACAAAGACCGAATACAGTTCAAAATCCCATGGTGCAACCCTCA

AAGATTCCTCTGGCAGTGTTCCAAACAGCTGGGTCTGGTGTTTCCATTGCAGTATCAGAAGAAAAGGTCAAGGAGATGGG

AAAGATGCTCAGCAAATCATCTTCATCAATTGCTGTGGCACAAAGACCACCAAAAGTGCAAAAGTCCATGGTGCAACCCT

CAAAGACTTCTCTGGCAGTGTTCCAGACAGCTGGATCCGGCGTTTCTATTGCAGTATCAGAAGAAAAGGTCAAGGAGATG

GGAAATATGCTCAGCAAATCATCTTCATCAATTGCTGTGGCACAAAGACCATCAAAAGTACAAAAGCCTGGTTCACAACC

CTCAAAGACTTCTCTGGCAGTGTTCCAGACAGCTGGATCTGGAGCTTCAATTACYGTATCAGAAGAAAAAGTCAAGGAGA

TGGGAAARATGCTGAACAAATCTTCCAGAAATAGATSGGGAGAAAKGGTTAGGGATAGGGGAAAAAYGATCAATGCATCT

TCTTCATCAACCAATGGGTCACAAAGACCATCGATAATTCAAAAAGCARATCTTAAATCGATAGCGACTTTTCAAACAGC

TGGGTCTGGCGTTTCTATTGCGGTGTCGGAAGAAAAGGTCAAGGAGATGGGAAAAATGCTCGATAGATCCTCTTTGTCAA

GCAATGGACCACCAAATTCATCAACACTGGAAGAATCTAGTTTCCAACCGTCAAAAACGGCTCTGGCAACATTCCAGACA

GCTGGCTCTGGAACATCTATARCTGTATCGAAAGGAAAGATCGAAGAGATGGGAAAGTTGCTCAACAAATTGTCCACTAA

TGAAGCAAAACGACCGTTGAYAGTGAAGAAATCYACTTTTCAACCGSCAACGGTTCCATTGGCAATGTTTCAGACCGCTG

ACTTCGGAGAATCCATTAATGCATCGCAAGAGAAGGTCGAGGAGATGGGAATGATGCCGAAGAAACCAARCATTGAAGCA

CCAGAATCACCGGCATTCAAAAGCAATCTGGGACCATCGAACACTGCTACTGTAATGTTCCAGAGAGATAGTTCTAGAAC

TTCCATATCTTTATCAGAAAAGGTTAAGGACATGGGAAAGATGCTCAATACGGTGTCCTCATCCAAGGCGCATCCATCTC

CCAGTACAGCAATCGCGTCCGAAAAAAGCCAAGAGAAARCATCAGCAATGCAAACGAGTCCTTCAATGGAWTTGTTCCGA

ACGTCGAAAGAGGGGGAAACGACCCTCAACGAGCCCTCTGCATCCGACCAGGAGAASATTACCAGCACGAGCCAATCAGA

AAATTTTTCGTTGACAACGATGCCAAGTCACTCGAAGCAACCAGGAATGCCAATAGCAACTGCGTCAAACGGCGTTCGAG

ACTCCATAATGGGAACAAATCGATCAATACCAACAGCTATGTTCCAGACCGCTGGATCTGGTGCTTCTATATCAGTGTCC

CAAAAAAAGATCGAAGAGATGGGTTTGATGCTCAGCGAGTCATCTTTTTCTTCCGAAGATCCAGGTTATAGTAAAACTCT

CTACGAAAGGCCGTTATTTACAGTGACTCAGGCAAGCAACTCATTCCAGACACCGAGAGCAGTAACAAAGACGAATGATG

ATTTTCCAGCCATTACAAATGAAAAAKCAGGGTCAACAAACTTGCCCATTCCTATGTTTCAGAAAATCTCTTCTRACACT

GAGACAGATGCTTCTAAAACGGAGCAGATTGAAGAAACAGGAAGACTCTCGCTTTGCAACACAGATCCAATTTGTCAGAA

AATCCACACGGATTACGAATTGTCGACTAGGACACCAATCACCGYGTTTCAAATTCCAAGGCTAGTAACTGCTGCCAAAG

ATTCAAATGCAGCTGGTAGGCCAACATCATTCTCTATATTCCAAACAGCAGGAAGCGGTAAAGCAATCGAAGTTTCAGAA

GAAACTGTCGAAAAAATGGGTTTCCTGCTGGAGAAGCCAAACTCATCAGATAACGCTTCAGCTAGTAAGAAAGTACCATC

TAGAGCAACGGAGCCTTCGTCKSAAAGTTWCTGTGCAACGAAGCCTGCCGAAGTTTCTACGATGACAGAATCAAACTATC

CAGAGAGAAGTCGTATAGATGGGCATGACTTTGTCGAGGCGGGTTCATCTTCCTACTCGTACTCCCCATTAAGAATAAAC

GAGGCGAAATCTGATTCGTCACCGTTGTTGAATCACGAAACAGTTACCTGCACACCACAAAAAGACACCCTGTCGACACC

CCACCAGAAAGTTGCCAGGTGGGACTCTATAAGACGCACAACATTTGGTATTACACCTCAAAGTGGTTCAATAAACAATA

ATTTAAGTTCGGTTCGTAGTGGTAATGGTGGGGCGTTAATGTCGCASTCGTCTACGGACRATAATGCCAGAGACAACACC

AATGTATTTGATGGCCAAAAAGCACAAGACGTGCATTTATCTCCACAAGGATCGAAGCCATCTTCTATGTCTGCGAATGT

CACCATGTCCTGTGACGATAGAACAATAGCCAGGAAAACGAAGTTAGATCGAAATTGCCTAATGACTCCCGTTCCATTCG

ATGTAAACACGCTAGATGGTGCGGAGACAAGAGTGAGGGAGCAGAGCCCTGCTGAGTTGACCCTTCAAAATGCGATTCGT

CTTGGTAATATGAGTACATGTCCCAGTTTGTGCCGACGTCATGGAGTAAGAGAAGTAACATTGATGATCAACTATACCAA

TTCTTTACAGTTGAGATTTGATAGTGACGGTATGCCTCTTGCTTTGGCGTCAAACGAAGAAGATTCACCTTCAAATCTCA

ATGGAAGRTGTGCAGATATTCGTCAATCCTTGATTGATAATGGTTGCGATGCAAGYCGACTCAAAGATGCGTGGATCCGT

AATCACACGAAATTGGTTGTCTGGAAACTTGCATGTTACGAAAGGAGYTTCTCTCAGTTTTTAGCAGATAAACATCTGAC

CTATCACAATCTGATACAAAACCTTRTTTCTCGCTTTCAAAAGGAAGTTATTAGTGGCCTTCGACCAGCGATTCGCAAAA

TACTCAACCGTGACGTTGCCGCAACCAAGATGATGATTCTTGTTGTTTGTCGAATACTTCCGTCGTCCGAATCTAAAAGC

AACGATACCTCACCACAATCAACAAAGATCGAGCTTAGCGATGGGTGGTATTCCGTAAAAGGCTGCCTTGACGCTAACCT

GTCGGAATRTGTCAAGAACGGTTGGATTAAAGTCGGAACGAAACTACTTGTTTCGAACGCCCGTCTAGTAGGTGCGGAAG

ATGGCATTGATCCATTGGATGAAGTTGTCGGGGGCAACTGTCAAAATTGTGCTGCAACACTCCAACTCACAACTAATGCC

ACAAGATTAGCAAGGTGGGATGCAAAGCTTGGATTCTTGAAAGCATCGAGCACTAAACGAAATCCAAAGGGGCGATTATT

GGTGAATCGAATCTCAGATGTGGTGGTTGGTGGGGGGAACATTCCAGCCATTCGGTTATTCGTRCAACGAGTATATCCTA

TGCTCTACTACGAAAAACGTGGGTGTTCTGATGATTYTGCCGACKTTTCTTGTTCCAAGCCTAGTGTCTTCACYGAACAA

GAAGAAGACAGTAGGCGCAGAGAGTTCGAAGCGAGAAAGCTTCGAGCGATAGAAAAGGTGACTGAAAAGATCCAAGCAGA

GATTGAAAAGGTATGTATATATAGAAGCTTTTGCRTTATGTTGTAAARCATSAAATCCCGTCTAATTTWAAAAAATCCAA

TYTATTTTTGGTAGGAGGTCGACGAGGGTTGTCCAGAAGTCTGGAAACAAATGATGGGCAGTTCAACTCCCGAAGAGACT

TTCGAATCTTTTAGCAATCACGACAAAGAAGTAATTTCTAGATGGAGGGACCAACGCACTTCTTTGGTAAACCGTAGGGT

CAGAGAAGAAGTTGAGTCCGAGCTAGAGAYACAACCATCACTCATCCGCGAAAGCACAAGTTTTCTGCGAGTCAAGGTGT

ACAGCATCAATCCAAAAAGTTCTTGTAACGAAAGTGCGACGCTGACTATCTGGCAACCCACCGAAGATCAGTTGRGTTTT

CTCACAGAAGGCACGTCCGTGGAGTTTCACAACTTGGCCGTTCGAGAATCAAAAAGCTAYGATGGCACTYTGCAGCTTGT

TGCAAACAACCGGACAGTTGTTGAACCATTTTCATTTGATGCATSTTCACTCGCSGAGAAAATTGGATTTCGACAGCGCC

AATTCTTGAGTATGTTTCAAGTGCATGCACTTGCGCATGAAACGGTAGGCAAGCGATCCAGGACCAAAACCAGCGTTGAT

TTCGACGTAGTGGGAGTCCAGGTYCATGTTGTTCCACGTACCAACTCATCGGATGAGGTTATTTTCTACATATCCGATGA

AACAAACCTCGTTCTACGAATTCATTGTAGAAATCCACCCTCTGCACTGAAGACGCTAATTTTGGCCGAAAAACAATCCT

TTCMGTCCTATGGTATGCGGGATCTATCCATCCGTTCGTTCGACTACGAGAAGCAATGTGCAGTTGCCGAGTTCAGAGAG

ACATCTAGCGTTGTCCTGACAAACAAGCGTTTAGAAAATCTGGGGAAATGGGCTTCATCGTCAGCGCACAACGAGTTGCA

CCAGATCACGGCCTACCTCAATGCAGGYCTTCCTATTTGGGAACAGGAGTGCAATGAGAAGACGTACTTAGGATATGCAA

TAGGGCTCAGGTGCGAAAGCGTCGAAAATGTATTCATCATTGCAGATTGCTGTGGTCAGGGGACAAATGAATGGAAGCTT

CCCGTCCATGTTTTGCAGCAGATGATATCCTCGATTTCAGAAGAGAATCTTCGAGTGACTCTTTGTCCGGACGAGGAAGA

CTTAATTGCAAAACTAGGGAGGATAGGTTCCATTCTTCGAGCAAGAGGCGTTCTTTGGCAATTTCAGATCTCGTCGCAGT

CCGAATCGGTTGTTTACAGCGCCACCAAGGCAAGCAAGACCAACGTTGGTCACATTTACGCGGCATTACAGCAGTAGTTT

AAAAAGTAAGGATGATGCTATTTTCTATC
