# Supplementary material for: Correction to: Identification of the meiotic toolkit in diatoms and exploration of meiosis-specific SPO11 and RAD51 homologs in the sexual species Pseudo-nitzschia multistriata and Seminavis robusta
Source: BMC Genomics. 2019 Jul 5;20:544. doi: 10.1186/s12864-019-5942-4 (PMC6610888; doi:10.1186/s12864-019-5942-4)
Supplement: Supplementary file 1 — HMM profiles used for Hmmsearch. (ZIP 1469 kb) [file 12864_2019_5942_MOESM1_ESM.zip › Additional file10/HMM profiles_notes.pdf]

Database search to confirm absence of meiotic proteins

An alternative approach to confirm absence of the certain genes involved search using HMMER program (HMMER3.1b). Protein sequences (covering major taxa from different eukaryotic groups) for each of these gene families from NCBI protein database were downloaded and aligned using MUSCLE program [115]. Further, HMM profiles were generated using default settings of HMMBUILD command in HMMER software and these HMM profiles for respective gene families were then used to search against respective diatom protein databases (already mentioned in materials and methods section). HMM profiles are provided in additional file 10.

Following are the names of HMM profiles

Sr No.	Name of the protein	Name of HMM profile
1	Syn1/Rec8	SYN1_REC8_HMM
2	Spo11-1	SPO11-1_HMM
3	Xrs2/Nbs1	NBS1_HMM
4	Hop1/Asy1	HOP1_HMM
5	Red1/Asy3	RED1_ASY_HMM
6	Zip1/Zyp1	Please see the table below
7	Zip2	Please see the table below
89	Zip3/Hei10	Please see the table below
10	Zip4	Please see the table below
11	Dmc1	DMC1_HMM
12	Hop2	HOP2_HMM
13	Rad51-D	RAD51_HMM
14	Xrcc2	XRCC2_HMM
15	Mlh2	MLH2_HMM
16	Mlh3	MLH3_HMM
17	Mms4/Eme1	MMS4_HMM
18	Mus81	MUS81_HMM
19	Brca1	BRCA1_HMM

1. Notes for proteins related to synaptonemal complexes

As already mentioned in the main text proteins related to synaptonemal complex, ZIP proteins, are divergent among plants animals and fungi. Therefore separate HMM profiles were generated for plant homologues, animal homologues and fungal homologues.

Name of the protein	HMM profile for plant homologues	HMM profile for animal homologues	HMM profile for fungal homologues
Zip1/Zyp1	ZIP1_ZYP1_Plants_HMM	ZIP1_SYCP1_Animals_HMM	ZIP1_fungi_HMM
Zip2/Sycp2	No detectable homologues in plants	ZIP2_SYCP2_Animals_HMM	ZIP2_fungi_HMM
Zip3/Zyp3	ZYP3_HEI10_Plants_HMM	ZIP3_SYCP3_Animals_HMM	ZIP3_fungi_HMM
Zip4/Spo22	ZIP4_SPO22_Plants_HMM	ZIP4_TEX11_Animals_HMM	ZIP4_SPO22_fungi_HMM
